# Convergence in LINE-1 nucleotide variations can benefit redundantly forming triplexes with lncRNA in mammalian X-chromosome inactivation

**DOI:** 10.1186/s13100-019-0173-4

**Published:** 2019-07-30

**Authors:** Yoko Matsuno, Takefumi Yamashita, Michiru Wagatsuma, Hajime Yamakage

**Affiliations:** 1 Division of Clinical Preventive Medicine, Niigata University, Niigata, Japan; 2 Laboratory for Systems Biology and Medicine, Research Center for Advanced Science and Technology, The University of Tokyo, Tokyo, Japan; 3 ULVAC Inc., Kanagawa, Japan; 4 Satista Inc., Kyoto, Japan

**Keywords:** LINE-1, Long noncoding RNA, X-chromosome inactivation, Xist RNA, XIST RNA, Rsx RNA, Hoogsteen triplex, Reverse Hoogsteen triplex, MC simulation

## Abstract

**Background:**

Associations between X-inactive transcript (Xist)–long noncoding RNA (lncRNA) and chromatin are critical intermolecular interactions in the X-chromosome inactivation (XCI) process. Despite high-resolution analyses of the Xist RNA-binding sites, specific interaction sequences are yet to be identified. Based on elusive features of the association between Xist RNA and chromatin and the possible existence of multiple low-affinity binding sites in Xist RNA, we defined short motifs (≥5 nucleotides), termed as redundant UC/TC (r-UC/TC) or AG (r-AG) motifs, which may help in the mediation of triplex formation between the lncRNAs and duplex DNA.

**Results:**

The study showed that r-UC motifs are densely dispersed throughout mouse and human Xist/XIST RNAs, whereas r-AG motifs are even more densely dispersed along opossum RNA-on-the-silent X (Rsx) RNA, and also along both full-length and truncated long interspersed nuclear elements (LINE-1s, L1s) of the three species. Predicted secondary structures of the lncRNAs showed that the length range of these sequence motifs available for forming triplexes was even shorter, mainly 5- to 9-nucleotides long. Quartz crystal microbalance (QCM) measurements and Monte Carlo (MC) simulations indicated that minimum-length motifs can reinforce the binding state by increasing the copy number of the motifs in the same RNA or DNA molecule. Further, r-AG motifs in L1s had a similar length-distribution pattern, regardless of the similarities in the length or sequence of L1s across the three species; this also applies to high-frequency mutations in r-AG motifs, which suggests convergence in L1 sequence variations.

**Conclusions:**

Multiple short motifs in both RNA and duplex DNA molecules could be brought together to form triplexes with either Hoogsteen or reverse Hoogsteen hydrogen bonding, by which their associations are cooperatively enhanced. This novel triplex interaction could be involved in associations between lncRNA and chromatin in XCI, particularly at the sites of L1s. Potential binding of Xist/XIST/Rsx RNAs specifically at L1s is most likely preserved through the r-AG motifs conserved in mammalian L1s through convergence in L1 nucleotide variations and by maintaining a particular r-UC/r-AG motif ratio in each of these lncRNAs, irrespective of their poorly conserved sequences.

**Electronic supplementary material:**

The online version of this article (10.1186/s13100-019-0173-4) contains supplementary material, which is available to authorized users.

## Background

A 17–19-kb mouse Xist (X-inactive specific transcript)/human XIST long noncoding RNA (lncRNA) plays a pivotal role in a process called X-chromosome inactivation (XCI), a mechanism by which mammalian dosage compensation for the X-linked genes occurs between the sexes. In early female embryogenesis, Xist/XIST RNA, expressed from the future inactive X chromosome (Xi), spreads in *cis* and associates with the Xi, as a result of which gene silencing and a repressive chromatin state are achieved [1–3]. Despite the functional similarities between Xist and XIST RNAs, the gene sequences were found to be only 66% similar for exons [4], a finding which is in contrast with the overall 85% identity for 1,880 rodent/human mRNA sequence pairs [5]. A 24-kb Rsx (RNA-on-the-silent X) RNA, a lncRNA involved in marsupial XCI, exhibited no homology with Xist/XIST RNAs, although it showed similar functions, such as association with the entire Xi chromatin and subsequent induction of gene silencing in *cis* [6]. This enigmatic sequence−function relationship could be linked to elusive features of the Xist/XIST/Rsx RNA−chromatin association, where no specific sequence motifs or factors involved in the interactions have been identified [7, 8].

Intriguingly, in a series of *Xist* deletion analyses, multiple low-affinity or functionally redundant, but unspecified, sequences were predicted to be dispersed throughout the Xist RNA, by which Xist RNA could cooperatively associate with chromatin [9]. With regard to DNA elements, long interspersed nuclear element 1 (LINE-1, L1) repetitive sequences have been proposed as candidate way stations for Xist/XIST RNA spreading on the mouse and human X chromosomes, a concept known as the Lyon repeat hypothesis [10], which is an extension of the Gartler–Riggs model [11]. This hypothesis, albeit still controversial, has been supported by multiple evidences based on cytological and computational studies: (1) increased L1 content in the mouse and human X chromosomes compared to the autosomes [12, 13]; (2) the non-random distribution of L1s on the X chromosome, which is consistent with the locations of the chromatin segments harboring the genes subject to or escaping XCI [12, 14]; (3) a strong positive association between the spreading efficiency of XCI signals and the L1 density, which was observed in X-autosome translocations or in *Xist* transgene-inducible systems [15–17]; and (4) the preferential enrichment of multiple oligomers in L1 elements that could predict the expression status of X-linked genes [18]. Furthermore, RNA fluorescence in situ hybridization (RNA FISH) revealed the possible involvement of both silent (or truncated) and actively expressed L1s in the process of XCI: the former created a repressive nuclear compartment which was initially coated with Xist RNA, while the latter contributed to further local spreading of XCI [19]. On the other hand, high-resolution mapping studies of Xist RNA localization produced results different from those of the works supporting the Lyon repeat hypothesis: Xist RNA did not localize to any specific sequences [20, 21], but to gene-rich regions as early domains, showing a strong negative association with L1 elements, and subsequently to gene-poor regions as late domains, to which Xist RNAs bind only weakly [21]. Although Engreitz et al. [20] revealed that Xist RNA spreading on a 150-Mb scale is mediated by dynamic organization of the three-dimensional architecture of the Xi, both of two key components—spatial proximity and sequence specificity—would be required for proper localization of Xist RNA to specific target sites [20, 22].

L1 elements have been identified in all mammalian species, including both metatherians (marsupial mammals) and eutherians (placental mammals). However, the X chromosome of the marsupial opossum, unlike that in eutherians, does not show L1 enrichment compared to autosomes [14, 23], a finding which is, according to the Lyon repeat hypothesis, consistent with the observation of incomplete XCI in marsupials [24, 25]. Thus, it can be considered that, despite the dissimilarity in both the L1 contents in the X chromosome and the Xist/XIST/Rsx RNA sequences, epigenetic features of the XCI process are conserved across metatherians and eutherians [6, 24, 26].

In recent years, it has been recognized that lncRNAs achieve regulatory diversity and specificity through modular combinations with other biomolecules such as RNA, DNA, proteins, and small molecules [7, 27–29]. An example of a protein involved in lncRNA–protein–DNA modules is hnRNPU (heterogeneous nuclear ribonucleoprotein U)/SAF-A (scaffold attachment factor A), which can bind to both Xist RNA and DNA, and is required for Xist RNA localization [30]. Also, the RNA–dsDNA (double-stranded DNA) triplex is another module, which has been thought to serve as a guide for noncoding RNA to sequence-specific DNA targets, with examples such as pRNA (promoter-associated RNA, ~ 90–100 nucleotides) and MEG3 (maternally expressed gene 3) lncRNA having been reported [31, 32]. However, it has been pointed out that no direct evidence of triplex conformation and their functions in living cells has been provided because of the transient and dynamic nature of these structures, and the limitations of current technical capabilities, hence more careful evaluation of experimental results is required [33–36].

**Fig. 1:**
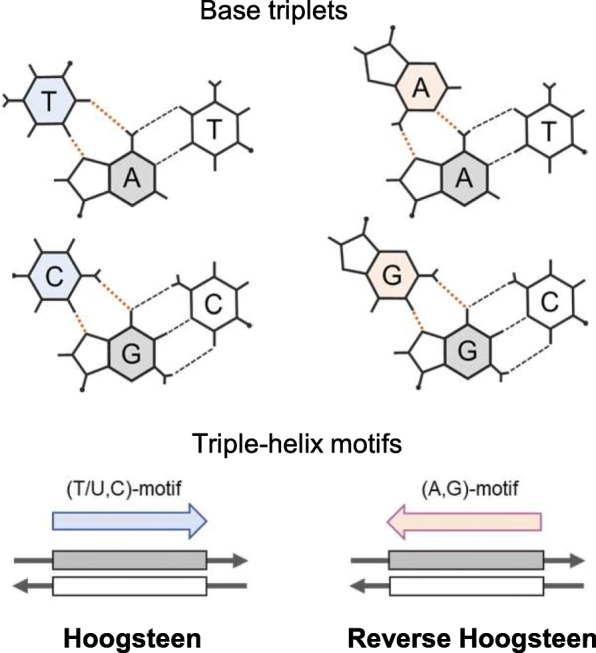
Schematic representation of triple helices. Canonical base triplets formed in pyrimidine and purine triple-helix motifs through Hoogsteen (*Left*) and reverse Hoogsteen (*Right*) hydrogen bonding (red dotted lines) are depicted. Parallel and antiparallel triplex formations are shown at the bottom

Keeping these points in mind, here we report examples of short-sequence motifs that were designed to fulfill the redundancy in forming triplexes between Xist/XIST/Rsx RNA and duplex DNA, especially the L1 elements on the mouse, human, and opossum X chromosomes. Using bioinformatics, computer simulations, and in vitro analyses of circular dichroism (CD) spectra and quartz crystal microbalance (QCM) measurements, we identified characteristic features of the short motifs, which are abundantly localized in both Xist/XIST/Rsx RNAs and L1s of the three species. We also showed convergent patterns of variation in L1 base composition, which results in the conservation of the short motifs in L1s, thereby contributing to the retention of their ability across mammalian species to redundantly form triplexes with the lncRNAs involved in XCI, these occurring through Hoogsteen (the third strand pyrimidine in parallel orientation) or reverse Hoogsteen (the third strand purine in antiparallel orientation) base-pairing (Fig. 1; [37]).

## Results

### Short motifs that can mediate triplex formation are densely dispersed throughout XIST/XIST/Rsx lncRNAs

**Fig. 2:**
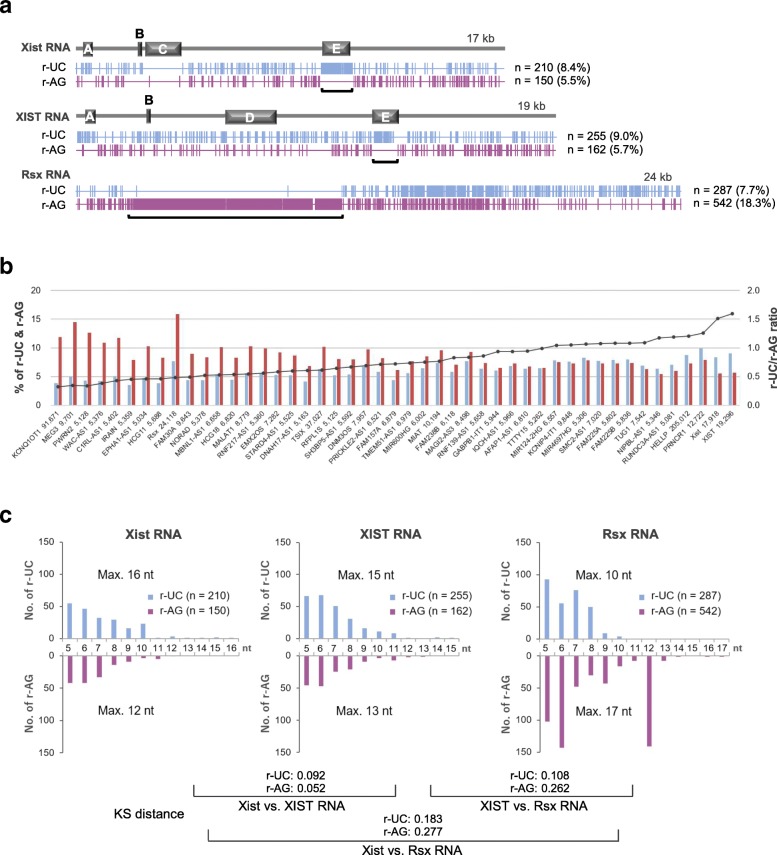
Redundant-UC/redundant-AG (r-UC/r-AG) motifs in Xist/XIST/Rsx RNAs and human long noncoding RNAs (lncRNAs), and motif–length distributions. **a** Distributions of r-UC/r-AG motifs dispersed in Xist/XIST/Rsx RNAs, with alignments of Xist/XIST RNA tandem repeats (A–E repeats). Vertical light blue and red bars represent the sites of r-UC and r-AG motifs, respectively. The total numbers and proportions of the motifs in the lncRNAs are indicated. Bold square brackets are under the regions of singly clustered r-UC or r-AG motifs. **b** r-UC/r-AG motifs in lncRNAs longer than 5,000 nucleotides with known functions. The left Y-axis shows the percentage of redundant-UC (r-UC) (light blue) and redundant-AG (r-AG) (red) motifs in 46 human lncRNAs and Xist/XIST/Rsx RNAs (listed below the X-axis with their length (in nucleotides)). The right Y-axis shows the r-UC/r-AG motif ratio (black line with dots) occupied in lncRNAs. **c** r-UC/r-AG motif–length distribution in Xist/XIST/Rsx RNA. The total number and the maximum length (Max.) of the motifs are indicated. Kolmogorov–Smirnov (KS) distance values are indicated at the bottom. nt, nucleotides

We defined short RNA motifs ≥5 nucleotides that may help to mediate triplex formation between Xist/XIST/Rsx RNA and duplex DNA as “redundant UC (r-UC) motifs”. Based on the features required for Hoogsteen triplex formation at neutral pH in vitro, these motifs should meet the following criteria [38]: (1) have at least three nucleotides between the first and last Us; (2) have no more than three Us in a row; and (3) have, at most, two Cs in a row. AG motifs complementary to these r-UC motifs (termed r-AG motifs) were also searched for under the same criteria by replacing U with A and C with G and changing criterion (3) to “have no limit to the number of consecutive Gs,” because consecutive Gs in r-AG motifs provide greater affinity for reverse Hoogsteen triplexes [39, 40]. Following these criteria, the distributions of r-UC/r-AG motifs were plotted along Xist/XIST/Rsx RNAs (Fig. 2a). A total of 210 sites containing r-UC motifs (8.4%) and 150 sites with r-AG motifs (5.5%) were observed in 17-kb Xist RNA, 255 r-UC sites (9.0%) and 162 r-AG sites (5.7%) in 19-kb XIST RNA, and 287 r-UC sites (7.7%) and 542 r-AG sites (18.3%) in 24-kb Rsx RNA. These motifs, with length ranges (in nucleotides) of 5–16/5–12 (r-UC/r-AG motifs) for mouse, 5–15/5–13 for human, and 5–10/5–17 for opossum, were dispersed throughout the lengths (see Additional file 1 for further information), producing specific regions where either r-UC or r-AG motifs were densely clustered. In the Xist/XIST RNAs of eutherian mammals, the regions with predominantly r-UC motifs corresponded to E repeats, one of the tandem repeats denoted A to E, all of which had previously been reported (see labeling Fig. 2a; [1, 2]), whereas in the Rsx RNA of metatherian mammals, the region with predominantly r-AG motifs accounted for one-third of the total length. The result with respect to Xist RNA is consistent with the view that functionally redundant sequences (or multiple low-affinity binding sites), which have little or no homology, are universally present along the entire Xist sequence, except for the C-repeat region [9].

To confirm whether these features are specific to the three lncRNAs involved in XCI, we investigated the frequency of occurrence and the occupancy of r-UC/r-AG motifs in 44 other human lncRNAs, which are 5,000 nucleotides or longer and well characterized (Fig. 2b; Additional file 2). Among the lncRNAs (*n* = 44) and the Xist/XIST/Rsx RNAs examined, the XIST/Xist RNAs had the two highest r-UC/r-AG ratios (%/%), with ratios of 1.60 and 1.51, respectively, whereas that of Rsx RNA was 0.48, the ninth lowest, with the highest frequency and occupancy of the r-AG motifs (22.5/kb, 15.9%). Interestingly, a 91-kb lncRNA, KCNQ1 opposite strand/antisense transcript 1 (KCNQ1ot1), the function of which is known to resemble that of Xist/XIST RNA, silencing the expression of neighboring cluster genes in *cis* over ~ 1 Mb in the Kcnq1 imprinting domain, possibly through long-range chromatin interactions [41, 42], showed the lowest r-UC/r-AG motif ratio, 0.32. Additionally, MEG3 variant 16 (9,701 nucleotides in length), a well-known candidate of triplex-forming lncRNA [32], showed the second lowest r-UC/r-AG motif ratio, i.e., 0.34. Among the 47 lncRNAs studied, there were only three lncRNAs with a ratio > 1.20, whereas there were 27 lncRNAs with a ratio < 0.80, meaning that lncRNAs with a predominance of r-AG motifs were more frequent, and the sequence composition of Xist/XIST RNA, with a predominance of r-UC motifs, appeared to be unique.

**Fig. 3:**
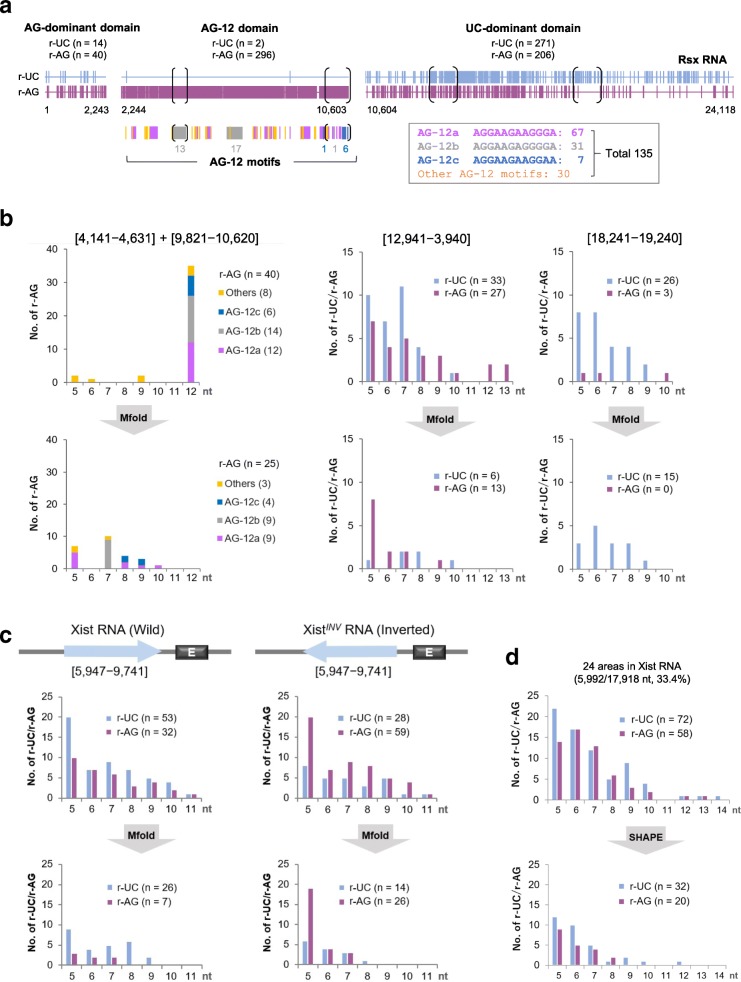
Number and length of redundant-UC/redundant-AG (r-UC/r-AG) motifs in Rsx/Xist RNAs available for triplex structures. **a** Three domains of Rsx RNA with r-UC/r-AG motif distributions are mapped. The distribution of three representative motifs (AG-12a, b, and c) and other AG-12 motifs is depicted at the bottom along the AG-12 domain. **b** r-AG or r-UC/r-AG motif–length distributions in four regions of Rsx RNA (indicated with black brackets in (**a**)). Upper and lower panels show the distributions before and after the analyses based on the predicted RNA secondary structures by the Mfold web server (unafold.rna.albany.edu/?/q=mfold), respectively. The numbers in square brackets indicate the positions of the sequences studied. **c** The inverted region in Xist^*INV*^ RNA allele (*Top*) and r-UC/r-AG motif–length distributions (wild-type vs. inverted; *Left* and *Right*) before and after Mfold analyses (*Center* and *Bottom*, respectively). E repeat is located directly downstream of the inverted region. **d** r-UC/r-AG motif–length distributions in Xist RNA based on SHAPE-MaP analyses (before and after in *Top* and *Bottom*, respectively) [44]. nt, nucleotides

We then studied the motif–length distribution of the r-UC/r-AG motifs in the Xist/XIST/Rsx RNAs. As Fig. 2c shows, Xist/XIST RNAs showed a similar length-distribution pattern for both the r-UC and the r-AG motifs (Kolmogorov–Smirnov (KS) distance = 0.092/0.052 (r-UC/r-AG motifs) < 0.100), whereas the pattern for Rsx RNA was distinct from those of the Xist and XIST RNAs (KS distance: 0.183/0.277 and 0.108/0.262 (r-UC/r-AG motifs), respectively), featuring many of the 12-nucleotide r-AG motifs (AG-12 motifs, *n* = 141 among 542 r-AG motifs in Rsx RNA), most of which were located within the r-AG motif-clustered region described above (*n* = 135 among 141). To explore further this result, we partitioned the Rsx RNA sequence into three domains, temporarily naming them as AG-dominant, AG-12, and UC-dominant domains (Fig. 3a). Among the 135 AG-12 motifs in the AG-12 domain that harbors 296 r-AG motifs, we selected three representative AG-12 motifs based on their high frequency, maximum number of consecutive Gs (“Gn”), and densely clustered distribution: aggaagaaggga (AG-12a: *n* = 67, G3), aggaagagggga (AG-12b: *n* = 31, G4), and aggaagaaggaa (AG-12c: *n* = 7, G2) (Fig. 3a; Additional file 1). The AG-12a and AG-12b motifs are a part of the conserved 34- and 35-base motifs in Rsx RNA, respectively, which were previously reported [6]. We then analyzed these AG-12 motifs as described below.

### The length of r-UC/r-AG motifs potentially available for triplex formation is rather short

The length of the r-UC/r-AG motifs available for triplex formation was investigated by analyzing single-strand parts (or loop structures) of the motifs in predicted RNA secondary structures. This kind of analysis would be useful for the detection of putative triple-helix sequence motifs as referred to by Buske et al. [34]. We focused this analysis on the following four regions in Rsx RNA: two regions in the AG-12 domain, one region containing the clustered AG-12b (*n* = 13, positions 4,141–4,631) and the other containing the clustered AG-12c (*n* = 6, positions 9,821–10,620); and the two regions in the UC-dominant domain showing different levels of the predominance of the r-UC motifs (positions 12,941–13,940 and 18,241–19,240) (black brackets in Fig. 3a). Figure 3b demonstrates a drastic reduction in both number and length of the r-UC/r-AG motifs, the length of which ranged from 5 to 9 or 10 nucleotides. For example, for the 14 AG-12b motifs and the 6 AG-12c motifs, as exhibited in the same graphs (two left-hand graphs), only seven nucleotides of each of the nine AG-12b motifs and eight or nine nucleotides of each of the four AG-12c motifs were located in the single-stranded loops of the secondary structures predicted by the Mfold web server (Additional file 3). For the region of positions 12,941–13,940, the total numbers of r-UC and r-AG motifs (r-UC:r-AG) were surprisingly reversed from 33:27 to 6:13, with length ranges of 5–10 and 5–9 nucleotides, respectively (two center graphs). However, those in the region of positions 18,241–19,240 changed from 26:3 to 15:0 with a length range of 5–9 nucleotides (two right-hand graphs).

We then performed the same analysis on the motifs located in the region of positions 5,947–9,741 in the *Xist* exon 1, which was used in a previous study as the inverted region of the Xist^*INV*^ RNA allele in the functional analysis of Xist RNA ([43]; top panel in Fig. 3c, this study) and was also the deleted region (corresponding to almost the same region in positions 5,650–10,044) in the ΔP construct for a targeted *Xist* mutation in the deletion analysis [9]. These two studies provided markedly different results—only Xist^*INV*^ RNA showed reduced affinity to chromatin, from which Senner et al. [43] suggested the involvement of the sequence in Xist RNA localization but by an unknown mechanism. As expected, in our analysis, the numbers of the r-UC and r-AG motifs (r-UC:r-AG) in this region (positions 5,947–9,741) for the wild-type and the inverted sequences were reversed (53:32 and 28:59, respectively) (Fig. 3c, two upper graphs); these, by further Mfold analysis, were reduced to 26:7 and 14:26, respectively, with a similar range of motif lengths (5–9 and 5–8 nucleotides, respectively) (Fig. 3c, two lower graphs). This local predominance of the r-AG motifs generated by sequence inversion may hamper triplex formation with genomic DNA by annealing with complementary r-UC motifs in the other regions of Xist RNA. This could be one of the explanations for the discrepancy in the results of the two studies described above. Our result also showed the shortness of the motif length available for triplex formation.

In addition, we investigated the number and length of the motifs in Xist RNA, using the secondary structures previously predicted by SHAPE-MaP (selective 2′-hydroxyl acylation analyzed by primer extension and mutational profiling) analysis [44], in which the 33 regions with well-defined structures were identified over the entire Xist RNA sequence. Among them, we used for our analysis the 24 regions (total of 5,992 nucleotides, representing 33.4% of the Xist RNA) that contained at least one r-UC or r-AG motif. The result demonstrated that the number of the r-UC and r-AG motifs available for triplex formation was 32 and 20, respectively, less than 50% of the total numbers in the region examined (72 and 58, respectively), and that the range of their lengths was reduced in both to almost 5–10 nucleotides (Fig. 3d). These data demonstrated that RNA folding structures predicted by both Mfold and SHAPE-MaP analyses resulted in similar short-length ranges of the r-UC/r-AG motifs, which resided in the loop structures of Rsx/Xist lncRNAs.

### The ratios of r-TC/r-AG motifs are opposite in L1s and L2s and the motif–length distribution patterns of L1s are similar, independent of their length and species

**Fig. 4:**
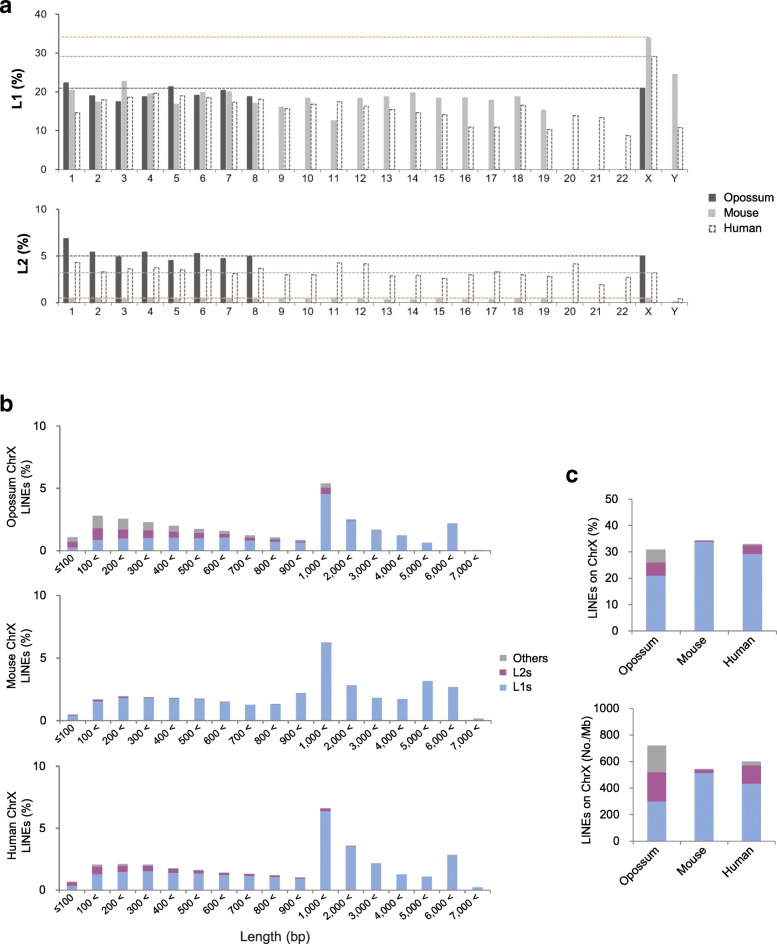
Long interspersed nuclear element (LINE) families in opossum, mouse, and human chromosomes. **a** Proportions of LINE-1s (L1s) and LINE-2s (L2s) in each chromosome of the three species. Opossum and mouse autosomes are 1 to 8 and 1 to 19, respectively. No data for opossum Y chromosome were available. **b** Proportions of LINE families of the indicated length ranges (bp) on the X chromosomes. The detailed data are shown in Additional file 4. **c** Proportions and frequencies of LINE families (L1s, L2s, and others) on the X chromosomes in the three species

Our preliminary analysis revealed the high frequency of r-AG (DNA) motifs in the LINEs randomly selected from the three species studied, namely, human, mouse and opossum, the frequencies being much higher than those of SINE (short interspersed nuclear repeats) (data not shown). Here, we first confirmed the proportions of L1s and L2s, the two main LINE families, on each chromosome in the three species (Fig. 4a). The proportion of L1s on the X chromosomes in the placental mammals mouse and human, as is known [12], was almost twice as high as the average on the autosomes (X:autosomes (%) = 33.8:18.2 in mouse and 29.1:15.4 in human), whereas in the marsupial opossum, the frequencies of L1s were almost equal for all chromosomes (X:autosome = 20.9:19.7). On the other hand, the proportion of the L2s was almost equal for all the chromosomes in each species (0.5:0.4 in mouse; 3.2:3.3 in human; 5.0:5.3 in opossum), revealing the lowest frequency in mouse and the highest frequency in opossum.

Since most L1s in genomes are 5′-truncated [45], some down to tens of nucleotides or less in length, we then examined the length distribution of LINEs on the X chromosome of the three species, with respect to the proportions of L1s, L2s, and other families (Fig. 4b). The results demonstrated that L2s tended to be shorter when compared with L1s, according to which L2s were not found longer than 2,000 bp in mouse or 3,000 bp in human or opossum (Additional file 4). Because the L2s are short, the proportion of L2 sequence becomes low, as observed in the opossum and human X chromosomes, which have a relatively high number of L2s (Fig. 4c).

**Fig. 5:**
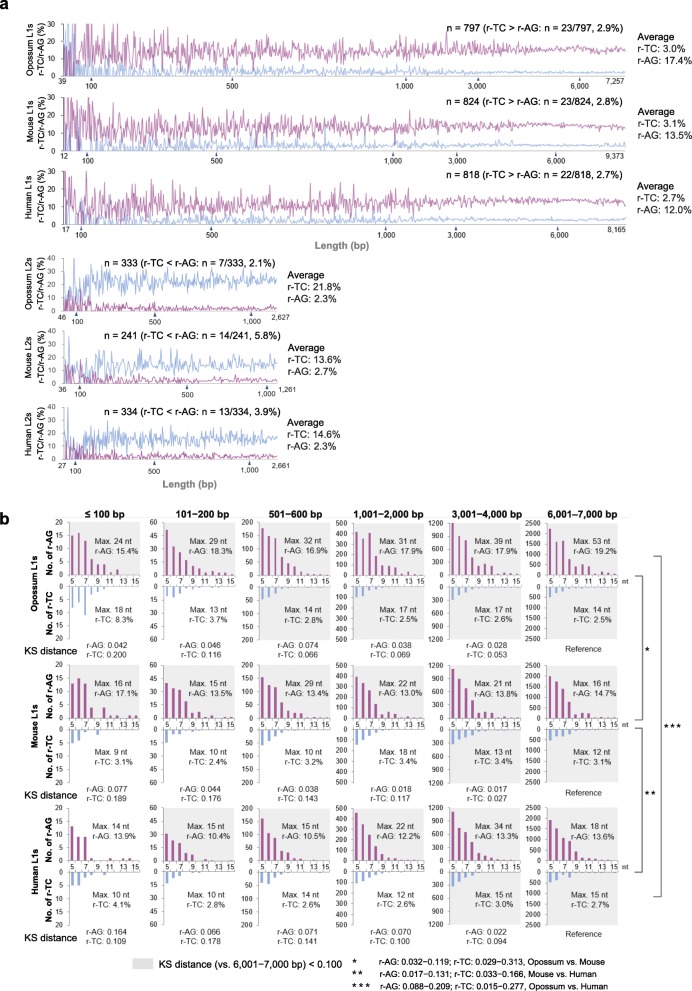
Redundant-TC/redundant-AG (r-TC/r-AG) motifs in LINE-1s (L1s) and LINE-2s (L2s) on opossum, mouse, and human X chromosomes. **a** Proportions of r-TC (light blue)/r-AG (red) motifs occupied in randomly selected L1s (see Additional file 5) and L2s of various lengths on the X chromosome in the three species. The total numbers and proportions of the L1s and L2s and the average proportions of the motifs are indicated at the top and right of each graph, respectively. **b** r-TC/r-AG motif–length distributions in L1s of the indicated length ranges (bp) obtained from (**a**). L1s ≤100 bp of the three species and human L1s of 101–200 bp were fewer than 50 in number because of the lack of both r-UC and r-AG motifs. Maximum length (Max.) and average proportion of the r-TC/r-AG motifs in the L1s are indicated in each graph. Kolmogorov–Smirnov (KS) distance values (vs. 6,001-7,000 bp) are indicated at the bottom and the graphs with KS distance < 0.100 are dark-colored. nt, nucleotides

Next, we explored the proportions of r-TC/r-AG motifs in the L1s and L2s of various lengths on the X chromosomes, which were randomly selected for each length range in the three species: 50 each for L1s (see Additional file 5 for the detail of L1 subfamilies) and 30 each for L2s of the same length ranges as in Fig. 4b: every 100 bp up to 1,000 bp and every 1,000 bp above 1,000 bp, for L1s up to a length exceeding 6,000 bp. Such longer L1s (> 6,000 bp) have been known to acquire an additional sequence through a process known as 3′ transduction [46–48]. The ranges of the L1 lengths analyzed, therefore, were 39–7,257 bp in opossum (*n* = 797), 12–9,373 bp in mouse (*n* = 824), and 17–8,165 bp in human (*n* = 818), whereas the range of the L2 lengths were 46–2,627 bp in opossum (*n* = 333), 36–1,261 bp in mouse (*n* = 241), and 27–2,661 bp in human (*n* = 334) (Fig. 5a). For the L1s, with a few exceptions (representing 2.7–2.9% of the L1s), the r-AG motifs occupied more space in the L1s than did the r-TC motifs, regardless of L1 length (17.4, 13.5, and 12.0% for r-AG motifs and 3.0, 3.1, and 2.7% for r-TC motifs on average in opossum, mouse, and human, respectively). Contrary to the L1s, however, for the L2s, with relatively few exceptions (representing 2.1–5.8% of the L2s), the r-TC motifs occupied more space than did the r-AG motifs (2.3, 2.7, and 2.3% for r-AG motifs and 21.8, 13.6, and 14.6% for r-TC motifs on average in opossum, mouse, and human, respectively). For the other families such as L3s (also known as CR-1) and RTEs (retrotransposable elements), which were present at relatively high frequencies in opossum [49], the occupancy by r-TC/r-AG motifs was similar to the L1s but contrary in the L2s (see Additional file 6).

**Fig. 6:**
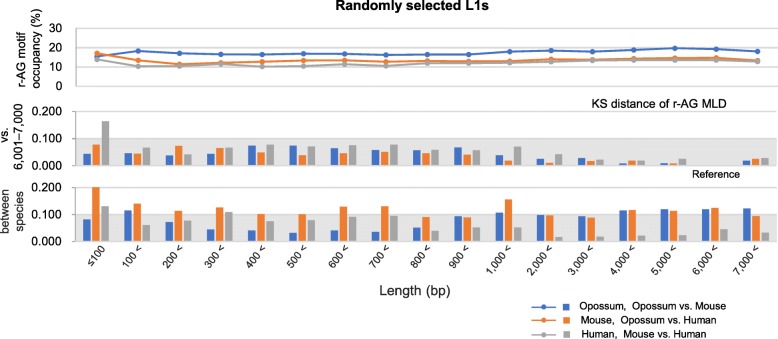
Restricted variations in length-distributions and occupancies of redundant-AG (r-AG) motifs in randomly selected L1s. Dark areas of Kolmogorov–Smirnov (KS) distance < 0.100 indicate high similarities in terms of r-AG motif–length distribution (MLD) in L1s, showing the independence both of L1 length (*Center*) and the species (*Bottom*)

Figure 5b shows the motif length distribution in the L1s, with length ranges of ≤100, 101–200, 501–600, 1,001–2,000, 3,001–4,000, and 6,001–7,000 bp analyzed in Fig. 5a, demonstrating that the length-distribution patterns of r-AG motifs were similar (mainly 5 to 11 nucleotides in length), independent of L1 length (KS distances < 0.100, vs. L1s of 6,001–7,000 bp, except for human L1s ≤100 bp). Figure 6 summarizes all data of r-AG motifs in the sequences of the randomly selected L1s used in Fig. 5a. These results confirm the similarity in terms of r-AG motif–length distribution in all three species, which is independent of L1 length (center panel) and is found in these species (bottom panel; KS distance < 0.100, mouse vs. human except for L1s ≤100 bp; 0.017–0.209 for opossum vs. mouse or human). It should be noted that the number of consecutive Gs in the r-AG motifs defined for opossum LINEs is different from that defined for mice and humans (see Methods). Further, Fig. 6 shows that r-AG motif occupancy was similar and ranged from 10 to 20% for all length ranges of L1s of the three species (top panel). For shorter L1s, similarities in terms of motif occupancy and motif–length distribution were observed only when analyzed as L1 assemblies of defined length ranges. In Fig. 5a, when r-AG/r-TC motif occupancies were analyzed for individual L1s, a different feature was observed: shorter L1s corresponded to larger variations in r-AG/r-TC motif occupancies, which is common among the three species.

### High-frequency variations in the r-AG motifs in full-length L1s despite similar motif–length distribution

**Fig. 7:**
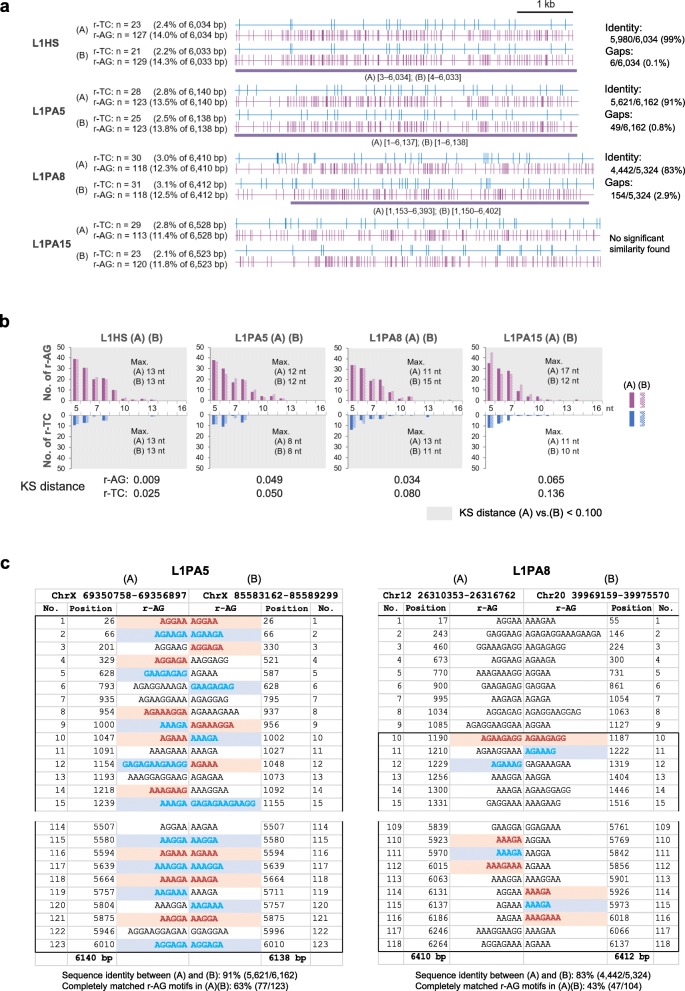
Mapping and length distributions of redundant-TC/redundant-AG (r-TC/r-AG) motifs in the paired LINE-1s (L1s) of representative subfamilies. **a** Alignments of the distribution of r-TC/r-AG motifs dispersed in paired human full-length L1s of the same subfamily. Bold purple underlines indicate the range of the regions assessed for sequence identities and gaps between (A) and (B), shown at the right of each alignment. **b** r-TC/r-AG motif–length distributions in the paired L1s analyzed in (**a**). Maximum length (Max.) of the r-TC/r-AG motifs and Kolmogorov–Smirnov (KS) distance values are indicated in and at the bottom of each graph, respectively. See Additional file 7 for all other representative subfamilies of the three species. nt, nucleotides. **c** The start and end parts of the alignments of r-AG motif sequences in the paired L1PA5 and L1PA8 (see Additional file 9 for the whole alignments). Alternative colored motifs (pink and light blue color from top to bottom) indicate completely matched motifs between (A) and (B). Their proportions to the total number of r-AG motifs are indicated under the alignments

To further investigate the details of the nucleotide composition of the L1s that showed similar motif–length distribution patterns, we chose a pair of L1s from each of the representative subfamilies from NCBI according to RepeatMasker annotation [45, 50, 51] that were comparable both in their lengths (> 6,000 bp) and in the number of r-TC/r-AG motifs: 22 subfamilies of human L1s, seven of mouse L1s, and five of opossum L1s (note that some subfamilies are < 6,000 bp in length, or < 5 in number, which are not enough to choose a pair). As shown in Fig. 7a, we aligned the plots of the r-TC/r-AG motif distribution for each L1 pair, (A) and (B) (see Additional file 7 for all the other L1 pairs). The plots were consistent between the paired younger L1s with high sequence identity, such as L1HS (99% with gaps of 0.1%); however, the plots were quite different between the paired older L1s with lower sequence identity, such as L1PA8 (83% with gaps of 2%). Nonetheless, all the human paired L1s with the exception of L1PA12 showed similar r-AG motif–length distribution (KS distance < 0.100), even for the L1s with no significant similarities in sequence (L1PA15, L1 PB4, and L1MA3, searched by BLAST) as shown in Fig. 7b (see Additional files 7 and 8 for details and other two species). We then aligned the r-AG motif sequences of the paired L1s to explore motif identities (Fig. 7c; see Additional file 9 for whole alignments and further examples).

**Fig. 8:**
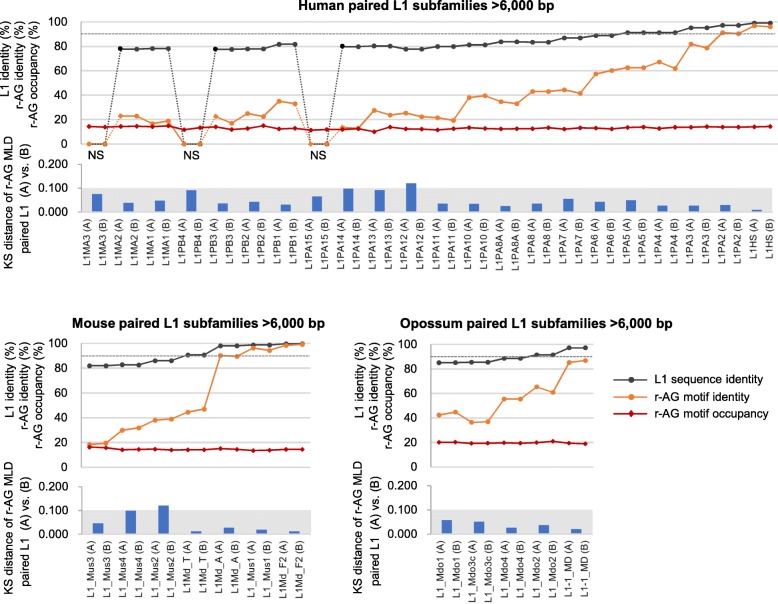
Restricted variations in length-distributions and occupancies of redundant-AG (r-AG) motifs in paired L1 subfamilies. L1 sequence identities are plotted for each of the paired L1s (having the same value) with black lines with dots in the graphs. Dark areas of Kolmogorov–Smirnov (KS) distance < 0.100 indicate high similarities in terms of r-AG motif–length distribution (MLD) of (A) and (B). Contrastingly, r-AG motif identities (orange lines with dots) show much larger variations (< 13–99%) (see Additional file 10 for the details). NS, no significant sequence similarity

Figure 8 summarizes all the results for paired L1s of the three species and their harbored r-AG motifs. In particular, when compared with the changes in L1 sequence identities, the r-AG motifs at the corresponding sites of the paired L1s showed a larger difference in base composition, which could be attributed to transition, transversion, or deletion/insertion mutations (see Additional file 9). The L1 pairs with < 90% identities had r-AG motif identities of < 60% across the three species, some of which (within the 77–82% sequence identity range) had now < 20% motif identities (Additional file 10). Moreover, such extensive multiple substitutions, while causing changes in length of r-AG motifs, or even loss or generation of the motifs, exclusively resulted in similar r-AG motif occupancies (10.1–15.0% for human; 13.5–16.3% for mouse; 18.9–20.9% for opossum paired L1s) and similar motif–length distributions (KS distances < 0.100 except for L1PA12 with 0.119 and L1_Mus2 with 0.120) (see Additional file 10). This observation along with the conservation of r-AG motifs even in shorter L1s suggests the existence of convergence in L1 base composition across the three species, which would assist in retaining the ability to provide triplex-forming target sites in L1s to Xist/XIST/Rsx lncRNAs.

### CD spectra and QCM analyses confirm weak triplex-forming interactions via r-UC/r-AG motifs

To confirm the predicted formation of DNAs–RNA triplexes via short motifs and to examine their binding affinity in vitro, we performed circular dichroism (CD) spectroscopy and quartz crystal microbalance (QCM) measurements, using dsDNA oligos (27–31 mer) and RNA oligos (26–27 mer), some of which were designed from L1s of the three species, and Xist/XIST/Rsx RNAs. CD spectroscopy is a technique for investigating conformational properties of molecules [52], whereas QCM can quantitatively evaluate interactions (by measurement of the dissociation constant, *K*_*d*_) between molecules at the nanogram level in solution [53]. As listed in Table 1, except for the two binding controls (#21: full-length having a perfect 27-bp match, #22: having 0-bp match), 20 samples were classified into four types of combination, exhibited as H (Hoogsteen) or rH (reverse Hoogsteen) (RNA, dsDNA bp), according to the number (one or more (multi)) of the motifs in each RNA or dsDNA molecule: (I) single:single (#1–8); (II) single:multi (#9, #10); (III) multi:single (#11–17); (IV) multi:multi (#18–20).

**Table 1:**
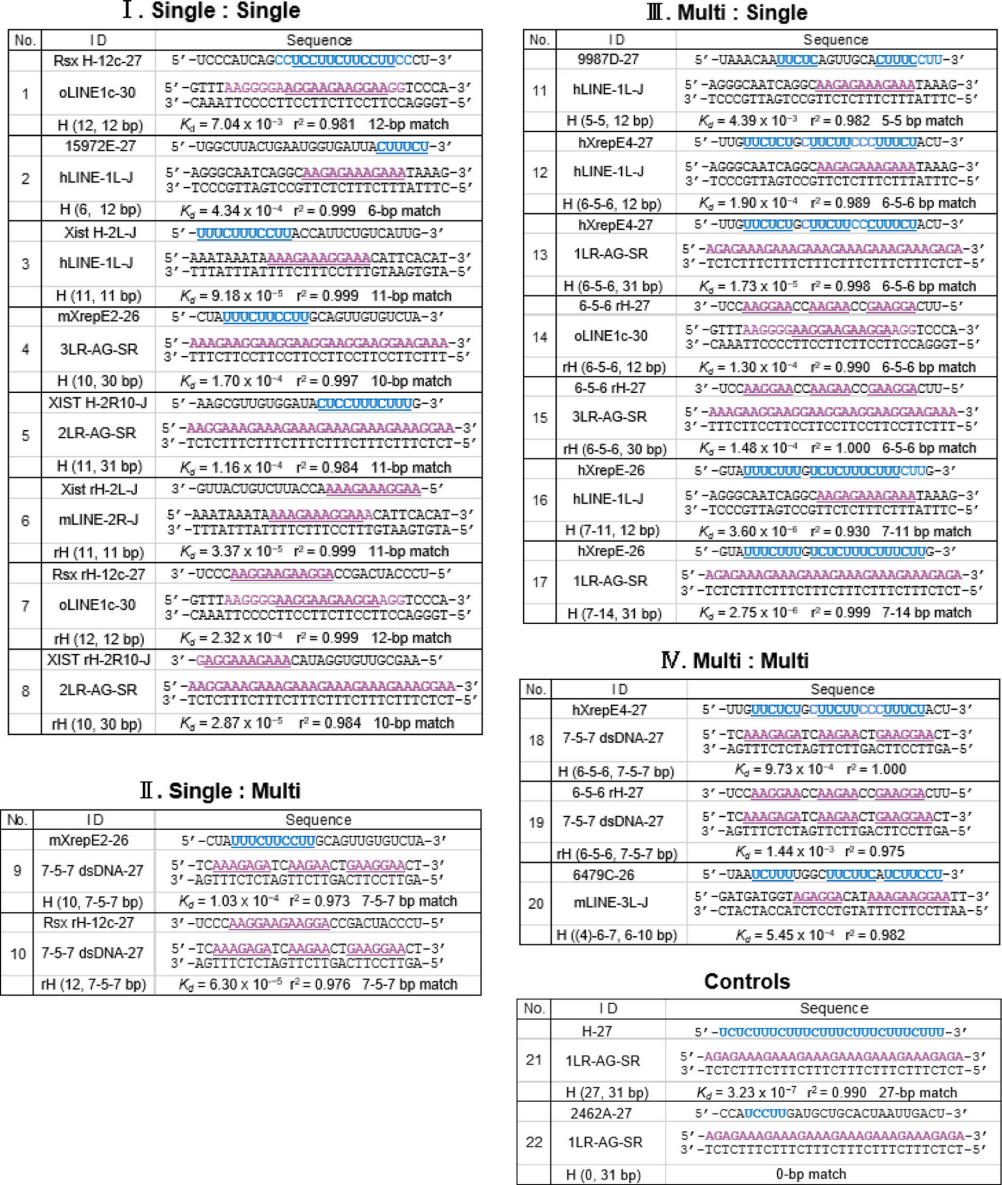
Redundant-UC (r-UC) or redundant-AG (r-AG) motifs in RNA and dsDNA oligomers

r-UC and r-AG motifs are shown in light blue and red, respectively. Underlined and bold colored nucleotides can form Hoogsteen (H) or reverse Hoogsteen (rH) base-pairing. *K*_*d*_, dissociation constant

**Fig. 9:**
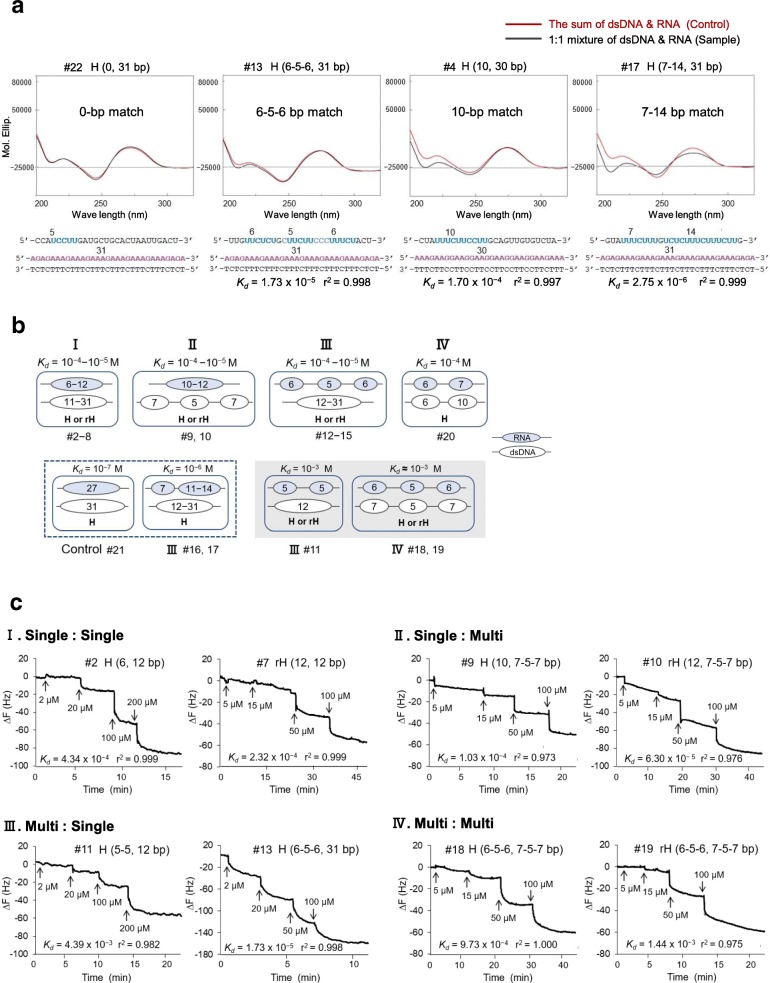
Circular dichroism (CD) spectra and quartz crystal microbalance (QCM) analysis for dsDNA–RNA triplex formation. **a** CD spectra of 1:1 mixtures of paired samples are shown in black. The sums of individual CD spectra of paired samples for controls are shown in red. *K*_*d*_ values were obtained from QCM measurements shown in Table 1. *K*_*d*_, dissociation constant. **b** Schematic description of four types of dsDNA–RNA triplexes (I, II, III, and IV), classified into three levels of *K*_*d*_ values (10^− 3^, 10^− 4^–10^− 5^, and 10^− 6^–10^− 7^ M) from Table 1. The numbers in ellipsoids indicate the length or length range of the motifs (in nucleotides) in RNA and dsDNA oligomers. H, Hoogsteen base-pairing; rH, reverse Hoogsteen base-pairing. **c** Time courses of frequency changes of dsDNA-immobilized QCM in response to addition of RNA. Two each of the representative results from triplex types I to IV are shown. Negative controls were measured without immobilized dsDNA and recorded almost no change in QCM frequencies (data not shown)

Of the four samples (#4, 13, 17, and control 22) with measurements of CD spectra, three, namely, #4, 13, and 17, showed an increased negative peak around 210 nm (Fig. 9a), which is a known characteristic of triplex formation [32, 54]. The changes observed in CD spectra were consistent with the numbers or the lengths of the Hoogsteen base-pairing motifs (from 0-bp match to 7-14 bp match). Although detection of triplex formation in sample #13 (6-5-6 bp match) was difficult using CD analysis, we calculated a greater binding affinity using QCM (*K*_*d*_ = 10^− 5^ M) compared with that for sample #4 (10-bp match) (*K*_*d*_ = 10^− 4^ M). This indicates that short motifs (< 10 nucleotides), as in sample #13, could reinforce binding affinity when interaction motifs were localized contiguously (in this case, three motifs in 27-mer RNA, each of which was ≥5 nucleotides), whereas optical CD measurements may require a minimum length of individual motifs to detect the conformational changes.

The results of the QCM analysis are summarized in Fig. 9b, wherein the magnitude of the *K*_*d*_ values is displayed at three levels, 10^− 3^, 10^− 4^ to 10^− 5^, and 10^− 6^ to 10^− 7^ M, for the four types of combination, including the control #21 (27-bp match). The course of their representative interactions is displayed in Fig. 9c. Although the control sample #21 showed the greatest binding affinity (*K*_*d*_ = 10^− 7^ M) as expected, such high affinity interaction would not occur for individual motifs in the case of Xist/XIST/Rsx RNAs because of their maximum motif length (15–17 nucleotides) (Additional file 1). For all types of combination, the measurements of most samples (14 out of 20) resulted in *K*_*d*_ values between 10^− 4^ and 10^− 5^ M under the following conditions: (1) a single motif of 10–11 nucleotides or longer in at least one molecule; (2) multi-motifs should include a motif of at least 6–7 nucleotides. For combination IV, however, it should be longer than 6–7 nucleotides in at least one molecule. One exception for this was sample #1 (H (12, 12 bp), *K*_*d*_ = 7.04 × 10^− 3^ M), the RNA sequence for which was designed from the sequence of Rsx RNA, having the same sequence as sample #7 but with a reversed AG-12c motif (uccuucuuccuu) (see sample #7, which contains a AG-12c motif and showed *K*_*d*_ = 2.32 × 10^− 4^ M) and from the L1 element containing its target motif (aggaagaaggaa) (Table 1). This suggests that binding affinities for forming triplexes could depend not only on motif lengths but also on motif sequences, including neighboring sequences. Triplex structures in the same sample (#1) could not be detected in CD spectra under the conditions examined (data not shown).

In our analysis, multi:multi combination IV could not demonstrate an effect of motif number (> 3) on the binding affinity due to a limitation of the length of RNA oligomers and yielded only the minimum *K*_*d*_ values of around 10^− 3^ M for both Hoogsteen (#18) and reverse Hoogsteen triplexes (#19) (Table 1; Fig. 9b).

Collectively, these data provided additional evidence for DNAs–RNA triplex formation via short motifs. Here, we focused on local interactions, assuming four types of combination via multiple (two or three) motifs, particularly of 5–7 nucleotides in length, considering their preferential usage on the basis of high occupancy. As shown in Additional file 11, 85.2–91.8% and 72.5–88.8% of the total motifs were in the three paired L1s (L1PA5, L1Md_T, and L1_Mdo4) and XIST/Xist/Rsx RNAs, respectively, when the r-TC (UC)/r-AG motifs, including overlaps, were counted. Several short motifs are found within longer motifs: for example, a 10-nucleotide motif, uuucuuccuu, contains four 5-nucleotide motifs (uuucu, uucuu, uuccu, and uccuu) as well as additional 6- and 7-nucleotide motifs. In other words, longer motifs in L1s, although fewer in number, would provide more chances for shorter target motifs to bind. This situation also reflects the results obtained from RNA-folding predictions of the local regions of Xist/Rsx RNAs, as shown in Fig. 3b, c, and d. These features may suggest that triplex interactions between Xist/XIST/Rsx RNA and genomic DNA, including L1s, could redundantly occur, depending on length or binding affinity of motifs encountered in the nucleus. These triplex-forming interactions via short motifs were weak, with the highest *K*_*d*_ value of 10^− 3^ M, but were rapid in reaction as observed in the time courses for both Hoogsteen and reverse Hoogsteen hydrogen bonding. This first observation of multiple-motif triplex formation in vitro revealed the potential of their characteristic molecular interactions for promoting binding between Xist RNA and DNA of retrotransposons, including L1s.

### MC simulation demonstrates possible DNA–RNA interactions via multiple weak binding sites

**Fig. 10:**
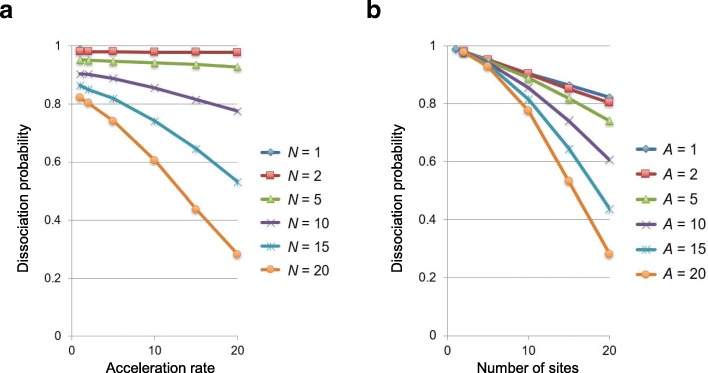
Monte Carlo (MC) simulations for dissociation probability (*P*_*diss*_) between DNA and RNA molecules via multiple weak binding sites. The *P*_*diss*_ values are given as a function of (**a**) acceleration rate, *A*, and (**b**) number of sites, *N*

To further investigate the cooperative effect of multiple RNA-binding sites, we conducted Monte Carlo (MC) simulations [55] of a kinetic model. Results are shown in Fig. 10, where the dissociation probability of RNA (*P*_*diss*_) is displayed as a function of the acceleration rate (*A*), with the number of binding sites (*N*) fixed (Fig. 10a). *k*_*on*_ = 0.002 and *k*_*off*_ = 0.2/step were set according to the weakest *K*_*d*_ value of 10^− 3^ M in the QCM experiment. For all cases, *P*_*diss*_ monotonically decreases as *A* increases, suggesting the importance of the effect of the linkers that connect the two adjacent sites. When *N* is small, most of the samples are in the dissociation state. However, where *N* = 20, the dissociation probability becomes lower than 30%. In Fig. 10b, the acceleration rate (*A*) is fixed and the dissociation probability is given as a function of *N*. Although *P*_*diss*_ can be regarded as a linear function of N for small *A* values, it decreases non-linearly for large *A* values. This is probably because the increase in *N* cooperatively enhances the binding state, increasing the entropy of the binding state. These simulation results indicate the possibility that the large number of binding sites drastically enhances the binding affinity of RNA and DNA, with the effect that linkers largely limit the search space for binding.

According to the reaction conditions simulated above, the high abundance of r-UC/r-AG motifs in both Xist/XIST/Rsx RNAs and L1 elements may cooperatively enhance their reciprocal binding. These simulations may remind us of the proximity-guided search model proposed by Quinodoz and Guttman [22], in which lncRNAs localize to target sites on genomes with a probability in proportion to its concentration with spatial gradient locally generated in the nucleus. In our model, however, there might be additional factors to be considered, one of which is the three-dimensional complex structures of the lncRNAs [56], which would determine distance between the two motifs, referred to here as linkers, and more conceivably, accessibilities around their target sites on genomes.

## Discussion

Triplex structures formed between DNAs and RNAs have been progressively recognized as potential effectors of biological activities [57]. A study on XIST RNA localization [58] demonstrated that abundant XIST RNA signals associated with Xi were not abolished following RNase H treatment, suggesting the involvement of DNA–RNA triplex interactions, which are known to resist RNase H digestion. Moreover, the existence of redundant features in the localization sequences on Xist RNA, as previously proposed [9], and findings from numerous studies supporting the Lyon repeat hypothesis [12, 16–19] led us to the speculation that short motifs (≥5 nucleotides long) can function as interaction motifs for forming triplexes between LINE elements and lncRNAs involved in XCI.

In addition to mouse and human LINEs and Xist/XIST RNA, we explored LINEs and Rsx RNA from the marsupial opossum, which uses different XCI mechanisms, such as non-random (or imprinted) XCI, and has a different genome composition, as shown by similar L1 occupancy on the X chromosome and autosomes (Fig. 4a, this study; [14, 23]). Owing to the generally low levels of the sequence conservation of lncRNAs [59] and species-specific LINE elements [60–62], this wider target of species in mammals (both eutherians and metatherians) may help in explaining the common features of interaction motifs required for Xist/XIST/Rsx lncRNA–chromatin associations in XCI. Using bioinformatics analyses, we demonstrated the features of short motifs shared by the aforementioned three species as follows: (1) densely dispersed r-UC/r-AG motifs throughout Xist/XIST/Rsx RNAs, which are fairly consistent with the pattern of functionally redundant sequences in Xist RNA [9]; (2) a particularly high/low r-UC/r-AG motif ratio compared with other human lncRNAs (> 5,000 nucleotides) with known functions; (3) densely dispersed r-AG and r-TC motifs in L1s and L2s, respectively, exhibiting r-TC/r-AG motif ratios opposite to one another; and (4) a similar length-distribution pattern of r-AG/r-TC motifs in L1s (statistically quantified using KS distance tests). This is regardless of the length/sequence similarities exhibited by the L1s and despite the high-frequency mutations in r-AG motif sequences, suggesting convergent patterns of variation in L1 base composition in mammalian species. Although not shared by Rsx RNA, this feature—a similar r-UC/r-AG motif–length distribution pattern despite their low level of sequence homology—was also recognized between mouse Xist and human XIST RNAs. This may be relevant to the importance of RNA secondary structures over DNA sequences with respect to function, which is a view proposed by Migeon [63] based on an experiment wherein a human *XIST* transgene could induce random X inactivation in male mice [64]. RNA secondary structures, such as stem–loop formation, are determined partly by the balance of the number of complementary r-UC/r-AG motifs and by the motif sequences.

We identified that an inversion of a part of the Xist RNA sequence used in a previous study [43] resulted in the formation of excess r-AG motifs of the complementary r-UC motifs. Such local predominance of r-AG motifs may lead to their annealing with r-UC motifs in the other regions of Xist RNA, resulting in disrupted association with chromatin mediated by r-UC motifs via formation of RNA–dsDNA triplexes and/or Xist RNA−protein complexes. For example, the E-repeat domain, where the r-UC motifs are most densely clustered (Fig. 2a), is localized immediately downstream of the inverted region and is believed to be bound by RNA-binding proteins, such as human antigen R (HuR) [44]. This scenario can explain the discrepancy in the results between two previous studies: the first study demonstrating the detrimental effect of Xist RNA sequence inversion on the localization to chromatin [43] and the other showing no effect of deletion of the same region [9]. This also suggests the importance of balancing the numbers (or appropriate ratios) of r-UC and r-AG motifs in Xist RNA.

A particularly high density of r-UC or r-AG motifs in Xist/XIST/Rsx RNAs may function through Hoogsteen or reverse Hoogsteen base-pairing, recognizing L1 elements as “way stations” where r-AG motifs are abundant. This concept largely conforms to the Lyon repeat hypothesis, which suggests that L1s can enhance the attachment of Xist RNA to the chromatin and facilitate the heterochromatization [15]. For L2 elements that have a predominance of r-TC motifs over r-AG motifs, the r-AG motifs on the opposite strand of the L2s, although small in number and/or short in length, may also function as Xist/XIST/Rsx RNA target sites in the genome; this is consistent with the results of a study by Tannan et al. [16] that indicated the involvement of L2s in addition to L1s in XCI spreading. In the recognition processes for the formation of triplexes, the density of motifs affects the occurrence of interactions, whereas the length of motifs affects the binding affinity. Based on the predicted Xist/Rsx RNA secondary structures, we demonstrated a decrease in the length range of the r-UC/r-AG motifs (to mainly 5–9 nucleotides), which are considered usable for triplex interactions. In addition, the length range of r-AG motifs in L1s is similarly limited to approximately 5–11 nucleotides in the three species.

Usually, high-affinity molecular interactions demonstrate *K*_*d*_ values of < 10^− 6^ M. In a previous study [40] on high-affinity triplex-forming oligomers, the authors described that these oligomers have a minimum length of 15 nucleotides, and showed that the *K*_*d*_ values for the in vitro interactions of 17–28 nucleotides were estimated between 10^− 7^ and 10^− 9^ M. This was expected to achieve a site-specific recognition of duplex DNA. Conversely, the *K*_*d*_ values we calculated for the dsDNA–RNA interactions via 2- or 3-combined short motifs (5–10 nucleotides each) in the oligomers ranged from 10^− 3^ to 10^− 5^ M for Hoogsteen as well as for reverse Hoogsteen base-pairings. Such weak affinities have been observed in biologically important molecular interactions, such as the interaction between sugar-binding proteins and complex carbohydrates expressed on cell surfaces [65].

In our study, QCM was able to detect molecular interactions between dsDNA and RNA oligomers with a highest *K*_*d*_ value of approximately 10^− 3^ M and visualize their binding reactions real-time, similar to previously reported studies dealing with different templates [66]. However, a limitation to the use of QCM lies in the length of tested molecules because QCM response is strongly damped by the liquid surrounding them, which is relevant to the viscoelastic properties of molecules [67, 68]. Furthermore, in our model where multiple binding sites mediate DNAs–RNA interactions, an accurate value of *K*_*d*_ cannot be determined from such a mode of interaction. To complement the in vitro experiments, we performed MC simulations and identified that several binding sites cooperatively decrease the dissociation rate with a moderate acceleration of site-binding. Here several mechanisms may cause this acceleration; one important mechanism is that once a binding site of noncoding RNA binds to DNA, the finite lengths of linkers that connect binding sites limit motion space of the other binding sites, thus enhancing the chance of encounter and increasing the binding rate. In our model, the high abundance of r-UC or r-AG motifs present in Xist/XIST/Rsx RNA and L1s on the X chromosome, may provide greater interaction opportunities for individual binding sites; however, they may still not have sufficient affinities for stable associations along the length, rather causing redundancy in their association. To preserve this characteristic triplex interaction across the mammalian species, the conserved motif–length distribution in L1s, with short and limited length ranges, may be an important factor, possibly being inherited as an innate feature of L1 elements.

Our study reports particularly high/low r-UC/r-AG motif ratios in Xist/XIST/Rsx RNAs, compared with those of other human lncRNAs (> 5,000 nucleotides) with known functions. Some lncRNAs with a very-low motif ratio, such as KCNQ1ot1 RNA, may also possess the ability to locally form triplex structures with duplex DNA. If the motifs have functional roles, the length of the lncRNAs could be crucial to the expression of this function, as addressed in the case of KCNQ1ot1 RNA [69]. LncRNAs need a larger space to harbor a number of sequence motifs, even if they are short in length. Additionally, the numbers of motifs available for triplex formation are limited due to three-dimensional folding structures of lncRNAs. Regarding L1s as targets that occupy approximately 20–30% of the X chromosomes, along with the non-random properties of L1 distribution [12, 14], r-AG motifs occupy such L1s at a frequency of approximately 10–20%, regardless of the length or sequences of these L1s in the three species. These genomic features and such particularly long RNAs may be crucial factors for facilitating the association of the lncRNA with the entire X chromosome. More importantly, this molecular interaction may be cooperatively involved in lncRNA–chromatin associations with RNA–protein–DNA interaction modules and three-dimensional nuclear organization, where the lncRNA can be guided to genomic DNA sites in a sequence-specific manner [7, 20, 22]. In such a scenario, the features of redundancy in Xist/XIST/Rsx RNA–chromatin associations may provide more advantageous circumstances for proteins in modules to exert their own functions in the surroundings. For instance, dissociation and relocalization of Xist RNA–polycomb repressive complex 2 (PRC2) complexes onto the Xi are serially processed through a hit-and-run model [70], leading to local nucleosome methylation by the enhancer of zeste homolog 2 (EZH2)—a PRC2 component. According to the “hit-and-run” model, a mechanism first proposed decades ago, a transcription factor binds transiently to genes (the “hit”) initiating transcription, and then vacates its binding sites (the “run”) [71]. Furthermore, based on a model proposed by Hall and Lawrence [72, 73], the expression of 5′-truncated L1s that are abundant in the X chromosome can be suppressed through their interaction with Xist RNA, which causes the loss of Cot-1 euchromatin-associated chromosomal RNAs (ecRNAs), followed by the induction of chromosome condensation.

Taken together, this novel triplex interaction via multiple short motifs could be seen as one of the mechanisms of Xist/XIST/Rsx lncRNA–chromatin association in XCI. This mechanism could be conserved across mammals by conserving r-AG motifs in L1s by convergence in nucleotide variations and by maintaining particular r-UC/r-AG motif ratios in the involved lncRNAs, despite their poorly conserved sequences. Future efforts will focus on characterizing the functions of short UC (TC)/AG-motifs as well as other triple-helix motifs such as (G, T)-motifs [37, 74] to further aid our understanding of the redundant mechanisms of lncRNA–chromatin associations in the XCI process.

## Conclusions

Chromatin-associated lncRNAs have been identified as crucial regulators of gene expression and epigenetic chromatin state [7]. In mechanistic studies of XCI, elusive features of the lncRNA–chromatin associations present a challenge in investigating their interaction sequences. Using a combination of bioinformatics, computational simulations, and analyses of CD spectra and QCM measurements in vitro, we have characterized r-UC (TC)/r-AG motifs (mainly 5–9 nucleotides in length) abundantly localized in both Xist/XIST/Rsx RNAs and L1s on mouse, human, and opossum genomes. These multiple short motifs in lncRNA and DNA molecules could be brought together to form Hoogsteen or reverse Hoogsteen triplexes, possibly cooperatively or in a redundant manner. This novel triplex interaction largely conforms to the Lyon repeat hypothesis. This study further illustrates two key characteristics of the genomic features: (1) convergent patterns of variation in L1 sequences, which may contribute to the conservation of the r-AG motifs in L1s of any length or base composition, providing similar motif–length distribution, and (2) particularly high or low r-UC/r-AG motif ratios in the Xist/XIST/Rsx RNAs compared to other human lncRNAs of > 5,000 bp with known functions, irrespective of their poorly conserved sequences. These characteristic features may help to retain the ability to form triplexes across mammalian species as an essential interaction for the Xist/XIST/Rsx lncRNA–chromatin associations in the process of XCI.

## Methods

### Characterization of short motifs in lncRNAs and LINEs in human, mouse, and opossum genomes

To investigate the features of short motifs, we used the reference sequences of XIST/Xist/Rsx (NR_001564/NR_001463/JQ937282), human lncRNAs (RNAcentral), and LINEs on the X and other chromosomes (NCBI hg38 for human/mm10 for mouse/monDom5 for opossum) according to RepeatMasker annotations. For performing the searches of the number, location, length-distribution, and ratio of r-UC (TC)/r-AG motifs, we used tailor-made software (DNAMotifFinder) that can set the search parameters (minimum length and number of consecutive nucleotides in indicated situations) according to the Hoogsteen or reverse Hoogsteen base-pairing rules [37, 38]. For mouse and human LINEs, the parameters were subject to the conditions suitable for Hoogsteen base-paring with XIST/Xist RNAs, which predominantly contain r-UC motifs, whereas, for opossum LINEs, the parameters were subject to the conditions suitable for reverse Hoogsteen base-paring with Rsx RNA, where r-AG motifs are predominant. For all lncRNAs, the parameters were separately set for r-UC and r-AG motifs, subject to the conditions for Hoogsteen and reverse Hoogsteen base-pairing, respectively. As an indicator of the difference in r-TC/r-AG motif–length distribution across species or L1 length-ranges, we used KS distance, which is defined as the maximum value of the absolute difference between two cumulative distribution functions [75]. Large differences between the two length-distributions result in high KS distance values.

### Analysis of motif lengths usable for triplex structures

Using the Mfold web server (unafold.rna.albany.edu/?/q=mfold), we obtained the secondary structures of the partial sequences of Xist RNA (positions 5,947–9,741 from the *Xist* start site) and Rsx RNA (positions 4,141–4,631; 9,821–10,620; 12,941–13,940; 18,241–19,240 from the *Rsx* start site). The sequence of each region contained at least one r-AG or r-TC motif and 50 nucleotides at each end. Among the secondary structures obtained from the AG-12 domain of Rsx RNA, we used a representative structure because of their marked similarities to each other, whereas, for an UC-dominant domain of Rsx RNA and Xist RNA, we used all the structures predicted by Mfold. We manually counted the number of nucleotides in the motifs located in hairpin or internal single-stranded loops. We also performed the same analysis for Xist RNA, using its secondary structures obtained by selective 2′-hydroxyl acylation and analyzed by primer extension and mutational profiling (SHAPE-MaP) analysis in the previous study [44].

### Frequencies of LINE families and nucleotide composition analysis of redundant AG (r-AG) motif sequences in paired L1s

The features of LINEs, such as families, subfamilies, and length, were based on RepeatMasker annotations. To investigate the sequence homology (percentage identities and gaps) between the paired L1s, we aligned the paired L1s using the Basic Local Alignment Search Tool (BLAST) provided by NCBI. For nucleotide composition analyses, the r-AG motif sequences of paired L1s were aligned in the order of nucleotide positions using DNAMotifFinder, and then the base compositions of the r-AG motifs were manually compared.

### Characterization of triplex formation by CD spectra and QCM analysis

For CD measurements, dsDNA and RNA oligomers (synthesized by GeneDesign) were separately dissolved in triplex-forming buffer (TFB, 10 mM Tris pH 7.5, 25 mM NaCl, and 10 mM MgCl_2_) at 2.2 μM for controls, and at 2.2 μM each for 1:1 mixed samples and equilibrated for 1 h at 30 °C, following the method of Mondal et al. [32]. CD spectra were recorded on a Jasco J-820 spectropolarimeter using a 1-mm cuvette containing 200 μL of solution incubated at 25 °C at a scan rate of 50 nm/min, and averaged over an accumulation of four spectra. The ellipticity was converted to the mean residue molar ellipticity based on the concentration of nucleotides in the sample. For QCM analysis, biotinylated dsDNA and RNA oligomers (GeneDesign) were dissolved in TFB at 50 μM and 0.2–1.0 mM, respectively. QCM measurements were performed with an AFFINIX QNμ (ULVAC, Inc., Japan). A gold-coated 27-MHz QCM plate mounted in a 500 μL-cell was washed twice by immersion in a 3:1 mixture of concentrated sulfuric acid (98%) and hydrogen peroxide solution (30%) for 5 min, followed by treatment with 100 μg/mL NeutrAvidin (Thermo Scientific) for 1 h at room temperature. Then, biotinylated dsDNAs were immobilized onto the QCM plate, followed by RNA injection. The frequency changes in response to dsDNA–RNA hybridization were monitored at 25 °C, and a kinetic parameter, dissociation constant (*K*_*d*_), was calculated using AQUA computer software. As a control, RNAs were added in TFB solution without immobilized dsDNA. We repeated the measurement procedure twice or three times for each sample and used the representative value.

### Theory and computational detail for MC simulation

To investigate how many binding sites cooperatively suppress the dissociation of RNA from DNA, we introduced a simple kinetic model. The model consists of *N* binding sites of RNA and the site *i* (1 ≤ *i* ≤ *N*) have a binding state (*Bi*) and dissociation state (*Di*). The rate constants of the transfer from *Di* to *Bi* and of the transfer from *Bi* to *Di* are denoted as *k*_*on*_ and *k*_*off*_, respectively. *A*s suggested in the experimental section of this paper and experiments of the bispecific antibody [76], when one site (*j*) binds to its target, the binding of another site (*i*) should be accelerated. This is mainly because the search space of site *i* is drastically decreased by the presence of linkers, which connect sites *i* and *j*, and thus site *j* plays a role of anchor. To represent this effect, we assume that the rate constant of the transfer from *Di* to *Bi* becomes *Ak*_*on*_, when some sites are in the binding state. Here, *A* is the acceleration rate and is usually larger than the unity. Although the three-dimensional conformations affect the acceleration rate largely, it is very difficult to include this effect in the acceleration rate, which is beyond the purpose of this paper. Therefore, we consider *A* constant by ignoring the dependence of *A* on the detailed conformation. The dissociation of the RNA from DNA is defined as the state where all the binding states are in the dissociation state. When *A* = 1, the system loses the linker effect.

To demonstrate the cooperative effect of multiple binding sites of RNA, we conducted MC simulations of the above theoretical model, where *k*_*on*_ = 0.002 and *k*_*off*_ = 0.2/step. Here, the important property is the dissociation rate of RNA, which is defined as *P*_*diss*_ = (Number of samples in the dissociation state)/(Number of samples in total). To investigate the dependency of *P*_*diss*_ on the number of sites (*N*) and on the acceleration rate (*A*), MC simulations were carried out for *N* = 1, 2, 5, 10, 15, 20 and *A* = 1, 2, 5, 10, 15, 20. Note that both the values can take much larger values. For each system, ten MC simulations were used and the number of steps for each simulation is 20,000. For all the systems, the standard errors of *P*_*diss*_ are very small (< 10^− 3^). For the case of *N* = 1 and *A* = 1, the MC simulation reproduced the analytic value (*P*_*diss*_ = *k*_*off*_ /(*k*_*on*_ + *k*_*off*_) = 0.99).

## Additional files

Additional file 1: Lists of redundant UC (TC)/AG-motifs in Xist/XIST/Rsx RNAs. The distributions of these listed motifs are depicted in Fig. 2a. AG-12a, b, and c motifs in opossum Rsx are indicated at the right of the motifs as 12a (pink), 12b (green), and 12c (blue), respectively. The 12-nucleotide motifs, aggaggaaggga (seven copies, shown in red), are not clustered in Rsx RNA. T is used instead of U as the RNA sequences are derived from genomic sequences. (PDF 503 kb)

Additional file 2: A list of human lncRNAs studied for r-UC/r-AG motifs. (PDF 242 kb)

Additional file 3: Representative secondary structures of Rs*x* RNA predicted by Mfold. The numbers denoted at some loops indicate the number of nucleotides in each motif that are located in the single-stranded loops of Rsx RNA. Note that the motifs in the Rsx AG-12 domain, positions 4,141–4,630 and 9,821–10,620 are only redundant-AG (r-AG) motifs because of the lack of redundant-UC (r-UC) motifs in these regions (see Fig. 3a). The RNA secondary structures predicted for each of these two regions in the Rsx AG-12 domain are similar, and a representative Structure 1 is presented. On the other hand, those predicted for the Rsx UC-dominant domain are varied, and three examples are presented here (Structures 1–3). (PDF 282 kb)

Additional file 4: Proportions of L1s, L2s, and other families of indicated length ranges on X chromosomes. This table shows the details of Fig. 4b, indicating that the maximum length of L2s is shorter than that of L1s in the three species. (PDF 340 kb)

Additional file 5: A list of 50 randomly selected L1s of the three species. Start and end positions on X chromosomes, subfamilies, and length were based on RepeatMasker annotations. (+) and (−) represent plus and minus strand DNAs, respectively. L1s longer than 7,000 bp are fewer than 50 in number, namely 46 for human, 39 for mouse, and 4 for opossum, all of which were used in Fig. 5a. (PDF 719 kb)

Additional file 6: Proportions of r-TC/r-AG motifs occupied in randomly selected L3s and RTEs in opossum X chromosome. Thirty each of L3s (LINE-3s or CR-1s) and RTEs (retrotransposable elements) of the same length ranges as in Fig. 5a (every 100 bp up to 1,000 bp, and every 1,000 bp above 1,000 bp) were randomly selected. Total ranges of the L3 and RTE lengths are 26–3,307 bp and 30–4,097 bp, respectively. The total number and the average proportion of the r-TC/r-AG motifs are indicated at the top and right of each graph, respectively. The tables show the details of the r-TC/r-AG motif proportions in L1s, L2s, L3s, and RTEs examined. (PDF 1242 kb)

Additional file 7: Mapping and length–distributions of r-TC/r-AG motifs in other representative L1 subfamilies of the three species. (PDF 1719 kb)

Additional file 8: Lists of r-TC/r-AG motifs in paired L1s of the representative subfamilies of the three species. The following tables list the r-TC/r-AG motifs in the human paired L1s depicted in Fig. 7b, and also in the paired L1MD_T (mouse) and L1_Mdo4 (opossum) that show moderate-to-high sequence identities (91 and 89%, respectively). (PDF 699 kb)

Additional file 9: Whole alignments of r-AG motif sequences in the paired L1s of the three species. Only the first and last parts of the alignments of human paired L1PA5 and L1PA8 are depicted in Fig. 7c. The paired L1Md_T (mouse) and L1_Mdo4 (mouse) with moderate-to-high sequence identities (91 and 89%, respectively) are also shown. In contrast to colored motifs, non-colored motifs reflect variations in nucleotides. A rectangular line surrounding an entire or part of an alignment indicates an area that was analyzed for sequence identities by BLAST. For example, positions 740–6,118 for (A) and 717–6,117 for (B) of L1Md_T indicate areas analyzed by a BLAST search. (PDF 712 kb)

Additional file 10: Detailed data of r-AG motifs in the paired representative L1 subfamilies from the three species. The following table summarizes the data presented in Fig. 8. The order of human subfamilies is based on its phylogenetic tree, while the order of mouse and opossum subfamilies is based on values of L1 sequence identities. r-AG motif identities of the paired L1s with no significant similarity were not determined. NS, no significant sequence similarity; ND, not determined; r-AG MLD, r-AG motif–length distribution. (PDF 323 kb)

Additional file 11: Cumulative ratios of r-UC (TC)/r-AG motifs including overlaps in the paired L1s and XIST/Xist/Rsx RNAs. The cumulative ratios for 5–7-nucleotide motifs are shown in red. *No. represents motif number including overlaps. These three paired L1s were used in Additional file 9. (PDF 4301 kb)

## Data Availability

Please contact author for data requests.

## Abbreviations

CD: Circular dichroism
ecRNA: Euchromatin-associated chromosomal RNA
EZH2: Enhancer of zeste homolog 2
HuR: Human antigen R
KCNQ1ot1: KCNQ1 opposite strand/antisense transcript 1
*K*_*d*_: Dissociation constant
KS distance: Kolmogorov–Smirnov distance
LINE-1, 2, 3 or L1, L2, L3: Long interspersed nuclear element 1, 2, 3
lncRNA: Long noncoding RNA
MC simulation: Monte Carlo simulation
MEG3: Maternally expressed gene 3
PRC2: Polycomb repressive complex 2
pRNA: Promoter-associated RNA
QCM: Quartz crystal microbalance
RNA FISH: RNA fluorescence in situ hybridization
Rsx: RNA-on-the-silent X
RTE: Retrotransposable element
r-UC (TC)/r-AG motifs: Redundant UC (TC)/redundant-AG motifs
SINE: Short interspersed nuclear element
TFB: Triplex forming buffer
XCI: X-chromosome inactivation
Xi: Inactive X chromosome
Xist/XIST: X-inactive specific transcript
