# Supplementary material for: Convergence in LINE-1 nucleotide variations can benefit redundantly forming triplexes with lncRNA in mammalian X-chromosome inactivation
Source: Mob DNA. 2019 Jul 30;10:33. doi: 10.1186/s13100-019-0173-4 (PMC6664574; doi:10.1186/s13100-019-0173-4)
Supplement: Supplementary file 1 — Lists of redundant UC (TC)/AG-motifs in Xist/XIST/Rsx RNAs. The distributions of these listed motifs are depicted in Fig. 2a. AG-12a, b, and c motifs in opossum Rsx are indicated at the right of the motifs as 12a (pink), 12b (green), and 12c (blue), respectively. The 12-nucleotide motifs, aggaggaaggga (seven copies, shown in red), are not clustered in Rsx RNA. T is used instead of U as the RNA sequences are derived from genomic sequences. (PDF 503 kb) [file 13100_2019_173_MOESM1_ESM.pdf]

Additional file 1: Lists of redundant UC (TC)/AG-motifs in Xist/XIST/Rsx RNAs

The distributions of these listed motifs are depicted in Figure 2a. AG-12a, b, and c motifs in opossum Rsx are indicated at the right of the motifs as 12a (pink), 12b (green), and 12c (blue), respectively. The 12-nucleotide motifs, aggaggaaggga (seven copies, shown in red), are not clustered in Rsx RNA. T is used instead of U as the RNA sequences are derived from genomic sequences.

Gene Name	Transcript ID	No.	r-UC Sequence	Length	Start Position in Transcript	End Position in Transcript	No.	r-AG Sequence	Length	Start Position in Transcript	End Position in Transcript
**Xist**	**NR_001463**	1	ttcttcc	7	45	51	1	aaggag	6	69	74
		2	tttctctcc	9	56	64	2	aagaaa	6	126	131
		3	tttcttt	7	258	264	3	aaggaa	6	250	255
		4	cctttctttc	10	308	317	4	agaaa	5	843	847
		5	ttctt	5	330	334	5	aagaa	5	950	954
		6	ttcttt	6	417	422	6	agaaag	6	1095	1100
		7	tttctt	6	426	431	7	aaggag	6	1103	1108
		8	tttct	5	442	446	8	agagga	6	1145	1150
		9	ttcttt	6	483	488	9	agggga	6	1153	1158
		10	tttcttctcctt	12	490	501	10	aggaaa	6	1347	1352
		11	cctttctc	8	560	567	11	agaaa	5	1666	1670
		12	tcttt	5	600	604	12	agaaa	5	1715	1719
		13	tttcttt	7	653	659	13	aagga	5	1778	1782
		14	ttcttt	6	660	665	14	gagaga	6	1909	1914
		15	ctttcttt	8	1019	1026	15	aaagaaagaaa	11	1966	1976
		16	tcttt	5	1299	1303	16	aagga	5	1995	1999
		17	ttctt	5	1368	1372	17	agagaaa	7	3070	3076
		18	tttctttc	8	1501	1508	18	agaga	5	4334	4338
		19	tcttt	5	1812	1816	19	aaagaa	6	4739	4744
		20	ttctt	5	1928	1932	20	agaga	5	4798	4802
		21	tctctt	6	2028	2033	21	aaggaaagaaa	11	4843	4853
		22	ttccttt	7	2081	2087	22	agaaa	5	4953	4957
		23	ttctct	6	2154	2159	23	aggaa	5	5264	5268
		24	ccttcttcc	9	2238	2246	24	aaagaggg	8	5312	5319
		25	tcctt	5	2441	2445	25	gagagaa	7	5557	5563
		26	ctttctttc	9	2504	2512	26	aaagaaa	7	5681	5687
		27	tcctct	6	2714	2719	27	aagaaga	7	5720	5726
		28	ctttct	6	2765	2770	28	aaagagg	7	6053	6059
		29	ttctt	5	2776	2780	29	agaaggaaa	9	6361	6369
		30	ttctcctc	8	2799	2806	30	aaaga	5	6585	6589
		31	tttct	5	4482	4486	31	aaagga	6	6604	6609
		32	tccttc	6	4751	4756	32	aggga	5	6658	6662
		33	tctctc	6	4821	4826	33	aaggga	6	6769	6774
		34	tctcctttcctttt	13	4830	4842	34	gaaagga	7	6979	6985
		35	tttct	5	4891	4895	35	aggaag	6	7014	7019
		36	tcttt	5	4912	4916	36	aagaa	5	7075	7079
		37	tctttct	7	5145	5151	37	agaaagaggg	10	7109	7118
		38	ctttctt	7	5205	5211	38	aagga	5	7396	7400
		39	tcttt	5	5274	5278	39	aaggaagag	9	7874	7882
		40	cttctct	7	5452	5458	40	agaga	5	8045	8049
		41	tctttct	7	5466	5472	41	aggaa	5	8237	8241
		42	cctcttcct	9	5661	5669	42	agggag	6	8301	8306
		43	ttcct	5	5840	5844	43	gagaaggaag	10	8368	8377
		44	tctttc	6	5866	5871	44	aggaaggag	9	8407	8415
		45	tcctt	5	5954	5958	45	aagaaa	6	8430	8435
		46	cttctttc	8	6075	6082	46	agaaa	5	8482	8486
		47	tccttc	6	6123	6128	47	aagaaagg	8	8510	8517
		48	tctct	5	6144	6148	48	ggaaaga	7	8640	8646
		49	tcctcttt	8	6160	6167	49	agaga	5	8983	8987
		50	ccttcctc	8	6206	6213	50	aaagagaga	9	9065	9073
		51	tttct	5	6347	6351	51	aagaaa	6	9084	9089
		52	tcttt	5	6503	6507	52	gagaaa	6	9408	9413
		53	cttcttc	7	6511	6517	53	gaggagga	8	9429	9436
		54	tcttcct	7	6519	6525	54	aagaagaagga	11	9554	9564
		55	cctcctttcc	10	6547	6556	55	agaggag	7	9577	9583
		56	ctcttt	6	6558	6563	56	ggagagag	8	9595	9602
		57	tctctt	6	6753	6758	57	aaaggga	7	9606	9612
		58	tcttccttt	9	6826	6834	58	aagga	5	9636	9640
		59	tcttt	5	6836	6840	59	ggagaaa	7	9730	9736
		60	ctccttc	7	6861	6867	60	aaaga	5	9738	9742
		61	ttcttt	6	6915	6920	61	aggggaa	7	9837	9843
		62	cttcctt	7	6995	7001	62	aaggaaga	8	9909	9916
		63	cttctct	7	7068	7074	63	aaagaa	6	9926	9931
		64	cctcttctcc	10	7089	7098	64	aggagaaa	8	9951	9958
		65	tcctt	5	7201	7205	65	gaggaag	7	9987	9993
		66	ctcttt	6	7209	7214	66	aggaaag	7	10029	10035
		67	tttct	5	7238	7242	67	agaaa	5	10138	10142
		68	cttctctt	8	7248	7255	68	aaaggaagg	9	10181	10189
		69	ctctcttt	8	7409	7416	69	gagaggag	8	11616	11623
		70	ctctcttcctt	11	7584	7594	70	gagaaa	6	11692	11697
		71	tttct	5	7647	7651	71	agaaga	6	11766	11771
		72	ttccttt	7	7802	7808	72	aaagga	6	11797	11802
		73	tttct	5	7840	7844	73	aaagaagaggaa	12	11857	11868
		74	cctttctc	8	7890	7897	74	aagga	5	11960	11964
		75	cctcttt	7	7922	7928	75	aagaga	6	11991	11996
		76	ccttctctt	9	7956	7964	76	ggaagga	7	12021	12027
		77	ccttctcctt	10	8022	8031	77	aagaagggaaa	11	12055	12065
		78	ttctt	5	8059	8063	78	aaaggaa	7	12175	12181
		79	tcttt	5	8180	8184	79	aggaa	5	12348	12352
		80	ttctt	5	8245	8249	80	aagaaa	6	12413	12418
		81	ctttccttt	9	8332	8340	81	aggaaag	7	12419	12425
		82	tcttcct	7	8361	8367	82	agggggaaa	9	12438	12446
		83	tttctttcc	9	8387	8395	83	agagaaa	7	12464	12470
		84	ttcttcctcc	10	8397	8406	84	aaagag	6	12475	12480
		85	ttcctt	6	8504	8509	85	gagaagaa	8	12496	12503
		86	tttct	5	8591	8595	86	aaaggaa	7	12676	12682
		87	tcttt	5	8682	8686	87	gagaga	6	12719	12724
		88	tcttt	5	8693	8697	88	aagga	5	12729	12733
		89	tcttt	5	8728	8732	89	aagagggaag	10	12779	12788
		90	tcctt	5	8815	8819	90	aagaaa	6	12962	12967
		91	ttcct	5	8960	8964	91	agggaa	6	13019	13024
		92	ttccttt	7	9045	9051	92	aggaaa	6	13133	13138
		93	tttcct	6	9075	9080	93	aaaga	5	13241	13245
		94	ctttctct	8	9094	9101	94	aggaa	5	13253	13257
		95	tcttt	5	9139	9143	95	agaagagaaa	10	13385	13394
		96	tttct	5	9424	9428	96	aaaggggga	9	13508	13516
		97	cttctcttc	9	9681	9689	97	aaagaa	6	13542	13547
		98	ctttcct	7	9893	9899	98	aagaa	5	13676	13680
		99	ttctctctcc	10	10012	10021	99	aaggaa	6	13704	13709
		100	tttctt	6	10276	10281	100	aagaa	5	13766	13770
		101	tttcctt	7	10299	10305	101	agaaa	5	13855	13859
		102	ttcttt	6	10321	10326	102	agaaa	5	13997	14001
		103	tttcttcctt	10	10337	10346	103	ggagaga	7	14093	14099
		104	tttcttcctt	10	10361	10370	104	aaagag	6	14376	14381
		105	tttcttcctt	10	10385	10394	105	aaagaaa	7	14405	14411
		106	ttcttt	6	10408	10413	106	gaggaaa	7	14768	14774
		107	tttcttcctt	10	10424	10433	107	agaaa	5	14782	14786
		108	tttcttcctt	10	10446	10455	108	aggaa	5	14902	14906
		109	ttcttt	6	10470	10475	109	agaga	5	14951	14955
		110	tttcttcctt	10	10486	10495	110	aaaggag	7	14979	14985
		111	tttcttcctt	10	10510	10519	111	agggaa	6	15028	15033
		112	ttcttt	6	10533	10538	112	ggaggga	7	15112	15118
		113	tttcttcctt	10	10549	10558	113	aggaga	6	15225	15230
		114	tcttcctt	8	10573	10580	114	gaagaa	6	15325	15330
		115	tttcttcctt	10	10593	10602	115	aaagaa	6	15362	15367
		116	ttcttt	6	10617	10622	116	agaagaggg	9	15417	15425
		117	tttcttcctt	10	10633	10642	117	aaggaag	7	15626	15632
		118	tctttctttctt	12	10654	10665	118	aaaggagaa	9	15738	15746
		119	tttcttcctt	10	10681	10690	119	aagaagaa	8	15808	15815
		120	ttcttt	6	10705	10710	120	aaagaa	6	15892	15897
		121	tttcttcctt	10	10721	10730	121	aaaga	5	15899	15903
		122	tttcttcctt	10	10746	10755	122	aaagga	6	15909	15914
		123	ttcttt	6	10769	10774	123	gagggga	7	15950	15956
		124	tttcttc	7	10785	10791	124	agaggagg	8	16015	16022
		125	tctcctt	7	10811	10817	125	aaagagg	7	16083	16089
		126	tttcttcctt	10	10845	10854	126	agaaga	6	16119	16124
		127	tttcttcctt	10	10869	10878	127	agaggga	7	16199	16205
		128	tcttcctt	8	10895	10902	128	agaaa	5	16221	16225
		129	tcttcctt	8	10919	10926	129	gaagaa	6	16251	16256
		130	cttcttcctt	10	10941	10950	130	aggaagg	7	16299	16305
		131	ttcttt	6	10961	10966	131	aagga	5	16347	16351
		132	tttcttccttccttt	15	10977	10991	132	aaaggaaa	8	16396	16403
		133	tctccttctttccttt	16	10997	11012	133	agggaag	7	16413	16419
		134	tcttt	5	11018	11022	134	aagaaa	6	16594	16599
		135	tttctcct	8	11055	11062	135	aggaggga	8	16647	16654
		136	ttctttctt	9	11068	11076	136	agaaa	5	16813	16817
		137	ctttctct	8	11100	11107	137	aagaa	5	16917	16921
		138	ctctct	6	11146	11151	138	agaaa	5	16942	16946
		139	tttctctt	8	11173	11180	139	ggaggaa	7	16950	16956
		140	tttctttc	8	11185	11192	140	gaaaga	6	16958	16963
		141	cctctcttt	9	11240	11248	141	gaaagaa	7	16971	16977
		142	ttctt	5	11249	11253	142	aaaga	5	16984	16988
		143	ttcttt	6	11270	11275	143	gaaggaaaggg	11	17059	17069
		144	ttctcttt	8	11283	11290	144	aaggaag	7	17271	17277
		145	tttctttct	9	11307	11315	145	aaagaaa	7	17353	17359
		146	tcctt	5	11326	11330	146	aggaa	5	17360	17364
		147	ttctt	5	11337	11341	147	agagaaga	8	17366	17373
		148	tttcttcc	8	11349	11356	148	aggaa	5	17517	17521
		149	cctttctt	8	11360	11367	149	aaaggaaa	8	17546	17553
		150	ctttctct	8	11374	11381	150	gaaaga	6	17669	17674
		151	tttcttt	7	11384	11390					
		152	tctcttt	7	11401	11407					
		153	tttctt	6	11420	11425					
		154	tttct	5	11463	11467					
		155	tcttct	6	11469	11474					
		156	ctttcctcctttct	14	11476	11489					
		157	tctcttt	7	11509	11515					
		158	tctctctt	8	11527	11534					
		159	tcctttctt	9	11549	11557					
		160	tttcttt	7	11563	11569					
		161	ttcctc	6	11671	11676					

162	tttct	5	11872	11876
163	tttct	5	12030	12034
164	tccttct	7	12036	12042
165	tttctt	6	12109	12114
166	tttct	5	12118	12122
167	tttctcttc	9	12271	12279
168	ccttctttcttt	12	12559	12570
169	tttct	5	12595	12599
170	tcttt	5	12751	12755
171	ccttcctcttcctct	15	12762	12776
172	tttcct	6	12870	12875
173	ttcctt	6	12932	12937
174	tccttt	6	12947	12952
175	tttccttcc	9	13042	13050
176	tttcttct	8	13337	13344
177	tttct	5	13474	13478
178	ctttctt	7	13803	13809
179	tttct	5	13848	13852
180	tctct	5	13886	13890
181	ttctctttc	9	14075	14083
182	tttctt	6	14196	14201
183	ctttct	6	14600	14605
184	ctttct	6	14704	14709
185	tttctt	6	14921	14926
186	tctctcc	7	15526	15532
187	ttccttc	7	15594	15600
188	cttcct	6	15616	15621
189	tttctc	6	15646	15651
190	tccttt	6	15690	15695
191	tttccttt	8	15710	15717
192	tttct	5	15878	15882
193	tttct	5	15945	15949
194	ttctct	6	16090	16095
195	tttcctc	7	16097	16103
196	ctcttt	6	16208	16213
197	tttct	5	16241	16245
198	ctttcctc	8	16259	16266
199	tctctt	6	16360	16365
200	tctcttt	7	16440	16446
201	tcctt	5	16469	16473
202	tcttctcc	8	16514	16521
203	tttct	5	16606	16610
204	ttctcctt	8	16820	16827
205	ttctt	5	16901	16905
206	tttctttc	8	17021	17028
207	tttct	5	17144	17148
208	tctcttt	7	17226	17232
209	tttct	5	17532	17536
210	ccttcctttc	10	17567	17576

Gene Name	Transcript ID	No.	r-UC Sequence	Length	Start Position in Transcript	End Position in Transcript	No.	r-AG Sequence	Length	Start Position in Transcript	End Position in Transcript
**XIST**	**NR_001564**	1	ttctt	5	8	12	1	aaagaaag	8	240	247
		2	tttctt	6	43	48	2	aaggaa	6	282	287
		3	ctctctc	7	50	56	3	agagaa	6	873	878
		4	cttcct	6	68	73	4	ggaggaaa	8	916	923
		5	tctctct	7	83	89	5	agggagg	7	991	997
		6	ttctttct	8	100	107	6	agaggaag	8	1023	1030
		7	tttct	5	115	119	7	aagaa	5	1120	1124
		8	tttcttt	7	146	152	8	aaggga	6	1142	1147
		9	ctttctcct	9	198	206	9	aggaaaga	8	1168	1175
		10	tcttctt	7	211	217	10	aggaaa	6	1366	1371
		11	tttcttt	7	290	296	11	aggga	5	1459	1463
		12	tctct	5	317	321	12	aaaga	5	1539	1543
		13	ctctcttt	8	353	360	13	gggagaaa	8	1577	1584
		14	tttcttt	7	407	413	14	agaaa	5	1629	1633
		15	ttcttt	6	446	451	15	agaaga	6	1666	1671
		16	tttcctt	7	496	502	16	aggagaaagaag	12	1760	1771
		17	tttcttt	7	599	605	17	aagga	5	1833	1837
		18	tttcctt	7	652	658	18	aagga	5	2152	2156
		19	tttct	5	697	701	19	agaagaggaa	10	2196	2205
		20	ctctct	6	801	806	20	aagaggaa	8	2220	2227
		21	tcttt	5	1128	1132	21	agaaa	5	2350	2354
		22	cctcttt	7	1415	1421	22	agaaa	5	2580	2584
		23	tcttt	5	1423	1427	23	aaagag	6	2619	2624
		24	tcttt	5	1747	1751	24	aggaaa	6	2710	2715
		25	tcttt	5	1826	1830	25	aaagaa	6	3125	3130
		26	ttcctct	7	1941	1947	26	agggaa	6	3156	3161
		27	ttcct	5	2206	2210	27	aaaggagaaa	10	3240	3249
		28	ccttcctc	8	2212	2219	28	aaagaa	6	3556	3561
		29	tcttt	5	2275	2279	29	agagaaga	8	3828	3835
		30	ttcctt	6	2418	2423	30	agaaaggga	9	4166	4174
		31	ttcctt	6	2428	2433	31	aagga	5	4217	4221
		32	ttcctt	6	2459	2464	32	agggga	6	4303	4308
		33	tttct	5	2502	2506	33	agaaa	5	4450	4454
		34	tttcttt	7	2532	2538	34	aagga	5	4647	4651
		35	tttccttt	8	2556	2563	35	aaaga	5	4707	4711
		36	tcctt	5	2667	2671	36	aaggga	6	4852	4857
		37	tcctct	6	2738	2743	37	agaaaggga	9	4960	4968
		38	ttcctcc	7	2807	2813	38	aagga	5	5608	5612
		39	ttcctc	6	2931	2936	39	agagag	6	5766	5771
		40	cttctctc	8	2949	2956	40	gaagga	6	5968	5973
		41	ctcctt	6	3069	3074	41	agagaaggaaag	12	6055	6066
		42	tttcctt	7	3131	3137	42	aggaag	6	6099	6104
		43	ctttct	6	3175	3180	43	agaaa	5	6331	6335
		44	tttctc	6	3220	3225	44	agggag	6	6382	6387
		45	tttctct	7	3253	3259	45	agggag	6	6673	6678
		46	tcttt	5	3301	3305	46	aaggaag	7	6909	6915
		47	ctcttt	6	3334	3339	47	agggag	6	6965	6970
		48	tcttt	5	3437	3441	48	agggag	6	7266	7271
		49	ttctt	5	3543	3547	49	aggaag	6	7555	7560
		50	tttctt	6	3662	3667	50	agggag	6	7846	7851
		51	tcttt	5	3732	3736	51	aagaa	5	7864	7868
		52	ttctt	5	3762	3766	52	aaggaag	7	8084	8090
		53	tcctcttt	8	3949	3956	53	agggag	6	8140	8145
		54	tttccttctctt	12	3987	3998	54	aaggag	6	8394	8399
		55	ctcttctt	8	4222	4229	55	aagga	5	8636	8640
		56	tcttt	5	4376	4380	56	aagga	5	8975	8979
		57	tcttt	5	4402	4406	57	aggggga	7	9103	9109
		58	ttccttt	7	4517	4523	58	aggagaaa	8	9287	9294
		59	tcttt	5	4578	4582	59	aagaa	5	10301	10305
		60	tttcttt	7	4654	4660	60	agggag	6	10367	10372
		61	cctcttcct	9	4691	4699	61	aggaaggaagg	11	10429	10439
		62	tctttc	6	4811	4816	62	aagaa	5	10486	10490
		63	tcctcctt	8	4834	4841	63	agaaa	5	10550	10554
		64	tctctcc	7	4920	4926	64	aagaaaga	8	10586	10593
		65	tccttctctt	10	4985	4994	65	aaaga	5	10748	10752
		66	tttct	5	5012	5016	66	agaaa	5	10815	10819
		67	tcttt	5	5110	5114	67	aagaaga	7	10882	10888
		68	tttcct	6	5189	5194	68	gagggaa	7	10909	10915
		69	tctttctt	8	5199	5206	69	aagaaa	6	11069	11074
		70	tttct	5	5210	5214	70	aaagaa	6	11257	11262
		71	cctcctt	7	5386	5392	71	ggagaagga	9	11274	11282
		72	cttcttctctt	11	5425	5435	72	aaaga	5	11288	11292
		73	cttcctt	7	5499	5505	73	agggag	6	11305	11310
		74	ctctctt	7	5551	5557	74	aaagaagag	9	11343	11351
		75	ccttctcttc	10	5790	5799	75	ggagaaa	7	11564	11570
		76	tccttc	6	5836	5841	76	agaagaa	7	11583	11589
		77	tttct	5	5855	5859	77	aggggaaa	8	11668	11675
		78	cctcttt	7	5872	5878	78	aggaaga	7	11744	11750
		79	cctttctc	8	5913	5920	79	agagag	6	11833	11838
		80	tccttccttt	10	5957	5966	80	aagaaa	6	11858	11863
		81	tttctt	6	6003	6008	81	gaaggaag	8	11876	11883
		82	tttctt	6	6282	6287	82	aagaaa	6	12952	12957
		83	tcctctcc	8	6343	6350	83	aagaa	5	13059	13063
		84	tttcct	6	6566	6571	84	aagga	5	13133	13137
		85	ttcct	5	6605	6609	85	agagga	6	13174	13179
		86	tcttcctttcc	11	6654	6664	86	aggaagga	8	13243	13250
		87	tttctt	6	6854	6859	87	gagagaga	8	13527	13534
		88	ttcct	5	6893	6897	88	gagaaga	7	13650	13656
		89	tcttcctttcc	11	6946	6956	89	agagaaa	7	13701	13707
		90	tttctt	6	7159	7164	90	gaagaga	7	13713	13719
		91	tttct	5	7198	7202	91	gagaagaa	8	13734	13741
		92	tcttcctttcc	11	7247	7257	92	aagaaa	6	13748	13753
		93	tttcct	6	7448	7453	93	aaggggga	7	13896	13902
		94	ttcct	5	7487	7491	94	aaaga	5	13993	13997
		95	tcttcctttcc	11	7536	7546	95	gagagaa	7	13999	14005
		96	cctcttcct	9	7735	7743	96	aagagaaa	8	14114	14121
		97	tcttcctttcc	11	7827	7837	97	agagaaa	7	14386	14392
		98	tttcct	6	8029	8034	98	aaagaaaggag	11	14552	14562
		99	tttct	5	8068	8072	99	aaagga	6	14587	14592
		100	cctcctct	8	8091	8098	100	aaggaa	6	14612	14617
		101	tctttcttt	9	8121	8129	101	agaaa	5	14713	14717
		102	cctcttt	7	8318	8324	102	aaggga	6	14767	14772
		103	ttctt	5	8341	8345	103	aaagggggaaa	11	14832	14842
		104	ttctt	5	8419	8423	104	agaaagg	7	14869	14875
		105	tttctt	6	8580	8585	105	aagaggaaag	10	15030	15039
		106	ttctt	5	8620	8624	106	aaggaa	6	15065	15070
		107	cctcttt	7	8648	8654	107	aggaaagag	9	15155	15163
		108	ctcctt	6	8672	8677	108	gaggaaa	7	15171	15177
		109	tcttt	5	8682	8686	109	aggaaa	6	15622	15627
		110	cctcttt	7	8753	8759	110	aaggga	6	15667	15672
		111	tttcttt	7	8830	8836	111	gaagaa	6	15683	15688
		112	tcttt	5	8839	8843	112	aaaga	5	15746	15750
		113	cctttccttt	10	8872	8881	113	agaagaaa	8	15780	15787
		114	ctttctt	7	8989	8995	114	agaga	5	15875	15879
		115	ttctct	6	9064	9069	115	agaga	5	15927	15931
		116	ttcct	5	9077	9081	116	aggaa	5	15972	15976
		117	ccttcttt	8	9128	9135	117	agaaggaaa	9	16074	16082
		118	cctcttcttt	10	9167	9176	118	agagaga	7	16191	16197

119	tttcttt	7	9243	9249	119	agaaga	6	16211	16216
120	ttcctcc	7	9306	9312	120	gaggaga	7	16238	16244
121	cttcctc	7	9428	9434	121	aaaggag	7	16349	16355
122	tctccttct	9	9508	9516	122	aagagaga	8	16445	16452
123	cttctcct	8	9584	9591	123	agaagaa	7	16484	16490
124	tttctcc	7	9629	9635	124	agaaga	6	16576	16581
125	cctctctct	9	9824	9832	125	aaagaaagaaa	11	16619	16629
126	cctcttt	7	9958	9964	126	aaagaga	7	16651	16657
127	cctttctctt	10	9991	10000	127	aggaa	5	16674	16678
128	ctttcctt	8	10025	10032	128	aggaga	6	16717	16722
129	cttcctttc	9	10063	10071	129	agaga	5	16844	16848
130	tttctttc	8	10248	10255	130	aggaaga	7	16850	16856
131	ttctt	5	10309	10313	131	aaaga	5	16874	16878
132	tccttt	6	10384	10389	132	gaaaga	6	16946	16951
133	ctttctct	8	10446	10453	133	aggaa	5	16999	17003
134	ttcttctttcc	11	10457	10467	134	aagaga	6	17014	17019
135	tctctt	6	10491	10496	135	gagaagaaa	9	17036	17044
136	tctctc	6	10683	10688	136	aaggaaa	7	17077	17083
137	tcttt	5	10783	10787	137	aagagaggg	9	17126	17134
138	tccttt	6	10839	10844	138	agaaa	5	17320	17324
139	tttcttc	7	10847	10853	139	aaaga	5	17344	17348
140	ttcttt	6	11094	11099	140	aggagaaagg	10	17384	17393
141	ttcttt	6	11239	11244	141	agaga	5	17480	17484
142	tttctc	6	11268	11273	142	agagaa	6	17511	17516
143	tctct	5	11352	11356	143	aggaa	5	17678	17682
144	ctcttt	6	11359	11364	144	aggaggga	8	17755	17762
145	tcctt	5	11403	11407	145	agaaa	5	17932	17936
146	tctcctt	7	11418	11424	146	agaaa	5	18013	18017
147	ctcctctt	8	11428	11435	147	aagaaggg	8	18047	18054
148	tctttcc	7	11501	11507	148	aagaa	5	18119	18123
149	tcttcctc	8	11575	11582	149	gaaaggaaa	9	18157	18165
150	ctttcct	7	11727	11733	150	aggaggaagag	11	18248	18258
151	ttcct	5	11793	11797	151	gaaggaaa	8	18260	18267
152	cttcct	6	11816	11821	152	aagaa	5	18317	18321
153	tttcttt	7	11953	11959	153	aagaa	5	18482	18486
154	tctctttctttctt	14	11961	11974	154	aaagaagagga	11	18563	18573
155	tcttt	5	11976	11980	155	ggagaaggaaa	11	18652	18662
156	ctcttt	6	11982	11987	156	ggagggag	8	18689	18696
157	ttctct	6	11989	11994	157	ggaaagaagaaag	13	18865	18877
158	tttctctt	8	12024	12031	158	aagaa	5	18933	18937
159	tttctttc	8	12037	12044	159	aagagaa	7	18939	18945
160	tttctcttt	9	12064	12072	160	aaggaa	6	19016	19021
161	tctctc	6	12111	12116	161	aaaga	5	19053	19057
162	tttctcttt	9	12136	12144	162	agaaag	6	19186	19191
163	ctctcct	7	12146	12152					
164	ctcttctttc	10	12161	12170					
165	tctccttt	8	12186	12193					
166	tccttt	6	12220	12225					
167	cctctcctttctct	14	12240	12253					
168	cctctcttt	9	12259	12267					
169	tctcttt	7	12268	12274					
170	tttct	5	12333	12337					
171	cctcctttct	10	12346	12355					
172	tttct	5	12360	12364					
173	tttctc	6	12380	12385					
174	tttct	5	12387	12391					
175	tttcctttct	10	12395	12404					
176	cctctctt	8	12406	12413					
177	ttctctctctcctcc	15	12420	12434					
178	tctctt	6	12451	12456					
179	tttct	5	12469	12473					
180	tctct	5	12487	12491					
181	ttctct	6	12507	12512					
182	cttcttcc	8	12514	12521					
183	ctttct	6	12522	12527					
184	cctctcttt	9	12569	12577					
185	tctctcctt	9	12590	12598					
186	tttcttt	7	12604	12610					
187	cttcct	6	12660	12665					
188	ttcctt	6	12722	12727					
189	cctcttt	7	13301	13307					
190	cttctt	6	13321	13326					
191	ttctcct	7	13345	13351					
192	tcttt	5	13357	13361					
193	ttcttt	6	13370	13375					
194	tttct	5	13503	13507					
195	tcttctcc	8	13515	13522					
196	tcctttcttt	10	13619	13628					
197	ctctct	6	13695	13700					
198	cttccttcttt	11	13798	13808					
199	tttct	5	13842	13846					
200	tctct	5	13982	13986					
201	ctttcctt	8	14032	14039					
202	tttcttcct	9	14046	14054					
203	tttct	5	14211	14215					
204	tttct	5	14260	14264					
205	ttctt	5	14311	14315					
206	tttct	5	14394	14398					
207	tttccttt	8	14411	14418					
208	ctctttc	7	14579	14585					
209	ttctcct	7	15016	15022					
210	tcctct	6	15044	15049					
211	ctttct	6	15235	15240					
212	ctttct	6	15276	15281					
213	ctttct	6	15294	15299					
214	tctct	5	15315	15319					
215	tttctc	6	15553	15558					
216	tcttt	5	15647	15651					
217	tcttt	5	15758	15762					
218	tcctt	5	15870	15874					
219	ctttct	6	16006	16011					
220	cttcct	6	16223	16228					
221	tttctt	6	16290	16295					
222	tcttt	5	16407	16411					
223	tcttt	5	16493	16497					
224	ttctct	6	16531	16536					
225	tttctttc	8	16638	16645					
226	tttcct	6	16662	16667					
227	cttcctc	7	16691	16697					
228	ttcttt	6	16746	16751					
229	ttcttt	6	16879	16884					
230	ttctt	5	16933	16937					
231	ttctct	6	17008	17013					
232	tttcttt	7	17025	17031					
233	ctttccttt	9	17526	17534					
234	tttctt	6	17543	17548					
235	ttcct	5	17593	17597					
236	tctcttct	8	17606	17613					
237	ctcctt	6	17665	17670					
238	ctctcctt	8	17683	17690					
239	tttcct	6	17707	17712					
240	ttcttt	6	17877	17882					
241	tctctttc	8	17924	17931					
242	tttctc	6	17980	17985					
243	cttctct	7	17995	18001					
244	ttctccttt	9	18020	18028					
245	tttctct	7	18179	18185					
246	tccttt	6	18398	18403					
247	ccttctt	7	18515	18521					
248	tttct	5	18621	18625					
249	tttct	5	18726	18730					
250	cctttcttc	9	18824	18832					
251	ccttcctttc	10	18888	18897					
252	tttct	5	18902	18906					
253	tccttct	7	19041	19047					
254	tctcttt	7	19125	19131					
255	tcttt	5	19136	19140					

**Gene Name**	**Transcript ID**	**No.**	**r-UC Sequence**	**Length**	**Start Position in Transcript**	**End Position in Transcript**	No.	r-AG Sequence	Length	**Start Position in Transcript**	**End Position in Transcript**
**Rsx**	**JQ937282.1**	1	tttct	5	51	55	1	gaagaaa	7	91	97
		2	ttcct	5	63	67	2	agaaga	6	141	146
		3	tctct	5	147	151	3	agaaa	5	453	457
		4	ctcttt	6	581	586	4	aggga	5	463	467
		5	tctctt	6	669	674	5	agagagaaa	9	491	499
		6	tccttctt	8	744	751	6	gagagggaaa	10	566	575
		7	cttcctt	7	755	761	7	gagagaaaga	10	587	596
		8	ccttcctt	8	1330	1337	8	agaggaa	7	610	616
		9	cttcct	6	1446	1451	9	aagaag	6	646	651
		10	tcttcct	7	1478	1484	10	ggagagaggag	11	655	665
		11	ttctttc	7	1554	1560	11	aggaaagg	8	729	736
		12	tccttct	7	1588	1594	12	aaagga	6	866	871
		13	cctcctcct	9	1788	1796	13	aagaga	6	921	926
		14	tcctttc	7	1852	1858	14	aaaggga	7	947	953
		15	cctctcct	8	2420	2427	15	aaggga	6	1003	1008
		16	ttcttcc	7	8447	8453	16	aaagggaa	8	1034	1041
		17	tccttct	7	10606	10612	17	aaagga	6	1091	1096
		18	ccttcctct	9	10672	10680	18	aaggga	6	1119	1124
		19	ttcctt	6	10728	10733	19	aggaga	6	1140	1145
		20	tttct	5	10772	10776	20	aaagga	6	1304	1309
		21	ctcttctt	8	10988	10995	21	aggaa	5	1344	1348
		22	tttct	5	11012	11016	22	aaggaa	6	1377	1382
		23	tcttt	5	11057	11061	23	aagaga	6	1412	1417
		24	tttct	5	11119	11123	24	aaggaa	6	1536	1541
		25	cttcctctt	9	11453	11461	25	aagga	5	1571	1575
		26	ccttcctttc	10	11472	11481	26	aaagaga	7	1706	1712
		27	ctcttt	6	11530	11535	27	aggagaa	7	1756	1762
		28	tcttt	5	11695	11699	28	aagga	5	1777	1781
		29	tctct	5	11776	11780	29	aaggaa	6	1833	1838
		30	ttctt	5	11947	11951	30	aggga	5	1864	1868
		31	tttctcttt	9	11964	11972	31	aggga	5	1888	1892

32	tcctct	6	12024	12029	32	agggga	6	1896	1901
33	tctttc	6	12050	12055	33	aagga	5	1984	1988
34	ttcctt	6	12361	12366	34	aagga	5	2052	2056
35	ctttct	6	12400	12405	35	aaggag	6	2094	2099
36	tcttt	5	12417	12421	36	aaggaaa	7	2128	2134
37	tcttt	5	12626	12630	37	aagga	5	2184	2188
38	tttcttc	7	12640	12646	38	aagga	5	2210	2214
39	tttcctc	7	12649	12655	39	gaagga	6	2216	2221
40	tcttt	5	12670	12674	40	aggaga	6	2237	2242
41	tcctt	5	12735	12739	41	gggaagaaagga	12	2244	2255
42	ctcttt	6	12840	12845	42	gaagga	6	2290	2295
43	cctctct	7	12865	12871	43	aagga	5	2326	2330
44	ttcctt	6	12962	12967	44	aggaa	5	2359	2363
45	tttctctt	8	12989	12996	45	aaggaa	6	2401	2406
46	ttcttc	6	13021	13026	46	aagga	5	2409	2413
47	ctcctt	6	13050	13055	47	gaagga	6	2445	2450
48	ttctcct	7	13082	13088	48	aggaagaaaggag	13	2473	2485
49	ttcct	5	13091	13095	49	aagaa	5	2488	2492
50	ttctt	5	13113	13117	50	aaagaa	6	2527	2532
51	ttctt	5	13134	13138	51	aagga	5	2565	2569
52	ttcctct	7	13169	13175	52	agaaa	5	2585	2589
53	ttccttt	7	13199	13205	53	aaaggaa	7	2591	2597
54	ttccttt	7	13313	13319	54	aagaag	6	2600	2605
55	cctcctt	7	13356	13362	55	aaggaa	6	2616	2621
56	tctct	5	13385	13389	56	**aggaagaaggga**	**12a**	2651	2662
57	ttcctctt	8	13405	13412	57	**aggaagaaggga**	**12a**	2686	2697
58	ttcttt	6	13439	13444	58	**aggaagaaggga**	**12a**	2720	2731
59	tttcct	6	13497	13502	59	**aggaagaaggga**	**12a**	2754	2765
60	ttccttt	7	13524	13530	60	**aggaagaaggga**	**12a**	2788	2799
61	cctcctt	7	13560	13566	61	aaaga	5	2802	2806
62	ttcct	5	13568	13572	62	aggaggaag	9	2822	2830
63	ttctcct	7	13581	13587	63	aggaaa	6	2862	2867
64	ttcctcc	7	13614	13620	64	aggaagaag	9	2896	2904
65	tttcttt	7	13643	13649	65	aggaaa	6	2931	2936
66	tcctt	5	13664	13668	66	gaggaaa	7	2964	2970
67	ttcct	5	13672	13676	67	gaagaaggaa	10	3001	3010
68	ttcct	5	13700	13704	68	gaagaaggaa	10	3035	3044
69	tcctt	5	13707	13711	69	aggaaa	6	3067	3072
70	tttctctt	8	13727	13734	70	aaggga	6	3073	3078
71	ttccttt	7	13760	13766	71	agaaga	6	3081	3086
72	ttccttt	7	13791	13797	72	gggaagaaggga	12	3109	3120
73	ttcctt	6	13826	13831	73	**aggaggaaggga**	**12**	3151	3162
74	ttcct	5	13833	13837	74	**aggaggaaggga**	**12**	3193	3204
75	ttcctctcct	10	13844	13853	75	agaaga	6	3207	3212
76	ttcctctt	8	13876	13883	76	ggagagaggaagag	14	3229	3242
77	ttccttt	7	13991	13997	77	aggaggaaagga	12	3268	3279
78	tccttct	7	14012	14018	78	aagga	5	3283	3287
79	ttcct	5	14020	14024	79	**aggaagaaggga**	**12a**	3310	3321
80	ctccttt	7	14042	14048	80	**aggaagaaggga**	**12a**	3344	3355
81	ttcct	5	14052	14056	81	**aggaagaaggga**	**12a**	3378	3389
82	ttcctcct	8	14074	14081	82	**aggaagaaggga**	**12a**	3412	3423
83	ttcctctt	8	14106	14113	83	**aggaagaaggga**	**12a**	3446	3457
84	ttccttt	7	14138	14144	84	**aggaagaaggga**	**12a**	3480	3491
85	ttccttt	7	14168	14174	85	**aggaagaaggga**	**12a**	3514	3525
86	ctcctt	6	14205	14210	86	**aggaagaaggga**	**12a**	3548	3559
87	ttcct	5	14212	14216	87	**aggaagaaggga**	**12a**	3582	3593
88	ttctcct	7	14225	14231	88	aaagga	6	3622	3627
89	ttcctcc	7	14256	14262	89	aaagga	6	3645	3650
90	ttccttt	7	14285	14291	90	aaagga	6	3668	3673
91	ttcct	5	14314	14318	91	aaagga	6	3691	3696
92	ttcct	5	14342	14346	92	aaagga	6	3714	3719
93	ttcctcct	8	14362	14369	93	aaagga	6	3737	3742
94	tcctt	5	14372	14376	94	aaagga	6	3760	3765
95	ttctcttc	8	14392	14399	95	aggaag	6	3796	3801
96	ttccttt	7	14424	14430	96	aagaag	6	3827	3832
97	ttcctctt	8	14454	14461	97	aagaag	6	3858	3863
98	tcttt	5	14487	14491	98	aagaag	6	3889	3894
99	ttcct	5	14503	14507	99	aagaag	6	3920	3925
100	ttccttt	7	14545	14551	100	aagaag	6	3951	3956
101	ttcct	5	14582	14586	101	**aggaggaaggga**	**12**	3982	3993
102	ttcctctcct	10	14593	14602	102	aggaagaag	9	4024	4032
103	ttcctctt	8	14629	14636	103	aggaagaag	9	4054	4062
104	cttctt	6	14679	14684	104	aggaagaag	9	4084	4092
105	ttccttt	7	14743	14749	105	aggaagaag	9	4114	4122
106	tccttct	7	14764	14770	106	aggaagagg	9	4149	4157
107	ttcct	5	14772	14776	107	**aggaagagggga**	**12b**	4184	4195
108	ctccttt	7	14794	14800	108	**aggaagagggga**	**12b**	4219	4230
109	ttcct	5	14804	14808	109	**aggaagagggga**	**12b**	4254	4265
110	tcctt	5	14837	14841	110	**aggaagagggga**	**12b**	4289	4300
111	tttctctt	8	14857	14864	111	**aggaagagggga**	**12b**	4324	4335
112	ttccttt	7	14890	14896	112	**aggaagagggga**	**12b**	4359	4370
113	ttccttt	7	14920	14926	113	**aggaagagggga**	**12b**	4394	4405
114	ttcctcc	7	14959	14965	114	**aggaagagggga**	**12b**	4429	4440
115	ttcct	5	14969	14973	115	**aggaagagggga**	**12b**	4464	4475
116	ttcct	5	14990	14994	116	**aggaagagggga**	**12b**	4499	4510
117	ttccttt	7	15007	15013	117	**aggaagagggga**	**12b**	4534	4545
118	ttcctt	6	15037	15042	118	**aggaagagggga**	**12b**	4569	4580
119	ttcct	5	15044	15048	119	**aggaagagggga**	**12b**	4604	4615
120	ttcctctcct	10	15055	15064	120	aggaagaaagga	12	4639	4650
121	ttcctctt	8	15089	15096	121	aggaaa	6	4662	4667
122	cttctt	6	15138	15143	122	agaaga	6	4676	4681
123	ttccttt	7	15201	15207	123	aggaagaag	9	4704	4712
124	tccttct	7	15223	15229	124	aggagaaaggga	12	4738	4749
125	ttcct	5	15231	15235	125	aaaga	5	4753	4757
126	ctccttt	7	15253	15259	126	aggaagaaagga	12	4780	4791
127	ttcct	5	15263	15267	127	aaaga	5	4795	4799
128	ttcctctt	8	15285	15292	128	aggaagaag	9	4822	4830
129	ttccttt	7	15318	15324	129	agaaa	5	4856	4860
130	ttccttt	7	15348	15354	130	gggagaaaggga	12	4887	4898
131	ttctt	5	15378	15382	131	aaaga	5	4902	4906
132	ttcttc	6	15403	15408	132	ggaagaag	8	4930	4937
133	tcctctt	7	15433	15439	133	aggaagaaag	10	4957	4966
134	ttccttt	7	15460	15466	134	aaaga	5	4972	4976
135	tttct	5	15470	15474	135	**aggaagaaggga**	**12a**	4999	5010
136	ttcttt	6	15491	15496	136	**aggaagaaggga**	**12a**	5033	5044
137	tttct	5	15500	15504	137	**aggaagaaggga**	**12a**	5067	5078
138	ttccttt	7	15519	15525	138	aggaagaagggg	12	5101	5112
139	ttcctctt	8	15602	15609	139	**aggaagaaggga**	**12a**	5135	5146
140	ttcttctt	8	15640	15647	140	agaaga	6	5159	5164
141	ctcctctt	8	15801	15808	141	agaaagaaagga	12	5169	5180
142	ttcctctt	8	15820	15827	142	aggaaa	6	5192	5197
143	ctccttt	7	15860	15866	143	agaaga	6	5206	5211
144	ttcttct	7	15891	15897	144	aggaagaag	9	5234	5242
145	tttcct	6	15900	15905	145	aggagaaag	9	5268	5276
146	ttcttctt	8	15921	15928	146	aaaga	5	5283	5287
147	tttccttt	8	15953	15960	147	aggaagaaagga	12	5310	5321
148	ttccttct	8	16003	16010	148	aaaga	5	5325	5329
149	tttctcc	7	16027	16033	149	aaaga	5	5332	5336
150	tttctctt	8	16035	16042	150	ggaagaag	8	5353	5360
151	ttcctctt	8	16062	16069	151	aggaagaaag	10	5380	5389
152	tttcttt	7	16090	16096	152	aaaga	5	5395	5399
153	tttctctt	8	16119	16126	153	**aggaagaaggga**	**12a**	5422	5433
154	tttctctt	8	16148	16155	154	**aggaagaaggga**	**12a**	5456	5467
155	tctct	5	16184	16188	155	**aggaagaaggga**	**12a**	5490	5501
156	tcctct	6	16194	16199	156	**aggaagaaggga**	**12a**	5524	5535
157	ttcct	5	16229	16233	157	**aggaagaaggga**	**12a**	5558	5569
158	ttcctctt	8	16333	16340	158	aaagga	6	5598	5603
159	ttcctct	7	16366	16372	159	aaagga	6	5621	5626
160	tttctctt	8	16397	16404	160	aaagga	6	5644	5649
161	ttcttctt	8	16429	16436	161	aaagga	6	5667	5672
162	cttcctctt	9	16439	16447	162	aaagga	6	5690	5695
163	ttcttt	6	16475	16480	163	aaagga	6	5713	5718
164	tttctt	6	16505	16510	164	aaagga	6	5736	5741
165	tttct	5	16559	16563	165	aggaag	6	5772	5777
166	ctcttt	6	16569	16574	166	aagaag	6	5803	5808
167	tttctcc	7	16617	16623	167	aagaag	6	5834	5839
168	ttcctctt	8	16659	16666	168	aagaag	6	5865	5870
169	tctctt	6	16712	16717	169	aagaag	6	5896	5901
170	tcctt	5	16720	16724	170	aagaag	6	5927	5932
171	ttcctctt	8	16746	16753	171	**aggaggaaggga**	**12**	5958	5969
172	tttcctctt	9	16774	16782	172	aggaagaag	9	6000	6008
173	tttctctt	8	16860	16867	173	aggaagaag	9	6030	6038
174	tttct	5	16891	16895	174	aggaagaag	9	6060	6068
175	ctttctt	7	16933	16939	175	aggaagaag	9	6090	6098
176	ctttct	6	16941	16946	176	aggaagagg	9	6125	6133
177	tttctctt	8	17013	17020	177	**aggaagagggga**	**12b**	6160	6171
178	tttctttc	8	17126	17133	178	**aggaagagggga**	**12b**	6195	6206
179	tcttt	5	17218	17222	179	**aggaagagggga**	**12b**	6230	6241
180	tttct	5	17439	17443	180	**aggaagagggga**	**12b**	6265	6276
181	tttctctt	8	17548	17555	181	**aggaagagggga**	**12b**	6300	6311
182	tttct	5	17576	17580	182	**aggaagagggga**	**12b**	6335	6346
183	tttcct	6	17612	17617	183	**aggaagagggga**	**12b**	6370	6381
184	ttcctct	7	17684	17690	184	**aggaagagggga**	**12b**	6405	6416
185	tttct	5	17712	17716	185	**aggaagagggga**	**12b**	6440	6451
186	tcttt	5	17777	17781	186	**aggaagagggga**	**12b**	6475	6486
187	tttctctt	8	17849	17856	187	**aggaagagggga**	**12b**	6510	6521
188	tttct	5	17877	17881	188	**aggaagagggga**	**12b**	6545	6556
189	cttcttt	7	17900	17906	189	**aggaagagggga**	**12b**	6580	6591
190	tctcct	6	17938	17943	190	**aggaagagggga**	**12b**	6615	6626
191	ttctct	6	18082	18087	191	**aggaagagggga**	**12b**	6650	6661
192	ctttct	6	18111	18116	192	**aggaagagggga**	**12b**	6685	6696
193	tcttt	5	18126	18130	193	**aggaagagggga**	**12b**	6720	6731
194	cttctt	6	18205	18210	194	aggaagaaagga	12	6755	6766
195	tttcctct	8	18293	18300	195	aggaaa	6	6778	6783
196	tttcctcc	8	18322	18329	196	agaaga	6	6792	6797
197	ctttct	6	18338	18343	197	aggaagaag	9	6820	6828
198	tttct	5	18354	18358	198	aggagaaaggga	12	6854	6865
199	tttct	5	18367	18371	199	aaaga	5	6869	6873

200	cctctcttc	9	18380	18388
201	tttct	5	18400	18404
202	tttcct	6	18425	18430
203	ttctt	5	18459	18463
204	tttct	5	18472	18476
205	tccttc	6	18523	18528
206	tttct	5	18530	18534
207	tttct	5	18589	18593
208	tttcctt	7	18607	18613
209	ttcttt	6	18680	18685
210	tttctc	6	18735	18740
211	tttcct	6	18762	18767
212	tttct	5	18774	18778
213	tttccttt	8	18822	18829
214	cttctt	6	18836	18841
215	ctttctt	7	18860	18866
216	tttccttct	9	18882	18890
217	ttcttt	6	19006	19011
218	tttctct	7	19036	19042
219	tcctcctt	8	19132	19139
220	tttcttt	7	19157	19163
221	tttct	5	19243	19247
222	ctttcct	7	19260	19266
223	ttccttcc	8	19291	19298
224	tttct	5	19306	19310
225	tttct	5	19475	19479
226	tttct	5	19495	19499
227	tctcttt	7	19581	19587
228	tcttctt	7	19692	19698
229	ttctcct	7	19761	19767
230	tttcct	6	19850	19855
231	tctccttt	8	19976	19983
232	tcctct	6	20078	20083
233	ttcttc	6	20323	20328
234	tttctt	6	20468	20473
235	tttcttcc	8	20619	20626
236	ccttcctt	8	20863	20870
237	tttct	5	20910	20914
238	tcttt	5	20936	20940
239	tttct	5	20973	20977
240	ttctt	5	21031	21035
241	tcttt	5	21083	21087
242	tcttt	5	21136	21140
243	tcttt	5	21289	21293
244	tctcttt	7	21297	21303
245	ttctt	5	21430	21434
246	ttctctt	7	21541	21547
247	tttctcct	8	21713	21720
248	ctccttt	7	21746	21752
249	tcctt	5	21817	21821
250	tttcttt	7	21843	21849
251	ttcttt	6	21850	21855
252	ttcttt	6	21909	21914
253	tttcttt	7	22056	22062
254	ttctt	5	22106	22110
255	tcctt	5	22112	22116
256	tttcttt	7	22131	22137
257	tcttcctt	8	22159	22166
258	ttctcctt	8	22184	22191
259	tttct	5	22281	22285
260	tttcct	6	22297	22302
261	tcctt	5	22323	22327
262	ctccttt	7	22349	22355
263	tcttt	5	22375	22379
264	tcctt	5	22416	22420
265	ttccttt	7	22445	22451
266	tcttt	5	22463	22467
267	tcctt	5	22499	22503
268	tcttt	5	22576	22580
269	ttcttt	6	22633	22638
270	ttctcctt	8	22672	22679
271	tttcttt	7	22729	22735
272	tttccttcc	9	22747	22755
273	tcttt	5	22809	22813
274	tttcct	6	23055	23060
275	tcttt	5	23075	23079
276	tttctc	6	23206	23211
277	ttctct	6	23263	23268
278	tcctctt	7	23317	23323
279	tttct	5	23465	23469
280	tcctt	5	23504	23508
281	tccttt	6	23516	23521
282	tctctct	7	23522	23528
283	tcttt	5	23577	23581
284	tctct	5	23593	23597
285	tcttctc	7	23753	23759
286	tttct	5	23768	23772
287	tccttt	6	23930	23935

200	aggaagaaagga	12	6896	6907
201	aaaga	5	6911	6915
202	aggaagaag	9	6938	6946
203	agaaa	5	6972	6976
204	gggagaaaggga	12	7003	7014
205	aaaga	5	7018	7022
206	ggaagaag	8	7046	7053
207	aggaagaaag	10	7073	7082
208	aaaga	5	7088	7092
209	**aggaagaaggga**	**12a**	7115	7126
210	**aggaagaaggga**	**12a**	7149	7160
211	**aggaagaaggga**	**12a**	7183	7194
212	aggaagaagggg	12	7217	7228
213	**aggaagaaggga**	**12a**	7251	7262
214	agaaga	6	7275	7280
215	agaaagaaagga	12	7285	7296
216	aggaaa	6	7308	7313
217	agaaga	6	7322	7327
218	aggaagaag	9	7350	7358
219	aggagaaag	9	7384	7392
220	aaaga	5	7399	7403
221	gggaagaaagga	12	7426	7437
222	aaaga	5	7441	7445
223	aaaga	5	7448	7452
224	ggaagaag	8	7470	7477
225	aggaagaaag	10	7497	7506
226	aaaga	5	7512	7516
227	**aggaagaaggga**	**12a**	7538	7549
228	**aggaagaaggga**	**12a**	7572	7583
229	**aggaagaaggga**	**12a**	7606	7617
230	**aggaagaaggga**	**12a**	7640	7651
231	aggaagaa	8	7674	7681
232	**aggaagaaggga**	**12a**	7708	7719
233	**aggaagaaggga**	**12a**	7742	7753
234	**aggaagaaggga**	**12a**	7776	7787
235	aggaagaagaga	12	7810	7821
236	**aggaagaaggga**	**12a**	7844	7855
237	aaggag	6	7859	7864
238	aggaaa	6	7867	7872
239	agaaga	6	7881	7886
240	aggaa	5	7909	7913
241	aggaag	6	7943	7948
242	aaaggga	7	7979	7985
243	aaaga	5	7989	7993
244	ggaagaag	8	8017	8024
245	agaaag	6	8048	8053
246	aaaga	5	8059	8063
247	**aggaagaaggga**	**12a**	8086	8097
248	aggaaagaaggga	13	8120	8132
249	aggaaagaaggga	13	8155	8167
250	aggaaagaaggga	13	8190	8202
251	aaggag	6	8206	8211
252	aggaaa	6	8214	8219
253	aggaagaag	9	8256	8264
254	aaagga	6	8296	8301
255	gaggaga	7	8305	8311
256	**aggaggaaggga**	**12**	8332	8343
257	aaaga	5	8347	8351
258	aggaagaag	9	8374	8382
259	aagaggaaagga	12	8399	8410
260	aagaggaaagga	12	8422	8433
261	aagga	5	8437	8441
262	**aggaagaaggga**	**12a**	8464	8475
263	aggaaagga	9	8501	8509
264	aggaaa	6	8527	8532
265	gaggaagaag	10	8568	8577
266	agaagaag	8	8584	8591
267	aaagaa	6	8614	8619
268	aaagga	6	8637	8642
269	aggaaa	6	8654	8659
270	agagga	6	8668	8673
271	aggaagaag	9	8696	8704
272	aaagga	6	8736	8741
273	gaggaga	7	8745	8751
274	aggaagaag	9	8772	8780
275	**aggaggaaggga**	**12**	8806	8817
276	aaaga	5	8821	8825
277	**aggaggaaggga**	**12**	8848	8859
278	aaaga	5	8863	8867
279	**aggaagaaggga**	**12a**	8890	8901
280	**aggaagaaggga**	**12a**	8920	8931
281	**aggaagaaggga**	**12a**	8950	8961
282	**aggaagaaggga**	**12a**	8980	8991
283	**aggaagaaggga**	**12a**	9010	9021
284	**aggaagaaggga**	**12a**	9040	9051
285	**aggaagaaggga**	**12a**	9070	9081
286	**aggaagaaggga**	**12a**	9100	9111
287	**aggaagaaggga**	**12a**	9130	9141
288	**aggaagaaggga**	**12a**	9160	9171
289	**aggaagaaggga**	**12a**	9190	9201
290	aggaa	5	9220	9224
291	aaggga	6	9226	9231
292	agaaa	5	9251	9255
293	aaaggga	7	9256	9262
294	agaaa	5	9282	9286
295	aaaggga	7	9287	9293
296	agaaa	5	9313	9317
297	aaaggga	7	9318	9324
298	aaaggga	7	9351	9357
299	aaagggaa	8	9385	9392
300	aaaggga	7	9421	9427
301	gaaggga	7	9533	9539
302	**aggaagaaggga**	**12a**	9562	9573
303	**aggaagaaggga**	**12a**	9597	9608
304	**aggaagaaggga**	**12a**	9631	9642
305	**aggaagaaggga**	**12a**	9665	9676
306	**aggaagaaggaa**	**12c**	9699	9710
307	**aggaagaaggga**	**12a**	9733	9744
308	**aggaagaaggga**	**12a**	9763	9774
309	**aggaagaaggga**	**12a**	9793	9804
310	**aggaagaaggga**	**12a**	9823	9834
311	**aggaagaaggga**	**12a**	9853	9864
312	aggaagaagggg	12	9887	9898
313	**aggaagaaggga**	**12a**	9921	9932
314	**aggaagaaggga**	**12a**	9955	9966
315	**aggaagaaggga**	**12a**	9989	10000
316	**aggaagaaggga**	**12a**	10023	10034
317	aggaag	6	10053	10058
318	aggga	5	10060	10064
319	**aggaagaaggga**	**12a**	10087	10098
320	aggaagaagaga	12	10121	10132
321	**aggaagaggga**	**12b**	10151	10162
322	**aggaagaaggga**	**12a**	10181	10192
323	aggaagaagggg	12	10211	10222
324	aggaaga	7	10241	10247
325	**aggaagaaggga**	**12a**	10271	10282
326	**aggaagaaggga**	**12a**	10301	10312
327	**aggaagaaggga**	**12a**	10331	10342
328	**aggaagaaggga**	**12a**	10361	10372
329	**aggaagaaggaa**	**12c**	10395	10406
330	**aggaagaaggaa**	**12c**	10426	10437
331	**aggaagaaggaa**	**12c**	10457	10468
332	**aggaagaaggaa**	**12c**	10488	10499
333	**aggaagaaggaa**	**12c**	10519	10530
334	aggaagagg	9	10550	10558
335	agaga	5	10586	10590
336	**aggaagaaggaa**	**12c**	10592	10603
337	aggaa	5	10659	10663
338	aagaga	6	10856	10861
339	aggaaa	6	10882	10887
340	aggaaa	6	10914	10919
341	agaaa	5	11021	11025
342	gagaaa	6	11039	11044
343	gaagaa	6	11095	11100
344	aagagaaa	8	11181	11188
345	aaggagg	7	11207	11213
346	aggaaa	6	11246	11251
347	aggaga	6	11267	11272
348	aggaaa	6	11314	11319
349	gagggaag	8	11331	11338
350	aggaagaggaga	12	11412	11423
351	aggaaa	6	11519	11524
352	aagaga	6	11542	11547
353	aggaa	5	11565	11569
354	aggaaa	6	11571	11576
355	aaaga	5	11629	11633
356	aagaggaagaaa	12	11658	11669
357	gaaggaga	8	11681	11688
358	agaaa	5	11690	11694
359	gagaaa	6	11733	11738
360	aaaga	5	11849	11853
361	agaaa	5	11900	11904
362	aggaga	6	11923	11928
363	agggaa	6	11990	11995
364	agaga	5	12008	12012
365	gagaaa	6	12037	12042
366	aggaaa	6	12056	12061
367	agaaagaag	9	12092	12100

368	gagagggaa	9	12120	12128
369	gaggggagaaagagagg	17	12130	12146
370	agaaggga	8	12152	12159
371	agaga	5	12204	12208
372	gaaaga	6	12214	12219
373	aagagaaggg	10	12248	12257
374	gagggga	7	12323	12329
375	agaga	5	12458	12462
376	agagaggag	9	12507	12515
377	aagaaa	6	12678	12683
378	aaggag	6	12701	12706
379	aaaggaag	8	12812	12819
380	aagga	5	12924	12928
381	agggggaaa	9	12975	12983
382	ggagaaa	7	13004	13010
383	gggaggaagaaga	13	13037	13049
384	ggaagaggggaaa	13	13064	13076
385	aaaga	5	13077	13081
386	ggaaggaaa	9	13096	13104
387	aaaga	5	13105	13109
388	agaggaagaaga	12	13122	13133
389	agaaa	5	13158	13162
390	aggaaggaaa	10	13183	13192
391	aaaga	5	13266	13270
392	aaaga	5	13279	13283
393	aaagaga	7	13347	13353
394	aagaa	5	13373	13377
395	ggaaggaa	8	13390	13397
396	aaagga	6	13399	13404
397	aaggaaa	7	13453	13459
398	agagaa	6	13510	13515
399	aaaga	5	13553	13557
400	ggaaaggggaaa	12	13595	13606
401	agaaaga	7	13607	13613
402	aaaga	5	13688	13692
403	ggaaggaa	8	13712	13719
404	aaagga	6	13721	13726
405	ggagaaa	7	13742	13748
406	aggaag	6	13752	13757
407	aaaga	5	13821	13825
408	ggaaggaaa	9	13861	13869
409	aaagga	6	13870	13875
410	aagaggaaa	9	13911	13919
411	aaaga	5	13920	13924
412	gagggaaa	8	13941	13948
413	gagaagaaaga	11	14031	14041
414	aaaga	5	14069	14073
415	ggaagggaaa	10	14089	14098
416	aaagga	6	14100	14105
417	gagggaaa	8	14188	14195
418	aaaga	5	14197	14201
419	ggaaagggaaa	11	14239	14249
420	aaaga	5	14251	14255
421	aaaga	5	14330	14334
422	aaaga	5	14357	14361
423	ggaaggaa	8	14377	14384
424	aaagga	6	14386	14391
425	ggagaaa	7	14406	14412
426	agaggggaaa	10	14469	14478
427	aaaga	5	14570	14574
428	ggaagaaa	8	14610	14617
429	aaagga	6	14623	14628
430	aagaggaaa	9	14664	14672
431	gaggaaa	7	14693	14699
432	gagaagaaaga	11	14783	14793
433	ggaaggaa	8	14842	14849
434	aaagga	6	14851	14856
435	ggagaaa	7	14872	14878
436	agaaag	6	14882	14887
437	agagggaggaaa	12	14935	14946
438	aaaga	5	15032	15036
439	ggaaggaaa	9	15072	15080
440	aaagga	6	15083	15088
441	aaaggaaa	8	15125	15132
442	gaggaaa	7	15152	15158
443	gagaagaaaga	11	15242	15252
444	aaaga	5	15280	15284
445	agagaa	6	15333	15338
446	aaaga	5	15373	15377
447	gaggaaa	7	15391	15397
448	agaaa	5	15420	15424
449	ggaggaaa	8	15447	15454
450	aagga	5	15455	15459
451	agaggagggaagaaga	16	15475	15490
452	aaagaa	6	15541	15546
453	aaagaaa	7	15586	15592
454	aaaga	5	15597	15601
455	aaaggga	7	15618	15624
456	agaaga	6	15675	15680
457	aggaga	6	15697	15702
458	aagaa	5	15706	15710
459	aaggaga	7	15724	15730
460	agagggaga	9	15772	15780
461	aaaga	5	15796	15800
462	aagga	5	15815	15819
463	gagaaa	6	15839	15844
464	agggaaa	7	15845	15851
465	aaaga	5	15855	15859
466	agaggaaa	8	15875	15882
467	aagga	5	15886	15890
468	aggaagaaa	9	15908	15916
469	ggaaggaaa	9	15968	15976
470	agggggaaa	9	15988	15996
471	aaagga	6	15997	16002
472	ggagaaa	7	16018	16024
473	agagaaa	7	16050	16056
474	aaagaaagga	10	16080	16089
475	ggaaagaaagaaa	13	16098	16110
476	ggaggaaa	8	16134	16141
477	aaagga	6	16142	16147
478	ggagaga	7	16163	16169
479	agaaa	5	16210	16214
480	aagga	5	16242	16246
481	aggaggaaa	9	16320	16328
482	aaagaaa	7	16356	16362
483	ggaaggaaa	9	16381	16389
484	aaagga	6	16391	16396
485	ggaggaaggaaa	12	16412	16423
486	agaaa	5	16424	16428
487	ggaaggaaa	9	16458	16466
488	aaggggaaa	10	16492	16501
489	aaaggaagaga	11	16624	16634
490	aaaga	5	16674	16678
491	aagaga	6	16809	16814
492	gaagaaga	8	16871	16878
493	agaaa	5	16982	16986
494	aggaaa	6	16999	17004
495	gaagaa	6	17037	17042
496	ggagaga	7	17067	17073
497	agaaa	5	17095	17099
498	aggaaa	6	17112	17117
499	agaaa	5	17253	17257
500	agagaaa	7	17309	17315
501	aaggaaa	7	17432	17438
502	agaaa	5	17524	17528
503	aaagga	6	17532	17537
504	agagaag	7	17625	17631
505	agggggaaga	11	17783	17793
506	aaggaa	6	17795	17800
507	agaaa	5	17815	17819
508	aggaa	5	17910	17914
509	agagaag	7	17951	17957
510	aggaaa	6	17976	17981
511	agagaag	7	18064	18070
512	aggaaa	6	18088	18093
513	aaggaa	6	18226	18231
514	agagaagaaa	10	18268	18277
515	gaagaa	6	18405	18410
516	agaga	5	19220	19224
517	gagagaa	7	19341	19347
518	aagaga	6	19451	19456
519	aggggaa	7	19510	19516
520	gagaga	6	19678	19683
521	gagaga	6	19747	19752
522	aggaaa	6	19913	19918
523	aggaga	6	20064	20069
524	aggaga	6	20184	20189
525	aggaga	6	20309	20314
526	ggaagaaa	8	20335	20342
527	aagaa	5	20794	20798
528	gaggaa	6	20811	20816
529	aggaga	6	21307	21312
530	aaagga	6	21777	21782
531	agaaa	5	21902	21906
532	aggaga	6	22145	22150
533	agaaga	6	22399	22404
534	agaaga	6	22936	22941
535	gaaaggaa	8	23385	23392
536	aagaggaa	8	23425	23432
537	aagaggggagaaa	13	23447	23459
538	aaggag	6	23726	23731

539	gaggaga	7	23831	23837
540	agaga	5	23852	23856
541	aagaagagaaa	11	23884	23894
542	aaagaaa	7	24092	24098
