# Supplementary material for: Convergence in LINE-1 nucleotide variations can benefit redundantly forming triplexes with lncRNA in mammalian X-chromosome inactivation
Source: Mob DNA. 2019 Jul 30;10:33. doi: 10.1186/s13100-019-0173-4 (PMC6664574; doi:10.1186/s13100-019-0173-4)
Supplement: Supplementary file 2 — A list of human lncRNAs studied for r-UC/r-AG motifs. (PDF 242 kb) [file 13100_2019_173_MOESM2_ESM.pdf]

Additional file 2: A list of human lncRNAs studied for r-UC/r-AG motifs

No.	ID	Name	Length(nt)	r-UC(No./kb)	r-AG(No./kb)	r-UC/r-AG(No./No.)	r-UC(%)	r-AG(%)	r-UC/r-AG(%/%)
1	NR_002728.3	KCNQ1OT1	91,671	5.9	17.2	0.34	3.83	11.88	0.32
2	NR_046473	MEG3 v16	9,701	7.2	17.9	0.40	4.94	14.46	0.34
3	NR_026647	PWRN2	5,128	6.0	18.9	0.32	4.27	12.68	0.34
4	NR_033805	WAC-AS1	5,376	6.1	15.4	0.40	4.19	10.92	0.38
5	NR_026947	C1RL-AS1	5,402	7.8	13.9	0.56	5.04	11.74	0.43
6	NR_126453	IRAIN	5,359	4.9	9.5	0.51	3.53	7.86	0.45
7	NR_033897	EPHA1-AS1	5,034	6.6	14.1	0.46	4.71	10.25	0.46
8	NR_026790	HCG11	5,688	6.5	10.2	0.64	3.83	8.30	0.46
9	JQ937287	Rsx	24,118	11.9	22.5	0.53	7.66	15.89	0.48
10	NR_026800	FAM30A	9,643	6.2	11.3	0.55	4.37	8.93	0.49
11	NR_027451	NORAD	5,378	7.1	9.9	0.71	4.33	8.37	0.52
12	NR_027037	MBNL1-AS1 v2	6,658	7.5	14.3	0.53	5.33	10.09	0.53
13	NR_024052	HCG18 v1	6,820	6.6	12.0	0.55	4.41	8.30	0.53
14	NR_002819	MALAT1 v1	8,779	8.8	14.4	0.61	5.56	10.31	0.54
15	NR_026876	RNF217-AS1	5,360	8.6	15.3	0.56	5.52	9.89	0.56
16	NR_002791	EMX2OS v1	7,282	7.8	12.2	0.64	5.30	9.17	0.58
17	NR_040093	STARD4-AS1	5,525	7.8	11.9	0.65	5.19	8.67	0.60
18	NR_102401	DNAH17-AS1	5,163	5.8	9.3	0.62	4.14	6.82	0.61
19	NR_003255	TSIX	37,027	10.6	12.2	0.87	6.28	10.20	0.62
20	NR_002727	RFPL1S	5,125	7.8	11.1	0.70	5.17	8.04	0.64
21	NR_046084	SH3BP5-AS1	5,592	8.0	11.6	0.69	5.33	7.99	0.67
22	NR_038397	DNM3OS v1	7,957	10.7	13.8	0.77	6.74	9.73	0.69
23	NR_045697	PRICKLE2-AS1	6,521	8.9	11.8	0.75	5.84	8.19	0.71
24	NR_146164	FAM157A	6,879	6.1	8.4	0.73	4.38	6.09	0.72
25	NR_027136	TMEM51-AS1	6,979	7.7	10.7	0.72	5.62	7.67	0.73
26	NR_026677	MIR600HG	6,002	9.3	11.8	0.79	6.41	8.51	0.75
27	NR_003491	MIAT v1	10,194	10.8	12.7	0.85	7.32	9.55	0.77
28	NR_026794	FAM238B	6,118	8.2	10.5	0.78	5.85	7.06	0.83
29	NR_038343	MAGI2-AS3 v1	8,498	11.9	13.1	0.91	7.70	9.24	0.83
30	NR_108047	RNF139-AS1 v1	5,658	9.9	10.8	0.92	6.34	7.37	0.86
31	NR_026891	GABPB1-IT1	5,944	8.7	9.6	0.91	6.04	6.48	0.93
32	NR_040051	IQCH-AS1	5,966	9.4	10.6	0.89	6.79	7.26	0.94
33	NR_026892	AFAP1-AS1	6,810	9.0	9.7	0.92	6.37	6.75	0.94
34	NR_001545	TTTY15	5,262	9.9	9.7	1.02	6.42	6.48	0.99
35	NR_109792	MIR124-2HG	6,557	11.4	11.0	1.04	7.82	7.52	1.04
36	NR_002813	KCNIP4-IT1	9,848	11.3	11.4	0.99	7.62	7.25	1.05
37	NR_024344	MIR4697HG	5,306	10.9	10.0	1.09	8.29	7.78	1.07
38	NR_121580	SMC2-AS1	7,020	11.7	11.5	1.02	7.76	7.24	1.07
39	NR_024366	FAM225A	5,802	10.5	9.8	1.07	7.86	7.29	1.08
40	NR_024376	FAM225B	5,836	10.8	9.9	1.09	7.98	7.37	1.08
41	NR_002323	TUG1 v3	7,542	10.3	9.8	1.06	6.85	6.31	1.09
42	NR_046262	NIPBL-AS1	5,346	9.9	8.8	1.13	6.32	5.41	1.17
43	NR_110802	RUNDC3A-AS1	5,081	10.8	8.5	1.27	7.07	5.96	1.18
44	JX088243	HELLP associated lr	205,012	13.1	10.5	1.25	8.71	7.25	1.20
45	NR_109833	PRNCR1	12,722	14.1	11.0	1.29	9.92	7.89	1.26
46	NR_001463	Xist	17,918	11.7	8.4	1.39	8.38	5.54	1.51
47	NR_001564	XIST	19,296	13.2	8.4	1.57	9.02	5.65	1.60
