# Supplementary material for: Convergence in LINE-1 nucleotide variations can benefit redundantly forming triplexes with lncRNA in mammalian X-chromosome inactivation
Source: Mob DNA. 2019 Jul 30;10:33. doi: 10.1186/s13100-019-0173-4 (PMC6664574; doi:10.1186/s13100-019-0173-4)

Additional file 3: Representative secondary structures of Rsx RNA predicted by Mfold

The numbers denoted at some loops indicate the number of nucleotides in each motif that are located in the single-stranded loops of Rsx RNA. Note that the motifs in the Rsx AG-12 domain, positions 4,141–4,630 and 9,821–10,620 are only redundant-AG (r-AG) motifs because of the lack of redundant-UC (r-UC) motifs in these regions (see Figure 3a). The RNA secondary structures predicted for each of these two regions in the Rsx AG-12 domain are similar, and a representative Structure 1 is presented. On the other hand, those predicted for the Rsx UC-dominant domain are varied, and three examples are presented here (Structures 1–3).

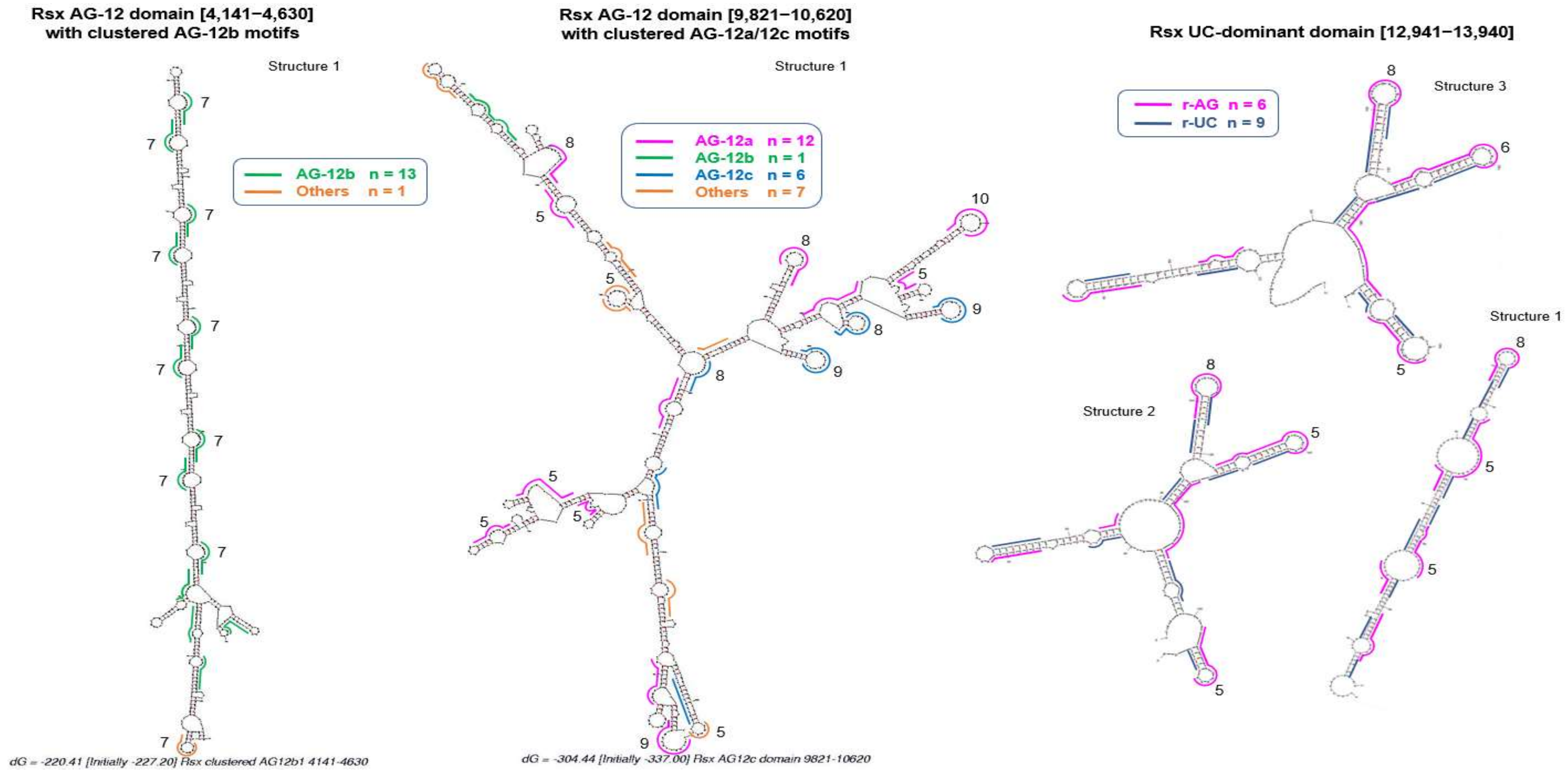

Supplement: Supplementary file 3 — Representative secondary structures of Rsx RNA predicted by Mfold. The numbers denoted at some loops indicate the number of nucleotides in each motif that are located in the single-stranded loops of Rsx RNA. Note that the motifs in the Rsx AG-12 domain, positions 4,141–4,630 and 9,821–10,620 are only redundant-AG (r-AG) motifs because of the lack of redundant-UC (r-UC) motifs in these regions (see Fig. 3a). The RNA secondary structures predicted for each of these two regions in the Rsx AG-12 domain are similar, and a representative Structure 1 is presented. On the other hand, those predicted for the Rsx UC-dominant domain are varied, and three examples are presented here (Structures 1–3). (PDF 282 kb) [file 13100_2019_173_MOESM3_ESM.pdf]
