# Supplementary material for: Convergence in LINE-1 nucleotide variations can benefit redundantly forming triplexes with lncRNA in mammalian X-chromosome inactivation
Source: Mob DNA. 2019 Jul 30;10:33. doi: 10.1186/s13100-019-0173-4 (PMC6664574; doi:10.1186/s13100-019-0173-4)
Supplement: Supplementary file 4 — Proportions of L1s, L2s, and other families of indicated length ranges on X chromosomes. This table shows the details of Fig. 4b, indicating that the maximum length of L2s is shorter than that of L1s in the three species. (PDF 340 kb) [file 13100_2019_173_MOESM4_ESM.pdf]

Additional file 4: Proportions of L1s, L2s, and other families of indicated length ranges on X chromosome

This table shows the details of Figure 4b, indicating that the maximum length of L2s is shorter than that of L1s in the three species.

Opossum

LINE bp	≤100	100 <	200 <	300 <	400 <	500 <	600 <	700 <	800 <	900 <	1,000 <	2,000 <	3,000 <	4,000 <	5,000 <	6,000 <	7,000 <	Total(%)
L1s	0.23	0.86	0.99	1.03	1.04	1.01	1.05	0.81	0.72	0.65	4.54	2.37	1.62	1.18	0.63	2.17	0.04	20.94
L2s	0.48	0.95	0.71	0.60	0.48	0.40	0.29	0.23	0.19	0.10	0.51	0.05	0.00	0.00	0.00	0.00	0.00	4.98
Others	0.36	0.98	0.86	0.65	0.47	0.33	0.25	0.18	0.15	0.10	0.35	0.09	0.07	0.05	0.00	0.00	0.00	4.88
Total	1.06	2.79	2.56	2.29	1.99	1.74	1.59	1.22	1.06	0.85	5.40	2.51	1.69	1.23	0.63	2.17	0.04	30.80

Mouse

LINE bp	≤100	100 <	200 <	300 <	400 <	500 <	600 <	700 <	800 <	900 <	1,000 <	2,000 <	3,000 <	4,000 <	5,000 <	6,000 <	7,000 <	Total(%)
L1s	0.40	1.52	1.81	1.80	1.77	1.72	1.48	1.24	1.29	2.17	6.22	2.83	1.83	1.74	3.16	2.69	0.17	33.83
L2s	0.06	0.14	0.10	0.06	0.04	0.03	0.02	0.01	0.01	0.00	0.01	0.00	0.00	0.00	0.00	0.00	0.00	0.48
Others	0.02	0.03	0.02	0.01	0.01	0.00	0.00	0.00	0.00	0.00	0.00	0.00	0.00	0.00	0.00	0.00	0.00	0.09
Total	0.48	1.69	1.93	1.88	1.82	1.76	1.51	1.24	1.30	2.17	6.22	2.83	1.83	1.74	3.16	2.69	0.17	34.41

Human

LINE bp	≤100	100 <	200 <	300 <	400 <	500 <	600 <	700 <	800 <	900 <	1,000 <	2,000 <	3,000 <	4,000 <	5,000 <	6,000 <	7,000 <	Total(%)
L1s	0.36	1.28	1.46	1.52	1.39	1.34	1.21	1.14	1.07	0.93	6.37	3.54	2.17	1.26	1.08	2.85	0.22	29.19
L2s	0.25	0.62	0.52	0.45	0.34	0.25	0.17	0.15	0.11	0.08	0.22	0.02	0.00	0.00	0.00	0.00	0.00	3.19
Others	0.07	0.15	0.13	0.08	0.05	0.03	0.03	0.01	0.01	0.01	0.01	0.00	0.00	0.00	0.00	0.00	0.00	0.58
Total	0.69	2.05	2.11	2.05	1.78	1.62	1.41	1.30	1.19	1.02	6.61	3.56	2.17	1.26	1.08	2.85	0.22	32.96
