# Supplementary material for: Convergence in LINE-1 nucleotide variations can benefit redundantly forming triplexes with lncRNA in mammalian X-chromosome inactivation
Source: Mob DNA. 2019 Jul 30;10:33. doi: 10.1186/s13100-019-0173-4 (PMC6664574; doi:10.1186/s13100-019-0173-4)
Supplement: Supplementary file 5 — A list of 50 randomly selected L1s of the three species. Start and end positions on X chromosomes, subfamilies, and length were based on RepeatMasker annotations. (+) and (−) represent plus and minus strand DNAs, respectively. L1s longer than 7,000 bp are fewer than 50 in number, namely 46 for human, 39 for mouse, and 4 for opossum, all of which were used in Fig. 5a. (PDF 719 kb) [file 13100_2019_173_MOESM5_ESM.pdf]

Additional file 5: A list of randomly selected 50 L1s of the three species

Start and end positions on X chromosomes, subfamilies, and length were based on RepeatMasker annotations. (+) and (–) represent plus and minus strand DNAs, respectively. L1s longer than 7,000 bp are fewer than 50 in number, namely 46 for human, 39 for mouse, and 4 for opossum, all of which were used in Figure 5a.

hg38ChrX

<=100	Start	End	Subfamily	Length	
1	2412232	2412263	L1MA8	32	+
2	2523530	2523568	L1MC3	39	+
3	15040957	15040996	L1MB5	40	-
4	15300899	15300948	L1ME4b	50	+
5	21171017	21171105	HAL1ME	89	-
6	22507992	22508075	L1ME3A	84	+
7	24038717	24038773	L1MB8	57	+
8	24194227	24194297	HAL1	71	-
9	32229590	32229681	L1MA10	92	-
10	33564030	33564090	L1M2	61	+
11	34696922	34696966	L1PA6	45	-
12	36924592	36924633	L1M1	42	+
13	44343998	44344082	L1ME3Cz	85	-
14	45605305	45605391	L1PA15	87	-
15	45672592	45672653	L1MA7	62	+
16	46501957	46502025	L1MEd	69	+
17	46891107	46891139	L1ME4b	33	+
18	47434658	47434703	L1M5	46	+
19	47793933	47794000	L1MD2	68	+
20	50294240	50294257	L1MC4	18	-
21	52196748	52196815	L1ME3A	68	+
22	66817740	66817818	L1PA10	79	-
23	67985269	67985333	L1PB3	65	+
24	68384389	68384437	L1PB2	49	+
25	73886356	73886368	L1PA15-16	13	+
26	77344765	77344851	HAL1b	87	+
27	83781533	83781618	L1MA6	86	-
28	85658571	85658669	L1MA9	99	-
29	86699659	86699682	L1PB	24	+
30	87707786	87707845	L1M2	60	+
31	102158827	102158877	L1ME3Cz	51	-
32	104198393	104198433	L1MC5	41	-
33	108511031	108511117	L1MC4	87	-
34	109234631	109234704	L1MD1	74	+
35	111365218	111365295	L1ME3G	78	+
36	118908517	118908534	L1MA9	18	-
37	122888100	122888155	L1M6	56	+
38	123858651	123858694	L1MB4	44	+
39	128963426	128963505	L1ME2	80	+
40	133717260	133717309	L1M4	50	+
41	134421839	134421913	L1ME4a	75	-
42	136901679	136901764	L1MD	86	-
43	137737668	137737766	L1MC1	99	-
44	140423597	140423643	L1MC2	47	+
45	142979312	142979390	L1ME3G	79	+
46	146020502	146020590	L1MA6	89	+
47	147833065	147833102	HAL1	38	+
48	148272467	148272550	HAL1ME	84	-
49	152467873	152467895	L1M5	23	+
50	154733769	154733840	L1MD	72	-
>100	Start	End	Subfamily	Length	
1	3686862	3687009	L1MB3	148	-
2	16809231	16809344	L1ME4b	114	-
3	20100277	20100399	L1ME3	123	+
4	22016177	22016313	L1M5	137	-
5	24524740	24524850	HAL1ME	111	+
6	32236280	32236462	L1ME3Cz	183	-
7	32262231	32262365	L1ME1	135	+
8	33839187	33839334	L1MC4	148	+
9	35608818	35608977	L1MA9	160	+
10	36328846	36328971	L1M5	126	+
11	38873864	38873979	L1ME4a	116	-
12	52439603	52439738	L1MA3	136	-
13	54268127	54268239	L1MC4	113	+
14	65656071	65656236	L1ME3G	166	-
15	70073976	70074098	L1ME3G	123	+
16	72771118	72771239	L1MEh	122	-
17	73989544	73989734	L1MEf	191	+
18	78031958	78032060	L1MB7	103	-
19	83001902	83002044	L1ME3Cz	143	+
20	83057546	83057646	L1MB7	101	+
21	83406637	83406744	L1MA3	108	+
22	88052742	88052911	L1MA9	170	+
23	90290798	90290978	L1MEc	181	-
24	91545289	91545394	L1PA13	106	+
25	94161311	94161426	L1PA15	116	+
26	95974352	95974509	L1MA8	158	-
27	100951256	100951420	L1PA17	165	+
28	104536722	104536847	L1MEg	126	-
29	106175737	106175886	L1ME4c	150	-
30	113093901	113094038	L1PB1	138	+
31	116204953	116205113	L1MB1	161	+
32	125416213	125416406	L1ME2	194	+
33	125471800	125471989	L1MD3	190	+
34	126032474	126032601	L1PA3	128	-
35	131410795	131410912	L1MA4A	118	+
36	131701514	131701617	L1M3	104	+
37	132900560	132900747	L1MB8	188	+
38	133463636	133463752	L1PA15	117	-
39	134993573	134993710	L1ME4b	138	+
40	136374770	136374896	L1ME2z	127	-
41	136595410	136595565	L1MD2	156	-
42	141139297	141139420	L1PA11	124	+
43	145533007	145533171	L1M5	165	-
44	146681082	146681206	L1ME3Cz	125	-
45	147680841	147681023	L1ME4a	183	+
46	150957323	150957499	L1ME5	177	+
47	151030697	151030815	L1MA7	119	-
48	151859958	151860116	L1M4	159	+
49	152887774	152887969	L1ME3G	196	+
50	153836368	153836525	L1ME3F	158	-
>200	Start	End	Subfamily	Length	
1	853301	853549	L1MA6	249	+
2	3614012	3614215	L1MA4A	204	+
3	3772203	3772414	L1MB5	212	-
4	10036340	10036595	L1MB5	256	-
5	18561216	18561420	L1ME4a	205	+
6	19907453	19907738	L1PB1	286	+
7	20899318	20899544	HAL1	227	+
8	24690880	24691087	L1PB1	208	-
9	26671825	26672041	L1MA9	217	+
10	26681336	26681581	L1P4	246	+
11	29779400	29779618	L1PA15	219	+

mm10ChrX

<=100	Start	End	Subfamily	Length	
1	5740037	5740123	L1VL2	87	-
2	6479189	6479214	L1M4a2	26	-
3	13341567	13341640	L1MC4a	74	-
4	22074488	22074532	L1_Mur1	45	-
5	41043245	41043296	L1_Mur2	52	-
6	41861030	41861076	Lx3_Mus	47	+
7	43712816	43712879	L1M4c	64	+
8	43752837	43752868	L1MB7	32	+
9	50774009	50774036	L1_Mus1	28	+
10	62731062	62731125	Lx8	64	+
11	55221797	55221809	L1_Mus1	13	+
12	60485245	60485310	L1MC4	66	+
13	60868279	60868360	L1M5	82	+
14	61424439	61424509	Lx2B2	71	+
15	61744480	61744523	L1_Mur1	44	-
16	65875990	65876080	Lx3C	91	-
17	67105559	67105595	Lx8b	37	+
18	73820245	73820316	L1M4	72	+
19	78092028	78092086	Lx9	59	-
20	79977559	79977615	L1_Mus1	57	-
21	81592464	81592497	Lx5	34	+
22	84314162	84314227	Lx5c	66	+
23	85216229	85216313	L1ME4a	85	-
24	88930654	88930722	L1_Mur2	69	-
25	92795397	92795495	Lx10	99	+
26	94263971	94264067	Lx3_Mus	97	-
27	98045109	98045206	L1_Mur3	98	-
28	99723263	99723327	L1M5	65	+
29	103665435	103665519	Lx2	85	-
30	104274574	104274673	L1MC	100	-
31	126554009	126554022	Lx3A	14	-
32	106055078	106055159	L1_Mus3	82	+
33	106914988	106915081	L1MB8	94	-
34	128044222	128044285	L1_Mus1	64	+
35	137481578	137481640	Lx	63	+
36	118552774	118552870	L1M4	97	-
37	132254683	132254744	Lx6	62	+
38	155549047	155549136	Lx9	90	-
39	133986824	133986881	Lx8b	58	+
40	163379059	163379137	L1MB4	79	-
41	164276176	164276260	L1Md_A	85	+
42	140119892	140119976	L1MA9	85	+
43	143815061	143815122	Lx9	62	-
44	146126446	146126497	L1_Mus3	52	+
45	147177015	147177102	L1MA4	88	-
46	148985319	148985373	Lx5b	55	+
47	152928448	152928519	L1M2	72	+
48	153879406	153879454	Lx6	49	-
49	156086661	156086754	L1MB1	94	+
50	165185777	165185872	Lx9	96	-
>100	Start	End	Subfamily	Length	
1	5607898	5608078	L1_Mur2	181	-
2	7477122	7477300	L1M3	179	+
3	9820212	9820341	Lx8	130	-
4	19723027	19723202	L1M4	176	-
5	21359740	21359873	L1_Mur3	134	+
6	29656522	29656633	Lx4A	112	-
7	30286826	30286984	L1_Mus3	159	-
8	36120250	36120394	L1M4c	145	-
9	39499347	39499459	Lx8b	113	-
10	45167326	45167433	Lx9	108	-
11	43118347	43118545	Lx6	199	-
12	47039135	47039328	Lx6	194	+
13	51655629	51655755	L1MD	127	+
14	48437589	48437716	L1MB8	128	+
15	52769240	52769425	L1MA9	186	+
16	48645208	48645326	Lx	119	-
17	55893221	55893321	L1_Mus1	101	-
18	57530565	57530726	L1ME4a	162	-
19	61729451	61729633	Lx2	183	-
20	78930935	78931111	L1_Mur2	177	+
21	79110940	79111105	Lx	166	-
22	95648205	95648388	L1_Rod	184	+
23	97286254	97286379	L1MA8	126	-
24	98418497	98418673	L1M2	177	+
25	100787451	100787564	Lx8	114	-
26	101657215	101657389	L1MA7	175	+
27	104363226	104363381	L1MA9	156	-
28	113706474	113706667	L1MC2	194	+
29	109944512	109944705	L1M2	194	-
30	111011784	111011895	Lx9	112	-
31	112016233	112016337	Lx2	105	-
32	114080503	114080700	L1M3c	198	-
33	114539887	114540010	L1MA5	124	-
34	114968439	114968604	L1MCa	166	-
35	117335893	117336076	Lx3A	184	-
36	121049888	121049994	L1Md_F3	107	-
37	121808027	121808206	L1_Mur1	180	+
38	126675619	126675737	L1MCa	119	-
39	132655174	132655358	Lx5b	185	+
40	135372627	135372738	L1_Mus3	112	+
41	143498740	143498935	L1MB2	196	+
42	146618932	146619078	Lx	147	-
43	149496300	149496435	L1_Mur1	136	-
44	151783950	151784084	L1_Rod	135	-
45	154070789	154070910	L1_Mur2	122	+
46	155349176	155349358	L1MB8	183	-
47	156840272	156840376	Lx7	105	+
48	157119075	157119178	Lx9	104	+
49	168416486	168416664	L1M4b	179	+
50	169499779	169499887	L1_Mus3	109	+
>200	Start	End	Subfamily	Length	
1	3871408	3871621	Lx2	214	-
2	12423628	12423862	L1MC3	235	+
3	13043942	13044191	L1ME2	250	-
4	13684733	13685008	L1ME3Cz	276	-
5	16278827	16279100	Lx5c	274	-
6	16416248	16416475	Lx2	228	-
7	17767716	17767923	Lx8	208	-
8	21166540	21166831	Lx8b	292	-
9	27878264	27878492	Lx6	229	+
10	39151309	39151578	L1MA4	270	+
11	48188926	48189133	Lx8	208	+

Mdo5ChrX

≦100	Start	End	Subfamily	Length	
1	6142992	6143036	L1-1_MD	45	-
2	13286334	13286404	L1_Mdo3a	71	-
3	14566124	14566176	L1_Mars1a	53	+
4	27988270	27988360	HAL1_Opos	91	+
5	47772379	47772473	L1_Mdo1	95	+
6	50600832	50600926	L1_Opos0	95	-
7	51904691	51904738	L1_Opos	48	+
8	64571896	64571982	L1_Mdo3b	87	+
9	67056921	67056968	HAL1_Opos	48	+
10	69019738	69019821	L1_Opos0	84	+
11	4884668	4884757	L1_Opos4b	90	+
12	5447254	5447302	L1_Mars1b	49	+
13	5673501	5673600	L1_Mars1b	100	+
14	6200536	6200587	L1_Mdo3	52	-
15	9088048	9088147	L1_Mars1b	100	-
16	10817993	10818041	L1_Opos0	49	+
17	11815798	11815849	L1_Mdo3b	52	+
18	12004534	12004596	L1_Opos	63	+
19	14289157	14289215	L1_Mdo2	59	-
20	15316700	15316761	L1_Mars1a	62	+
21	15489054	15489132	L1_Mars1	79	-
22	16156917	16156968	L1_Mars1a	52	-
23	17633417	17633495	L1_Opos	79	-
24	17939695	17939789	HAL1_Opos	95	+
25	20032689	20032728	L1_Mdo3	40	-
26	20692365	20692403	L1_Mdo3a	39	-
27	21774483	21774546	HAL1_Opos	64	-
28	23052747	23052811	L1_Mdo3c	65	-
29	23110741	23110805	L1_Opos	65	-
30	25849614	25849675	L1_Mdo1	62	-
31	26500712	26500781	L1_Mdo2	70	-
32	32362558	32362650	HAL1_Opos	93	+
33	33264288	33264354	L1_Opos0	67	+
34	35530957	35531008	L1_Mdo5	52	+
35	37586646	37586704	L1_Mars1b	59	-
36	40027613	40027691	L1_Mars1	79	-
37	43139214	43139308	L1_Mdo3c	95	+
38	45491070	45491146	L1_Mdo5	77	-
39	45847819	45847918	L1_Mdo3c	100	+
40	51592589	51592677	L1_Opos3	89	-
41	52888844	52888889	L1_Mars1	46	-
42	54022293	54022377	L1_Mdo2	85	+
43	56001805	56001844	L1_Mdo3b	40	+
44	56069873	56069898	L1_Mars1a	26	+
45	62764956	62765031	L1_Mars1	76	-
46	66897845	66897935	L1_Mdo3c	91	-
47	70190739	70190832	L1_Mars1b	94	-
48	70995958	70996047	L1_Mdo3a	90	+
49	75274807	75274853	L1_Mars1b	47	+
50	75492944	75493032	L1_Mars1b	89	+
>100	Start	End	Subfamily	Length	
1	5499838	5499946	L1_Mdo3a	109	+
2	9058064	9058251	L1_Mars1b	188	+
3	9618659	9618782	L1_Opos2	124	-
4	10696480	10696651	L1_Mars1b	172	-
5	17038073	17038268	L1_Mdo5	196	-
6	21291953	21292147	L1_Mars1b	195	+
7	22056637	22056747	L1_Mars1b	111	+
8	27829623	27829726	L1_Mars1b	104	-
9	33275935	33276095	L1_Mdo6	161	+
10	37813379	37813482	HAL1_Opos	104	-
11	38385166	38385356	L1_Mars1	191	-
12	38953853	38953977	L1_Mars1b	125	+
13	40812807	40812943	L1_Mdo3b	137	+
14	47188355	47188458	L1_Mars1	104	-
15	54144387	54144519	L1_Opos2	133	-
16	57999893	58000000	L1_Mars1	108	+
17	58506071	58506240	HAL1-2a_MD	170	-
18	6363715	6363839	L1_Mdo4	125	+
19	7721796	7721985	L1_Mars1b	190	-
20	8272442	8272555	L1_Mars1b	114	+
21	8599706	8599879	L1_Mars1b	174	-
22	9345869	9345987	L1_Opos1	119	+
23	10426122	10426290	HAL1N1_MD	169	+
24	12594149	12594258	L1_Mars1	110	-
25	13284230	13284330	L1_Opos2	101	+
26	15162158	15162283	L1_Opos	126	+
27	18784828	18785024	L1_Opos	197	+
28	19018180	19018304	HAL1_Opos	125	+
29	23271147	23271253	L1_Mars1	107	+
30	23433168	23433269	L1_Mdo4	102	-
31	25670296	25670469	HAL1_Opos1	174	+
32	28001307	28001453	L1_Mdo3a	147	+
33	30354891	30355045	L1_Mdo6	155	+
34	30741696	30741806	L1_Mdo3b	111	-
35	36324650	36324815	L1_Mdo6	166	-
36	37398190	37398319	L1M5	130	-
37	38620583	38620688	HAL1_Opos	106	+
38	42889089	42889227	L1_Mdo5	139	+
39	46441053	46441174	L1_Mdo6	122	-
40	51524388	51524522	L1_Mars1b	135	-
41	51548482	51548627	L1_Opos0	146	+
42	54513148	54513320	L1_Mars1	173	+
43	55139730	55139929	L1_Mdo3a	200	-
44	58474425	58474579	L1_Mars1b	155	+
45	60143416	60143614	L1_Mdo5	199	-
46	60184379	60184521	L1_Mdo1	143	-
47	61094449	61094613	L1_Mdo4	165	-
48	65914378	65914567	L1_Opos0	190	-
49	67960710	67960826	L1_Mars1	117	+
50	73743407	73743559	L1_Opos	153	-
>200	Start	End	Subfamily	Length	
1	3353148	3353386	L1_Mdo4	239	+
2	4943130	4943331	L1_Opos3	202	-
3	8504877	8505123	L1_Opos4b	247	-
4	8619123	8619397	L1_Mars1b	275	-
5	16094842	16095117	L1_Opos0	276	-
6	17536387	17536596	L1_Mdo3a	210	+
7	18725021	18725235	L1_Mdo3	215	+
8	24405812	24406044	L1_Opos	233	+
9	25752224	25752452	HAL1N1_MD	229	-
10	25799736	25800020	L1_Mdo4	285	-
11	28800758	28801033	L1_Mdo3c	276	-

12	34262315	34262575	L1MC4a	261	+
13	35181152	35181411	L1MB5	260	-
14	35214543	35214749	L1M4	207	-
15	37057354	37057630	L1MA9	277	-
16	41294711	41294980	L1MC5	270	+
17	41838152	41838356	L1MD2	205	-
18	43150689	43150943	HAL1	255	-
19	44592484	44592728	L1ME3Cz	245	-
20	48373064	48373318	L1MD2	255	-
21	48739659	48739913	L1MD	255	+
22	53657082	53657291	L1MC1	210	+
23	54578042	54578325	L1ME1	284	-
24	56135724	56135975	L1PA15	252	+
25	69044937	69045207	L1M5	271	-
26	74618578	74618866	L1ME4a	289	+
27	75670911	75671117	L1MA5	207	-
28	75895926	75896145	L1PA7	220	-
29	79358260	79358524	L1PA3	265	-
30	80707863	80708091	L1MC5	229	-
31	85010099	85010362	L1MC	264	-
32	87722029	87722239	L1ME2	211	-
33	101034441	101034732	L1MC5	292	+
34	101970353	101970614	L1ME2z	262	-
35	107237486	107237702	L1MB5	217	-
36	117214968	117215180	L1MA8	213	+
37	121852059	121852342	L1M1	284	+
38	121884338	121884584	HAL1ME	247	-
39	122519942	122520143	L1MB4	202	+
40	132047783	132047986	L1MA8	204	-
41	136837553	136837783	L1MD2	231	-
42	140016269	140016501	L1P1	233	-
43	140185091	140185307	L1MC5	217	-
44	144828967	144829177	L1MC5	211	+
45	147105032	147105258	L1MC	227	-
46	147567066	147567277	HAL1b	212	+
47	150046421	150046666	HAL1M8	246	-
48	153293569	153293865	L1MB5	297	-
49	155002676	155002961	L1MB2	286	-
50	155177686	155177982	L1MEf	297	-
>300	Start	End	Subfamily	Length	
1	2712051	2712409	L1MA8	359	+
2	2986689	2987008	L1PA4	320	-
3	7214551	7214919	L1PB1	369	-
4	10875053	10875357	L1ME4b	305	-
5	13586054	13586367	L1MB7	314	+
6	13701071	13701433	L1MB7	363	-
7	14990340	14990722	L1MA9	383	+
8	17869376	17869733	L1ME4c	358	-
9	19860293	19860639	L1MB4	347	+
10	24243859	24244227	L1ME3G	369	-
11	34177230	34177583	L1MD2	354	+
12	35609116	35609416	L1M1	301	+
13	35671675	35672039	L1PA16	365	-
14	35752521	35752879	L1MB1	359	+
15	36997502	36997866	L1M4	365	+
16	37748935	37749309	HAL1	375	+
17	39710028	39710397	HAL1	370	+
18	39993460	39993843	L1MB7	384	+
19	47694104	47694422	L1MC4	319	+
20	51914358	51914672	L1MA4	315	-
21	52154808	52155111	L1MA9	304	+
22	54052897	54053244	L1P1	348	-
23	54148015	54148364	L1ME1	350	+
24	54703191	54703564	L1ME3Cz	374	+
25	65155257	65155571	L1MEi	315	-
26	65337914	65338231	L1PA8	318	-
27	74872204	74872559	L1MA3	356	+
28	76683174	76683535	L1PB4	362	-
29	80361835	80362159	L1PA16	325	-
30	84021738	84022101	L1MB7	364	-
31	84327383	84327762	L1ME1	380	+
32	89607761	89608155	L1M5	395	-
33	92484520	92484847	L1PB1	328	+
34	93117931	93118289	L1PA15	359	+
35	94805071	94805421	L1P1	351	-
36	96533787	96534089	L1MA7	303	+
37	106538639	106538982	L1M4	344	-
38	107047679	107048064	L1MEg	386	-
39	108086627	108086938	L1ME4a	312	+
40	117773804	117774129	L1MB7	326	+
41	126582188	126582500	L1M4c	313	+
42	126746241	126746607	L1MC2	367	-
43	130337005	130337349	L1M5	345	-
44	134519471	134519831	L1PA10	361	-
45	135185637	135186032	L1MA6	396	+
46	139361184	139361554	L1MC	371	+
47	140332216	140332532	L1MA7	317	-
48	144511306	144511620	L1PA17	315	+
49	150797445	150797762	L1MB4	318	+
50	155791593	155791919	L1MA1	327	+
>400	Start	End	Subfamily	Length	
1	12059462	12059946	L1PA16	485	-
2	14216013	14216488	L1MC5a	476	+
3	17112321	17112806	L1MDb	486	+
4	17993733	17994193	L1ME4a	461	-
5	20412419	20412836	L1PA8A	418	-
6	22085591	22086004	L1M3f	414	-
7	27732019	27732480	L1MA4	462	-
8	28759407	28759863	L1MC5a	457	+
9	31696095	31696594	L1ME3A	500	+
10	34922127	34922564	L1MEg1	438	+
11	41570385	41570877	L1ME1	493	-
12	43033632	43034129	L1MB7	498	-
13	44943541	44944026	L1ME1	486	-
14	46502962	46503406	L1MEi	445	+
15	47476367	47476773	L1MB8	407	+
16	48451746	48452148	L1M4	403	+
17	50859316	50859733	L1MB4	418	-
18	54528125	54528619	L1ME3E	495	-
19	55594625	55595040	L1ME3G	416	-
20	56156914	56157394	L1PA5	481	+
21	67033262	67033682	L1MA3	421	-
22	67865901	67866346	L1PA17	446	-
23	68974838	68975288	L1MC1	451	-
24	72852440	72852910	L1MC5	471	-
25	73074852	73075265	L1M4	414	+
26	76122508	76122941	L1MD2	434	+
27	77803331	77803762	L1M4	432	-
28	78013615	78014089	L1ME4c	475	-
29	78874219	78874696	L1PA10	478	+
30	82487276	82487676	L1MB3	401	+
31	84590835	84591300	L1PREC2	466	+
32	86751746	86752228	L1PA16	483	+

12	52263358	52263619	Lx4B	262	+
13	49819871	49820134	Lx8	264	+
14	56766748	56766963	L1ME4b	216	+
15	62179936	62180221	L1_Mus4	286	-
16	62469492	62469776	L1_Mur3	285	-
17	63581910	63582204	Lx2	295	+
18	65574910	65575148	L1Md_F3	239	+
19	66107682	66107941	L1MB1	260	+
20	67082932	67083177	L1_Mus1	246	+
21	68619616	68619844	Lx5	229	+
22	69139336	69139564	L1MA6	229	-
23	76661976	76662187	L1_Mus1	212	+
24	84447784	84448076	L1MA9	293	-
25	89333775	89334035	L1MC1	261	+
26	91982756	91983027	L1Md_F2	272	+
27	92343216	92343446	L1M4a1	231	+
28	93397897	93398126	L1MD2	230	-
29	95317665	95317902	L1MB4	238	-
30	97388374	97388661	Lx8	288	-
31	101872378	101872594	L1MB8	217	-
32	107915710	107915952	L1_Mus3	243	+
33	109366860	109367107	L1MA4	248	-
34	109469382	109469595	L1_Mus2	214	-
35	110429248	110429449	Lx7	202	+
36	117161027	117161248	L1MA4	222	+
37	120557529	120557771	L1MB8	243	-
38	121864552	121864844	L1_Mus3	293	-
39	132240527	132240751	Lx2B2	225	-
40	128049393	128049617	L1_Mus2	225	+
41	129925810	129926030	L1_Mus1	221	-
42	138744890	138745160	Lx9	271	+
43	141802886	141803156	L1M3	271	+
44	142434796	142435028	Lx9	233	+
45	144512804	144513062	Lx2B	259	-
46	145993027	145993263	L1_Mur2	237	+
47	146917989	146918274	L1M4b	286	+
48	149645403	149645688	Lx	286	-
49	153744507	153744751	Lx10	245	-
50	163752654	163752977	Lx8	224	-
>300	Start	End	Subfamily	Length	
1	5967363	5967681	L1MEf	319	+
2	12343831	12344158	L1_Mus4	328	-
3	13621894	13622289	L1_Mus1	396	-
4	15600651	15601002	Lx7	352	+
5	18731379	18731698	L1M3b	320	-
6	21507248	21507573	L1_Mm	326	+
7	36163307	36163614	Lx8b	308	-
8	39811935	39812287	L1_Mus4	353	+
9	40646887	40647205	Lx3C	319	-
10	42238443	42238781	L1M2	339	-
11	42803788	42804123	L1_Mur1	336	+
12	43443216	43443519	Lx9	304	+
13	44819187	44819578	Lx9	392	-
14	46389222	46389620	L1_Mus3	399	+
15	46520790	46521138	Lx9	349	-
16	47979577	47979878	L1MB2	302	+
17	52084206	52084512	Lx9	307	-
18	53174210	53174554	L1_Mus1	345	+
19	59009062	59009437	Lx2	376	+
20	64306471	64306839	Lx4A	369	+
21	66691177	66691517	Lx2	341	+
22	72364546	72364941	L1Md_F2	396	+
23	72664307	72664651	L1MEf	345	+
24	76723038	76723352	L1Md_F2	315	-
25	78319006	78319341	L1_Mur2	336	-
26	83786329	83786694	Lx	366	-
27	88608417	88608742	Lx7	326	-
28	92785727	92786051	L1MC1	325	+
29	94222493	94222872	Lx5	380	+
30	95392807	95393170	L1M2	364	-
31	96354974	96355281	Lx5	308	+
32	97875434	97875807	Lx8b	374	-
33	98669164	98669503	Lx8b	340	+
34	105296015	105296315	L1MB5	301	-
35	106039581	106039946	Lx5c	366	-
36	108270406	108270762	L1_Mus1	357	+
37	108491456	108491779	L1Md_F3	324	-
38	111111817	111112159	L1_Mus3	343	-
39	113690317	113690674	L1_Mur2	358	+
40	113999428	113999803	Lx8	376	-
41	127460531	127460891	Lx7	361	-
42	133455851	133456195	L1_Mur2	345	+
43	135319161	135319558	L1_Mur2	398	-
44	137697071	137697449	Lx6	379	-
45	143967583	143967924	Lx8	342	+
46	147186640	147186980	Lx	341	-
47	147310700	147311081	L1Md_F2	382	-
48	148413052	148413354	L1_Mus3	303	+
49	156593728	156594077	L1Md_F2	350	+
50	165220474	165220830	L1_Rod	357	+
>400	Start	End	Subfamily	Length	
1	14554476	14554918	L1ME3A	443	+
2	18790807	18791234	L1MDb	428	-
3	21633037	21633440	L1MB2	404	-
4	23456942	23457349	Lx5	408	-
5	25655520	25655969	L1_Mur2	450	+
6	34131148	34131558	L1VL4	411	-
7	36584795	36585286	L1_Mus1	492	+
8	39956698	39957122	L1Md_F2	425	+
9	40215982	40216395	L1Md_F2	414	-
10	49400444	49400889	Lx8	446	-
11	49880253	49880738	L1_Mus1	486	-
12	50269377	50269827	Lx5	451	-
13	53924198	53924671	L1M2	474	-
14	54124388	54124851	L1MEj	464	+
15	59306298	59306758	L1_Mus1	461	+
16	60101198	60101609	L1Md_F2	412	+
17	64455456	64455895	Lx4B	440	-
18	70629379	70629805	Lx8	427	+
19	70998505	70998905	Lx9	401	+
20	73725335	73725778	L1MB7	444	-
21	74185866	74186286	L1_Mur1	421	+
22	82997157	82997575	L1Md_F3	419	-
23	84306099	84306571	L1_Mus2	473	-
24	84762962	84763444	L1Md_F3	483	+
25	86973014	86973438	Lx7	425	+
26	88238740	88239237	L1MB7	498	+
27	89351806	89352299	L1Md_A	494	-
28	91103940	91104428	HAL1	489	+
29	96481185	96481585	L1MA5A	401	-
30	98178118	98178585	Lx9	468	-
31	105655921	105656349	Lx4A	429	+
32	107273117	107273569	Lx8b	453	-

12	41407279	41407520	L1_Mdo3a	242	+
13	44641455	44641733	L1_Opos2	279	-
14	45921859	45922129	L1_Mdo3c	271	-
15	50915578	50915811	L1_Mdo3b	234	+
16	55648303	55648594	HAL1_Opos1	292	+
17	58616995	58617241	L1_Mars1b	247	+
18	59613918	59614163	L1-1_MD	246	-
19	60469583	60469788	L1_Opos	206	+
20	61775532	61775786	L1_Mars1b	255	+
21	70210344	70210617	L1_Opos0	274	-
22	71269990	71270238	L1_Mdo3c	249	+
23	78104082	78104345	L1_Mdo6	264	+
24	5001365	5001660	L1_Mdo3b	296	-
25	5930492	5930748	L1_Mdo1	257	-
26	8662819	8663056	L1_Opos	238	+
27	19275335	19275537	HAL1-2a_MD	203	-
28	20189624	20189830	L1_Mdo5	207	+
29	22549441	22549658	L1_Mdo3c	218	-
30	22868742	22868984	L1_Opos4b	243	+
31	28024303	28024590	HAL1_Opos	288	-
32	30513424	30513719	L1_Opos	296	-
33	34322778	34322988	L1_Mars1b	211	-
34	38111707	38111986	L1_Mdo6	280	+
35	38612816	38613058	L1_Opos	243	-
36	41867251	41867451	L1_Opos0	201	+
37	41956611	41956878	L1_Opos	268	-
38	43580045	43580341	HAL1_Opos	297	+
39	57267794	57268009	HAL1_Opos	216	+
40	62696063	62696351	L1_Opos0	289	+
41	66015325	66015536	L1_Opos2	212	-
42	68046127	68046408	L1_Mdo4	282	+
43	68820367	68820612	L1_Opos4b	246	-
44	69261795	69262007	L1_Opos4b	213	+
45	70249796	70250031	L1_Mdo5	236	-
46	74111083	74111349	L1_Mdo3b	267	-
47	75275953	75276176	L1_Mars1b	224	+
48	75693627	75693897	L1_Opos	271	+
49	76924268	76924567	L1_Mars1b	300	-
50	77324130	77324423	L1_Mdo3c	294	-
>300	Start	End	Subfamily	Length	
1	4855402	4855706	L1_Mdo3	305	+
2	9925067	9925457	L1_Mdo3c	391	+
3	19010116	19010463	L1_Opos	348	+
4	23976743	23977082	L1_Opos4b	340	+
5	24783462	24783825	L1_Opos0	364	+
6	26345862	26346211	L1_Opos2	350	-
7	27697046	27697395	L1_Mdo3	350	+
8	28098472	28098844	HAL1_Opos	373	+
9	36162321	36162679	L1_Opos0	359	+
10	36460736	36461122	L1_Opos0	387	-
11	38611439	38611780	L1_Opos	342	+
12	45166142	45166459	L1_Mdo3b	318	-
13	46405633	46405983	HAL1_Opos	351	+
14	47213982	47214357	L1_Opos0	376	-
15	50623935	50624253	L1_Mdo5	319	-
16	58664356	58664714	L1_Mars1b	359	-
17	61065494	61065819	HAL1_Opos	326	-
18	64196996	64197305	L1_Mdo3a	310	+
19	66326629	66326996	L1_Mdo6	368	+
20	66646982	66647309	L1_Mars1b	328	-
21	66723863	66724205	HAL1_Opos	343	-
22	72415465	72415798	L1_Mars1b	334	+
23	4997274	4997619	L1_Mdo3b	346	-
24	7862207	7862587	L1_Mars1	381	+
25	10326287	10326602	L1_Opos2	316	+
26	10783720	10784107	HAL1N1_MD	388	+
27	13589662	13590021	L1_Mdo2	360	-
28	18663055	18663404	L1_Opos0	350	+
29	20329371	20329726	L1_Mars1	356	-
30	21986376	21986710	L1_Mdo3c	335	+
31	25057499	25057804	L1_Mdo2	306	+
32	26955712	26956110	L1_Mars1	399	+
33	30601468	30601836	L1_Opos4b	369	-
34	31400058	31400371	L1_Mdo3a	314	+
35	32509623	32510004	L1_Opos3	382	+
36	33103988	33104334	L1_Mdo3c	347	-
37	36213920	36214305	HAL1N1_MD	386	-
38	36933081	36933400	L1_Opos2	320	-
39	38514673	38515052	L1_Mdo3b	380	+
40	42726947	42727272	L1_Opos4b	326	+
41	46229951	46230300	L1_Opos0	350	-
42	47297339	47297697	L1_Mdo3c	359	+
43	47615140	47615528	L1-1_MD	389	-
44	48013143	48013540	L1_Mdo3c	398	-
45	49670852	49671202	HAL1_Opos	351	+
46	54700151	54700478	L1_Opos2	328	+
47	56710247	56710595	L1_Mars1	349	-
48	60237134	60237462	L1_Mdo4	329	+
49	64726873	64727252	L1_Mdo3a	380	+
50	71473570	71473952	L1_Mars1	383	-
>400	Start	End	Subfamily	Length	
1	3356121	3356536	HAL1_Opos	416	-
2	7806961	7807422	L1_Opos	462	-
3	9544211	9544619	L1_Mdo3c	409	-
4	12168209	12168645	L1_Opos2	437	-
5	12539947	12540425	L1_Mdo2	479	-
6	12683650	12684127	L1_Opos0	478	-
7	12814898	12815378	L1_Mdo3c	481	-
8	13296566	13297016	L1_Mdo3	451	+
9	13976457	13976928	L1_Opos	472	+
10	19555402	19555802	L1_Mars1b	401	+
11	21191188	21191663	L1_Mars1b	476	+
12	25020446	25020938	L1_Opos0	493	-
13	32138320	32138804	L1_Mars1b	485	-
14	41471840	41472321	L1_Opos1	482	+
15	47649088	47649527	L1_Mars1b	440	-
16	52851382	52851830	L1_Opos	449	-
17	53349596	53350031	L1_Opos	436	+
18	54201460	54201916	L1_Opos3	457	+
19	55393450	55393882	L1_Mdo3b	433	-
20	56378019	56378500	L1_Mars1b	482	-
21	58291451	58291933	L1_Opos0	483	-
22	60403717	60404175	L1_Opos4b	459	+
23	67295774	67296192	L1_Mars1b	419	-
24	67873506	67873969	L1_Opos2	464	-
25	68275603	68276032	L1_Mdo5	430	+
26	74082387	74082814	L1_Mdo3c	428	-
27	15426623	15427072	L1_Mdo6	450	-
28	16240128	16240591	L1_Mdo6	464	-
29	20206841	20207272	L1_Mdo3a	432	-
30	21606792	21607275	L1_Mdo4	484	-
31	22652898	22653374	L1_Mdo3	477	+
32	24678430	24678889	L1_Mdo3a	460	+

33	89442793	89443273	L1MA3	481	-
34	94151826	94152308	L1PA6	483	+
35	98144926	98145369	L1PA16	444	-
36	102437523	102437951	L1MC4a	429	+
37	107269726	107270201	L1M4	476	+
38	117798325	117798797	L1PA7	473	-
39	134279864	134280315	L1MD	452	-
40	137670889	137671327	L1PA10	439	-
41	139545918	139546360	L1PA14	443	-
42	139568547	139569001	L1MA2	455	+
43	140556363	140556832	L1MA4	470	+
44	141270275	141270772	L1MA9	498	-
45	143255727	143256137	L1M5	411	+
46	144651844	144652276	L1ME3E	433	+
47	147127954	147128404	L1MC3	451	-
48	147798031	147798439	L1MA4A	409	-
49	152092812	152093299	L1ME1	488	+
50	152464931	152465331	L1MA9	401	-
>500	Start	End	Subfamily	Length	
1	4561261	4561821	L1MA8	561	-
2	6893694	6894277	L1PA11	584	+
3	10974053	10974594	L1ME3	542	-
4	20460828	20461371	L1ME1	544	-
5	21347471	21347977	L1MDa	507	-
6	24161052	24161606	L1M5	555	+
7	27195095	27195684	L1MA4A	590	+
8	27678267	27678866	L1MB3	600	+
9	29270295	29270853	L1ME1	559	-
10	30761720	30762305	L1PREC2	586	+
11	31672777	31673323	L1MB7	547	-
12	34003652	34004170	L1PA16	519	+
13	38592771	38593364	L1MEc	594	-
14	47974865	47975408	L1ME4b	544	-
15	48505792	48506389	L1MC4	598	+
16	50550088	50550686	L1MB8	599	-
17	51884543	51885096	L1MD2	554	+
18	56514357	56514945	L1MA3	589	+
19	56666743	56667249	L1PREC2	507	+
20	63386971	63387518	L1ME1	548	-
21	66776416	66777005	L1MA1	590	-
22	67761101	67761688	L1PA3	588	-
23	76347129	76347629	L1M4a1	501	+
24	77573526	77574088	L1ME1	563	+
25	78088279	78088844	L1M1	566	-
26	82058356	82058940	L1ME1	585	+
27	84512701	84513246	L1MA4	546	+
28	87236725	87237320	L1M1	596	+
29	87238573	87239123	L1M1	551	-
30	87705994	87706526	L1M2	533	+
31	95313652	95314162	L1PB4	511	-
32	99002106	99002682	L1MCc	577	+
33	99919693	99920197	L1ME3F	505	-
34	101200455	101201039	L1ME3	585	-
35	101789456	101789991	L1MEf	536	-
36	107687770	107688292	L1MC4a	523	+
37	108103304	108103848	L1MB1	545	-
38	114159359	114159872	L1M7	514	-
39	117141781	117142342	L1MB7	562	+
40	123316172	123316748	L1ME4a	577	+
41	126581346	126581901	L1MB3	556	+
42	127947911	127948489	L1M4	579	-
43	129024645	129025159	L1M4	515	+
44	132980046	132980573	L1PA15	528	+
45	133768968	133769565	L1MA10	598	+
46	134608121	134608654	L1MB5	534	+
47	135715720	135716290	L1PA7	571	-
48	144261475	144261976	L1MA4A	502	+
49	146339965	146340520	HAL1	556	-
50	154260402	154260960	L1M8	559	-
>600	Start	End	Subfamily	Length	
1	4140687	4141359	L1MB3	673	-
2	5157181	5157870	L1ME2	690	+
3	8784550	8785170	L1PB1	621	+
4	11059280	11059882	L1ME2	603	+
5	16399546	16400169	L1MA2	624	-
6	18541819	18542517	L1MD2	699	-
7	22986087	22986699	L1MB8	613	-
8	25148913	25149522	L1MA1	610	-
9	27110407	27111055	L1P1	649	+
10	41993113	41993799	L1MB8	687	+
11	51462480	51463143	L1PA4	664	+
12	51661848	51662475	L1MC2	628	+
13	52842572	52843228	L1MCc	657	+
14	54488613	54489300	L1PA15	688	+
15	54883828	54884478	L1ME1	651	+
16	63968768	63969369	L1MCa	602	+
17	79430092	79430699	L1MA10	608	+
18	80900863	80901518	L1PREC2	656	+
19	80946342	80946976	L1PA15	635	-
20	81010983	81011619	L1MEc	637	-
21	81041460	81042091	L1MA4A	632	-
22	83001209	83001888	L1PA4	680	+
23	83968810	83969478	L1PA10	669	+
24	90042584	90043276	L1PA16	693	-
25	90166348	90167016	L1PA14	669	+
26	92816722	92817339	L1MB5	618	+
27	98457495	98458187	L1MA8	693	-
28	100537149	100537756	L1PA6	608	-
29	101571309	101571951	L1MEg	643	-
30	102308995	102309694	L1ME1	700	+
31	105448664	105449297	L1MA2	634	-
32	114205437	114206121	L1MEc	685	+
33	116060464	116061098	L1M1	635	-
34	116095137	116095741	L1M1	605	-
35	116616365	116616988	L1PA5	624	+
36	120396130	120396756	L1MEc	627	-
37	120680299	120680941	L1MA9	643	-
38	121707989	121708655	L1MEf	667	-
39	124330686	124331381	L1MB4	696	+
40	125989640	125990303	L1M4	664	+
41	127148067	127148742	L1ME1	676	+
42	127200090	127200732	L1PREC2	643	-
43	130374055	130374712	L1MC1	658	-
44	131336879	131337557	L1ME1	679	-
45	132537380	132537980	L1M5	601	-
46	135139480	135140083	L1MC	604	-
47	138995314	138995917	L1PA14	604	-
48	140776603	140777299	L1PA8	697	+
49	143785369	143786041	L1ME3	673	+
50	150424724	150425368	L1MB8	645	-
>700	Start	End	Subfamily	Length	
1	2774887	2775640	L1MB5	754	+
2	20606085	20606810	L1MA8	726	+

33	114836914	114837348	Lx9	435	-
34	115355383	115355834	Lx3C	452	-
35	115991710	115992119	Lx6	410	-
36	117748048	117748539	L1M2	492	+
37	119087859	119088322	L1Md_F2	464	+
38	121045856	121046316	L1MA4A	461	-
39	123088595	123088998	Lx6	404	-
40	125926229	125926703	L1Md_F3	475	-
41	127258892	127259309	Lx5c	418	+
42	129673144	129673598	Lx	455	+
43	133510344	133510772	L1_Mur3	429	+
44	136019567	136020064	L1M2	498	+
45	138176486	138176978	L1MC3	493	-
46	152835903	152836357	L1_Mus1	455	-
47	160938497	160938909	Lx4B	413	+
48	166634457	166634866	L1Md_T	410	+
49	167608717	167609153	Lx2B2	437	-
50	169074966	169075446	Lx8	481	+
>500	Start	End	Subfamily	Length	
1	3832516	3833054	Lx4A	539	+
2	11013599	11014198	L1MB2	600	+
3	16846927	16847453	Lx6	527	-
4	19310149	19310692	L1MA7	544	+
5	23244025	23244602	L1Md_F2	578	+
6	32454418	32454940	Lx4A	523	-
7	33348836	33349391	Lx4A	556	+
8	34247351	34247877	L1Md_Gf	527	+
9	35779352	35779896	Lx5c	545	-
10	42460940	42461480	L1Md_F2	541	+
11	45506672	45507221	L1_Mur3	550	-
12	46461966	46462508	L1Md_F2	543	-
13	52334859	52335440	L1MB8	582	+
14	51833081	51833664	L1_Mus2	584	-
15	57518412	57518931	Lx8	520	-
16	57535576	57536112	L1ME2z	537	+
17	61539808	61540363	Lx2B2	556	-
18	61956605	61957112	L1Md_F2	508	+
19	67214349	67214931	L1_Mus3	583	-
20	67800341	67800904	Lx6	564	+
21	68418635	68419212	L1_Mus2	578	+
22	78137547	78138095	L1_Rod	549	+
23	78380206	78380804	L1Md_F2	599	-
24	79157720	79158279	L1_Mus2	560	+
25	87560911	87561422	Lx8b	512	+
26	91096400	91096950	L1_Mur2	551	-
27	92045690	92046251	L1_Mus1	562	+
28	92826178	92826732	L1MA6	555	+
29	95366293	95366873	L1M3	581	+
30	97763893	97764434	Lx7	542	-
31	100340201	100340716	Lx8	516	+
32	102944560	102945113	L1VL2	554	+
33	110181983	110182579	Lx5	597	+
34	111853409	111853911	L1M2	503	+
35	114203400	114203956	L1_Mur3	557	-
36	115070046	115070560	Lx9	515	-
37	116810641	116811168	L1_Mur3	528	+
38	124439850	124440350	Lx2B	501	+
39	126639946	126640492	L1_Mus4	547	+
40	127514437	127514989	L1MA4	553	+
41	131781810	131782312	L1Md_F	503	-
42	131989765	131990302	L1_Mus3	538	-
43	148192150	148192660	Lx	511	+
44	148986876	148987414	L1_Mus3	539	-
45	151129357	151129922	Lx6	566	-
46	151706423	151706972	L1_Mur3	550	-
47	155502969	155503516	L1M2	548	-
48	167192649	167193155	L1_Mus4	507	+
49	167240609	167241113	Lx5b	505	+
50	169548901	169549404	Lx6	504	-
>600	Start	End	Subfamily	Length	
1	25564233	25564922	L1_Mus3	690	+
2	28043134	28043743	L1Md_F2	610	+
3	31770248	31770925	L1Md_F2	678	+
4	33241094	33241697	L1VL4	604	-
5	45402689	45403374	Lx7	686	+
6	45813693	45814381	L1_Mur2	689	+
7	46156661	46157325	MusHAL1	665	+
8	46553866	46554560	Lx3_Mus	695	-
9	46859389	46860043	L1Md_F2	655	+
10	47765917	47766591	Lx	675	-
11	49865557	49866176	L1VL2	620	+
12	55058002	55058645	L1VL1	644	-
13	63747774	63748427	Lx3C	654	+
14	66265309	66265973	L1_Mur2	665	+
15	75556944	75557575	Lx	632	-
16	76532223	76532883	L1VL4	661	+
17	80729837	80730452	Lx3_Mus	616	+
18	82147119	82147799	L1_Mur2	681	+
19	90496505	90497203	L1_Mus3	699	+
20	91703549	91704208	L1_Mus1	660	+
21	91921743	91922419	L1_Mus4	677	+
22	93515527	93516224	L1M3	698	-
23	95397349	95398039	L1_Mus4	691	-
24	97351002	97351697	Lx9	696	+
25	104021899	104022569	Lx7	671	-
26	111393989	111394633	L1MEc	645	+
27	113635875	113636567	Lx8	693	-
28	114319822	114320508	L1_Mur2	687	-
29	116475435	116476052	Lx8	618	-
30	116765766	116766388	L1_Mur1	623	+
31	121779562	121780170	L1_Mus4	609	+
32	121791850	121792517	Lx9	668	+
33	132391813	132392430	L1MEg	618	-
34	132592941	132593549	Lx	609	+
35	132995842	132996495	Lx6	654	+
36	133366276	133366921	L1_Mus3	646	-
37	135861702	135862362	Lx10	661	-
38	136020537	136021172	Lx7	636	-
39	137493375	137494013	L1_Mus4	639	-
40	142077682	142078370	Lx2B2	689	-
41	146774986	146775644	Lx2	659	-
42	147482432	147483044	L1Md_F3	613	+
43	148557140	148557829	L1_Mus1	690	-
44	151383621	151384269	Lx3_Mus	649	-
45	153049196	153049848	Lx8	653	-
46	154706415	154707026	Lx	612	-
47	155654876	155655480	L1_Rod	605	-
48	155966395	155967091	L1_Mur2	697	-
49	163226349	163226990	Lx6	642	-
50	168501354	168502026	L1Md_A	673	+
>700	Start	End	Subfamily	Length	
1	4161412	4162125	L1_Mur3	714	-
2	8742419	8743132	L1_Rod	714	-

33	25608783	25609280	L1_Opos0	498	-
34	27257613	27258026	L1_Mdo3c	414	-
35	36813595	36814045	L1_Mars1b	451	+
36	37655969	37656382	L1_Mars1b	414	+
37	37756802	37757222	L1_Mdo1	421	+
38	40176049	40176454	L1_Opos0	406	-
39	40661148	40661614	L1_Opos0	467	-
40	42287071	42287524	L1_Mdo3c	454	-
41	43251357	43251757	L1_Opos4b	401	-
42	43504269	43504728	L1_Mars1b	460	-
43	43792860	43793344	L1_Mars1b	485	+
44	46483257	46483711	L1_Mdo2	455	+
45	46907719	46908215	L1_Mdo5	497	+
46	49670430	49670856	L1_Mars1b	427	+
47	56889310	56889758	L1_Mdo3b	449	-
48	57557354	57557782	L1_Mdo3c	429	+
49	62621480	62621971	L1_Mars1b	492	-
50	78491008	78491485	L1_Opos	478	+
>500	Start	End	Subfamily	Length	
1	4297684	4298221	L1_Opos0	538	-
2	7767486	7768004	L1_Mdo6	519	-
3	11637262	11637773	L1_Mdo6	512	+
4	12721723	12722236	L1_Mdo4	514	+
5	13986259	13986760	L1_Opos	502	+
6	15246885	15247388	L1_Mdo3b	504	+
7	17585350	17585905	L1_Mars1	556	+
8	19161599	19162103	HAL1_Opos1	505	-
9	20172948	20173463	L1_Mars1b	516	-
10	23433418	23433925	L1_Mdo4	508	-
11	25194267	25194824	HAL1_Opos1	558	+
12	25629213	25629771	HAL1_Opos1	559	+
13	27566194	27566762	L1_Mars1	569	+
14	31698350	31698857	L1_Mdo3c	508	+
15	34333822	34334412	L1_Mdo5	591	+
16	38078923	38079487	L1_Opos	565	-
17	39179176	39179728	L1_Opos	553	-
18	39194744	39195277	L1_Mdo3a	534	+
19	43503310	43503858	L1_Mars1b	549	-
20	48395508	48396104	L1_Mdo2	597	+
21	48990718	48991316	L1_Opos0	599	-
22	49039263	49039776	L1_Mdo3b	514	-
23	49055447	49056006	L1-1_MD	560	-
24	51538581	51539098	L1_Opos	518	+
25	54063769	54064298	L1_Mdo5	530	-
26	55283607	55284152	L1_Opos0	546	+
27	56426695	56427242	L1_Mars1a	548	+
28	61871573	61872088	L1_Mdo3a	516	-
29	63888724	63889281	L1_Opos2	558	-
30	67339533	67340060	L1_Mdo3	528	-
31	68718272	68718806	L1_Opos4b	535	+
32	75757253	75757838	L1_Opos	586	-
33	76277104	76277652	L1_Opos2	549	-
34	76871463	76872056	HAL1_Opos1	594	-
35	77726517	77727058	L1_Opos3	542	+
36	79100661	79101173	L1_Opos2	513	-
37	4070794	4071322	L1_Mdo3c	529	+
38	6086481	6087009	L1_Mdo6	529	+
39	16801733	16802252	L1_Opos	520	+
40	20712718	20713301	L1_Mdo3c	584	-
41	24402881	24403384	L1_Mdo5	504	-
42	27992660	27993206	L1_Mdo3b	547	+
43	30689746	30690327	HAL1_Opos	582	+
44	33260352	33260864	L1_Mars1b	513	-
45	36486346	36486937	L1_Mars1b	592	-
46	38945775	38946327	L1_Opos	553	+
47	60929944	60930480	L1_Opos0	537	+
48	67209272	67209830	HAL1_Opos1	559	-
49	69104042	69104639	L1_Opos	598	+
50	79136115	79136639	L1_Mdo4	525	-
>600	Start	End	Subfamily	Length	
1	3286442	3287075	L1_Opos2	634	+
2	10858269	10858905	L1_Mars1b	637	+
3	13322901	13323578	L1_Mdo3c	678	+
4	14335421	14336115	L1_Opos1	695	+
5	16065938	16066586	L1_Mars1b	649	+
6	18085279	18085919	HAL1_Opos	641	+
7	18650947	18651644	L1_Mdo5	698	-
8	20784734	20785383	L1_Mars1	650	+
9	21037114	21037788	L1_Mdo3b	675	-
10	23973809	23974457	L1_Opos	649	-
11	25918355	25919043	HAL1N1_MD	689	-
12	26180545	26181233	L1_Mdo3	689	-
13	26584464	26585104	L1_Mdo5	641	-
14	27058377	27059056	L1_Mdo3a	680	+
15	34152683	34153371	HAL1_Opos	689	+
16	34914949	34915606	L1_Opos0	658	+
17	35575208	35575867	L1_Opos0	660	-
18	37370775	37371412	L1_Mdo3b	638	+
19	38732069	38732706	HAL1_Opos	638	+
20	38885773	38886390	L1_Opos1	618	+
21	42865649	42866258	L1_Opos1	610	-
22	43685651	43686295	L1_Mars1b	645	-
23	43957064	43957670	L1_Mars1b	607	-
24	44348174	44348806	L1_Opos0	633	-
25	46027379	46028016	L1_Mdo3b	638	+
26	48468256	48468864	L1_Opos4b	609	-
27	61850896	61851500	L1_Opos1	605	+
28	62445142	62445804	L1_Opos2	663	+
29	63779286	63779927	L1_Opos1	642	+
30	65929738	65930379	L1_Opos3	642	+
31	66318312	66318979	L1_Mdo3c	668	+
32	68412719	68413358	L1_Mdo3b	640	+
33	69657939	69658539	L1_Opos1	601	-
34	73549771	73550378	L1_Opos0	608	-
35	74368765	74369394	L1_Mdo3b	630	-
36	75064262	75064908	L1_Mdo3a	647	-
37	76145643	76146326	L1_Mdo5	684	+
38	78938505	78939138	L1_Mdo3b	634	-
39	5561724	5562411	L1_Mars1b	688	-
40	9555475	9556086	L1_Mdo3c	612	+
41	9941008	9941676	L1_Mdo3b	669	-
42	25704449	25705137	HAL1N1_MD	689	-
43	25855455	25856105	L1_Mdo3b	651	-
44	29924198	29924860	L1_Opos	663	-
45	31209328	31209974	L1_Opos2	647	-
46	31252928	31253614	L1_Mdo3b	687	+
47	66255763	66256451	L1_Mars1b	689	+
48	66299814	66300440	L1_Mars1b	627	-
49	68620928	68621585	L1_Mars1b	658	+
50	76872495	76873179	L1_Mars1	685	-
>700	Start	End	Subfamily	Length	
1	7608064	7608765	L1_Mdo4	702	+
2	10832042	10832823	L1_Opos	782	-

3	20789599	20790306	L1ME4b	708	-
4	21445306	21446063	L1MA5	758	-
5	27733156	27733889	L1MA4	734	-
6	32399853	32400557	L1P1	705	-
7	32841264	32841966	L1M5	703	+
8	32854637	32855384	L1MA3	748	+
9	33732453	33733232	L1ME3	780	+
10	34308321	34309079	L1MB3	759	+
11	35484290	35485002	L1PA13	713	-
12	35985141	35985907	L1PA13	767	-
13	44081740	44082469	L1MC5	730	-
14	45566497	45567198	L1MA3	702	+
15	47072640	47073409	L1PA2	770	+
16	47445433	47446144	L1ME3A	712	+
17	47471631	47472420	L1ME3A	790	+
18	52468644	52469382	L1M5	739	-
19	64876506	64877246	L1MA2	741	+
20	69330109	69330878	L1MA4	770	-
21	69376613	69377387	L1M4	775	+
22	70953255	70953959	L1MB7	705	+
23	72402762	72403545	L1MB7	784	-
24	74211024	74211772	L1ME1	749	+
25	76719274	76720009	L1PREC2	736	-
26	80196648	80197391	L1MA9	744	-
27	83853055	83853807	L1M3b	753	+
28	84196441	84197180	L1MA2	740	-
29	85820285	85820992	L1PREC2	708	+
30	90506530	90507294	L1MA1	765	+
31	91300623	91301374	L1M3	752	+
32	93687526	93688314	L1MA6	789	-
33	94814152	94814866	L1PA13	715	-
34	96099786	96100501	L1ME1	716	+
35	96756164	96756911	L1MB8	748	-
36	98231559	98232358	L1PREC2	800	+
37	111704063	111704854	L1MB8	792	+
38	113026707	113027425	L1MA2	719	-
39	113118081	113118822	L1MC5a	742	+
40	117649843	117650622	L1MA2	780	+
41	119369588	119370300	L1ME3F	713	-
42	121045852	121046624	L1M3	773	-
43	128993929	128994672	L1MA4A	744	+
44	135602291	135603077	L1PA11	787	-
45	138634645	138635395	L1PB2	751	+
46	139273345	139274065	L1MA8	721	+
47	145444640	145445342	L1ME2z	703	+
48	148141629	148142352	L1PA11	724	+
49	151906088	151906872	L1M5	765	+
50	154101802	154102515	L1MA6	714	+

>800	Start	End	Subfamily	Length	
1	3997845	3998669	L1PA17	825	-
2	7452393	7453211	L1PA4	819	+
3	16018291	16019163	L1MB5	873	-
4	35899591	35900439	L1MD2	849	-
5	36642017	36642822	L1MA4A	806	-
6	37725413	37726308	L1MC1	896	+
7	37831993	37832794	L1ME3Cz	802	+
8	41845888	41846743	L1MA2	856	+
9	42140249	42141147	L1M4a2	899	-
10	56507257	56508148	L1PBa1	892	+
11	63048983	63049800	L1P1	818	+
12	63975241	63976041	L1MCa	801	+
13	66669890	66670707	L1ME3A	818	-
14	66787812	66788655	L1MDa	844	+
15	68175828	68176696	L1MB2	869	+
16	70305185	70306073	L1MB7	889	-
17	71805192	71806063	L1M1	872	+
18	75674650	75675511	L1M4	862	+
19	77561736	77562546	L1ME1	811	-
20	79058578	79059419	L1P1	842	+
21	81322322	81323127	L1MA3	806	-
22	81604891	81605748	L1MB8	858	+
23	82539807	82540684	L1MD	878	-
24	85738556	85739423	L1M4b	868	+
25	87532818	87533714	L1PA3	897	+
26	88259951	88260771	L1MB5	821	-
27	93319129	93320023	L1P3	895	+
28	94560538	94561414	L1PBa	877	-
29	100088000	100088813	L1ME3A	814	+
30	107035478	107036321	L1HS	844	+
31	119182588	119183461	L1MD1	874	+
32	122853021	122853897	L1MA2	877	+
33	124758188	124759061	L1MB1	874	+
34	125626715	125627562	L1PB3	848	+
35	127759170	127759990	L1PA7	821	-
36	131495314	131496163	L1MEd	850	+
37	131976432	131977242	L1PA10	811	-
38	137603099	137603946	L1M1	848	+
39	140675001	140675890	L1PA15	890	+
40	141960450	141961342	L1MC3	893	-
41	143130694	143131560	L1M7	867	+
42	144155557	144156388	L1MA2	832	+
43	144707972	144708808	L1MEc	837	-
44	145551895	145552776	L1MA4A	882	-
45	145888341	145889183	L1PB4	843	-
46	146663145	146664013	L1MDb	869	+
47	147050466	147051362	L1ME1	897	+
48	148061871	148062723	L1MA2	853	-
49	151193614	151194445	L1MA3	832	+
50	152125800	152126620	L1M5	821	-

>900	Start	End	Subfamily	Length	
1	4567888	4568844	L1MCa	957	-
2	9192329	9193236	HAL1M8	908	+
3	14456220	14457212	L1MA5A	993	-
4	16617949	16618889	L1MB7	941	-
5	25096423	25097392	L1MA9	970	-
6	25468073	25468993	L1MA8	921	-
7	25861152	25862123	L1PB1	972	-
8	27862674	27863640	L1MA8	967	+
9	32615705	32616690	L1MC3	986	-
10	33404383	33405324	L1PA13	942	-
11	40355697	40356622	L1ME3B	926	+
12	45832795	45833786	L1MEg2	992	+
13	50715493	50716487	L1MA8	995	+
14	52307111	52308096	L1MC2	986	+
15	56417976	56418881	L1P4e	906	+
16	71678061	71679057	L1M1	997	-
17	76285867	76286792	L1MA2	926	-
18	79744010	79744962	L1PA3	953	+
19	80781680	80782616	L1MA3	937	+
20	82887403	82888337	L1MA9	935	+
21	83731872	83732798	L1MA4	927	-
22	86998999	86999951	L1M1	953	+
23	89265111	89266093	L1MA10	983	-

3	9689028	9689747	L1Md_F2	720	+
4	9918341	9919131	L1VL4	791	-
5	14153541	14154293	L1MC1	753	-
6	14890062	14890795	L1_Mus3	734	+
7	19935282	19936045	L1VL4	764	-
8	20108986	20109711	L1Md_F2	726	-
9	28732064	28732860	Lx3C	797	-
10	33110159	33110924	L1Md_F2	766	+
11	34309019	34309744	L1_Mur2	726	-
12	45618452	45619241	Lx2	790	+
13	45792938	45793651	Lx3A	714	+
14	45937829	45938594	L1_Mus3	766	-
15	49975617	49976364	L1MB4	748	-
16	56350027	56350756	Lx5	730	-
17	56443729	56444501	Lx6	773	-
18	58355859	58356633	Lx3C	775	-
19	66699573	66700286	Lx7	714	+
20	67666042	67666781	L1_Mus1	740	-
21	67985307	67986021	Lx8	715	-
22	69079291	69080013	L1_Mus4	723	-
23	69627215	69628001	L1Md_F2	787	-
24	89624996	89625755	L1_Mus2	760	-
25	95743040	95743740	Lx7	701	-
26	96140434	96141167	Lx8	734	-
27	97750226	97750957	Lx7	732	+
28	97883682	97884403	L1_Mur3	722	-
29	100752759	100753471	Lx	713	-
30	104010382	104011093	Lx3_Mus	712	+
31	105502335	105503079	L1VL4	745	-
32	107182108	107182878	Lx8b	771	+
33	111548657	111549430	Lx3B	774	+
34	112411958	112412710	Lx2B2	753	-
35	112596892	112597675	L1Md_F2	784	+
36	113689025	113689731	L1Md_T	707	+
37	113785032	113785748	Lx3A	717	-
38	117053804	117054591	Lx5b	788	-
39	117634830	117635562	L1Md_A	733	+
40	122465642	122466377	Lx5	736	-
41	129173414	129174126	Lx7	713	+
42	134823565	134824353	L1_Mus1	789	+
43	138418466	138419173	Lx5	708	-
44	139477385	139478128	Lx	744	-
45	145199325	145200072	L1_Mus3	748	-
46	145344104	145344853	Lx4A	750	+
47	153494271	153494993	Lx7	723	+
48	154451515	154452254	L1_Mus1	740	-
49	160008635	160009414	L1Md_F	780	+
50	167870576	167871290	L1_Mus3	715	+

>800	Start	End	Subfamily	Length	
1	6102666	6103558	L1Md_F3	893	-
2	8871239	8872123	Lx4B	885	+
3	9976185	9977066	L1_Mur1	882	-
4	16368867	16369726	L1_Mus1	860	-
5	23395472	23396350	L1MA5	879	+
6	24455430	24456248	Lx2	819	+
7	29368523	29369356	Lx	834	+
8	32307129	32308018	L1_Mur2	890	+
9	38286272	38287166	Lx5	895	+
10	40223574	40224376	Lx7	803	-
11	40360776	40361583	Lx3A	808	-
12	44234017	44234854	L1Md_F2	838	+
13	45598580	45599414	L1Md_F2	835	+
14	46833131	46833994	Lx5b	864	-
15	47520415	47521220	L1Md_A	806	+
16	49137279	49138139	L1MDa	861	-
17	54448676	54449522	Lx	847	-
18	59608460	59609319	L1_Rod	860	+
19	62963327	62964175	Lx3A	849	+
20	67911763	67912590	L1Md_F2	828	-
21	68049782	68050620	Lx6	839	-
22	68094311	68095205	L1_Mur3	895	-
23	68965181	68965984	Lx6	804	-
24	70704490	70705296	L1_Mus1	807	-
25	81766779	81767617	L1_Mur1	839	-
26	84867931	84868759	Lx5	829	-
27	91956601	91957461	L1Md_F3	861	+
28	95395593	95396396	L1VL4	804	-
29	97006696	97007525	L1_Mus4	830	+
30	105455707	105456565	Lx	859	+
31	107126593	107127394	L1_Mus2	802	-
32	108936631	108937525	L1_Mur3	895	+
33	110986730	110987539	Lx5c	810	-
34	119898322	119899138	L1_Mus4	817	-
35	120775606	120776485	Lx5c	880	-
36	123871379	123872278	L1Md_F3	900	+
37	124932377	124933264	L1Md_F3	888	-
38	126500000	126500840	L1Md_A	841	-
39	133080166	133080969	L1_Mus3	804	+
40	144954169	144954995	L1Md_A	827	-
41	146561321	146562153	L1M2	833	+
42	146891025	146891921	Lx	897	+
43	150046499	150047357	Lx5	859	+
44	150673088	150673922	L1_Mus3	835	-
45	155025322	155026193	Lx6	872	-
46	158021244	158022143	Lx8	900	+
47	161007382	161008270	Lx5	889	+
48	162604709	162605580	Lx7	872	+
49	165714371	165715226	L1_Mus2	856	+
50	167626028	167626845	Lx4B	818	+

>900	Start	End	Subfamily	Length	
1	5946182	5947120	Lx9	939	-
2	9545549	9546534	L1Md_F2	986	+
3	14394600	14395538	L1MA8	939	-
4	15593963	15594889	L1_Mur3	927	+
5	16445782	16446778	L1Md_A	997	+
6	19948647	19949590	Lx5	944	-
7	23659459	23660433	L1Md_F2	975	+
8	23980293	23981276	L1Md_A	984	+
9	24181072	24182052	L1Md_F2	981	+
10	25544250	25545243	L1Md_F2	994	+
11	26282885	26283868	L1Md_F2	984	+
12	36722289	36723284	L1Md_F2	996	+
13	39810957	39811935	L1Md_T	979	+
14	42833270	42834247	Lx8	978	+
15	50107523	50108437	L1Md_F2	915	+
16	50522886	50523875	L1Md_F2	990	+
17	50681251	50682224	L1Md_F3	974	+
18	55016289	55017276	L1Md_A	988	+
19	57051725	57052655	L1Md_F2	931	+
20	62590477	62591447	Lx	971	+
21	67660307	67661297	L1Md_F2	991	-
22	67784743	67785739	L1Md_T	997	+
23	72585480	72586476	L1Md_T	997	+

3	12860017	12860797	L1_Mdo4	781	-
4	14638320	14639035	L1_Opos	716	-
5	16127494	16128220	L1_Mdo4	727	+
6	21355190	21355985	L1_Mdo1	796	+
7	25108377	25109091	L1_Mdo3b	715	+
8	25187157	25187872	L1_Mdo3b	716	-
9	25937828	25938542	L1_Mdo3b	715	+
10	27704673	27705457	L1_Mdo3a	785	-
11	28775492	28776247	L1_Mdo3c	756	-
12	29500002	29500796	L1_Opos	795	-
13	35472280	35473013	L1_Mdo2	734	+
14	35922313	35923020	L1_Opos	708	-
15	35943672	35944441	L1_Opos1	770	+
16	39435272	39435980	L1_Mars1b	709	-
17	39811082	39811824	L1_Mdo5	743	-
18	40706834	40707538	L1_Opos0	705	+
19	50244662	50245417	L1_Mdo5	756	-
20	51364408	51365199	L1_Mars1	792	-
21	59339537	59340321	L1_Mars1a	785	-
22	59634964	59635715	L1_Mdo6	752	+
23	63749971	63750706	L1_Mars1b	736	-
24	66377331	66378039	L1_Mars1b	709	-
25	67883534	67884247	L1_Mars1	714	+
26	70255693	70256440	L1_Mdo3a	748	+
27	73941037	73941775	L1_Mdo3c	739	-
28	76134468	76135188	L1_Opos0	721	-
29	77904137	77904892	L1_Opos2	756	-
30	78218285	78219044	HAL1_Opos	760	+
31	79202869	79203612	L1_Opos2	744	+
32	4421237	4421971	L1_Opos0	735	-
33	4556509	4557286	L1_Opos	778	-
34	11657919	11658645	HAL1_Opos	727	-
35	16366382	16367144	L1_Opos1	763	-
36	24087563	24088271	L1_Mdo3b	709	+
37	26032028	26032770	L1-1_MD	743	+
38	26706100	26706853	L1_Mdo5	754	-
39	28837176	28837935	L1_Mdo6	760	-
40	30815512	30816271	HAL1_Opos	760	-
41	40890222	40890969	L1_Mars1	748	+
42	47097174	47097892	L1_Mdo2	719	-
43	50362230	50362943	HAL1_Opos	714	-
44	51456274	51457045	L1_Mdo3a	772	-
45	52149515	52150256	L1_Mdo3b	742	-
46	56262813	56263585	L1_Mdo1	773	-
47	66751640	66752432	HAL1_Opos	793	-
48	71937804	71938549	L1_Opos0	746	-
49	77052479	77053260	L1_Opos2	782	-
50	78197434	78198145	HAL1_Opos	712	+

>800	Start	End	Subfamily	Length	
1	7719176	7720029	L1_Mdo3b	854	-
2	12637091	12637985	L1_Mars1	895	-
3	18283153	18284033	L1_Mdo5	881	+
4	22602280	22603162	L1_Opos0	883	-
5	25384410	25385271	L1_Opos	862	+
6	28470602	28471461	L1-1_MD	860	-
7	30106752	30107644	L1_Opos0	893	+
8	30602055	30602952	L1_Mdo3b	898	+
9	33815183	33816002	L1_Mdo3b	820	-
10	35145825	35146625	L1_Mdo3a	801	+
11	36247440	36248275	L1_Mars1b	836	-
12	37546162	37547058	L1_Opos1	897	-
13	39813811	39814614	L1_Mdo6	804	-
14	41959375	41960175	L1_Mdo5	801	+
15	50132733	50133603	L1_Opos	871	-
16	52462334	52463218	L1_Opos4b	885	+
17	52758096	52758978	L1_Mdo3a	883	-
18	55150834	55151675	L1_Mdo5	842	-
19	55599080	55599923	HAL1_Opos	844	+
20	58631379	58632243	HAL1N1_MD	865	-
21	59073020	59073919	L1_Mdo4	900	-
22	59083312	59084202	L1_Mdo4	891	-
23	59090979	59091838	L1_Opos0	860	+
24	59115554	59116442	L1_Mars1	889	-
25	61716722	61717605	HAL1_Opos1	884	+
26	62825770	62826601	L1_Opos2	832	-
27	64187218	64188092	L1_Opos0	875	-
28	64233925	64234733	L1_Mdo5	809	-
29	64638771	64639642	L1_Opos4b	872	+
30	69365767	69366592	L1_Opos1	826	+
31	74315207	74316072	L1_Mars1	866	+
32	74359905	74360801	L1_Opos	897	-
33	76105189	76106037	L1_Mdo3	849	-
34	76681488	76682314	L1_Mdo4	827	-
35	13778102	13778952	L1_Mars1b	851	+
36	14354424	14355229	L1_Mdo4	806	-
37	19875883	19876748	L1_Opos0	866	+
38	21778068	21778963	L1_Mdo4	896	+
39	25226681	25227559	L1_Opos	879	+
40	38299983	38300874	L1_Opos3	892	+
41	38748941	38749804	L1_Mdo4	864	+
42	53354676	53355496	L1_Mars1b	821	-
43	56988874	56989770	L1_Mars1	897	+
44	59990342	59991214	L1_Mars1	873	-
45	61311969	61312805	L1_Mdo3a	837	+
46	64179818	64180691	L1_Mdo4	874	+
47	70659008	70659903	L1_Opos	896	+
48	70946943	70947774	L1_Opos	832	+
49	73539180	73540069	L1_Opos2	890	-
50	77793812	77794685	L1_Opos	874	+

>900	Start	End	Subfamily	Length	
1	3008943	3009906	L1_Opos2	964	-
2	12062147	12063052	L1_Opos2	906	-
3	12107638	12108624	L1_Mdo5	987	+
4	12175403	12176386	L1_Mdo3b	984	+
5	12571226	12572126	L1_Mdo3c	901	-
6	15016036	15016992	L1_Mdo3a	957	-
7	19599379	19600288	L1_Opos3	910	+
8	22327196	22328156	L1_Opos1	961	-
9	22931376	22932296	HAL1_Opos1	921	+
10	23989713	23990675	L1_Mars1	963	+
11	24434022	24434957	L1_Opos2	936	-
12	24521262	24522168	L1_Mdo3a	907	+
13	28988361	28989307	L1_Mdo2	947	+
14	29192764	29193691	L1_Mdo3b	928	+
15	31575394	31576295	L1_Mdo3a	902	+
16	32992481	32993425	L1_Opos	945	-
17	34830048	34831047	L1_Mars1a	1000	-
18	35412614	35413600	L1_Mdo3c	987	+
19	36187184	36188119	L1_Opos1	936	-
20	36355446	36356347	L1_Opos	902	+
21	38611810	38612712	HAL1N1_MD	903	+
22	44484718	44485691	L1_Opos1	974	+
23	45346165	45347153	L1_Mars1	989	+

24	91160015	91161000	L1MA2	986	-
25	94664990	94665964	L1M5	975	-
26	98250557	98251519	L1PA7	963	-
27	98467232	98468178	L1PA13	947	+
28	101866762	101867737	L1P1	976	-
29	102021156	102022062	L1M3c	907	+
30	106676592	106677498	L1M4	907	-
31	117104164	117105087	L1MA8	924	+
32	125754147	125755105	L1MB5	959	+
33	126605248	126606166	L1MB4	919	+
34	127664734	127665716	L1PA13	983	+
35	128205137	128206100	L1PA15	964	+
36	128505863	128506834	L1MA9	972	-
37	129084993	129085893	L1PA3	901	+
38	132138801	132139780	L1PB4	980	-
39	132431072	132432006	L1MC4a	935	-
40	136969170	136970123	L1ME1	954	-
41	137649595	137650517	L1MA6	923	+
42	140275523	140276458	L1MD	936	-
43	144219579	144220537	L1M3f	959	-
44	146108522	146109479	L1MA4A	958	-
45	149161267	149162196	L1PREC2	930	+
46	149767147	149768092	L1ME1	946	+
47	151349809	151350748	L1M5	940	+
48	152563643	152564605	L1MA5A	963	+
49	153014608	153015564	L1PA15	957	+
50	155243475	155244411	L1M2	937	-
>1000	Start	End	Subfamily	Length	
1	6346830	6347856	L1MEd	1027	-
2	8976902	8978817	L1PA16	1916	-
3	20658913	20660430	L1P1	1518	-
4	24094723	24096044	L1MD1	1322	-
5	25838458	25839960	L1PB3	1503	+
6	28021973	28023848	L1MB4	1876	-
7	30328837	30329985	L1MB7	1149	-
8	38242027	38243222	L1ME1	1196	+
9	45576395	45578281	L1MA8	1887	-
10	47898438	47900068	L1ME1	1631	-
11	54942392	54943469	L1MEd	1078	+
12	63215053	63217043	L1M2	1991	-
13	63282821	63284524	L1PA16	1704	+
14	65563848	65565651	L1PA7	1804	-
15	66478713	66479822	L1MDa	1110	+
16	66811024	66812485	L1PB1	1462	-
17	68215023	68216498	L1MC2	1476	+
18	70690869	70691962	L1MA9	1094	+
19	71969612	71970774	L1MB8	1163	-
20	72557221	72559029	L1PBa	1809	+
21	75084494	75085599	L1ME1	1106	+
22	76132209	76133947	L1PA11	1739	+
23	76479779	76481022	L1PB1	1244	-
24	80579616	80580910	L1M1	1295	-
25	82003118	82005068	L1P3	1951	-
26	84606979	84608399	L1P4	1421	-
27	87795316	87797229	L1PB	1914	+
28	88246245	88247772	L1MB7	1528	-
29	90474739	90476129	L1MB8	1391	+
30	91507653	91508928	L1PREC2	1276	+
31	104547692	104548954	L1PA3	1263	-
32	105366246	105368013	L1MA7	1768	-
33	106357279	106358410	L1PA13	1132	-
34	110967699	110969017	L1MD2	1319	-
35	114794378	114795800	L1PA4	1423	-
36	116989047	116990635	L1MC3	1589	+
37	121720411	121722018	L1MEf	1608	-
38	121764412	121765662	L1MB1	1251	+
39	124760519	124761706	L1PA6	1188	+
40	126330973	126332727	L1PA16	1755	-
41	131789961	131791350	L1M4	1390	-
42	139668768	139670076	L1MB8	1309	+
43	143154303	143155624	L1PA3	1322	-
44	146706748	146708167	L1PA16	1420	-
45	146777389	146779201	L1PA15	1813	+
46	147393295	147394328	L1PA13	1034	+
47	148616576	148617648	L1PA4	1073	-
48	152234275	152235376	L1P4	1102	-
49	154917300	154918767	L1MA4	1468	-
50	155901520	155903230	L1PA3	1711	-
>2000	Start	End	Subfamily	Length	
1	5471234	5473448	L1PREC2	2215	+
2	12662195	12665148	L1PA7	2954	-
3	14172986	14175277	L1MA2	2292	-
4	32794616	32796751	L1MA4A	2136	+
5	34343976	34346565	L1PA8A	2590	+
6	35596208	35598539	L1PB1	2332	+
7	36624867	36627013	L1MA3	2147	-
8	43612021	43614216	L1MA2	2196	-
9	46052010	46054575	L1PA4	2566	-
10	46142897	46145233	L1MA1	2337	+
11	50557239	50559794	L1PB1	2556	+
12	51018802	51021520	L1PA7	2719	-
13	54986904	54988923	L1MB7	2020	+
14	55360028	55362733	L1PA10	2706	+
15	55734261	55736385	L1PBa	2125	-
16	55945476	55947648	L1PA3	2173	+
17	57662326	57664836	L1PA10	2511	+
18	58030267	58032656	L1PREC2	2390	-
19	58040260	58042649	L1PA13	2390	+
20	64491965	64494665	L1MB7	2701	+
21	66138257	66140435	L1MA1	2179	-
22	66654525	66657411	L1MA4A	2887	+
23	67916825	67918943	L1PA7	2119	-
24	72661774	72663922	L1M2	2149	+
25	72967768	72970683	L1PA6	2916	+
26	73469363	73472131	L1P4a	2769	-
27	75170336	75172536	L1PB1	2201	-
28	78205547	78207913	L1PA7	2367	+
29	78933688	78936061	L1PA13	2374	-
30	81310768	81312857	L1PB1	2090	-
31	81656238	81659146	L1MA4A	2909	-
32	84969152	84972106	L1PA8A	2955	-
33	86336355	86339187	L1P2	2833	+
34	90196147	90198828	L1P2	2682	+
35	92563885	92565942	L1MA2	2058	+
36	92796588	92799149	L1MA2	2562	+
37	94809142	94812058	L1PREC2	2917	-
38	95332068	95335029	L1PBa	2962	+
39	99167235	99169842	L1PB3	2608	+
40	102912635	102915179	L1PB1	2545	+
41	110558906	110561867	L1PA10	2962	-
42	116317195	116319486	L1M1	2292	+
43	116837076	116839284	L1M1	2209	-
44	117674333	117676779	L1PA15	2447	+

24	75443656	75444601	Lx	946	-
25	82792846	82793746	L1Md_T	901	+
26	84466832	84467808	Lx3A	977	-
27	88434140	88435123	L1Md_F3	984	+
28	91778475	91779394	L1_Mus2	920	+
29	92725076	92726013	L1_Mus3	938	-
30	94814902	94815898	L1_Mur1	997	+
31	103134750	103135732	L1Md_F3	983	+
32	104596509	104597427	L1_Mus1	919	+
33	105217675	105218596	L1_Rod	922	-
34	108329295	108330288	L1Md_T	994	+
35	108714694	108715684	L1Md_F2	991	+
36	109220450	109221426	L1Md_F2	977	+
37	111934431	111935363	L1_Mus1	933	-
38	116937075	116938074	L1Md_F2	1000	-
39	128382138	128383119	L1Md_F2	982	+
40	129200254	129201164	L1VL4	911	+
41	130895268	130896255	L1Md_F2	988	+
42	133309184	133310177	L1Md_F2	994	+
43	135160352	135161340	L1Md_F2	989	+
44	146666243	146667169	Lx2B	927	-
45	147211836	147212829	L1Md_T	994	+
46	158899248	158900239	L1Md_F2	992	+
47	162203472	162204414	L1Md_A	943	-
48	162674535	162675531	L1Md_A	997	+
49	165402932	165403928	L1_Mur1	997	-
50	169441997	169442993	L1Md_T	997	+
>1000	Start	End	Subfamily	Length	
1	6281589	6282618	L1_Mur3	1030	+
2	10049944	10051560	L1VL2	1617	+
3	16169701	16171065	Lx6	1365	+
4	16217491	16218633	L1_Mus3	1143	+
5	17426039	17427081	L1Md_F2	1043	-
6	17468326	17470192	L1_Mus2	1867	+
7	20440496	20441935	L1Md_F2	1440	-
8	38722911	38723929	Lx8	1019	+
9	44651815	44653365	Lx2	1551	-
10	45383827	45385363	L1Md_F2	1537	+
11	46477876	46478925	Lx8	1050	+
12	59996438	59997627	L1_Mus4	1190	+
13	64007714	64008768	L1_Mur2	1055	+
14	65311220	65312842	Lx7	1623	-
15	66054185	66056023	L1_Mus2	1839	+
16	68855529	68856829	L1_Mus2	1301	-
17	70180269	70181478	L1_Mur2	1210	+
18	72063736	72064867	Lx7	1132	+
19	79064968	79066170	Lx2	1203	+
20	80020602	80022167	L1Md_F2	1566	-
21	80701207	80702362	Lx3B	1156	+
22	82128125	82129132	L1_Mus3	1008	+
23	82542453	82544258	L1_Mur3	1806	-
24	87450115	87451320	Lx5	1206	+
25	89683583	89684691	L1_Mus3	1109	-
26	90555540	90556826	L1_Mur2	1287	+
27	91943782	91945656	L1Md_F2	1875	-
28	92991181	92992208	L1Md_F2	1028	-
29	93592706	93593775	Lx6	1070	+
30	94573973	94574990	L1Md_A	1018	-
31	94667886	94669007	L1_Mur3	1122	+
32	97539733	97541269	L1Md_T	1537	-
33	103168408	103169530	L1Md_T	1123	+
34	104582695	104584347	Lx9	1653	+
35	105698408	105699759	L1Md_F2	1352	-
36	107018526	107020272	Lx3A	1747	+
37	107143171	107144189	Lx8b	1019	+
38	109275153	109276457	L1_Mus1	1305	-
39	114155429	114157206	L1_Mm	1778	-
40	117088187	117089202	L1Md_F	1016	+
41	118249930	118250975	Lx3B	1046	-
42	119023787	119025097	L1_Mus3	1311	-
43	125352243	125353639	L1Md_F	1397	+
44	128015638	128017000	Lx5c	1363	-
45	129108854	129110116	L1_Mur2	1263	+
46	131802052	131803660	L1Md_F2	1609	-
47	139043713	139045510	L1_Mus2	1798	-
48	142493709	142495448	Lx2B2	1740	-
49	147083506	147085205	L1_Mur2	1700	-
50	155543546	155544577	Lx5b	1032	+
>2000	Start	End	Subfamily	Length	
1	3399554	3401876	L1Md_F2	2323	-
2	8487400	8489768	L1Md_T	2369	-
3	14106917	14109201	Lx5c	2285	-
4	15949630	15951724	L1Md_F2	2095	+
5	18052651	18054871	L1Md_F	2221	+
6	18095463	18097476	L1_Mus3	2014	+
7	22974739	22977036	L1VL2	2298	+
8	26653037	26656024	Lx4A	2988	+
9	26794359	26797340	Lx4A	2982	-
10	26917943	26920630	L1VL2	2688	+
11	26983155	26985898	L1Md_A	2744	+
12	28275891	28278584	L1VL2	2694	+
13	28578004	28580744	L1Md_F2	2741	+
14	33202039	33204850	L1_Mm	2812	+
15	33279682	33281839	L1Md_F	2158	-
16	38925566	38928376	L1Md_F2	2811	-
17	41083522	41085960	Lx2A	2439	-
18	57525508	57528198	L1_Mus2	2691	-
19	58332709	58335340	L1Md_F2	2632	+
20	64633371	64635743	L1_Mus4	2373	+
21	69585306	69587775	Lx2	2470	+
22	76529380	76531809	L1VL4	2430	+
23	77286361	77288816	Lx7	2456	-
24	78761923	78764652	Lx4B	2730	-
25	80202420	80204504	L1_Mus2	2085	-
26	81847962	81850824	L1Md_F	2863	+
27	83137245	83139777	L1_Mur3	2533	-
28	83238632	83241338	L1Md_F2	2707	-
29	86431893	86434679	L1_Mus3	2787	-
30	88778052	88780542	L1_Mus3	2491	+
31	93133434	93135464	L1_Mus3	2031	+
32	93210026	93212646	Lx2B2	2621	+
33	95428715	95430727	L1_Mm	2013	+
34	95501835	95503984	Lx3_Mus	2150	+
35	100066872	100068913	L1_Mur3	2042	-
36	101495360	101498022	L1Md_A	2663	-
37	116365284	116367381	L1_Mur2	2098	-
38	121360045	121362460	L1_Mus1	2416	-
39	121754339	121756776	Lx2B2	2438	-
40	121987812	121990142	Lx3A	2331	+
41	123531624	123535384	L1Md_A	2761	-
42	124161410	124163503	L1Md_Gf	2094	+
43	127625386	127627682	Lx3A	2297	-
44	128348949	128351046	L1_Mus4	2098	+

24	45806978	45807963	L1_Mars1b	986	+
25	46263167	46264159	L1_Mdo3c	993	-
26	47934826	47935813	HAL1N1_MD	988	+
27	50144195	50145095	L1_Mdo4	901	-
28	51868132	51869058	L1_Mdo3b	927	+
29	56549618	56550601	L1_Mdo2	984	+
30	58365583	58366508	L1_Mdo3b	926	+
31	60713427	60714417	L1_Opos0	991	+
32	66551037	66552032	L1_Mars1b	996	-
33	67196225	67197174	L1_Mdo3a	950	+
34	70508982	70509884	L1_Opos	903	+
35	72383733	72384691	L1_Mdo5	959	-
36	74960859	74961852	L1_Mdo3c	994	+
37	78318420	78319374	L1_Opos0	955	+
38	79201152	79202090	L1_Opos2	939	+
39	4885172	4886116	L1_Mdo3a	945	+
40	23015370	23016297	L1_Opos1	928	-
41	24370672	24371653	L1_Opos0	982	-
42	24564368	24565325	L1_Mdo1	958	-
43	37765163	37766089	L1_Opos0	927	+
44	38886584	38887582	L1_Opos1	999	+
45	41658113	41659020	HAL1_Opos1	908	+
46	42874789	42875741	L1_Opos1	953	+
47	52732464	52733400	L1_Mdo3b	937	-
48	59315995	59316931	L1_Mdo5	937	-
49	62847823	62848781	L1_Opos0	959	+
50	69277500	69278420	L1_Mdo3a	921	+
>1000	Start	End	Subfamily	Length	
1	3476548	3478062	L1_Mdo4	1515	+
2	3712482	3714311	L1_Mdo3c	1830	+
3	5026818	5028264	L1_Mdo1	1447	+
4	6172810	6173970	L1_Mdo2	1161	+
5	6932098	6933207	HAL1N1_MD	1110	-
6	10609814	10610864	L1_Mars1	1051	-
7	10800119	10801122	HAL1_Opos	1004	+
8	13010375	13011395	L1_Opos4b	1021	+
9	16375639	16377535	L1_Mdo3c	1897	+
10	19600355	19601737	L1_Opos4b	1383	+
11	22438183	22439465	L1_Mdo1	1283	+
12	22518263	22519778	L1_Mdo3	1516	-
13	22816586	22818001	L1_Opos0	1416	+
14	23970681	23971831	L1_Mdo5	1151	-
15	25832810	25833851	L1_Mdo3	1042	+
16	25869180	25870227	L1_Mdo4	1048	-
17	30315708	30317048	L1_Mdo4	1341	+
18	34904179	34905389	L1_Opos0	1211	+
19	40153523	40154709	L1_Mdo3	1187	-
20	41213069	41214349	L1_Opos1	1281	-
21	43413997	43415405	L1_Mdo2	1409	-
22	44743226	44744643	L1_Opos2	1418	+
23	48668015	48669127	L1_Mdo3c	1113	+
24	50231752	50233490	L1_Mdo6	1739	+
25	51905696	51907063	L1_Opos0	1368	+
26	52579239	52580310	HAL1N1_MD	1072	+
27	54114716	54116611	L1_Mdo3c	1896	+
28	58141133	58142152	L1_Mdo3a	1020	-
29	61063847	61065129	HAL1_Opos	1283	-
30	61162046	61163387	L1_Mdo6	1342	+
31	65459603	65461420	L1_Mdo3c	1818	-
32	67621242	67622528	L1_Opos	1287	-
33	74725168	74726692	L1_Mdo3a	1525	+
34	76007503	76008669	L1_Mdo3c	1167	-
35	4291935	4293652	L1_Mdo3c	1718	+
36	4624094	4625770	L1-1_MD	1677	+
37	6196047	6197810	L1_Mars1	1764	-
38	18151769	18152907	L1_Opos4b	1139	+
39	19188459	19190216	L1_Mdo5	1758	+
40	19849656	19851134	L1_Mdo5	1479	+
41	28869458	28870898	L1_Mdo4	1441	-
42	29184236	29185542	L1_Mdo3c	1307	-
43	36451506	36452652	L1_Opos2	1147	-
44	47684696	47686189	L1_Mdo5	1494	+
45	49490192	49491881	L1_Opos0	1690	-
46	53272746	53273904	L1_Opos2	1159	+
47	54627288	54628617	L1_Opos	1330	-
48	57263016	57264701	HAL1_Opos	1686	-
49	62386251	62388178	L1_Mdo3c	1928	+
50	70803662	70804842	L1_Mdo3c	1181	-
>2000	Start	End	Subfamily	Length	
1	3752921	3755306	L1_Mdo1	2386	+
2	3755303	3757565	L1_Mdo1	2263	-
3	4288080	4290142	L1_Mdo4	2063	+
4	4858145	4860734	L1_Opos0	2590	-
5	4905698	4908152	L1_Mars1	2455	-
6	9222239	9224649	L1_Mdo3c	2411	-
7	9640978	9643050	L1_Opos0	2073	-
8	11433344	11436081	L1_Mdo1	2738	-
9	17345108	17347431	HAL1N1_MD	2324	-
10	17736283	17738904	L1_Mdo3	2622	-
11	20653465	20655819	L1_Mdo3c	2355	-
12	21889430	21891591	L1_Mdo2	2162	-
13	23930772	23933234	L1_Mdo5	2463	+
14	27590071	27592842	L1_Mdo3c	2772	+
15	27705484	27707729	L1_Mdo3a	2246	-
16	28795089	28797835	L1_Mdo2	2747	-
17	31771218	31773671	L1_Mdo5	2454	+
18	32997924	33000000	L1_Mdo3c	2077	+
19	36856075	36858610	L1_Mdo3c	2536	-
20	38127324	38129934	L1_Opos	2611	-
21	39280876	39282933	L1_Mars1	2058	+
22	41531750	41534061	L1_Mdo3c	2312	+
23	44621613	44624014	L1_Mdo5	2402	+
24	49268764	49271636	L1_Mdo1	2873	-
25	51232772	51234908	L1_Mdo3c	2137	-
26	51306301	51308841	HAL1_Opos	2541	-
27	51585474	51588170	L1_Mdo3c	2697	+
28	59805369	59807710	L1_Mdo1	2342	+
29	60011688	60013753	L1_Mdo3b	2066	+
30	60043087	60045919	HAL1_Opos	2833	+
31	61005673	61007710	L1_Mdo2	2038	+
32	61059870	61062046	HAL1N1_MD	2177	-
33	62305574	62307722	HAL1N1_MD	2149	-
34	64217421	64219674	L1_Mdo3a	2254	+
35	64537484	64540290	L1_Mdo2	2807	-
36	68156376	68159202	L1_Mdo2	2827	+
37	69328946	69331051	L1_Mdo2	2106	-
38	41176060	41178148	L1_Mars1b	2089	-
39	72418837	72421540	L1_Mdo2	2704	+
40	72711396	72713407	L1_Opos2	2012	-
41	73508110	73510502	L1_Mdo4	2393	-
42	74921984	74924513	L1_Mdo3c	2530	-
43	75397624	75400553	L1_Mdo2	2930	-
44	76611799	76613975	L1_Mdo4	2177	-

45	121224977	121227106	L1PA13	2130	-
46	138030024	138032668	L1PA16	2645	+
47	141744805	141747042	L1PA3	2238	-
48	142123602	142125846	L1PA5	2245	+
49	143368685	143370907	L1MCa	2223	+
50	143775507	143778497	L1PA16	2991	-
>3000	Start	End	Subfamily	Length	
1	32652142	32655976	L1PA5	3835	-
2	33684597	33688050	L1MA1	3454	-
3	34949626	34953432	L1PREC2	3807	+
4	36613000	36616493	L1PA13	3494	+
5	39077793	39081179	L1PA12	3387	-
6	42222511	42225562	L1MA4A	3052	-
7	43279813	43283566	L1PA15	3754	-
8	43346866	43350213	L1PA17	3348	-
9	45418450	45421461	L1M1	3012	-
10	52216380	52219873	L1MA2	3494	+
11	56970531	56973657	L1PA4	3127	+
12	57953449	57956773	L1PA4	3325	+
13	58015159	58018485	L1PREC2	3327	+
14	58026981	58030256	L1PREC2	3276	-
15	67011408	67015040	L1PA14	3633	+
16	67378210	67381524	L1MA1	3315	+
17	68620694	68624107	L1MA2	3414	-
18	70613122	70616531	L1M1	3410	+
19	73666661	73670366	L1PA11	3706	-
20	74562659	74566096	L1MA2	3438	+
21	74856583	74860185	L1MA3	3603	+
22	75626074	75629246	L1PB1	3173	-
23	75839722	75843294	L1PREC2	3573	-
24	76204026	76207323	L1MA1	3298	-
25	78176143	78179165	L1P1	3023	+
26	78774282	78777376	L1PB1	3095	-
27	78860857	78863872	L1M1	3016	+
28	81046041	81049078	L1PB4	3038	-
29	81723802	81727426	L1PA14	3625	+
30	82683994	82687197	L1PA10	3204	-
31	84450114	84453142	L1M2	3029	+
32	84453136	84457131	L1MA3	3996	+
33	85805503	85809069	L1PREC2	3567	-
34	88317181	88320770	L1PA5	3590	-
35	91955392	91958786	L1PA11	3395	-
36	92033105	92036303	L1MA2	3199	-
37	96656453	96660383	L1PA13	3931	+
38	108098364	108101571	L1MD2	3208	-
39	114086742	114090623	L1MA3	3882	+
40	116131429	116134490	L1MA2	3062	-
41	121277193	121281180	L1MA2	3988	+
42	122001194	122004781	L1PA10	3588	-
43	127480314	127483521	L1MA2	3208	-
44	127486733	127490437	L1M1	3705	-
45	129419635	129423210	L1PA7	3576	-
46	142949474	142953261	L1PA11	3788	-
47	142953257	142957052	L1PA7	3796	-
48	143960673	143963877	L1PA13	3205	-
49	144594425	144598051	L1MA2	3627	-
50	155430461	155433680	L1PA5	3220	+
>4000	Start	End	Subfamily	Length	
1	21218621	21222739	L1PA5	4119	-
2	21533799	21538523	L1MA5	4725	-
3	26891800	26896290	L1M2	4491	+
4	29647366	29651911	L1MA8	4546	+
5	34597237	34601391	L1PREC2	4155	-
6	36120307	36124854	L1MA2	4548	-
7	42344803	42349416	L1MEf	4614	-
8	42407689	42412413	L1PA11	4725	-
9	48869850	48874138	L1PA4	4289	+
10	50540548	50545382	L1MCa	4835	+
11	51576975	51581780	L1PA8A	4806	+
12	54697499	54702045	L1P1	4547	+
13	54731465	54735789	L1PA4	4325	-
14	55416116	55420875	L1PA10	4760	+
15	55627238	55631768	L1PA6	4531	+
16	56190046	56194072	L1MA3	4027	+
17	56388485	56392677	L1PA4	4193	-
18	57242459	57246636	L1P3	4178	+
19	57559280	57563525	L1PA4	4246	+
20	57891568	57895623	L1PB4	4056	+
21	65401005	65405337	L1MA2	4333	+
22	66347285	66352091	L1PA3	4807	+
23	67815861	67820237	L1MA2	4377	+
24	69003952	69008752	L1MC3	4801	-
25	73231545	73235732	L1PA6	4188	+
26	73542581	73546602	L1PA7	4022	+
27	75720032	75725001	L1PA7	4970	-
28	78383265	78387481	L1MA2	4217	+
29	78502263	78506919	L1M1	4657	+
30	78506966	78511879	L1PB1	4914	+
31	78714313	78718779	L1PA3	4467	+
32	83839860	83844072	L1PA2	4213	+
33	88309839	88314507	L1PA8	4669	+
34	90633994	90638875	L1PA16	4882	+
35	91649681	91653778	L1MA3	4098	-
36	96254213	96258374	L1PA5	4162	-
37	98051128	98055316	L1MA2	4189	-
38	101587664	101592186	L1PA13	4523	-
39	109314879	109319740	L1PA7	4862	-
40	113319741	113323839	L1MB1	4099	+
41	113363058	113367412	L1PA8A	4355	-
42	115962698	115966769	L1PB1	4072	+
43	122260249	122264960	L1MA1	4712	-
44	125823090	125827147	L1PA16	4058	-
45	125874482	125878978	L1MA2	4497	-
46	128378368	128382528	L1MA7	4161	+
47	129206477	129211272	L1PB1	4796	-
48	139158581	139163556	L1PA7	4976	+
49	141799232	141803971	L1PA4	4740	-
50	147869208	147873823	L1MA2	4616	+
>5000	Start	End	Subfamily	Length	
1	6933211	6938641	L1PA2	5431	+
2	13724626	13729626	L1PA6	5001	+
3	14683910	14688939	L1PA5	5030	-
4	22000000	22005591	L1P2	5592	-
5	25408765	25414189	L1ME4a	5425	-
6	42435646	42440874	L1PA15	5229	+
7	53888926	53894539	L1PA4	5614	+
8	56075957	56081346	L1PA3	5390	-
9	56370879	56376277	L1PA3	5399	+
10	56464081	56469736	L1PBa	5656	-
11	57609837	57614905	L1PA4	5069	-
12	65760413	65765752	L1PA7	5340	-
13	67830589	67835749	L1PA7	5161	+
14	74712706	74718083	L1PA3	5378	+

45	129226487	129228741	L1VL2	2255	-
46	131304390	131307327	L1VL4	2938	-
47	154531497	154533506	L1Md_F2	2010	-
48	156493473	156495678	L1_Mus3	2206	+
49	160665416	160667846	L1_Mur2	2431	+
50	165421577	165423975	L1_Mus2	2399	-
>3000	Start	End	Subfamily	Length	
1	5153950	5157042	L1_Mus3	3093	-
2	5518592	5521766	L1_Mus2	3175	+
3	14246775	14250009	L1_Mus1	3235	+
4	14417261	14420951	L1Md_F2	3691	+
5	15406399	15409885	L1_Mus2	3487	-
6	19922639	19925754	L1Md_F	3116	-
7	27985326	27988441	L1_Mm	3116	-
8	28159063	28162166	L1Md_F2	3104	-
9	28646419	28649534	L1_Mm	3116	-
10	30664383	30667713	L1_Mus3	3331	+
11	33662503	33665593	L1_Mus3	3091	-
12	35652829	35656048	L1VL1	3220	-
13	35829476	35832632	Lx2B2	3157	+
14	39366012	39369950	L1Md_A	3939	+
15	40826594	40830488	L1_Mus2	3895	+
16	45743467	45746954	L1Md_F2	3488	+
17	50707372	50710393	L1VL2	3022	-
18	56410299	56413527	L1Md_T	3229	+
19	61983753	61986780	L1Md_F2	3028	+
20	65010742	65013908	L1_Mus2	3167	+
21	65676189	65680144	L1_Mus3	3956	+
22	67324544	67328151	Lx3_Mus	3608	+
23	68161426	68165244	L1Md_F2	3819	+
24	79102025	79105066	L1Md_F2	3042	+
25	83340737	83344273	L1Md_F2	3537	-
26	91582504	91586254	L1_Mur1	3751	-
27	92735883	92739141	Lx4A	3259	+
28	106731101	106735019	L1Md_T	3919	+
29	107213897	107217295	L1Md_F2	3399	-
30	108680507	108683626	L1Md_F	3120	+
31	109170394	109174203	L1Md_F2	3810	-
32	109710255	109713865	L1_Mm	3611	+
33	110840770	110844067	L1_Mus2	3298	-
34	111478938	111482486	L1Md_T	3549	+
35	116523976	116527817	L1Md_F2	3842	+
36	116814387	116818365	L1_Mur2	3979	+
37	116930444	116934150	L1Md_F2	3707	-
38	118524127	118528024	L1Md_F2	3898	+
39	119846146	119849813	L1Md_F2	3668	-
40	120088478	120091889	L1_Mus3	3412	-
41	126795992	126799090	L1_Mus1	3099	-
42	126835066	126838784	L1_Mus2	3719	-
43	126838786	126841816	L1_Mus1	3031	-
44	127567813	127570893	L1Md_F2	3081	+
45	129026527	129030117	L1Md_F	3591	-
46	132593549	132596708	Lx7	3160	+
47	136472418	136476066	L1Md_F2	3649	-
48	145830869	145834266	Lx5	3398	-
49	147315590	147318764	L1_Mus3	3175	+
50	147734443	147738281	L1VL1	3839	+
>4000	Start	End	Subfamily	Length	
1	3759824	3764392	L1_Mus1	4569	-
2	9822562	9826737	L1_Mus1	4176	-
3	17371110	17375328	L1VL2	4219	+
4	18132861	18137092	L1_Mus3	4232	+
5	26848044	26852969	L1Md_F	4926	-
6	28715814	28719842	L1Md_F2	4029	+
7	31748364	31752682	L1_Mus1	4319	-
8	33645677	33650220	L1_Mus1	4544	+
9	39880480	39884839	L1_Mus1	4360	-
10	47410746	47415549	L1Md_F2	4804	+
11	55514144	55518722	L1Md_F	4579	-
12	61012929	61017641	L1_Mus3	4713	-
13	61215125	61219821	L1Md_F2	4697	-
14	61309101	61313152	L1_Mus2	4052	-
15	61493724	61498715	L1Md_F2	4992	+
16	64759078	64763349	L1Md_T	4272	-
17	68427086	68431832	L1Md_A	4747	+
18	76742045	76746327	L1Md_F2	4283	-
19	78004123	78008866	L1Md_F	4744	+
20	78814226	78818469	L1Md_F2	4244	+
21	79949324	79953963	L1Md_F3	4640	+
22	80309501	80313880	L1_Mus4	4380	+
23	80401940	80405982	Lx4B	4043	-
24	81567153	81571953	L1Md_T	4801	-
25	82336776	82341376	L1Md_F2	4601	-
26	88346785	88351468	L1Md_A	4684	+
27	89062803	89067015	L1Md_F2	4213	-
28	90627662	90632639	L1_Mus2	4978	-
29	90705495	90709647	L1Md_T	4153	+
30	107516126	107521048	L1Md_T	4923	+
31	108167862	108171908	Lx5c	4047	+
32	109347632	109352559	L1VL1	4928	-
33	109452315	109457160	L1Md_F	4846	-
34	110133885	110137967	L1Md_F	4083	+
35	112784355	112788867	L1_Mus3	4513	+
36	114178612	114183108	L1Md_F2	4497	+
37	115116339	115120934	L1Md_F3	4596	-
38	117862030	117866657	L1Md_F2	4628	+
39	120832050	120836235	L1Md_A	4186	+
40	121168574	121172749	L1_Mus3	4176	+
41	121833849	121838463	L1Md_F2	4615	-
42	123874455	123878947	Lx5b	4493	+
43	124422119	124426607	Lx5b	4489	-
44	124432991	124437603	L1Md_T	4613	-
45	129320788	129325246	L1Md_F	4459	-
46	132745201	132750023	L1Md_T	4823	+
47	147206435	147211117	L1Md_Gf	4683	+
48	150698232	150702633	L1Md_T	4402	-
49	152838652	152843253	L1Md_F2	4602	-
50	156003209	156007432	L1VL4	4224	-
>5000	Start	End	Subfamily	Length	
1	9706136	9711204	L1Md_A	5069	-
2	14961333	14966553	L1Md_A	5221	+
3	16305824	16311143	L1Md_F2	5320	+
4	16439998	16445780	L1Md_A	5783	+
5	16868878	16873888	L1Md_A	5011	+
6	18830149	18836015	L1Md_F2	5867	-
7	18898972	18904064	L1Md_T	5093	+
8	23864787	23870029	L1Md_F2	5243	-
9	26546450	26552346	L1_Mus1	5897	-
10	28683918	28689397	L1Md_F2	5480	-
11	34016378	34021679	L1Md_F2	5302	+
12	35015778	35021043	L1_Mus3	5266	+
13	41094413	41099639	L1Md_A	5227	+
14	41231748	41237508	L1Md_T	5761	+

45	76802260	76805058	L1_Mdo4	2799	-
46	34909784	34911820	L1_Mdo3	2037	-
47	38750081	38752389	L1_Mdo1	2309	+
48	49262288	49264596	L1_Mdo3c	2309	-
49	53585723	53587810	L1_Mdo1	2088	-
50	67587822	67590244	L1_Mdo3b	2423	-
>3000	Start	End	Subfamily	Length	
1	4430126	4433536	HAL1_Opos	3411	-
2	12172376	12175402	L1_Mdo3b	3027	+
3	12760692	12763894	L1_Mdo1	3203	+
4	15259973	15263360	L1_Mdo3c	3388	+
5	16256316	16259695	L1_Mdo2	3380	-
6	18850346	18854194	L1_Opos	3849	+
7	19168728	19172077	L1_Opos2	3350	+
8	19239063	19242949	L1_Opos0	3887	-
9	21205781	21209760	L1_Mdo3c	3980	-
10	21443264	21446942	L1_Mdo4	3679	+
11	21686307	21689968	L1_Mdo4	3662	+
12	22596231	22599423	L1_Mdo4	3193	+
13	23130765	23133956	L1_Mdo3c	3192	+
14	23174631	23178490	L1-1_MD	3860	-
15	23251006	23254025	L1_Mdo3c	3020	-
16	26301753	26304826	L1_Mdo3a	3074	-
17	27561429	27564875	L1_Mdo3c	3447	+
18	27571519	27574625	L1_Mdo3c	3107	+
19	29657454	29661018	L1_Mdo3c	3565	+
20	30181401	30185246	L1_Opos1	3846	+
21	30393136	30396962	L1_Mdo4	3827	-
22	30719139	30722635	L1_Mdo3c	3497	+
23	30936565	30940169	L1_Mdo3a	3605	+
24	32600127	32603629	L1_Mdo3c	3503	+
25	33635189	33638366	L1_Mdo3c	3178	+
26	34436425	34439504	L1_Mdo4	3080	-
27	37844232	37847483	L1_Mdo6	3252	-
28	41712115	41715613	HAL1N1_MD	3499	+
29	47824826	47828292	L1_Mdo3a	3467	-
30	48502509	48506262	L1_Mdo5	3754	-
31	49584411	49588206	L1_Mdo3c	3796	+
32	51483528	51487057	L1_Opos	3530	-
33	51740613	51743628	L1_Mdo1	3016	+
34	51788196	51791487	L1_Opos0	3292	-
35	54684787	54688140	L1_Mdo2	3354	+
36	56624852	56628561	L1-1_MD	3710	-
37	60427304	60431057	L1_Mdo4	3754	-
38	60749533	60753246	L1_Mdo4	3714	-
39	61232048	61235747	L1_Mdo1	3700	-
40	62538625	62541710	L1_Mdo3	3086	+
41	66000268	66003595	HAL1_Opos1	3328	-
42	70258804	70262127	L1_Opos0	3324	-
43	72422046	72425470	L1_Mdo2	3425	+
44	72893129	72896153	L1_Mdo3c	3025	-
45	73967803	73971009	L1_Opos1	3207	-
46	74917599	74921379	L1_Mdo3c	3781	-
47	76294366	76298248	L1_Opos1	3883	-
48	76630758	76634175	L1_Mdo3a	3418	+
49	8914044	8917321	L1_Mdo3c	3278	-
50	18703290	18706968	L1-1_MD	3679	+
>4000	Start	End	Subfamily	Length	
1	5256875	5260981	L1_Mdo3a	4107	-
2	5368632	5372640	L1_Mdo1	4009	-
3	6136250	6141115	L1_Mdo3c	4866	-
4	6779708	6784273	L1-1_MD	4566	+
5	8379721	8383773	L1_Mdo1	4053	-
6	8874166	8878990	L1_Mdo3c	4825	+
7	8985283	8989863	L1_Mdo3c	4581	+
8	11436081	11440801	L1-1_MD	4721	+
9	13519987	13524411	L1_Mdo4	4425	+
10	15534137	15538348	L1_Mdo3c	4212	-
11	18619644	18623678	L1_Mdo4	4035	+
12	20505249	20509582	L1_Mdo3c	4334	+
13	20881620	20886381	L1_Mdo3c	4762	-
14	21491822	21496756	L1_Mdo3	4935	+
15	24079633	24084245	L1_Mdo3c	4613	-
16	27612775	27617654	L1_Mdo2	4880	+
17	27710003	27714843	L1_Mdo2	4841	-
18	28978976	28983460	L1_Mdo2	4485	+
19	29489799	29494035	L1_Mdo3a	4237	-
20	29892091	29896674	L1_Mdo3	4584	-
21	30350384	30354856	L1_Opos0	4473	+
22	33435571	33439737	L1_Mdo3c	4167	-
23	36374752	36379043	L1_Opos2	4292	-
24	38487767	38492643	L1_Mdo1	4877	-
25	39824898	39829621	L1_Mdo4	4724	+
26	47615529	47619942	L1_Mdo2	4414	-
27	47705674	47710131	L1_Mdo4	4458	-
28	47759414	47764328	L1_Mdo4	4915	+
29	48073034	48077215	L1_Mdo3b	4182	-
30	48401657	48406325	L1_Mdo5	4669	-
31	48785798	48790387	L1_Mdo2	4590	-
32	48963883	48968029	L1_Mdo3c	4147	+
33	49102778	49107132	L1_Mdo3b	4355	-
34	51600434	51605362	L1_Mdo3a	4929	+
35	51726099	51730722	L1_Mdo3a	4624	+
36	51855496	51860213	L1_Mdo3a	4718	+
37	57428776	57432983	L1_Mdo3	4208	-
38	58008132	58012911	L1_Mdo1	4780	-
39	62806930	62811454	L1_Mdo3c	4525	+
40	68513691	68518534	L1_Mdo1	4844	+
41	69385597	69389964	L1_Opos0	4368	-
42	71488085	71492574	L1_Mdo3a	4490	-
43	71493169	71497796	L1_Mdo3c	4628	-
44	72009936	72014897	L1_Mdo3c	4962	+
45	72425470	72429471	L1_Mdo3b	4002	+
46	73927520	73932331	L1_Mdo3c	4812	+
47	74552786	74557077	L1_Mdo4	4292	+
48	76568169	76572920	L1_Mdo3c	4752	-
49	6639639	6643995	L1_Mdo3a	4357	+
50	49597634	49602007	L1-1_MD	4374	+
>5000	Start	End	Subfamily	Length	
1	3834876	3840256	L1-1_MD	5381	+
2	4168127	4173682	L1_Mdo1	5556	-
3	5082160	5087638	L1_Mdo4	5479	-
4	5378573	5384273	L1_Mdo4	5701	+
5	6159980	6165386	L1-1_MD	5407	+
6	6562266	6568217	L1_Mdo3c	5952	+
7	6629944	6635623	L1_Mdo4	5680	+
8	6656985	6662919	L1_Mdo1	5935	+
9	8549567	8555376	L1_Mdo4	5810	-
10	11979934	11985241	L1_Mdo3b	5308	-
11	12238639	12244146	L1_Mdo1	5508	+
12	13297891	13303438	L1_Mdo3a	5548	+
13	14342906	14347981	L1_Mdo1	5076	-
14	15007342	15012411	L1_Mdo3c	5070	-

15	75088141	75093766	L1MC2	5626	+
16	76013973	76019797	L1PB1	5825	+
17	76597101	76602933	L1PA3	5833	-
18	78152445	78158029	L1PA4	5585	-
19	79008286	79013878	L1PA7	5593	-
20	81332985	81338702	L1PREC2	5718	-
21	81792122	81797219	L1PA4	5098	+
22	81881852	81887735	L1PB1	5884	-
23	82060753	82066238	L1PREC2	5486	+
24	82253941	82259316	L1PA15	5376	-
25	83232584	83238554	L1PB1	5971	+
26	86165197	86170705	L1MA1	5509	-
27	87356334	87362103	L1MA1	5770	+
28	88630836	88635913	L1MA2	5078	+
29	90002189	90007499	L1PA17	5311	-
30	91384375	91389710	L1MA3	5336	+
31	92715936	92721671	L1PA6	5736	+
32	99042497	99047517	L1MC1	5021	-
33	99202343	99207567	L1MA3	5225	-
34	99266352	99272048	L1PB1	5697	-
35	99771821	99777666	L1MA1	5846	-
36	100090504	100095545	L1PA14	5042	+
37	100304047	100309154	L1PB1	5108	+
38	106358705	106364205	L1PA13	5501	-
39	109603801	109609551	L1PA7	5751	+
40	113446054	113451461	L1PA3	5408	+
41	115931093	115936482	L1MA1	5390	-
42	117589279	117594775	L1PA10	5497	+
43	121551321	121557215	L1MA1	5895	+
44	125500000	125505873	L1M1	5874	-
45	125629048	125634462	L1PB3	5415	+
46	126770794	126776707	L1PB1	5914	+
47	139007865	139013479	L1PB1	5615	+
48	141044310	141050237	L1PA5	5928	+
49	141448494	141454070	L1PA5	5577	+
50	146456450	146461601	L1PA16	5152	+

>6000	Start	End	Subfamily	Length	
1	19940253	19946296	L1PA2	6044	-
2	20874271	20880729	L1PA10	6459	+
3	22866381	22872850	L1PA7	6470	+
4	26314417	26320446	L1HS	6030	-
5	34977424	34983563	L1PA13	6140	+
6	37768611	37774761	L1PA4	6151	+
7	42004299	42010329	L1PA3	6031	+
8	51644758	51650770	L1PA3	6013	+
9	52291785	52297932	L1PA4	6148	+
10	55046934	55052940	L1PA4	6007	-
11	55668332	55674513	L1PA7	6182	-
12	55681153	55687183	L1PA3	6031	-
13	56047265	56053280	L1PA4	6016	-
14	56183880	56190030	L1PA3	6151	+
15	56581519	56587672	L1PA3	6154	-
16	56695884	56701916	L1HS	6033	+
17	57094341	57100758	L1PA7	6418	+
18	62517103	62523125	L1PA3	6023	+
19	63286004	63292073	L1PA4	6070	-
20	63519156	63525277	L1PA4	6122	+
21	63838642	63844790	L1PA3	6149	+
22	66718300	66724758	L1PA8	6459	+
23	67659222	67665252	L1PA4	6031	-
24	68264543	68270753	L1PA5	6211	+
25	69350757	69356897	L1PA5	6141	+
26	70728178	70734314	L1PA4	6137	+
27	73093936	73100083	L1PA4	6148	-
28	73476191	73482637	L1PA7	6447	-
29	73492039	73498087	L1PA7	6049	-
30	77244267	77250292	L1PA3	6026	+
31	80650595	80656722	L1PA5	6128	+
32	81469490	81475588	L1PA6	6099	-
33	83407135	83413200	L1PA5	6066	+
34	85555430	85561869	L1PA8A	6440	-
35	85583161	85589299	L1PA5	6139	+
36	88207885	88213919	L1PA2	6035	+
37	95612970	95619412	L1PA7	6443	-
38	96597640	96603783	L1PA5	6144	+
39	104889804	104895934	L1PA5	6131	-
40	105959686	105965737	L1PA4	6052	+
41	110640008	110646163	L1PA4	6156	-
42	114720516	114726531	L1PA2	6016	-
43	116159201	116165340	L1PA3	6140	+
44	119435468	119441493	L1HS	6026	-
45	128041692	128048556	L1PA16	6865	-
46	131524490	131530619	L1PA5	6130	+
47	132284468	132290617	L1PA5	6150	-
48	132445801	132452124	L1PA8	6324	-
49	141421202	141427246	L1HS	6045	-
50	147200309	147206474	L1PA6	6166	+

>7000	Start	End	Subfamily	Length	
1	23524128	23531723	L1MA2	7596	+
2	36887118	36894338	L1MA2	7221	+
3	37319423	37327019	L1MA2	7597	+
4	42336457	42343834	L1PA7	7378	-
5	42920470	42928181	L1MA3	7712	+
6	45354126	45361574	L1MA2	7449	-
7	45370650	45378338	L1MA3	7689	+
8	51367789	51375287	L1MA1	7499	+
9	57313485	57320856	L1PA7	7372	+
10	64457591	64465678	L1PA13	8088	-
11	75397600	75405393	L1PA14	7794	+
12	79225838	79233079	L1MA2	7242	-
13	80300222	80307242	L1MA1	7021	-
14	81083643	81090986	L1PA8	7344	+
15	82096260	82103835	L1MA1	7576	-
16	82213138	82220837	L1PA13	7700	-
17	83648724	83655748	L1PA3	7025	-
18	84309533	84316657	L1MA1	7125	+
19	85231360	85238868	L1MA3	7509	+
20	86639056	86646066	L1PA14	7011	+
21	89492298	89499883	L1MA1	7586	-
22	93467452	93474683	L1PA16	7232	+
23	94607182	94614339	L1MA1	7158	-
24	95238867	95246445	L1MA1	7579	+
25	98099897	98107265	L1MA2	7369	-
26	99273191	99280734	L1MA2	7544	+
27	102274646	102281803	L1MA1	7158	-
28	102405312	102412468	L1MA1	7157	+
29	102618923	102626033	L1PREC2	7111	-
30	106682450	106690042	L1MA3	7593	+
31	109096389	109104025	L1MA1	7637	-
32	109257834	109265406	L1MA2	7573	-
33	113385401	113392971	L1PA15	7571	-
34	116799760	116807095	L1MA2	7336	-
35	117694786	117702345	L1MA1	7560	+

15	44073069	44078116	L1Md_F3	5048	-
16	49825215	49830506	Lx	5292	+
17	50211682	50217173	L1Md_A	5492	+
18	50314692	50320449	Lx	5758	+
19	58288442	58293683	L1Md_T	5242	+
20	61886757	61891865	L1_Mus2	5109	+
21	64971594	64977584	Lx2	5991	+
22	65539940	65545760	L1Md_A	5821	+
23	72143801	72149052	L1Md_T	5252	+
24	77027482	77033108	L1Md_F2	5627	+
25	77242384	77247592	L1Md_A	5209	+
26	79819564	79825238	L1Md_T	5675	+
27	79921596	79926782	L1Md_A	5187	+
28	80488710	80493919	L1Md_F2	5210	+
29	84808145	84813324	L1Md_T	5180	+
30	85683817	85689454	L1_Mus3	5638	+
31	86910452	86915936	L1Md_F2	5485	-
32	90077996	90083015	L1_Mus2	5020	+
33	90168786	90174010	L1Md_T	5225	+
34	91489244	91494455	L1Md_F2	5212	-
35	92712243	92717621	L1Md_T	5379	+
36	108370500	108375784	L1Md_F2	5285	+
37	113351889	113357273	L1Md_T	5385	+
38	117138888	117143908	L1Md_F2	5021	-
39	117690949	117696189	L1Md_F2	5241	+
40	127665420	127671411	L1Md_F2	5992	-
41	128535979	128541494	Lx3_Mus	5516	+
42	132070214	132075742	L1_Mus1	5529	+
43	132562520	132567932	L1Md_A	5413	+
44	145780345	145785491	L1Md_A	5147	+
45	148730630	148735839	L1Md_Gf	5210	+
46	151708566	151714371	Lx3C	5806	-
47	154110907	154115992	L1Md_F2	5086	+
48	154160919	154166643	L1_Mus1	5725	-
49	156743562	156749120	L1Md_F2	5559	+
50	160569111	160574148	L1Md_F2	5038	-

>6000	Start	End	Subfamily	Length	
1	4889376	4895777	L1Md_T	6402	-
2	6503937	6510252	L1_Mus1	6316	-
3	9503133	9509558	L1Md_A	6426	-
4	9889792	9896202	L1Md_A	6411	-
5	22500320	22507312	L1Md_A	6993	-
6	25022161	25029118	L1_Mus1	6958	-
7	25324510	25331481	L1_Mus1	6972	+
8	25794923	25801653	L1_Mus1	6731	+
9	26807186	26813465	L1_Mus1	6280	-
10	26958687	26965592	L1_Mus1	6906	+
11	30113132	30119916	L1Md_A	6785	-
12	30941076	30947218	L1Md_F2	6143	-
13	31989527	31995814	L1Md_F2	6288	-
14	32120048	32126380	L1Md_F2	6333	-
15	33869116	33875587	L1Md_F2	6472	-
16	45481752	45488186	L1Md_F2	6435	-
17	45572714	45578812	L1Md_F2	6099	-
18	47145091	47151279	L1_Mus4	6189	+
19	50098195	50104601	L1Md_F2	6407	-
20	53802653	53808995	L1Md_A	6343	-
21	58795284	58801594	L1Md_T	6311	-
22	59460147	59466549	L1Md_T	6403	-
23	63925960	63932152	L1Md_A	6193	-
24	66123163	66129428	L1Md_F2	6266	-
25	67575035	67581182	L1Md_F2	6148	-
26	68607348	68613554	L1Md_F2	6207	-
27	72577989	72584052	L1Md_T	6064	+
28	76037930	76044341	L1Md_F3	6412	-
29	76253228	76259356	L1Md_F2	6129	-
30	76701434	76707521	L1Md_T	6088	-
31	79197725	79203914	L1Md_F2	6190	-
32	79610302	79616498	L1Md_A	6197	-
33	80365785	80372191	L1Md_A	6407	-
34	80502967	80509359	L1Md_A	6393	-
35	85505183	85511572	L1Md_T	6390	-
36	89892921	89899251	L1Md_F2	6331	+
37	90984726	90990976	L1_Mus1	6251	-
38	101823081	101829759	Lx	6679	-
39	109369028	109375220	L1Md_T	6193	-
40	109824909	109831455	L1Md_A	6547	-
41	114703745	114709995	L1Md_T	6251	-
42	118973270	118979459	L1Md_F2	6190	-
43	119291832	119297924	L1Md_F2	6093	-
44	119434117	119440177	L1Md_A	6061	-
45	121367332	121373539	L1Md_T	6208	-
46	121949583	121956235	L1Md_T	6653	-
47	128946718	128953296	L1Md_F2	6579	-
48	133428382	133434962	L1Md_T	6581	-
49	135631627	135638203	L1Md_A	6577	-
50	150861629	150867869	L1Md_T	6241	-

>7000	Start	End	Subfamily	Length	
1	14570277	14577440	L1Md_T	7164	+
2	15393326	15400853	L1Md_T	7528	-
3	18715565	18722765	L1Md_A	7201	-
4	21238942	21246066	L1Md_T	7125	-
5	21254673	21261764	L1Md_T	7092	-
6	25590630	25597659	L1_Mus1	7030	+
7	30241624	30249231	L1Md_A	7608	-
8	35316109	35323716	L1Md_A	7608	-
9	35501873	35508901	L1Md_T	7029	-
10	40304675	40311979	L1Md_T	7305	-
11	43673157	43680650	L1Md_T	7494	-
12	46594160	46601433	Lx	7274	+
13	61251843	61258918	L1Md_T	7076	-
14	61259207	61266477	L1Md_T	7271	-
15	61962509	61969624	L1Md_T	7116	-
16	64626076	64633369	L1Md_T	7294	-
17	67373450	67380537	L1Md_T	7088	-
18	68444052	68451196	L1Md_F3	7145	-
19	69930907	69938522	L1Md_T	7616	-
20	73268095	73275199	L1Md_T	7105	-
21	73300016	73307715	L1Md_T	7700	-
22	75194300	75201424	L1Md_T	7125	-
23	79737865	79744900	L1Md_T	7036	-
24	79855090	79862171	L1Md_T	7082	-
25	80596221	80603239	L1Md_A	7019	+
26	80823429	80830519	L1Md_F2	7091	-
27	80959138	80966825	L1Md_T	7688	-
28	84153347	84160558	L1Md_T	7212	-
29	86892148	86899464	L1Md_T	7317	-
30	91835105	91844478	L1Md_T	9374	-
31	97960209	97967812	L1Md_T	7604	-
32	104871693	104878924	L1Md_T	7232	+
33	114260688	114268026	L1Md_T	7339	-
34	119502566	119509615	L1Md_T	7050	-
35	121733063	121740539	L1Md_T	7477	-

15	16931051	16936260	L1_Mdo1	5210	-
16	18937000	18942306	L1_Mdo3c	5307	+
17	19344031	19349797	L1-1_MD	5767	+
18	20049079	20054131	L1_Mdo4	5053	-
19	20398266	20403970	L1_Mdo3c	5705	-
20	22138476	22143707	L1_Mdo1	5232	-
21	23681100	23687059	L1_Mdo4	5960	+
22	23697169	23702486	L1_Mdo2	5318	-
23	23963488	23969202	L1_Mdo4	5715	+
24	24472868	24478622	L1-1_MD	5755	+
25	24688441	24693585	L1_Mdo4	5145	-
26	27138486	27143861	L1_Mdo2	5376	+
27	29746186	29751724	L1_Mdo5	5539	-
28	29908952	29914219	L1_Mdo4	5268	-
29	31113477	31119456	L1-1_MD	5980	+
30	33098149	33103985	L1_Mdo3c	5837	-
31	34923947	34929151	L1_Mdo4	5205	-
32	39338181	39343299	L1_Mdo3	5119	+
33	44355248	44360775	L1_Mdo4	5528	-
34	44360904	44366773	L1_Mdo3c	5870	+
35	45285762	45291235	L1_Opos0	5474	+
36	46210934	46216433	L1_Mdo3a	5500	+
37	46656212	46661289	L1_Mdo3c	5078	-
38	49391838	49397008	L1_Mdo2	5171	+
39	50942096	50947753	L1_Mdo3c	5658	-
40	56192894	56198386	L1_Mdo3	5493	-
41	57365683	57371658	L1_Mdo1	5976	-
42	58030735	58036184	L1_Mdo3c	5450	-
43	58892345	58897814	L1_Mdo3a	5470	-
44	64204586	64209689	L1_Mdo3a	5104	+
45	66405432	66411064	L1-1_MD	5633	-
46	73903012	73908071	L1_Mdo1	5060	-
47	74098538	74104195	L1_Mdo3b	5658	-
48	74490795	74496757	L1-1_MD	5963	-
49	76484454	76490082	L1_Mdo3b	5629	-
50	76792474	76798374	L1_Mdo3b	5901	-

>6000	Start	End	Subfamily	Length	
1	4246531	4253037	L1_Mdo1	6507	+
2	8568021	8574718	L1_Mdo1	6698	-
3	8729589	8735894	L1-1_MD	6306	-
4	10835545	10841960	L1_Mdo2	6416	-
5	12524955	12531257	L1_Mdo3c	6303	-
6	12770891	12777490	L1_Mdo2	6600	+
7	13048141	13054494	L1-1_MD	6354	+
8	13158811	13165503	L1_Mdo4	6693	-
9	16030013	16036691	L1_Mdo3c	6679	-
10	18179790	18186144	L1-1_MD	6355	-
11	18423045	18429614	L1_Mdo3c	6570	-
12	20034325	20041058	L1_Mdo1	6734	-
13	20866797	20873153	L1_Mdo3c	6357	-
14	22264881	22271148	L1-1_MD	6268	-
15	24423513	24429684	L1_Mdo4	6172	+
16	24442474	24448597	L1_Mdo2	6124	+
17	27411150	27417785	L1_Mdo3c	6636	-
18	28065294	28071939	L1_Mdo3c	6646	-
19	30403346	30409512	L1_Mdo2	6167	-
20	36601420	36607927	L1_Mdo2	6508	-
21	36722229	36728400	L1-1_MD	6172	+
22	38843335	38849356	L1_Mdo3c	6022	+
23	40768202	40774884	L1_Mdo1	6683	+
24	41799898	41805964	L1_Mdo2	6067	+
25	42828251	42835021	L1_Mdo2	6771	-
26	42867599	42874202	L1_Mdo5	6604	-
27	45417089	45423836	L1_Mdo4	6748	+
28	46104709	46111317	L1_Mdo3c	6609	+
29	48257084	48263405	L1-1_MD	6322	-
30	48635926	48642157	L1-1_MD	6232	-
31	49760389	49766542	L1-1_MD	6154	+
32	50208678	50215402	L1_Mdo1	6725	-
33	52519013	52525728	L1_Mdo4	6716	-
34	53587812	53594330	L1_Mdo1	6519	-
35	59684739	59690959	L1_Mdo2	6221	-
36	60148260	60154613	L1-1_MD	6354	+
37	60285425	60291782	L1-1_MD	6358	+
38	60409867	60416109	L1-1_MD	6243	+
39	61028939	61035295	L1-1_MD	6357	-
40	61079446	61086006	L1-1_MD	6561	-
41	61359627	61366030	L1-1_MD	6404	+
42	62489703	62496137	L1_Mdo1	6435	+
43	62966489	62972811	L1-1_MD	6323	-
44	66196069	66202187	L1_Mdo1	6119	+
45	66220228	66226501	L1_Mdo2	6274	-
46	66830805	66837192	L1-1_MD	6388	-
47	71153832	71160192	L1_Mdo4	6361	-
48	73958777	73965357	L1-1_MD	6581	-
49	76615268	76621763	L1_Mdo1	6496	+
50	78466139	78472590	L1_Mdo4	6452	-

>7000	Start	End	Subfamily	Length	
1	15153087	15160107	L1_Mdo2	7021	-
2	54771906	54778938	L1_Mdo2	7033	+
3	61389257	61396314	L1_Mdo2	7058	-
4	62837253	62844510	HAL1_Opos	7258	-

36	122357875	122365678	L1PA13	7804	-
37	126004918	126013083	L1PREC2	8166	+
38	128772433	128780093	L1MA2	7661	-
39	129172372	129180057	L1MA2	7686	-
40	131108095	131115774	L1MA2	7680	-
41	139348446	139356047	L1MA2	7602	-
42	140277331	140284445	L1MA2	7115	-
43	141924916	141932495	L1MA2	7580	-
44	145388045	145395239	L1MA3	7195	-
45	146273377	146280975	L1PA14	7599	-
46	148272840	148279858	L1PA13	7019	+

36	138663528	138670937	L1Md_A	7410	-
37	141561114	141568691	L1Md_T	7578	-
38	150733039	150740066	L1Md_A	7028	-
39	155221323	155228375	L1Md_T	7053	-
