# Supplementary material for: Convergence in LINE-1 nucleotide variations can benefit redundantly forming triplexes with lncRNA in mammalian X-chromosome inactivation
Source: Mob DNA. 2019 Jul 30;10:33. doi: 10.1186/s13100-019-0173-4 (PMC6664574; doi:10.1186/s13100-019-0173-4)
Supplement: Supplementary file 6 — Proportions of r-TC/r-AG motifs occupied in randomly selected L3s and RTEs in opossum X chromosome. Thirty each of L3s (LINE-3s or CR-1s) and RTEs (retrotransposable elements) of the same length ranges as in Fig. 5a (every 100 bp up to 1,000 bp, and every 1,000 bp above 1,000 bp) were randomly selected. Total ranges of the L3 and RTE lengths are 26–3,307 bp and 30–4,097 bp, respectively. The total number and the average proportion of the r-TC/r-AG motifs are indicated at the top and right of each graph, respectively. The tables show the details of the r-TC/r-AG motif proportions in L1s, L2s, L3s, and RTEs examined. (PDF 1242 kb) [file 13100_2019_173_MOESM6_ESM.pdf]

Additional file 6: Proportions of r-TC/r-AG motifs occupied in randomly selected L3s and RTEs in opossum X-chromosome

Thirty of each of L3s (LINE-3s or CR-1s) and RTEs (retrotransposable elements) of the same length ranges as in Figure 5a (every 100 bp up to 1,000 bp, and every 1,000 bp above 1,000 bp) were randomly selected. Total ranges of the L3 and RTE lengths are 26–3,307 bp and 30–4,097 bp, respectively. The total number and the average proportion of the r-TC/r-AG motifs are indicated at the top and right of each graph, respectively. The tables show the details of the r-TC/r-AG motif proportions of L1s, L2s, L3s, and RTEs examined.

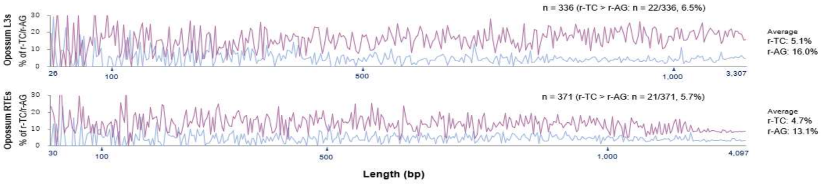

Randomly selected L1s in opossum ChrX

No.	File Name	r-TC (%)	r-AG (%)	r-TC – r-AG (%)	Length
1	monDom5ChrX 20032690-20032728 L1 Mdo3 result	20.51	0.00	-20.51	39
2	monDom5ChrX 56001806-56001844 L1 Mdo3b result	12.82	17.95	5.13	39
3	monDom5ChrX 6142993-6143036 L1-1 MD result	0.00	29.55	29.55	44
4	monDom5ChrX 52888845-52888889 L1 Mars1 result	26.67	0.00	-26.67	45
5	monDom5ChrX 75274808-75274853 L1 Mars1b result	10.87	0.00	-10.87	46
6	monDom5ChrX 67056922-67056968 HAL1 Opos0 result	0.00	40.43	40.43	47
7	monDom5ChrX 10817994-10818041 L1 Opos0 result	14.58	0.00	-14.58	48
8	monDom5ChrX 5447255-5447302 L1 Mars1b result	52.08	14.58	-37.50	48
9	monDom5ChrX 11815799-11815849 L1 Mdo3b result	0.00	13.73	13.73	51
10	monDom5ChrX 16156918-16156968 L1 Mars1a result	19.61	0.00	-19.61	51
11	monDom5ChrX 35530958-3553100 L1 Mdo5 result	0.00	11.76	11.76	51
12	monDom5ChrX 6200537-6200587 L1 Mdo3 result	0.00	19.61	19.61	51
13	monDom5ChrX 14566125-14566176 L1 Mars1a result	0.00	11.54	11.54	52
14	monDom5ChrX 14289158-14289215 L1 Mdo2 result	8.62	0.00	-8.62	58
15	monDom5ChrX 37586647-37586704 L1 Mars1b result	32.76	0.00	-32.76	58
16	monDom5ChrX 25849615-25849675 L1 Mdo1 result	0.00	8.20	8.20	61
17	monDom5ChrX 12004535-12004596 L1 Opos result	38.71	0.00	-38.71	62
18	monDom5ChrX 23052748-23052811 L1 Mdo3c result	0.00	14.06	14.06	64
19	monDom5ChrX 23110742-23110805 L1 Opos result	0.00	46.88	46.88	64
20	monDom5ChrX 26500713-26500781 L1 Mdo2 result	13.04	11.59	-1.45	69
21	monDom5ChrX 13286335-13286404 L1 Mdo3a result	0.00	7.14	7.14	70
22	monDom5ChrX 62764957-62765031 L1 Mars1 result	16.00	9.33	-6.67	75
23	monDom5ChrX 45491071-45491146 L1 Mdo5 result	0.00	38.16	38.16	76
24	monDom5ChrX 15489055-15489132 L1 Mars1 result	7.69	6.41	-1.28	78
25	monDom5ChrX 17633418-17633495 L1 Opos result	0.00	14.10	14.10	78
26	monDom5ChrX 40027614-40027691 L1 Mars1 result	8.97	14.10	5.13	78
27	monDom5ChrX 69019739-69019821 L1 Opos0 result	0.00	14.46	14.46	83
28	monDom5ChrX 54022294-54022377 L1 Mdo2 result	5.95	20.24	14.29	84
29	monDom5ChrX 64571897-64571982 L1?Mdo3b result	0.00	19.77	19.77	86
30	monDom5ChrX 51592590-51592677 L1 Opos3 result	0.00	11.36	11.36	88
31	monDom5ChrX 75492945-75493032 L1 Mars1b result	6.82	13.64	6.82	88
32	monDom5ChrX 4884669-4884757 L1 Opos4b result	0.00	24.72	24.72	89
33	monDom5ChrX 27988271-27988360 HAL1 Opos result	5.56	12.22	6.67	90
34	monDom5ChrX 66897846-66897935 L1 Mdo3c result	7.78	5.56	-2.22	90
35	monDom5ChrX 32362559-32362650 HAL1 Opos result	0.00	36.96	36.96	92
36	monDom5ChrX 70190740-70190832 L1 Mars1b result	0.00	29.03	29.03	93
37	monDom5ChrX 17939696-17939789 HAL1 Opos result	23.40	10.64	-12.77	94
38	monDom5ChrX 43139215-43139308 L1 Mdo3c result	5.32	41.49	36.17	94
39	monDom5ChrX 47772380-47772473 L1 Mdo1 result	0.00	14.89	14.89	94
40	monDom5ChrX 50600833-50600926 L1 Opos0 result	0.00	5.32	5.32	94
41	monDom5ChrX 45847820-45847918 L1 Mdo3c result	0.00	20.20	20.20	99
42	monDom5ChrX 5673502-5673600 L1 Mars1b result	7.07	38.38	31.31	99
43	monDom5ChrX 9088049-9088147 L1 Mars1b result	12.12	15.15	3.03	99
44	monDom5ChrX 13284231-13284330 L1 Opos2 result	0.00	19.00	19.00	100
45	monDom5ChrX 23433169-23433269 L1 Mdo4 result	18.81	17.82	-0.99	101
46	monDom5ChrX 27829624-27829726 L1 Mars1b result	0.00	9.71	9.71	103
47	monDom5ChrX 37813380-37813482 HAL1 Opos result	0.00	6.80	6.80	103
48	monDom5ChrX 47188356-47188458 L1Mars1 result	4.85	20.39	15.53	103
49	monDom5ChrX 38620584-38620688 HAL1 Opos result	0.00	12.38	12.38	105
50	monDom5ChrX 23271148-23271253 L1 Mars1 result	0.00	16.98	16.98	106
51	monDom5ChrX 57999894-58000000 L1 Mars1 result	0.00	29.91	29.91	107
52	monDom5ChrX 5499839-5499946 L1 Mdo3a result	4.63	21.30	16.67	108
53	monDom5ChrX 12594150-12594258 L1 Mars1 result	0.00	16.51	16.51	109
54	monDom5ChrX 22056638-22056747 L1 Mars1b result	6.36	20.00	13.64	110
55	monDom5ChrX 30741697-30741806 L1 Mdo3b result	0.00	19.09	19.09	110
56	monDom5ChrX 8272443-8272555 L1 Mars1b result	5.31	22.12	16.81	113
57	monDom5ChrX 67960711-67960826 L1 Mars1 result	0.00	27.59	27.59	116
58	monDom5ChrX 9345870-9345987 L1 Opos1 result	0.00	18.64	18.64	118
59	monDom5ChrX 46441054-46441174 L1 Mdo6 result	0.00	21.49	21.49	121
60	monDom5ChrX 9618660-9618782 L1 Opos2 result	0.00	39.84	39.84	123
61	monDom5ChrX 19018181-19018304 HAL1 Opos result	10.48	9.68	-0.81	124
62	monDom5ChrX 38953854-38953977 L1 Mars1b result	6.45	23.39	16.94	124
63	monDom5ChrX 6363716-6363839 L1 Mdo4 result	4.03	16.94	12.90	124
64	monDom5ChrX 15162159-15162283 L1 Opos result	0.00	15.20	15.20	125
65	monDom5ChrX 37398191-37398319 L1M5 result	4.65	17.05	12.40	129
66	monDom5ChrX 54144388-54144519 L1 Opos2 result	0.00	5.30	5.30	132
67	monDom5ChrX 51524389-51524522 L1 Mars1b result	29.10	15.67	-13.43	134
68	monDom5ChrX 40812808-40812943 L1 Mdosb result	0.00	25.74	25.74	136
69	monDom5ChrX 42889090-42889227 L1 Mdo5 result	0.00	17.39	17.39	138
70	monDom5ChrX 60184380-60184521 L1 Mdo1 result	0.00	23.94	23.94	142
71	monDom5ChrX 51548483-51548627 L1 Opos0 result	0.00	26.21	26.21	145
72	monDom5ChrX 28001308-28001453 L1 Mdo3a result	0.00	19.86	19.86	146
73	monDom5ChrX 73743408-73743559 L1 Opos result	5.92	40.79	34.87	152
74	monDom5ChrX 30354892-30355045 L1 Mdo6 result	0.00	12.34	12.34	154
75	monDom5ChrX 58474426-58474579 L1 Mars1b result	0.00	17.53	17.53	154
76	monDom5ChrX 33275936-33276095 L1 Mdo6 result	0.00	18.75	18.75	160
77	monDom5ChrX 61094450-61094613 L1 Mdo4 result	3.05	15.85	12.80	164
78	monDom5ChrX 36324651-36324815 L1 Mdo6 result	0.00	13.33	13.33	165
79	monDom5ChrX 10426123-10426290 HAL1N1 MD result	5.95	32.74	26.79	168
80	monDom5ChrX 58506072-58506240 HAL1-2a MD result	10.65	8.28	-2.37	169
81	monDom5ChrX 10696481-10696651 L1 Mars1b result	0.00	14.04	14.04	171
82	monDom5ChrX 54513149-54513320 L1 Mars1 result	8.14	15.70	7.56	172
83	monDom5ChrX 25670297-25670469 HAL1 Opos1 result	2.89	10.98	8.09	173
84	monDom5ChrX 8599707-8599879 L1 Mars1b result	3.47	8.67	5.20	173
85	monDom5ChrX 9058065-9058251 L1 Mars1b result	20.32	11.76	-8.56	187
86	monDom5ChrX 65914379-65914567 L1 Opos0 result	0.00	11.11	11.11	189
87	monDom5ChrX 7721797-7721985 L1 Mars1b result	6.35	27.51	21.16	189
88	monDom5ChrX 38385167-38385356 L1Mars1 result	0.00	19.47	19.47	190
89	monDom5ChrX 21291954-21292147 L1 Mars1b result	4.64	8.76	4.12	194
90	monDom5ChrX 17038074-17038268 L1 Mdo5 result	3.59	25.13	21.54	195
91	monDom5ChrX 18784829-18785024 L1 Opos result	9.69	6.12	-3.57	196
92	monDom5ChrX 60143417-60143614 L1 Mdo5 result	0.00	17.68	17.68	198
93	monDom5ChrX 55139731-55139929 L1 Mdo3a result	3.02	24.12	21.11	199
94	monDom5ChrX 41867252-41867451 L1 Opos0 result	0.00	19.50	19.50	200
95	monDom5ChrX 4943131-4943331 L1 Opos3 result	4.48	13.93	9.45	201
96	monDom5ChrX 19275336-19275537 HAL1-2a MD result	5.94	17.82	11.88	202
97	monDom5ChrX 60469584-60469788 L1 Opos result	2.93	11.22	8.29	205
98	monDom5ChrX 20189625-20189830 L1 Mdo5 result	0.00	6.80	6.80	206
99	monDom5ChrX 17536388-17536596 L1Mdo3a result	0.00	17.22	17.22	209
100	monDom5ChrX 34322779-34322988 L1 Mars1b result	0.00	4.76	4.76	210
101	monDom5ChrX 66015326-66015536 L1 Opos2 result	3.32	17.06	13.74	211
102	monDom5ChrX 69261796-69262007 L1 Opos4b result	0.00	33.96	33.96	212
103	monDom5ChrX 18725022-18725235 L1Mdo3 result	5.14	16.36	11.21	214
104	monDom5ChrX 57267795-57268009 HAL1 Opos result	2.79	22.79	20.00	215
105	monDom5ChrX 22549442-22549658 L1 Mdo3c result	2.30	13.36	11.06	217
106	monDom5ChrX 75275954-75276176 L1 Mars1b result	8.97	4.93	-4.04	223
107	monDom5ChrX 25752225-25752452 HAL1N1 MD result	0.00	20.61	20.61	228
108	monDom5ChrX 24405813-24406044 L1 Opos result	0.00	15.95	15.95	232
109	monDom5ChrX 50915579-50915811 L1Mdo3b result	3.43	15.45	12.02	233
110	monDom5ChrX 70249797-70250031 L1 Mdo5 result	6.81	14.47	7.66	235
111	monDom5ChrX 8662820-8663056 L1 Opos result	2.95	22.36	19.41	237
112	monDom5ChrX 3353149-3353386 L1 Mdo4 result	0.00	9.66	9.66	238
113	monDom5ChrX 41407280-41407520 L1Mdo3a result	0.00	8.71	8.71	241
114	monDom5ChrX 22868743-22868984 L1 Opos4b result	0.00	13.22	13.22	242
115	monDom5ChrX 38612817-38613058 L1 Opos result	4.55	19.42	14.88	242
116	monDom5ChrX 59613919-59614163 L1-1 MD result	2.04	15.10	13.06	245
117	monDom5ChrX 68820368-68820612 L1 Opos4b result	2.45	17.14	14.69	245
118	monDom5ChrX 58616996-58617241 L1 Mars1b result	2.44	13.41	10.98	246
119	monDom5ChrX 8504878-8505123 L1 Opos4b result	2.44	13.82	11.38	246
120	monDom5ChrX 71269991-71270238 L1 Mdo3c result	2.42	12.10	9.68	248
121	monDom5ChrX 61775533-61775786 L1 Mars1b result	0.00	17.32	17.32	254
122	monDom5ChrX 5930493-5930748 L1 Mdo1 result	0.00	14.06	14.06	256
123	monDom5ChrX 78104083-78104345 L1 Mdo6 result	0.00	23.95	23.95	263
124	monDom5ChrX 74111084-74111349 L1 Mdo3b result	4.14	14.29	10.15	266
125	monDom5ChrX 41956612-41956878 L1 Opos result	2.25	25.09	22.85	267
126	monDom5ChrX 45921860-45922129 L1 Mdo3c result	0.00	21.48	21.48	270
127	monDom5ChrX 75693628-75693897 L1 Opos result	0.00	30.00	30.00	270
128	monDom5ChrX 70210345-70210617 L1 Opos0 result	0.00	15.02	15.02	273
129	monDom5ChrX 8619124-8619397 L1 Mars1b result	12.04	14.60	2.55	274
130	monDom5ChrX 16094843-16095117 L1 Opos0 result	8.00	12.00	4.00	275
131	monDom5ChrX 28800759-28801033 L1 Mdo3c result	0.00	29.45	29.45	275
132	monDom5ChrX 44641456-44641733 L1 Opos2 result	7.19	14.03	6.83	278
133	monDom5ChrX 38111708-38111986 L1 Mdo6 result	1.79	21.15	19.35	279
134	monDom5ChrX 68046128-68046408 L1 Mdo4 result	0.00	19.93	19.93	281
135	monDom5ChrX 25799737-25800020 L1 Mdo4 result	5.99	19.72	13.73	284
136	monDom5ChrX 28024304-28024590 HAL1 Opos result	2.44	19.16	16.72	287
137	monDom5ChrX 62696064-62696351 L1 Opos0 result	3.47	13.54	10.07	288
138	monDom5ChrX 55648304-55648594 HAL1 Opos1 result	0.00	19.24	19.24	291
139	monDom5ChrX 77324131-77324423 L1 Mdo3c result	1.71	19.45	17.75	293
140	monDom5ChrX 30513425-30513719 L1 Opos result	0.00	27.80	27.80	295
141	monDom5ChrX 5001366-5001660 L1 Mdo3b result	1.69	27.46	25.76	295
142	monDom5ChrX 43580046-43580341 HAL1 Opos result	9.12	4.39	-4.73	296
143	monDom5ChrX 76924269-76924567 L1 Mars1b result	5.69	12.37	6.69	299
144	monDom5ChrX 4855403-4855706L1 Mdo3 result	1.64	26.97	25.33	304
145	monDom5ChrX 25057500-25057804 L1 Mdo2 result	5.90	20.66	14.75	305
146	monDom5ChrX 64196997-64197305 L1 Mdo3a result	1.62	19.09	17.48	309
147	monDom5ChrX 31400059-31400371 L1 Mdo3a result	0.00	16.29	16.29	313
148	monDom5ChrX 10326288-10326602 L1 Opos2 result	1.90	16.51	14.60	315
149	monDom5ChrX 45166143-45166459 L1 Mdo3b result	3.79	13.56	9.78	317
150	monDom5ChrX 50623936-50624253 L1 Mdo5 result	0.00	30.82	30.82	318
151	monDom5ChrX 36933082-36933400 L1 Opos2 result	1.57	12.23	10.66	319
152	monDom5ChrX 42726948-42727272 L1 Opos4b result	0.00	21.54	21.54	325
153	monDom5ChrX 61065495-61065819 HAL1 Opos result	0.00	12.92	12.92	325
154	monDom5ChrX 54700152-54700478 L1 Opos2 result	6.73	13.76	7.03	327
155	monDom5ChrX 66646983-66647309 L1 Mars1b result	3.36	14.37	11.01	327
156	monDom5ChrX 60237135-60237462 L1 Mdo4 result	3.05	21.04	17.99	328
157	monDom5ChrX 72415466-72415798 L1 Mars1b result	0.00	9.31	9.31	333
158	monDom5ChrX 21986377-21986710 L1 Mdo3c result	0.00	24.85	24.85	334
159	monDom5ChrX 23976744-23977082 L1 Opos4b result	0.00	14.16	14.16	339
160	monDom5ChrX 38611440-38611780 L1 Opos result	2.64	14.66	12.02	341
161	monDom5ChrX 66723864-66724205 HAL1\Opos result	1.46	12.87	11.40	342
162	monDom5ChrX 4997275-4997619 L1 Mdo3b result	3.48	28.99	25.51	345
163	monDom5ChrX 33103989-33104334 L1 Mdo3c result	4.34	6.94	2.60	346
164	monDom5ChrX 19010117-19010463 L1 Opos result	5.48	12.97	7.49	347
165	monDom5ChrX 56710248-56710595 L1 Mars1 result	2.59	30.17	27.59	348
166	monDom5ChrX 18663056-18663404 L1 Opos0 result	1.43	9.46	8.02	349
167	monDom5ChrX 26345863-26346211 L1 Opos2 result	3.44	10.89	7.45	349

Randomly selected L1s in mouse ChrX

No.	File Name	r-TC (%)	r-AG (%)	r-TC – r-AG (%)	Length
1	mm10ChrX 55221798-55221809 L1 Mus1	0.00	50.00	50.00	12
2	mm10ChrX 126554010-126554022 Lx3A	0.00	46.15	46.15	13
3	mm10ChrX 50774010-50774036 L1 Mus1	0.00	18.52	18.52	27
4	mm10ChrX 61744481-61744523 L1 Mur1	16.28	0.00	-16.28	43
5	mm10ChrX 22074489-22074532 L1 Mur1	0.00	11.36	11.36	44
6	mm10ChrX 41861031-41861076 Lx3 Mus	13.04	15.22	2.17	46
7	mm10ChrX 153879407-153879454 Lx6 r	0.00	12.50	12.50	48
8	mm10ChrX 146126447-146126497 L1 Mu	0.00	39.22	39.22	51
9	mm10ChrX 148985320-148985373 Lx5b	9.26	11.11	1.85	54
10	mm10ChrX 79977560-79977615 L1 Mus1	0.00	8.93	8.93	56
11	mm10ChrX 132254684-132254744 Lx6 r	16.39	0.00	-16.39	61
12	mm10ChrX 137481579-137481640 Lx re	0.00	27.42	27.42	62
13	mm10ChrX 128044223-128044285 L1 Mu	0.00	30.16	30.16	63
14	mm10ChrX 43712817-43712879 L1M4c r	0.00	7.94	7.94	63
15	mm10ChrX 62731063-62731125 Lx8 res	0.00	26.98	26.98	63
16	mm10ChrX 99723264-99723327 L1M5 re	0.00	17.19	17.19	64
17	mm10ChrX 60485246-60485310 L1MC4 r	0.00	23.08	23.08	65
18	mm10ChrX 152928449-152928519 L1M2	0.00	7.04	7.04	71
19	mm10ChrX 73820246-73820316 L1M4 re	0.00	19.72	19.72	71
20	mm10ChrX 13341568-13341640 L1MC4a	0.00	6.85	6.85	73
21	mm10ChrX 60868280-60868360 L1M5 re	11.11	28.40	17.28	81
22	mm10ChrX 103665436-103665519 Lx2 r	0.00	20.24	20.24	84
23	mm10ChrX 140119893-140119976 L1MA9	13.10	0.00	-13.10	84
24	mm10ChrX 164276177-16427626 L1Md A	0.00	8.33	8.33	84
25	mm10ChrX 85216230-85216313 L1ME4a	23.81	0.00	-23.81	84
26	mm10ChrX 5740038-5740123 L1VL2 res	0.00	5.81	5.81	86
27	mm10ChrX 147177016-147177102 L1MA4	6.90	25.29	18.39	87
28	mm10ChrX 155549048-155549136 Lx9 r	0.00	21.35	21.35	89
29	mm10ChrX 65875991-65876080 Lx3C re	0.00	20.00	20.00	90
30	mm10ChrX 106914989-106915081 L1MB8	0.00	7.53	7.53	93
31	mm10ChrX 165185778-165185872 Lx9 r	0.00	23.16	23.16	95
32	mm10ChrX 118552775-118552870 L1M4	0.00	16.67	16.67	96
33	mm10ChrX 94263972-94264067 Lx3 Mus	0.00	16.67	16.67	96
34	mm10ChrX 98045110-98045206 L1 Mur3	0.00	22.68	22.68	97
35	mm10ChrX 92795398-92795495 Lx10 re	0.00	6.12	6.12	98
36	mm10ChrX 104274575-104274673 L1MC	0.00	12.12	12.12	99
37	mm10ChrX 55893222-55893321 L1 Mus1	0.00	25.00	25.00	100
38	mm10ChrX 157119076-157119178 Lx9 r	0.00	39.81	39.81	103
39	mm10ChrX 112016234-112016337 Lx2 r	0.00	6.73	6.73	104
40	mm10ChrX 156840273-156840376 Lx7 r	0.00	25.96	25.96	104
41	mm10ChrX 121049889-121049994 L1Md	0.00	6.60	6.60	106
42	mm10ChrX 45167327-45167433 Lx9 res	14.02	5.61	-8.41	107
43	mm10ChrX 169499780-169499887 L1 Mu	4.63	12.04	7.41	108
44	mm10ChrX 111011785-111011895 Lx9 r	0.00	27.93	27.93	111
45	mm10ChrX 135372628-135372738 L1 Mu	0.00	20.72	20.72	111
46	mm10ChrX 29656523-29656633 Lx4A re	0.00	5.41	5.41	111
47	mm10ChrX 39499348-39499459 Lx8b re	0.00	19.64	19.64	112
48	mm10ChrX 100787452-100787564 Lx8 r	0.00	14.16	14.16	113
49	mm10ChrX 126675620-126675737 L1MCa	6.78	11.02	4.24	118
50	mm10ChrX 48645209-48645326 Lx resu	0.00	5.93	5.93	118
51	mm10ChrX 154070790-154070910 L1 Mu	10.74	14.05	3.31	121
52	mm10ChrX 114539888-114540010 L1MA5	0.00	17.89	17.89	123
53	mm10ChrX 97286255-97286379 L1MA8 r	0.00	14.40	14.40	125
54	mm10ChrX 51655630-51655755 L1MD re	0.00	21.43	21.43	126
55	mm10ChrX 48437590-48437716 L1MB8 r	5.51	7.09	1.57	127
56	mm10ChrX 9820213-9820341 Lx8 resul	0.00	17.05	17.05	129
57	mm10ChrX 21359741-21359873 L1 Mur3	0.00	12.78	12.78	133
58	mm10ChrX 151783951-151784084 L1 Ro	4.48	4.48	0.00	134
59	mm10ChrX 149496301-149496435 L1 Mu	0.00	9.63	9.63	135
60	mm10ChrX 36120251-36120394 L1M4c r	0.00	8.33	8.33	144
61	mm10ChrX 146618933-146619078 Lx re	3.42	13.70	10.27	146
62	mm10ChrX 104363227-104363381 L1MA9	3.23	8.39	5.16	155
63	mm10ChrX 30286827-30286984 L1 Mus3	8.86	8.23	-0.63	158
64	mm10ChrX 57530566-57530726 L1ME4a	12.42	10.56	-1.86	161
65	mm10ChrX 114968440-114968604 L1MCa	3.64	13.94	10.30	165
66	mm10ChrX 79110941-79111105 Lx resu	3.64	13.33	9.70	165
67	mm10ChrX 101657216-101657389 L1MA7	0.00	6.90	6.90	174
68	mm10ChrX 19723028-19723202 L1M4 re	5.71	9.14	3.43	175
69	mm10ChrX 78930936-78931111 L1 Mur2	3.98	18.75	14.77	176
70	mm10ChrX 98418498-98418673 L1M2 re	2.84	2.84	0.00	176
71	mm10ChrX 168416487-168416664 L1M4b	2.81	3.37	0.56	178
72	mm10ChrX 7477123-7477300 L1M3 resu	2.81	15.17	12.36	178
73	mm10ChrX 121808028-121808206 L1 Mu	0.00	12.85	12.85	179
74	mm10ChrX 5607899-5608078 L1 Mur2 r	0.00	27.22	27.22	180
75	mm10ChrX 155349177-155349358 L1MB8	2.75	6.04	3.30	182
76	mm10ChrX 61729452-61729633 Lx2 res	0.00	10.99	10.99	182
77	mm10ChrX 117335894-117336076 Lx3A	0.00	12.57	12.57	183
78	mm10ChrX 95648206-95648388 L1 Rod	0.00	4.37	4.37	183
79	mm10ChrX 132655175-132655358 Lx5b	2.72	9.24	6.52	184
80	mm10ChrX 52769241-52769425 L1MA9 r	0.00	11.35	11.35	185
81	mm10ChrX 109944513-109944705 L1M2	0.00	12.44	12.44	193
82	mm10ChrX 113706475-113706667 L1MC2	0.00	19.17	19.17	193
83	mm10ChrX 47039136-47039328 Lx6 res	2.59	21.76	19.17	193
84	mm10ChrX 143498741-143498935 L1MB2	6.15	19.49	13.33	195
85	mm10ChrX 114080504-114080700 L1M3c	0.00	12.69	12.69	197
86	mm10ChrX 43118348-43118545 Lx6 res	3.54	14.14	10.61	198
87	mm10ChrX 110429249-110429449 Lx7 r	9.95	4.98	-4.98	201
88	mm10ChrX 17767717-17767923 Lx8 res	4.83	5.31	0.48	207
89	mm10ChrX 48188927-48189133 Lx8 res	2.90	12.56	9.66	207
90	mm10ChrX 76661977-76662187 L1 Mus1	0.00	7.58	7.58	211
91	mm10ChrX 109469383-109469595 L1 Mu	4.23	10.80	6.57	213
92	mm10ChrX 3871409-3871621 Lx2 resul	0.00	13.62	13.62	213
93	mm10ChrX 56766749-56766963 L1ME4b	0.00	16.74	16.74	215
94	mm10ChrX 101872379-101872594 L1MB8	0.00	7.87	7.87	216
95	mm10ChrX 129925811-129926030L1 Mus	2.27	18.64	16.36	220
96	mm10ChrX 117161028-117161248 L1M2	2.26	13.57	11.31	221
97	mm10ChrX 163752755-163752977 Lx8 r	0.00	13.00	13.00	223
98	mm10ChrX 128049394-128049617 L1 Mu	0.00	10.71	10.71	224
99	mm10ChrX 132240528-132240751 Lx2B2	2.68	9.38	6.70	224
100	mm10ChrX 16416249-16416475 Lx2 res	0.00	5.73	5.73	227
101	mm10ChrX 27878265-27878492 Lx6 res	0.00	19.30	19.30	228
102	mm10ChrX 68619617-68619844 Lx5 res	0.00	14.47	14.47	228
103	mm10ChrX 69139337-69139564 L1MA6 r	6.14	5.26	-0.88	228
104	mm10ChrX 93397898-93398126 L1MD2 r	0.00	11.35	11.35	229
105	mm10ChrX 92343217-92343446 L1M4a1	0.00	17.39	17.39	230
106	mm10ChrX 142434797-142435028 Lx9 r	0.00	14.66	14.66	232
107	mm10ChrX 12423629-12423862 L1MC3 r	2.56	5.13	2.56	234
108	mm10ChrX 145993028-145993263 L1 Mu	2.54	7.63	5.08	236
109	mm10ChrX 95317666-95317902 L1MB4 r	0.00	9.70	9.70	237
110	mm10ChrX 65574911-65575148 L1Md F3	0.00	12.61	12.61	238
111	mm10ChrX 107915711-107915952 L1 Mu	0.00	16.12	16.12	242
112	mm10ChrX 120557530-120557771 L1MB8	5.79	15.29	9.50	242
113	mm10ChrX 153744508-153744751 Lx10	0.00	9.43	9.43	244
114	mm10ChrX 67082933-67083177 L1 Mus1	2.04	11.02	8.98	245
115	mm10ChrX 109366861-109367107 L1MA4	2.43	3.64	1.21	247
116	mm10ChrX 13043943-13044191 L1ME2 r	0.00	16.87	16.87	249
117	mm10ChrX 144512805-144513062 Lx2B	1.94	18.22	16.28	258
118	mm10ChrX 66107683-66107941 L1MB1 r	0.00	5.02	5.02	259
119	mm10ChrX 89333776-89334035 L1MC1 r	3.85	2.31	-1.54	260
120	mm10ChrX 52263359-52263619 Lx4B re	1.92	6.51	4.60	261
121	mm10ChrX 49819872-49820134 Lx8 res	3.80	8.75	4.94	263
122	mm10ChrX 39151310-39151578 L1MA4 r	2.23	4.09	1.86	269
123	mm10ChrX 138744891-138745160 Lx9 r	0.00	16.67	16.67	270
124	mm10ChrX 141802887-141803156 L1M3	0.00	10.74	10.74	270
125	mm10ChrX 91982757-91983027 L1Md F2	4.06	9.59	5.54	271
126	mm10ChrX 16278828-16279100 Lx5c re	4.40	17.58	13.19	273
127	mm10ChrX 13684734-13685008 L1ME3Cz	10.55	19.27	8.73	275
128	mm10ChrX 62469493-62469776 L1 Mur3	0.00	9.86	9.86	284
129	mm10ChrX 146917990-146918274 L1M4b	6.32	8.42	2.11	285
130	mm10ChrX 149645404-149645688 Lx re	0.00	11.93	11.93	285
131	mm10ChrX 62179937-62180221 L1 Mus4	0.00	16.14	16.14	285
132	mm10ChrX 97388375-97388661 Lx8 res	3.14	5.92	2.79	287
133	mm10ChrX 21166541-21166831 Lx8b re	4.12	7.90	3.78	291
134	mm10ChrX 121864553-121864844 L1 Mu	6.16	13.70	7.53	292
135	mm10ChrX 63581911-63582204 Lx2 res	4.08	21.09	17.01	294
136	mm10ChrX 105296016-105296315 L1MB5	3.33	10.00	6.67	300
137	mm10ChrX 47979578-47979878 L1MB2 r	3.99	2.99	-1.00	301
138	mm10ChrX 148413053-148413354 L1 Mu	1.66	17.88	16.23	302
139	mm10ChrX 43443217-43443519 Lx9 res	6.60	10.89	4.29	303
140	mm10ChrX 52084207-52084512 Lx9 res	2.61	17.97	15.36	306
141	mm10ChrX 36163308-36163614 Lx8b re	2.61	23.45	20.85	307
142	mm10ChrX 96354975-96355281 Lx5 res	0.00	14.01	14.01	307
143	mm10ChrX 76723039-76723352 L1Md F2	3.82	12.74	8.92	314
144	mm10ChrX 40646888-40647205 Lx3C re	3.77	20.13	16.35	318
145	mm10ChrX 5967364-5967681 L1MEf res	2.20	10.06	7.86	318
146	mm10ChrX 18731380-18731698 L1M3b r	1.57	14.42	12.85	319
147	mm10ChrX 108491457-108491779 L1Md	0.00	11.15	11.15	323
148	mm10ChrX 92785728-92786051 L1MC1 r	0.00	12.35	12.35	324
149	mm10ChrX 21507249-21507573 1 Mm re	5.54	8.92	3.38	325
150	mm10ChrX 88608418-88608742 Lx7 res	7.38	16.31	8.92	325
151	mm10ChrX 12343832-12344158 L1 Mus4	0.00	17.43	17.43	327
152	mm10ChrX 42803789-42804123 L1 Mur1	1.79	11.04	9.25	335
153	mm10ChrX 78319007-78319341 L1 Mur2	2.09	11.04	8.96	335
154	mm10ChrX 42238444-42238781 L1M2 re	1.78	16.27	14.50	338
155	mm10ChrX 98669165-98669503 Lx8b re	7.08	5.01	-2.06	339
156	mm10ChrX 147186641-147186980 Lx re	3.24	6.76	3.53	340
157	mm10ChrX 66691178-66691517 Lx2 res	2.06	3.82	1.76	340
158	mm10ChrX 143967584-143967924 Lx8 r	7.04	13.20	6.16	341
159	mm10ChrX 111111818-111112159 L1 Mu	1.75	9.06	7.31	342
160	mm10ChrX 133455852-133456195 L1 Mu	2.91	12.79	9.88	344
161	mm10ChrX 53174211-53174554 L1 Mus2	1.45	12.50	11.05	344
162	mm10ChrX 72664308-72664651 L1MEf r	2.03	10.47	8.43	344
163	mm10ChrX 46520791-46521138 Lx9 res	2.01	6.03	4.02	348
164	mm10ChrX 156593729-156594077 L1Md	0.00	10.60	10.60	349
165	mm10ChrX 15600652-15601002 Lx7 res	3.70	18.80	15.10	351
166	mm10ChrX 39811936-39812287 L1 Mus4	0.00	20.17	20.17	352
167	mm10ChrX 108270407-108270762 L1 Mu	1.40	12.08	10.67	356

Randomly selected L1s in human ChrX

No.	File Name	r-TC (%)	r-AG (%)	r-TC – r-AG (%)	Length
1	hg38ChrX 118908518-118908534 L1MA9	0.00	82.35	82.35	17
2	hg38ChrX 46891108-46891139 L1ME4b	0.00	15.63	15.63	32
3	hg38ChrX 2523531-2523568 L1MC3 res	0.00	31.58	31.58	38
4	hg38ChrX 15040958-15040996 L1MB5 r	20.51	0.00	-20.51	39
5	hg38ChrX 123858652-123858694 L1MB4	0.00	13.95	13.95	43
6	hg38ChrX 47434659-47434703 L1M5 re	0.00	11.11	11.11	45
7	hg38ChrX 68384390-68384437 L1PB2 r	10.42	14.58	4.17	48
8	hg38ChrX 133717261-133717309 L1M4	20.41	26.53	6.12	49
9	hg38ChrX 15300900-15300948 L1ME4b	0.00	26.53	26.53	49
10	hg38ChrX 102158828-102158877 L1ME3	0.00	10.00	10.00	50
11	hg38ChrX 24038718-24038773 L1MB8 r	0.00	12.50	12.50	56
12	hg38ChrX 87707787-87707845 L1M2 re	0.00	27.12	27.12	59
13	hg38ChrX 47793934-47794000 L1MD2 r	0.00	8.96	8.96	67
14	hg38ChrX 52196749-52196815 L1ME3A	0.00	10.45	10.45	67
15	hg38ChrX 46501958-46502025 L1MEd r	0.00	7.35	7.35	68
16	hg38ChrX 24194228-24194297 HAL1 re	0.00	20.00	20.00	70
17	hg38ChrX 154733770-154733840 L1MD	0.00	18.31	18.31	71
18	hg38ChrX 134421840-134421913 L1ME4	6.76	9.46	2.70	74
19	hg38ChrX 142979313-142979390 L1ME3	0.00	6.41	6.41	78
20	hg38ChrX 128963427-128963505 L1ME2	0.00	6.33	6.33	79
21	hg38ChrX 148272468-148272550 HAL1M	0.00	6.02	6.02	83
22	hg38ChrX 22507993-22508075 L1M3A r	6.02	0.00	-6.02	83
23	hg38ChrX 44343999-44344082 L1ME3Cz	7.14	7.14	0.00	84
24	hg38ChrX 136901680-136901764 L1MD	12.94	8.24	-4.71	85
25	hg38ChrX 83781534-83781618 L1MA6 r	7.06	12.94	5.88	85
26	hg38ChrX 108511032-108511117 L1MC4	15.12	0.00	-15.12	86
27	hg38ChrX 45605306-45605391 L1PA15	0.00	5.81	5.81	86
28	hg38ChrX 77344766-77344851 HAL1b r	13.95	8.14	-5.81	86
29	hg38ChrX 21171018-21171105 HAL1ME	0.00	6.82	6.82	88
30	hg38ChrX 32229591-32229681 L1MA10	6.59	0.00	-6.59	91
31	hg38ChrX 85658572-85658669 L1MA9 r	0.00	16.33	16.33	98
32	hg38ChrX 131701515-131701617 L1M3	5.83	10.68	4.85	103
33	hg38ChrX 83406638-83406744 L1MA3 r	0.00	29.91	29.91	107
34	hg38ChrX 24524741-24524850 HAL1ME	9.09	24.55	15.45	110
35	hg38ChrX 54268128-54268239 L1MC4 r	8.93	6.25	-2.68	112
36	hg38ChrX 16809232-16809344 L1ME4b	4.42	5.31	0.88	113
37	hg38ChrX 38873865-38873979 L1ME4a	4.35	6.09	1.74	115
38	hg38ChrX 94161312-94161426 L1PA15	0.00	10.43	10.43	115
39	hg38ChrX 133463637-133463752 L1PA1	5.17	0.00	-5.17	116
40	hg38ChrX 131410796-131410912 L1MA4	5.98	10.26	4.27	117
41	hg38ChrX 72771119-72771239 L1MEh r	0.00	9.92	9.92	121
42	hg38ChrX 20100278-20100399 L1ME3 r	0.00	15.57	15.57	122
43	hg38ChrX 141139298-141139420 L1PA1	0.00	5.69	5.69	123
44	hg38ChrX 104536723-104536847 L1MEg	4.00	8.80	4.80	125
45	hg38ChrX 36328847-36328971 L1M5 re	0.00	8.00	8.00	125
46	hg38ChrX 136374771-136374896 L1ME2	0.00	20.63	20.63	126
47	hg38ChrX 32262232-32262365 L1ME1 r	0.00	14.93	14.93	134
48	hg38ChrX 52439604-52439738 L1MA3 r	4.44	0.00	-4.44	135
49	hg38ChrX 22016178-22016313 L1M5 re	0.00	3.68	3.68	136
50	hg38ChrX 113093902-113094038 L1PB1	4.38	3.65	-0.73	137
51	hg38ChrX 134993574-134993710 L1ME4	0.00	4.38	4.38	137
52	hg38ChrX 83001903-83002044 L1ME3Cz	3.52	11.97	8.45	142
53	hg38ChrX 33839188-33839334 L1MC4 r	6.12	17.69	11.56	147
54	hg38ChrX 106175738-106175886 L1ME4	3.36	19.46	16.11	149
55	hg38ChrX 136595411-136595565 L1MD2	3.87	10.97	7.10	155
56	hg38ChrX 153836369-153836525 L1ME3	3.18	4.46	1.27	157
57	hg38ChrX 95974353-95974509 L1MA8 r	4.46	16.56	12.10	157
58	hg38ChrX 151859959-151860116 L1M4	4.43	12.03	7.59	158
59	hg38ChrX 35608819-35608977 L1MA9 r	0.00	11.32	11.32	159
60	hg38ChrX 116204954-116205113 L1MB1	0.00	23.75	23.75	160
61	hg38ChrX 145533008-145533171 L1M5	3.05	3.66	0.61	164
62	hg38ChrX 65656072-65656236 L1ME3G	0.00	7.27	7.27	165
63	hg38ChrX 88052743-88052911 L1MA9 r	0.00	3.55	3.55	169
64	hg38ChrX 150957324-150957499 L1ME5	0.00	11.93	11.93	176
65	hg38ChrX 90290799-90290978 L1MEc r	0.00	10.00	10.00	180
66	hg38ChrX 147680842-147681023 L1ME4	16.48	6.59	-9.89	182
67	hg38ChrX 32236281-32236462 L1ME3Cz	2.75	2.75	0.00	182
68	hg38ChrX 132900561-132900747 L1MB8	0.00	17.11	17.11	187
69	hg38ChrX 125471801-125471989 L1MD3	2.65	11.64	8.99	189
70	hg38ChrX 73989545-73989734 L1MEf r	0.00	13.68	13.68	190
71	hg38ChrX 125416214-125416406 L1ME2	0.00	3.63	3.63	193
72	hg38ChrX 152887775-152887969 L1ME3	3.08	9.23	6.15	195
73	hg38ChrX 122519943-122520143 L1MB4	0.00	8.46	8.46	201
74	hg38ChrX 132047784-132047986 L1MA8	0.00	10.84	10.84	203
75	hg38ChrX 3614013-3614215 L1MA4A re	0.00	7.39	7.39	203
76	hg38ChrX 18561217-18561420 L1ME4a	0.00	12.75	12.75	204
77	hg38ChrX 41838153-41838356 L1MD2 r	0.00	2.45	2.45	204
78	hg38ChrX 35214544-35214749 L1M4 re	3.40	6.31	2.91	206
79	hg38ChrX 75670912-75671117 L1MA5 r	0.00	12.62	12.62	206
80	hg38ChrX 24690881-24691087 L1PB1 r	0.00	3.86	3.86	207
81	hg38ChrX 53657083-53657291 L1MC1 r	3.35	14.35	11.00	209
82	hg38ChrX 144828968-144829177 L1MC5	2.38	12.86	10.48	210
83	hg38ChrX 87722030-87722239 L1ME2 r	2.38	0.00	-2.38	210
84	hg38ChrX 147567067-147567277 HAL1b	9.48	2.37	-7.11	211
85	hg38ChrX 3772204-3772414 L1MB5 res	6.16	2.37	-3.79	211
86	hg38ChrX 117214969-117215180 L1MA8	2.36	13.68	11.32	212
87	hg38ChrX 107237487-107237702 L1MB5	3.70	9.26	5.56	216
88	hg38ChrX 140185092-140185307 L1MC5	2.78	14.35	11.57	216
89	hg38ChrX 26671826-26672041 L1MA9 r	0.00	2.31	2.31	216
90	hg38ChrX 29779401-29779618 L1PA15	0.00	9.17	9.17	218
91	hg38ChrX 75895927-75896145 L1PA7 r	8.68	10.50	1.83	219
92	hg38ChrX 147105033-147105258 L1MC	0.00	18.14	18.14	226
93	hg38ChrX 20899319-20899544 HAL1 re	5.31	13.72	8.41	226
94	hg38ChrX 80707864-80708091 L1MC5 r	2.63	4.82	2.19	228
95	hg38ChrX 136837554-136837783 L1MD2	2.17	14.78	12.61	230
96	hg38ChrX 140016270-140016501 L1P1	0.00	14.22	14.22	232
97	hg38ChrX 44592485-44592728 L1ME3Cz	4.10	2.87	-1.23	244
98	hg38ChrX 150046422-150046666 HAL1M	3.67	10.20	6.53	245
99	hg38ChrX 26681337-26681581 L1P4 re	2.45	15.10	12.65	245
100	hg38ChrX 121884339-121884584 HAL1M	0.00	22.76	22.76	246
101	hg38ChrX 853302-853549 L1MA6 resul	0.00	9.27	9.27	248
102	hg38ChrX 56135725-56135975 L1PA15	4.78	17.53	12.75	251
103	hg38ChrX 43150690-43150943 HAL1 re	10.24	17.32	7.09	254
104	hg38ChrX 48373065-48373318 L1MD2 r	0.00	7.87	7.87	254
105	hg38ChrX 48739660-48739913 L1MD re	0.00	22.83	22.83	254
106	hg38ChrX 10036341-10036595 L1MB5 r	3.14	9.02	5.88	255
107	hg38ChrX 35181153-35181411 L1MB5 r	0.00	15.83	15.83	259
108	hg38ChrX 34262316-34262575 L1MC4a	0.00	5.00	5.00	260
109	hg38ChrX 101970354-101970614 L1ME2	0.00	9.96	9.96	261
110	hg38ChrX 85010100-85010362 L1MC re	0.00	10.65	10.65	263
111	hg38ChrX 79358261-79358524 L1PA3 r	0.00	4.92	4.92	264
112	hg38ChrX 41294712-41294980 L1MC5 r	2.60	13.75	11.15	269
113	hg38ChrX 69044938-69045207 L1M5 re	4.81	15.19	10.37	270
114	hg38ChrX 37057355-37057630 L1MA9 r	0.00	6.88	6.88	276
115	hg38ChrX 121852060-121852342 L1M1	4.59	9.54	4.95	283
116	hg38ChrX 54578043-54578325 L1ME1 r	3.53	12.37	8.83	283
117	hg38ChrX 155002677-155002961 L1MB2	2.46	15.44	12.98	285
118	hg38ChrX 19907454-19907738 L1PB1 r	1.75	7.37	5.61	285
119	hg38ChrX 74618579-74618866 L1ME4a	2.08	10.76	8.68	288
120	hg38ChrX 101034442-101034732 L1MC5	1.72	2.06	0.34	291
121	hg38ChrX 153293570-153293865 L1MB5	5.41	18.92	13.51	296
122	hg38ChrX 155177687-155177982 L1MEf	1.69	5.74	4.05	296
123	hg38ChrX 35609117-35609416 L1M1 re	2.67	4.00	1.33	300
124	hg38ChrX 96533788-96534089 L1MA7 r	0.00	8.94	8.94	302
125	hg38ChrX 52154809-52155111 L1MA9 r	2.31	12.54	10.23	303
126	hg38ChrX 10875054-10875357 L1ME4b	7.24	11.51	4.28	304
127	hg38ChrX 108086628-108086938 L1ME4	3.86	7.72	3.86	311
128	hg38ChrX 126582189-126582500 L1M4c	1.60	25.64	24.04	312
129	hg38ChrX 13586055-13586367 L1MB7 r	1.92	12.78	10.86	313
130	hg38ChrX 144511307-144511620 L1PA1	0.00	5.10	5.10	314
131	hg38ChrX 51914359-51914672 L1MA4 r	0.00	22.61	22.61	314
132	hg38ChrX 65155258-65155571 L1MEi r	2.23	12.42	10.19	314
133	hg38ChrX 140332217-140332532 L1MA7	0.00	11.71	11.71	316
134	hg38ChrX 150797446-150797762 L1MB4	1.89	15.77	13.88	317
135	hg38ChrX 65337915-65338231 L1PA8 r	1.58	11.04	9.46	317
136	hg38ChrX 47694105-47694422 L1MC4 r	3.46	11.95	8.49	318
137	hg38ChrX 2986690-2987008 L1PA4 res	1.57	5.64	4.08	319
138	hg38ChrX 80361836-80362159 L1PA16	1.85	10.19	8.33	324
139	hg38ChrX 117773805-117774129 L1MB7	1.54	17.54	16.00	325
140	hg38ChrX 155791594-155791919 L1MA1	1.84	7.67	5.83	326
141	hg38ChrX 92484521-92484847 L1PB1 r	0.00	14.37	14.37	327
142	hg38ChrX 106538640-106538982 L1M4	1.75	19.53	17.78	343
143	hg38ChrX 130337006-130337349 L1M5	0.00	11.05	11.05	344
144	hg38ChrX 19860294-19860639 L1MB4 r	1.45	8.96	7.51	346
145	hg38ChrX 54052898-54053244 L1P1 re	3.75	7.49	3.75	347
146	hg38ChrX 54148016-54148364 L1ME1 r	1.43	16.91	15.47	349
147	hg38ChrX 94805072-94805421 L1P1 re	4.00	8.00	4.00	350
148	hg38ChrX 34177231-34177583 L1MD2 r	2.83	8.50	5.67	353
149	hg38ChrX 74872205-74872559 L1MA3 r	0.00	8.73	8.73	355
150	hg38ChrX 17869377-17869733 L1ME4c	6.44	5.88	-0.56	357
151	hg38ChrX 2712052-2712409 L1MA8 res	0.00	5.31	5.31	358
152	hg38ChrX 35752522-35752879 L1MB1 r	6.70	12.01	5.31	358
153	hg38ChrX 93117932-93118289 L1PA15	3.35	9.50	6.15	358
154	hg38ChrX 134519472-134519831 L1PA1	1.67	8.06	6.39	360
155	hg38ChrX 76683175-76683535 L1PB4 r	1.39	7.76	6.37	361
156	hg38ChrX 13701072-13701433 L1MB7 r	0.00	5.25	5.25	362
157	hg38ChrX 84021739-84022101 L1MB7 r	0.00	7.71	7.71	363
158	hg38ChrX 35671676-35672039 L1PA16	1.37	12.91	11.54	364
159	hg38ChrX 36997503-36997866 L1M4 re	0.00	25.27	25.27	364
160	hg38ChrX 126746242-126746607 L1MC2	1.37	14.48	13.11	366
161	hg38ChrX 24243860-24244227 L1ME3G	2.99	8.70	5.71	368
162	hg38ChrX 7214552-7214919 L1PB1 res	0.00	6.25	6.25	368
163	hg38ChrX 39710029-39710397 HAL1 re	0.00	19.78	19.78	369
164	hg38ChrX 139361185-139361554 L1MC	1.35	11.35	10.00	370
165	hg38ChrX 54703192-54703564 L1ME3Cz	1.61	13.67	12.06	373
166	hg38ChrX 37748936-37749309 HAL1 re	1.60	13.37	11.76	374
167	hg38ChrX 84327384-84327762 L1ME1 r	3.17	11.61	8.44	379

168	monDom5ChrX 27697047-27697395 L1 Mdo3 result	4.87	5.44	0.57	349
169	monDom5ChrX 46229952-46230300 L1 Opos0 result	3.44	14.04	10.60	349
170	monDom5ChrX 46405634-46405983 HAL1 Opos result	2.57	20.29	17.71	350
171	monDom5ChrX 49670853-49671202 HAL1 Opos result	6.00	11.71	5.71	350
172	monDom5ChrX 20329372-20329726 L1 Mars1 result	1.41	18.59	17.18	355
173	monDom5ChrX 36162322-36162679 L1 Opos0 result	1.40	15.64	14.25	358
174	monDom5ChrX 47297340-47297697 L1 Mdo3c result	6.15	16.20	10.06	358
175	monDom5ChrX 58664357-58664714 L1 Mars1b result	3.07	12.85	9.78	358
176	monDom5ChrX 13589663-13590021 L1 Mdo2 result	0.00	11.42	11.42	359
177	monDom5ChrX 24783463-24783825 L1 Opos0 result	0.00	17.63	17.63	363
178	monDom5ChrX 66326630-66326996 L1 Mdo6 result	1.36	25.61	24.25	367
179	monDom5ChrX 30601469-30601836 L1 Opos4b result	5.43	7.61	2.17	368
180	monDom5ChrX 28098473-28098844 HAL1 Opos result	5.65	15.32	9.68	372
181	monDom5ChrX 47213983-47214357 L1 Opos0 result	3.73	9.87	6.13	375
182	monDom5ChrX 38514674-38515052 L1 Mdo3b result	1.85	16.36	14.51	379
183	monDom5ChrX 64726874-64727252 L1 Mdo3a result	0.00	21.90	21.90	379
184	monDom5ChrX 7862208-7862587 L1 Mars1 result	3.16	20.00	16.84	380
185	monDom5ChrX 32509624-32510004 L1 Opos3 result	1.31	11.81	10.50	381
186	monDom5ChrX 71473571-71473952 L1 Mars1 result	4.71	16.75	12.04	382
187	monDom5ChrX 36213921-36214305 HAL1N1 MD result	1.56	27.27	25.71	385
188	monDom5ChrX 36460737-36461122 L1 Opos0 result	0.00	10.10	10.10	386
189	monDom5ChrX 10783721-10784107 HAL1N1 MD result	3.62	19.12	15.50	387
190	monDom5ChrX 47615141-47615528 L1-1 MD result	2.58	18.56	15.98	388
191	monDom5ChrX 9925068-9925457 L1 Mdo3c result	1.28	21.79	20.51	390
192	monDom5ChrX 48013144-48013540 L1 Mdo3c result	0.00	20.65	20.65	397
193	monDom5ChrX 26955713-26956110 L1 Mars1 result	5.78	11.56	5.78	398
194	monDom5ChrX 19555403-19555802 L1 Mars1b result	2.50	18.25	15.75	400
195	monDom5ChrX 43251358-43251757 L1 Opos4b result	3.00	17.50	14.50	400
196	monDom5ChrX 40176050-40176454 L1 Opos0 result	2.96	14.07	11.11	405
197	monDom5ChrX 9544212-9544619 L1Mdo3c result	0.00	19.85	19.85	408
198	monDom5ChrX 27257614-27258026 L1 Mdo3c result	1.69	23.49	21.79	413
199	monDom5ChrX 37655970-37656382 L1 Mars1b result	4.36	12.59	8.23	413
200	monDom5ChrX 3356122-3356536 HAL1 Opos result	4.82	10.12	5.30	415
201	monDom5ChrX 67295775-67296192 L1 Mars1b result	3.83	20.10	16.27	418
202	monDom5ChrX 37756803-37757222 L1 Mdo1 result	1.19	19.29	18.10	420
203	monDom5ChrX 49670431-49670856 L1 Mars1b result	5.87	19.95	14.08	426
204	monDom5ChrX 74082388-74082814 L1 Mdo3c result	3.28	24.82	21.55	427
205	monDom5ChrX 57557355-57557782 L1 Mdo3c result	0.00	20.79	20.79	428
206	monDom5ChrX 68275604-68276032 L1 Mdo5 result	3.73	13.75	10.02	429
207	monDom5ChrX 20206842-20207272 L1 Mdo3a result	4.18	16.71	12.53	431
208	monDom5ChrX 55393451-55393882 L1 Mdo3b result	1.62	23.15	21.53	432
209	monDom5ChrX 53349597-53350031 L1 Opos result	5.75	12.41	6.67	435
210	monDom5ChrX 12168210-12168645 L1 Opos2 result	5.73	17.20	11.47	436
211	monDom5ChrX 47649089-47649527 L1 Mars1b result	0.00	18.68	18.68	439
212	monDom5ChrX 52851383-52851830 L1 Opos result	4.02	20.31	16.29	448
213	monDom5ChrX 56889311-56889758 L1 Mdo3b result	1.56	19.20	17.63	448
214	monDom5ChrX 15426624-15427072 L1 Mdo6 result	0.00	22.27	22.27	449
215	monDom5ChrX 13296567-13297016 L1 Mdo3 result	3.78	10.22	6.44	450
216	monDom5ChrX 36813596-36814045 L1 Mars1b result	4.00	20.22	16.22	450
217	monDom5ChrX 42287072-42287524 L1 Mdo3c result	3.75	19.21	15.45	453
218	monDom5ChrX 46483258-46483711 L1 Mdo2 result	5.51	10.79	5.29	454
219	monDom5ChrX 54201461-54201916 L1 Opos3 result	0.00	13.60	13.60	456
220	monDom5ChrX 60403718-60404175 L1 Opos4b result	5.68	21.40	15.72	458
221	monDom5ChrX 24678431-24678889 L1 Mdo3a result	2.83	20.70	17.86	459
222	monDom5ChrX 43504270-43504728 L1 Mars1b result	2.40	18.95	16.56	459
223	monDom5ChrX 7806962-7807422 L1 Opos result	1.30	15.40	14.10	461
224	monDom5ChrX 16240129-16240591 L1 Mdo6 result	3.89	14.47	10.58	463
225	monDom5ChrX 67873507-67873969 L1 Opos2 result	2.16	12.10	9.94	463
226	monDom5ChrX 40661149-40661614 L1 Opos0 result	3.43	16.95	13.52	466
227	monDom5ChrX 13976458-13976928 L1 Opos result	1.06	18.26	17.20	471
228	monDom5ChrX 21191189-21191663 L1 Mars1b result	0.00	19.37	19.37	475
229	monDom5ChrX 22652899-22653374 L1 Mdo3 result	6.93	5.88	-1.05	476
230	monDom5ChrX 12683651-12684127 L1 Opos0 result	5.66	11.74	6.08	477
231	monDom5ChrX 78491009-78491485 L1 Opos result	2.31	15.93	13.63	477
232	monDom5ChrX 12539948-12540425 L1 Mdo2 result	5.23	8.58	3.35	478
233	monDom5ChrX 12814899-12815378 L1 Mso3c result	0.00	10.63	10.63	480
234	monDom5ChrX 41471841-41472321 L1 Opos1 result	6.65	9.15	2.49	481
235	monDom5ChrX 56378020-56378500 L1 Mars1b result	3.53	18.71	15.18	481
236	monDom5ChrX 58291452-58291933 L1 Opos0 result	3.32	17.01	13.69	482
237	monDom5ChrX 21606793-21607275 L1 Mdo4 result	3.93	15.32	11.39	483
238	monDom5ChrX 32138321-32138804 L1 Mars1b result	0.00	12.40	12.40	484
239	monDom5ChrX 43792861-43793344 L1 Mars1b result	2.48	17.36	14.88	484
240	monDom5ChrX 62621481-62621971 L1 Mars1b result	4.68	19.35	14.66	491
241	monDom5ChrX 25020447-25020938 L1 Opos0 result	3.05	19.31	16.26	492
242	monDom5ChrX 46907720-46908215 L1 Mdo5 result	4.64	15.12	10.48	496
243	monDom5ChrX 25608784-25609280 L1 Opos0 result	3.22	15.49	12.27	497
244	monDom5ChrX 13986260-13986760 L1 Opos result	5.19	19.36	14.17	501
245	monDom5ChrX 15246886-15247388 L1 Mdo3b result	1.39	18.89	17.50	503
246	monDom5ChrX 24402882-24403384 L1 Mdo5 result	0.99	22.66	21.67	503
247	monDom5ChrX 19161600-19162103 HAL1 Opos1 result	1.98	10.32	8.33	504
248	monDom5ChrX 23433419-23433925 L1 Mdo4 result	7.89	8.88	0.99	507
249	monDom5ChrX 31698351-31698857 L1 Mdo3c result	2.37	13.81	11.44	507
250	monDom5ChrX 11637263-11637773 L1 Mdo6 result	3.33	18.98	15.66	511
251	monDom5ChrX 33260353-33260864 L1 Mars1b result	1.95	15.43	13.48	512
252	monDom5ChrX 79100662-79101173 L1 Opos2 result	3.52	16.41	12.89	512
253	monDom5ChrX 12721724-12722236 L1 Mdo4 result	4.09	12.48	8.38	513
254	monDom5ChrX 49039264-49039776 L1 Mdo3b result	0.00	19.88	19.88	513
255	monDom5ChrX 20172949-20173463 L1 Mars1b result	8.16	14.37	6.21	515
256	monDom5ChrX 61871574-61872088 L1 Mdo3a result	1.17	19.22	18.06	515
257	monDom5ChrX 51538582-51539098 L1 Opos result	3.87	17.79	13.93	517
258	monDom5ChrX 7767487-7768004 L1 Mdo6 result	1.35	17.57	16.22	518
259	monDom5ChrX 16801734-16802252 L1 Opos result	3.66	20.81	17.15	519
260	monDom5ChrX 79136116-79136639 L1 Mdo4 result	1.91	16.41	14.50	524
261	monDom5ChrX 67339534-67340060 L1 Mdo3 result	3.23	17.27	14.04	527
262	monDom5ChrX 4070795-4071322 L1 Mdo3c result	1.33	16.48	15.15	528
263	monDom5ChrX 6086482-6087009 L1 Mdo6 result	2.08	14.96	12.88	528
264	monDom5ChrX 54063770-54064298 L1 Mdo5 result	3.59	15.12	11.53	529
265	monDom5ChrX 39194745-39195277 L1 Mdo3a result	0.94	18.20	17.26	533
266	monDom5ChrX 68718273-68718806 L1 Opos4b result	1.50	19.29	17.79	534
267	monDom5ChrX 60929945-60930480 L1 Opos0 result	2.24	13.99	11.75	536
268	monDom5ChrX 4297685-4298221 L1 Opos0 result	2.23	14.90	12.66	537
269	monDom5ChrX 77726518-77727058 L1 Opos3 result	0.92	9.61	8.69	541
270	monDom5ChrX 55283608-55284152 L1Opos0 result	5.14	16.51	11.38	545
271	monDom5ChrX 27992661-27993206 L1 Mdo3b result	2.38	7.51	5.13	546
272	monDom5ChrX 56426696-56427242 L1 Mars1a result	5.67	15.17	9.51	547
273	monDom5ChrX 43503311-43503858 L1 Mars1b result	0.00	10.40	10.40	548
274	monDom5ChrX 76277105-76277652 L1 Opos2 result	5.84	14.05	8.21	548
275	monDom5ChrX 38945776-38946327 L1 Opos result	0.91	21.01	20.11	552
276	monDom5ChrX 39179177-39179728 L1 Opos result	1.09	22.10	21.01	552
277	monDom5ChrX 17585351-17585905 L1 Mars1 result	5.23	17.84	12.61	555
278	monDom5ChrX 25194268-25194824 HAL1 Opos1 result	1.26	16.88	15.62	557
279	monDom5ChrX 63888725-63889281 L1 Opos2 result	5.92	24.06	18.13	557
280	monDom5ChrX 25629214-25629771 HAL1 Opos1 result	2.33	16.85	14.52	558
281	monDom5ChrX 67209273-67209830 HAL1 Opos1 result	0.90	12.19	11.29	558
282	monDom5ChrX 49055448-49056006 L1-1 MD result	1.79	20.75	18.96	559
283	monDom5ChrX 38078924-38079487 L1 Opos result	1.95	21.28	19.33	564
284	monDom5ChrX 27566195-27566762 L1 Mars1 result	2.29	13.73	11.44	568
285	monDom5ChrX 30689747-30690327 HAL1 Opos result	6.54	14.80	8.26	581
286	monDom5ChrX 20712719-20713301 L1 Mdo3c result	1.03	19.21	18.18	583
287	monDom5ChrX 75757254-75757838 L1 Opos result	2.91	15.73	12.82	585
288	monDom5ChrX 34333823-34334412 L1 Mdo5 result	5.25	20.34	15.08	590
289	monDom5ChrX 36486347-36486937 L1 Mars1b result	2.54	13.87	11.34	591
290	monDom5ChrX 76871464-76872056 HAL1 Opos1 result	1.01	30.02	29.01	593
291	monDom5ChrX 48395509-48396104 L1 Mdo2 result	3.52	19.80	16.28	596
292	monDom5ChrX 69104043-69104639 L1 Opos result	2.85	23.12	20.27	597
293	monDom5ChrX 48990719-48991316 L1 Opos0 result	3.01	16.72	13.71	598
294	monDom5ChrX 69657940-69658539L1 Opos1 result	5.67	15.33	9.67	600
295	monDom5ChrX 61850897-61851500 L1 Opos1 result	3.31	18.05	14.74	604
296	monDom5ChrX 43957065-43957670 L1 Mars1b result	9.41	15.35	5.94	606
297	monDom5ChrX 73549772-73550378 L1 Opos0 result	3.95	15.32	11.37	607
298	monDom5ChrX 48468257-48468864 L1 Opos4b result	0.82	18.59	17.76	608
299	monDom5ChrX 42865650-42866258 L1 Opos1 result	3.45	17.08	13.63	609
300	monDom5ChrX 9555476-9556086 L1 Mdo3c result	2.95	19.64	16.69	611
301	monDom5ChrX 38885774-38886390 L1 Opos1 result	0.00	17.83	17.83	617
302	monDom5ChrX 66299815-66300440 L1 Mars1b result	3.51	11.66	8.15	626
303	monDom5ChrX 74368766-74369394 L1 Mdo3b result	3.18	22.89	19.71	629
304	monDom5ChrX 44348175-44348806 L1 Opos0 result	4.27	15.82	11.55	632
305	monDom5ChrX 3286443-3287075 L1 Opos2 result	4.58	12.80	8.21	633
306	monDom5ChrX 78938506-78939138 L1 Mdo3b result	0.00	15.48	15.48	633
307	monDom5ChrX 10858270-10858905 L1 Mars1b result	1.57	15.41	13.84	636
308	monDom5ChrX 37370776-37371412 L1 Mdo3b result	4.08	17.27	13.19	637
309	monDom5ChrX 38732070-38732706 HAL1 Opos result	3.61	20.72	17.11	637
310	monDom5ChrX 46027380-46028016L1 Mdo3b result	1.73	17.74	16.01	637
311	monDom5ChrX 68412720-68413358 L1 Mdo3b result	4.69	10.80	6.10	639
312	monDom5ChrX 18085280-18085919 HAL1 Opos result	0.94	15.16	14.22	640
313	monDom5ChrX 26584465-26585104 L1 Mdo5 result	4.22	22.97	18.75	640
314	monDom5ChrX 63779287-63779927 L1 Opos1 result	5.46	15.60	10.14	641
315	monDom5ChrX 65929739-65930379 L1 Opos3 result	6.08	17.16	11.08	641
316	monDom5ChrX 43685652-43686295 L1 Mars1b result	4.35	19.88	15.53	644
317	monDom5ChrX 31209329-31209974 L1 Opos2 result	1.08	11.92	10.84	646
318	monDom5ChrX 75064263-75064908 L1 Mdo3a result	2.79	19.04	16.25	646
319	monDom5ChrX 16065939-16066586 L1 Mars1b result	2.31	11.11	8.80	648
320	monDom5ChrX 23973810-23974457 L1 Opos result	2.93	12.19	9.26	648
321	monDom5ChrX 20784735-20785383 L1 Mars1 result	5.08	13.25	8.17	649
322	monDom5ChrX 25855456-25856105 L1 Mdo3b result	3.69	17.69	14.00	650
323	monDom5ChrX 34914950-34915606 L1 Opos0 result	1.83	15.07	13.24	657
324	monDom5ChrX 68620929-68621585 L1 Mars1b result	3.35	13.55	10.20	657
325	monDom5ChrX 35575209-35575867 L1 Opos0 result	2.43	17.15	14.72	659
326	monDom5ChrX 29924199-29924860 L1 Opos result	6.04	28.55	22.51	662
327	monDom5ChrX 62445143-62445804 L1 Opos2 result	1.06	17.82	16.77	662
328	monDom5ChrX 66318313-66318979 L1 Mdo3c result	1.65	22.04	20.39	667
329	monDom5ChrX 9941009-9941676 L1 Mdo3b result	2.69	20.06	17.37	668
330	monDom5ChrX 21037115-21037788 L1 Mdo3b result	4.30	17.06	12.76	674
331	monDom5ChrX 13322902-13323578 L1 Mdo3c result	0.89	20.24	19.35	677
332	monDom5ChrX 27058378-27059056 L1 Mso3a result	1.91	17.23	15.32	679
333	monDom5ChrX 76145644-76146326 L1 Mdo5 result	1.61	19.47	17.86	683
334	monDom5ChrX 76872496-76873179 L1 Mars1 result	2.63	17.25	14.62	684
335	monDom5ChrX 31252929-31253614 L1 Mdo3b result	1.90	21.87	19.97	686
336	monDom5ChrX 5561725-5562411 L1 Mars1b result	0.87	11.35	10.48	687
337	monDom5ChrX 25704450-25705137 HAL1N1 MD result	2.76	17.44	14.68	688
338	monDom5ChrX 25918356-25919043 HAL1N1 MD result	1.74	17.01	15.26	688
339	monDom5ChrX 26180546-26181233 L1Mdo3 result	4.36	13.81	9.45	688
340	monDom5ChrX 34152684-34153371 HAL1 Opos result	1.89	14.68	12.79	688
341	monDom5ChrX 66255764-66256451 L1 Mars1b result	6.83	13.37	6.54	688
342	monDom5ChrX 14335422-14336115 L1 Opos1 result	6.63	18.01	11.38	694
343	monDom5ChrX 18650948-18651644 L1 Mdo5 result	3.30	12.20	8.90	697
344	monDom5ChrX 7608065-7608765 L1Mdo4 result	1.00	32.38	31.38	701
345	monDom5ChrX 40706835-40707538 L1 Opos0 result	2.56	16.62	14.06	704
346	monDom5ChrX 35922314-35923020 L1 Opos result	1.56	12.59	11.03	707
347	monDom5ChrX 24087564-24088271 L1 Mdo3b result	4.10	13.84	9.75	708
348	monDom5ChrX 39435273-39435980 L1 Mars1b result	2.12	19.49	17.37	708
349	monDom5ChrX 66377332-66378039 L1 Mars1b result	3.39	14.55	11.16	708
350	monDom5ChrX 78197435-78198145 HAL1 Opos result	1.41	13.36	11.95	711
351	monDom5ChrX 50362231-50362943 HAL1 Opos result	4.91	12.06	7.15	713
352	monDom5ChrX 67883535-67884247 L1 Mars1 result	2.38	14.45	12.06	713
353	monDom5ChrX 25108378-25109091 L1 Mdo3b result	3.36	17.09	13.73	714
354	monDom5ChrX 25937829-25938542 L1 Mdo3b result	3.64	17.23	13.59	714
355	monDom5ChrX 14638321-14639035 L1 Opos result	1.82	22.38	20.56	715
356	monDom5ChrX 25187158-25187872 L1 Mdo3b result	3.36	15.52	12.17	715
357	monDom5ChrX 47097175-47097892 L1 Mdo2 result	3.20	14.35	11.14	718
358	monDom5ChrX 76134469-76135188 L1 Opos0 result	3.75	14.44	10.69	720
359	monDom5ChrX 11657920-11658645 HAL1 Opos result	3.99	18.60	14.60	726
360	monDom5ChrX 16127495-16128220 L1 Mdo4 result	2.20	17.63	15.43	726
361	monDom5ChrX 35472281-35473013 L1 Mdo2 result	3.14	19.24	16.10	733
362	monDom5ChrX 4421238-4421971 L1 Opos0 result	2.18	14.99	12.81	734
363	monDom5ChrX 63749972-63750706 L1 Mars1b result	0.82	13.47	12.65	735
364	monDom5ChrX 73941038-73941775 L1 Mdo3c result	0.81	20.33	19.51	738
365	monDom5ChrX 52149516-52150256 L1 Mdo3b result	1.48	14.71	13.23	741
366	monDom5ChrX 26032029-26032770 L1-1 MD result	2.16	15.63	13.48	742
367	monDom5ChrX 39811083-39811824 L1 Mdo5 result	1.75	19.54	17.79	742
368	monDom5ChrX 79202870-79203612 L1 Opos2 result	3.23	15.21	11.98	743
369	monDom5ChrX 71937805-71938549 L1 Opos0 result	0.00	18.12	18.12	745

168	mm10ChrX 165220475-165220830 L1 Ro	10.96	7.87	-3.09	356
169	mm10ChrX 113690318-113690674 L1 Mu	0.00	12.04	12.04	357
170	mm10ChrX 127460532-127460891 Lx7 r	1.67	14.44	12.78	360
171	mm10ChrX 95392808-95393170 L1M2 re	3.31	12.12	8.82	363
172	mm10ChrX 106039582-106039946 Lx5c	7.67	15.34	7.67	365
173	mm10ChrX 83786330-83786694 Lx resu	0.00	6.85	6.85	365
174	mm10ChrX 64306472-64306839 Lx4a re	4.08	10.33	6.25	368
175	mm10ChrX 97875435-97875807 Lx8b re	5.90	7.77	1.88	373
176	mm10ChrX 113999429-113999803 Lx8 r	4.27	16.53	12.27	375
177	mm10ChrX 59009063-59009437 Lx2 res	0.00	16.00	16.00	375
178	mm10ChrX 137697072-137697449 Lx6 r	2.91	11.38	8.47	378
179	mm10ChrX 94222494-94222872 Lx5 res	5.28	6.86	1.58	379
180	mm10ChrX 147310701-147311081 L1Md	0.00	9.71	9.71	381
181	mm10ChrX 44819188-44819578 Lx9 res	6.91	13.30	6.39	391
182	mm10ChrX 13621895-13622289 L1 Mus1	1.27	10.63	9.37	395
183	mm10ChrX 72364547-72364941 L1Md F2	3.29	16.96	13.67	395
184	mm10ChrX 135319162-135319558 L1 Mu	0.00	11.08	11.08	397
185	mm10ChrX 46389223-46389620 L1 Mus2	2.51	11.06	8.54	398
186	mm10ChrX 70998506-70998905 Lz9 res	0.00	21.75	21.75	400
187	mm10ChrX 96481186-96481585 L1MA5A	2.75	13.75	11.00	400
188	mm10ChrX 123088596-123088998 Lx6 r	6.70	16.63	9.93	403
189	mm10ChrX 21633038-21633440 L1MB2 r	1.24	12.66	11.41	403
190	mm10ChrX 23456943-23457349 Lx5 res	1.23	16.46	15.23	407
191	mm10ChrX 115991711-115992119 Lx6 r	8.31	8.80	0.49	409
192	mm10ChrX 166634458-166634866 L1Md	1.47	7.33	5.87	409
193	mm10ChrX 34131149-34131558 L1VL4 r	2.93	20.73	17.80	410
194	mm10ChrX 60101199-60101609 L1Md F2	4.14	12.17	8.03	411
195	mm10ChrX 160938498-160938909 Lx4B	1.70	9.95	8.25	412
196	mm10ChrX 40215983-40216395 L1Md F2	3.39	10.90	7.51	413
197	mm10ChrX 127258893-127259309 Lx5c	1.68	18.23	16.55	417
198	mm10ChrX 82997158-82997575 L1Md F3	1.44	8.85	7.42	418
199	mm10ChrX 74185867-74186286 L1 Mur1	2.62	12.14	9.52	420
200	mm10ChrX 39956699-39957122 L1Md F2	3.77	14.62	10.85	424
201	mm10ChrX 86973015-86973438 Lx7 res	10.61	9.20	-1.42	424
202	mm10ChrX 70629380-70629805 Lx8 res	1.41	8.22	6.81	426
203	mm10ChrX 18790808-18791234 L1MDb r	1.17	12.65	11.48	427
204	mm10ChrX 105655922-105656349 Lx4A	3.27	14.49	11.21	428
205	mm10ChrX 133510345-133510772 L1 Mu	1.17	24.30	23.13	428
206	mm10ChrX 114836915-114837348 Lx9 r	0.00	8.76	8.76	434
207	mm10ChrX 167608718-167609153 Lx2B2	0.00	18.35	18.35	436
208	mm10ChrX 64455457-64455895 Lx4B re	4.33	12.98	8.66	439
209	mm10ChrX 14554477-14554918 L1ME3A	5.88	8.82	2.94	442
210	mm10ChrX 73725336-737257781MB7 res	4.06	11.74	7.67	443
211	mm10ChrX 49400445-49400889 Lx8 res	1.12	8.99	7.87	445
212	mm10ChrX 25655521-25655969 L1 Mur2	4.01	10.02	6.01	449
213	mm10ChrX 50269378-50269827 Lx5 res	1.33	19.33	18.00	450
214	mm10ChrX 115355384-115355834 Lx3C	5.54	13.97	8.43	451
215	mm10ChrX 107273118-107273569 Lx8b	3.10	13.72	10.62	452
216	mm10ChrX 129673145-129673598 Lx re	2.42	17.84	15.42	454
217	mm10ChrX 152835904-152836357 L1 Mu	2.42	11.89	9.47	454
218	mm10ChrX 121045857-121046316 L1MA4	1.09	5.00	3.91	460
219	mm10ChrX 59306299-59306758 L1 Mus1	3.91	10.87	6.96	460
220	mm10ChrX 119087860-119088322 L1Md	5.62	12.31	6.70	463
221	mm10ChrX 54124389-54124851 L1MEj r	5.18	14.25	9.07	463
222	mm10ChrX 98178119-98178585 Lx9 res	3.85	8.35	4.50	467
223	mm10ChrX 84306100-84306571 L1 Mus2	4.45	15.89	11.44	472
224	mm10ChrX 53924199-53924671 L1M2 re	4.86	8.67	3.81	473
225	mm10ChrX 125926230-125926703 L1Md	6.54	13.92	7.38	474
226	mm10ChrX 169074967-169075446 Lx8 r	0.00	9.17	9.17	480
227	mm10ChrX 84762963-84763444 L1Md F3	1.24	11.83	10.58	482
228	mm10ChrX 49880254-49880738 L1 Mus1	2.27	11.75	9.48	485
229	mm10ChrX 91103941-91104428 HAL1 re	3.07	19.26	16.19	488
230	mm10ChrX 117748049-117748539 L1M2	2.04	13.65	11.61	491
231	mm10ChrX 36584796-36585286 L1 Mus1	1.22	11.41	10.18	491
232	mm10ChrX 138176487-138176978 L1MC3	5.08	15.85	10.77	492
233	mm10ChrX 89351807-89352299 L1Md A	1.22	7.30	6.09	493
234	mm10ChrX 136019568-136020064 L1M2	1.01	9.46	8.45	497
235	mm10ChrX 88238741-88239237 L1MB7 r	3.22	8.85	5.63	497
236	mm10ChrX 124439851-124440350 Lx2B	1.00	15.80	14.80	500
237	mm10ChrX 111853410-111853911 L1M2	2.39	8.37	5.98	502
238	mm10ChrX 131781811-131782312 L1Md	2.59	6.57	3.98	502
239	mm10ChrX 169548902-169549404 Lx6 r	3.18	5.96	2.78	503
240	mm10ChrX 167240610-167241113 Lx5b	3.17	23.41	20.24	504
241	mm10ChrX 167192650-167193155 L1 Mu	1.19	16.40	15.22	506
242	mm10ChrX 61956606-61957112 L1Md F2	2.17	10.65	8.48	507
243	mm10ChrX 148192151-148192660 Lx re	0.98	13.73	12.75	510
244	mm10ChrX 87560912-87561422 Lx8b re	6.26	10.76	4.50	511
245	mm10ChrX 115070047-115070560 Lx9 r	6.42	10.70	4.28	514
246	mm10ChrX 100340202-100340716 Lx8 r	4.08	16.89	12.82	515
247	mm10ChrX 57518413-57518931 Lx8 res	2.12	12.33	10.21	519
248	mm10ChrX 32454419-32454940 Lx4A re	1.34	17.62	16.28	522
249	mm10ChrX 16846928-16847453 Lx6 res	2.28	19.39	17.11	526
250	mm10ChrX 34247352-34247877 L1Md Gf	2.85	22.43	19.58	526
251	mm10ChrX 116810642-116811168 L1 Mu	2.85	8.16	5.31	527
252	mm10ChrX 57535577-57536112 L1ME2z	5.04	7.65	2.61	536
253	mm10ChrX 131989766-131990302 L1 Mu	4.10	16.95	12.85	537
254	mm10ChrX 148986877-148987414 L1 Mu	0.93	12.08	11.15	538
255	mm10ChrX 3832517-3833054 Lx4A resu	2.97	13.57	10.59	538
256	mm10ChrX 42460941-42461480 L1Md F2	3.33	15.00	11.67	540
257	mm10ChrX 97763894-97764434 Lx7 res	10.72	8.13	-2.59	541
258	mm10ChrX 46461967-46462508 L1Md F2	1.11	11.25	10.15	542
259	mm10ChrX 19310150-19310692 L1MA7 r	0.00	14.18	14.18	543
260	mm10ChrX 35779353-35779896 Lx5c re	7.17	16.36	9.19	544
261	mm10ChrX 126639947-126640492 L1 Mu	2.56	19.60	17.03	546
262	mm10ChrX 155502970-155503516 L1M2	2.01	12.98	10.97	547
263	mm10ChrX 78137548-78138095 L1 Rod	4.93	13.14	8.21	548
264	mm10ChrX 151706424-151706972 L1 Mu	4.01	16.39	12.39	549
265	mm10ChrX 45506673-45507221 L1 Mur3	2.91	23.50	20.58	549
266	mm10ChrX 91096401-91096950 L1 Mur2	1.09	15.82	14.73	550
267	mm10ChrX 127514438-127514989 L1MA4	2.17	13.04	10.87	552
268	mm10ChrX 102944561-102945113 L1VL2	1.81	11.21	9.40	553
269	mm10ChrX 92826179-92826732 L1MA6 r	1.08	11.19	10.11	554
270	mm10ChrX 33348837-33349391 Lx4A re	2.88	15.50	12.61	555
271	mm10ChrX 61539809-61540363 Lx2B2 r	3.06	13.33	10.27	555
272	mm10ChrX 114203401-114203956 L1 Mu	0.00	17.81	17.81	556
273	mm10ChrX 79157721-79158279 L1 Mus2	2.15	13.77	11.63	559
274	mm10ChrX 92045691-92046251 L1 Mus1	1.07	13.55	12.48	561
275	mm10ChrX 67800342-67800904 Lx6 res	7.46	8.53	1.07	563
276	mm10ChrX 151129358-151129922 Lx6 r	10.80	10.09	-0.71	565
277	mm10ChrX 23244026-23244602 L1Md F2	1.04	11.96	10.92	577
278	mm10ChrX 68418636-68419212 L1 Mus2	1.91	14.73	12.82	577
279	mm10ChrX 95366294-95366873 L1M3 re	1.72	14.83	13.10	580
280	mm10ChrX 52334860-52335440 L1MB8 r	3.61	12.91	9.29	581
281	mm10ChrX 67214350-67214931 L1 Mus3	7.04	13.06	6.01	582
282	mm10ChrX 51833082-51833664 L1 Mus2	1.89	18.01	16.12	583
283	mm10ChrX 110181984-110182579 Lx5 r	2.01	10.91	8.89	596
284	mm10ChrX 78380207-78380804 L1Md F2	11.20	2.17	-9.03	598
285	mm10ChrX 11013600-11014198 L1MB2 r	1.17	9.68	8.51	599
286	mm10ChrX 33241095-33241697 L1VL4 r	1.00	15.92	14.93	603
287	mm10ChrX 155654877-155655480 L1 Ro	2.65	9.93	7.28	604
288	mm10ChrX 121779563-121780170 L1 Mu	2.80	13.98	11.18	608
289	mm10ChrX 132592942-132593549 Lx re	2.14	16.61	14.47	608
290	mm10ChrX 28043135-28043743 L1Md F2	3.61	11.17	7.55	609
291	mm10ChrX 154706416-154707026 Lx re	1.80	20.95	19.15	611
292	mm10ChrX 147482433-147483044 L1Md	2.45	15.36	12.91	612
293	mm10ChrX 80729838-80730452 Lx3 Mus	2.93	15.45	12.52	615
294	mm10ChrX 116475436-116476052 Lx8 r	8.59	9.56	0.97	617
295	mm10ChrX 132391814-132392430 L1MEg	1.94	14.91	12.97	617
296	mm10ChrX 49865558-49866176 L1VL2 r	3.55	10.02	6.46	619
297	mm10ChrX 116765767-116766388 L1 Mu	1.77	19.29	17.52	622
298	mm10CHrX 75556945-75557575 Lx resu	3.49	11.25	7.77	631
299	mm10ChrX 136020538-136021172 Lx7 r	13.86	7.40	-6.46	635
300	mm10ChrX 137493376-137494013 L1 Mu	2.98	19.75	16.77	638
301	mm10ChrX 163226350-163226990 Lx6 r	1.72	12.64	10.92	641
302	mm10ChrX 55058003-55058645 L1VL1 r	3.27	21.46	18.20	643
303	mm10ChrX 111393990-111394633 L1MEc	2.17	11.65	9.47	644
304	mm10ChrX 133366277-133366921 L1 Mu	2.17	15.04	12.87	645
305	mm10ChrX 151383622-151384269 Lx3 M	1.70	13.73	12.04	648
306	mm10ChrX 113635876-113636567 Lx8 r	6.46	6.00	-0.46	650
307	mm10ChrX 153049197-153049848 Lx8 r	2.45	10.28	7.82	652
308	mm10ChrX 132995843-132996495 Lx6 r	9.95	10.26	0.31	653
309	mm10ChrX 63747775-63748427 Lx3C re	4.44	10.26	5.82	653
310	mm10ChrX 46859390-46860043 L1Md F2	1.99	14.68	12.69	654
311	mm10ChrX 146774987-146775644 Lx2 r	2.43	12.01	9.57	658
312	mm10ChrX 91703550-91704208 L1 Mus1	2.12	14.87	12.75	659
313	mm10ChrX 135861703-135862362 Lx10	2.73	15.30	12.58	660
314	mm10ChrX 76532224-76532883 L1VL4 r	5.45	7.88	2.42	660
315	mm10ChrX 46156662-46157325 MusHAL1	3.61	15.81	12.20	664
316	mm10ChrX 66265310-66265973 L1 Mur2	0.00	15.51	15.51	664
317	mm10CHrX 121791851-121792517 Lx9 r	4.65	11.54	6.90	667
318	mm10ChrX 104021900-104022569 Lx7 r	3.58	11.34	7.76	670
319	mm10ChrX 168501355-168502026 L1Md	1.93	10.42	8.48	672
320	mm10ChrX 47765918-47766591 Lx resu	4.01	20.33	16.32	674
321	mm10ChrX 91921744-91922419 L1 Mus4	4.73	15.38	10.65	676
322	mm10ChrX 31770249-31770925 L1Md F2	1.92	11.96	10.04	677
323	mm10ChrX 82147120-82147799 L1 Mur2	1.47	14.56	13.09	680
324	mm10ChrX 45402690-45403374 Lx7 res	5.84	8.61	2.77	685
325	mm10ChrX 114319823-114320508 L1 Mr	3.06	12.54	9.48	686
326	mm10ChrX 142077683-142078370 Lx2B2	1.89	20.49	18.60	688
327	mm10ChrX 45813694-45814381 L1 Mur2	2.18	12.21	10.03	688
328	mm10ChrX 148557141-148557829 L1 Mu	1.02	13.21	12.19	689
329	mm10ChrX 25564234-25564922 L1 Mus3	4.21	15.38	11.18	689
330	mm10ChrX 95397350-95398039 L1 Mus4	2.90	18.55	15.65	690
331	mm10ChrX 46553867-46554560 Lx3 Mus	1.73	11.38	9.65	694
332	mm10ChrX 97351003-97351697 Lx9 res	3.74	7.91	4.17	695
333	mm10ChrX 155966396-155967091 L1 Mu	1.87	9.48	7.61	696
334	mm10ChrX 93515528-93516224 L1M3 re	3.30	9.33	6.03	697
335	mm10ChrX 90496506-90497203 L1 Mus3	2.72	19.48	16.76	698
336	mm10ChrX 95743041-95743740 Lx7 res	4.57	9.43	4.86	700
337	mm10ChrX 113689026-113689731 L1Md	2.69	10.91	8.22	706
338	mm10ChrX 138418467-138419173 Lx5 r	1.56	13.58	12.02	707
339	mm10ChrX 104010383-104011093 Lx3 M	2.25	10.97	8.72	711
340	mm10ChrX 100752760-100753471 Lx re	2.67	14.04	11.38	712
341	mm10ChrX 129173415-129174126 Lx7 r	6.74	9.55	2.81	712
342	mm10ChrX 4161413-4162125 L1 Mur3 r	1.68	14.31	12.62	713
343	mm10ChrX 45792939-45793651 Lx3A re	2.81	14.59	11.78	713
344	mm10ChrX 66699574-66700286 Lx7 res	8.98	7.71	-1.26	713
345	mm10ChrX 8742420-8743132 L1 Rod re	2.52	7.43	4.91	713
346	mm10ChrX 167870577-167871290 L1 Mu	2.80	13.45	10.64	714
347	mm10ChrX 67985308-67986021 Lx8 res	3.50	6.44	2.94	714
348	mm10ChrX 113785033-113785748 Lx3A	0.70	15.64	14.94	716
349	mm10ChrX 9689029-9689747 L1Md F2 r	1.81	10.57	8.76	719
350	mm10ChrX 97883683-97884403 L1 Mur3	3.05	14.15	11.10	721
351	mm10ChrX 153494272-153494993 Lx7 r	2.22	14.68	12.47	722
352	mm10ChrX 69079292-69080013 L1 Mus4	1.94	20.22	18.28	722
353	mm10ChrX 20108987-20109711 L1Md F2	4.41	11.72	7.31	725
354	mm10ChrX 34309020-34309744 L1 Mur2	1.24	13.93	12.69	725
355	mm10ChrX 56350028-56350756 Lx5 res	2.19	12.76	10.56	729
356	mm10ChrX 97750227-97750957 Lx7 res	4.51	9.30	4.79	731
357	mm10ChrX 117634831-117635562 L1Md	3.01	12.70	9.70	732
358	mm10ChrX 14890063-14890795 L1 Mus3	3.27	14.46	11.19	733
359	mm10ChrX 96140435-96141167 Lx8 res	2.18	11.46	9.28	733
360	mm10ChrX 122465643-122466377 Lx5 r	1.50	13.06	11.56	735
361	mm10ChrX 154451516-154452254 L1 Mu	3.38	12.99	9.61	739
362	mm10ChrX 67666043-67666781 L1 Mus1	2.57	13.40	10.83	739
363	mm10ChrX 139477386-139478128 Lx re	2.29	15.75	13.46	743
364	mm10ChrX 105502336-105503079 L1VL4	1.61	18.41	16.80	744
365	mm10ChrX 145199326-145200072 L1 Mu	1.87	15.13	13.25	747
366	mm10ChrX 49975618-49976364 L1MB4 r	2.68	8.17	5.49	747
367	mm10ChrX 145344105-145344853Lx4A r	2.54	10.41	7.88	749
368	mm10ChrX 112411959-112412710 Lx2B2	6.38	6.78	0.40	752
369	mm10ChrX 14153542-14154293 L1MC1 r	2.39	8.38	5.98	752

168	hg38ChrX 14990341-14990722 L1MA9 r	1.57	7.07	5.50	382
169	hg38ChrX 39993461-39993843 L1MB7 r	1.31	5.22	3.92	383
170	hg38ChrX 107047680-107048064 L1MEg	1.56	22.34	20.78	385
171	hg38ChrX 89607762-89608155 L1M5 re	4.06	10.41	6.35	394
172	hg38ChrX 135185638-135186032 L1MA6	6.33	14.68	8.35	395
173	hg38ChrX 152464932-152465331 L1MA9	0.00	9.00	9.00	400
174	hg38ChrX 82487277-82487676L1MB3 re	2.00	11.00	9.00	400
175	hg38ChrX 48451747-48452148 L1M4 re	2.74	18.41	15.67	402
176	hg38ChrX 47476368-47476773 L1MB8 r	3.20	17.00	13.79	406
177	hg38ChrX 147798032-147798439 L1MA4	2.70	6.37	3.68	408
178	hg38ChrX 143255728-143256137 L1M5	3.90	9.76	5.85	410
179	hg38ChrX 22085592-22086004 L1M3f r	2.66	6.78	4.12	413
180	hg38ChrX 73074853-73075265 L1M4 re	3.39	10.90	7.51	413
181	hg38ChrX 55594626-55595040 L1ME3G	1.20	9.64	8.43	415
182	hg38ChrX 20412420-20412836 L1PA8A	3.12	11.03	7.91	417
183	hg38ChrX 50859317-50859733 L1MB4 r	5.28	5.28	0.00	417
184	hg38ChrX 67033263-67033682 L1MA3 r	0.00	7.86	7.86	420
185	hg38ChrX 102437524-102437951 L1MC4	4.21	16.36	12.15	428
186	hg38ChrX 77803332-77803762 L1M4 re	2.78	13.92	11.14	431
187	hg38ChrX 144651845-144652276 L1ME3	1.39	10.42	9.03	432
188	hg38ChrX 76122509-76122941 L1MD2 r	1.15	8.78	7.62	433
189	hg38ChrX 34922128-34922564 L1MEg1	3.20	11.21	8.01	437
190	hg38ChrX 137670890-137671327 L1PA1	1.37	6.16	4.79	438
191	hg38ChrX 139545919-139546360 L1PA1	2.26	13.57	11.31	442
192	hg38ChrX 98144927-98145369 L1PA16	1.35	8.80	7.45	443
193	hg38ChrX 46502963-46503406 L1MEi r	5.86	21.17	15.32	444
194	hg38ChrX 67865902-67866346 L1PA17	4.72	11.24	6.52	445
195	hg38ChrX 147127955-147128404 L1MC3	1.11	15.33	14.22	450
196	hg38ChrX 68974839-68975288 L1MC1 r	5.56	11.33	5.78	450
197	hg38ChrX 134279865-134280315 L1MD	2.22	13.30	11.09	451
198	hg38ChrX 139568548-139569001 L1MA2	0.00	7.27	7.27	454
199	hg38ChrX 28759408-28759863 L1MC5a	0.00	10.75	10.75	456
200	hg38ChrX 17993734-17994193 L1ME4a	3.04	2.39	-0.65	460
201	hg38ChrX 27732020-27732480 L1MA4 r	2.39	13.02	10.63	461
202	hg38ChrX 84590836-84591300 L1PREC2	4.95	7.96	3.01	465
203	hg38ChrX 140556364-140556832 L1MA4	2.13	10.23	8.10	469
204	hg38ChrX 72852441-72852910 L1MC5 r	8.51	10.64	2.13	470
205	hg38ChrX 117798326-117798797 L1PA7	2.75	7.63	4.87	472
206	hg38ChrX 78013616-78014089 L1ME4c	2.53	11.60	9.07	474
207	hg38ChrX 107269727-107270201 L1M4	5.26	15.58	10.32	475
208	hg38ChrX 14216014-14216488 L1MC5a	0.00	11.37	11.37	475
209	hg38ChrX 78874220-78874696 L1PA10	4.61	7.97	3.35	477
210	hg38ChrX 56156915-56157394 L1PA5 r	3.13	10.21	7.08	480
211	hg38ChrX 89442794-89443273 L1MA3 r	0.00	8.54	8.54	480
212	hg38ChrX 86751747-86752228 L1PA16	2.49	7.26	4.77	482
213	hg38ChrX 94151827-94152308 L1PA6 r	0.00	11.83	11.83	482
214	hg38ChrX 12059463-12059946 L1PA16	1.24	6.40	5.17	484
215	hg38ChrX 17112322-17112806 L1MDb r	4.12	14.85	10.72	485
216	hg38ChrX 44943542-44944026 L1ME1 r	0.00	3.92	3.92	485
217	hg38ChrX 152092813-152093299 L1ME1	2.87	9.45	6.57	487
218	hg38ChrX 41570386-41570877 L1ME1 r	0.00	6.10	6.10	492
219	hg38ChrX 54528126-54528619 L1ME3E	1.01	11.34	10.32	494
220	hg38ChrX 141270276-141270772 L1MA9	1.41	7.04	5.63	497
221	hg38ChrX 43033633-43034129 L1MB7 r	1.61	7.44	5.84	497
222	hg38ChrX 31696096-31696594 L1ME3A	5.21	8.02	2.81	499
223	hg38ChrX 76347130-76347629 L1M4a1	1.40	15.00	13.60	500
224	hg38ChrX 144261476-144261976 L1MA4	2.40	6.99	4.59	501
225	hg38ChrX 99919694-99920197 L1ME3F	0.99	10.52	9.52	504
226	hg38ChrX 21347472-21347977 L1MDa r	1.19	18.77	17.59	506
227	hg38ChrX 56666744-56667249 L1PREC2	2.77	9.09	6.32	506
228	hg38ChrX 95313653-95314162 L1PB4 r	1.18	7.45	6.27	510
229	hg38ChrX 114159360-114159872 L1M7	2.14	10.33	8.19	513
230	hg38ChrX 129024646-129025159 L1M4	0.97	15.95	14.98	514
231	hg38ChrX 34003653-34004170 L1PA16	3.47	9.07	5.60	518
232	hg38ChrX 107687771-107688292 L1MC4	1.53	13.41	11.88	522
233	hg38ChrX 2980047-132980573 L1PA15	2.09	11.20	9.11	527
234	hg38ChrX 87705995-87706526 L1M2 re	4.14	10.90	6.77	532
235	hg38ChrX 134608122-134608654 L1MB5	3.56	5.07	1.50	533
236	hg38ChrX 101789457-101789991 L1MEf	4.86	11.96	7.10	535
237	hg38ChrX 10974054-10974594 L1ME3 r	5.91	7.76	1.85	541
238	hg38ChrX 20460829-20461371 L1ME1 r	1.10	13.08	11.97	543
239	hg38ChrX 47974866-47975408 L1ME4b	2.21	7.92	5.71	543
240	hg38ChrX 108103305-108103848 L1MB1	4.41	13.60	9.19	544
241	hg38ChrX 84512702-84513246 L1MA4 r	1.83	10.64	8.81	545
242	hg38ChrX 31672778-31673323 L1MB7 r	7.51	5.31	-2.20	546
243	hg38ChrX 63386972-63387518 L1ME1 r	5.48	7.13	1.65	547
244	hg38ChrX 87238574-87239123 L1M1 re	5.27	12.73	7.45	550
245	hg38ChrX 51884544-51885096 L1MD2 r	4.70	12.84	8.14	553
246	hg38ChrX 24161053-24161606 L1M5 re	3.97	9.57	5.60	554
247	hg38ChrX 126581347-126581901 L1MB3	1.44	6.67	5.23	555
248	hg38ChrX 146339966-146340520 HAL1	3.24	16.04	12.79	555
249	hg38ChrX 154260403-154260960 L1M8	0.00	8.96	8.96	558
250	hg38ChrX 29270296-29270853 L1ME1 r	4.12	7.53	3.41	558
251	hg38ChrX 4561262-4561821 L1MA8 res	0.00	7.32	7.32	560
252	hg38ChrX 117141782-117142342 L1MB7	1.07	7.49	6.42	561
253	hg38ChrX 77573527-77574088 L1ME1 r	1.07	6.41	5.34	562
254	hg38ChrX 78088280-78088844 L1M1 re	1.95	15.75	13.81	565
255	hg38ChrX 135715721-135716290 L1PA7	2.28	11.40	9.12	570
256	hg38ChrX 123316173-123316748 L1ME4	3.47	9.55	6.08	576
257	hg38ChrX 99002107-99002682 L1MCc r	1.91	11.28	9.38	576
258	hg38ChrX 127947912-127948489 L1M4	1.90	20.24	18.34	578
259	hg38ChrX 6893695-6894277 L1PA11 re	1.89	9.95	8.06	583
260	hg38ChrX 101200456-101201039 L1ME3	0.00	11.30	11.30	584
261	hg38ChrX 82058357-82058940 L1ME1 r	1.88	5.65	3.77	584
262	hg38ChrX 30761721-30762305 L1PREC2	2.91	8.72	5.81	585
263	hg38ChrX 67761102-67761688 L1PA3 r	1.02	8.86	7.84	587
264	hg38ChrX 56514358-56514945 L1MA3 r	1.02	7.31	6.29	588
265	hg38ChrX 27195096-27195684 L1MA4A	0.85	10.19	9.34	589
266	hg38ChrX 66776417-66777005 L1MA1 r	2.55	14.26	11.71	589
267	hg38ChrX 38592772-38593364 L1MEc r	3.71	13.32	9.61	593
268	hg38ChrX 87236726-87237320 L1M1 re	3.53	15.46	11.93	595
269	hg38ChrX 133768969-133769565 L1MA1	0.84	13.74	12.90	597
270	hg38ChrX 48505793-48506389 L1MC4 r	3.85	10.05	6.20	597
271	hg38ChrX 50550089-50550686 L1MB8 r	1.00	10.54	9.53	598
272	hg38ChrX 27678268-27678866 L1MB3 r	1.84	9.02	7.18	599
273	hg38ChrX 132537381-132537980 L1M5	8.33	11.17	2.83	600
274	hg38ChrX 63968769-63969369 L1MCa r	0.00	16.97	16.97	601
275	hg38ChrX 11059281-11059882 L1ME2 r	1.83	8.64	6.81	602
276	hg38ChrX 135139481-135140083 L1MC	5.47	12.11	6.63	603
277	hg38ChrX 138995315-138995917 L1PA1	1.82	11.28	9.45	603
278	hg38ChrX 116095138-116095741 L1M1	0.99	14.57	13.58	604
279	hg38ChrX 100537150-100537756 L1PA6	1.81	7.74	5.93	607
280	hg38ChrX 79430093-79430699 L1MA10	4.28	11.70	7.41	607
281	hg38ChrX 25148914-25149522 L1MA1 r	3.61	12.97	9.36	609
282	hg38ChrX 22986088-22986699 L1MB8 r	1.14	15.36	14.22	612
283	hg38ChrX 92816723-92817339 L1MB5 r	0.97	8.10	7.13	617
284	hg38ChrX 8784551-8785170 L1PB1 res	4.84	8.23	3.39	620
285	hg38ChrX 116616366-116616988 L1PA5	1.93	8.35	6.42	623
286	hg38ChrX 16399547-16400169 L1MA2 r	0.00	11.24	11.24	623
287	hg38ChrX 120396131-120396756 L1MEc	3.51	11.66	8.15	626
288	hg38ChrX 51661849-51662475 L1MC2 r	2.55	13.24	10.69	627
289	hg38ChrX 81041461-81042091 L1MA4A	1.11	7.61	6.50	631
290	hg38ChrX 105448665-105449297 L1MA2	1.58	15.96	14.38	633
291	hg38ChrX 116060465-116061098 L1M1	9.15	12.15	3.00	634
292	hg38ChrX 80946343-80946976 L1PA15	1.74	11.36	9.62	634
293	hg38ChrX 81010984-81011619 L1MEc r	3.62	13.21	9.59	636
294	hg38ChrX 101571310-101571951 L1MEg	3.12	11.21	8.10	642
295	hg38ChrX 120680300-120680941 L1MA9	3.58	16.20	12.62	642
296	hg38ChrX 127200091-127200732 L1PRE	4.52	11.99	7.48	642
297	hg38ChrX 150424725-150425368 L1MB8	2.64	9.78	7.14	644
298	hg38ChrX 27110408-27111055 L1P1 re	3.40	17.13	13.73	648
299	hg38ChrX 54883829-54884478 L1ME1 r	1.69	11.54	9.85	650
300	hg38ChrX 80900864-80901518L1PREC2	1.83	21.98	20.15	655
301	hg38ChrX 52842573-52843228 L1MCc r	0.00	14.33	14.33	656
302	hg38ChrX 130374056-130374712 L1MC1	2.74	8.07	5.33	657
303	hg38ChrX 125989641-125990303 L1M4	1.51	6.64	5.13	663
304	hg38ChrX 51462481-51463143 L1PA4 r	1.81	10.71	8.90	663
305	hg38ChrX 121707990-121708655 L1MEf	3.60	9.61	6.01	666
306	hg38ChrX 83968811-83969478 L1PA10	4.49	6.44	1.95	668
307	hg38ChrX 90166349-90167016 L1PA14	3.44	10.03	6.59	668
308	hg38ChrX 143785370-143786041 L1ME3	2.83	10.27	7.44	672
309	hg38ChrX 4140688-4141359 L1MB3 res	1.64	9.82	8.18	672
310	hg38ChrX 127148068-127148742 L1ME1	1.48	11.11	9.63	675
311	hg38ChrX 131336880-131337557 L1ME1	0.88	7.82	6.93	678
312	hg38ChrX 83001210-83001888 L1PA4 r	1.91	10.75	8.84	679
313	hg38ChrX 114205438-114206121 L1MEc	3.22	9.21	5.99	684
314	hg38ChrX 41993114-41993799 L1MB8 r	0.87	14.58	13.70	686
315	hg38ChrX 54488614-54489300 L1PA15	1.16	12.95	11.79	687
316	hg38ChrX 5157182-5157870 L1ME2 res	4.93	14.80	9.87	689
317	hg38ChrX 90042585-90043276 PA16 re	1.88	8.96	7.08	692
318	hg38ChrX 98457496-98458187 L1MA8 r	2.60	11.27	8.67	692
319	hg38ChrX 124330687-124331381 L1MB4	0.86	8.92	8.06	695
320	hg38ChrX 140776604-140777299 L1PA8	1.58	9.34	7.76	696
321	hg38ChrX 18541820-18542517 L1MD2 r	3.30	15.19	11.89	698
322	hg38ChrX 102308996-102309694 L1ME1	2.15	7.87	5.72	699
323	hg38ChrX 45566498-45567198 L1MA3 r	0.86	9.70	8.84	701
324	hg38ChrX 145444641-145445342 L1ME2	2.56	13.39	10.83	702
325	hg38ChrX 32841265-32841966 L1M5 re	2.71	8.12	5.41	702
326	hg38ChrX 32399854-32400557 L1P1 re	4.69	9.66	4.97	704
327	hg38ChrX 70953256-70953959 L1MB7 r	3.69	10.37	6.68	704
328	hg38ChrX 20789600-20790306 L1ME4b	5.09	9.34	4.24	707
329	hg38ChrX 85820286-85820992 L1PREC2	3.54	13.01	9.48	707
330	hg38ChrX 47445434-47446144 L1ME3A	2.25	4.64	2.39	711
331	hg38ChrX 119369589-119370300 L1ME3	1.54	14.33	12.78	712
332	hg38ChrX 35484291-35485002 L1PA13	0.84	9.41	8.57	712
333	hg38ChrX 154101803-154102515 L1MA6	0.84	13.18	12.34	713
334	hg38ChrX 94814153-94814866 L1PA13	1.40	4.76	3.36	714
335	hg38ChrX 96099787-96100501 L1ME1 r	2.52	10.07	7.55	715
336	hg38ChrX 113026708-113027425 L1MA2	0.70	11.98	11.28	718
337	hg38ChrX 139273346-139274065 L1MA8	1.81	11.67	9.86	720
338	hg38ChrX 148141630-148142352 L1PA1	1.66	9.27	7.61	723
339	hg38ChrX 20606086-20606810 L1MA8 r	0.69	8.00	7.31	725
340	hg38ChrX 44081741-44082469 L1MC5 r	2.47	14.27	11.80	729
341	hg38ChrX 27733157-27733889 L1MA4 r	2.46	9.41	6.96	733
342	hg38ChrX 76719275-76720009 L1PREC2	2.31	11.56	9.25	735
343	hg38ChrX 52468645-52469382 L1M5 re	1.63	8.67	7.05	738
344	hg38ChrX 84196442-84197180 L1MA2 r	1.62	9.34	7.71	739
345	hg38ChrX 64876507-64877246 L1MA2 r	4.05	9.73	5.68	740
346	hg38ChrX 113118082-113118822 L1MC5	2.83	10.39	7.56	741
347	hg38ChrX 128993930-128994672 L1MA4	1.48	8.34	6.86	743
348	hg38ChrX 80196649-80197391 L1MA9 r	4.85	13.19	8.34	743
349	hg38ChrX 32854638-32855384 L1MA3 r	2.28	9.50	7.23	747
350	hg38ChrX 96756165-96756911 L1MB8 r	2.68	10.17	7.50	747
351	hg38ChrX 74211025-74211772 L1ME1 r	0.94	10.29	9.36	748
352	hg38ChrX 138634646-138635395 L1PB2	1.33	4.93	3.60	750
353	hg38ChrX 91300624-91301374 L1M3 re	1.73	10.39	8.66	751
354	hg38ChrX 83853056-83853807 L1M3b r	1.99	8.78	6.78	752
355	hg38ChrX 2774888-2775640 L1MB5 res	0.93	9.03	8.10	753
356	hg38ChrX 21445307-21446063 L1MA5 r	0.66	12.81	12.15	757
357	hg38ChrX 34308322-34309079 L1MB3 r	2.11	9.89	7.78	758
358	hg38ChrX 151906089-151906852 L1M5	0.65	10.47	9.82	764
359	hg38ChrX 90506531-90507294 L1MA1 r	3.40	23.30	19.90	764
360	hg38ChrX 35985142-35985907 L1PA13	5.22	4.18	-1.04	766
361	hg38ChrX 47072641-47073409 L1PA2 r	1.56	9.62	8.06	769
362	hg38ChrX 69330110-69330878 L1MA4 r	3.38	13.65	10.27	769
363	hg38ChrX 121045853-121046624 L1M3	1.42	13.08	11.66	772
364	hg38ChrX 69376614-69377387 L1M4 re	3.49	14.21	10.72	774
365	hg38ChrX 117649844-117650622 L1MA2	1.41	8.60	7.19	779
366	hg38ChrX 33732454-33733232 L1ME3 r	0.00	7.32	7.32	779
367	hg38ChrX 72402763-72403545L1MB7 re	0.77	10.86	10.09	783
368	hg38ChrX 135602292-135603077 L1PA1	3.31	8.52	5.22	786
369	hg38ChrX 93687527-93688314 L1MA6 r	1.27	13.83	12.56	788

370	monDom5ChrX 40890223-40890969 L1 Mars1 result	6.83	17.00	10.17	747
371	monDom5ChrX 70255694-70256440 L1 Mdo3a result	1.74	15.26	13.52	747
372	monDom5ChrX 59634965-59635715 L1 Mdo6 result	0.00	12.65	12.65	751
373	monDom5ChrX 26706101-26706853 L1 Mdo5 result	0.66	13.55	12.88	753
374	monDom5ChrX 28775493-28776247 L1Mdo3c result	3.44	16.42	12.98	755
375	monDom5ChrX 50244663-50245417 L1 Mdo5 result	3.58	9.40	5.83	755
376	monDom5ChrX 77904138-77904892 L1 Opos2 result	3.31	11.13	7.81	755
377	monDom5 30815513-30816271 HAL1 Opos result	2.11	18.45	16.34	759
378	monDom5ChrX 28837177-28837935 L1 Mdo6 result	1.98	14.23	12.25	759
379	monDom5ChrX 78218286-78219044 HAL1 Opos result	2.24	15.28	13.04	759
380	monDom5ChrX 16366383-16367144 L1 Opos1 result	3.41	13.39	9.97	762
381	monDom5ChrX 35943673-35944441 L1 Opos1 result	4.03	13.65	9.62	769
382	monDom5ChrX 51456275-51457045 L1 Mdo3a result	0.91	11.15	10.25	771
383	monDom5ChrX 56262814-56263585 L1 Mdo1 result	2.07	19.69	17.62	772
384	monDom5ChrX 4556510-4557286 L1 Opos result	2.06	19.69	17.63	777
385	monDom5ChrX 12860018-12860797 L1 Mdo4 result	2.95	16.54	13.59	780
386	monDom5ChrX 10832043-10832823 L1 Opos result	2.43	14.72	12.29	781
387	monDom5ChrX 77052480-77053260 L1 Opos2 result	2.18	16.52	14.34	781
388	monDom5ChrX 27704674-27705457 L1 Mdo3a result	0.00	29.46	29.46	784
389	monDom5ChrX 59339538-59340321 L1 Mars1a result	4.97	11.73	6.76	784
390	monDom5ChrX 51364409-51365199 L1 Mars1 result	5.44	10.62	5.18	791
391	monDom5ChrX 66751641-66752432 HAL1 Opos result	1.89	14.27	12.37	792
392	monDom5ChrX 29500003-29500796 L1 Opos result	2.27	14.86	12.59	794
393	monDom5ChrX 21355191-21355985 L1 Mdo1 result	2.01	23.14	21.13	795
394	monDom5ChrX 35145826-35146625 L1 Mdo3a result	1.50	18.63	17.13	800
395	monDom5ChrX 41959376-41960175 L1 Mdo5 result	5.13	7.88	2.75	800
396	monDom5ChrX 39813812-39814614 L1 Mdo6 result	2.37	12.95	10.59	803
397	monDom5ChrX 14354425-14355229 L1 Mdo4 result	0.62	25.84	25.22	805
398	monDom5ChrX 64233926-64234733 L1Mdo5 result	2.97	12.25	9.28	808
399	monDom5ChrX 33815184-33816002 L1 Mdo3b result	0.61	13.43	12.82	819
400	monDom5ChrX 53354677-53355496 L1 Mars1b result	4.63	15.24	10.61	820
401	monDom5ChrX 69365768-69366592 L1 Opos1 result	4.12	15.76	11.64	825
402	monDom5ChrX 76681489-76682314 L1 Mdo4 result	1.21	19.49	18.28	826
403	monDom5ChrX 62825771-62826601 L1Opos2 result	1.81	12.76	10.95	831
404	monDom5ChrX 70946944-70947774 L1 Opos result	3.85	17.45	13.60	831
405	monDom5ChrX 36247441-36248275 L1 Mars1b result	2.16	15.45	13.29	835
406	monDom5ChrX 61311970-61312805 L1 Mdo3a result	1.56	18.78	17.22	836
407	monDom5ChrX 55150835-55151675 L1 Mdo5 result	1.66	17.12	15.46	841
408	monDom5ChrX 55599081-55599923 HAL1 Opos result	4.63	16.96	12.34	843
409	monDom5ChrX 76105190-76106037 L1 Mdo3 result	2.83	10.97	8.14	848
410	monDom5ChrX 13778103-13778952 L1 Mars1b result	4.35	19.65	15.29	850
411	monDom5ChrX 7719177-7720029 L1 Mdo3b result	2.11	18.41	16.30	853
412	monDom5ChrX 28470603-28471461 L1-1 MD result	1.86	18.16	16.30	859
413	monDom5ChrX 59090980-59091838 L1 Opos0 result	3.84	12.34	8.50	859
414	monDom5ChrX 25384411-25385271 L1 Opos result	3.02	17.19	14.17	861
415	monDom5ChrX 38748942-38749804 L1 Mdo4 result	1.97	17.61	15.64	863
416	monDom5ChrX 58631380-58632243 HAL1N1 MD result	3.59	16.44	12.85	864
417	monDom5ChrX 19875884-19876748 L1 Opos0 result	1.62	14.45	12.83	865
418	monDom5ChrX 74315208-74316072 L1 Mars1 result	3.35	15.14	11.79	865
419	monDom5ChrX 50132734-50133603 L1 Opos result	5.98	12.87	6.90	870
420	monDom5ChrX 64638772-64639642 L1 Opos4b result	2.64	17.68	15.04	871
421	monDom5ChrX 59990343-59991214 L1 Mars1 result	0.69	13.99	13.30	872
422	monDom5ChrX 64179819-64180691 L1 Mdo4 result	2.06	21.42	19.36	873
423	monDom5ChrX 77793813-77794685 L1 Opos result	6.19	16.72	10.54	873
424	monDom5ChrX 64187219-64188092 L1 Opos0 result	1.72	14.87	13.16	874
425	monDom5ChrX 25226682-25227559 L1 Opos result	1.37	17.43	16.06	878
426	monDom5ChrX 18283154-18284033 L1 Mdo5 result	1.48	15.91	14.43	880
427	monDom5ChrX 22602281-22603162 L1 Opos0 result	3.51	13.72	10.20	882
428	monDom5ChrX 52758097-52758978 L1 Mdo3a result	3.29	16.89	13.61	882
429	monDom5ChrX 61716723-61717605 HAL1 Opos1 result	3.62	12.80	9.17	883
430	monDom5ChrX 52462335-52463218 L1 Opos4b result	2.83	18.21	15.38	884
431	monDom5ChrX 59115555-59116442 L1 Mars1 result	1.80	16.55	14.75	888
432	monDom5ChrX 73539181-73540069 L1 Opos2 result	4.61	13.84	9.22	889
433	monDom5ChrX 59083313-59084202 L1 Mdo4 result	1.57	14.04	12.47	890
434	monDom5ChrX 38299984-38300874 L1 Opos3 result	1.91	12.46	10.55	891
435	monDom5ChrX 30106753-30107644 L1 Opos0 result	4.48	14.80	10.31	892
436	monDom5ChrX 12637092-12637985 L1 Mars1 result	3.80	18.90	15.10	894
437	monDom5ChrX 21778069-21778963 L1 Mdo4 result	0.00	28.94	28.94	895
438	monDom5ChrX 70659009-70659903 L1 Opos result	2.68	16.31	13.63	895
439	monDom5ChrX 37546163-37547058 L1 Opos1 result	3.35	16.52	13.17	896
440	monDom5ChrX 56988875-56989770 L1 Mars1 result	1.12	21.32	20.20	896
441	monDom5ChrX 74359906-74360801 L1 Opos result	4.80	16.85	12.05	896
442	monDom5ChrX 30602056-30602952 L1 Mdo3b result	2.68	17.06	14.38	897
443	monDom5ChrX 59073021-59073919 L1 Mdo4 result	2.22	16.24	14.02	899
444	monDom5ChrX 12571227-12572126 L1 Mdo3c result	2.33	13.22	10.89	900
445	monDom5ChrX 50144196-50145095 L1 Mdo4 result	2.00	14.33	12.33	900
446	monDom5ChrX 31575395-31576295 L1 do3a result	2.77	16.43	13.65	901
447	monDom5ChrX 36355447-36356347 L1 Opos result	3.33	13.76	10.43	901
448	monDom5ChrX 70508983-70509884 L1 Opos result	1.44	15.41	13.97	902
449	monDom5ChrX 12062148-12063052 L1 Opos2 result	2.76	17.13	14.36	905
450	monDom5ChrX 24521263-24522168 L1 Mdo3a result	1.43	18.21	16.78	906
451	monDom5ChrX 41658114-41659020 HAL1 Opos1 result	4.30	14.77	10.47	907
452	monDom5ChrX 19599380-19600288 L1 Opos3 result	0.55	13.09	12.54	909
453	monDom5ChrX 22931377-22932296 HAL1 Opos1 result	1.41	12.50	11.09	920
454	monDom5ChrX 69277501-69278420 L1 Mdo3a result	2.17	14.57	12.39	920
455	monDom5ChrX 58365584-58366508 L1 Mdo3b result	1.95	16.97	15.03	925
456	monDom5ChrX 37765164-37766089 L1 Opos0 result	0.65	15.01	14.36	926
457	monDom5ChrX 51868133-51869058 L1 Mdo3b result	1.40	17.93	16.52	926
458	monDom5ChrX 23015371-23016297 L1 Opos1 result	2.37	19.53	17.15	927
459	monDom5ChrX 29192765-29193691 L1 Mdo3b result	1.40	17.48	16.07	927
460	monDom5ChrX 24434023-24434957 L1 Opos2 result	4.81	12.41	7.59	935
461	monDom5ChrX 36187185-36188119 L1 Opos1 result	2.03	13.48	11.44	935
462	monDom5ChrX 52732465-52733400 L1 Mdo3b result	3.21	19.12	15.92	936
463	monDom5ChrX 59315996-59316931 L1 Mdo5 result	1.50	14.42	12.93	936
464	monDom5ChrX 79201153-79202090 L1 Opos2 result	2.13	13.75	11.62	938
465	monDom5ChrX 32992482-32993425 L1 Opos result	3.50	19.39	15.89	944
466	monDom5ChrX4885173-4886116 L1 Mdo3a result	1.80	15.89	14.09	944
467	monDom5ChrX 28988362-28989307 L1 Mdo2 result	2.85	19.66	16.81	946
468	monDom5ChrX 67196226-67197174 L1 Mdo3a result	2.00	18.34	16.33	949
469	monDom5ChrX 42874790-42875741 L1 Opos1 result	2.21	15.44	13.24	952
470	monDom5ChrX 78318421-78319374 L1 Opos0 result	4.40	17.61	13.21	954
471	monDom5ChrX 15016037-15016992 L1 Mdo3a result	2.82	20.50	17.68	956
472	monDom5ChrX 24564369-24565325 L1 Mdo1 result	1.57	19.75	18.18	957
473	monDom5ChrX 62847824-62848781 L1 Opos0 result	2.92	22.13	19.21	958
474	monDom5ChrX 72383734-72384691 L1 Mdo5 result	7.20	12.11	4.91	958
475	monDom5ChrX 22327197-22328156 L1 Opos1 result	3.65	14.69	11.04	960
476	monDom5ChrX 23989714-23990675 L1 Mars1 result	2.49	19.96	17.46	962
477	monDom5ChrX 3008944-3009906 L1 Opos2 result	2.28	11.42	9.14	963
478	monDom5ChrX 38611811-38612712 HAL1N1 MD result	2.47	24.95	22.47	970
479	monDom5ChrX 44484719-44485691 L1 Opos1 result	4.32	18.29	13.98	973
480	monDom5ChrX 24370673-24371653 L1 Opos0 result	3.77	21.81	18.04	981
481	monDom5ChrX 12175404-12176386 L1 Mdo3b result	2.75	15.36	12.61	983
482	monDom5ChrX 56549619-56550601 L1 Mdo2 result	2.75	20.55	17.80	983
483	monDom5ChrX 45806979-45807963 L1 Mars1b result	4.06	12.89	8.83	985
484	monDom5ChrX 12107639-12108624 L1 Mdo5 result	1.52	20.18	18.66	986
485	monDom5ChrX 35412615-35413600 L1 Mdo3c result	1.72	14.71	12.98	986
486	monDom5ChrX 47934827-47935813 HAL1N1 MD result	2.33	16.31	13.98	987
487	monDom5ChrX 45346166-45347153 L1 Mars1 result	2.94	23.08	20.14	988
488	monDom5ChrX 60713428-60714417 L1 Opos0 result	3.33	10.20	6.87	990
489	monDom5ChrX 46263168-46264159 L1 Mdo3c result	2.22	15.93	13.71	992
490	monDom5ChrX 74960860-74961852 L1 Mdo3c result	2.42	16.92	14.50	993
491	monDom5ChrX 66551038-66552032 L1 Mars1b result	3.32	15.88	12.56	995
492	monDom5ChrX 38886585-38887582 L1 Opos1 result	6.01	10.22	4.21	998
493	monDom5ChrX 34830049-34831047 L1 Mars1a result	0.50	17.62	17.12	999
494	monDom5ChrX 10800120-10801122 HAL1 Opos result	3.29	19.94	16.65	1003
495	monDom5ChrX 58141134-58142152 L1 Mdo3a result	2.55	17.17	14.62	1019
496	monDom5ChrX 13010376-13011395 L1 Opos4b result	3.92	16.86	12.94	1020
497	monDom5ChrX 25832811-25833851 resultL1 Mdo3	2.21	30.45	28.24	1041
498	monDom5ChrX 25869181-25870227 L1 Mdo4 result	4.58	17.57	12.99	1047
499	monDom5ChrX 10609815-10610864 L1 Mars1 result	7.05	16.19	9.14	1050
500	monDom5ChrX 52579240-52580310 HAL1N1 MD result	4.86	22.60	17.74	1071
501	monDom5ChrX 6932099-6933207 HAL1N1 MD result	5.05	15.06	10.01	1109
502	monDom5ChrX 48668016-48669127 L1 Mdo3c result	1.08	19.24	18.17	1112
503	monDom5ChrX 18151770-18152907 L1 Opos4b result	1.05	16.26	15.20	1138
504	monDom5ChrX 36451507-36452652 L1 Opos2 result	2.53	12.04	9.51	1146
505	monDom5ChrX 23970682-23971831 L1 Mdo5 result	3.30	19.30	16.00	1150
506	monDom5ChrX 53272747-53273904 L1 Opos2 result	5.44	17.44	12.00	1158
507	monDom5ChrX 6172811-6173970 L1 Mdo2 result	2.33	17.50	15.17	1160
508	monDom5ChrX 76007504-76008669 L1 Mdo3c result	2.32	15.52	13.21	1166
509	monDom5ChrX 70803663-70804842 L1 Mdo3c result	1.44	17.46	16.02	1180
510	monDom5ChrX 40153524-40154709 L1 Mdo3 result	0.93	23.44	22.51	1186
511	monDom5ChrX 34904180-34905389 L1 Opos0 result	2.23	13.39	11.16	1210
512	monDom5ChrX 41213070-41214349 L1 Opos1 result	4.69	12.73	8.05	1280
513	monDom5ChrX 22438184-22439465 L1 Mdo1 result	1.40	15.13	13.73	1282
514	monDom5ChrX 61063848-61065129 HAL1 Opos result	2.81	12.56	9.75	1282
515	monDom5ChrX 67621243-67622528 L1 Opos result	3.97	16.72	12.75	1286
516	monDom5ChrX 29184237-29185542 L1 Mdo3c result	0.38	16.46	16.08	1306
517	monDom5ChrX 54627289-54628617 L1 Opos result	2.41	17.76	15.35	1329
518	monDom5ChrX 30315709-30317048 L1 Mdo4 result	0.82	25.82	25.00	1340
519	monDom5ChrX 61162047-61163387 L1 Mdo6 result	2.46	14.99	12.53	1341
520	monDom5ChrX 51905697-51907063 L1 Opos0 result	2.63	16.17	13.53	1367
521	monDom5ChrX 19600356-19601737 L1 Opos4b result	1.95	15.77	13.82	1382
522	monDom5ChrX 43413998-43415405 L1 Mdo2 result	2.27	18.39	16.12	1408
523	monDom5ChrX 22816587-22818001 L1 Opos0 result	1.70	16.11	14.42	1415
524	monDom5ChrX 44743227-44744643 L1 Opos2 result	3.67	13.90	10.23	1417
525	monDom5ChrX 28869459-28870898 L1 Mdo4 result	2.15	20.63	18.47	1440
526	monDom5ChrX 5026819-5028264 L1 Mdo1 result	1.52	19.16	17.63	1446
527	monDom5ChrX 19849657-19851134 L1 Mdo5 result	2.37	21.72	19.35	1478
528	monDom5ChrX 47684697-47686189 L1 Mdo5 result	2.61	16.88	14.27	1493
529	monDom5ChrX 3476549-3478062 L1 Mdo4 result	2.05	22.52	20.48	1514
530	monDom5ChrX 22518264-22519778 L1 Mdo3 result	3.04	21.98	18.94	1515
531	monDom5ChrX 74725169-74726692 L1 Mdo3a result	1.90	16.67	14.76	1524
532	monDom5ChrX 4624095-4625770 L1-1 MD result	1.73	16.23	14.50	1676
533	monDom5ChrX 57263017-57264701 HAL1 Opos result	3.44	14.48	11.04	1685
534	monDom5ChrX 49490193-49491881 L1 Opos0 result	3.73	16.93	13.20	1689
535	monDom5ChrX 4291936-4293652 L1 Mdo3c result	0.93	17.47	16.54	1717
536	monDom5ChrX 50231753-50233490 L1 Mdo6 result	1.67	26.75	25.09	1738
537	monDom5ChrX 19188460-19190216 L1 Mdo5 result	0.91	14.46	13.55	1757
538	monDom5ChrX 6196048-6197810 L1 Mars1 result	5.22	17.98	12.76	1763
539	monDom5ChrX 65459604-65461420 L1 Mdo3c result	1.21	16.13	14.91	1817
540	monDom5ChrX 3712483-3714311 L1 Mdo3c result	1.48	15.58	14.11	1829
541	monDom5ChrX 54114717-54116611 L1 Mdo3c result	1.58	16.73	15.15	1895
542	monDom5ChrX 16375640-16377535 L1 Mdo3c result	0.95	15.61	14.66	1896
543	monDom5ChrX 62386252-62388178 L1 Mdo3c result	1.09	27.24	26.15	1927
544	monDom5ChrX 72711397-72713407 L1 Opos2 result	2.54	22.43	19.89	2011
545	monDom5ChrX 34909785-34911820 L1 Mdo3 result	1.62	15.91	14.29	2036
546	monDom5ChrX 61005674-61007710 L1 Mdo2 result	2.21	15.95	13.75	2037
547	monDom5ChrX 39280877-39282933 L1 Mars1 result	4.28	14.34	10.06	2057
548	monDom5ChrX 4288081-4290142 L1 Mdo4 result	2.57	20.85	18.28	2062
549	monDom5ChrX 60011689-60013753 L1 Mdo3b result	2.66	15.88	13.22	2065
550	monDom5ChrX 9640979-9643050 L1 Opos0 result	2.65	16.12	13.47	2072
551	monDom5ChrX 32997925-33000000 L1 Mdo3c result	1.30	16.96	15.66	2076
552	monDom5ChrX 53585723-53587810 L1 Mdo1	2.25	16.57	14.32	2088
553	monDom5ChrX 41176060-41178148 L1 Mars1b result	2.44	25.61	23.17	2089
554	monDom5ChrX 69328947-69331051 L1 Mdo2 result	1.90	17.01	15.11	2105
555	monDom5ChrX 51232773-51234908 L1 Mdo3c result	2.57	26.97	24.39	2136
556	monDom5ChrX 62305575-62307722 HAL1N1 MD result	4.00	18.06	14.06	2148
557	monDom5ChrX 21889431-21891591 L1 Mdo2 result	2.50	15.73	13.23	2161
558	monDom5ChrX 61059871-61062046 HAL1N1 MD result	2.67	20.40	17.74	2176
559	monDom5ChrX 76611800-76613975 L1 Mdo4 result	1.88	25.05	23.16	2176
560	monDom5ChrX 27705485-27707729 L1 Mdo3a result	1.96	21.60	19.64	2245
561	monDom5ChrX 64217422-64219674 L1 Mdo3a result	2.35	16.20	13.85	2253
562	monDom5ChrX 3755304-3757565 L1 Mdo1 result	2.12	17.42	15.30	2262
563	monDom5ChrX 38750082-38752389 L1 Mdo1 result	1.43	14.38	12.95	2308
564	monDom5ChrX 49262289-49264596 L1 Mdo3c result	1.69	15.03	13.34	2308
565	monDom5ChrX 41531751-41534061 L1 Mdo3c result	1.77	17.14	15.36	2311
566	monDom5ChrX 17345109-17347431 HAL1N1 MD result	3.23	20.75	17.52	2323
567	monDom5ChrX 59805370-59807710 L1 Mdo1 result	1.84	14.91	13.07	2341
568	monDom5ChrX 20653466-20655819 L1 Mdo3c result	3.65	14.95	11.30	2354
569	monDom5ChrX 3752922-3755306 L1 Mdo1 result	2.81	19.12	16.31	2385
570	monDom5ChrX 73508111-73510502 L1 Mdo4 result	1.51	22.03	20.53	2392
571	monDom5ChrX 44621614-44624014 L1 Mdo5 result	2.33	13.49	11.16	2401

370	mm10ChrX 89624997-89625755 L1 Mus2	3.29	15.28	11.99	759
371	mm10ChrX 19935283-19936045 L1VL4 r	4.06	19.53	15.47	763
372	mm10ChrX 33110160-33110924 L1Md F2	5.10	13.07	7.97	765
373	mm10ChrX 45937830-45938594 L1 Mus3	1.83	15.69	13.86	765
374	mm10ChrX 107182109-107182878 Lx8b	3.77	11.04	7.27	770
375	mm10ChrX 56443730-56444501 Lx6 res	4.02	11.14	7.12	772
376	mm10ChrX 111548658-111549430 Lx3B	1.55	15.91	14.36	773
377	mm10ChrX 58355860-58356633 Lx3C re	3.10	11.50	8.40	774
378	mm10ChrX 160008636-160009414 L1Md	0.00	15.66	15.66	779
379	mm10ChrX 112596893-112597675 L1Md	2.30	10.73	8.43	783
380	mm10ChrX 69627216-69628001 L1Md F2	2.93	9.80	6.87	786
381	mm10ChrX 117053805-117054591 Lx5b	2.92	10.55	7.62	787
382	mm10ChrX 134823566-134824353 L1 Mu	3.93	13.45	9.52	788
383	mm10ChrX 45618453-45619241 Lx2 res	0.00	18.38	18.38	789
384	mm10ChrX 9918342-9919131 L1VL4 res	16.58	13.67	-2.91	790
385	mm10ChrX 28732065-28732860 Lx3C re	3.14	14.32	11.18	796
386	mm10ChrX 107126594-107127394 L1 Mu	3.75	14.73	10.99	801
387	mm10ChrX 40223575-40224376 Lx7 res	2.00	12.72	10.72	802
388	mm10ChrX 133080167-133080969 L1 Mu	4.11	14.32	10.21	803
389	mm10ChrX 68965182-68965984 Lx6 res	3.24	16.81	13.57	803
390	mm10ChrX 95395594-95396396 L1VL4 r	5.60	14.57	8.97	803
391	mm10ChrX 47520416-47521220 L1Md A	2.24	11.43	9.19	805
392	mm10ChrX 70704491-70705296 L1 Mus1	3.10	15.38	12.28	806
393	mm10ChrX 40360777-40361583 Lx3A re	1.36	17.72	16.36	807
394	mm10ChrX 110986731-110987539 Lx5c	4.45	13.60	9.15	809
395	mm10ChrX 119898323-119899138 L1 Mu	1.35	14.83	13.48	816
396	mm10ChrX 167626029-167626845 Lx4B	2.57	12.48	9.91	817
397	mm10ChrX 24455431-24456248 Lx2 res	2.93	18.83	15.89	818
398	mm10ChrX 144954170-144954995 L1Md	2.18	10.05	7.87	826
399	mm10ChrX 67911764-67912590 L1Md F2	0.00	20.31	20.31	827
400	mm10ChrX 84867932-84868759 Lx5 res	1.81	16.06	14.25	828
401	mm10ChrX 97006697-97007525 L1 Mus4	2.41	14.84	12.42	829
402	mm10ChrX 146561322-146562153 L1M2	3.25	10.94	7.69	832
403	mm10ChrX 29368524-29369356 Lx resu	3.24	11.28	8.04	833
404	mm10ChrX 150673089-150673922 L1 Mu	4.56	15.59	11.03	834
405	mm10ChrX 45598581-45599414 L1Md F2	2.16	12.71	10.55	834
406	mm10ChrX 44234018-44234854 L1 Md F	2.15	10.27	8.12	837
407	mm10ChrX 68049783-68050620 Lx6 res	2.63	8.00	5.37	838
408	mm10ChrX 81766780-81767617 L1 Mur1	2.27	16.95	14.68	838
409	mm10ChrX 126500001-126500840 L1Md	2.86	11.31	8.45	840
410	mm10ChrX 54448677-54449522 Lx resu	3.66	13.12	9.46	846
411	mm10ChrX 62963328-62964175 Lx3A re	2.24	10.97	8.73	848
412	mm10ChrX 165714372-165715226 L1 Mu	4.68	13.80	9.12	855
413	mm10ChrX 105455708-105456565 Lx re	1.98	16.90	14.92	858
414	mm10ChrX 150046500-150047357 Lx5 r	3.15	12.35	9.21	858
415	mm10ChrX 16368868-16369726 L1 Mus1	3.03	14.09	11.06	859
416	mm10ChrX 59608461-59609319 L1 Rod	2.33	14.09	11.76	859
417	mm10ChrX 49137280-49138139 L1MDa r	3.37	8.84	5.47	860
418	mm10ChrX 91956602-91957461 L1Md F3	3.84	12.79	8.95	860
419	mm10ChrX 46833132-46833994 Lx5b re	2.43	16.34	13.90	863
420	mm10ChrX 155025323-155026193 Lx6 r	3.10	9.99	6.89	871
421	mm10ChrX 162604710-162605580 Lx7 r	2.30	11.83	9.53	871
422	mm10ChrX 23395473-23396350 L1MA5 r	2.39	8.66	6.26	878
423	mm10ChrX 120775607-120776485 Lx5c	4.78	5.92	1.14	879
424	mm10ChrX 9976186-9977066 L1 Mur1 r	3.52	12.94	9.42	881
425	mm10ChrX 8871240-8872123 Lx4B resu	2.60	12.67	10.07	884
426	mm10ChrX 124932378-124933264 L1Md	1.58	11.27	9.70	887
427	mm10ChrX 161007383-161008270 Lx5 r	0.68	12.50	11.82	888
428	mm10ChrX 32307130-32308018 L1 Mur2	3.26	15.75	12.49	889
429	mm10ChrX 6102667-6103558 L1Md F3 r	2.02	13.34	11.32	892
430	mm10ChrX 108936632-108937525 L1 Mu	3.13	11.52	8.39	894
431	mm10ChrX 38286273-38287166 Lx5 res	2.80	11.19	8.39	894
432	mm10ChrX 68094312-68095205 L1 Mur3	1.23	8.72	7.49	894
433	mm10ChrX 146891026-146891921 Lx re	1.67	16.41	14.73	896
434	mm10ChrX 123871380-123872278 L1Md	1.56	11.90	10.34	899
435	mm10ChrX 158021245-158022143 Lx8 r	2.00	11.01	9.01	899
436	mm10ChrX 82792847-82793746 L1Md T	3.67	13.56	9.89	900
437	mm10ChrX 129200255-129201164 L1VL4	1.43	19.56	18.13	910
438	mm10ChrX 50107524-50108437 L1Md F2	2.52	13.13	10.61	914
439	mm10ChrX 104596510-104597427 L1 Mu	2.51	14.71	12.20	918
440	mm10ChrX 91778476-91779394 L1 Mus2	3.48	15.78	12.30	919
441	mm10ChrX 105217676-105218596 L1 Ro	3.15	12.49	9.34	921
442	mm10ChrX 146666244-146667169 Lx2B	5.08	13.71	8.64	926
443	mm10ChrX 15593964-15594889 L1 Mur3	11.45	8.10	-3.35	926
444	mm10ChrX 57051726-57052655 L1Md F2	1.94	13.33	11.40	930
445	mm10ChrX 111934432-111935363 L1 Mu	2.68	15.56	12.88	932
446	mm10ChrX 92725077-92726013 L1 Mus3	2.56	13.98	11.42	937
447	mm10ChrX 14394601-14395538 L1MA8 r	2.77	16.74	13.97	938
448	mm10ChrX 5946183-5947120 Lx9 resul	1.17	20.58	19.40	938
449	mm10ChrX 162203473-162204414 L1Md	1.91	11.78	9.87	942
450	mm10ChrX 19948648-19949590 Lx5 res	4.35	10.92	6.57	943
451	mm10ChrX 75443657-75444601 Lx resu	2.43	14.29	11.85	945
452	mm10ChrX 62590478-62591447 Lx resu	1.86	13.61	11.75	970
453	mm10ChrX 50681252-50682224 L1Md F3	2.67	12.44	9.76	973
454	mm10ChrX 23659460-23660433 L1Md F2	3.39	13.14	9.75	974
455	mm10ChrX 109220451-109221426 L1Md	2.46	12.19	9.73	976
456	mm10ChrX 84466833-84467808 Lx3A re	1.13	13.83	12.70	976
457	mm10ChrX 42833271-42834247 Lx8 res	6.04	8.90	2.87	977
458	mm10ChrX 39810958-39811935 L1Md T	3.27	11.55	8.28	978
459	mm10ChrX 24181073-24182052 L1Md F2	1.84	13.37	11.53	980
460	mm10ChrX 128382139-128383119 L1Md	2.65	10.09	7.44	981
461	mm10ChrX 103134751-103135732 L1Md	1.83	14.46	12.63	982
462	mm10ChrX 23980294-23981276 L1Md A	2.64	11.39	8.75	983
463	mm10ChrX 26282886-26283868 L1Md F2	3.56	12.72	9.16	983
464	mm10ChrX 88434141-88435123 L1Md F3	3.76	14.14	10.38	983
465	mm10ChrX 9545550-9546534 L1 Md F2	2.64	12.59	9.95	985
466	mm10ChrX 130895269-130896255 L1 Md	2.63	13.27	10.64	987
467	mm10ChrX 55016290-55017276 L1Md A	2.63	11.75	9.12	987
468	mm10ChrX 135160353-135161340 L1Md	1.82	11.64	9.82	988
469	mm10ChrX 50522887-50523875 L1Md F2	2.12	10.82	8.70	989
470	mm10ChrX 108714695-108715684 L1Md	3.43	11.11	7.68	990
471	mm10ChrX 67660308-67661297 L1Md F2	2.63	10.71	8.08	990
472	mm10ChrX 158899249-158900239 L1Md	3.13	13.52	10.39	991
473	mm10ChrX 108329296-108330288 L1Md	3.22	11.48	8.26	993
474	mm10ChrX 133309185-133310177 L1Md	3.73	12.59	8.86	993
475	mm10ChrX 147211837-147212829 L1Md	3.22	11.78	8.56	993
476	mm10ChrX 25544251-25545243 L1Md F2	2.72	12.69	9.97	993
477	mm10ChrX 36722290-36723284 L1Md F2	2.71	12.76	10.05	995
478	mm10ChrX 162674536-162675531 L1Md	2.61	11.75	9.14	996
479	mm10ChrX 16445783-16446778 L1Md A	2.61	11.75	9.14	996
480	mm10ChrX 165402933-165403928 L1 Mu	2.71	13.45	10.74	996
481	mm10ChrX 169441998-169442993 L1Md	2.61	11.75	9.14	996
482	mm10ChrX 67784744-67785739 L1Md T	2.11	11.85	9.74	996
483	mm10ChrX 72585481-72586476 L1Md T	3.21	12.25	9.04	996
484	mm10ChrX 94814903-94815898 L1 Mur1	3.21	12.45	9.24	996
485	mm10ChrX 116937076-116938074 L1Md	4.60	15.82	11.21	999
486	mm10ChrX 82128126-82129132 L1 Mus3	2.88	10.72	7.85	1007
487	mm10ChrX 117088188-117089202 L1Md	9.36	3.45	-5.91	1015
488	mm10ChrX 94573974-94574990 L1Md A	3.83	17.80	13.96	1017
489	mm10ChrX 107143172-107144189 Lx8b	5.21	10.31	5.11	1018
490	mm10ChrX 38722912-38723929 Lx8 res	5.89	10.12	4.22	1018
491	mm10ChrX 92991182-92992208 L1Md F2	2.14	13.15	11.00	1027
492	mm10ChrX 6281590-6282618 L1 Mur3 r	2.53	12.15	9.62	1029
493	mm10ChrX 155543547-155544577 Lx5b	2.13	15.03	12.90	1031
494	mm10ChrX 17426040-17427081 L1Md F2	4.32	14.49	10.17	1042
495	mm10ChrX 118249931-118250975 Lx3B	1.63	12.73	11.10	1045
496	mm10ChrX 46477877-46478925 Lx8 res	4.96	9.34	4.39	1049
497	mm10ChrX 64007715-64008768 L1 Mur2	1.71	15.09	13.38	1054
498	mm10ChrX 93592707-93593775 Lx6 res	5.71	9.64	3.93	1069
499	mm10ChrX 89683584-89684691 L1 Mus3	3.79	13.54	9.75	1108
500	mm10ChrX 94667887-94669007 L1 Mur3	2.23	10.70	8.47	1121
501	mm10ChrX 103168409-103169530 L1Md	3.74	13.73	9.98	1122
502	mm10ChrX 72063737-72064867 Lx7 res	2.39	14.94	12.56	1131
503	mm10ChrX 16217492-16218633 L1 Mus3	3.24	10.60	7.36	1142
504	mm10ChrX 80701208-80702362 Lx3B re	3.72	11.00	7.27	1155
505	mm10ChrX 59996439-59997627 L1 Mus4	2.44	14.97	12.53	1189
506	mm10ChrX 79064969-79066170 Lx2 res	0.83	9.57	8.74	1202
507	mm10ChrX 87450116-87451320 Lx5 res	2.82	11.29	8.46	1205
508	mm10ChrX 70180270-70181478 L1 Mur2	1.16	22.99	21.84	1209
509	mm10ChrX 129108855-129110116 L1 Mu	3.57	14.50	10.94	1262
510	mm10ChrX 90555541-90556826 L1 Mur2	1.24	13.69	12.44	1286
511	mm10ChrX 68855530-68856829 L1 Mus2	4.46	13.31	8.85	1300
512	mm10ChrX 109275154-109276457 L1 Mu	3.99	13.04	9.05	1304
513	mm10ChrX 119023788-119025097 L1 Mu	3.51	12.52	9.01	1310
514	mm10ChrX 105698409-105699759 L1Md	2.74	12.66	9.92	1351
515	mm10ChrX 128015639-128017000 Lx5c	3.16	11.01	7.86	1362
516	mm10ChrX 16169702-16171065 Lx6 res	6.16	8.14	1.98	1364
517	mm10ChrX 125352244-125353639 L1Md	1.79	17.48	15.69	1396
518	mm10ChrX 20440497-20441935 L1Md F2	4.17	12.51	8.34	1439
519	mm10ChrX 45383828-45385363 L1Md F2	4.04	12.76	8.72	1536
520	mm10ChrX 97539734-97541269 L1Md T	3.78	12.04	8.27	1536
521	mm10ChrX 44651816-44653365 Lx2 res	2.52	11.23	8.71	1550
522	mm10ChrX 80020603-80022167 L1Md F2	1.92	15.78	13.87	1565
523	mm10ChrX 131802053-131803660 L1Md	2.86	12.87	10.01	1608
524	mm10ChrX 10049945-10051560 L1VL2 r	2.35	18.75	16.40	1616
525	mm10ChrX 65311221-65312842 Lx7 res	1.36	11.96	10.60	1622
526	mm10ChrX 104582696-104584347 Lx9 r	3.33	11.99	8.66	1652
527	mm10ChrX 147083507-147085205 L1 Mu	3.77	13.95	10.18	1699
528	mm10ChrX 142493710-142495448 Lx2B2	2.36	13.92	11.56	1739
529	mm10ChrX 107018527-107020272 Lx3A	4.47	18.27	13.80	1746
530	mm10ChrX 114155430-114157206 L1 Mm	3.71	13.11	9.40	1777
531	mm10ChrX 139043714-139045510 L1 Mu	3.45	14.80	11.35	1797
532	mm10ChrX 82542454-82544258 LI Mur3	4.60	12.30	7.70	1805
533	mm10ChrX 66054186-66056023 L1 Mus2	3.26	12.30	9.03	1838
534	mm10ChrX 17468327-17470192 L1 Mus2	3.70	13.99	10.29	1866
535	mm10ChrX 91943783-91945656 L1Md F2	3.20	13.34	10.14	1874
536	mm10ChrX 154531498-154533506 L1Fd	3.19	13.04	9.86	2009
537	mm10ChrX 95428716-95430727 L1 Mm r	3.53	13.72	10.19	2012
538	mm10ChrX 18095464-18097476 L1 Mus3	2.78	15.70	12.92	2013
539	mm10ChrX 93133435-93135464 L1 Mus3	3.15	18.28	15.12	2030
540	mm10ChrX 100066873-100068913 L1 Mu	3.53	15.09	11.56	2041
541	mm10ChrX 80202421-80204504 L1 Mus2	4.27	12.48	8.21	2084
542	mm10ChrX 124161411-124163503 L1Md	2.05	17.96	15.91	2093
543	mm10ChrX 15949631-15951724 L1Md F2	3.15	13.13	9.98	2094
544	mm10ChrX 116365285-116367381 L1 Mu	5.10	13.45	8.35	2097
545	mm10ChrX 128348950-128351046 L1 Mu	2.91	13.88	10.97	2097
546	mm10ChrX 95501836-95503984 Lx3 Mus	4.00	13.36	9.35	2149
547	mm10ChrX 33279683-33281839 L1Md F	2.13	16.69	14.56	2157
548	mm10ChrX 156493474-156495678 L1 Mu	3.17	12.24	9.07	2205
549	mm10ChrX 18052652-18054871 L1Md F	1.80	17.39	15.59	2220
550	mm10ChrX 129226488-129228741 L1VL2	6.30	9.01	2.71	2254
551	mm10ChrX 14106918-14109201 Lx5c re	2.98	16.37	13.40	2284
552	mm10ChrX 127625387-127627682 Lx3A	3.09	16.03	12.94	2296
553	mm10ChrX 22974740-22977036 L1VL2 r	3.09	15.72	12.63	2297
554	mm10ChrX 3399555-3401876 L1Md F2 r	2.67	15.89	13.22	2322
555	mm10ChrX 121987813-121990142 Lx3A	0.94	16.65	15.71	2330
556	mm10ChrX 8487400-8489768 L1Md T re	4.09	13.68	9.58	2369
557	mm10ChrX 64633372-64635743 L1 Mus4	2.82	13.95	11.13	2372
558	mm10ChrX 165421578-165423975 L1 Mu	3.59	13.76	10.18	2398
559	mm10ChrX 121360046-121362460 L1 Mu	3.52	13.37	9.86	2415
560	mm10ChrX 76529381-76531809 L1VL4 r	1.81	16.76	14.94	2429
561	mm10ChrX 160665417-160667846 L1 Mu	1.81	12.59	10.78	2430
562	mm10ChrX 121754340-121756776 Lx2B2	2.09	15.76	13.66	2437
563	mm10ChrX 41083523-41085960 Lx2A re	3.28	14.44	11.16	2438
564	mm10ChrX 77286362-77288816 Lx7 res	3.18	10.47	7.29	2455
565	mm10ChrX 69585307-69587775 Lx2 res	2.88	13.49	10.61	2469
566	mm10ChrX 88778053-88780542 L1 Mus3	2.53	10.80	8.27	2490
567	mm10ChrX 83137246-83139777 L1 Mur3	2.65	11.02	8.37	2532
568	mm10ChrX 93210027-93212646 Lx2B2 r	2.63	14.16	11.53	2620
569	mm10ChrX 58332710-58335340 L1Md F2	3.65	14.29	10.64	2631
570	mm10ChrX 101495361-101498022 L1Md	3.57	14.05	10.48	2662
571	mm10ChrX 26917944-26920630 L1VL2 r	3.05	14.77	11.72	2687

370	hg38ChrX 47471632-47472420 L1ME3A	1.14	13.31	12.17	789
371	hg38ChrX 111704064-111704854 L1MB8	1.26	6.70	5.44	791
372	hg38ChrX 98231560-98232358 L1PREC2	3.13	17.65	14.52	799
373	hg38ChrX 63975242-63976041 L1MCa r	2.75	18.88	16.13	800
374	hg38ChrX 37831994-37832794 L1ME3Cz	2.12	11.49	9.36	801
375	hg38ChrX 36642018-36642822 L1MA4A	1.24	10.31	9.07	805
376	hg38ChrX 81322323-81323127 L1MA3 r	2.48	11.18	8.70	805
377	hg38ChrX 131976433-131977242 L1PA1	1.48	10.37	8.89	810
378	hg38ChrX 77561737-77562546 L1ME1 r	1.36	4.57	3.21	810
379	hg38ChrX 100088001-100088813 L1ME3	1.35	12.30	10.95	813
380	hg38ChrX 63048984-63049800 L1P1 re	3.43	12.48	9.06	817
381	hg38ChrX 66669891-66670707 L1ME3A	3.18	10.89	7.71	817
382	hg38ChrX 7452394-7453211 L1PA4 res	1.59	13.69	12.10	818
383	hg38ChrX 127759171-127759990 L1PA7	2.32	11.46	9.15	820
384	hg38ChrX 152125801-152126620 L1M5	0.61	8.17	7.56	820
385	hg38ChrX 88259952-88260771 L1MB5 r	1.59	11.22	9.63	820
386	hg38ChrX 3997846-3998669 L1PA17 re	3.03	6.19	3.16	824
387	hg38ChrX 144155558-144156388 L1MA2	0.60	14.08	13.48	831
388	hg38ChrX 151193615-151194445 L1MA3	0.60	10.11	9.51	831
389	hg38ChrX 144707973-144708808 L1MEc	3.59	12.32	8.73	836
390	hg38ChrX 79058579-79059419 L1P1 re	1.31	18.31	17.00	841
391	hg38ChrX 145888342-145889183 L1PB4	2.38	10.10	7.72	842
392	hg38ChrX 107035479-107036321 L1HS	1.42	10.32	8.90	843
393	hg38ChrX 66787813-66788655 L1MDa r	2.49	17.44	14.95	843
394	hg38ChrX 125626716-125627562 L1PB3	5.90	5.79	-0.12	847
395	hg38ChrX 137603100-137603946 L1M1	2.24	20.78	18.54	847
396	hg38ChrX 35899592-35900439 L1MD2 r	4.83	7.43	2.59	848
397	hg38ChrX 131495315-131496163 L1MEd	1.30	11.43	10.13	849
398	hg38ChrX 148061872-148062723 L1MA2	2.82	9.04	6.22	852
399	hg38ChrX 41845889-41846743 L1MA2 r	0.70	8.89	8.19	855
400	hg38ChrX 81604892-81605748 L1MB8 r	2.57	8.28	5.72	857
401	hg38ChrX 75674651-75675511 L1M4 re	0.93	15.45	14.52	861
402	hg38ChrX 143130695-143131560 L1M7	5.54	15.94	10.39	866
403	hg38ChrX 85738557-85739423 L1M4b r	4.15	23.99	19.84	867
404	hg38ChrX 146663146-146664013 L1MDb	0.81	11.41	10.60	868
405	hg38ChrX 68175829-68176696 L1MB2 r	2.65	14.29	11.64	868
406	hg38ChrX 71805193-71806063 L1M1 re	3.56	17.34	13.78	871
407	hg38ChrX 16018292-16019163 L1MB5 r	2.87	9.29	6.42	872
408	hg38ChrX 124758189-124759061 L1MB1	1.37	14.09	12.71	873
409	hg38ChrX 19182589-119183461 L1MD1	0.80	13.97	13.17	873
410	hg38ChrX 122853022-122853897 L1MA2	2.05	14.27	12.21	876
411	hg38ChrX 94560539-94561414 L1PBa r	2.74	6.28	3.54	876
412	hg38ChrX 82539808-82540684 L1MD re	0.80	14.37	13.57	877
413	hg38ChrX 145551896-145552776 L1MA4	1.70	13.17	11.46	881
414	hg38ChrX 70305186-70306073 L1MB7 r	2.93	6.31	3.38	888
415	hg38ChrX 140675002-140675890 L1PA1	1.35	10.24	8.89	889
416	hg38ChrX 56507258-56508148 L1PBa1	1.68	14.48	12.79	891
417	hg38ChrX 141960451-141961342 L1MC3	6.17	14.24	8.07	892
418	hg38ChrX 93319130-93320023 L1P3 re	1.90	3.47	1.57	894
419	hg38ChrX 37725414-37726308 L1MC1 r	1.90	6.15	4.25	895
420	hg38ChrX 147050467-147051362 L1ME1	1.23	11.05	9.82	896
421	hg38ChrX 87532819-87533714 L1PA3 r	1.34	11.05	9.71	896
422	hg38ChrX 42140250-42141147 L1M4a2	4.23	22.83	18.60	898
423	hg38ChrX 129084994-129085893 L1PA3	1.33	9.67	8.33	900
424	hg38ChrX 56417977-56418881 L1P4e r	2.54	13.59	11.05	905
425	hg38ChrX 102021157-102022062 L1M3c	9.60	13.58	3.97	906
426	hg38ChrX 106676593-106677498 L1M4	3.97	9.38	5.41	906
427	hg38ChrX 9192330-9193236 HAL1M8 re	3.42	12.13	8.71	907
428	hg38ChrX 126605249-126606166 L1MB4	1.42	14.60	13.18	918
429	hg38ChrX 25468074-25468993 L1MA8 r	2.61	14.78	12.17	920
430	hg38ChrX 137649596-137650517 L1MA6	3.25	10.52	7.27	922
431	hg38ChrX 117104165-117105087 L1MA8	1.41	10.51	9.10	923
432	hg38ChrX 40355698-40356622 L1ME3B	2.59	12.76	10.16	925
433	hg38ChrX 76285868-76286792 L1MA2 r	1.08	10.81	9.73	925
434	hg38ChrX 83731873-83732798 L1MA4 r	4.10	7.34	3.24	926
435	hg38ChrX 149161268-149162196 L1PRE	2.26	10.23	7.97	929
436	hg38ChrX 132431073-132432006 L1MC4	5.25	10.49	5.25	934
437	hg38ChrX 82887404-82888337 L1MA9 r	1.07	10.17	9.10	934
438	hg38ChrX 140275524-140276458 L1MD	2.14	9.95	7.81	935
439	hg38ChrX 155243476-155244411 L1M2	1.71	11.86	10.15	936
440	hg38ChrX 80781681-80782616 L1MA3 r	3.63	17.63	14.00	936
441	hg38ChrX 151349810-151350748 L1M5	2.24	9.48	7.24	939
442	hg38ChrX 16617950-16618889 L1MB7 r	0.74	7.23	6.49	940
443	hg38ChrX 33404384-33405324 L1PA13	2.23	12.33	10.10	941
444	hg38ChrX 149767148-149768092 L1ME1	2.01	9.10	7.09	945
445	hg38ChrX 98467233-98468178 L1PA13	1.06	13.42	12.37	946
446	hg38ChrX 79744011-79744962 L1PA3 r	3.47	14.11	10.63	950
447	hg38ChrX 86999000-86999951 L1M1 re	1.37	13.34	11.97	952
448	hg38ChrX 136969171-136970123 L1ME1	1.05	15.11	14.06	953
449	hg38ChrX 153014609-153015564 L1PA1	3.97	13.60	9.62	956
450	hg38ChrX 4567889-4568844 L1MCa res	8.26	8.05	-0.21	956
451	hg38ChrX 146108523-146109479 L1MA4	1.67	10.34	8.67	957
452	hg38ChrX 125754148-125755105 L1MB5	3.44	6.26	2.82	958
453	hg38ChrX 144219580-144220537 L1M3f	5.11	13.36	8.25	958
454	hg38ChrX 152563644-152564605 L1MA5	0.62	14.14	13.51	962
455	hg38ChrX 98250558-98251519 L1PA7 r	1.98	13.10	11.12	962
456	hg38ChrX 128205138-128206100 L1PA1	2.39	11.63	9.24	963
457	hg38ChrX 27862675-27863640 L1MA8 r	2.28	6.94	4.66	966
458	hg38ChrX 25096424-25097392 L1MA9 r	3.61	9.80	6.19	969
459	hg38ChrX 128505864-128506834 L1MA9	1.75	11.12	9.37	971
460	hg38ChrX 25861153-25862123 L1PB1 r	1.75	12.36	10.61	971
461	hg38ChrX 94664991-94665964 L1M5 re	4.62	6.67	2.05	974
462	hg38ChrX 101866763-101867737 L1P1	1.95	12.92	10.97	975
463	hg38ChrX 132138802-132139780 L1PB4	1.84	11.64	9.81	979
464	hg38ChrX 127664735-127665716 L1PA1	1.73	12.12	10.39	982
465	hg38ChrX 89265112-89266093 L1MA10	1.43	14.15	12.73	982
466	hg38ChrX 32615706-32616690 L1MC3 r	3.15	11.68	8.53	985
467	hg38ChrX 52307112-52308096 L1MC2 r	3.15	11.88	8.73	985
468	hg38ChrX 91160016-91161000 L1MA2 r	0.61	10.25	9.64	985
469	hg38ChrX 45832796-45833786 L1MEg2	2.42	16.25	13.82	991
470	hg38ChrX 14456221-14457212 L1MA5A	1.11	10.99	9.88	992
471	hg38ChrX 50715494-50716487 L1MA8 r	1.71	9.05	7.34	994
472	hg38ChrX 71678062-71679057 L1M1 re	3.71	16.97	13.25	996
473	hg38ChrX 6346831-6347856 L1MEd res	2.44	16.08	13.65	1026
474	hg38ChrX 147393296-147394328 L1PA1	5.91	10.84	4.94	1033
475	hg38ChrX 148616577-148617648 L1PA4	1.12	10.63	9.51	1072
476	hg38ChrX 54942393-54943469 L1MEd r	2.32	19.31	16.99	1077
477	hg38ChrX 70690870-70691962 L1MA9 r	1.28	6.77	5.49	1093
478	hg38ChrX 152234276-152235376 L1P4	1.36	17.80	16.44	1101
479	hg38ChrX 75084495-75085599 L1ME1 r	1.18	12.85	11.67	1105
480	hg38ChrX 66478714-66479822 L1MDa r	2.34	13.53	11.18	1109
481	hg38ChrX 106357280-106358410 L1PA1	1.50	12.20	10.70	1131
482	hg38ChrX 30328838-30329985 L1MB7 r	3.14	9.15	6.01	1148
483	hg38ChrX 71969613-71970774 L1MB8 r	1.29	20.31	19.02	1162
484	hg38ChrX 124760520-124761706 L1PA6	1.01	12.72	11.71	1187
485	hg38ChrX 38242028-38243222 L1ME1 r	1.42	10.96	9.54	1195
486	hg38ChrX 76479780-76481022 L1PB1 r	1.85	11.34	9.49	1243
487	hg38ChrX 121764413-121765662 L1MB1	2.80	11.36	8.56	1250
488	hg38ChrX 104547693-104548954 L1PA3	3.88	12.60	8.72	1262
489	hg38ChrX 91507654-91508928 L1PREC2	4.78	13.41	8.63	1275
490	hg38ChrX 80579617-80580910 L1M1 re	3.01	13.99	10.97	1294
491	hg38ChrX 139668769-139670076 L1MB8	2.29	11.01	8.72	1308
492	hg38ChrX 110967700-110969017 L1MD2	1.67	9.71	8.04	1318
493	hg38ChrX 143154304-143155624 L1PA3	1.89	11.28	9.39	1321
494	hg38ChrX 24094724-24096044 L1MD1 r	2.57	10.98	8.40	1321
495	hg38ChrX 131789962-131791350 L1M4	2.59	13.39	10.80	1389
496	hg38ChrX 90474740-90476129 L1MB8 r	2.81	9.06	6.26	1390
497	hg38ChrX 146706749-146708167 L1PA1	2.26	16.63	14.38	1419
498	hg38ChrX 84606980-84608399 L1P4 re	2.96	15.00	12.04	1420
499	hg38ChrX 114794379-114795800 L1PA4	2.18	13.01	10.83	1422
500	hg38ChrX 66811025-66812485 L1PB1 r	1.98	9.51	7.53	1461
501	hg38ChrX 154917301-154918767 L1MA4	1.91	16.16	14.25	1467
502	hg38ChrX 68215024-68216498 L1MC2 r	2.24	20.61	18.37	1475
503	hg38ChrX 25838459-25839960 L1PB3 r	2.73	6.72	3.99	1502
504	hg38ChrX 20658914-20660430 L1P1 re	3.69	13.12	9.43	1517
505	hg38ChrX 88246246-88247772 L1MB7 r	3.86	11.13	7.27	1527
506	hg38ChrX 116989048-116990635 L1MC3	4.60	11.15	6.55	1588
507	hg38ChrX 121720412-121722018 L1MEf	1.49	13.19	11.70	1607
508	hg38ChrX 47898439-47900068 L1ME1 r	2.88	10.06	7.18	1630
509	hg38ChrX 63282822-63284524 L1PA16	5.58	5.23	-0.35	1703
510	hg38ChrX 155901521-155903230 L1PA3	3.39	12.87	9.47	1710
511	hg38ChrX 76132210-76133947 L1PA11	2.07	13.58	11.51	1738
512	hg38ChrX 126330974-126332727 L1PA1	1.25	12.09	10.83	1754
513	hg38ChrX 105366247-105368013 L1MA7	2.72	15.05	12.34	1767
514	hg38ChrX 65563849-65565651 L1PA7 r	2.94	10.43	7.49	1803
515	hg38ChrX 72557222-72559029 L1PBa r	2.21	11.73	9.51	1808
516	hg38ChrX 146777390-146779201 L1PA1	3.15	10.15	7.01	1812
517	hg38ChrX 28021974-28023848 L1PB4 r	2.24	7.79	5.55	1875
518	hg38ChrX 45576396-45578281 L1MA8 r	2.49	8.22	5.73	1886
519	hg38ChrX 87795317-87797229 L1PB re	2.46	14.85	12.39	1913
520	hg38ChrX 8976903-8978817 L1PA16 re	1.83	10.55	8.72	1915
521	hg38ChrX 82003119-82005068 L1P3 re	3.85	10.31	6.46	1950
522	hg38ChrX 63215054-63217043 L1M2 re	2.71	9.95	7.24	1990
523	hg38ChrX 54986905-54988923 L1MB7 r	2.53	13.67	11.14	2019
524	hg38ChrX 92563886-92565942 L1MA2 r	2.43	14.44	12.01	2057
525	hg38ChrX 81310769-81312857 L1PB1 r	2.97	11.73	8.76	2089
526	hg38ChrX 67916826-67918943 L1PA7 r	3.54	13.03	9.49	2118
527	hg38ChrX 55734262-55736385 L1PBa r	3.44	16.71	13.28	2124
528	hg38ChrX 121224978-121227106 L1PA1	2.96	14.28	11.32	2129
529	hg38ChrX 32794617-32796751 L1MA4A	2.81	12.37	9.56	2135
530	hg38ChrX 36624868-36627013 L1MA3 r	2.56	12.67	10.11	2146
531	hg38ChrX 72661775-72663922 L1M2 re	3.40	11.96	8.57	2148
532	hg38ChrX 55945477-55947648 L1PA3 r	2.49	12.15	9.67	2172
533	hg38ChrX 66138258-66140435 L1MA1 r	3.49	15.15	11.66	2178
534	hg38ChrX 43612022-43614216 L1MA2 r	2.87	10.11	7.24	2195
535	hg38ChrX 75170337-75172536 L1PB1 r	3.68	13.59	9.91	2200
536	hg38ChrX 116837077-116839284 L1M1	4.44	10.28	5.84	2208
537	hg38ChrX 5471235-5473448 L1PREC2 r	2.89	13.78	10.89	2214
538	hg38ChrX 143368686-143370907 L1MCa	3.83	12.60	8.78	2222
539	hg38ChrX 141744806-141747042 L1PA3	2.41	11.04	8.63	2237
540	hg38ChrX 142123603-142125846 L1PA5	2.36	12.52	10.16	2244
541	hg38ChrX 116317196-116319486 L1M1	3.67	12.35	8.69	2291
542	hg38ChrX 14172987-14175277 L1MA2 r	3.23	11.17	7.94	2291
543	hg38ChrX 35596209-35598539 L1PB1 r	1.76	14.41	12.66	2331
544	hg38ChrX 46142898-46145233 L1MA1 r	2.23	12.80	10.57	2336
545	hg38ChrX 78205548-78207913 L1PA7 r	3.13	12.05	8.92	2366
546	hg38ChrX 78933689-78936061 L1PA13	3.37	10.20	6.83	2373
547	hg38ChrX 58030268-58032656 L1PREC2	5.11	8.54	3.43	2389
548	hg38ChrX 58040261-58042649 L1PA13	3.39	9.88	6.49	2389
549	hg38ChrX 117674334-117676779 L1PA1	2.66	12.10	9.44	2446
550	hg38ChrX 57662327-57664836 L1PA10	3.43	12.35	8.92	2510
551	hg38ChrX 102912636-102915179 L1PB1	2.08	14.15	12.07	2544
552	hg38ChrX 50557240-50559794 L1PB1 r	3.01	13.97	10.96	2555
553	hg38ChrX 92796589-92799149 L1MA2 r	1.68	11.91	10.23	2561
554	hg38ChrX 46052011-46054575 L1PA4 r	2.30	10.45	8.15	2565
555	hg38ChrX 34343977-34346565 L1PA8A	3.17	14.02	10.85	2589
556	hg38ChrX 99167236-99169842 L1PB3 r	2.57	11.66	9.09	2607
557	hg38ChrX 138030025-138032668 L1PA1	2.72	11.95	9.23	2644
558	hg38ChrX 90196148-90198828 L1P2 re	4.70	14.47	9.77	2681
559	hg38ChrX 64491966-64494665 L1PB7 r	2.44	14.89	12.44	2700
560	hg38ChrX 55360029-55362733 L1PA10	3.44	11.24	7.80	2705
561	hg38ChrX 51018803-51021520 L1PA7 r	3.20	13.61	10.41	2718
562	hg38ChrX 73469364-73472131 L1P4a r	3.25	12.75	9.50	2768
563	hg38ChrX 86336356-86339187 L1P2 re	3.35	15.11	11.76	2832
564	hg38ChrX 66654526-66657411 L1MA4A	2.95	12.47	9.53	2886
565	hg38ChrX 81656239-81659146 L1MA4A	2.82	11.73	8.91	2908
566	hg38ChrX 72967769-72970683 L1PA6 r	2.71	12.18	9.47	2915
567	hg38ChrX 94809143-94812058 L1PREC2	2.78	11.28	8.50	2916
568	hg38ChrX 12662196-12665148 L1PA7 r	2.37	13.11	10.73	2953
569	hg38ChrX 84969153-84972106 L1PA8A	3.52	14.32	10.80	2954
570	hg38ChrX 110558907-110561867 L1PA1	3.07	13.04	9.96	2961
571	hg38ChrX 95332069-95335029 L1PBa r	3.21	13.78	10.57	2961

572	monDom5ChrX 9222240-9224649 L1 Mdo3c result	1.95	14.52	12.57	2410	572	mm10ChrX 57525509-57528198 L1 Mus2	2.90	12.60	9.70	2690	572	hg38ChrX 143775508-143778497 L1PA1	3.14	13.18	10.03	2990
573	monDom5ChrX 67587823-67590244 L1 Mdo3b result	2.52	17.13	14.62	2422	573	mm10ChrX 28275892-28278584 L1VL2 r	3.12	14.63	11.51	2693	573	hg38ChrX 45418451-45421461 L1M1 re	2.92	13.62	10.69	3011
574	monDom5ChrX 31771219-31773671 L1 Mdo5 result	1.39	19.08	17.69	2453	574	mm10ChrX 83238633-83241338 L1Md F2	3.58	12.53	8.94	2706	574	hg38ChrX 78860858-78863872 L1M1 re	3.12	14.76	11.64	3015
575	monDom5ChrX 4905699-4908152 L1 Mars1 result	1.87	23.06	21.19	2454	575	mm10ChrX 78761924-78764652 Lx4B re	2.89	12.13	9.23	2729	575	hg38ChrX 78176144-78179165 L1P1 re	3.18	16.64	13.47	3022
576	monDom5ChrX 23930773-23933234 L1 Mdo5 result	2.68	17.14	14.46	2462	576	mm10ChrX 28578005-28580744 L1Md F2	3.50	14.85	11.35	2740	576	hg38ChrX 84450115-84453142 L1M2 re	2.58	14.46	11.89	3028
577	monDom5ChrX 74921985-74924513 L1 Mdo3c result	1.86	24.59	22.74	2529	577	mm10ChrX 26983156-26985898 L1Md A	2.95	15.75	12.80	2743	577	hg38ChrX 81046042-81049078 L1PB4 r	2.21	13.60	11.39	3037
578	monDom5ChrX 36856076-36858610 L1 Mdo3c result	1.89	15.90	14.00	2535	578	mm10ChrX 123531625-123534384 L1Md	2.43	14.64	12.21	2760	578	hg38ChrX 42222512-42225562 L1MA4A	2.62	15.31	12.68	3051
579	monDom5ChrX 51306302-51308841 HAL1 Opos result	2.60	14.88	12.28	2540	579	mm10ChrX 86431894-86434679 L1 Mus3	3.37	13.85	10.48	2786	579	hg38ChrX 116131430-116134490 L1MA2	3.17	9.51	6.34	3061
580	monDom5ChrX 4858146-4860734 L1 Opos0 result	3.13	13.87	10.74	2589	580	mm10ChrX 38925567-38928376 L1Md F2	4.34	12.56	8.22	2810	580	hg38ChrX 78774283-78777376 L1PB1 r	3.39	10.96	7.56	3094
581	monDom5ChrX 38127325-38129934 L1 Opos result	1.76	23.33	21.57	2610	581	mm10ChrX 33202040-33204850 L1 Mm r	3.06	15.12	12.06	2811	581	hg38ChrX 56970532-56973657 L1PA4 r	2.43	12.86	10.43	3126
582	monDom5ChrX 17736284-17738904 L1 Mdo3 result	2.29	21.59	19.31	2621	582	mm10ChrX 81847963-81850824 L1Md F	2.73	15.41	12.68	2862	582	hg38ChrX 75626075-75629246 L1PB1 r	2.11	13.24	11.13	3172
583	monDom5ChrX 51585475-51588170 L1 Mdo3c result	1.89	15.06	13.17	2696	583	mm10ChrX 131304391-131307327 L1VL4	3.64	14.20	10.55	2937	583	hg38ChrX 92033106-92036303 L1MA2 r	1.56	16.48	14.92	3198
584	monDom5ChrX 72418838-72421540 L1 Mdo2 result	1.48	26.93	25.45	2703	584	mm10ChrX 26794360-26797340 Lx4A re	2.11	10.27	8.15	2981	584	hg38ChrX 82683995-82687197 L1PA10	2.78	12.24	9.46	3203
585	monDom5ChrX 11433345-11436081 L1 Mdo1 result	1.75	15.86	14.10	2737	585	mm10ChrX 26653038-26656024 Lx4A re	2.18	10.65	8.47	2987	585	hg38ChrX 143960674-143963877 L1PA1	3.00	13.42	10.42	3204
586	monDom5ChrX 28795090-28797835 L1 Mdo2 result	3.53	23.05	19.52	2746	586	mm10ChrX 50707373-50710393 L1VL2 r	2.98	15.89	12.91	3021	586	hg38ChrX 108098365-108101571L1MD2	4.40	9.79	5.39	3207
587	monDom5ChrX 27590072-27592842 L1 Mdo3c result	2.60	16.38	13.79	2771	587	mm10ChrX 61983754-61986780 L1Md F2	2.18	15.89	13.71	3027	587	hg38ChrX 127480315-127483521 L1MA2	3.12	10.94	7.83	3207
588	monDom5ChrX 76802261-76805058 L1 Mdo4 result	2.07	24.34	22.27	2798	588	mm10ChrX 126838787-126841816 L1 Mu	4.52	14.13	9.60	3030	588	hg38ChrX 155430462-155433680 L1PA5	2.89	14.20	11.31	3219
589	monDom5ChrX 64537485-64540290 L1 Mdo2 result	2.67	15.61	12.94	2806	589	mm10ChrX 79102026-79105066 L1Md F2	2.93	15.88	12.96	3041	589	hg38ChrX 58026982-58030256 L1PREC2	2.87	14.53	11.66	3275
590	monDom5ChrX 68156377-68159202 L1 Mdo2 result	2.65	14.90	12.24	2826	590	mm10ChrX 127567814-127570893 L1Md	2.53	15.58	13.05	3080	590	hg38ChrX 76204027-76207323 L1MA1 r	3.28	14.86	11.59	3297
591	monDom5ChrX 60043088-60045919 HAL1 Opos result	2.22	17.06	14.83	2832	591	mm10ChrX 33662504-33665593 L1 Mus3	3.88	12.62	8.74	3090	591	hg38ChrX 67378211-67381524 L1MA1 r	2.87	14.79	11.92	3314
592	monDom5ChrX 49268765-49271636 L1 Mdo1 result	2.30	24.23	21.94	2872	592	mm10ChrX 5153951-5157042 L1 Mus3 r	3.69	12.61	8.93	3092	592	hg38ChrX 57953450-57956773 L1PA4 r	3.19	14.29	11.10	3324
593	monDom5ChrX 75397625-75400553 L1 Mdo2 result	2.90	16.25	13.35	2929	593	mm10ChrX 126795993-126799090 L1 Mu	3.42	13.52	10.10	3098	593	hg38ChrX 58015160-58018485 L1PREC2	4.63	9.44	4.81	3326
594	monDom5ChrX 51740614-51743628 L1 Mdo1 result	2.49	16.15	13.67	3015	594	mm10ChrX 28159064-28162166 L1Md F2	3.83	12.92	9.09	3103	594	hg38ChrX 43346867-43350213 L1PA17	3.11	13.06	9.95	3347
595	monDom5ChrX 23251007-23254025 L1 Mdo3c result	2.75	16.50	13.75	3019	595	mm10ChrX 19922640-19925754 L1Md F	3.98	16.57	12.58	3115	595	hg38ChrX 39077794-39081179 L1PA12	2.92	12.99	10.07	3386
596	monDom5ChrX 72893130-72896153 L1 Mdo3c result	2.94	23.02	20.07	3024	596	mm10ChrX 27985327-27988441 L1 Mm r	3.37	13.45	10.08	3115	596	hg38ChrX 91955393-91958786 L1PA11	4.24	11.99	7.75	3394
597	monDom5ChrX 12172377-12175402 L1 Mdo3b result	1.39	21.88	20.49	3026	597	mm10ChrX 28646420-28649534 L1 Mm r	3.37	13.64	10.27	3115	597	hg38ChrX 70613123-70616531 L1M1 re	2.14	16.22	14.08	3409
598	monDom5ChrX 26301754-26304826 L1 Mdo3a result	2.57	16.04	13.47	3073	598	mm10ChrX 108680508-108683626L1Md F	2.08	15.61	13.53	3119	598	hg38ChrX 68620695-68624107 L1MA2 r	2.67	15.03	12.36	3413
599	monDom5ChrX 34436426-34439504 L1 Mdo4 result	2.50	14.75	12.24	3079	599	mm10ChrX 35829477-35832632 Lx2B2 r	2.79	14.39	11.60	3156	599	hg38ChrX 74562660-74566096 L1MA2 r	2.09	12.71	10.62	3437
600	monDom5ChrX 62538626-62541710 L1 Mdo3 result	2.40	17.21	14.81	3085	600	mm10ChrX 132593550-132596708 Lx7 r	3.61	10.60	7.00	3159	600	hg38ChrX 33684598-33688050 L1MA1 r	2.23	14.45	12.22	3453
601	monDom5ChrX 27571520-27574625 L1 Mdo3c result	2.77	17.29	14.52	3106	601	mm10ChrX 65010743-65013908 L1 Mus2	3.22	13.99	10.77	3166	601	hg38ChrX 36613001-36616493 L1PA13	2.81	13.51	10.71	3493
602	monDom5ChrX 33635190-33638366 L1 Mdo3c result	1.48	15.52	14.04	3177	602	mm10ChrX 147315591-147318764 L1 Mu	2.17	14.24	12.07	3174	602	hg38ChrX 52216381-52219873 L1MA2 r	3.29	14.03	10.74	3493
603	monDom5ChrX 23130766-23133956 L1 Mdo3c result	2.88	20.37	17.49	3191	603	mm10ChrX 5518593-5521766 L1 Mus2 r	3.62	11.41	7.78	3174	603	hg38ChrX 85805504-85809069 L1PREC2	3.65	12.11	8.47	3566
604	monDom5ChrX 22596232-22599423 NN L1 Mdo4 result	3.01	21.77	18.77	3192	604	mm10ChrX 35652830-35656048 L1VL1 r	3.60	14.35	10.75	3219	604	hg38ChrX 75839723-75843294 L1PREC2	3.39	13.10	9.71	3572
605	monDom5ChrX 12760693-12763894 L1 Mdo1 result	2.53	21.61	19.08	3202	605	mm10ChrX 56410300-56413527 L1Md T	3.87	13.23	9.36	3228	605	hg38ChrX 129419636-129423210 L1PA7	2.94	13.17	10.24	3575
606	monDom5ChrX 73967804-73971009 L1 Opos1 result	1.75	19.28	17.53	3206	606	mm10ChrX 14246776-14250009 L1 Mus1	3.87	15.71	11.84	3234	606	hg38ChrX 122001195-122004781 L1PA1	3.76	11.60	7.83	3587
607	monDom5ChrX 37844233-37847483 L1 Mdo6 result	2.92	15.07	12.15	3251	607	mm10ChrX 92735884-92739141 Lx4A re	2.58	13.29	10.71	3258	607	hg38ChrX 88317182-88320770 L1PA5 r	2.26	12.82	10.56	3589
608	monDom5ChrX 8914045-8917321 L1 Mdo3c result	1.95	16.30	14.34	3277	608	mm10ChrX 110840771-110844067 L1 Mu	3.94	12.25	8.31	3297	608	hg38ChrX 74856584-74860185 L1MA3 r	2.97	14.24	11.27	3602
609	monDom5ChrX 51788197-51791487 L1 Opos0 result	3.40	12.40	8.99	3291	609	mm10ChrX 30664384-30667713 L1 Mus3	2.82	13.00	10.18	3330	609	hg38ChrX 81723803-81727426 L1PA14	3.48	13.44	9.96	3624
610	monDom5ChrX 70258805-70262127 L1 Opos0 result	2.23	14.17	11.95	3323	610	mm10ChrX 145830870-145834266 Lx5 r	3.83	11.39	7.57	3397	610	hg38ChrX 144594426-144598051 L1MA2	3.03	12.11	9.07	3626
611	monDom5ChrX 66000269-66003595 HAL1 Opos1 result	2.61	25.49	22.87	3327	611	mm10ChrX 107213898-107217295 L1Md	4.38	11.62	7.24	3398	611	hg38ChrX 67011409-67015040 L1PA14	3.06	11.78	8.73	3632
612	monDom5ChrX 19168729-19172077 L1 Opos2 result	2.54	16.54	14.00	3349	612	mm10ChrX 120088479-120091889 L1 Mu	2.84	16.56	13.72	3411	612	hg38ChrX 127486734-127490437 L1M1	3.73	14.17	10.45	3704
613	monDom5ChrX 54684788-54688140 L1 Mdo2 result	3.19	16.02	12.82	3353	613	mm10ChrX 15406400-15409885 L1 Mus2	3.10	16.04	12.94	3486	613	hg38ChrX 73666662-73670366 PA11 re	3.86	11.04	7.18	3705
614	monDom5ChrX 16256317-16259695 L1 Mdo2 result	3.11	16.37	13.26	3379	614	mm10ChrX 45743468-45746954 L1Md F2	4.45	14.17	9.72	3487	614	hg38ChrX 43279814-43283566 L1PA15	3.33	10.42	7.09	3753
615	monDom5ChrX 15259974-15263360 L1 Mdo3c result	1.98	16.42	14.44	3387	615	mm10ChrX 83340738-83344273 L1Md F2	3.70	12.78	9.08	3536	615	hg38ChrX 142949475-142953261 L1PA1	2.59	12.44	9.85	3787
616	monDom5ChrX 4430127-4433536 HAL1 Opos result	3.17	21.70	18.53	3410	616	mm10ChrX 111478939-111482486 L1Md	3.69	13.42	9.72	3548	616	hg38ChrX 142953258-142957052 L1PA7	2.40	12.65	10.25	3795
617	monDom5ChrX 76630759-76634175 L1 Mdo3a result	3.16	20.66	17.50	3417	617	mm10ChrX 129026528-129030117 L1Md	3.93	15.88	11.95	3590	617	hg38ChrX 34949627-34953432 L1PREC2	2.92	13.90	10.98	3806
618	monDom5ChrX 72422047-72425470 L1 Mdo2 result	3.13	16.00	12.88	3424	618	mm10ChrX 67324545-67328151 Lx3 Mus	2.58	13.92	11.34	3607	618	hg38ChrX 32652143-32655976 L1PA5 r	2.14	12.57	10.43	3834
619	monDom5ChrX 27561430-27564875 L1 Mdo3c result	2.35	15.38	13.03	3446	619	mm10ChrX 109710256-109713865 L1 Mm	3.96	13.80	9.83	3610	619	hg38ChrX 114086743-114090623 L1MA3	2.68	14.64	11.96	3881
620	monDom5ChrX 47824827-47828292 L1 Mdo3a result	2.05	21.49	19.45	3466	620	mm10ChrX 136472419-136476066 L1Md	3.73	13.40	9.68	3648	620	hg38ChrX 96656454-96660383 L1PA13	3.10	15.57	12.47	3930
621	monDom5ChrX 30719140-30722635 NN L1 Mdo3c result	2.46	13.99	11.53	3496	621	mm10ChrX 119846147-119849813 L1Md	3.55	12.76	9.22	3667	621	hg38ChrX 121277194-121281180 L1MA2	2.58	13.49	10.91	3987
622	monDom5ChrX 41712116-41715613 HAL1N1 MD result	3.20	17.50	14.29	3498	622	mm10ChrX 14417262-14420951 L1Md F2	4.12	12.17	8.05	3690	622	hg38ChrX 84453137-84457131 L1MA3 r	2.85	13.19	10.34	3995
623	monDom5ChrX 32600128-32603629 L1 Mdo3c result	2.48	16.28	13.79	3502	623	mm10ChrX 116930445-116934150 L1Md	3.70	12.36	8.66	3706	623	hg38ChrX 73542582-73546602 L1PA7 r	3.06	14.57	11.51	4021
624	monDom5ChrX 51483529-51487057 L1 Opos result	2.44	20.88	18.45	3529	624	mm10ChrX 126835067-126838784 L1 Mu	3.77	14.20	10.44	3718	624	hg38ChrX 56190047-56194072 L1MA3 r	3.38	13.46	10.08	4026
625	monDom5ChrX 29657455-29661018 L1 Mdo3c result	2.58	16.11	13.52	3564	625	mm10ChrX 91582505-91586254 L1 Mur1	2.96	12.40	9.44	3750	625	hg38ChrX 57891569-57895623 L1PB4 r	2.34	12.18	9.84	4055
626	monDom5ChrX 30936566-30940169 L1 Mdo3a result	2.39	16.26	13.87	3604	626	mm10ChrX 109170395-109174203 L1Md	2.60	16.12	13.52	3809	626	hg38ChrX 125823091-125827147 L1PA1	3.60	10.30	6.70	4057
627	monDom5ChrX 21686308-21689968 L1 Mdo4 result	2.73	18.52	15.79	3661	627	mm10ChrX 68161427-68165244 L1Md F2	3.35	14.30	10.95	3818	627	hg38ChrX 115962699-115966769 L1PB1	2.87	11.37	8.50	4071
628	monDom5ChrX 18703291-18706968 L1-1 MD result	2.77	17.10	14.33	3678	628	mm10ChrX 147734444-147738281 L1VL1	3.39	14.43	11.05	3838	628	hg38ChrX 91649682-91653778 L1MA3 r	2.93	14.35	11.42	4097
629	monDom5ChrX 21443265-21446942 L1 Mdo4 result	2.53	22.24	19.71	3678	629	mm10ChrX 116523977-116527817 L1Md	2.42	15.44	13.02	3841	629	hg38ChrX 113319742-113323839 L1MB1	2.29	15.23	12.93	4098
630	monDom5ChrX 61232049-61235747 L1 Mdo1 result	2.87	17.17	14.30	3699	630	mm10ChrX 40826595-40830488 L1 Mus2	3.80	12.97	9.17	3894	630	hg38ChrX 21218622-21222739 L1PA5 r	2.72	12.38	9.66	4118
631	monDom5ChrX 56624853-56628561 L1-1 MD result	2.88	16.39	13.51	3709	631	mm10ChrX 118524128-118528024 L1Md	3.49	13.32	9.83	3897	631	hg38ChrX 34597238-34601391 L1PREC2	3.37	14.30	10.93	4154
632	monDom5ChrX 60749534-60753246 L1 Mdo4 result	2.18	18.74	16.56	3713	632	mm10ChrX 106731102-106735019 L1Md	3.34	13.68	10.34	3918	632	hg38ChrX 128378369-128382528 L1MA7	2.72	12.84	10.12	4160
633	monDom5ChrX 48502510-48506262 L1 Mdo5 result	2.32	20.65	18.33	3753	633	mm10ChrX 39366013-39369950 L1Md A	3.22	13.89	10.67	3938	633	hg38ChrX 96254214-96258374 L1PA5 r	2.45	13.10	10.65	4161
634	monDom5ChrX 60427305-60431057 L1 Mdo4 result	1.97	21.77	19.80	3753	634	mm10ChrX 65676190-65680144 L1 Mus3	5.92	11.20	5.28	3955	634	hg38ChrX 57242460-57246636 L1P3 re	3.18	14.34	11.16	4177
635	monDom5ChrX 74917600-74921379 L1 Mdo3c result	2.25	15.53	13.28	3780	635	mm10ChrX 116814388-116818365 L1 Mu	2.82	14.40	11.59	3978	635	hg38ChrX 73231546-73235732 L1PA6 r	2.91	13.57	10.65	4187
636	monDom5ChrX 49584412-49588206 L1 Mdo3c result	2.87	16.73	13.86	3795	636	mm10ChrX 28715815-28719842 L1Md F2	3.13	12.16	9.04	4028	636	hg38ChrX 98051129-98055316 L1MA2 r	2.63	12.56	9.93	4188
637	monDom5ChrX 30393137-30396962 L1 Mdo4 result	2.67	17.56	14.90	3826	637	mm10ChrX 80401941-80405982 Lx4B re	2.92	15.34	12.42	4042	637	hg38ChrX 56388486-56392677 L1PA4 r	2.58	13.36	10.78	4192
638	monDom5ChrX 30181402-30185246 L1 Opos1 result	2.05	17.58	15.53	3845	638	mm10ChrX 108167863-108171908 Lx5c	4.15	13.10	8.95	4046	638	hg38ChrX 83839861-83844072 L1PA2 r	2.54	13.51	10.97	4212
639	monDom5ChrX 18850347-18854194 L1 Opos result	2.49	17.70	15.20	3848	639	mm10ChrX 61309102-61313152 L1 Mus2	6.44	10.64	4.20	4051	639	hg38ChrX 78383266-78387481 L1MA2 r	3.23	16.60	13.38	4216
640	monDom5ChrX 23174632-23178490 NN L1-1 MD result	2.82	21.27	18.45	3859	640	mm10ChrX 110133886-110137967 L1Md	4.43	13.35	8.92	4082	640	hg38ChrX 57559281-57563525 L1PA4 r	2.66	13.12	10.46	4245
641	monDom5ChrX 76294367-76298248 L1 Opos1 result	3.68	15.66	11.98	3882	641	mm10ChrX 90705496-90709647 L1Md T	3.73	14.38	10.65	4152	641	hg38ChrX 48869851-48874138 L1PA4 r	2.73	13.06	10.33	4288
642	monDom5ChrX 19239064-19242949 L1 Opos0 result	2.55	13.90	11.35	3886	642	mm10ChrX 121168575-121172749 L1 Mu	2.78	16.36	13.58	4175	642	hg38ChrX 54731466-54735789 L1PA4 r	3.01	13.99	10.99	4324
643	monDom5ChrX 21205782-21209760 L1 Mdo3c result	2.11	21.16	19.05	3979	643	mm10ChrX 9822563-9826737 L1 Mus1 r	4.17	13.08	8.91	4175	643	hg38ChrX 65401006-65405337 L1MA2 r	3.49	12.28	8.80	4332
644	monDom5ChrX 72425471-72429471 L1 Mdo3b result	2.77	20.54	17.77	4001	644	mm10ChrX 120832051-120836235 L1Md	3.75	14.22	10.47	4185	644	hg38ChrX 113363059-113367412 L1PA8	1.84	12.36	10.52	4354
645	monDom5ChrX 5368633-5372640 L1 Mdo1 result	3.14	23.40	20.26	4008	645	mm10ChrX 89062804-89067015 L1Md F2	3.44	13.53	10.09	4212	645	hg38ChrX 67815862-67820237 L1MA2 r	2.10	13.53	11.43	4376
646	monDom5ChrX 18619645-18623678 L1 Mdo4 result	1.91	23.67	21.76	4034	646	mm10ChrX 17371111-17375328 L1VL2 r	3.22	15.67	12.45	4218	646	hg38ChrX 78714314-78718779 L1PA3 r	2.98	14.04	11.06	4466
647	monDom5ChrX 8379722-8383773 L1 Mdo1 result	3.04	15.79	12.76	4052	647	mm10ChrX 156003210-156007432 L1VL4	3.46	14.40	10.94	4223	647	hg38ChrX 26891801-26896290 L1M2 re	1.94	13.74	11.80	4490
648	monDom5ChrX 5256876-5260981 L1 Mdo3a result	1.83	17.78	15.95	4106	648	mm10ChrX 18132862-18137092 L1 Mus3	4.49	14.54	10.04	4231	648	hg38ChrX 125874483-125878978 L1MA2	3.71	14.37	10.65	4496
649	monDom5ChrX 48963884-48968029 L1 Mdo3c result	2.68	16.23	13.56	4146	649	mm10ChrX 78814227-78818469 L1Md F2	3.72	14.59	10.86	4243	649	hg38ChrX 101587665-101592186 L1PA1	2.94	15.17	12.23	4522
650	monDom5ChrX 33435572-33439737 L1 Mdo3c result	2.38	17.21	14.83	4166	650	mm10ChrX 64759079-64763349 L1Md T	3.51	13.63	10.11	4271	650	hg38ChrX 55627239-55631768 L1PA6 r	2.74	14.88	12.14	4530
651	monDom5ChrX 48073035-48077215 L1 Mdo3b result	2.39	19.68	17.29	4181	651	mm10ChrX 76742046-76746327 L1Md F2	3.76	12.98	9.22	4282	651	hg38ChrX 29647367-29651911 L1MA8 r	2.60	12.94	10.34	4545
652	monDom5ChrX 57428777-57432983 L1 Mdo3 result	2.92	19.85	16.92	4207	652	mm10ChrX 31748365-31752682 L1 Mus1	3.47	13.13	9.66	4318	652	hg38ChrX 54697500-54702045 L1P1 re	2.75	14.94	12.19	4546
653	monDom5ChrX 15534138-15538348 L1 Mdo3c result	2.30	17.72	15.41	4211	653	mm10ChrX 39880481-39884839 L1 Mus1	2.91	17.64	14.73	4359	653	hg38ChrX 36120308-36124854 L1MA2 r	2.18	12.62	10.45	4547
654	monDom5ChrX 29489800-29494035 L1 Mdo3a result	2.53	15.32	12.80	4236	654	mm10ChrX 80309502-80313880 L1 Mus4	2.81	15.39	12.58	4379	654	hg38ChrX 42344804-42349416 L1MEf r	2.80	13.68	10.88	4613
655	monDom5ChrX 36374753-36379043 L1 Opos2 result	2.61	14.89	12.28	4291	655	mm10ChrX 150698233-150702633 L1Md	3.27	13.29	10.02	4401	655	hg38ChrX 147869209-147873823 L1MA2	2.77	14.21	11.44	4615
656	monDom5ChrX 74552787-74557077 L1 Mdo4 result	3.31	20.86	17.55	4291	656	mm10ChrX 129320789-129325246 L1Md	2.76	16.06	13.30	4458	656	hg38ChrX 78502264-78506919 L1M1 re	2.45	13.45	11.00	4656
657	monDom5ChrX 20505250-20509582 L1 Mdo3c result	2.28	16.29	14.01	4333	657	mm10ChrX 124422120-124426607 Lx5b	5.86	11.48	5.61	4488	657	hg38ChrX 88309840-88314507 L1PA8 r	3.26	13.90	10.65	4668
658	monDom5ChrX 49102779-49107132 L1 Mdo3b result	2.16	18.49	16.33	4354	658	mm10ChrX 123874456-123878947 Lx5b	6.01	11.22	5.21	4492	658	hg38ChrX 122260250-122264960 L1MA1	2.29	11.93	9.64	4711
659	monDom5ChrX 6639640-6643995 L1 Mdo3a result	1.88	18.20	16.32	4356	659	mm10ChrX 114178613-114183108 L1Md	3.67	16.08	12.41	4496	659	hg38ChrX 21533800-21538523 L1MA5 r	2.01	15.18	13.17	4724
660	monDom5ChrX 69385598-69389964 L1 Opos0 result	2.36	16.08	13.72	4367	660	mm10ChrX 112784356-112788867 L1 Mu	3.30	15.27	11.97	4512	660	hg38ChrX 42407690-42412413 L1PA11	2.48	12.51	10.03	4724
661	monDom5ChrX 49597635-49602007 L1-1 MD result	2.17	18.48	16.30	4373	661	mm10ChrX 33645678-33650220 L1 Mus1	3.28	13.60	10.32	4543	661	hg38ChrX 141799233-141803971 L1PA4	2.76	14.60	11.84	4739
662	monDom5ChrX 47615530-47619942 L1 Mdo2 result	2.95	20.39	17.45	4413	662	mm10ChrX 3759825-3764392 L1 Mus1 r	3.15	13.79	10.64	4568	662	hg38ChrX 55416117-55420875 L1PA10	4.20	11.85	7.65	4759
663	monDom5ChrX 13519988-13524411 L1 Mdo4 result	2.24	22.26	20.03	4424	663	mm10ChrX 55514145-55518722 L1Md F	3.49	15.20	11.71	4578	663	hg38ChrX 129206478-129211272 L1OB1	3.44	13.37	9.93	4795
664	monDom5ChrX 47705675-47710131 L1 Mdo4 result	2.09	17.01	14.92	4457	664	mm10ChrX 115116340-115120934 L1Md	3.18	14.02	10.84	4595	664	hg38ChrX 69003953-69008752 L1MC3 r	3.52	12.27	8.75	4800
665	monDom5ChrX 30350385-30354856 L1 Opos0 result	2.66	18.38	15.72	4472	665	mm10ChrX 82336777-82341376 L1Md F2	3.46	14.26	10.80	4600	665	hg38ChrX 51576976-51581780 L1PA8A	2.64	13.86	11.22	4805
666	monDom5ChrX 28978977-28983460 L1 Mdo2 result	3.06	20.56	17.51	4484	666	mm10ChrX 152838653-152843253 L1Md	2.98	13.30	10.32	4601	666	hg38ChrX 66347286-66352091 L1PA3 r	2.73	14.90	12.17	4806
667	monDom5ChrX 71488086-71492574 L1 Mdo3a result	2.61	18.27	15.66	4489	667	mm10ChrX 124432992-124437603 L1Md	3.38	12.88	9.50	4612	667	hg38ChrX 50540549-50545382 L1MCa r	3.04	15.66	12.62	4834
668	monDom5ChrX 62806931-62811454 L1 Mdo3c result	2.37	17.22	14.85	4524	668	mm10ChrX 121833850-121838463 L1Md	3.27	14.46	11.18	4614	668	hg38ChrX 109314880-109319740 L1PA7	3.23	13.97	10.74	4861
669	monDom5ChrX 6779709-6784273 L1-1 MD result	2.61	20.81	18.20	4565	669	mm10ChrX 117862031-117866657 L1Md	3.96	14.96	11.00	4627	669	hg38ChrX 90633995-90638875 L1PA16	4.00	11.64	7.64	4881
670	monDom5ChrX 8985284-8989863 L1 Mdo3c result	2.18	18.47	16.29	4580	670	mm10ChrX 79949325-79953963 L1Md F3	3.32	14.42	11.10	4639	670	hg38ChrX 78506967-78511879 L1PB1 r	2.91	13.03	10.12	4913
671	monDom5ChrX 29892092-29896674 NN L1 Mdo3 result	2.09	19.51	17.41	4583	671	mm10ChrX 147206436-147211117 L1Md	2.90	15.42	12.52	4682	671	hg38ChrX 75720033-75725001 L1PA7 r	3.68	13.16	9.48	4969
672	monDom5ChrX 48785799-48790387 L1 Mdo2 result	2.46	19.92	17.45	4589	672	mm10ChrX 88346786-88351468 L1Md A	2.97	13.92	10.95	4683	672	hg38ChrX 139158582-139163556 L1PA7	2.89	14.65	11.76	4975
673	monDom5ChrX 24079634-24084245 L1 Mdo3c result	2.67	18.04	15.37	4612	673	mm10ChrX 61215126-61219821 L1Md F2	3.68	13.99	10.31	4696	673	hg38ChrX 13724627-13729626 L1PA6 r	3.08	13.50	10.42	5000
674	monDom5ChrX 51726100-51730722 L1 Mdo3a result	2.64	19.55	16.92	4623	674	mm10ChrX 61012930-61017641 L1 Mus3	3.18	13.26	10.08	4712	674	hg38ChrX 99042498-99047517 L1MC1 r	3.15	12.49	9.34	5020
675	monDom5ChrX 71493170-71497796 L1 Mdo3c result	1.75	18.15	16.40	4627	675	mm10ChrX 78004124-78008866 L1Md F	3.69	15.14	11.45	4743	675	hg38ChrX 14683911-14688939 L1PA5 r	2.74	14.56	11.81	5029
676	monDom5ChrX 48401658-48406325 L1 Mdo5 result	2.31	18.85	16.54	4668	676	mm10ChrX 68427087-68431832 L1Md A	3.62	14.64	11.02	4746	676	hg38ChrX 100090505-100095545 L1PA1	2.96	11.15	8.19	5041
677	monDom5ChrX 51855497-51860213 L1 Mdo3a result	2.63	18.13	15.50	4717	677	mm10ChrX 81567154-81571953 L1Md T	3.67	13.56	9.90	4800	677	hg38ChrX 57609838-57614905 L1PA4 r	2.66	14.74	12.08	5068
678	monDom5ChrX 11436082-11440801 L1-1 MD result	2.54	20.36	17.82	4720	678	mm10ChrX 47410747-47415549 L1Md F2	3.41	14.35	10.93	4803	678	hg38ChrX 88630837-88635913 L1MA2 r	1.95	15.93	13.98	5077
679	monDom5ChrX 39824899-39829621 L1 Mdo4 result	2.39	19.29	16.90	4723	679	mm10ChrX 132745202-132750023 L1Md	2.94	14.31	11.36	4822	679	hg38ChrX 81792123-81797219 L1PA4 r	2.20	15.19	12.99	5097
680	monDom5ChrX 76568170-76572920 L1 Mdo3c result	2.15	19.11	16.96	4751	680	mm10ChrX 109452316-109457160 L1Md	3.74	15.38	11.64	4845	680	hg38ChrX 100304048-100309154 L1PB1	2.47	13.33	10.87	5107
681	monDom5ChrX 20881621-20886381 L1 Mdo3c result	1.81	20.14	18.34	4761	681	mm10ChrX 107516127-107521048 L1Md	3.49	16.38	12.88	4922	681	hg38ChrX 146456451-146461601 L1PA1	2.70	11.80	9.11	5151
682	monDom5ChrX 58008133-58012911 L1 Mdo1 result	2.72	19.77	17.05	4779	682	mm10ChrX 26848045-26852969 L1Md F	3.63	15.84	12.20	4925	682	hg38ChrX 67830590-67835749 L1PA7 r	2.97	14.11	11.14	5160
683	monDom5ChrX 73927521-73932331 L1 Mdo3c result	2.39	18.21	15.82	4811	683	mm10ChrX 109347633-109352559 L1VL1	3.37	14.65	11.28	4927	683	hg38ChrX 99202344-99207567 L1MA3 r	1.95	15.16	13.21	5224
684	monDom5ChrX 8874167-8878990 NN L1 Mdo3c result	1.70	17.74	16.04	4824	684	mm10ChrX 90627663-90632639 L1 Mus2	2.63	14.61	11.98	4977	684	hg38ChrX 42435647-42440874 L1PA15	2.16	13.29	11.13	5228
685	monDom5ChrX 27710004-27714843 L1 Mdo2 result	2.40	18.47	16.07	4840	685	mm10ChrX 61493725-61498715 L1Md F2	3.97	15.53	11.56	4991	685	hg38ChrX 90002190-90007499 L1PA17	3.37	14.76	11.39	5310
686	monDom5ChrX 68513692-68518534 L1 Mdo1 result	2.17	19.00	16.83	4843	686	mm10ChrX 16868879-16873888 L1Md A	3.15	15.51	12.36	5010	686	hg38ChrX 91384376-91389710 L1MA3 r	2.72	13.38	10.67	5335
687	monDom5ChrX 6136251-6141115 L1 Mdo3c result	2.67	19.59	16.92	4865	687	mm10ChrX 90077997-90083015 L1 Mus2	3.07	15.64	12.57	5019	687	hg38ChrX 65760414-65765752 L1PA7 r	3.35	12.61	9.25	5339
688	monDom5ChrX 38487768-38492643 L1 Mdo1 result	2.30	20.08	17.78	4876	688	mm10ChrX 117138889-117143908 L1Md	2.51	13.27	10.76	5020	688	hg38ChrX 82253942-82259316 L1PA15	2.53	12.45	9.92	5375
689	monDom5ChrX 27612776-27617654 L1 Mdo2 result	1.97	20.05	18.08	4879	689	mm10ChrX 160569112-160574148 L1Md	2.58	14.55	11.97	5037	689	hg38ChrX 74712707-74718083 L1PA3 r	3.01	14.84	11.83	5377
690	monDom5ChrX 47759415-47764328 L1 Mdo4 result	3.91	18.64	14.73	4914	690	mm10ChrX 44073070-44078116 L1Md F3	3.51	15.18	11.67	5047	690	hg38ChrX 115931094-115936482 L1MA1	3.19	13.32	10.13	5389
691	monDom5ChrX 51600435-51605362 L1 Mdo3a result	3.15	16.11	12.97	4928	691	mm10ChrX 9706137-9711204 L1Md A re	3.04	14.74	11.70	5068	691	hg38ChrX 56075958-56081346 L1PA3 r	2.76	13.73	10.97	5389
692	monDom5ChrX 21491823-21496756 L1 Mdo3 result	2.84	17.82	14.98	4934	692	mm10ChrX 154110908-154115992 L1Md	3.48	15.89	12.41	5085	692	hg38ChrX 56370880-56376277 L1PA3 r	2.83	14.30	11.47	5398
693	monDom5ChrX 72009937-72014897 L1 Mdo3c result	2.34	21.08	18.75	4961	693	mm10ChrX 18898973-18904064 L1Md T	2.53	14.55	12.02	5092	693	hg38ChrX 113446055-113451461 L1PA3	2.64	14.98	12.34	5407
694	monDom5ChrX 20049080-20054131 L1 Mdo4 result	2.51	19.54	17.02	5052	694	mm10ChrX 61886758-61891865 L1 Mus2	3.39	14.19	10.81	5108	694	hg38ChrX 125629049-125634462 L1PB3	2.49	12.97	10.47	5414
695	monDom5ChrX 73903013-73908071 L1 Mdo1 result	1.98	19.75	17.77	5059	695	mm10ChrX 145780346-145785491 L1Md	3.07	14.15	11.08	5146	695	hg38ChrX 25408766-25414189 L1ME4a	3.02	12.89	9.86	5424
696	monDom5ChrX 15007343-15012411 L1 Mdo3c result	2.47	18.09	15.62	5069	696	mm10ChrX 84808146-84813324 L1Md T	3.30	15.91	12.61	5179	696	hg38ChrX 6933212-6938641 L1PA2 res	2.71	14.46	11.75	5430
697	monDom5ChrX 14342907-14347981 L1 Mdo1 result	2.07	20.49	18.42	5075	697	mm10ChrX 79921597-79926782 L1Md A	3.20	14.73	11.53	5186	697	hg38ChrX 82060754-82066238 L1PREC2	3.06	13.20	10.14	5485
698	monDom5ChrX 46656213-46661289 L1 Mdo3c result	2.38	19.62	17.23	5077	698	mm10ChrX 77242385-77247592 L1Md A	2.92	14.92	12.00	5208	698	hg38ChrX 117589280-117594775 L1PA1	2.62	13.70	11.08	5496
699	monDom5ChrX 64204587-64209689 L1 Mdo3a result	2.76	18.22	15.46	5103	699	mm10ChrX 148730631-148735839 L1Md	3.65	15.40	11.75	5209	699	hg38ChrX 106358706-106364205 L1PA1	2.49	11.29	8.80	5500
700	monDom5ChrX 39338182-39343299 L1 Mdo3 result	2.42	20.48	18.05	5118	700	mm10ChrX 80488711-80493919 L1Md F2	3.19	15.01	11.83	5209	700	hg38ChrX 86165198-86170705 L1MA1 r	2.23	14.31	12.07	5508
701	monDom5ChrX 24688442-24693585 L1 Mdo4 result	2.08	20.55	18.47	5144	701	mm10ChrX 91489245-91494455 L1Md F2	3.20	13.97	10.77	5211	701	hg38ChrX 141448495-141454070 L1PA5	2.58	14.71	12.12	5576
702	monDom5ChrX 49391839-49397008 L1 Mdo2 result	2.65	19.52	16.87	5170	702	mm10ChrX 14961334-14966553 L1Md A	3.35	14.27	10.92	5220	702	hg38ChrX 78152446-78158029 L1PA4 r	3.21	12.64	9.44	5584
703	monDom5ChrX 34923948-34929151 NN L1 Mdo4 result	1.88	18.43	16.54	5204	703	mm10ChrX 90168787-90174010 L1Md T	3.62	15.64	12.02	5224	703	hg38ChrX 22000001-22005591 L1P2 re	2.86	13.56	10.70	5591
704	monDom5ChrX 16931052-16936260 L1 Mdo1 result	2.15	20.10	17.95	5209	704	mm10ChrX 41094414-41099639 L1Md A	3.88	15.14	11.25	5226	704	hg38ChrX 79008287-79013878 L1PA7 r	3.38	12.27	8.89	5592
705	monDom5ChrX 22138477-22143707 L1 Mdo1 result	3.33	19.02	15.69	5231	705	mm10ChrX 117690950-117696189 L1Md	3.23	14.87	11.64	5240	705	hg38ChrX 53888927-53894539 L1PA4 r	2.19	14.80	12.61	5613
706	monDom5ChrX 29908953-29914219 L1 Mdo4 result	1.59	19.18	17.58	5267	706	mm10ChrX 58288443-58293683 L1Md T	3.17	15.65	12.48	5241	706	hg38ChrX 139007866-139013479 L1PB1	3.08	12.26	9.17	5614
707	monDom5ChrX 18937001-18942306 L1 Mdo3c result	2.26	18.79	16.53	5306	707	mm10ChrX 23864788-23870029 L1Md F2	3.13	14.15	11.03	5242	707	hg38ChrX 75088142-75093766 L1MC2 r	3.13	13.07	9.94	5625
708	monDom5ChrX 11979935-11985241 NN L1 Mdo3b result	2.51	17.51	15.00	5307	708	mm10ChrX 72143802-72149052 L1Md T	3.05	15.69	12.65	5251	708	hg38ChrX 56464082-56469736 L1PBa r	2.74	13.26	10.52	5655
709	monDom5ChrX 23697170-23702486 L1 Mdo2 result	3.61	20.12	16.51	5317	709	mm10ChrX 35015779-35021043 L1 Mus3	3.30	14.59	11.28	5265	709	hg38ChrX 99266353-99272048 L1PB1 r	2.33	13.15	10.81	5696
710	monDom5ChrX 27138487-27143861 L1 Mdo2 result	2.90	20.07	17.17	5375	710	mm10ChrX 108370501-108375784 L1Md	3.84	14.78	10.94	5284	710	hg38ChrX 81332986-81338702 L1PREC2	4.44	12.45	8.01	5717
711	monDom5ChrX 3834877-3840256 L1-1 MD result	2.49	19.61	17.12	5380	711	mm10ChrX 49825216-49830506 Lx resu	2.89	15.52	12.63	5291	711	hg38ChrX 92715937-92721671 L1PA6 r	2.14	12.50	10.36	5735
712	monDom5ChrX 6159981-6165386 L1-1 MD result	2.59	18.96	16.37	5406	712	mm10ChrX 34016379-34021679 L1Md F2	3.57	14.71	11.15	5301	712	hg38ChrX 109603802-109609551 L1PA7	2.87	14.40	11.53	5750
713	monDom5ChrX 58030736-58036184 L1 Mdo3c result	2.26	17.69	15.43	5449	713	mm10ChrX 16305825-16311143 L1Md F2	3.65	14.80	11.15	5319	713	hg38ChrX 87356335-87362103 L1MA1 r	2.70	14.99	12.29	5769
714	monDomChrX 58892346-58897814 L1 Mdo3a result	2.34	19.49	17.15	5469	714	mm10ChrX 92712244-92717621 L1Md T	2.94	15.49	12.55	5378	714	hg38ChrX 76013974-76019797 L1PB1 r	2.66	13.67	11.01	5824
715	monDom5ChrX 45285763-45291235 L1 Opos0 result	2.23	17.98	15.75	5473	715	mm10ChrX 113351890-113357273 L1Md	3.18	15.17	12.00	5384	715	hg38ChrX 76597102-76602933 L1PA3 r	2.21	14.49	12.28	5832
716	monDom5ChrX 5082161-5087638 L1 Mdo4 result	2.01	20.24	18.24	5478	716	mm10ChrX 132562521-132567932 L1Md	3.16	14.26	11.10	5412	716	hg38ChrX 99771822-99777666 L1MA1 r	2.53	14.41	11.87	5845
717	monDom5ChrX 56192895-56198386 L1 Mdo3 result	2.64	19.45	16.81	5492	717	mm10ChrX 28683919-28689397 L1Md F2	3.38	14.64	11.26	5479	717	hg38ChrX 125500001-125505873 L1M1	2.25	14.00	11.75	5873
718	monDom5ChrX 46210935-46216433 L1 Mdo3a result	2.00	19.95	17.95	5499	718	mm10ChrX 86910453-86915936 L1Md F2	3.12	14.33	11.21	5484	718	hg38ChrX 81881853-81887735 L1PB1 r	2.74	12.24	9.50	5883
719	monDom5ChrX 12238640-12244146 L1 Mdo1 result	2.98	20.70	17.72	5507	719	mm10ChrX 50211683-50217173 L1Md A	2.88	13.59	10.71	5491	719	hg38ChrX 121551322-121557215 L1MA1	2.61	15.22	12.61	5894
720	monDom5ChrX 44355249-44360775 L1 Mdo4 result	1.90	19.16	17.26	5527	720	mm10ChrX 128535980-128541494 Lx3 M	2.77	14.11	11.33	5515	720	hg38ChrX 126770795-126776707 L1PB1	3.11	12.73	9.62	5913
721	monDom5ChrX 29746187-29751724 L1 Mdo5 result	2.78	19.25	16.47	5538	721	mm10ChrX 132070215-132075742 L1 Mu	3.11	14.09	10.98	5528	721	hg38ChrX 141044311-141050237 L1PA5	2.18	13.21	11.03	5927
722	monDom5ChrX 13297892-13303438 L1 Mdo3a result	2.54	19.88	17.34	5547	722	mm10ChrX 156743563-156749120 L1Md	3.17	14.16	10.99	5558	722	hg38ChrX 83232585-83238554 L1PB1 r	2.93	12.75	9.82	5970
723	monDom5ChrX 4168128-4173682 L1 Mdo1 result	2.68	21.42	18.74	5555	723	mm10ChrX 77027483-77033108 L1Md F2	3.23	14.70	11.46	5626	723	hg38ChrX 55046935-55052940 L1PA4 r	2.93	14.00	11.07	6006
724	monDom5ChrX 76484455-76490082 L1 Mdo3b result	2.13	19.01	16.88	5628	724	mm10ChrX 85683818-85689454 L1 Mus3	3.03	13.57	10.54	5637	724	hg38ChrX 51644759-51650770 L1PA3 r	2.46	13.97	11.51	6012
725	monDom5ChrX 66405433-66411064 L1-1 MD result	2.36	18.93	16.57	5632	725	mm10ChrX 79819565-79825238 L1Md T	2.87	14.43	11.56	5674	725	hg38ChrX 114720517-114726531 L1PA2	2.76	14.35	11.59	6015
726	monDom5ChrX 50942097-50947753 L1 Mdo3c result	2.56	18.33	15.77	5657	726	mm10ChrX 154160920-154166643 L1 Mu	3.65	14.48	10.83	5724	726	hg38ChrX 56047266-56053280 L1PA4 r	2.73	13.48	10.76	6015
727	monDom5ChrX 74098539-74104195 L1 Mdo3b result	1.94	18.14	16.19	5657	727	mm10ChrX 50314693-50320449 Lx resu	2.99	15.09	12.11	5757	727	hg38ChrX 62517104-62523125 L1PA3 r	2.56	13.27	10.71	6022
728	monDom5ChrX 6629945-6635623 L1 Mdo4 result	2.03	21.83	19.81	5679	728	mm10ChrX 41231749-41237508 L1Md T	2.97	14.51	11.55	5760	728	hg38ChrX 119435469-119441493 L1HS	2.36	14.06	11.70	6025
729	monDom5ChrX 5378574-5384273 L1 Mdo4 result	2.44	21.00	18.56	5700	729	mm10ChrX 16439999-16445780 L1Md A	2.72	13.11	10.39	5782	729	hg38ChrX 77244268-77250292 L1PA3 r	2.47	13.63	11.15	6025
730	monDom5ChrX 20398267-20403970 L1 Mdo3c result	2.58	20.62	18.04	5704	730	mm10ChrX 151708567-151714371 Lx3C	2.58	15.62	13.04	5805	730	hg38ChrX 26314418-26320446 L1HS re	2.36	14.07	11.71	6029
731	monDom5ChrX 23963489-23969202 L1 Mdo4 result	2.19	19.50	17.31	5714	731	mm10ChrX 65539941-65545760 L1Md A	2.51	12.68	10.17	5820	731	hg38ChrX 42004300-42010329 L1PA3 r	2.64	13.70	11.06	6030
732	monDom5ChrX 24472869-24478622 L1-1 MD result	2.40	19.92	17.52	5754	732	mm10ChrX 18830150-18836015 L1Md F2	3.10	13.71	10.60	5866	732	hg38ChrX 55681154-55687183 L1PA3 r	2.60	13.80	11.19	6030
733	monDom5ChrX 19344032-19349797 L1-1 MD result	2.20	20.36	18.16	5766	733	mm10ChrX 26546451-26552346 L1 Mus1	3.24	13.23	9.99	5896	733	hg38ChrX 67659223-67665252 L1PA4 r	2.97	13.40	10.43	6030
734	monDom5ChrX 8549568-8555376 L1 Mdo4 result	2.32	20.61	18.28	5809	734	mm10ChrX 64971595-64977584 Lx2 res	2.99	16.24	13.26	5990	734	hg38ChrX 56695885-56701916 L1HS re	2.35	14.16	11.80	6032
735	monDom5ChrX 33098150-33103985 L1 Mdo3c result	2.78	20.24	17.46	5836	735	mm10ChrX 127665421-127671411 L1Md	3.99	13.10	9.11	5991	735	hg38ChrX 88207886-88213919 L1PA2 r	2.92	13.87	10.95	6034
736	monDom5ChrX 44360905-44366773 L1 Mdo3c result	1.62	17.99	16.37	5869	736	mm10ChrX 119434118-119440177 L1Md	3.15	13.96	10.81	6060	736	hg38ChrX 19940254-19946296 L1PA2 r	2.50	13.42	10.92	6043
737	monDom5ChrX 76792475-76798374 L1 Mdo3b result	2.66	18.24	15.58	5900	737	mm10ChrX 72577990-72584052 L1Md T	2.69	14.17	11.48	6063	737	hg38ChrX 141421203-141427246 L1HS	2.35	13.90	11.55	6044
738	monDom5ChrX 6656986-6662919 L1 Mdo1 result	2.43	21.69	19.26	5934	738	mm10ChrX 76701435-76707521 L1Md T	3.33	15.18	11.84	6087	738	hg38ChrX 73492040-73498087 L1PA7 r	3.21	13.49	10.28	6048
739	monDom5ChrX 6562267-6568217 L1 Mdo3c result	2.00	20.28	18.28	5951	739	mm10ChrX 119291833-119297924 L1Md	2.99	14.28	11.29	6092	739	hg38ChrX 105959687-105965737 L1PA4	2.61	14.41	11.80	6051
740	monDom5ChrX 23681101-23687059 L1 Mdo4 result	2.42	20.71	18.29	5959	740	mm10ChrX 45572715-45578812 L1Md F2	2.59	14.05	11.46	6098	740	hg38ChrX 83407136-83413200 L1PA5 r	2.09	14.94	12.84	6065
741	monDom5ChrX 74490796-74496757 NN L1-1 MD result	1.91	20.23	18.32	5962	741	mm10ChrX 76253229-76259356 L1Md F2	3.17	14.15	10.98	6128	741	hg38ChrX 63286005-63292073 L1PA4 r	2.57	13.77	11.20	6069
742	monDom5ChrX 57365684-57371658 L1 Mdo1 result	2.44	20.22	17.77	5975	742	mm10ChrX 30941077-30947218 L1Md F2	3.50	14.18	10.68	6142	742	hg38ChrX 81469491-81475588 L1PA6 r	2.62	13.61	10.99	6098
743	monDom5ChrX 31113478-31119456 L1-1 MD result	2.29	20.97	18.68	5979	743	mm10ChrX 67575036-67581182 L1Md F2	3.42	14.38	10.96	6147	743	hg38ChrX 63519157-63525277 L1PA4 r	2.65	13.45	10.80	6121
744	monDom5ChrX 38843336-38849356 L1 Mdo3c result	2.86	19.23	16.38	6021	744	mm10ChrX 47145092-47151279 L1 Mus4	3.41	14.67	11.26	6188	744	hg38ChrX 80650596-80656722 L1PA5 r	2.25	14.69	12.44	6127
745	monDom5ChrX 41799899-41805964 L1 Mdo2 result	2.21	20.01	17.80	6066	745	mm10ChrX 118973271-118979459 L1Md	2.63	14.38	11.75	6189	745	hg38ChrX 131524491-131530619 L1PA5	2.50	14.13	11.63	6129
746	monDom5ChrX 66196070-66202187 L1 Mdo1 result	2.63	19.40	16.77	6118	746	mm10ChrX 79197726-79203914 L1Md F2	3.18	14.07	10.89	6189	746	hg38ChrX 104889805-104895934 L1PA5	2.01	14.03	12.02	6130
747	monDom5ChrX 24442475-24448597 L1 Mdo2 result	2.19	20.84	18.65	6123	747	mm10ChrX 109369029-109375220 L1Md	3.39	15.00	11.61	6192	747	hg38ChrX 70728179-70734314 L1PA4 r	2.54	13.07	10.53	6136
748	monDom5ChrX 49760390-49766542 L1-1 MD result	2.45	17.96	15.50	6153	748	mm10ChrX 63925961-63932152 L1Md A	3.23	14.65	11.42	6192	748	hg38ChrX 85583162-85589299 L1PA5 r	2.51	13.75	11.24	6138
749	monDom5ChrX 30403347-30409512 L1 Mdo2 result	2.77	20.47	17.69	6166	749	mm10ChrX 79610303-79616498 L1Md A	3.05	14.17	11.12	6196	749	hg38ChrX 116159202-116165340 L1PA3	2.69	14.42	11.73	6139
750	monDom5ChrX 24423514-24429684 L1 Mdo4 result	2.97	19.88	16.92	6171	750	mm10ChrX 68607349-68613554 L1Md F2	2.84	14.16	11.33	6206	750	hg38ChrX 34977425-34983563 L1PA13	3.26	12.23	8.98	6139
751	monDom5ChrX 36722230-36728400 L1-1 MD result	2.48	18.17	15.69	6171	751	mm10ChrX 121367333-121373539 L1Md	3.19	14.45	11.26	6207	751	hg38ChrX 69350758-69356897 L1PA5 r	2.83	13.50	10.67	6140
752	monDom5ChrX 59684740-59690959 L1 Mdo2 result	2.99	20.00	17.01	6220	752	mm10ChrX 150861630-150867869 L1Md	3.09	14.79	11.70	6240	752	hg38ChrX 96597641-96603783 L1PA5 r	2.83	14.31	11.48	6143
753	monDom5ChrX 48635927-48642157 L1-1 MD result	2.12	17.22	15.10	6231	753	mm10ChrX 114703746-114709995 L1Md	3.44	15.02	11.58	6250	753	hg38ChrX 52291786-52297932 L1PA4 r	2.91	14.01	11.09	6147
754	monDom5ChrX 60409868-60416109 L1-1 MD result	2.16	18.79	16.63	6242	754	mm10ChrX 90984727-90990976 L1 Mus1	2.90	14.93	12.03	6250	754	hg38ChrX 73093937-73100083 L1PA4 r	2.52	13.83	11.31	6147
755	monDom5ChrX 22264882-22271148 L1-1 MD result	2.43	18.84	16.42	6267	755	mm10ChrX 66123164-66129428 L1Md F2	3.42	14.08	10.66	6265	755	hg38ChrX 63838643-63844790 L1PA3 r	3.11	13.22	10.12	6148
756	monDom5ChrX 66220229-66226501 L1 Mdo2 result	2.69	19.62	16.93	6273	756	mm10ChrX 26807187-26813465 1 Mus1	3.04	13.52	10.48	6279	756	hg38ChrX 132284469-132290617 L1PA5	2.63	13.97	11.34	6149
757	monDom5ChrX 12524956-12531257 L1 Mdo3c result	2.70	19.42	16.72	6302	757	mm10ChrX 31989528-31995814 L1Md F2	3.10	13.97	10.86	6287	757	hg38ChrX 37768612-37774761 L1PA4 r	2.63	13.58	10.94	6150
758	monDom5ChrX 8729590-8735894 L1-1 MD result	1.97	17.11	15.15	6305	758	mm10ChrX 58795285-58801594 L1Md T	3.15	14.88	11.73	6310	758	hg38ChrX 56183881-56190030 L1PA3 r	2.89	13.76	10.86	6150
759	monDom5ChrX 48257085-48263405 L1-1 MD result	2.40	19.33	16.93	6321	759	mm10ChrX 6503938-6510252 L1 Mus1 r	3.53	14.74	11.21	6315	759	hg38ChrX 56581520-56587672 L1PA3 r	2.36	14.51	12.16	6153
760	monDom5ChrX 62966490-62972811 L1-1 MD result	2.36	16.43	14.08	6322	760	mm10ChrX 89892922-89899251 L1Md F2	3.36	13.06	9.70	6330	760	hg38ChrX 110640009-110646163 L1PA4	2.39	13.97	11.58	6155
761	monDom5ChrX 13048142-13054494 L1-1 MD result	2.46	19.27	16.81	6353	761	mm10ChrX 32120049-32126380 L1Md F2	3.36	12.59	9.22	6332	761	hg38ChrX 147200310-147206474 L1PA6	2.11	13.03	10.92	6165
762	monDom5ChrX 60148261-60154613 L1-1 MD result	2.35	19.42	17.08	6353	762	mm10ChrX 53802654-53808995 L1Md A	2.90	13.39	10.49	6342	762	hg38ChrX 55668333-55674513 L1PA7 r	2.67	13.30	10.63	6181
763	monDom5ChrX 18179791-18186144 L1-1 MD result	2.34	19.66	17.31	6354	763	mm10ChrX 85505184-85511572 L1Md T	3.33	13.62	10.28	6389	763	hg38ChrX 68264544-68270753 L1PA5 r	2.74	14.06	11.32	6210
764	monDom5ChrX 20866798-20873153 L1 Mdo3c result	2.77	19.18	16.41	6356	764	mm10ChrX 80502968-80509359 L1Md A	3.65	14.05	10.40	6392	764	hg38ChrX 132445802-132452124 L1PA8	3.59	12.60	9.01	6323
765	monDom5ChrX 61028940-61035295 L1-1 MD result	2.12	19.08	16.96	6356	765	mm10ChrX 4889377-4895777 L1Md T re	2.73	14.47	11.73	6401	765	hg38ChrX 57094342-57100758 L1PA7 r	3.44	12.44	8.99	6417
766	monDom5ChrX 60285426-60291782 L1-1 MD result	2.34	19.27	16.93	6357	766	mm10ChrX 59460148-59466549 L1Md T	3.17	14.34	11.17	6402	766	hg38ChrX 85555431-85561869 L1PA8A	3.29	13.17	9.88	6439
767	monDom5ChrX 71153833-71160192 L1 Mdo4 result	2.33	18.60	16.27	6360	767	mm10ChrX 50098196-50104601 L1Md F2	2.73	13.80	11.07	6406	767	hg38ChrX 95612971-95619412 L1PA7 r	2.81	13.43	10.62	6442
768	monDom5ChrX 66830806-66837192 L1-1 MD result	2.30	19.49	17.19	6387	768	mm10ChrX 80365786-80372191 L1Md A	3.01	13.69	10.68	6406	768	hg38ChrX 73476192-73482637 L1PA7 r	3.09	13.00	9.91	6446
769	monDom5ChrX 61359628-61366030 L1-1 MD result	2.33	19.43	17.10	6403	769	mm10ChrX 9889793-9896202 L1Md A re	2.96	13.35	10.39	6410	769	hg38ChrX 20874272-20880729 L1PA10	3.16	12.54	9.38	6458
770	monDom5ChrX 10835546-10841960 L1 Mdo2 result	4.29	17.46	13.17	6415	770	mm10ChrX 76037931-76044341 L1Md F3	2.70	14.74	12.04	6411	770	hg38ChrX 66718301-66724758 L1PA8 r	3.24	12.02	8.78	6458
771	monDom5ChrX 62489704-62496137 L1 Mdo1 result	2.35	20.42	18.08	6434	771	mm10ChrX 9503134-9509558 L1Md A re	3.08	13.77	10.69	6425	771	hg38ChrX 22866382-22872850 L1PA7 r	2.91	12.77	9.86	6469
772	monDom5ChrX 78466140-78472590 L1 Mdo4 result	2.48	19.00	16.52	6451	772	mm10ChrX 45481753-45488186 L1Md F2	2.70	12.64	9.93	6434	772	hg38ChrX 128041693-128048556 L1PA1	3.66	11.79	8.13	6864
773	monDom5ChrX 76615269-76621763 L1 Mdo1 result	2.46	20.29	17.83	6495	773	mm10ChrX 33869117-33875587 L1Md F2	3.43	14.82	11.39	6471	773	hg38ChrX 86639057-86646066 L1PA14	3.68	12.17	8.49	7010

774	monDom5ChrX 4246532-4253037 L1 Mdo1 result	2.35	19.66	17.31	6506
775	monDom5ChrX 36601421-36607927 L1 Mdo2 result	3.07	18.81	15.74	6507
776	monDom5ChrX 53587813-53594330 L1 Mdo1 result	2.58	19.94	17.37	6518
777	monDom5ChrX 61079447-61086006 L1-1 MD result	2.64	18.20	15.56	6560
778	monDom5ChrX 18423046-18429614 L1 Mdo3c result	2.42	19.81	17.38	6569
779	monDom5ChrX 73958778-73965357 L1-1 MD result	2.89	18.78	15.90	6580
780	monDom5ChrX 12770892-12777490 L1 Mdo2 result	3.17	19.61	16.44	6599
781	monDom5ChrX 42867600-42874202 L1 Mdo5 result	2.26	19.75	17.49	6603
782	monDom5ChrX 46104710-46111317 L1 Mdo3c result	2.07	18.95	16.87	6608
783	monDom5ChrX 27411151-27417785 L1 Mdo3c result	2.05	18.58	16.53	6635
784	monDom5ChrX 28065295-28071939 L1 Mdo3c result	2.00	20.54	18.54	6645
785	monDom5ChrX 16030014-16036691 L1 Mdo3c result	1.99	19.93	17.94	6678
786	monDom5ChrX 40768203-40774884 L1 Mdo1 result	2.83	17.85	15.03	6682
787	monDom5ChrX 13158812-13165503 L1 Mdo4 result	2.47	19.37	16.90	6692
788	monDom5ChrX 8568022-8574718 L1 Mdo1 result	3.49	18.19	14.69	6697
789	monDom5ChrX 52519014-52525728 L1 Mdo4 result	2.49	19.64	17.16	6715
790	monDom5ChrX 50208679-50215402 L1 Mdo1 result	2.54	19.60	17.06	6724
791	monDom5ChrX 20034326-20041058 L1 Mdo1 result	2.15	19.59	17.44	6733
792	monDom5ChrX 45417090-45423836 L1 Mdo4 result	2.55	19.30	16.75	6747
793	monDom5ChrX 42828252-42835021 L1 Mdo2 result	2.23	18.38	16.14	6770
794	monDom5ChrX 15153088-15160107 L1 Mdo2 result	4.07	17.96	13.89	7020
795	monDom5ChrX 54771907-54778938 L1 Mdo2 result	2.50	19.41	16.91	7032
796	monDom5ChrX 61389258-61396314 L1 Mdo2 result	3.07	19.31	16.24	7057
797	monDom5ChrX 62837254-62844510 HAL1 Opos result	2.63	16.43	13.79	7257

774	mm10ChrX 109824910-109831455 L1Md	2.86	13.03	10.17	6546
775	mm10ChrX 135631628-135638203 L1Md	3.22	14.46	11.24	6576
776	mm10ChrX 128946719-128953296 L1Md	3.27	14.02	10.75	6578
777	mm10ChrX 133428383-133434962 L1Md	3.09	14.39	11.31	6580
778	mm10ChrX 121949584-121956235 L1Md	3.05	14.42	11.37	6652
779	mm10ChrX 101823082-101829759 Lx re	3.62	14.63	11.01	6678
780	mm10ChrX 25794924-25801653 L1 Mus1	3.61	14.22	10.61	6730
781	mm10ChrX 30113133-30119916 L1Md A	2.71	12.63	9.92	6784
782	mm10ChrX 26958688-26965592 L1 Mus1	3.30	13.41	10.11	6905
783	mm10ChrX 25022162-25029118 L1 Mus1	3.38	13.51	10.13	6957
784	mm10ChrX 25324511-25331481 L1 Mus1	3.40	13.60	10.20	6971
785	mm10ChrX 22500321-22507312 L1Md A	2.63	12.20	9.57	6992
786	mm10ChrX 80596222-80603239 L1Md A	2.25	10.59	8.34	7018
787	mm10ChrX 150733040-150740066 L1Md	2.90	14.36	11.46	7027
788	mm10ChrX 35501874-35508901 L1Md T	2.79	12.65	9.86	7028
789	mm10ChrX 25590631-25597659 L1 Mus1	3.61	13.74	10.13	7029
790	mm10ChrX 79737866-79744900 L1Md T	2.93	14.17	11.24	7035
791	mm10ChrX 119502567-119509615 L1Md	2.88	14.03	11.15	7049
792	mm10ChrX 155221324-155228375 L1Md	2.88	13.83	10.95	7052
793	mm10ChrX 61251844-61258918 L1Md T	2.87	13.98	11.11	7075
794	mm10ChrX 79855091-79862171 L1Md T	4.46	13.43	8.97	7081
795	mm10ChrX 67373451-67380537 L1Md T	4.33	13.70	9.37	7087
796	mm10ChrX 80823430-80830519 L1Md F2	4.01	13.64	9.63	7090
797	mm10ChrX 21254674-21261764 L1Md T	4.29	13.68	9.39	7091
798	mm10ChrX 73268096-73275199 L1Md T	2.86	13.67	10.81	7104
799	mm10ChrX 61962510-61969624 L1Md T	4.37	13.69	9.32	7115
800	mm10ChrX 21238943-21246066 L1Md T	2.78	14.36	11.58	7124
801	mm10ChrX 75194301-75201424 L1Md T	2.85	13.83	10.98	7124
802	mm10ChrX 68444053-68451196 L1Md F3	2.76	14.04	11.28	7144
803	mm10ChrX 14570278-14577440 L1Md T	2.39	12.69	10.30	7163
804	mm10ChrX 18715566-18722765 L1Md A	2.56	11.85	9.29	7200
805	mm10ChrX 84153348-84160558 L1Md T	2.88	13.53	10.65	7211
806	mm10ChrX 104871694-104878924 L1Md	3.15	15.07	11.92	7231
807	mm10ChrX 61259208-61266477 L1Md T	2.79	14.17	11.38	7270
808	mm10ChrX 46594161-46601433 Lx resu	2.41	14.16	11.76	7273
809	mm10ChrX 64626077-64633369 L1Md T	4.36	13.42	9.06	7293
810	mm10ChrX 40304676-40311979 L1Md T	3.08	13.81	10.73	7304
811	mm10ChrX 86892149-86899464 L1Md T	2.99	14.61	11.62	7316
812	mm10ChrX 114260689-114268026 L1Md	2.77	13.80	11.04	7338
813	mm10ChrX 138663529-138670937 L1Md	2.48	11.59	9.11	7409
814	mm10ChrX 121733064-121740539 L1Md	2.65	13.38	10.73	7476
815	mm10ChrX 43673158-43680650 L1Md T	2.71	13.23	10.52	7493
816	mm10ChrX 15393327-15400853 L1Md T	4.57	13.22	8.65	7527
817	mm10ChrX 141561115-141568691 L1Md	2.68	13.63	10.95	7577
818	mm10ChrX 97960210-97967812 L1Md T	2.67	13.67	11.00	7603
819	mm10ChrX 30241625-30249231 L1Md A	2.50	11.98	9.48	7607
820	mm10ChrX 35316110-35323716 L1Md A	2.60	11.74	9.14	7607
821	mm10ChrX 69930908-69938522 L1Md T	2.67	13.30	10.64	7615
822	mm10ChrX 80959139-80966825 L1Md T	4.70	12.92	8.22	7687
823	mm10ChrX 73300017-73307715 L1Md T	4.97	12.88	7.91	7699
824	mm10ChrX 91835106-91844478 L1Md T	2.17	12.63	10.47	9373

774	hg38ChrX 148272841-148279858 L1PA1	3.58	11.67	8.09	7018
775	hg38ChrX 80300223-80307242 L1MA1 r	2.86	13.79	10.93	7020
776	hg38ChrX 83648725-83655748 L1PA3 r	2.52	13.94	11.42	7024
777	hg38ChrX 102618924-102626033 L1PRE	3.73	11.22	7.50	7110
778	hg38ChrX 140277332-140284445 L1MA2	2.43	13.65	11.22	7114
779	hg38ChrX 84309534-84316657 L1MA1 r	2.04	14.12	12.09	7124
780	hg38ChrX 102405313-102412468 L1MA1	2.64	14.59	11.95	7156
781	hg38ChrX 102274647-102281803 L1MA1	2.64	14.59	11.95	7157
782	hg38ChrX 94607183-94614339 L1MA1 r	3.00	12.27	9.26	7157
783	hg38ChrX 145388046-145395239 L1MA3	2.91	12.15	9.24	7194
784	hg38ChrX 36887119-36894338 L1MA2 r	2.27	12.40	10.12	7220
785	hg38ChrX 93467453-93474683 L1PA16	2.84	10.29	7.45	7231
786	hg38ChrX 79225839-79233079 L1MA2 r	2.80	14.50	11.70	7241
787	hg38ChrX 116799761-116807095 L1MA2	2.48	14.56	12.08	7335
788	hg38ChrX 81083644-81090986 L1PA8 r	2.71	13.01	10.30	7343
789	hg38ChrX 98099898-98107265 L1MA2 r	2.90	12.26	9.35	7368
790	hg38ChrX 57313486-57320856 L1PA7 r	3.27	13.43	10.16	7371
791	hg38ChrX 42336458-42343834 L1PA7 r	3.05	12.78	9.73	7377
792	hg38ChrX 45354127-45361574 L1MA2 r	2.62	14.14	11.52	7448
793	hg38ChrX 51367790-51375287 L1MA1 r	2.24	13.87	11.63	7498
794	hg38ChrX 85231361-85238868 L1MA3 r	2.80	12.11	9.31	7508
795	hg38ChrX 99273192-99280734 L1MA2 r	2.21	12.16	9.94	7543
796	hg38ChrX 117694787-117702345 L1MA1	2.75	12.62	9.87	7559
797	hg38ChrX 113385402-113392971 L1PA1	3.42	12.48	9.06	7570
798	hg38ChrX 109257835-109265406 L1MA2	2.06	13.50	11.44	7572
799	hg38ChrX 82096261-82103835 L1MA1 r	3.06	12.20	9.14	7575
800	hg38ChrX 95238868-95246445 L1MA1 r	3.01	12.68	9.67	7578
801	hg38ChrX 141924917-141932495 L1MA2	2.27	13.45	11.18	7579
802	hg38ChrX 89492299-89499883 L1MA1 r	2.97	13.00	10.03	7585
803	hg38ChrX 106682451-106690042 L1MA3	2.59	14.54	11.95	7592
804	hg38ChrX 23524129-23531723 L1MA2 r	2.92	12.22	9.30	7595
805	hg38ChrX 37319424-37327019 L1MA2 r	2.66	14.34	11.68	7596
806	hg38ChrX 146273378-146280975 L1PA1	3.59	10.53	6.94	7598
807	hg38ChrX 139348447-139356047 L1MA2	2.51	13.52	11.01	7601
808	hg38ChrX 109096390-109104025 L1MA1	2.13	12.21	10.07	7636
809	hg38ChrX 128772434-128780093 L1MA2	2.92	13.90	10.98	7660
810	hg38ChrX 131108096-131115774 L1MA2	3.00	12.74	9.74	7679
811	hg38ChrX 129172373-129180057 L1MA2	2.97	13.34	10.37	7685
812	hg38ChrX 45370651-45378338 L1MA3 r	3.25	13.36	10.11	7688
813	hg38ChrX 82213139-82220837 L1PA13	3.62	12.51	8.88	7699
814	hg38ChrX 42920471-42928181 L1MA3 r	2.67	12.97	10.30	7711
815	hg38ChrX 75397601-75405393 L1PA14	3.44	12.01	8.57	7793
816	hg38ChrX 122357876-122365678 L1PA1	3.36	10.29	6.93	7803
817	hg38ChrX 64457592-64465678 L1PA13	3.75	10.72	6.97	8087
818	hg38ChrX 126004919-126013083 L1MAP	4.35	10.78	6.43	8165

Randomly selected L2s in opossum ChrX

No.	File Name	r-TC (%)	r-AG (%)	r-TC – r-AG	Length
1	monDom5ChrX L2c 33928815-33928860 result	30.43	10.87	19.57	46
2	monDom5ChrX L2 Mars 76773037-76773087 result	25.49	0.00	25.49	51
3	monDom5ChrX L2 Mars 70925077-70925128 result	11.54	0.00	11.54	52
4	monDom5ChrX L2 Mars 20903271-20903329 result	0.00	8.47	-8.47	59
5	monDom5ChrX L2 Mars 26711669-26711728 result	8.33	0.00	8.33	60
6	monDom5ChrX L2c 28401952-28402016 result	12.31	9.23	3.08	65
7	monDom5ChrX L2b 13055853-13055922 result	11.43	0.00	11.43	70
8	monDom5ChrX L2 Mars 30136155-30136225 result	8.45	15.49	-7.04	71
9	monDom5ChrX L2a 70366531-70366602 result	15.28	15.28	0.00	72
10	monDom5ChrX L2c 29226328-29226400 result	19.18	0.00	19.18	73
11	monDom5ChrX L2 Mars 16907778-16907856 result	7.59	0.00	7.59	79
12	monDom5ChrX L2 Mars 33215890-33215977 result	18.18	5.68	12.50	88
13	monDom5ChrX L2 Mars 43593895-43593984 result	11.11	0.00	11.11	90
14	monDom5ChrX L2c 23742196-23742288 result	5.38	0.00	5.38	93
15	monDom5ChrX L2a 28893355-28893450 result	6.25	11.46	-5.21	96
16	monDom5ChrX L2 Mars 31365309-31365405 result	29.90	0.00	29.90	97
17	monDom5ChrX L2 Mars 54101729-54101825 result	46.39	0.00	46.39	97
18	monDom5ChrX L2 Mars 70480618-70480714 result	23.71	5.15	18.56	97
19	monDom5ChrX L2 Mars 39526560-39526658 result	16.16	11.11	5.05	99
20	monDom5ChrX L2b 39574591-39574691 result	7.92	4.95	2.97	101
21	monDom5ChrX L2 55449529-55449630 result	17.65	5.88	11.76	102
22	monDom5ChrX L2 Mars 56127582-56127684 result	5.83	0.00	5.83	103
23	monDom5ChrX L2c 62085603-62085706 result	5.77	4.81	0.96	104
24	monDom5ChrX L2 Mars 34885389-34885493 result	16.19	0.00	16.19	105
25	monDom5ChrX L2 Mars 57162513-57162621 result	14.68	0.00	14.68	109
26	monDom5ChrX L2c 43053395-43053504 result	25.45	0.00	25.45	110
27	monDom5ChrX L2a 65167314-65167424 result	13.51	17.12	-3.60	111
28	monDom5ChrX L2 Mars 53362713-53362824 result	16.07	0.00	16.07	112
29	monDom5ChrX L2 Mars 76091041-76091152 result	8.93	0.00	8.93	112
30	monDom5ChrX L2b 74855067-74855181 result	5.22	6.09	-0.87	115
31	monDom5ChrX L2b 34307943-34308061 result	14.29	5.04	9.24	119
32	monDom5ChrX L2c 9668899-9669019 result	24.79	4.96	19.83	121
33	monDom5ChrX L2 Mars 21139649-21139769 result	12.40	4.13	8.26	121
34	monDom5ChrX L2 Mars 60907243-60907368 result	11.11	7.94	3.17	126
35	monDom5ChrX L2b 56710770-56710896 result	26.77	0.00	26.77	127
36	monDom5ChrX L2 Mars 29981354-29981484 result	14.50	3.82	10.69	131
37	monDom5ChrX L2 Mars 54732682-54732818 result	27.74	0.00	27.74	137
38	monDom5ChrX L2c 65200473-65200618 result	19.18	6.85	12.33	146
39	monDom5ChrX L2 Mars 65066881-65067026 result	18.49	11.64	6.85	146
40	monDom5ChrX L2 Mars 18229676-18229828 result	27.45	3.92	23.53	153
41	monDom5ChrX L2a 51656262-51656416 result	4.52	7.74	-3.23	155
42	monDom5ChrX L2 Mars 7738363-7738518 result	3.85	5.77	-1.92	156
43	monDom5ChrX L2 Mars 67099453-67099609 result	35.67	7.01	28.66	157
44	monDom5ChrX L2 Mars 70812180-70812339 result	31.25	0.00	31.25	160
45	monDom5ChrX L2 Mars 34132652-34132812 result	31.06	0.00	31.06	161
46	monDom5ChrX L2 Mars 73333620-73333803 result	21.74	0.00	21.74	184
47	monDom5ChrX L2 Mars 65771962-65772155 result	27.84	0.00	27.84	194
48	monDom5ChrX L2 56385515-56385713 result	21.61	0.00	21.61	199
49	monDom5ChrX L2 Mars 28605009-28605210 result	20.30	0.00	20.30	202
50	monDom5ChrX L2 Mars 13753367-13753573 result	22.22	0.00	22.22	207
51	monDom5ChrX L2 Mars 45476596-45476802 result	15.94	2.42	13.53	207
52	monDom5ChrX L2 Mars 70955281-70955487 result	22.71	5.31	17.39	207
53	monDom5ChrX L2 Mars 55268576-55268786 result	30.33	2.37	27.96	211
54	monDom5ChrX L2 Mars 12182918-12183129 result	26.89	0.00	26.89	212
55	monDom5ChrX L2 Mars 3352919-3353130 result	18.40	3.30	15.09	212
56	monDom5ChrX L2c 5334001-5334216 result	11.11	0.00	11.11	216
57	monDom5ChrX L2 45438813-45439030 result	23.39	0.00	23.39	218
58	monDom5ChrX L2 Mars 4752141-4752365 result	16.89	2.67	14.22	225
59	monDom5ChrX L2 Mars 12597884-12598109 result	19.47	0.00	19.47	226
60	monDom5ChrX L2c 9230786-9231016 result	35.06	0.00	35.06	231
61	monDom5ChrX L2 Mars 68331594-68331828 result	20.85	0.00	20.85	235
62	monDom5ChrX L2 Mars 11597811-11598047 result	12.66	2.11	10.55	237
63	monDom5ChrX L2 Mars 15330933-15331170 result	20.59	6.72	13.87	238
64	monDom5ChrX L2 Mars 31002635-31002876 result	29.75	2.48	27.27	242
65	monDom5ChrX L2 14402166-14402408 result	27.57	2.06	25.51	243
66	monDom5ChrX L2 Mars 25164475-25164717 result	23.05	0.00	23.05	243
67	monDom5ChrX L2b 42538965-42539220 result	29.30	5.08	24.22	256
68	monDom5ChrX L2 44255046-44255303 result	31.78	2.33	29.46	258
69	monDom5ChrX L2c 57503534-57503793 result	15.77	2.31	13.46	260
70	monDom5ChrX L2c 45605503-45605777 result	26.91	5.82	21.09	275
71	monDom5ChrX L2 Mars 48333803-48334081 result	29.39	1.79	27.60	279
72	monDom5ChrX L2 Mars 45722276-45722558 result	21.20	1.77	19.43	283
73	monDom5ChrX L2 Mars 30871985-30872272 result	31.94	0.00	31.94	288
74	monDom5ChrX L2 Mars 45451893-45452182 result	18.28	1.72	16.55	290
75	monDom5ChrX L2c 78245839-78246129 result	17.53	3.09	14.43	291
76	monDom5ChrX L2 Mars 20942576-20942868 result	25.60	3.75	21.84	293
77	monDom5ChrX L2 Mars 17712675-17712970 result	25.00	3.38	21.62	296
78	monDom5ChrX L2 Mars 60251229-60251526 result	18.46	0.00	18.46	298
79	monDom5ChrX L2 Mars 50220291-50220593 result	19.47	2.31	17.16	303
80	monDom5ChrX L2 Mars 65070746-65071051 result	19.93	6.21	13.73	306
81	monDom5ChrX L2 Mars 19796890-19797196 result	27.04	1.63	25.41	307
82	monDom5ChrX L2b 27521474-27521784 result	26.69	1.61	25.08	311
83	monDom5ChrX L2 Mars 56729109-56729423 result	24.13	4.13	20.00	315
84	monDom5ChrX L2c 46363877-46364192 result	24.68	3.48	21.20	316
85	monDom5ChrX L2 Mars 16599170-16599485 result	23.42	3.48	19.94	316
86	monDom5ChrX L2 Mars 55612005-55612328 result	20.06	0.00	20.06	324
87	monDom5ChrX L2 Mars 3428604-3428932 result	15.50	5.17	10.33	329
88	monDom5ChrX L2 Mars 64160642-64160972 result	22.66	0.00	22.66	331
89	monDom5ChrX L2 Mars 78381147-78381481 result	25.37	1.79	23.58	335
90	monDom5ChrX L2 73344162-73344498 result	26.71	3.26	23.44	337
91	monDom5ChrX L2 Mars 49901700-49902036 result	10.98	0.00	10.98	337
92	monDom5ChrX L2 Mars 60985641-60985977 result	20.77	5.34	15.43	337
93	monDom5ChrX L2 Mars 16242024-16242369 result	20.81	1.73	19.08	346
94	monDom5ChrX L2 Mars 5579373-5579721 result	14.61	5.16	9.46	349
95	monDom5ChrX L2 Mars 72865223-72865573 result	23.36	1.71	21.65	351
96	monDom5ChrX L2 Mars 38190023-38190377 result	11.83	3.10	8.73	355
97	monDom5ChrX L2c 48974309-48974664 result	15.17	1.69	13.48	356
98	monDom5ChrX L2 Mars 27586283-27586640 result	28.21	4.47	23.74	358
99	monDom5ChrX L2 Mars 50491062-50491425 result	22.25	6.59	15.66	364
100	monDom5ChrX L2 Mars 16641451-16641815 result	15.62	4.11	11.51	365
101	monDom5ChrX L2 Mars 13596479-13596846 result	19.84	2.45	17.39	368
102	monDom5ChrX L2 Mars 70972546-70972914 result	29.00	1.63	27.37	369
103	monDom5ChrX L2 Mars 17855726-17856105 result	23.16	2.63	20.53	380
104	monDom5ChrX L2 Mars 70888429-70888810 result	25.65	1.57	24.08	382
105	monDom5ChrX L2 Mars 60962991-60963381 result	23.53	2.81	20.72	391
106	monDom5ChrX L2 Mars 42787193-42787584 result	21.17	0.00	21.17	392
107	monDom5ChrX L2b 41416291-41416685 result	25.57	2.78	22.78	395
108	monDom5ChrX L2 Mars 52053009-52053406 result	23.12	1.51	21.61	398
109	monDom5ChrX L2 Mars 41735126-41735536 result	19.71	0.00	19.71	411
110	monDom5ChrX L2 42294024-42294435 result	20.87	0.00	20.87	412
111	monDom5ChrX L2 Mars 32931410-32931823 result	21.98	0.00	21.98	414
112	monDom5ChrX L2 Mars 54766947-54767360 result	23.67	2.42	21.26	414
113	monDom5ChrX L2 Mars 20246783-20247197 result	12.53	1.20	11.33	415
114	monDom5ChrX L2b 38337042-38337458 result	24.46	2.64	21.82	417
115	monDom5ChrX L2 Mars 39233310-39233727 result	29.67	1.44	28.23	418
116	monDom5ChrX L2 Mars 71688097-71688522 result	22.54	1.41	21.13	426
117	monDom5ChrX L2 Mars 72442099-72442526 result	18.69	2.34	16.36	428
118	monDom5ChrX L2 Mars 76066437-76066866 result	19.07	3.02	16.05	430
119	monDom5ChrX L2 Mars 5206190-5206621 result	14.35	0.00	14.35	432
120	monDom5ChrX L2 Mars 50280827-50281259 result	16.63	4.85	11.78	433
121	monDom5ChrX L2 Mars 34343070-34343505 result	27.52	3.44	24.08	436
122	monDom5ChrX L2 Mars 12550705-12551149 result	20.67	2.25	18.43	445
123	monDom5ChrX L2 Mars 73006762-73007216 result	18.24	1.10	17.14	455
124	monDom5ChrX L2 Mars 63135020-63135476 result	31.07	3.28	27.79	457
125	monDom5ChrX L2 42218592-42219049 result	30.79	3.28	27.51	458
126	monDom5ChrX L2 Mars 74382156-74382617 result	22.94	3.03	19.91	462
127	monDom5ChrX L2 Mars 0594311-30594773 result	27.21	2.16	25.05	463
128	monDom5ChrX L2 53185025-53185489 result	23.23	0.00	23.23	465
129	monDom5ChrX L2 Mars 68025112-68025579 result	31.62	2.56	29.06	468
130	monDom5ChrX L2 Mars 58171481-58171951 result	30.15	0.00	30.15	471
131	monDom5ChrX L2 Mars 49723899-49724370 result	23.94	1.06	22.88	472
132	monDom5ChrX L2 Mars 58004953-58005429 result	20.34	2.10	18.24	477
133	monDom5ChrX L2 Mars 78203145-78203621 result	20.34	2.73	17.61	477
134	monDom5ChrX L2c 6972410-6972897 result	18.85	0.00	18.85	488
135	monDom5ChrX L2 Mars 70140749-70141237 result	15.34	5.73	9.61	489
136	monDom5ChrX L2 Mars 60250353-60250847 result	20.20	0.00	20.20	495
137	monDom5ChrX L2 Mars 7264862-7265357 result	15.93	1.21	14.72	496
138	monDom5ChrX L2 Mars 51164892-51165390 result	22.24	2.61	19.64	499
139	monDom5ChrX L2 Mars 69427492-69427992 result	27.15	2.40	24.75	501
140	monDom5ChrX L2 Mars 3524352-3524856 result	14.46	6.14	8.32	505
141	monDom5ChrX L2 Mars 48607091-48607595 result	11.68	1.58	10.10	505
142	monDom5ChrX L2 Mars 72552320-72552824 result	25.94	0.99	24.95	505
143	monDom5ChrX L2 Mars 36474957-36475462 result	16.60	7.11	9.49	506
144	monDom5ChrX L2 Mars 31411991-31412499 result	21.81	0.00	21.81	509
145	monDom5ChrX L2 Mars 47284325-47284838 result	24.12	1.17	22.96	514
146	monDom5ChrX L2 Mars 52608991-52609506 result	21.32	0.00	21.32	516

Randomly selected L2s in mouse ChrX

No.	File Name	r-TC (%)	r-AG (%)	r-TC – r-AG	Length
1	mm10ChrX L2a 134436177-134436212 r	0.00	13.89	-13.89	36
2	mm10ChrX L2c 59255939-59255976 res	18.42	13.16	5.26	38
3	mm10ChrX L2 68027935-68027979 resu	11.11	0.00	11.11	45
4	mm10ChrX L2c 145186179-145186228 r	0.00	12.00	-12.00	50
5	mm10ChrX L2c 152140154-152140205 r	9.62	15.38	-5.77	52
6	mm10ChrX L2b 73347533-73347588 res	35.71	0.00	35.71	56
7	mm10ChrX L2a 139572078-139572143 r	12.12	10.61	1.52	66
8	mm10ChrX L2a 156509720-156509785 r	7.58	0.00	7.58	66
9	mm10ChrX L2b r165775368-165775449	13.41	0.00	13.41	82
10	mm10ChrX L2a 20350872-20350955 res	5.95	14.29	-8.33	84
11	mm10ChrX L2c 139818752-139818835 r	7.14	5.95	1.19	84
12	mm10ChrX L2a 159563181-159563266 r	13.95	0.00	13.95	86
13	mm10ChrX L2c 141507935-141508026 r	7.61	0.00	7.61	92
14	mm10ChrX L2c 73626952-73627044 res	10.75	0.00	10.75	93
15	mm10ChrX L2b 10271825-10271921 res	17.53	0.00	17.53	97
16	mm10ChrX L2b 42522861-42522958 res	11.22	0.00	11.22	98
17	mm10ChrX L2 134248783-134248881 re	7.07	0.00	7.07	99
18	mm10ChrX L2a 43138475-43138578 res	0.00	4.81	-4.81	104
19	mm10ChrX L2a 156296812-156296919 r	0.00	18.52	-18.52	108
20	mm10ChrX L2c 6318455-6318562 resul	10.19	4.63	5.56	108
21	mm10ChrX L2c 140608439-140608548 r	14.55	5.45	9.09	110
22	mm10ChrX L2c 151117257-151117369 r	4.42	7.08	-2.65	113
23	mm10ChrX L2c 9255747-9255860 resul	7.89	0.00	7.89	114
24	mm10ChrX L2a 69524714-69524828 res	0.00	4.35	-4.35	115
25	mm10ChrX L2c 14189761-14189876 res	14.66	5.17	9.48	116
26	mm10ChrX L2a 152349865-152349981 r	19.66	0.00	19.66	117
27	mm10ChrX L2a 23136406-23136525 res	0.00	5.00	-5.00	120
28	mm10ChrX L2a 99387643-99387770 res	0.00	3.91	-3.91	128
29	mm10ChrX L2c 133463133-133463260 r	10.16	0.00	10.16	128
30	mm10ChrX L2b 99886484-99886614 res	0.00	3.82	-3.82	131
31	mm10ChrX L2a 99502657-99502788 res	10.61	0.00	10.61	132
32	mm10ChrX L2c 48052941-48053074 res	13.43	0.00	13.43	134
33	mm10ChrX L2a 60377982-60378116 res	4.44	9.63	-5.19	135
34	mm10ChrX L2b 47835322-47835457 res	5.15	3.68	1.47	136
35	mm10ChrX L2c 92938924-92939059 res	7.35	0.00	7.35	136
36	mm10ChrX L2a 70896723-70896859 res	22.63	8.76	13.87	137
37	mm10ChrX L2c 95924723-95924872 res	21.33	0.00	21.33	150
38	mm10ChrX L2 104323921-104324077 re	18.47	3.18	15.29	157
39	mm10ChrX L2 63712145-63712302 resu	3.16	4.43	-1.27	158
40	mm10ChrX L2 28932840-28933001 resu	25.93	3.09	22.84	162
41	mm10ChrX L2c 122201083-122201248 r	11.45	0.00	11.45	166
42	mm10ChrX L2a 99489080-99489250 res	18.71	4.09	14.62	171
43	mm10ChrX L2 130840240-130840420 re	13.81	0.00	13.81	181
44	mm10ChrX L2a 135059257-135059440 r	14.67	2.72	11.96	184
45	mm10ChrX L2 55177533-55177718 resu	25.27	0.00	25.27	186
46	mm10ChrX L2 55639594-55639781 resu	25.00	0.00	25.00	188
47	mm10ChrX L2a 99078969-99079160 res	0.00	2.60	-2.60	192
48	mm10ChrX L2c 157161484-157161683 r	6.50	2.50	4.00	200
49	mm10ChrX L2a 68272671-68272873 res	14.78	7.39	7.39	203
50	mm10ChrX L2a 93148442-93148646 res	16.59	0.00	16.59	205
51	mm10ChrX L2 101459396-101459602 re	10.63	2.90	7.73	207
52	mm10ChrX L2 59444764-59444973 resu	10.95	5.71	5.24	210
53	mm10ChrX L2a 42394899-42395111 res	2.35	2.35	0.00	213
54	mm10ChrX L2 64930416-64930630 resu	24.65	6.05	18.60	215
55	mm10ChrX L2 163716277-163716494 re	16.06	3.21	12.84	218
56	mm10ChrX L2 87818155-87818374 resu	16.36	0.00	16.36	220
57	mm10ChrX L2 111748570-111748795 re	3.98	0.00	3.98	226
58	mm10ChrX L2a 73466898-73467126 res	12.23	0.00	12.23	229
59	mm10ChrX L2 64882690-64882919 resu	12.17	2.17	10.00	230
60	mm10ChrX L2 25114089-25114322 resu	16.24	0.00	16.24	234
61	mm10ChrX L2 34874174-34874407 resu	16.24	0.00	16.24	234
62	mm10ChrX L2b 164016128-164016368 r	7.88	4.98	2.90	241
63	mm10ChrX L2 10273785-10274026 resu	11.57	0.00	11.57	242
64	mm10ChrX L2a 7873093-7873339 resul	14.98	0.00	14.98	247
65	mm10ChrX L2 74066895-74067143 resu	14.86	4.82	10.04	249
66	mm10ChrX L2a 16751396-16751644 res	16.87	4.02	12.85	249
67	mm10ChrX L2a 96592575-96592823 res	18.88	2.01	16.87	249
68	mm10ChrX L2c 130472002-130472253 r	15.87	0.00	15.87	252
69	mm10ChrX L2 152124689-152124945 re	14.79	0.00	14.79	257
70	mm10ChrX L2a 161977645-161977901 r	5.45	0.00	5.45	257
71	mm10ChrX L2 45148668-45148927 resu	9.62	3.46	6.15	260
72	mm10ChrX L2 62119671-62119931 resu	10.73	0.00	10.73	261
73	mm10ChrX L2 50429449-50429710 resu	17.18	0.00	17.18	262
74	mm10ChrX L2 161665508-161665797 re	14.83	2.76	12.07	290
75	mm10ChrX L2 57076743-57077038 resu	11.82	2.03	9.80	296
76	mm10ChrX L2 11210685-11210983 resu	5.35	0.00	5.35	299
77	mm10ChrX L2a 14321597-14321895 res	9.70	2.01	7.69	299
78	mm10ChrX L2a 98492529-98492830 res	8.61	1.66	6.95	302
79	mm10ChrX L2a 153179100-153179408 r	11.97	6.47	5.50	309
80	mm10ChrX L2a 23385045-23385353 res	17.15	2.27	14.89	309
81	mm10ChrX L2a 96706046-96706356 res	13.18	0.00	13.18	311
82	mm10ChrX L2a 15374585-15374896 res	12.18	3.21	8.97	312
83	mm10ChrX L2b 72108168-72108481 res	9.55	0.00	9.55	314
84	mm10ChrX L2 61759252-61759566 resu	22.54	1.59	20.95	315
85	mm10ChrX L2c 49284778-49285095 res	10.69	7.86	2.83	318
86	mm10ChrX L2 74430325-74430644 resu	26.88	1.88	25.00	320
87	mm10ChrX L2 36092071-36092393 resu	12.38	1.55	10.84	323
88	mm10ChrX L2a 165244265-165244588 r	25.62	1.85	23.77	324
89	mm10ChrX L2a 71310951-71311275 res	10.15	5.54	4.62	325
90	mm10ChrX L2a 46815060-46815389 res	14.85	3.33	11.52	330
91	mm10ChrX L2b 99352617-99352956 res	10.88	3.82	7.06	340
92	mm10ChrX L2a 165676727-165677067 r	18.48	4.11	14.37	341
93	mm10ChrX L2 137409163-137409504 re	11.70	1.46	10.23	342
94	mm10ChrX L2a 50487091-50487441 res	17.09	1.71	15.38	351
95	mm10ChrX L2 59446992-59447343 resu	12.22	3.13	9.09	352
96	mm10ChrX L2a 129830304-129830659 r	15.45	0.00	15.45	356
97	mm10ChrX L2 144851293-144851649 re	19.89	1.40	18.49	357
98	mm10ChrX L2b 15156182-15156540 res	21.45	1.67	19.78	359
99	mm10ChrX L2c 20654856-20655216 res	14.68	1.39	13.30	361
100	mm10ChrX L2 138188928-138189298 re	14.29	2.16	12.13	371
101	mm10ChrX L2 142803304-142803681 re	12.70	4.76	7.94	378
102	mm10ChrX L2 11210281-11210660 resu	12.63	5.26	7.37	380
103	mm10ChrX L2a 20691986-20692369 res	13.28	3.91	9.38	384
104	mm10ChrX L2 104950924-104951308 re	11.95	1.30	10.65	385
105	mm10ChrX L2b 32998261-32998649 res	13.62	2.06	11.57	389
106	mm10ChrX L2a 118327828-118328221 r	11.42	0.00	11.42	394
107	mm10ChrX L2 46241625-46242022 resu	14.32	1.26	13.07	398
108	mm10ChrX L2a 45994491-45994894 res	18.07	1.24	16.83	404
109	mm10ChrX L2 107337158-107337567 re	14.63	4.63	10.00	410
110	mm10ChrX L2a 144765456-144765875 r	26.43	1.19	25.24	420
111	mm10ChrX L2a 93694251-93694673 res	10.40	2.60	7.80	423
112	mm10ChrX L2a 47488066-47488491 res	15.96	0.00	15.96	426
113	mm10ChrX L2 56991154-56991581 resu	16.36	5.14	11.21	428
114	mm10ChrX L2a 15378383-15378810 res	13.32	4.44	8.88	428
115	mm10ChrX L2c 130633091-130633519 r	10.72	3.03	7.69	429
116	mm10ChrX L2 14729482-14729914 resu	9.24	4.39	4.85	433
117	mm10ChrX L2a 99351907-99352342 res	14.22	1.83	12.39	436
118	mm10ChrX L2a 48360142-48360583 res	12.67	0.00	12.67	442
119	mm10ChrX L2a 69100882-69101324 res	9.71	4.29	5.42	443
120	mm10ChrX L2 131127382-131127826 re	11.01	1.12	9.89	445
121	mm10ChrX L2 143425441-143425887 re	27.29	1.34	25.95	447
122	mm10ChrX L2a 48657237-48657683 res	12.30	1.57	10.74	447
123	mm10ChrX L2a 61801313-61801760 res	15.18	2.68	12.50	448
124	mm10ChrX L2a 7945029-7945478 resul	11.78	1.33	10.44	450
125	mm10ChrX L2 112193534-112193986 re	5.74	4.64	1.10	453
126	mm10ChrX L2a 36670714-36671171 res	19.65	1.31	18.34	458
127	mm10ChrX L2a 140659868-140660326 r	11.55	2.40	9.15	459
128	mm10ChrX L2 59906180-59906646 resu	26.12	0.00	26.12	467
129	mm10ChrX L2a 60614300-60614772 res	12.90	4.86	8.03	473
130	mm10ChrX L2 107644949-107645424 re	9.24	0.00	9.24	476
131	mm10ChrX L2a 48221916-48222395 res	18.75	3.13	15.63	480
132	mm10ChrX L2b 158371173-158371666 r	14.37	0.00	14.37	494
133	mm10ChrX L2 47202016-47202510 resu	10.51	4.44	6.06	495
134	mm10ChrX L2a 7661098-7661592 resul	15.15	1.41	13.74	495
135	mm10ChrX L2a 140153456-140153951 r	10.69	2.42	8.27	496
136	mm10ChrX L2 100912585-100913082 re	17.27	3.82	13.45	498
137	mm10ChrX L2c 160209825-160210322 r	13.05	1.00	12.05	498
138	mm10ChrX L2 52935760-52936261 resu	17.33	1.00	16.33	502
139	mm10ChrX L2 14166189-14166702 resu	14.59	3.50	11.09	514
140	mm10ChrX L2a 10767856-10768378 res	12.43	3.44	8.99	523
141	mm10ChrX L2a 60768323-60768849 res	7.78	2.47	5.31	527
142	mm10ChrX L2a 159036347-159036874 r	13.83	1.14	12.69	528
143	mm10ChrX L2a 33077653-33078180 res	12.69	2.08	10.61	528
144	mm10ChrX L2b 142114331-142114861 r	19.40	1.13	18.27	531
145	mm10ChrX L2 133982935-133983467 re	22.89	0.00	22.89	533
146	mm10ChrX L2a 100414444-100414986 r	9.39	0.00	9.39	543

Randomly selected L2s in human ChrX

No.	File Name	r-TC (%)	r-AG (%)	r-TC – r-AG	Length
1	hg38ChrX L2a 22057295-22057321 res	22.22	0.00	22.22	27
2	hg38ChrX L2b 119497418-119497450 r	18.18	0.00	18.18	33
3	hg38ChrX L2c 78491889-78491922 res	17.65	0.00	17.65	34
4	hg38ChrX L2a 49221663-49221697 res	0.00	14.29	-14.29	35
5	hg38ChrX L2a 68774519-68774563 res	11.11	0.00	11.11	45
6	hg38ChrX L2a 133403615-133403661 r	10.64	0.00	10.64	47
7	hg38ChrX L2c 107340245-107340296 r	44.23	0.00	44.23	52
8	hg38ChrX L2b 152614795-152614849 r	9.09	0.00	9.09	55
9	hg38ChrX L2b 64450588-64450645 res	8.62	0.00	8.62	58
10	hg38ChrX L2a 51412663-51412721 res	0.00	10.17	-10.17	59
11	hg38ChrX L2b 139860987-139861059 r	17.81	6.85	10.96	73
12	hg38ChrX L2a 90074846-90074922 res	6.49	0.00	6.49	77
13	hg38ChrX L2 26797681-26797758 resu	8.97	0.00	8.97	78
14	hg38ChrX L2b 138805956-138806037 r	6.10	0.00	6.10	82
15	hg38ChrX L2c 131144937-131145019 r	6.02	6.02	0.00	83
16	hg38ChrX L2a 111425787-111425870 r	16.67	15.48	1.19	84
17	hg38ChrX L2a 139633183-139633267 r	0.00	7.06	-7.06	85
18	hg38ChrX L2a 68297668-68297752 res	0.00	8.24	-8.24	85
19	hg38ChrX L2a 80092923-80093007 res	5.88	0.00	5.88	85
20	hg38ChrX L2a 16394212-16394299 res	0.00	6.82	-6.82	88
21	hg38ChrX L2b 139396137-139396227 r	13.19	0.00	13.19	91
22	hg38ChrX L2a 119238836-119238935 r	0.00	5.00	-5.00	100
23	hg38ChrX L2a 68999264-68999365 res	4.90	0.00	4.90	102
24	hg38ChrX L2c 124852916-124853023 r	0.00	4.63	-4.63	108
25	hg38ChrX L2 69672367-69672478 resu	6.25	11.61	-5.36	112
26	hg38ChrX L2b 65979865-65979977 res	8.85	0.00	8.85	113
27	hg38ChrX L2b 54323770-54323884 res	6.09	0.00	6.09	115
28	hg38ChrX L2a 111512554-111512669 r	0.00	10.34	-10.34	116
29	hg38ChrX L2b 129000083-129000201 r	5.04	9.24	-4.20	119
30	hg38ChrX L2b 147965537-147965662 r	6.35	9.52	-3.17	126
31	hg38ChrX L2c 30188999-30189125 res	18.11	0.00	18.11	127
32	hg38ChrX L2b 4327880-4328008 resul	4.65	4.65	0.00	129
33	hg38ChrX L2a 48738049-48738178 res	13.08	0.00	13.08	130
34	hg38ChrX L2 93124899-93125035 resu	21.90	4.38	17.52	137
35	hg38ChrX L2c 129433689-129433827 r	5.76	0.00	5.76	139
36	hg38ChrX L2b 146414634-146414773 r	20.71	3.57	17.14	140
37	hg38ChrX L2a 146880464-146880604 r	10.64	7.80	2.84	141
38	hg38ChrX L2c 6472277-6472422 resul	17.12	0.00	17.12	146
39	hg38ChrX L2 40538125-40538274 resu	14.00	0.00	14.00	150
40	hg38ChrX L2b 100962394-100962546 r	18.30	0.00	18.30	153
41	hg38ChrX L2a 97751559-97751714 res	3.85	4.49	-0.64	156
42	hg38ChrX L2a 128882655-128882814 r	13.75	0.00	13.75	160
43	hg38ChrX L2c 124433466-124433629 r	25.61	3.05	22.56	164
44	hg38ChrX L2c 20508945-20509119 res	9.71	3.43	6.29	175
45	hg38ChrX L2b 149032104-149032279 r	17.05	0.00	17.05	176
46	hg38ChrX L2a 138380404-138380588 r	9.73	14.59	-4.86	185
47	hg38ChrX L2a 50935446-50935633 res	2.66	0.00	2.66	188
48	hg38ChrX L2b 119198327-119198514 r	22.34	0.00	22.34	188
49	hg38ChrX L2a 117091445-117091635 r	9.95	3.14	6.81	191
50	hg38ChrX L2b 105625233-105625426 r	9.79	3.61	6.19	194
51	hg38ChrX L2c 30326820-30327019 res	5.50	5.00	0.50	200
52	hg38ChrX L2c 140460602-140460813 r	15.09	0.00	15.09	212
53	hg38ChrX L2a 146222633-146222845 r	18.31	2.35	15.96	213
54	hg38ChrX L2c 111210641-111210856 r	12.50	0.00	12.50	216
55	hg38ChrX L2b 124509118-124509334 r	23.04	3.23	19.82	217
56	hg38ChrX L2 134165670-134165891 re	3.15	3.15	0.00	222
57	hg38ChrX L2c 50141502-50141739 res	15.97	4.20	11.76	238
58	hg38ChrX L2c 97606164-97606405 res	10.74	0.00	10.74	242
59	hg38ChrX L2a 144764439-144764681 r	13.58	2.06	11.52	243
60	hg38ChrX L2 72473799-72474051 resu	15.02	1.98	13.04	253
61	hg38ChrX L2c 10443153-10443413 res	7.28	2.30	4.98	261
62	hg38ChrX L2a 55119452-55119716 res	20.75	0.00	20.75	265
63	hg38ChrX L2 154078618-154078883 re	11.65	0.00	11.65	266
64	hg38ChrX L2c 114654588-114654853 r	10.15	2.26	7.89	266
65	hg38ChrX L2c 22166180-22166451 res	20.96	3.68	17.28	272
66	hg38ChrX L2c 137291283-137291560 r	9.35	0.00	9.35	278
67	hg38ChrX L2c 132044199-132044479 r	17.44	1.78	15.66	281
68	hg38ChrX L2 80918584-80918865 resu	7.45	3.90	3.55	282
69	hg38ChrX L2c 51423069-51423350 res	29.43	1.77	27.66	282
70	hg38ChrX L2c 96246874-96247155 res	13.12	3.55	9.57	282
71	hg38ChrX L2a 89343509-89343792 res	11.97	0.00	11.97	284
72	hg38ChrX L2 53662946-53663230 resu	17.19	2.81	14.39	285
73	hg38ChrX L2 18974854-18975142 resu	21.45	0.00	21.45	289
74	hg38ChrX L2a 91316493-91316782 res	19.66	3.45	16.21	290
75	hg38ChrX L2 21193084-21193375 resu	20.55	4.11	16.44	292
76	hg38ChrX L2a 20181400-20181691 res	13.36	7.19	6.16	292
77	hg38ChrX L2a 63790704-63790996 res	20.48	6.48	13.99	293
78	hg38ChrX L2c 100030012-100030305 r	24.49	0.00	24.49	294
79	hg38ChrX L2c 74928606-74928900 res	7.80	0.00	7.80	295
80	hg38ChrX L2 108008305-108008600 re	13.85	7.77	6.08	296
81	hg38ChrX L2 106944472-106944772 re	21.93	0.00	21.93	301
82	hg38ChrX L2c 142833643-142833944 r	14.24	0.00	14.24	302
83	hg38ChrX L2b 17619752-17620055 res	11.51	4.61	6.91	304
84	hg38ChrX L2c 138749371-138749674 r	9.21	0.00	9.21	304
85	hg38ChrX L2 72549128-72549437 resu	22.26	0.00	22.26	310
86	hg38ChrX L2c 101890519-101890828 r	14.52	0.00	14.52	310
87	hg38ChrX L2c 69519782-69520093 res	25.00	2.24	22.76	312
88	hg38ChrX L2b 16483494-16483807 res	17.83	2.23	15.61	314
89	hg38ChrX L2c 74935642-74935963 res	12.42	1.55	10.87	322
90	hg38ChrX L2b 25610290-25610612 res	19.50	2.79	16.72	323
91	hg38ChrX L2a 13078068-13078392 res	9.85	3.38	6.46	325
92	hg38ChrX L2c 63796132-63796463 res	11.75	0.00	11.75	332
93	hg38ChrX L2 5227850-5228184 result	20.90	1.49	19.40	335
94	hg38ChrX L2a 33736859-33737202 res	11.05	0.00	11.05	344
95	hg38ChrX L2 99362320-99362664 resu	10.43	0.00	10.43	345
96	hg38ChrX L2c 117135132-117135478 r	20.46	1.73	18.73	347
97	hg38ChrX L2a 14329374-14329721 res	15.23	1.44	13.79	348
98	hg38ChrX L2 30357061-30357410 resu	19.43	0.00	19.43	350
99	hg38ChrX L2a 108839992-108840346 r	2.54	0.00	2.54	355
100	hg38ChrX L2c 141314368-141314723 r	14.33	3.37	10.96	356
101	hg38ChrX L2c 39955193-39955550 res	16.76	1.96	14.80	358
102	hg38ChrX L2 52117270-52117628 resu	13.65	0.00	13.65	359
103	hg38ChrX L2a 113193928-113194290 r	12.40	0.00	12.40	363
104	hg38ChrX L2 145484399-145484770 re	10.75	3.23	7.53	372
105	hg38ChrX L2-3 Crp 137416120-137416	17.33	2.93	14.40	375
106	hg38ChrX L2c 95817865-95818244 res	16.32	3.16	13.16	380
107	hg38ChrX L2c 24580930-24581311 res	16.23	4.19	12.04	382
108	hg38ChrX L2c 39267645-39268028 res	14.06	0.00	14.06	384
109	hg38ChrX L2a 24953340-24953734 res	16.96	2.03	14.94	395
110	hg38ChrX L2b 109711545-109711940 r	14.65	1.52	13.13	396
111	hg38ChrX L2c 14715444-14715848 res	17.28	1.23	16.05	405
112	hg38ChrX L2a 44184035-44184440 res	20.94	1.23	19.70	406
113	hg38ChrX L2b 119812395-119812800 r	16.26	1.72	14.53	406
114	hg38ChrX L2a 120586152-120586558 r	11.55	1.23	10.32	407
115	hg38ChrX L2a 42233572-42233978 res	14.99	1.23	13.76	407
116	hg38ChrX L2c 25068085-25068495 res	9.49	2.92	6.57	411
117	hg38ChrX L2b 104999100-104999513 r	9.18	2.66	6.52	414
118	hg38ChrX L2a 131614617-131615035 r	9.07	2.86	6.21	419
119	hg38ChrX L2 87396407-87396826 resu	17.14	0.00	17.14	420
120	hg38ChrX L2b 152424137-152424561 r	6.82	1.18	5.65	425
121	hg38ChrX L2a 123437178-123437604 r	20.14	0.00	20.14	427
122	hg38ChrX L2a 120795943-120796370 r	8.41	6.31	2.10	428
123	hg38ChrX L2a 70223837-70224264 res	7.94	2.34	5.61	428
124	hg38ChrX L2 21892482-21892913 resu	21.30	0.00	21.30	432
125	hg38ChrX L2a 56142185-56142618 res	14.06	2.30	11.75	434
126	hg38ChrX L2c 133639016-133639453 r	12.10	1.14	10.96	438
127	hg38ChrX L2 53771394-53771844 resu	16.19	2.00	14.19	451
128	hg38ChrX L2a 66910101-66910554 res	18.50	0.00	18.50	454
129	hg38ChrX L2a 69263143-69263597 res	18.24	1.10	17.14	455
130	hg38ChrX L2b 18135557-18136011 res	17.36	1.32	16.04	455
131	hg38ChrX L2b 123155518-123155973 r	15.57	0.00	15.57	456
132	hg38ChrX L2 137817555-137818012 re	15.50	2.84	12.66	458
133	hg38ChrX L2a 152213256-152213713 r	10.26	3.93	6.33	458
134	hg38ChrX L2c 141992034-141992491 r	15.07	2.40	12.66	458
135	hg38ChrX L2a 19248801-19249263 res	21.81	0.00	21.81	463
136	hg38ChrX L2a 144121832-144122302 r	12.31	1.27	11.04	471
137	hg38ChrX L2a 27926406-27926878 res	23.68	5.29	18.39	473
138	hg38ChrX L2c 18086393-18086867 res	9.26	3.58	5.68	475
139	hg38ChrX L2a 40120644-40121132 res	15.54	2.25	13.29	489
140	hg38ChrX L2a 54493252-54493740 res	6.13	3.48	2.66	489
141	hg38ChrX L2a 155205316-155205820 r	14.65	0.99	13.66	505
142	hg38ChrX L2c 134042758-134043263 r	18.58	1.98	16.60	506
143	hg38ChrX L2b 150601843-150602354 r	19.53	4.88	14.65	512
144	hg38ChrX L2 149543209-149543725 re	14.12	1.35	12.77	517
145	hg38ChrX L2a 86785190-86785710 res	19.77	2.50	17.27	521
146	hg38ChrX L2a 141178495-141179016 r	11.88	3.64	8.24	522

147	monDom5ChrX L2 Mars 55788341-55788859 result	19.85	0.00	19.85	519
148	monDom5ChrX L2 Mars 13664589-13665108 result	17.69	1.15	16.54	520
149	monDom5ChrX L2 Mars 37789701-37790224 result	22.33	3.63	18.70	524
150	monDom5ChrX L2 Mars 12121755-12122279 result	24.57	2.29	22.29	525
151	monDom5ChrX L2 Mars 66536270-66536797 result	25.00	0.00	25.00	528
152	monDom5ChrX L2 Mars 59218644-59219172 result	23.25	2.46	20.79	529
153	monDom5ChrX L2 Mars 5775699-5776232 result	20.79	1.12	19.66	534
154	monDom5ChrX L2 Mars 48042501-48043039 result	17.81	2.04	15.77	539
155	monDom5ChrX L2 Mars 44516707-44517247 result	15.53	4.07	11.46	541
156	monDom5ChrX L2 Mars 35233273-35233817 result	23.12	2.94	20.18	545
157	monDom5ChrX L2 Mars 60668745-60669291 result	20.84	1.10	19.74	547
158	monDom5ChrX L2 Mars 72210261-72210812 result	26.27	0.00	26.27	552
159	monDom5ChrX L2 5318725-5319277 result	29.66	0.90	28.75	553
160	monDom5ChrX L2 Mars 51177922-51178484 result	24.16	1.24	22.91	563
161	monDom5ChrX L2 Mars 75553887-75554453 result	21.34	3.00	18.34	567
162	monDom5ChrX L2c 46128191-46128759 result	26.36	2.99	23.37	569
163	monDom5ChrX L2 Mars 27278887-27279457 result	33.45	0.00	33.45	571
164	monDom5ChrX L2 Mars 76814032-76814611 result	23.79	0.86	22.93	580
165	monDom5ChrX L2 Mars 39296313-39296894 result	22.85	3.44	19.42	582
166	monDom5ChrX L2 42556936-42557519 result	23.29	0.86	22.43	584
167	monDom5ChrX L2 Mars 4790481-4791066 result	20.31	3.07	17.24	586
168	monDom5ChrX L2 Mars 11428995-11429586 result	22.97	2.20	20.78	592
169	monDom5ChrX L2 Mars 6859771-6860371 result	25.29	0.00	25.29	601
170	monDom5ChrX L2 Mars 32958297-32958900 result	20.70	0.00	20.70	604
171	monDom5ChrX L2 Mars 60171247-60171852 result	21.95	0.83	21.12	606
172	monDom5ChrX L2 Mars 15108698-15109304 result	17.46	2.47	14.99	607
173	monDom5ChrX L2 Mars 29806857-29807466 result	29.84	1.80	28.03	610
174	monDom5ChrX L2 Mars 66690793-66691403 result	19.64	0.00	19.64	611
175	monDom5ChrX L2 Mars 37858116-37858730 result	22.44	0.81	21.63	615
176	monDom5ChrX L2 Mars 64258497-64259113 result	17.99	2.59	15.40	617
177	monDom5ChrX L2 76759165-76759782 result	25.24	4.69	20.55	618
178	monDom5ChrX L2 Mars 48645285-48645902 result	25.24	0.00	25.24	618
179	monDom5ChrX L2 Mars 65084090-65084711 result	18.97	1.77	17.20	622
180	monDom5ChrX L2 Mars 45273542-45274167 result	18.37	0.96	17.41	626
181	monDom5ChrX L2 Mars 55027275-55027902 result	21.02	1.75	19.27	628
182	monDom5ChrX L2 Mars 26708922-26709556 result	21.57	1.89	19.69	635
183	monDom5ChrX L2 Mars 33155641-33156280 result	18.44	2.50	15.94	640
184	monDom5ChrX L2 Mars 15614719-15615369 result	22.73	1.69	21.04	651
185	monDom5ChrX L2 Mars 43739867-43740517 result	20.74	0.77	19.97	651
186	monDom5ChrX L2 Mars 48518181-48518841 result	27.99	2.57	25.42	661
187	monDom5ChrX L2 Mars 42190336-42191002 result	20.24	4.20	16.04	667
188	monDom5ChrX L2 Mars 40084078-40084748 result	21.31	0.00	21.31	671
189	monDom5ChrX L2 Mars 69317203-69317873 result	20.27	1.49	18.78	671
190	monDom5ChrX L2 Mars 34632873-34633547 result	18.67	1.63	17.04	675
191	monDom5ChrX L2 Mars 46673617-46674292 result	24.26	0.00	24.26	676
192	monDom5ChrX L2 Mars 56616844-56617520 result	28.95	3.55	25.41	677
193	monDom5ChrX L2 Mars 36360424-36361108 result	22.63	1.17	21.46	685
194	monDom5ChrX L2 Mars 76700769-76701458 result	13.33	0.00	13.33	690
195	monDom5ChrX L2 Mars 10844729-10845421 result	23.81	1.88	21.93	693
196	monDom5ChrX L2b 78956654-78957348 result	17.41	2.45	14.96	695
197	monDom5ChrX L2 Mars 52632702-52633396 result	23.88	1.58	22.30	695
198	monDom5ChrX L2 Mars 6264517-6265213 result	23.39	0.00	23.39	697
199	monDom5ChrX L2 Mars 42707544-42708243 result	20.00	2.43	17.57	700
200	monDom5ChrX L2c 63269376-63270091 result	19.83	1.54	18.30	716
201	monDom5ChrX L2 Mars 3053996-3054712 result	22.45	1.53	20.92	717
202	monDom5ChrX L2 Mars 32939519-32940235 result	19.25	0.70	18.55	717
203	monDom5ChrX L2 Mars 37302283-37303002 result	24.58	2.08	22.50	720
204	monDom5ChrX L2 Mars 41302570-41303291 result	18.01	2.49	15.51	722
205	monDom5ChrX L2 Mars 30929677-30930406 result	27.95	3.01	24.93	730
206	monDom5ChrX L2 Mars 3582509-3583239 result	21.89	3.83	18.06	731
207	monDom5ChrX L2 Mars 53772883-53773613 result	21.48	1.50	19.97	731
208	monDom5ChrX L2 Mars 24216329-24217060 result	19.40	3.01	16.39	732
209	monDom5ChrX L2 Mars 39957191-39957927 result	24.42	1.36	23.07	737
210	monDom5ChrX L2 Mars 27950302-27951040 result	30.18	0.95	29.23	739
211	monDom5ChrX L2 Mars 7694974-7695714 result	23.35	2.43	20.92	741
212	monDom5ChrX L2 Mars 15612746-15613496 result	22.10	2.93	19.17	751
213	monDom5ChrX L2 Mars 24675122-24675872 result	20.37	0.93	19.44	751
214	monDom5ChrX L2 Mars 49140658-49141408 result	21.84	0.80	21.04	751
215	monDom5ChrX L2 Mars 37566595-37567352 result	17.15	0.66	16.49	758
216	monDom5ChrX L2 Mars 13658660-13659420 result	22.86	1.84	21.02	761
217	monDom5ChrX L2 Mars 31588465-31589227 result	17.30	0.79	16.51	763
218	monDom5ChrX L2 Mars 39615236-39616005 result	25.84	4.03	21.82	770
219	monDom5ChrX L2 Mars 43951552-43952322 result	25.94	0.78	25.16	771
220	monDom5ChrX L2 Mars 66042550-66043323 result	15.50	1.55	13.95	774
221	monDom5ChrX L2 Mars 38556939-38557713 result	21.16	1.68	19.48	775
222	monDom5ChrX L2 Mars 34630431-34631207 result	22.14	1.93	20.21	777
223	monDom5ChrX L2a 41512987-41513772 result	23.66	0.64	23.03	786
224	monDom5ChrX L2 Mars 12490172-12490960 result	24.08	1.27	22.81	789
225	monDom5ChrX L2 Mars 74349237-74350028 result	16.67	1.77	14.90	792
226	monDom5ChrX L2 Mars 34411000-34411792 result	24.21	4.54	19.67	793
227	monDom5ChrX L2 Mars 36595265-36596059 result	22.26	2.77	19.50	795
228	monDom5ChrX L2 Mars 28227056-28227854 result	25.16	0.63	24.53	799
229	monDom5ChrX L2 Mars 65808216-65809018 result	17.56	2.49	15.07	803
230	monDom5ChrX L2 Mars 65393491-65394295 result	29.81	3.48	26.34	805
231	monDom5ChrX L2 Mars 74625135-74625947 result	29.64	2.95	26.69	813
232	monDom5ChrX L2 Mars 39782218-39783038 result	33.01	1.22	31.79	821
233	monDom5ChrX L2 Mars 40292270-40293092 result	26.49	1.46	25.03	823
234	monDom5ChrX L2 Mars 11027887-11028713 result	29.02	1.21	27.81	827
235	monDom5ChrX L2 Mars 59919955-59920781 result	17.05	2.18	14.87	827
236	monDom5ChrX L2 Mars 78289477-78290307 result	23.83	1.20	22.62	831
237	monDom5ChrX L2 Mars 39586597-39587428 result	25.12	1.44	23.68	832
238	monDom5ChrX L2 Mars 65571061-65571892 result	19.47	1.56	17.91	832
239	monDom5ChrX L2 Mars 4545077-4545910 result	24.10	0.72	23.38	834
240	monDom5ChrX L2 Mars 9392123-9392956 result	28.54	0.84	27.70	834
241	monDom5ChrX L2 Mars 23263565-23264402 result	20.29	2.63	17.66	838
242	monDom5ChrX L2 Mars 62180256-62181094 result	24.91	2.03	22.88	839
243	monDom5ChrX L2 Mars 16154265-16155104 result	20.71	1.79	18.93	840
244	monDom5ChrX L2 Mars 34706755-34707596 result	26.25	2.73	23.52	842
245	monDom5ChrX L2 Mars 56617704-56618545 result	27.67	0.71	26.96	842
246	monDom5ChrX L2 Mars 52375616-52376461 result	23.05	1.30	21.75	846
247	monDom5ChrX L2c 17836603-17837449 result	24.79	3.31	21.49	847
248	monDom5ChrX L2 Mars 63120729-63121576 result	20.28	1.89	18.40	848
249	monDom5ChrX L2 Mars 41185633-41186482 result	23.53	2.59	20.94	850
250	monDom5ChrX L2 Mars 12673231-12674097 result	22.26	2.19	20.07	867
251	monDom5ChrX L2 Mars 13469272-13470139 result	23.04	0.58	22.47	868
252	monDom5ChrC L2 Mars 12431878-12432749 result	21.22	2.87	18.35	872
253	monDom5ChrX L2 Mars 35989504-35990377 result	22.54	1.26	21.28	874
254	monDom5ChrX L2 Mars 66019365-66020242 result	27.68	1.94	25.74	878
255	monDom5ChrX L2 Mars 54900887-54901772 result	25.85	3.16	22.69	886
256	monDom5ChrX L2 Mars 17645410-17646296 result	25.82	2.93	22.89	887
257	monDom5ChrX L2 Mars 53246108-53246998 result	23.68	0.90	22.78	891
258	monDom5ChrX L2 Mars 10860868-10861764 result	24.75	2.34	22.41	897
259	monDom5ChrX L2 Mars 49594694-49595593 result	15.78	2.44	13.33	900
260	monDom5ChrX L2 Mars 10298181-10299083 result	22.04	1.22	20.82	903
261	monDom5ChrX L2 Mars 16485553-16486455 result	21.15	1.22	19.93	903
262	monDom5ChrX L2 Mars 61970348-61971254 result	18.08	1.32	16.76	907
263	monDom5ChrX L2 Mars 22025776-22026686 result	16.90	0.55	16.36	911
264	monDom5ChrX L2c 39479822-39480737 result	20.85	2.29	18.56	916
265	monDom5ChrX L2 Mars 62017734-62018651 result	26.58	1.74	24.84	918
266	monDom5ChrX L2 Mars 57254432-57255353 result	24.40	2.93	21.48	922
267	monDom5ChrX L2 Mars 9132986-9133914 result	20.88	2.05	18.84	929
268	monDom5ChrX L2 Mars 72543823-72544752 result	23.66	2.80	20.86	930
269	monDom5ChrX L2 Mars 55920484-55921415 result	22.85	2.04	20.82	932
270	monDom5ChrX L2 Mars 28296881-28297816 result	15.38	1.92	13.46	936
271	monDom5ChrX L2 Mars 15492065-15493003 result	23.64	3.62	20.02	939
272	monDom5ChrX L2 Mars 46506635-46507574 result	24.36	2.23	22.13	940
273	monDom5ChrX L2 Mars 63789637-63790580 result	20.76	0.53	20.23	944
274	monDom5ChrX L2 Mars 77426533-77427477 result	21.06	2.43	18.62	945
275	monDom5ChrX L2 Mars 11555234-11556181 result	20.89	0.74	20.15	948
276	monDom5ChrX L2 Mars 32615966-32616913 result	23.95	0.53	23.42	948
277	monDom5ChrX L2 Mars 41753985-41754940 result	26.57	1.67	24.90	956
278	monDom5ChrX L2 Mars 62369888-62370845 result	22.03	3.03	19.00	958
279	monDom5ChrX L2 Mars 65055205-65056162 result	22.44	0.00	22.44	958
280	monDom5ChrX L2 Mars 54381074-54382032 result	27.01	0.63	26.38	959
281	monDom5ChrX L2 Mars 59872546-59873505 result	32.40	2.50	29.90	960
282	monDom5ChrX L2 Mars 62063589-62064550 result	23.80	2.91	20.89	962
283	monDom5ChrX L2 Mars 68088193-68089159 result	21.51	0.83	20.68	967
284	monDom5ChrX L2 Mars 34590402-34591371 result	21.44	1.65	19.79	970
285	monDom5ChrX L2 Mars 34973741-34974720 result	21.33	1.73	19.59	980
286	monDom5ChrX L2 Mars 27528267-27529250 result	20.53	1.02	19.51	984
287	monDom5ChrX L2 Mars 22086951-22087935 result	26.90	0.81	26.09	985
288	monDom5ChrX L2 Mars 60532888-60533882 result	18.29	0.50	17.79	995
289	monDom5ChrX L2 Mars 38016705-38017704 result	18.50	4.20	14.30	1000
290	monDom5ChrX L2 Mars 73454241-73455240 result	21.70	1.20	20.50	1000
291	monDom5ChrX L2 Mars 13226840-13227864 result	25.17	1.17	24.00	1025
292	monDom5ChrX L2 Mars 18927451-18928490 result	19.33	2.60	16.73	1040
293	monDom5ChrX L2 Mars 72875862-72876919 result	30.91	3.12	27.79	1058
294	monDom5ChrX L2 Mars 70504720-70505792 result	24.70	0.47	24.23	1073
295	monDom5ChrX L2 Mars 63791561-63792652 result	20.97	1.19	19.78	1092
296	monDom5ChrX L2 Mars 52065304-52066437 result	26.72	0.97	25.75	1134
297	monDom5ChrX L2a 68621904-68623040 result	24.45	3.52	20.93	1137
298	monDom5ChrX L2 Mars 76053111-76054247 result	26.39	1.41	24.98	1137
299	monDom5ChrX L2 Mars 54222192-54223345 result	24.78	2.51	22.27	1154
300	monDom5ChrX L2 Mars 4456174-4457335 result	21.43	1.46	19.97	1162
301	monDom5ChrX L2 Mars 17313457-17314666 result	22.81	0.41	22.40	1210
302	monDom5ChrX L2 Mars 68440209-68441427 result	19.93	0.66	19.28	1219
303	monDom5ChrX L2 Mars 55948379-55949607 result	24.90	2.03	22.86	1229
304	monDom5ChrX L2 Mars 62045439-62046670 result	22.89	1.46	21.43	1232
305	monDom5ChrX L2 Mars 60542284-60543516 result	20.36	1.95	18.41	1233
306	monDom5ChrX L2 Mars 61663924-61665165 result	21.01	1.21	19.81	1242
307	monDom5ChrX L2 Mars 59880918-59882173 result	20.22	0.00	20.22	1256
308	monDom5ChrX L2 Mars 53114278-53115559 result	28.78	1.25	27.54	1282
309	monDom5ChrX L2 Mars 45126339-45127636 result	23.57	0.46	23.11	1298
310	monDom5ChrX L2 Mars 77783713-77785010 result	20.34	0.77	19.57	1298
311	monDom5ChrX L2 Mars 45592984-45594301 result	25.49	1.06	24.43	1318
312	monDom5ChrX L2 Mars 21299687-21301184 result	27.17	1.20	25.97	1498
313	monDom5ChrX L2 Mars 62511050-62512560 result	23.10	1.19	21.91	1511
314	monDom5ChrX L2 Mars 52329088-52330668 result	24.29	0.76	23.53	1581
315	monDom5ChrX L2 Mars 42525600-42527194 result	22.95	1.44	21.50	1595
316	monDom5ChrX L2 Mars 4306064-4307697 result	25.09	2.51	22.58	1634
317	monDom5ChrX L2 Mars 11381832-11383486 result	29.06	1.09	27.98	1655
318	monDom5ChrX L2 Mars 39572168-39574000 result	23.68	2.07	21.60	1833
319	monDom5ChrX L2 Mars 63554628-63556647 result	22.57	3.27	19.31	2020
320	monDom5ChrX L2 Mars 59296248-59298271 result	20.80	1.78	19.02	2024
321	monDom5ChrX L2 Mars 52716505-52718587 result	21.80	2.26	19.54	2083
322	monDom5ChrX L2 Mars 4026622-4028711 result	24.64	3.30	21.34	2090
323	monDom5ChrX L2 Mars 75076530-75078626 result	21.03	2.62	18.41	2097
324	monDom5ChrX L2 Mars 53776771-53778880 result	25.40	5.21	20.19	2110
325	monDom5ChrX L2 Mars 59487892-59490020 result	26.35	1.60	24.75	2129
326	monDom5ChrX L2 Mars 16026790-16028922 result	21.85	1.36	20.49	2133
327	monDom5ChrX L2 Mars 53981593-53983842 result	24.31	2.53	21.78	2250
328	monDom5ChrX L2 Mars 13799587-13801998 result	24.46	3.52	20.94	2412
329	monDom5ChrX L2 Mars 35526121-35528539 result	25.67	2.27	23.40	2419
330	monDom5ChrX L2 Mars 35235615-35238076 result	20.23	1.10	19.13	2462
331	monDom5ChrX L2 Mars 52976735-52979289 result	23.37	1.57	21.80	2555
332	monDom5ChrX L2 Mars 7530407-7533007 result	21.11	2.50	18.61	2601
333	monDom5ChrX L2 Mars 58801312-58803938 result	24.70	1.87	22.84	2627

147	mm10ChrX L2b 167315594-167316136 r	16.94	3.31	13.63	543
148	mm10ChrX L2a 35877688-35878233 res	14.47	0.92	13.55	546
149	mm10ChrX L2 87721120-87721685 resu	10.07	2.12	7.95	566
150	mm10ChrX L2a 142826388-142826954 r	12.17	4.06	8.11	567
151	mm10ChrX L2 164674211-164674781 re	9.11	2.10	7.01	571
152	mm10ChrX L2 145404716-145405288 re	8.90	0.00	8.90	573
153	mm10ChrX L2a 69140515-69141087 res	15.71	1.92	13.79	573
154	mm10ChrX L2a 142530406-142530979 r	18.99	2.09	16.90	574
155	mm10ChrX L2a 32218647-32219232 res	13.65	1.88	11.77	586
156	mm10ChrX L2a 3255139-3255724 resul	13.65	1.88	11.77	586
157	mm10ChrX L2a 32919519-32920104 res	14.33	1.88	12.46	586
158	mm10ChrX L2a 4481901-4482486 resul	13.65	1.88	11.77	586
159	mm10ChrX L2a 33456302-33456889 res	13.61	1.87	11.73	588
160	mm10ChrX L2a 33597285-33597872 res	12.59	1.87	10.71	588
161	mm10ChrX L2a 3679718-3680305 resul	14.46	1.87	12.59	588
162	mm10ChrX L2a 5361567-5362154 resul	13.61	1.87	11.73	588
163	mm10ChrX L2a 32459983-32460571 res	13.58	1.87	11.71	589
164	mm10ChrX L2c 39186498-39187086 res	13.58	2.55	11.04	589
165	mm10ChrX L2 140644006-140644598 re	21.42	1.85	19.56	593
166	mm10ChrX L2a 140193581-140194176 r	16.11	2.01	14.09	596
167	mm10ChrX L2 132490732-132491329 re	11.87	2.68	9.20	598
168	mm10ChrX L2a 95556550-95557150 res	16.81	2.66	14.14	601
169	mm10ChrX L2a 99092652-99093253 res	11.30	3.32	7.97	602
170	mm10ChrX L2a 17167243-17167845 res	17.91	5.64	12.27	603
171	mm10ChrX L2a 87833250-87833855 res	16.17	3.30	12.87	606
172	mm10ChrX L2a 31358914-31359521 res	11.18	2.30	8.88	608
173	mm10ChrX L2a 33337675-33338282 res	11.35	2.30	9.05	608
174	mm10ChrX L2a 3729738-3730345 resul	11.18	2.30	8.88	608
175	mm10ChrX L2a 4394960-4395567 resul	11.18	2.30	8.88	608
176	mm10ChrX L2a 153836344-153836953 r	9.51	4.10	5.41	610
177	mm10ChrX L2a 99753510-99754120 res	15.71	5.07	10.64	611
178	mm10ChrX L2a 152349252-152349866 r	11.06	0.00	11.06	615
179	mm10ChrX L2a 99287504-99288121 res	16.50	3.07	13.43	618
180	mm10ChrX L2 66196194-66196821 resu	12.42	1.75	10.67	628
181	mm10ChrX L2a 3350167-3350795 resul	10.81	3.02	7.79	629
182	mm10ChrX L2a 34312307-34312935 res	10.81	3.02	7.79	629
183	mm10ChrX L2a 4608475-4609103 resul	10.81	3.02	7.79	629
184	mm10ChrX L2a 5109894-5110522 resul	10.81	3.02	7.79	629
185	mm10ChrX L2a 56877975-56878609 res	14.17	3.46	10.71	635
186	mm10ChrX L2b 105465088-105465723 r	18.87	2.67	16.19	636
187	mm10ChrX L2a 6534470-6535107 resul	12.38	1.10	11.29	638
188	mm10ChrX L2 165549128-165549766 re	17.37	2.50	14.87	639
189	mm10ChrX L2 108237352-108237991 re	11.25	2.19	9.06	640
190	mm10ChrX L2a 137288998-137289645 r	15.59	1.54	14.04	648
191	mm10ChrX L2 153147695-153148349 re	21.83	1.68	20.15	655
192	mm10ChrX L2c 48358689-48359351 res	22.47	1.66	20.81	663
193	mm10ChrX L2 145428922-145429593 re	17.71	4.17	13.54	672
194	mm10ChrX L2a 142825591-142826262 r	19.20	6.70	12.50	672
195	mm10chrX L2 17284614-17285305 resu	12.43	0.87	11.56	692
196	mm10ChrX L2a 140134920-140135611 r	12.14	3.47	8.67	692
197	mm10ChrX L2a 140196784-140197480 r	12.63	4.59	8.03	697
198	mm10ChrX L2c 143850157-143850858 r	21.79	0.85	20.94	702
199	mm10ChrX L2 130445122-130445829 re	15.54	1.69	13.84	708
200	mm10ChrX L2 144706005-144706721 re	12.55	3.07	9.48	717
201	mm10ChrX L2 102353009-102353726 re	17.97	3.34	14.62	718
202	mm10ChrX L2c 69297296-69298016 res	18.72	1.53	17.20	721
203	mm10ChrX L2a 129477267-129477994 r	11.68	3.02	8.65	728
204	mm10ChrX L2a 69813192-69813921 res	15.34	3.70	11.64	730
205	mm10ChrX L2a 57665206-57665936 res	14.23	2.46	11.76	731
206	mm10ChrX L2 12340610-12341347 resu	12.87	2.30	10.57	738
207	mm10ChrX L2a 161662515-161663254 r	13.24	4.86	8.38	740
208	mm10ChrX L2 59907690-59908437 resu	14.84	2.01	12.83	748
209	mm10ChrX L2a 49779013-49779762 res	13.47	2.93	10.53	750
210	mm10ChrX L2b 143534257-143535006 r	13.60	3.73	9.87	750
211	mm10ChrX L2 57656414-57657165 resu	8.91	3.32	5.59	752
212	mm10ChrX L2a 59954728-59955498 res	15.69	0.65	15.05	771
213	mm10ChrX L2a 69309821-69310593 res	19.40	1.68	17.72	773
214	mm10ChrX L2 122786020-122786801 re	14.83	3.58	11.25	782
215	mm10ChrX L2 131577789-131578582 re	8.82	1.89	6.93	794
216	mm10ChrX L2 41350414-41351214 resu	15.98	3.12	12.86	801
217	mm10ChrX L2a 49390716-49391523 res	14.36	3.96	10.40	808
218	mm10ChrX L2 65220233-65221041 resu	14.46	1.98	12.48	809
219	mm10ChrX L2a 13897409-13898218 res	15.56	0.86	14.69	810
220	mm10ChrX L2 49690144-49690963 resu	12.68	2.32	10.37	820
221	mm10ChrX L2a 93007840-93008669 res	17.71	4.34	13.37	830
222	mm10ChrX L2c 152190935-152191782 r	18.40	4.48	13.92	848
223	mm10ChrX L2a 112371295-112372153 r	11.41	2.56	8.85	859
224	mm10ChrX L2a 17211347-17212207 res	16.14	2.44	13.70	861
225	mm10ChrX L2 14974472-14975356 resu	11.30	2.37	8.93	885
226	mm10ChrX L2 85294574-85295463 resu	16.29	2.92	13.37	890
227	mm10ChrX L2b 12689782-12690680 res	13.90	2.78	11.12	899
228	mm10ChrX L2a 14253737-14254669 res	11.15	3.64	7.50	933
229	mm10ChrX L2a 10272416-10273351 res	15.17	1.07	14.10	936
230	mm10ChrX L2a 85211424-85212392 res	15.27	2.48	12.80	969
231	mm10ChrX L2a 169276287-169277263 r	13.00	1.74	11.26	977
232	mm10ChrX L2 92939499-92940500 resu	14.17	1.90	12.28	1002
233	mm10ChrX L2a 140351569-140352582 r	23.18	1.58	21.60	1014
234	mm10ChrX L2a 99192358-99193372 res	17.44	1.97	15.47	1015
235	mm10ChrX L2 112551452-112552525 re	13.31	1.40	11.92	1074
236	mm10ChrX L2 59101227-59102346 resu	15.00	2.86	12.14	1120
237	mm10ChrX L2a 137217289-137218433 r	10.48	2.53	7.95	1145
238	mm10ChrX L2a 165713145-165714312 r	19.26	2.40	16.87	1168
239	mm10ChrX L2a 50787339-50788513 res	15.23	3.57	11.66	1175
240	mm10ChrX L2a 13628344-13629521 res	15.11	2.29	12.82	1178
241	mm10ChrX L2 155807692-155808952 re	15.78	1.51	14.27	1261

147	hg38ChrX L2 112625326-112625848 re	15.68	1.91	13.77	523
148	hg38ChrX L2c 123077614-123078136 r	16.25	6.31	9.94	523
149	hg38ChrX L2c 69728258-69728780 res	14.91	0.96	13.96	523
150	hg38ChrX L2a 32694008-32694531 res	16.79	2.10	14.69	524
151	hg38ChrX L2 135212815-135213342 re	13.83	1.89	11.93	528
152	hg38ChrX L2c 119227185-119227713 r	19.09	3.59	15.50	529
153	hg38ChrX L2c 27176719-27177249 res	11.49	0.94	10.55	531
154	hg38ChrX L2c 20681454-20681988 res	15.33	0.93	14.39	535
155	hg38ChrX L2 81213838-81214373 resu	24.81	0.00	24.81	536
156	hg38ChrX L2b 129684685-129685230 r	18.68	2.20	16.48	546
157	hg38ChrX L2a 109775303-109775849 r	8.96	2.01	6.95	547
158	hg38ChrX L2 98387986-98388545 resu	15.18	0.00	15.18	560
159	hg38ChrX L2c 107461171-107461731 r	14.44	3.03	11.41	561
160	hg38ChrX L2c 142583812-142584372 r	13.01	3.03	9.98	561
161	hg38ChrX L2a 148311751-148312316 r	15.37	1.06	14.31	566
162	hg38ChrX L2c 144351852-144352429 r	12.46	4.84	7.61	578
163	hg38ChrX L2c 128950248-128950826 r	10.19	2.59	7.60	579
164	hg38ChrX L2a 106699444-106700028 r	14.53	1.20	13.33	585
165	hg38ChrX L2c 107128434-107129019 r	12.12	2.05	10.07	586
166	hg38ChrX L2a 134698548-134699135 r	12.93	2.38	10.54	588
167	hg38ChrX L2a 98403743-98404332 res	16.61	2.03	14.58	590
168	hg38ChrX L2-3 Crp 136554969-136555	26.43	3.87	22.56	594
169	hg38ChrX L2a 148712733-148713327 r	9.92	2.52	7.39	595
170	hg38ChrX L2a 34675487-34676084 res	17.89	0.00	17.89	598
171	hg38ChrX L2c 73570356-73570955 res	21.00	0.00	21.00	600
172	hg38ChrX L2a 39943682-39944286 res	9.92	1.82	8.10	605
173	hg38ChrX L2b 64971199-64971803 res	15.54	0.99	14.55	605
174	hg38ChrX L2a 51084190-51084795 res	17.99	2.15	15.84	606
175	hg38ChrX L2b 109401547-109402153 r	13.67	3.95	9.72	607
176	hg38ChrX L2c 20807595-20808205 res	18.82	0.00	18.82	611
177	hg38ChrX L2 72356886-72357502 resu	17.50	1.78	15.72	617
178	hg38ChrX L2a 15566852-15567469 res	17.64	0.97	16.67	618
179	hg38ChrX L2a 142451786-142452406 r	11.27	0.00	11.27	621
180	hg38ChrX L2a 32810267-32810888 res	11.41	0.00	11.41	622
181	hg38ChrX L2a 34063201-34063822 res	13.50	2.41	11.09	622
182	hg38ChrX L2c 91481974-91482599 res	17.25	3.51	13.74	626
183	hg38ChrX L2a 146208125-146208752 r	9.87	0.80	9.08	628
184	hg38ChrX L2b 79096926-79097555 res	12.54	2.70	9.84	630
185	hg38ChrX L2 57380125-57380756 resu	15.19	3.96	11.23	632
186	hg38ChrX L2a 101662727-101663365 r	16.74	4.54	12.21	639
187	hg38ChrX L2 53845033-53845672 resu	17.34	3.28	14.06	640
188	hg38ChrX L2 52339925-52340565 resu	15.91	5.93	9.98	641
189	hg38ChrX L2a 92123492-92124143 res	15.64	0.77	14.88	652
190	hg38ChrX L2 27405733-27406385 resu	16.85	4.44	12.40	653
191	hg38ChrX L2c 124096010-124096664 r	14.96	2.14	12.82	655
192	hg38ChrX L2c 34815462-34816118 res	7.76	3.81	3.96	657
193	hg38ChrX L2a 147299524-147300181 r	16.72	1.06	15.65	658
194	hg38ChrX L2a 152615630-152616287 r	20.97	1.67	19.30	658
195	hg38ChrX L2a 123243156-123243816 r	12.41	2.42	9.98	661
196	hg38ChrX L2 110434111-110434772 re	10.57	0.76	9.82	662
197	hg38ChrX L2a 98199313-98199985 res	10.40	0.74	9.66	673
198	hg38ChrX L2a 96733067-96733750 res	10.53	1.61	8.92	684
199	hg38ChrX L2a 154138818-154139502 r	10.07	2.92	7.15	685
200	hg38ChrX L2c 106429738-106430424 r	17.61	2.33	15.28	687
201	hg38ChrX L2b 30369117-30369816 res	14.14	0.71	13.43	700
202	hg38ChrX L2a 42443190-42443891 res	11.97	0.71	11.25	702
203	hg38ChrX L2a 67129998-67130700 res	17.64	3.70	13.94	703
204	hg38ChrX L2a 140367754-140368457 r	17.47	4.55	12.93	704
205	hg38ChrX L2a 76087995-76088700 res	11.90	2.12	9.77	706
206	hg38ChrX L2c 127651164-127651869 r	10.20	1.70	8.50	706
207	hg38ChrX L2b 153463355-153464061 r	9.90	0.71	9.19	707
208	hg38ChrX L2b 111155692-111156399 r	13.84	3.11	10.73	708
209	hg38ChrX L2b 132198181-132198894 r	16.11	3.50	12.61	714
210	hg38ChrX L2a 14619172-14619893 res	9.28	0.69	8.59	722
211	hg38ChrX L2 75750902-75751627 resu	11.02	3.03	7.99	726
212	hg38ChrX L2a 117706225-117706959 r	9.25	0.68	8.57	735
213	hg38ChrX L2 93746906-93747641 resu	14.95	1.49	13.45	736
214	hg38ChrX L2a 37824257-37824994 res	15.85	2.30	13.55	738
215	hg38ChrX L2a 97992935-97993677 res	16.82	1.62	15.21	743
216	hg38ChrX L2 41764198-41764941 resu	21.64	2.28	19.35	744
217	hg38ChrX L2a 92155927-92156670 res	15.46	4.30	11.16	744
218	hg38ChrX L2b 16352106-16352852 res	17.27	3.75	13.52	747
219	hg38ChrX L2a 113288917-113289664 r	13.64	0.67	12.97	748
220	hg38ChrX L2a 148036789-148037536 r	17.11	0.00	17.11	748
221	hg38ChrX L2a 48924230-48924983 res	7.43	1.46	5.97	754
222	hg38ChrX L2a 138305140-138305905 r	21.54	0.91	20.63	766
223	hg38ChrX L2c 77986392-77987158 res	16.69	1.30	15.38	767
224	hg38ChrX L2a 47678757-47679525 res	15.21	4.16	11.05	769
225	hg38ChrX L2a 138304026-138304797 r	17.88	1.68	16.19	772
226	hg38ChrX L2b 150603353-150604124 r	17.62	1.42	16.19	772
227	hg38ChrX L2c 118668543-118669317 r	14.84	0.65	14.19	775
228	hg38ChrX L2 54025295-54026071 resu	12.48	2.32	10.17	777
229	hg38ChrX L2 138668433-138669215 re	19.54	0.00	19.54	783
230	hg38ChrX L2a 104210161-104210958 r	21.93	1.38	20.55	798
231	hg38ChrX L2 147810471-147811273 re	16.19	0.00	16.19	803
232	hg38ChrX L2c 127642766-127643568 r	16.94	0.62	16.31	803
233	hg38ChrX L2 102519001-102519804 re	16.04	4.98	11.07	804
234	hg38ChrX L2c 38294513-38295318 res	24.07	1.99	22.08	806
235	hg38ChrX L2c 45842520-45843325 res	17.37	0.00	17.37	806
236	hg38ChrX L2 134146141-134146953 re	14.51	4.92	9.59	813
237	hg38ChrX L2b 64439980-64440792 res	15.50	4.31	11.19	813
238	hg38ChrX L2 26711552-26712365 resu	13.64	3.56	10.07	814
239	hg38ChrX L2b 112651395-112652209 r	19.26	3.68	15.58	815
240	hg38ChrX L2a 136793708-136794524 r	15.54	3.30	12.24	817
241	hg38ChrX L2b 69526485-69527301 res	13.71	3.06	10.65	817
242	hg38ChrX L2a 19681421-19682238 res	13.94	1.96	11.98	818
243	hg38ChrX L2 72999147-72999966 resu	17.20	2.20	15.00	820
244	hg38ChrX L2c 131171595-131172415 r	9.87	2.92	6.94	821
245	hg38ChrX L2 90703825-90704649 resu	13.94	0.61	13.33	825
246	hg38ChrX L2b 56753373-56754197 res	24.97	1.21	23.76	825
247	hg38ChrX L2a 6061747-6062573 resul	12.82	1.21	11.61	827
248	hg38ChrX L2a 10652303-10653130 res	17.27	1.93	15.34	828
249	hg38ChrX L2 96741784-96742612 resu	15.44	1.45	13.99	829
250	hg38ChrX L2c 123461206-123462039 r	12.83	0.72	12.11	834
251	hg38ChrX L2c 131550828-131551662 r	13.89	3.23	10.66	835
252	hg38CrX L2a 101399655-101400489 re	12.46	1.44	11.02	835
253	hg38ChrX L2a 90811948-90812795 res	15.21	4.01	11.20	848
254	hg38ChrX L2c 8386874-8387724 resul	19.74	2.12	17.63	851
255	hg38ChrX L2a 151980513-151981366 r	18.85	3.40	15.46	854
256	hg38ChrX L2a 140065716-140066582 r	17.99	1.85	16.15	867
257	hg38ChrX L2c 105289165-105290045 r	14.98	0.57	14.42	881
258	hg38ChrX L2b 98496353-98497234 res	12.47	2.15	10.32	882
259	hg38ChrX L2 40641884-40642767 resu	20.02	0.79	19.23	884
260	hg38ChrX L2c 138187197-138188082 r	21.78	3.50	18.28	886
261	hg38ChrX L2 105202514-105203415 re	12.08	3.55	8.54	902
262	hg38ChrX L2c 147789780-147790681 r	21.95	0.00	21.95	902
263	hg38ChrX L2 153068590-153069494 re	20.88	0.00	20.88	905
264	hg38ChrX L2a 148932814-148933722 r	17.27	1.32	15.95	909
265	hg38ChrX L2 104324970-104325885 re	19.10	1.75	17.36	916
266	hg38ChrX L2a 151750442-151751358 r	13.96	1.85	12.10	917
267	hg38ChrX L2a 110957792-110958709 r	14.38	1.31	13.07	918
268	hg38ChrX L2a 104378614-104379532 r	18.93	1.20	17.74	919
269	hg38ChrX L2a 140351964-140352883 r	15.00	2.17	12.83	920
270	hg38ChrX L2a 106416993-106417914 r	17.46	0.54	16.92	922
271	hg38ChrX L2a 19110887-19111813 res	21.57	1.29	20.28	927
272	hg38ChrX L2b 140464196-140465122 r	11.33	1.83	9.49	927
273	hg38ChrX L2c 78852535-78853461 res	13.48	2.16	11.33	927
274	hg38ChrX L2a 124994490-124995422 r	15.11	1.93	13.18	933
275	hg38ChrX L2a 29884563-29885502 res	16.70	1.17	15.53	940
276	hg38ChrX L2a 141827361-141828301 r	14.03	0.85	13.18	941
277	hg38ChrX L2a 127081053-127081995 r	13.36	1.59	11.77	943
278	hg38ChrX L2 38465486-38466430 resu	23.39	1.48	21.90	945
279	hg38ChrX L2c 116005789-116006734 r	10.04	4.33	5.71	946
280	hg38ChrX L2a 45293561-45294510 res	18.95	2.32	16.63	950
281	hg38ChrX L2 136519144-136520097 re	18.34	1.68	16.67	954
282	hg38ChrX L2 96924949-96925902 resu	19.08	1.05	18.03	954
283	hg38ChrX L2a 42129482-42130445 res	9.44	2.49	6.95	964
284	hg38ChrX L2c 152594862-152595826 r	16.17	1.45	14.72	965
285	hg38ChrX L2 145770150-145771118 re	14.14	2.06	12.07	969
286	hg38ChrX L2 83862724-83863697 resu	15.91	0.62	15.30	974
287	hg38ChrX L2c 117549767-117550745 r	19.31	2.35	16.96	979
288	hg38ChrX L2a 51320320-51321299 res	10.92	2.24	8.67	980
289	hg38ChrX L2a 51511223-51512211 res	13.14	2.53	10.62	989
290	hg38ChrX L2a 142760240-142761234 r	15.08	0.70	14.37	995
291	hg38ChrX L2a 135196833-135197837 r	13.53	2.99	10.55	1005
292	hg38ChrX L2c 45561235-45562239 res	17.01	3.58	13.43	1005
293	hg38ChrX L2a 124794201-124795216 r	13.19	2.07	11.12	1016
294	hg38ChrX L2a 32133305-32134320 res	12.50	3.15	9.35	1016
295	hg38ChrX L2c 68116526-68117547 res	10.76	1.96	8.81	1022
296	hg38ChrX L2 97046157-97047186 resu	11.84	3.50	8.35	1030
297	hg38ChrX L2 105573358-105574390 re	21.10	2.13	18.97	1033
298	hg38ChrX L2c 85993013-85994073 res	10.84	3.02	7.82	1061
299	hg38ChrX L2a 135173278-135174344 r	18.65	4.03	14.62	1067
300	hg38ChrX L2b 140384729-140385798 r	18.41	0.65	17.76	1070
301	hg38ChrX L2a 90810733-90811808 res	12.55	1.39	11.15	1076
302	hg38ChrX L2c 32124564-32125666 res	14.69	1.81	12.87	1103
303	hg38ChrX L2 32456649-32457757 resu	14.34	3.16	11.18	1109
304	hg38ChrX L2a 49912695-49913816 res	18.18	2.41	15.78	1122
305	hg38ChrX L2 78785289-78786435 resu	17.61	2.27	15.34	1147
306	hg38ChrX L2c 86022479-86023631 res	16.31	0.00	16.31	1153
307	hg38ChrX L2a 31112496-31113669 res	13.80	0.94	12.86	1174
308	hg38ChrX L2a 124417512-124418691 r	11.19	0.59	10.59	1180
309	hg38ChrX L2 39258604-39259784 resu	21.08	1.95	19.14	1181
310	hg38ChrX L2a 28975587-28976831 res	22.81	2.17	20.64	1245
311	hg38ChrX L2a 33860518-33861780 res	12.59	1.11	11.48	1263
312	hg38ChrX L2a 144271437-144272722 r	15.55	4.04	11.51	1286
313	hg38ChrX L2 110163676-110164971 re	12.58	1.62	10.96	1296
314	hg38ChrX L2 25246100-25247449 resu	13.70	1.70	12.00	1350
315	hg38ChrX L2 145693114-145694473 re	11.99	1.99	10.00	1360
316	hg38ChrX L2a 87983960-87985340 res	15.13	3.55	11.59	1381
317	hg38ChrX L2a 115474870-115476344 r	12.20	3.39	8.81	1475
318	hg38ChrX L2 49959525-49961009 resu	16.57	2.56	14.01	1485
319	hg38ChrX L2b 42086143-42087718 res	16.75	1.02	15.74	1576
320	hg38ChrX L2a 14694795-14696769 res	17.06	0.35	16.71	1975
321	hg38ChrX L2 109218072-109220085 re	17.92	2.73	15.19	2014
322	hg38ChrX L2 110138406-110140454 re	16.01	3.71	12.30	2049
323	hg38ChrX L2c 37754139-37756200 res	16.49	3.64	12.85	2062
324	hg38ChrX L2a 104088593-104090708 r	16.12	1.98	14.13	2116
325	hg38ChrX L2a 151689820-151691958 r	14.35	2.15	12.20	2139
326	hg38ChrX L2a 25201238-25203444 res	16.67	3.22	13.46	2207
327	hg38ChrX L2c 111134490-111136769 r	17.76	0.75	17.02	2280
328	hg38ChrX L2a 95965915-95968212 res	16.14	2.57	13.58	2298
329	hg38ChrX L2 36333071-36335393 resu	15.54	1.55	13.99	2323
330	hg38ChrX L2a 140903936-140906369 r	14.91	1.44	13.48	2434
331	hg38ChrX L2a 140142611-140145056 r	15.54	1.59	13.94	2446
332	hg38ChrX L2b 112093513-112096040 r	17.29	2.22	15.07	2528
333	hg38ChrX L2c 130587743-130590333 r	16.06	1.81	14.24	2591
334	hg38ChrX L2c 64332359-64335019 res	17.62	2.41	15.22	2661

Randomly selected L3s in opossum ChrX

No.	File Name	r-TC (%)	r-AG (%)	r-TC – r-AG	Length
1	monDom5ChrX L3_Mars 37345210-37345235_result	0.00	19.23	19.23	26
2	monDom5ChrX Plat L3 66777706-66777750 result	28.89	0.00	-28.89	45
3	monDom5ChrX L3 Mars 32238238-32238284 result	10.64	12.77	2.13	47
4	monDom5ChrX L3 Mars 42393328-42393376 result	0.00	32.65	32.65	49
5	monDom5ChrX L3b 47332253-47332304 result	0.00	13.46	13.46	52
6	monDom5ChrX L3 Mars 49875182-49875233 result	9.62	0.00	-9.62	52
7	monDom5ChrX L3 Mars 16699394-16699447 result	9.26	9.26	0.00	54
8	monDom5ChrX Plat L3 19761508-19761564 result	22.81	0.00	-22.81	57
9	monDom5ChrX Plat L3 42980021-42980079 result	10.17	30.51	20.34	59

Randomly selected RTEs in opossum ChrX

No.	File Name	r-TC (%)	r-AG (%)	r-TC – r-AG	Length
1	monDom5ChrX RTE1 Mars 55121684-551	0.00	23.33	23.33	30
2	monDom5ChrX RTE1 Mars 33976718-339	13.04	19.57	6.52	46
3	monDom5ChrX RTE1 Mars 71681156-716	0.00	13.46	13.46	52
4	monDom5ChrX RTE1 Mars 18826271-188	13.21	0.00	-13.21	53
5	monDom5ChrX RTE1 Mars 50188515-501	0.00	12.73	12.73	55
6	monDom5ChrX RTE1 Mars 37989265-379	0.00	43.86	43.86	57
7	monDom5ChrX RTE1 Mars 39715985-397	23.44	18.75	-4.69	64
8	monDom5ChrX RTE1 Mars 75655402-756	7.58	7.58	0.00	66
9	monDom5ChrX RTE1 Mars 32670452-326	0.00	10.29	10.29	68

10	monDom5ChrX L3 Mars 59199060-59199121 result	8.06	0.00	-8.06	62
11	monDom5ChrX L3 69723035-69723102 result	0.00	8.82	8.82	68
12	monDom5ChrX L3 9493316-9493389 result	0.00	25.68	25.68	74
13	monDom5ChrX L3 Mars 50395299-50395372 result	8.11	0.00	-8.11	74
14	monDom5ChrX L3 27090539-27090614 result	0.00	30.26	30.26	76
15	monDom5ChrX L3 Mars 67370238-67370313 result	18.42	6.58	-11.84	76
16	monDom5ChrX L3 Mars 56615193-56615269 result	0.00	15.58	15.58	77
17	monDom5ChrX L3 Mars 45450937-45451014 result	17.95	0.00	-17.95	78
18	monDom5ChrX L3 Mars 50228208-50228285 result	0.00	6.41	6.41	78
19	monDom5ChrX L3 Mars 26849628-26849706 result	0.00	12.66	12.66	79
20	monDom5ChrX L3 59962401-59962480 result	0.00	17.50	17.50	80
21	monDom5ChrX L3 Mars 49046578-49046659 result	0.00	12.20	12.20	82
22	monDom5ChrX L3 Mars 53574194-53574275 result	17.07	34.15	17.07	82
23	monDom5ChrX L3 Mars 10404896-10404982 result	5.75	20.69	14.94	87
24	monDom5ChrX L3 Mars 51925533-51925622 result	6.67	6.67	0.00	90
25	monDom5ChrX L3 Mars 78350407-78350498 result	10.87	19.57	8.70	92
26	monDom5ChrX L3 Mars 43475679-43475771 result	0.00	16.13	16.13	93
27	monDom5ChrX L3 Mars 71799450-71799544 result	0.00	16.84	16.84	95
28	monDom5ChrX Plat L3 29608685-2960878 result	0.00	31.96	31.96	97
29	monDom5ChrX Plat L3 45389591-45389689 result	6.06	17.17	11.11	99
30	monDom5ChrX L3 Mars 43859909-43860011 result	4.85	18.45	13.59	103
31	monDom5ChrX L3 Mars 6589558-6589661 result	0.00	24.04	24.04	104
32	monDom5ChrX L3 Mars 65738016-65738122 result	0.00	12.15	12.15	107
33	monDom5ChrX L3 48035711-48035819 result	6.42	26.61	20.18	109
34	monDom5ChrX L3 30514860-30514969 result	4.55	5.45	0.91	110
35	monDom5ChrX L3 Mars 52608154-52608264 result	0.00	8.11	8.11	111
36	monDom5ChrX L3b 34745644-34745757 result	6.14	24.56	18.42	114
37	monDom5ChrX L3 Mars 41162636-41162751 result	15.52	4.31	-11.21	116
38	monDom5ChrX L3 78391762-78391879 result	4.24	16.95	12.71	118
39	monDom5ChrX L3 Mars 75624825-75624942 result	21.19	5.08	-16.10	118
40	monDom5ChrX L3 10749574-10749694 result	4.13	0.00	-4.13	121
41	monDom5ChrX L3 Mars 28595389-28595511 result	5.69	20.33	14.63	123
42	monDom5ChrX L3 Mars 30652132-30652256 result	8.80	16.00	7.20	125
43	monDom5ChrX L3 Mars 34858661-34858794 result	0.00	10.45	10.45	134
44	monDom5ChrX L3 Mars 59560144-59560282 result	14.39	20.86	6.47	139
45	monDom5ChrX L3 Mars 20523821-20523960 result	0.00	25.71	25.71	140
46	monDom5ChrX L3 Mars 63120005-63120146 result	9.86	8.45	-1.41	142
47	monDom5ChrX Plat L3 12973570-12973723 result	3.90	22.73	18.83	154
48	monDom5ChrX L3 43226675-43226830 result	4.49	15.38	10.90	156
49	monDom5ChrX L3 Mars 57663660-57663817 result	13.92	24.68	10.76	158
50	monDom5ChrX L3 66856556-66856715 result	8.13	4.38	-3.75	160
51	monDom5ChrX L3 Mars 40067268-40067427 result	11.88	8.13	-3.75	160
52	monDom5ChrX L3 Mars 5961885-5962045 result	3.11	14.91	11.80	161
53	monDom5ChrX L3 Mars 68550491-68550665 result	6.29	15.43	9.14	175
54	monDom5ChrX Plat L3 69029118-69029295 result	2.81	15.73	12.92	178
55	monDom5ChrX L3 Mars 26786399-26786578 result	11.11	13.33	2.22	180
56	monDom5ChrX L3 Mars 71148302-71148490 result	0.00	5.82	5.82	189
57	monDom5ChrX L3 68436345-68436534 result	10.00	12.63	2.63	190
58	monDom5ChrX L3 Mars 51481587-51481779 result	0.00	23.32	23.32	193
59	monDom5ChrX L3 70148424-70148622 result	2.51	4.02	1.51	199
60	monDom5ChrX L3 52081555-52081754 result	8.50	12.50	4.00	200
61	monDom5ChrX Plat L3 46753654-46753854 result	0.00	15.92	15.92	201
62	monDom5ChrX L3 20410117-20410319 result	3.45	12.32	8.87	203
63	monDom5ChrX L3 Mars 19400954-19401156 result	11.33	13.30	1.97	203
64	monDom5ChrX L3 Mars 19566381-19566588 result	0.00	22.60	22.60	208
65	monDom5ChrX Plat L3 44151803-44152026 result	0.00	6.25	6.25	224
66	monDom5ChrX L3 Mars 13313507-13313731 result	6.22	7.56	1.33	225
67	monDom5ChrX L3 Mars 45404039-45404263 result	11.11	11.56	0.44	225
68	monDom5ChrX L3 Mars 69438507-69438732 result	3.10	26.55	23.45	226
69	monDom5ChrX L3 Mars 16862037-16862264 result	14.91	7.89	-7.02	228
70	monDom5ChrX Plat L3 26892968-26893197 result	0.00	23.91	23.91	230
71	monDom5ChrX L3 Mars 31092987-31093222 result	7.20	2.12	-5.08	236
72	monDom5ChrX L3 Mars 55132071-55132307 result	10.13	8.02	-2.11	237
73	monDom5ChrX L3 Mars 54495116-54495360 result	0.00	24.08	24.08	245
74	monDom5ChrX L3 Mars 35830211-35830457 result	8.10	4.45	-3.64	247
75	monDom5ChrX L3 41791303-41791553 result	4.38	4.78	0.40	251
76	monDom5ChrX L3 Mars 34918125-34918381 result	9.73	18.68	8.95	257
77	monDom5ChrX L3 Mars 19153405-19153662 result	8.53	20.16	11.63	258
78	monDom5ChrX L3 10812990-10813249 result	19.23	16.15	-3.08	260
79	monDom5ChrX Plat L3 33762939-33763205 result	0.00	4.87	4.87	267
80	monDom5ChrX Plat L3 77861631-77861898 result	3.36	23.51	20.15	268
81	monDom5ChrX L3 5979310-5979579 result	9.63	6.67	-2.96	270
82	monDom5ChrX Plat L3 8146564-8146833 result	7.04	11.48	4.44	270
83	monDom5ChrX Plat L3 41340775-41341048 result	8.03	10.95	2.92	274
84	monDom5ChrX L3 11505522-11505799 result	2.16	11.15	8.99	278
85	monDom5ChrX L3 60296727-60297006 result	5.36	15.00	9.64	280
86	monDom5ChrX L3 Mars 62542264-62542544 result	10.32	3.91	-6.41	281
87	monDom5ChrX L3 Mars 16658937-16659228 result	6.16	15.07	8.90	292
88	monDom5ChrX L3 Mars 10533603-10533895 result	2.39	6.83	4.44	293
89	monDom5ChrX L3 Mars 6103721-6104015 result	9.49	15.93	6.44	295
90	monDom5ChrX L3 Mars 46776805-46777107 result	7.26	11.88	4.62	303
91	monDom5ChrX L3 Mars 68682848-68683150 result	7.92	9.24	1.32	303
92	monDom5ChrX L3 Mars 55878577-55878883 result	0.00	15.64	15.64	307
93	monDom5ChrX L3 46603960-46604267 result	9.09	21.10	12.01	308
94	monDom5ChrX L3 Mars 61212985-61213293 result	3.88	6.47	2.59	309
95	monDom5ChrX L3 Mars 52016663-52016972 result	8.71	16.77	8.06	310
96	monDom5ChrX Plat L3 15942767-15943076 result	11.29	13.87	2.58	310
97	monDom5ChrX L3 Mars 29845441-29845752 result	3.53	22.44	18.91	312
98	monDom5ChrX L3 13538976-13539288 result	10.54	21.41	10.86	313
99	monDom5ChrX L3 Mars 51630954-51631279 result	7.98	10.74	2.76	326
100	monDom5ChrX Plat L3 28481033-28481359 result	1.83	6.42	4.59	327
101	monDom5ChrX Plat L3 8330664-8330991 result	3.66	11.28	7.62	328
102	monDom5ChrX Plat L3 62289443-62289771 result	3.65	25.84	22.19	329
103	monDom5ChrX L3 Mars 70495665-70495994 result	4.85	4.55	-0.30	330
104	monDom5ChrX Plat L3 21220765-21221098 result	3.59	23.05	19.46	334
105	monDom5ChrX L3 Mars 69353612-69353946 result	12.24	21.79	9.55	335
106	monDom5ChrX L3 Mars 34917186-34917523 result	5.92	10.65	4.73	338
107	monDom5ChrX L3 Mars 29278245-29278586 result	8.77	15.79	7.02	342
108	monDom5ChrX L3 Mars 51702334-51702676 result	5.83	13.12	7.29	343
109	monDom5ChrX L3 Mars 39697727-39698075 result	0.00	15.47	15.47	349
110	monDom5ChrX L3b 44270556-44270910 result	4.79	12.96	8.17	355
111	monDom5ChrX L3 Mars 48676135-48676492 result	7.82	13.97	6.15	358
112	monDom5ChrX L3 Mars 55741907-55742264 result	3.35	19.55	16.20	358
113	monDom5ChrX Plat L3 30431346-30431705 result	6.39	9.72	3.33	360
114	monDom5ChrX L3 Mars 54314961-54315328 result	4.35	18.48	14.13	368
115	monDom5ChrX Plat L3 32647602-32647970 result	9.76	17.89	8.13	369
116	monDom5ChrX L3 41347481-41347852 result	9.14	14.78	5.65	372
117	monDom5ChrX L3 Mars 56138026-56138402 result	7.69	15.92	8.22	377
118	monDom5ChrX L3 57818651-57819031 result	14.44	19.16	4.72	381
119	monDom5ChrX L3 49582829-49583217 result	5.40	17.74	12.34	389
120	monDom5ChrX L3 75204063-75204464 result	12.19	17.91	5.72	402
121	monDom5ChrX L3 Mars 34278573-34278975 result	10.17	6.20	-3.97	403
122	monDom5ChrX L3 Mars 4793379-4793783 result	4.94	17.53	12.59	405
123	monDom5ChrX L3 Mars 53615465-53615869 result	4.20	16.79	12.59	405
124	monDom5ChrX L3 Mars 70721834-70722242 result	4.65	6.60	1.96	409
125	monDom5ChrX L3 Mars 42956453-42956866 result	4.83	16.91	12.08	414
126	monDom5ChrX L3 Mars 34649331-34649746 result	5.77	13.70	7.93	416
127	monDom5ChrX Plat L3 51268348-51268764 result	1.44	17.03	15.59	417
128	monDom5ChrX L3 Mars 30270243-30270661 result	7.88	23.15	15.27	419
129	monDom5ChrX L3 Mars 45759415-45759835 result	1.19	22.80	21.62	421
130	monDom5ChrX L3 Mars 8627844-8628266 result	3.78	22.93	19.15	423
131	monDom5ChrX L3 Mars 78440276-78440702 result	7.73	13.58	5.85	427
132	monDom5ChrX Plat L3 14506792-14507218 result	3.51	7.49	3.98	427
133	monDom5ChrX Plat L3 4883092-34883521 result	3.26	13.49	10.23	430
134	monDom5ChrX L3 Mars 40016611-40017046 result	5.05	22.71	17.66	436
135	monDom5ChrX L3 Mars 18033473-18033912 result	8.41	15.68	7.27	440
136	monDom5ChrX L3 Mars 19820889-19821328 result	0.00	13.41	13.41	440
137	monDom5ChrX L3 Mars 49130767-49131212 result	0.00	18.61	18.61	446
138	monDom5ChrX Plat L3 41260420-41260868 result	0.00	9.80	9.80	449
139	monDom5ChrX L3 Mars 53795940-53796390 result	5.76	14.41	8.65	451
140	monDom5ChrX L3 40120974-40121438 result	1.29	19.78	18.49	465
141	monDom5ChrX L3 31867279-31867745 result	6.00	17.34	11.35	467
142	monDom5ChrX L3 Mars 62756466-62756934 result	6.82	18.34	11.51	469
143	monDom5ChrX L3 Mars 69204185-69204656 result	1.48	16.31	14.83	472
144	monDom5ChrX L3 Mars 65220313-65220790 result	5.86	11.30	5.44	478
145	monDom5ChrX L3 Mars 11668486-11668964 result	3.13	9.81	6.68	479
146	monDom5ChrX L3 Mars 69006467-69006945 result	3.97	20.04	16.08	479
147	monDom5ChrX L3 Mars 21318700-21319180 result	3.95	16.22	12.27	481
148	monDom5ChrX L3 7733458-7733955 result	0.00	9.24	9.24	498
149	monDom5ChrX L3 Mars 77075203-77075701 result	4.81	7.82	3.01	499
150	monDom5ChrX L3 Mars 48960206-48960706 result	3.79	10.38	6.59	501
151	monDom5ChrX L3 Mars 58126602-58127105 result	2.98	18.65	15.67	504
152	monDom5ChrX L3 Mars 35086580-35087086 result	5.33	17.36	12.03	507
153	monDom5ChrX L3 Mars 40453168-40453679 result	2.93	18.55	15.63	512
154	monDom5ChrX L3 Mars 18441175-18441689 result	5.63	13.98	8.35	515
155	monDom5ChrX L3 Mars 18153635-18154150 result	7.17	9.69	2.52	516
156	monDom5ChrX L3 Mars 34183186-34183718 result	5.25	9.76	4.50	533
157	monDom5ChrX L3 Mars 64031094-64031628 result	8.60	10.65	2.06	535
158	monDom5ChrX L3 Mars 59572799-59573335 result	7.64	17.32	9.68	537
159	monDom5ChrX L3 Mars 16151082-16151619 result	3.72	12.64	8.92	538
160	monDom5ChrX L3 Mars 39357871-39358409 result	1.11	15.77	14.66	539
161	monDom5ChrX L3 Mars 20574120-20574661 result	2.40	18.63	16.24	542
162	monDom5ChrX L3 Mars 29319907-29320450 result	8.09	14.71	6.62	544
163	monDom5ChrX L3 Mars 74596343-74596888 result	4.03	15.93	11.90	546
164	monDom5ChrX L3 Mars 31694495-31695041 result	4.39	23.22	18.83	547
165	monDom5ChrX L3 Mars 31386794-31387343 result	10.55	19.27	8.73	550
166	monDom5ChrX L3 Mars 61473311-61473862 result	3.62	15.94	12.32	552
167	monDom5ChrX Plat L3 13745460-13746011 result	1.27	12.50	11.23	552
168	monDom5ChrX L3 Mars 49637075-49637630 result	5.58	25.36	19.78	556
169	monDom5ChrX L3 Mars 60156315-60156876 result	6.05	18.15	12.10	562
170	monDom5ChrX L3 Mars 15407472-15408036 result	0.00	14.16	14.16	565
171	monDom5ChrX L3 Mars 52897681-52898245 result	2.12	17.35	15.22	565
172	monDom5ChrX L3 Mars 49929957-49930524 result	5.63	16.20	10.56	568
173	monDom5ChrX L3 Mars 5941396-5941965 result	5.79	22.98	17.19	570
174	monDom5ChrX L3 Mars 4570453-4571023 result	6.30	18.04	11.73	571
175	monDom5ChrX Plat L3 33170988-33171575 result	1.02	8.16	7.14	588
176	monDom5ChrX Plat L3 56600233-56600820 result	2.21	14.63	12.41	588
177	monDom5ChrX L3 Mars 36373108-36373702 result	0.84	17.31	16.47	595
178	monDom5ChrX L3 Mars 19121262-19121858 result	2.85	18.26	15.41	597
179	monDom5ChrX L3 Mars 54635392-54635988 result	3.69	23.62	19.93	597
180	monDom5ChrX L3 Mars 18582057-18582659 result	4.31	20.23	15.92	603
181	monDom5ChrX L3 Mars 61731697-61732299 result	4.48	11.28	6.80	603
182	monDom5ChrX L3 Mars 31696570-31697173 result	4.47	18.87	14.40	604
183	monDom5ChrX L3 Mars 10797547-10798151 result	0.99	14.55	13.55	605
184	monDom5ChrX L3 Mars 17676953-17677558 result	3.80	10.89	7.10	606
185	monDom5ChrX L3 18257783-18258392 result	3.93	14.59	10.66	610
186	monDom5ChrX L3 Mars 77855838-77856453 result	3.73	16.23	12.50	616
187	monDom5ChrX L3 Mars 30825935-30826551 result	5.19	20.75	15.56	617
188	monDom5ChrX L3 Mars 20173495-20174113 result	6.14	15.51	9.37	619
189	monDom5ChrX L3 Mars 38412576-38413203 result	6.53	10.51	3.98	628
190	monDom5ChrX L3 Mars 33963276-33963905 result	7.14	21.90	14.76	630
191	monDom5ChrX L3 Mars 41425289-41425919 result	5.39	13.79	8.40	631
192	monDom5ChrX L3 Mars 7739689-7740322 result	0.79	24.13	23.34	634
193	monDom5ChrX L3 Mars 15328476-15329111 result	3.14	20.28	17.14	636
194	monDom5ChrX L3 Mars 28015704-28016340 result	5.97	16.33	10.36	637
195	monDom5ChrX Plat L3 52692820-52693456 result	5.18	17.90	12.72	637
196	monDom5ChrX L3 Mars 69449662-69450304 result	3.42	15.71	12.29	643
197	monDom5ChrX L3 Mars 34884735-34885380 result	3.25	16.87	13.62	646
198	monDom5ChrX L3 Mars 33450290-33450937 result	2.93	18.36	15.43	648
199	monDom5ChrX L3 Mars 46368369-46369016 result	5.40	10.03	4.63	648
200	monDom5ChrX Plat L3 44301926-44302577 result	1.99	17.33	15.34	652
201	monDom5ChrX L3 Mars 54594670-54595322 result	7.20	17.15	9.95	653
202	monDom5ChrX L3 Mars 9558425-9559079 result	6.11	12.52	6.41	655
203	monDom5ChrX Plat L3 33289460-33290115 result	4.88	10.52	5.64	656
204	monDom5ChrX L3 Mars 0098966-60099627 result	2.11	14.50	12.39	662
205	monDom5ChrX L3 Mars 18463837-18464499 result	13.57	18.10	4.52	663
206	monDom5ChrX L3 Mars 18317481-18318145 result	7.37	11.88	4.51	665
207	monDom5ChrX L3 Mars 62521631-62522303 result	3.86	21.25	17.38	673
208	monDom5ChrX L3 Mars 57720379-57721064 result	3.50	14.58	11.08	686
209	monDom5ChrX L3 Mars 62888932-62889621 result	4.64	10.29	5.65	690
210	monDom5ChrX L3 Mars 41876509-41877208 result	4.57	22.00	17.43	700
211	monDom5ChrX L3 Mars 44946409-44947108 result	5.29	15.00	9.71	700

10	monDom5ChrX RTE1 Mars 4574996-4575	0.00	11.76	11.76	68
11	monDom5ChrX RTE1 Mars 76362018-763	0.00	21.43	21.43	70
12	monDom5ChrX RTE1 Mars 61674879-616	0.00	10.96	10.96	73
13	monDom5ChrX RTE1 Mars 21089674-210	16.88	9.09	-7.79	77
14	monDom5ChrX RTE1 Mars 73870890-738	0.00	7.69	7.69	78
15	monDom5ChrX RTE1 Mars 43135928-431	0.00	13.75	13.75	80
16	monDom5ChrX RTE1 Mars 16845372-168	0.00	29.63	29.63	81
17	monDom5ChrX RTE1 Mars 51140394-511	14.29	13.10	-1.19	84
18	monDom5ChrX RTE1 Mars 49922245-499	0.00	23.26	23.26	86
19	monDom5ChrX RTE1 Mars 63498427-634	0.00	10.34	10.34	87
20	monDom5ChrX RTE1 Mars 37447404-374	6.82	11.36	4.55	88
21	monDom5ChrX RTE1 Mars 44954785-449	7.78	17.78	10.00	90
22	monDom5ChrX RTE1 Mars 16308696-163	8.70	7.61	-1.09	92
23	monDom5ChrX RTE1 Mars 58521147-585	0.00	12.90	12.90	93
24	monDom5ChrX RTE1 Mars 68674716-686	12.90	7.53	-5.38	93
25	monDom5ChrX RTE1 Mars 44562069-445	0.00	14.74	14.74	95
26	monDom5ChrX RTE Mdo 29370747-29370	9.28	7.22	-2.06	97
27	monDom5ChrX RTE1 Mars 35947187-359	10.00	7.00	-3.00	100
28	monDom5ChrX RTE1 Mars 34003191-340	5.94	14.85	8.91	101
29	monDom5ChrX RTE1 Mars 59581439-595	4.95	12.87	7.92	101
30	monDom5ChrX RTE1 Mars 15023483-150	6.54	6.54	0.00	107
31	monDom5ChrX RTE1 Mars 19816707-198	5.50	11.93	6.42	109
32	monDom5ChrX RTE1 Mars 51181609-511	10.00	13.64	3.64	110
33	monDom5ChrX RTE1 Mars 54529036-545	0.00	14.29	14.29	112
34	monDom5ChrX RTE1 Mars 78110736-781	12.39	5.31	-7.08	113
35	monDom5ChrX RTE1 Mars 51744820-517	0.00	16.10	16.10	118
36	monDom5ChrX RTE1 Mars 10579674-105	4.96	14.88	9.92	121
37	monDom5ChrX RTE1 Mars 45368238-453	8.80	0.00	-8.80	125
38	monDom5ChrX RTE1 Mars 20778971-207	0.00	25.58	25.58	129
39	monDom5ChrX RTE1 Mars 48984973-489	0.00	19.85	19.85	131
40	monDom5ChrX RTE1 Mars 31213392-312	5.63	14.08	8.45	142
41	monDom5ChrX RTE1 Mars 75560645-755	0.00	33.57	33.57	143
42	monDom5ChrX RTE1 Mars 78397667-783	3.50	0.00	-3.50	143
43	monDom5ChrX RTE1 Mars 45876815-458	8.05	8.05	0.00	149
44	monDom5ChrX RTE1 Mars 18322375-183	5.56	3.09	-2.47	162
45	monDom5ChrX RTE1 Mars 46323911-463	0.00	18.07	18.07	166
46	monDom5ChrX RTE1 Mars 67295049-672	7.23	19.88	12.65	166
47	monDom5ChrX RTE1 Mars 59290922-592	4.12	10.00	5.88	170
48	monDom5ChrX RTE1 Mars 39460967-394	3.49	19.19	15.70	172
49	monDom5ChrX RTE1 Mars 39483750-394	17.24	9.77	-7.47	174
50	monDom5ChrX RTE1 Mars 49861182-498	3.95	19.21	15.25	177
51	monDom5ChrX RTE1 Mars 45230832-452	3.24	5.41	2.16	185
52	monDom5ChrX RTE1 Mars 30108750-301	3.19	14.36	11.17	188
53	monDom5ChrX RTE1 Mars 34556944-345	4.26	11.17	6.91	188
54	monDom5ChrX RTE1 Mars 34134191-341	0.00	23.20	23.20	194
55	monDom5ChrX RTE1 Mars 13593403-135	4.62	15.90	11.28	195
56	monDom5ChrX RTE1 Mars 58664930-586	0.00	16.67	16.67	198
57	monDom5ChrX RTE1 Mars 65753429-657	14.00	17.50	3.50	200
58	monDom5ChrX RTE1 Mars 8778050-8778	2.45	11.76	9.31	204
59	monDom5ChrX RTE1 Mars 69456523-694	2.42	8.70	6.28	207
60	monDom5ChrX RTE1 Mars 57690248-576	0.00	24.53	24.53	212
61	monDom5ChrX RTE1 Mars 46284072-462	0.00	23.72	23.72	215
62	monDom5ChrX RTE1 Mars 49966211-499	11.87	10.50	-1.37	219
63	monDom5ChrX RTE1 Mars 18740673-187	7.27	8.18	0.91	220
64	monDom5ChrX RTE1 Mars 5907035-5907	6.33	8.14	1.81	221
65	monDom5ChrX RTE1 Mars 75609580-756	5.86	16.22	10.36	222
66	monDom5ChrX RTE1 Mars 34387723-343	6.17	14.10	7.93	227
67	monDom5ChrX RTE1 Mars 57084313-570	4.80	12.23	7.42	229
68	monDom5ChrX RTE1 Mars 53800917-538	4.78	5.22	0.43	230
69	monDom5ChrX RTE1 Mars 36245141-362	11.20	8.30	-2.90	241
70	monDom5ChrX RTE1 Mars 41590348-415	0.00	7.02	7.02	242
71	monDom5ChrX RTE1 Mars 44027000-440	3.70	9.88	6.17	243
72	monDom5ChrX RTE1 Mars 47135411-471	7.32	17.48	10.16	246
73	monDom5ChrX RTE1 Mars 5275371-5275	0.00	21.60	21.60	250
74	monDom5ChrX RTE1 Mars 59835538-598	0.00	11.07	11.07	253
75	monDom5ChrX RTE1 Mars 74984455-749	9.85	14.77	4.92	264
76	monDom5ChrX RTE1 Mars 34680146-346	5.88	13.60	7.72	272
77	monDom5ChrX RTE1 Mars 37377273-373	6.52	18.12	11.59	276
78	monDom5ChrX RTE1 Mars 62050558-620	2.51	22.94	20.43	279
79	monDom5ChrX RTE1 Mars 67361465-673	10.04	13.62	3.58	279
80	monDom5ChrX RTE-2 MD 6101358-61016	6.07	11.07	5.00	280
81	monDom5ChrX RTE1 Mars 39363215-393	6.29	22.38	16.08	286
82	monDom5ChrX RTE1 Mars 58020189-580	9.66	11.03	1.38	290
83	monDom5ChrX RTE1 Mars 29323426-293	0.00	15.07	15.07	292
84	monDom5ChrX RTE1 Mars 58433351-584	1.71	16.44	14.73	292
85	monDom5ChrX RTE1 Mars 68563664-685	1.71	21.16	19.45	293
86	monDom5ChrX RTE1 Mars 49809192-498	2.71	6.78	4.07	295
87	monDom5ChrX RTE1 Mars 65587289-655	6.31	16.94	10.63	301
88	monDom5ChrX RTE-2 MD 37695054-3769	4.55	8.44	3.90	308
89	monDom5ChrX RTE1 Mars 61072670-610	7.44	21.04	13.59	309
90	monDom5ChrX RTE1 Mars 35586090-355	1.89	24.92	23.03	317
91	monDom5ChrX RTE1 Mars 12273740-122	1.57	19.18	17.61	318
92	monDom5ChrX RTE1 Mars 48208732-482	7.41	10.80	3.40	324
93	monDom5ChrX RTE1 Mars 29986167-299	1.53	8.87	7.34	327
94	monDom5ChrX RTE1 Mars 48305507-483	3.03	18.48	15.45	330
95	monDom5ChrX RTE1 Mars 32367877-323	3.88	14.03	10.15	335
96	monDom5ChrX RTE1 Mars 31378505-313	6.85	11.90	5.06	336
97	monDom5ChrX RTE1 Mars 34750865-347	7.69	11.54	3.85	338
98	monDom5ChrX RTE1 Mars 71166873-711	9.38	17.30	7.92	341
99	monDom5ChrX RTE1 Mars 69149482-691	1.75	16.33	14.58	343
100	monDom5ChrX RTE1 Mars 28686926-286	6.40	20.06	13.66	344
101	monDom5ChrX RTE1 Mars 66436258-664	0.00	21.16	21.16	345
102	monDom5ChrX RTE1 Mars 33406701-334	7.34	15.25	7.91	354
103	monDom5ChrX RTE1 Mars 28381848-283	3.91	13.41	9.50	358
104	monDom5ChrX RTE1 Mars 16818770-168	7.18	17.13	9.94	362
105	monDom5ChrX RTE1 Mars 34847446-348	1.37	18.31	16.94	366
106	monDom5ChrX RTE1 Mars 54170767-541	3.23	6.74	3.50	371
107	monDom5ChrX RTE1 Mars 10528141-105	0.00	16.31	16.31	374
108	monDom5ChrX RTE1 Mars 34347201-343	4.50	8.20	3.70	378
109	monDom5ChrX RTE1 Mars 65388784-653	4.50	15.34	10.85	378
110	monDom5ChrX RTE1 Mars 52314012-523	0.00	16.09	16.09	379
111	monDom5ChrX RTE1 Mars 18972935-189	7.83	17.75	9.92	383
112	monDom5ChrX RTE1 Mars 27302447-273	5.47	8.59	3.13	384
113	monDom5ChrX RTE1 Mars 47153582-471	1.27	13.42	12.15	395
114	monDom5ChrX RTE1 Mars 17389300-173	4.55	5.30	0.76	396
115	monDom5ChrX RTE1 Mars 49967010-499	2.01	16.08	14.07	398
116	monDom5ChrX RTE1 Mars 76026246-760	5.51	19.05	13.53	399
117	monDom5ChrX RTE1 Mars 34388071-343	2.98	16.63	13.65	403
118	monDom5ChrX RTE1 Mars 53500002-535	5.12	12.20	7.07	410
119	monDom5ChrX RTE1 Mars 68664955-686	2.93	7.56	4.63	410
120	monDom5ChrX RTE1 Mars 51519105-515	5.60	8.76	3.16	411
121	monDom5ChrX RTE1 Mars 8528822-8529	7.73	11.35	3.62	414
122	monDom5ChrX RTE1 Mars 65531627-655	3.60	23.50	19.90	417
123	monDom5ChrX RTE1 Mars 59281264-592	8.61	6.70	-1.91	418
124	monDom5ChrX RTE1 Mars 63984866-639	5.01	18.14	13.13	419
125	monDom5ChrX RTE Mdo 32718526-32718	1.19	6.44	5.25	419
126	monDom5ChrX RTE1 Mars 62403434-624	1.43	15.71	14.29	420
127	monDom5ChrX RTE1 Mars 18124869-181	10.75	3.97	-6.78	428
128	monDom5ChrX RTE1 Mars 33998563-339	3.27	17.29	14.02	428
129	monDom5ChrX RTE1 Mars 53890969-538	5.59	14.92	9.32	429
130	monDom5ChrX RTE1 Mars 40172132-401	4.19	20.00	15.81	430
131	monDom5ChrX RTE1 Mars 78333100-783	2.99	11.95	8.97	435
132	monDom5ChrX RTE Mdo 21793815-21794	1.15	8.28	7.13	435
133	monDom5ChrX RTE1 Mars 77516349-775	2.75	12.81	10.07	437
134	monDom5ChrX RTE1 Mars 54912227-549	1.59	10.71	9.11	439
135	monDom5ChrX RTE1 Mars 6412853-6413	5.47	17.31	11.85	439
136	monDom5ChrX RTE1 Mars 54826741-548	4.07	16.52	12.44	442
137	monDom5ChrX RTE1 Mars 33406108-334	0.00	14.80	14.80	446
138	monDom5ChrX RTE1 Mars 74614585-746	7.08	6.86	-0.22	452
139	monDom5ChrX RTE1 Mars 38286096-382	4.98	18.61	13.64	462
140	monDom5ChrX RTE1 Mars 39536842-395	4.09	16.34	12.26	465
141	monDom5ChrX RTE-2 MD 3142218-31426	5.52	12.53	7.01	471
142	monDom5ChrX RTE1 Mars 74269075-742	4.02	21.14	17.12	473
143	monDom5ChrX RTE1 Mars 17549264-175	0.00	13.42	13.42	477
144	monDom5ChrX RTE1 Mars 63299855-633	9.22	16.80	7.58	488
145	monDom5ChrX RTE1 Mars 51704293-517	8.38	16.16	7.77	489
146	monDom5ChrX RTE1 Mars 38513487-385	1.61	20.97	19.35	496
147	monDom5ChrX RTE-2 MD 41046552-4104	4.79	13.17	8.38	501
148	monDom5ChrX RTE-2 MD 33643128-3364	3.78	10.36	6.57	502
149	monDom5ChrX RTE1 Mars 6372898-6373	12.95	8.76	-4.18	502
150	monDom5ChrX RTE1 Mars 56440977-564	7.91	12.25	4.35	506
151	monDom5ChrX RTE1 Mars 79332409-793	1.97	19.09	17.13	508
152	monDom5ChrX RTE1 Mars 42906917-429	4.13	10.41	6.29	509
153	monDom5ChrX RTE1 Mars 16917553-169	5.07	13.65	8.58	513
154	monDom5ChrX RTE1 Mars 35270212-352	8.38	15.01	6.63	513
155	monDom5ChrX RTE1 Mars 59419946-594	1.36	6.80	5.44	515
156	monDom5ChrX RTE1 Mars 62802854-628	4.64	16.25	11.61	517
157	monDom5ChrX RTE1 Mars 52021079-520	4.05	14.09	10.04	518
158	monDom5ChrX RTE1 Mars 66354145-663	8.40	14.31	5.92	524
159	monDom5ChrX RTE1 Mars 8774879-8775	7.20	13.64	6.44	528
160	monDom5ChrX RTE1 Mars 73410774-734	3.21	14.93	11.72	529
161	monDom5ChrX RTE1 Mars 33183088-331	2.62	19.66	17.04	534
162	monDom5CrX RTE1 Mars 66355782-6635	4.85	10.07	5.22	536
163	monDom5ChrX RTE1 Mars 48935390-489	5.15	24.82	19.67	544
164	monDom5ChrX RTE1 Mars 76750607-767	6.58	10.05	3.47	547
165	monDom5ChrX RTE1 Mars 44468920-444	3.82	15.82	12.00	550
166	monDom5ChrX RTE1 Mars 52715521-527	3.27	18.00	14.73	550
167	monDom5ChrX RTE1 Mars 76724492-767	3.64	17.45	13.82	550
168	monDom5ChrX RTE1 Mars 16304834-163	3.23	9.14	5.91	558
169	monDom5ChrX RTE1 Mars 45043325-450	8.87	13.48	4.61	564
170	monDom5ChrX RTE1 Mars 70354587-703	2.65	17.52	14.87	565
171	monDom5ChrX RTE1 Mars 34896382-348	3.68	11.40	7.72	570
172	monDom5ChrX RTE1 Mars 63883326-638	5.41	25.31	19.90	573
173	monDom5ChrX RTE1 Mars 43931157-439	6.27	15.08	8.81	590
174	monDom5ChrX RTE1 Mars 63069093-630	8.63	12.18	3.55	591
175	monDom5ChrX RTE1 Mars 5628462-5629	2.20	22.47	20.27	592
176	monDom5ChrX RTE1 Mars 65040339-650	5.57	9.80	4.22	592
177	monDom5ChrX RTE1 Mars 76529088-765	0.81	18.51	17.69	616
178	monDom5ChrX RTE1 Mars 19741164-197	2.09	16.53	14.45	623
179	monDom5ChrX RTE1 Mars 55681593-556	5.13	12.34	7.21	624
180	monDom5ChrX RTE1 Mars 18277155-182	6.70	8.29	1.59	627
181	monDom5ChrX RTE1 Mars 29030278-290	1.59	13.56	11.96	627
182	monDom5ChrX RTE1 Mars 33870556-338	5.57	10.99	5.41	628
183	monDom5ChrX RTE1 Mars 61854133-618	6.04	13.67	7.63	629
184	monDom5ChrX RTE1 Mars 65462442-654	8.11	10.33	2.23	629
185	monDom5ChrX RTE1 Mars 18301768-183	5.85	11.53	5.69	633
186	monDom5ChrX RTE1 Mars 30240546-302	5.91	15.24	9.33	643
187	monDom5hChrX RTE Mdo 8508212-85088	1.24	3.55	2.32	647
188	monDom5ChrX RTE-2 MD 57609654-5761	6.94	8.02	1.08	648
189	monDom5ChrX RTE1 Mars 52412568-524	4.30	23.81	19.51	651
190	monDom5ChrX RTE1 Mars 55983531-559	2.45	16.08	13.63	653
191	monDom5ChrX RTE1 Mars 8678682-8679	6.13	12.71	6.58	653
192	monDom5ChrX RTE1 Mars 39458798-394	1.67	13.66	11.99	659
193	monDom5ChrX RTE1 Mars 50294951-502	4.86	11.99	7.13	659
194	monDom5ChrX RTE1 Mars 54761362-547	6.18	8.75	2.56	663
195	monDom5ChrX RTE1 Mars 13599050-135	4.97	8.89	3.92	664
196	monDom5ChrX RTE1 Mars 17586343-175	6.76	11.86	5.11	666
197	monDom5ChrX RTE Mdo 15663877-15664	1.95	5.99	4.04	668
198	monDom5ChrX RTE1 Mars 13151105-131	8.06	15.67	7.61	670
199	monDom5ChrX RTE1 Mars 21038017-210	5.35	12.93	7.58	673
200	monDom5ChrX RTE1 Mars 33948752-339	6.63	13.11	6.48	679
201	monDom5ChrX RTE1 Mars 66102613-661	3.53	12.67	9.13	679
202	monDom5ChrX RTE1 Mars 56788626-567	7.71	11.06	3.35	687
203	monDom5ChrX RTE1 Mars 29662449-296	4.49	11.29	6.80	691
204	monDom5ChrX RTE1 Mars 62143424-621	4.19	17.77	13.58	692
205	monDom5ChrX RTE1 Mars 48006732-480	5.04	14.96	9.93	695
206	monDom5ChrX RTE1 Mars 7957619-7958	1.44	14.22	12.79	696
207	monDom5ChrX RTE1 Mars 65224729-652	4.81	11.60	6.79	707
208	monDom5ChrX RTE1 Mars 39202621-392	5.90	20.22	14.33	712
209	monDom5ChrX RTE1 Mars 74412000-744	6.02	15.83	9.80	714
210	monDom5ChrX RTE1 Mars 67943777-679	1.68	17.76	16.08	715
211	monDom5ChrX RTE1 Mars 10065267-100	7.66	5.85	-1.81	718

212	monDom5ChrX L3 Mars 62168869-62169570 result	3.42	15.95	12.54	702
213	monDom5ChrX L3 Mars 6614585-6615287 result	2.70	28.02	25.32	703
214	monDom5ChrX Plat L3 58599763-58600467 result	1.84	15.74	13.90	705
215	monDom5ChrX Plat L3 64228160-64228866 result	5.37	8.06	2.69	707
216	monDom5ChrX L3 41781742-41782449 result	6.21	12.15	5.93	708
217	monDom5ChrX Plat L3 58667937-58668648 result	0.70	18.40	17.70	712
218	monDom5ChrX L3 Mars 19444253-19444967 result	7.27	13.99	6.71	715
219	monDom5ChrX L3 Mars 50318406-50319120 result	4.76	22.80	18.04	715
220	monDom5ChrX L3 Mars 12378864-12379580 result	5.30	14.23	8.93	717
221	monDom5ChrX L3 Mars 10770438-10771156 result	5.56	22.95	17.39	719
222	monDom5ChrX L3 Mars 11157111-11157830 result	1.39	18.19	16.81	720
223	monDom5ChrX L3 Mars 30091568-30092287 result	8.06	13.06	5.00	720
224	monDom5ChrX Plat L3 16869662-16870400 result	0.00	15.83	15.83	739
225	monDom5ChrX L3 Mars 19803427-19804169 result	5.65	10.90	5.25	743
226	monDom5ChrX L3 Mars 38055013-38055755 result	3.50	14.00	10.50	743
227	monDom5ChrX Plat L3 16735804-16736550 result	2.95	8.43	5.49	747
228	monDom5ChrX Plat L3 29432641-29433387 result	1.87	12.45	10.58	747
229	monDom5ChrX L3 Mars 52874452-52875217 result	4.83	25.20	20.37	766
230	monDom5ChrX Plat L3 56560445-56561216 result	6.61	9.72	3.11	772
231	monDom5ChrX L3 Mars 78992990-78993762 result	1.94	13.07	11.13	773
232	monDom5ChrX L3 Mars 6302067-6302846 result	9.23	13.72	4.49	780
233	monDom5ChrX L3 Mars 30991521-30992301 result	2.30	10.12	7.81	781
234	monDom5ChrX L3 73362207-73362992 result	2.67	21.76	19.08	786
235	monDom5ChrX L3 Mars 41747005-41747791 result	4.07	21.47	17.41	787
236	monDom5ChrX L3 Mars 72777160-72777950 result	5.82	6.95	1.14	791
237	monDom5ChrX Plat L3 46287071-46287861 result	3.79	13.91	10.11	791
238	monDom5ChrX L3 Mars 66619160-66619956 result	6.15	19.20	13.05	797
239	monDom5ChrX L3 Mars 31544828-31545625 result	2.13	17.67	15.54	798
240	monDom5ChrX L3 Mars 19635205-19636005 result	7.24	17.10	9.86	801
241	monDom5ChrX L3 Mars 58825507-58826307 result	2.25	23.97	21.72	801
242	monDom5ChrX L3 Mars 15595355-15596161 result	4.21	23.17	18.96	807
243	monDom5ChrX L3 Mars 5762453-5763260 result	3.84	10.77	6.93	808
244	monDom5ChrX L3 Mars 12060858-12061666 result	6.43	20.89	14.46	809
245	monDom5ChrX L3 Mars 7995630-7996442 result	4.55	15.13	10.58	813
246	monDom5ChrX L3 Mars 5710378-5711196 result	5.25	18.07	12.82	819
247	monDom5ChrX L3 Mars 10793141-10793961 result	5.85	23.39	17.54	821
248	monDom5ChrX L3 Mars 41256882-41257702 result	4.14	17.90	13.76	821
249	monDom5ChrX L3 Mars 44190655-44191480 result	3.51	19.85	16.34	826
250	monDom5ChrX Plat L3 77526398-77527224 result	2.78	14.87	12.09	827
251	monDom5ChrX L3 Mars 6034016-6034843 result	5.19	18.12	12.92	828
252	monDom5ChrX L3 Mars 15946126-15946963 result	4.06	18.50	14.44	838
253	monDom5ChrX L3 Mars 15027324-15028163 result	3.10	16.55	13.45	840
254	monDom5ChrX L3 Mars 65659991-65660835 result	5.09	24.73	19.64	845
255	monDom5ChrX L3 Mars 45179942-45180795 result	1.99	19.91	17.92	854
256	monDom5ChrX L3 Mars 67906016-67906869 result	3.75	21.78	18.03	854
257	monDom5ChrX L3 Mars 47096232-47097086 result	2.57	15.20	12.63	855
258	monDom5ChrX L3 Mars 5761417-5762274 result	3.38	21.56	18.18	858
259	monDom5ChrX Plat L3 65710259-65711121 result	4.52	12.51	8.00	863
260	monDom5ChrX L3 Mars 50457492-50458356 result	3.70	22.43	18.73	865
261	monDom5ChrX Plat L3 61023521-61024392 result	4.59	9.06	4.47	872
262	monDom5ChrX L3 Mars 75154770-75155642 result	5.38	17.07	11.68	873
263	monDom5ChrX L3 Mars 59257311-59258184 result	5.72	22.65	16.93	874
264	monDom5ChrX L3 Mars 40015049-40015924 result	4.91	13.70	8.79	876
265	monDom5ChrX L3 Mars 6032895-6033771 result	2.62	19.38	16.76	877
266	monDom5ChrX Plat L3 39920664-39921540 result	3.19	12.66	9.46	877
267	monDom5ChrX L3 Mars 6798919-6799801 result	4.42	11.44	7.02	883
268	monDom5ChrX L3 15316764-15317656 result	5.49	23.40	17.92	893
269	monDom5ChrX L3 Mars 68491810-68492703 result	4.25	14.09	9.84	894
270	monDom5ChrX L3 Mars 50475920-50476819 result	2.44	13.78	11.33	900
271	monDom5ChrX L3 Mars 44601542-44602442 result	3.88	22.31	18.42	901
272	monDom5ChrX L3 Mars 21246839-21247744 result	3.86	16.34	12.47	906
273	monDom5ChrX L3b 33614223-33615131 result	4.40	22.66	18.26	909
274	monDom5ChrX L3 Mars 18933841-18934752 result	4.28	20.29	16.01	912
275	monDom5ChrX L3 Mars 59695430-59696343 result	5.80	20.35	14.55	914
276	monDom5ChrX L3 Mars 7698044-7698961 result	2.29	23.64	21.35	918
277	monDom5ChrX L3 14548280-14549203 result	4.55	19.70	15.15	924
278	monDom5ChrX L3 Mars 19464754-19465683 result	3.87	14.41	10.54	930
279	monDom5ChrX L3 Mars 32681942-32682873 result	5.79	22.64	16.85	932
280	monDom5ChrX L3 Mars 52541222-52542154 result	3.64	25.62	21.97	933
281	monDom5ChrX L3 Mars 22242549-22243486 result	3.62	18.87	15.25	938
282	monDom5ChrX Plat L3 16252970-16253907 result	2.99	14.50	11.51	938
283	monDom5ChrX L3 Mars 61819948-61820888 result	3.83	12.96	9.14	941
284	monDom5ChrX L3 Mars 55560615-55561560 result	4.55	18.29	13.74	946
285	monDom5ChrX L3 Mars 7942508-7943455 result	3.06	26.27	23.21	948
286	monDom5ChrX L3 Mars 17158083-17159031 result	2.85	13.70	10.85	949
287	monDom5ChrX L3 Mars 58759588-58760536 result	3.79	23.29	19.49	949
288	monDom5ChrX L3 Mars 43378125-43379088 result	2.90	18.46	15.56	964
289	monDom5ChrX L3 Mars 38167052-38168016 result	6.42	16.99	10.57	965
290	monDom5ChrX L3b 45538217-45539182 result	3.42	10.66	7.25	966
291	monDom5ChrX L3 Mars 54717895-54718862 result	3.82	21.90	18.08	968
292	monDom5ChrX L3 Mars 55932329-55933297 result	4.75	23.32	18.58	969
293	monDom5ChrX L3 Mars 57207312-57208283 result	2.98	22.12	19.14	972
294	monDom5ChrX L3 Mars 18808927-18809904 result	2.56	12.99	10.43	978
295	monDom5ChrX L3 Mars 39807036-39808014 result	3.47	19.41	15.93	979
296	monDom5ChrX L3 Mars 65309664-65310648 result	3.35	10.56	7.21	985
297	monDom5ChrX L3 29675589-29676579 result	1.92	11.20	9.28	991
298	monDom5ChrX L3 Mars 55642507-55643498 result	2.42	14.21	11.79	992
299	monDom5ChrX L3 Mars 65946606-65947598 result	6.24	17.62	11.38	993
300	monDom5ChrX L3 Mars 62459262-62460262 result	4.60	17.88	13.29	1001
301	monDom5ChrX L3 Mars 53629978-53630981 result	2.99	15.04	12.05	1004
302	monDom5ChrX L3 Mars 9227213-9228219 result	4.47	21.85	17.38	1007
303	monDom5ChrX L3 Mars 27822659-27823686 result	3.21	22.76	19.55	1028
304	monDom5ChrX Plat L3 9415289-9416332 result	4.79	16.09	11.30	1044
305	monDom5ChrX L3 Mars 62295855-62296900 result	11.19	21.89	10.71	1046
306	monDom5ChrX L3 Mars 37242114-37243161 result	1.72	15.36	13.65	1048
307	monDom5ChrX L3 Mars 68099716-68100777 result	3.20	15.25	12.05	1062
308	monDom5ChrX L3 Mars 34907366-34908431 result	2.63	16.23	13.60	1066
309	monDom5ChrX L3 Mars 55661240-55662314 result	2.70	20.19	17.49	1075
310	monDom5ChrX L3 Mars 33277019-33278100 result	5.08	20.33	15.25	1082
311	monDom5ChrX L3 Mars 22145485-22146571 result	4.42	21.25	16.84	1087
312	monDom5ChrX L3 Mars 53342588-53343725 result	5.01	15.99	10.98	1138
313	monDom5ChrX L3 Mars 4716545-4717695 result	6.34	17.46	11.12	1151
314	monDom5ChrX L3 Mars 42803411-42804562 result	5.30	21.79	16.49	1152
315	monDom5ChrX L3 Mars 52960152-52961311 result	5.95	25.52	19.57	1160
316	monDom5ChrX Plat L3 33740234-33741411 result	3.23	14.18	10.95	1178
317	monDom5ChrX L3 Mars 30946580-30947801 result	3.85	18.00	14.16	1222
318	monDom5ChrX L3 Mars 29702673-29703901 result	3.99	20.83	16.84	1229
319	monDom5ChrX L3 Mars 32551981-32553227 result	5.45	22.37	16.92	1247
320	monDom5ChrX L3 Mars 19123423-19124714 result	2.94	20.90	17.96	1292
321	monDom5ChrX L3 Mars 41805965-41807297 result	2.03	18.98	16.95	1333
322	monDom5ChrX L3 Mars 61690017-61691384 result	3.29	18.86	15.57	1368
323	monDom5ChrX L3 Mars 39904263-39905639 result	3.63	21.21	17.57	1377
324	monDom5ChrX L3 Mars 18847706-18849241 result	8.40	13.61	5.21	1536
325	monDom5ChrX L3 Mars 62602304-62603860 result	4.69	14.64	9.96	1557
326	monDom5ChrX L3b 57133959-57135602 result	4.32	20.92	16.61	1644
327	monDom5ChrX L3 Mars 50149599-50151258 result	4.64	17.47	12.83	1660
328	monDom5ChrX L3 Mars 50459859-50461574 result	5.01	20.80	15.79	1716
329	monDom5ChrX L3 Mars 73917686-73919573 result	5.24	22.09	16.84	1888
330	monDom5ChrX L3 Mars 76852976-76855089 result	4.78	16.93	12.16	2114
331	monDom5ChrX L3 Mars 19697939-19700085 result	5.78	21.01	15.23	2147
332	monDom5ChrX L3 Mars 17680554-17682724 result	5.39	20.31	14.92	2171
333	monDom5ChrX L3 Mars 18406631-18409028 result	5.17	19.27	14.10	2398
334	monDom5ChrX L3 Mars 43090500-43093332 result	6.35	18.96	12.60	2833
335	monDom5ChrX L3 Mars 36608794-36612083 result	4.80	15.47	10.67	3290
336	monDom5ChrX L3 Mars 49700577-49703883 result	4.48	15.72	11.25	3307

212	monDom5ChrX RTE Mdo 10780003-10780	1.81	4.87	3.06	718
213	monDom5ChrX RTE1 Mars 18123947-181	5.97	19.31	13.33	720
214	monDom5ChrX RTE1 Mars 55706540-557	4.29	10.25	5.96	722
215	monDom5ChrX RTE1 Mars 63674567-636	5.81	16.32	10.51	723
216	monDom5ChrX RTE1 Mars 17228254-172	5.03	10.34	5.31	735
217	monDom5ChrX RTE1 Mars 9166726-9167	7.76	12.79	5.03	735
218	monDom5ChrX RTE1 Mars 59512523-595	2.02	14.29	12.26	742
219	monDom5ChrX RTE1 Mars 23710866-237	2.54	14.82	12.28	749
220	monDom5ChrX RTE1 Mars 76225543-762	5.20	11.47	6.27	750
221	monDom5ChrX RTE1 Mars 63038386-630	3.57	12.96	9.39	756
222	monDom5ChrX RTE1 Mars 10528606-105	7.91	8.17	0.26	759
223	monDom5ChrX RTE1 Mars 35742737-357	4.73	19.97	15.24	761
224	monDom5ChrX RTE1 Mars 46522914-465	6.41	10.60	4.19	764
225	monDom5ChrX RTE1 Mars 46783243-467	3.26	18.25	14.99	767
226	monDom5ChrX RTE1 Mars 6539982-6540	8.87	10.82	1.96	767
227	monDom5ChrX RTE Mdo 24584442-24585	1.69	4.56	2.86	768
228	monDom5ChrX RTE1 Mars 55017680-550	2.73	6.88	4.16	770
229	monDom5ChrX RTE1 Mars 36576452-365	4.27	15.80	11.53	772
230	monDom5ChrX RTE1 Mars 62443961-624	6.56	9.91	3.35	777
231	monDom5ChrX RTE1 Mars 19083444-190	4.19	17.01	12.82	788
232	monDom5ChrX RTE1 Mars 63675302-636	3.05	19.54	16.50	788
233	monDom5ChrX RTE1 Mars 61458895-614	6.96	8.61	1.65	790
234	monDom5ChrX RTE1 Mars 74903569-749	2.51	10.78	8.27	798
235	monDom5ChrX RTE Mdo 76383362-76384	3.88	8.90	5.01	798
236	monDom5ChrX RTE1 Mars 65387735-653	6.13	10.14	4.01	799
237	monDom5ChrX RTE1 Mars 17473436-174	5.20	14.00	8.80	807
238	monDom5ChrX RTE1 Mars 40007284-400	7.42	11.12	3.71	809
239	monDom5ChrX RTE1 Mars 49813797-498	7.88	12.32	4.43	812
240	monDom5ChrX RTE1 Mars 46712039-467	6.76	14.25	7.49	814
241	monDom5ChrX RTE1 Mars 48183781-481	1.59	12.62	11.03	816
242	monDom5ChrX RTE1 Mars 16057980-160	8.28	11.81	3.53	821
243	monDom5ChrX RTE1 Mars 35463227-354	2.18	19.54	17.35	824
244	monDom5ChrX RTE1 Mars 48876595-488	4.36	9.81	5.45	826
245	monDom5ChrX RTE1 Mars 34507208-345	3.26	17.25	13.99	829
246	monDom5ChrX RTE1 Mars 52886643-528	5.43	15.92	10.49	829
247	monDom5ChrX RTE1 Mars 15774283-157	7.47	11.69	4.22	830
248	monDom5ChrX RTE1 Mars 48551619-485	6.99	10.60	3.61	830
249	monDom5ChrX RTE1 Mars 15870148-158	5.76	15.25	9.48	833
250	monDom5ChrX RTE1 Mars 37049657-370	4.26	12.31	8.05	845
251	monDom5ChrX RTE1 Mars 21391317-213	3.53	18.73	15.19	849
252	monDom5ChrX RTE1 Mars 15826980-158	5.52	14.81	9.28	851
253	monDom5ChrX RTE1 Mars 50735990-507	7.37	11.70	4.33	855
254	monDom5ChrX RTE1 Mars 29736745-297	5.21	17.96	12.75	863
255	monDom5ChrX RTE1 Mars 15979243-159	4.50	13.26	8.77	867
256	monDom5ChrX RTE1 Mars 50016011-500	3.00	14.17	11.18	868
257	monDom5ChrX RTE1 Mars 56611411-566	7.14	19.47	12.33	868
258	monDom5ChrX RTE1 Mars 10072784-100	7.67	7.21	-0.46	874
259	monDom5ChrX RTE1 Mars 32743236-327	5.60	18.06	12.46	875
260	monDom5ChrX RTE1 Mars 18210688-182	7.99	13.24	5.25	876
261	monDom5ChrX RTE1 Mars 32818121-328	1.36	14.59	13.24	884
262	monDom5ChrX RTE1 Mars 34494896-344	7.08	7.42	0.34	890
263	monDom5ChrX RTE1 Mars 18237512-182	3.82	13.80	9.99	891
264	monDom5ChrX RTE1 Mars 17973200-179	2.91	17.15	14.24	892
265	monDom5ChrX RTE1 Mars 35819489-358	3.02	15.31	12.29	895
266	monDom5ChrX RTE1 Mars 38492647-384	3.46	8.26	4.80	896
267	monDom5ChrX RTE1 Mars 52019961-520	8.66	13.32	4.66	901
268	monDom5ChrX RTE1 Mars 19305359-193	5.41	12.36	6.95	906
269	monDom5ChrX RTE1 Mars 57701843-577	5.85	14.68	8.83	906
270	monDom5ChrX RTE1 Mars 44617158-446	6.84	13.78	6.95	907
271	monDom5ChrX RTE1 Mars 39362268-393	4.80	18.78	13.97	916
272	monDom5ChrX RTE1 Mars 60575703-605	4.02	13.48	9.46	920
273	monDom5ChrX RTE1 Mars 54913821-549	4.11	13.08	8.97	925
274	monDom5ChrX RTE1 Mars 46829407-468	5.18	10.68	5.50	927
275	monDom5ChrX RTE1 Mars 26950274-269	8.84	12.93	4.09	928
276	monDom5ChrX RTE1 Mars 33577443-335	6.20	13.90	7.70	935
277	monDom5ChrX RTE1 Mars 51996643-519	6.28	14.04	7.77	940
278	monDom5ChrX RTE1 Mars 17226763-172	4.03	12.42	8.39	942
279	monDom5ChrX RTE1 Mars 18334142-183	3.29	17.30	14.01	942
280	monDom5ChrX RTE1 Mars 50435325-504	6.22	12.66	6.43	948
281	monDom5ChrX RTE1 Mars 50852042-508	5.48	8.43	2.95	949
282	monDom5ChrX RTE Mdo 21792866-21793	3.79	9.68	5.89	950
283	monDom5ChrX RTE1 Mars 33455905-334	3.67	18.45	14.78	954
284	monDom5ChrX RTE1 Mars 43866300-438	5.65	13.72	8.06	955
285	monDom5ChrX RTE1 Mars 58542893-585	4.39	15.06	10.67	956
286	monDom5ChrX RTE1 Mars 59582328-595	4.28	11.39	7.11	957
287	monDom5ChrX RTE1 Mars 37216106-372	4.80	13.14	8.34	959
288	monDom5ChrX RTE1 Mars 31591809-315	5.40	18.07	12.67	963
289	monDom5ChrX RTE1 Mars 45089286-450	4.66	13.68	9.02	965
290	monDom5ChrX RTE1 Mars 35035874-350	4.45	16.15	11.70	966
291	monDom5ChrX RTE1 Mars 57789304-577	6.07	12.55	6.48	972
292	monDom5ChrX RTE1 Mars 33947307-339	2.98	16.55	13.57	973
293	monDom5ChrX RTE1 Mars 39537600-395	6.27	13.85	7.58	989
294	monDom5ChrX RTE1 Mars 57810234-578	1.31	12.34	11.02	989
295	monDom5ChrX RTE1 Mars 53093538-530	5.34	10.08	4.74	992
296	monDom5ChrX RTE1 Mars 44994821-449	3.12	15.98	12.86	995
297	monDom5ChrX RTE1 Mars 11793622-117	4.91	10.89	5.99	1019
298	monDom5ChrX RTE1 Mars 65492773-654	5.93	8.84	2.92	1029
299	monDom5ChrX RTE1 Mars 18575410-185	4.95	6.64	1.68	1070
300	monDom5ChrX RTE1 Mars 18347279-183	5.42	17.55	12.14	1071
301	monDom5ChrX RTE Mdo 32712647-32713	3.06	5.57	2.51	1077
302	monDom5ChrX RTE Mdo 8450261-845134	3.88	11.36	7.48	1083
303	monDom5ChrX RTE1 Mars 49290273-492	4.51	15.10	10.59	1086
304	monDom5ChrX RTE1 Mars 32702830-327	6.10	13.39	7.29	1098
305	monDom5ChrX RTE1 Mars 57592267-575	6.26	10.81	4.56	1119
306	monDom5ChrX RTE Mdo 3746988-374813	2.36	5.24	2.88	1146
307	monDom5ChrX RTE1 Mars 48878150-488	5.71	13.68	7.97	1155
308	monDom5ChrX RTE Mdo 8562248-856341	4.47	5.07	0.60	1163
309	monDom5ChrX RTE Mdo 58176347-58177	2.82	5.12	2.31	1171
310	monDom5ChrX RTE1 Mars 78029981-780	3.54	16.60	13.06	1271
311	monDom5ChrX RTE1 Mars 60775848-607	2.90	14.58	11.68	1310
312	monDom5ChrX RTE1 Mars 34678766-346	5.56	13.01	7.46	1314
313	monDom5ChrX RTE1 Mars 71190689-711	5.39	14.73	9.34	1317
314	monDom5ChrX RTE1 Mars 62844596-628	3.48	11.35	7.87	1322
315	monDom5ChrX RTE1 Mars 9273697-9275	5.11	12.10	6.98	1389
316	monDom5ChrX RTE Mdo 3906016-390741	4.92	10.83	5.92	1403
317	monDom5ChrX RTE1 Mars 9410835-9412	4.09	13.04	8.95	1442
318	monDom5ChrX RTE Mdo 24722500-24723	2.68	5.49	2.82	1456
319	monDom5ChrX RTE Mdo 74944105-74945	2.79	8.71	5.92	1504
320	monDom5ChrX RTE Mdo 6291175-629291	2.82	6.97	4.15	1736
321	monDom5ChrX RTE1 Mars 0622890-7062	4.25	13.23	8.98	1860
322	monDom5ChrX RTE Mdo 12809011-12810	3.21	7.04	3.83	1933
323	monDom5ChrX RTE-2 MD 4269779-42717	6.23	11.08	4.85	1959
324	monDom5ChrX RTE Mdo 48314542-48316	3.20	7.26	4.06	1969
325	monDom5ChrX RTE1 Mars 27073910-270	3.19	14.32	11.13	1976
326	monDom5ChrX RTE Mdo 27181085-27183	3.64	7.28	3.64	1978
327	monDom5ChrX RTE Mdo 4150114-415212	3.83	7.01	3.18	2011
328	monDom5ChrX RTE1 Mars 17387044-173	4.50	13.07	8.56	2020
329	monDom5ChrX RTE Mdo 57470798-57472	3.96	7.32	3.36	2021
330	monDom5ChrX RTE1 Mars 56976165-569	4.30	13.62	9.33	2048
331	monDom5ChrX RTE Mdo 75479450-75481	3.10	7.79	4.70	2066
332	monDom5ChrX RTE Mdo 49989928-49992	3.35	9.05	5.70	2088
333	monDom5ChrX RTE1 Mars 33536724-335	4.28	14.32	10.04	2102
334	monDom5ChrX RTE1 Mars 5912290-5914	6.36	15.84	9.49	2140
335	monDom5ChrX RTE Mdo 63046302-63048	4.22	7.88	3.66	2157
336	monDom5ChrX RTE1 Mars 68685443-686	5.65	15.98	10.33	2159
337	monDom5ChrX RTE Mdo 76381185-76383	3.54	7.63	4.09	2175
338	monDom5ChrX RTE1 Mars 35038885-350	3.97	12.80	8.84	2218
339	monDom5ChrX RTE1 Mars 49719499-497	6.45	14.52	8.07	2355
340	monDom5ChrX RTE Mdo 5896869-589927	3.11	8.79	5.68	2411
341	monDom5ChrX RTE Mdo 60875347-60877	3.84	8.80	4.96	2421
342	monDom5ChrX RTE-2 MD 4451626-44541	8.06	12.26	4.19	2480
343	monDom5ChrX RTE Mdo 15138067-15140	2.74	8.46	5.72	2519
344	monDom5ChrX RTE Mdo 32899413-32902	3.40	8.65	5.25	2647
345	monDom5ChrX RTE Mdo 6061825-160644	3.21	8.68	5.47	2651
346	monDom5ChrX RTE Mdo 10777316-10780	3.24	10.60	7.36	2689
347	monDom5ChrX RTE Mdo 41146203-41148	3.03	7.91	4.89	2743
348	monDom5ChrX RTE Mdo 33058310-33061	3.47	8.33	4.86	2797
349	monDom5ChrX RTE Mdo 48805683-48808	3.97	9.77	5.80	2897
350	monDom5ChrX RTE Mdo 66123483-66126	2.66	8.53	5.87	2931
351	monDom5ChrX RTE Mdo 55246937-55249	3.37	10.38	7.01	3024
352	monDom5ChrX RTE Mdo 37130764-37133	3.07	8.42	5.36	3230
353	monDom5ChrX RTE Mdo 14140809-14144	3.63	9.16	5.53	3254
354	monDom5ChrX RTE Mdo 8508919-851224	3.25	9.39	6.14	3322
355	monDom5ChrX RTE Mdo 75564658-75568	3.43	8.69	5.26	3590
356	monDom5ChrX RTE Mdo 28404682-28408	3.20	8.36	5.17	3599
357	monDom5ChrX RTE Mdo 61762413-61766	3.11	8.29	5.18	3630
358	monDom5ChrX RTE Mdo 19967246-19971	3.65	8.15	4.50	3780
359	monDom5ChrX RTE Mdo 19493993-19497	3.37	9.43	6.06	3796
360	monDom5ChrX RTE Mdo 22115235-22119	3.33	9.21	5.88	3909
361	monDom5ChrX RTE Mdo 11975927-11979	2.97	8.12	5.15	3941
362	monDom5ChrX RTE Mdo 29602753-29606	3.11	8.60	5.49	3954
363	monDom5ChrX RTE Mdo 50074304-50078	2.87	9.28	6.42	3975
364	monDom5ChrX RTE Mdo 67527553-67531	4.39	7.95	3.57	4011
365	monDom5ChrX RTE Mdo 16560030-16564	4.31	8.60	4.29	4013
366	monDom5ChrX RTE Mdo 62917720-62921	3.17	8.48	5.31	4068
367	monDom5ChrX RTE Mdo 12177927-12181	3.24	8.55	5.31	4071
368	monDom5ChrX RTE Mdo 31945322-31949	3.72	8.69	4.97	4084
369	monDom5ChrX RTE Mdo 5252783-525687	2.88	8.48	5.60	4091
370	monDom5ChrX RTE Mdo 53721059-53725	3.20	8.86	5.67	4095
371	monDom5ChrX RTE Mdo 16643557-16647	3.15	8.69	5.54	4097
