# Supplementary material for: Convergence in LINE-1 nucleotide variations can benefit redundantly forming triplexes with lncRNA in mammalian X-chromosome inactivation
Source: Mob DNA. 2019 Jul 30;10:33. doi: 10.1186/s13100-019-0173-4 (PMC6664574; doi:10.1186/s13100-019-0173-4)

Additional file 7: Mapping and length-distributions of r-TC/r-AG motifs in other representative L1 subfamilies of the three species

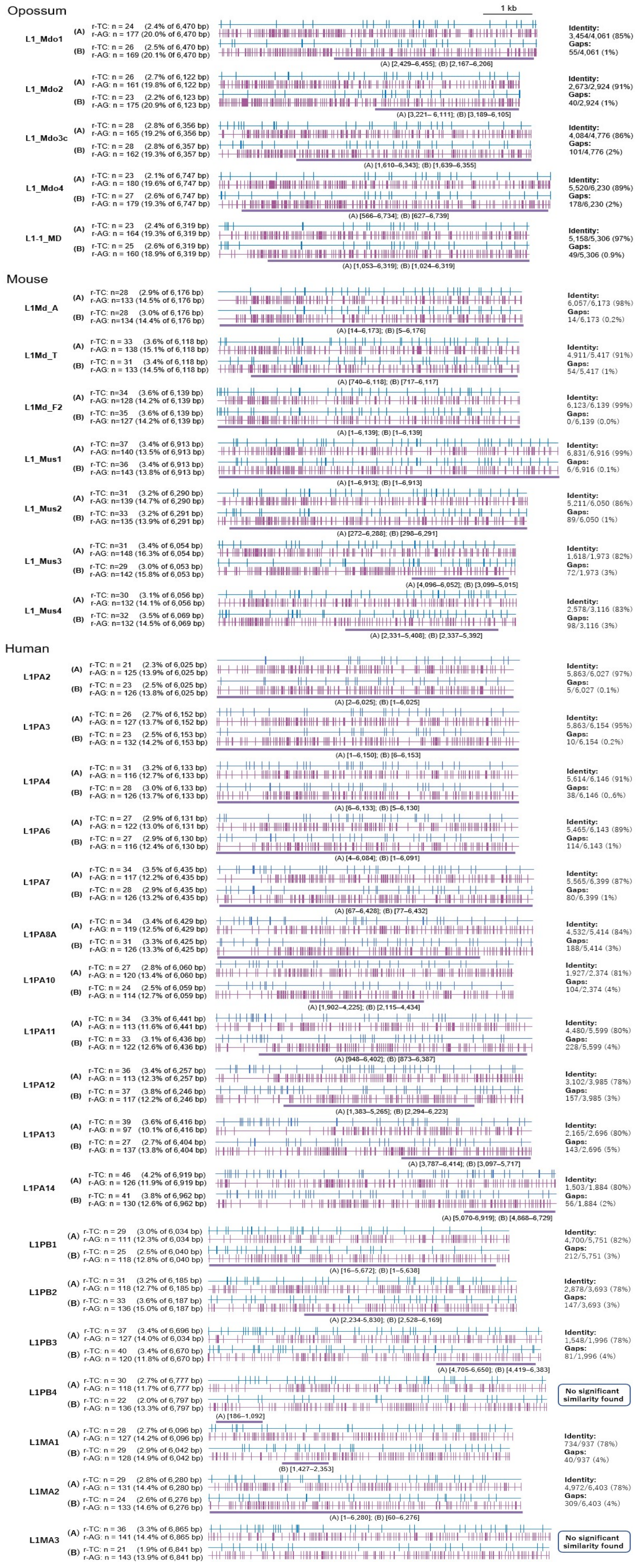

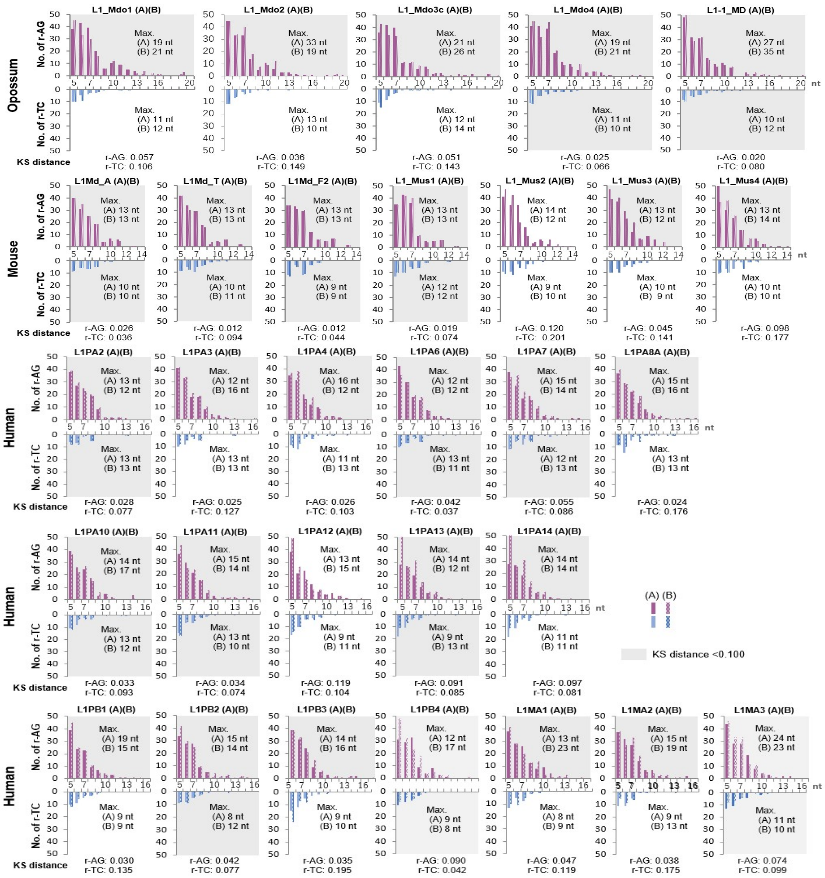

Supplement: Supplementary file 7 — Mapping and length–distributions of r-TC/r-AG motifs in other representative L1 subfamilies of the three species. (PDF 1719 kb) [file 13100_2019_173_MOESM7_ESM.pdf]
