# Supplementary material for: Convergence in LINE-1 nucleotide variations can benefit redundantly forming triplexes with lncRNA in mammalian X-chromosome inactivation
Source: Mob DNA. 2019 Jul 30;10:33. doi: 10.1186/s13100-019-0173-4 (PMC6664574; doi:10.1186/s13100-019-0173-4)
Supplement: Supplementary file 8 — Lists of r-TC/r-AG motifs in paired L1s of the representative subfamilies of the three species. The following tables list the r-TC/r-AG motifs in the human paired L1s depicted in Fig. 7b, and also in the paired L1MD_T (mouse) and L1_Mdo4 (opossum) that show moderate-to-high sequence identities (91 and 89%, respectively). (PDF 699 kb) [file 13100_2019_173_MOESM8_ESM.pdf]

Additional file 8: Lists of r-TC/r-AG motifs in paired L1s of representative subfamilies of the three species

The following tables list the r-TC/r-AG motifs in the human paired L1s depicted in Figure 7b, and also in the paired L1Md_T (mouse) and L1_Mdo4 (opossum) that show moderate-to-high sequence identities (91% and 89%, respectively).

Paired L1HS (A)

					r-TC in hg38					r-AG in hg38			r-TC in L1HS(A)			r-AG in L1HS(A)	
Subfamily	Chr:Positions	No.	r-TC Sequence	Length	Start Position	End Position	No.	r-AG Sequence	Length	Start Position	End Position	No.	Star Position	End position	No.	Star Position	End position
L1HS	ChrX:106469286-106475319	1	TTTCT	5	106469363	106469367	1	AGGAA	5	106469310	106469314	1	78	82	1	25	29
		2	CCTCTCCTCCTCC	13	106470239	106470251	2	AGAAGA	6	106469350	106469355	2	954	966	2	65	70
		3	TTCCTC	6	106470264	106470269	3	AAAGAAAGG	9	106469522	106469530	3	979	984	3	237	245
		4	TCTCTC	6	106470945	106470950	4	AAGAAA	6	106469596	106469601	4	1660	1665	4	311	316
		5	TTCTT	5	106470998	106471002	5	AAGGAGG	7	106469803	106469809	5	1713	1717	5	518	524
		6	TCTCT	5	106471502	106471506	6	AAAGA	5	106469861	106469865	6	2217	2221	6	576	580
		7	CTCTCCTC	8	106471904	106471911	7	GAAGAGAG	8	106469911	106469918	7	2619	2626	7	626	633
		8	TCTCTC	6	106471951	106471956	8	AGAAGGAAA	9	106470096	106470104	8	2666	2671	8	811	819
		9	TTCTTT	6	106472090	106472095	9	AGAAAGGA	8	106470115	106470122	9	2805	2810	9	830	837
		10	TCTCT	5	106472131	106472135	10	AAAGA	5	106470161	106470165	10	2846	2850	10	876	880
		11	TTCCTC	6	106472567	106472572	11	AAAGA	5	106470186	106470190	11	3282	3287	11	901	905
		12	TCTCT	5	106472615	106472619	12	AGAAA	5	106470208	106470212	12	3330	3334	12	923	927
		13	TTCCTTCT	8	106472744	106472751	13	AAAGGAA	7	106470252	106470258	13	3459	3466	13	967	973
		14	TCCTT	5	106472873	106472877	14	GAGAGAAGAAGG	12	106470315	106470326	14	3588	3592	14	1030	1041
		15	TCTTT	5	106473084	106473088	15	AAAGAAG	7	106470379	106470385	15	3799	3803	15	1094	1100
		16	CCTCTCTC	8	106473257	106473264	16	AGAAGAA	7	106470408	106470414	16	3972	3979	16	1123	1129
		17	TCTCCTT	7	106473424	106473430	17	AGAGAAG	7	106470438	106470444	17	4139	4145	17	1153	1159
		18	TTCTT	5	106473494	106473498	18	AAAGGAG	7	106470450	106470456	18	4209	4213	18	1165	1171
		19	CTTTCTTC	8	106473777	106473784	19	GAAGAA	6	106470493	106470498	19	4492	4499	19	1208	1213
		20	TCTTT	5	106474020	106474024	20	GGAAGAAAGG	10	106470531	106470540	20	4735	4739	20	1246	1255
		21	CCTTCCTT	8	106474125	106474132	21	AGAGAAA	7	106470592	106470598	21	4840	4847	21	1307	1313
		22	TTCCTC	6	106474751	106474756	22	AAAGAA	6	106470599	106470604	22	5466	5471	22	1314	1319
		23	TCCTTT	6	106474997	106475002	23	AAAGAAA	7	106470608	106470614	23	5712	5717	23	1323	1329
							24	AAGAAA	6	106470629	106470634				24	1344	1349
							25	AAAGA	5	106470650	106470654				25	1365	1369
							26	AGGAGAA	7	106470737	106470743				26	1452	1458
							27	AGGAAA	6	106470782	106470787				27	1497	1502
							28	AGAGAA	6	106470791	106470796				28	1506	1511
							29	AAAGA	5	106470803	106470807				29	1518	1522
							30	GAGAAGAG	8	106470816	106470823				30	1531	1538
							31	GAAGGAAA	8	106470867	106470874				31	1582	1589
							32	AGAGAGAAAGG	11	106470892	106470902				32	1607	1617
							33	AAAGGAAAG	9	106470916	106470924				33	1631	1639
							34	AGAAA	5	106470954	106470958				34	1669	1673
							35	AGAAGAGAG	9	106470970	106470978				35	1685	1693
							36	AAAGAAA	7	106471003	106471009				36	1718	1724
							37	GAAGGAGAAA	10	106471060	106471069				37	1775	1784
							38	GAGAGA	6	106471098	106471103				38	1813	1818
							39	AAAGAG	6	106471129	106471134				39	1844	1849
							40	GAAGGAAG	8	106471140	106471147				40	1855	1862
							41	GGAAAGGAA	9	106471158	106471166				41	1873	1881
							42	AAAGA	5	106471205	106471209				42	1920	1924
							43	AGGAAGAAA	9	106471221	106471229				43	1936	1944
							44	AAAGA	5	106471337	106471341				44	2052	2056
							45	AAAGAG	6	106471362	106471367				45	2077	2082
							46	AGGAAA	6	106471394	106471399				46	2109	2114
							47	AGAGA	5	106471414	106471418				47	2129	2133
							48	AAAGGA	6	106471438	106471443				48	2153	2158
							49	GGAGGAAGA	9	106471445	106471453				49	2160	2168
							50	AAAGA	5	106471530	106471534				50	2245	2249
							51	AAAGAGA	7	106471538	106471544				51	2253	2259
							52	AAAGAAGG	8	106471546	106471553				52	2261	2268
							53	AAGAGGAG	8	106471586	106471593				53	2301	2308
							54	AAAGAGA	7	106471667	106471673				54	2382	2388
							55	AGAAAG	6	106471745	106471750				55	2460	2465
							56	AAGGA	5	106471756	106471760				56	2471	2475
							57	AGGAA	5	106471766	106471770				57	2481	2485
							58	AAAGAA	6	106471922	106471927				58	2637	2642
							59	AGAAA	5	106471929	106471933				59	2644	2648
							60	AAGAA	5	106471991	106471995				60	2706	2710
							61	AGAAA	5	106472077	106472081				61	2792	2796
							62	AAAGA	5	106472083	106472087				62	2798	2802
							63	AAAGA	5	106472111	106472115				63	2826	2830
							64	AAGAGAAAG	9	106472189	106472197				64	2904	2912
							65	AGGAAGGA	8	106472199	106472206				65	2914	2921
							66	AAAGAA	6	106472237	106472242				66	2952	2957
							67	AGAAA	5	106472245	106472249				67	2960	2964
							68	AAGAAA	6	106472286	106472291				68	3001	3006
							69	GAAGGAAA	8	106472314	106472321				69	3029	3036
							70	AGAGA	5	106472323	106472327				70	3038	3042
							71	GAAAGGA	7	106472375	106472381				71	3090	3096
							72	AAAGAAA	7	106472416	106472422				72	3131	3137
							73	AAAGAGAGAAGAA	13	106472424	106472436				73	3139	3151
							74	AGAAA	5	106472488	106472492				74	3203	3207
							75	AGAGAA	6	106472507	106472512				75	3222	3227
							76	AGAAA	5	106472542	106472546				76	3257	3261
							77	AGAAGAAA	8	106472551	106472558				77	3266	3273
							78	AGGAAGAAG	9	106472601	106472609				78	3316	3324
							79	AAAGAG	6	106472677	106472682				79	3392	3397
							80	AAGGAGGAA	9	106472726	106472734				80	3441	3449
							81	AGAAA	5	106472770	106472774				81	3485	3489
							82	AGAGA	5	106472837	106472841				82	3552	3556
							83	AAAGAGAA	8	106472852	106472859				83	3567	3574
							84	AAAGA	5	106473041	106473045				84	3756	3760
							85	AGAAA	5	106473076	106473080				85	3791	3795
							86	AGGAGAAGGAAA	12	106473315	106473326				86	4030	4041
							87	AGGAAA	6	106473343	106473348				87	4058	4063
							88	AGAGGAAG	8	106473349	106473356				88	4064	4071
							89	AGAAA	5	106473397	106473401				89	4112	4116
							90	AGAGAG	6	106473520	106473525				90	4235	4240
							91	AAAGAGAA	8	106473562	106473569				91	4277	4284
							92	AGGAA	5	106473580	106473584				92	4295	4299
							93	GAAGGA	6	106473604	106473609				93	4319	4324
							94	AAGGAGAA	8	106473617	106473624				94	4332	4339
							95	AAAGAGGA	8	106473650	106473657				95	4365	4372
							96	GGAAGAA	7	106473669	106473675				96	4384	4390
							97	AGGAAGAA	8	106473694	106473701				97	4409	4416
							98	AAAGAG	6	106473828	106473833				98	4543	4548
							99	AAAGAA	6	106473862	106473867				99	4577	4582
							100	AGAGA	5	106473953	106473957				100	4668	4672
							101	AGAAA	5	106473988	106473992				101	4703	4707
							102	GAGAAA	6	106474034	106474039				102	4749	4754
							103	GGAAAGGA	8	106474052	106474059				103	4767	4774
							104	AGAAAG	6	106474106	106474111				104	4821	4826
							105	AAAGA	5	106474166	106474170				105	4881	4885
							106	AGAAGAAA	8	106474204	106474211				106	4919	4926
							107	AAGGA	5	106474247	106474251				107	4962	4966
							108	AAAGAG	6	106474321	106474326				108	5036	5041
							109	AGAAGAAA	8	106474339	106474346				109	5054	5061
							110	GGAGAAA	7	106474381	106474387				110	5096	5102
							111	AAGAAA	6	106474457	106474462				111	5172	5177
							112	GAAGGA	6	106474492	106474497				112	5207	5212
							113	AAAGAAGA	8	106474517	106474524				113	5232	5239
							114	AAAGA	5	106474539	106474543				114	5254	5258
							115	GAAGAAA	7	106474549	106474555				115	5264	5270
							116	AGAGAAA	7	106474577	106474583				116	5292	5298
							117	AGGAAA	6	106474648	106474653				117	5363	5368
							118	GGAGAGGA	8	106474665	106474672				118	5380	5387
							119	GGAGAAA	7	106474676	106474682				119	5391	5397
							120	AGGAA	5	106474684	106474688				120	5399	5403
							121	AGAAA	5	106474771	106474775				121	5486	5490
							122	AAAGGA	6	106474816	106474821				122	5531	5536
							123	AAAGA	5	106474841	106474845				123	5556	5560
							124	AAAGA	5	106474888	106474892				124	5603	5607
							125	AAGAAA	6	106474934	106474939				125	5649	5654
							126	AAGAA	5	106475052	106475056				126	5767	5771
							127	AGGAAGG	7	106475123	106475129				127	5838	5844

Paired L1HS (B)

					r-TC in hg38					r-AG in hg38			r-TC in L1HS(B)			r-AG in L1HS(B)	
Subfamily	Chr:Positions	No.	r-TC Sequence	Length	Start Position	End Position	No.	r-AG Sequence	Length	Start Position	End Position	No.	Star Position	End position	No.	Star Position	End position
L1HS	ChrX:147653735-147659767	1	TTTCT	5	147653813	147653817	1	GGAGGAGGAG	10	147653735	147653744	1	79	83	1	1	10
		2	CCTCTCCTCCTCC	13	147654690	147654702	2	AGGAA	5	147653760	147653764	2	956	968	2	26	30
		3	TTCCTC	6	147654715	147654720	3	AGAAGA	6	147653800	147653805	3	981	986	3	66	71
		4	TCTCTC	6	147655396	147655401	4	AGAGAG	6	147653852	147653857	4	1662	1667	4	118	123
		5	TTCTT	5	147655449	147655453	5	AAAGAAAGG	9	147653972	147653980	5	1715	1719	5	238	246
		6	TCTCT	5	147655953	147655957	6	AAGAAA	6	147654046	147654051	6	2219	2223	6	312	317
		7	CTCTCCTC	8	147656355	147656362	7	AAGGAGG	7	147654253	147654259	7	2621	2628	7	519	525
		8	TCTCTC	6	147656398	147656403	8	AAAGA	5	147654311	147654315	8	2664	2669	8	577	581
		9	TTCTTT	6	147656537	147656542	9	GAAGAGAG	8	147654361	147654368	9	2803	2808	9	627	634
		10	TCTCT	5	147656578	147656582	10	AGAAGGAAA	9	147654547	147654555	10	2844	2848	10	813	821
		11	TTCCTC	6	147657014	147657019	11	AGAAAGGA	8	147654566	147654573	11	3280	3285	11	832	839
		12	TCTCT	5	147657062	147657066	12	AAAGA	5	147654612	147654616	12	3328	3332	12	878	882
		13	TTCCTTCT	8	147657191	147657198	13	AAAGA	5	147654637	147654641	13	3457	3464	13	903	907
		14	TCCTT	5	147657320	147657324	14	AGAAA	5	147654659	147654663	14	3586	3590	14	925	929
		15	CCTCTCTC	8	147657704	147657711	15	AAAGGAA	7	147654703	147654709	15	3970	3977	15	969	975
		16	TTCTT	5	147657941	147657945	16	GAGAGAAGAAGG	12	147654766	147654777	16	4207	4211	16	1032	1043
		17	CTTTCTTC	8	147658224	147658231	17	AAAGAAG	7	147654830	147654836	17	4490	4497	17	1096	1102
		18	TCTTT	5	147658467	147658471	18	AGAAGAA	7	147654859	147654865	18	4733	4737	18	1125	1131
		19	CCTTCCTT	8	147658572	147658579	19	AGAGAAG	7	147654889	147654895	19	4838	4845	19	1155	1161
		20	TTCCTC	6	147659198	147659203	20	AAAGGAG	7	147654901	147654907	20	5464	5469	20	1167	1173
		21	TCCTTT	6	147659444	147659449	21	GAAGAA	6	147654944	147654949	21	5710	5715	21	1210	1215
							22	GGAAGAAAGG	10	147654982	147654991				22	1248	1257
							23	AGAGAAA	7	147655043	147655049				23	1309	1315
							24	AAAGAA	6	147655050	147655055				24	1316	1321
							25	AAAGAAA	7	147655059	147655065				25	1325	1331
							26	AAGAAA	6	147655080	147655085				26	1346	1351
							27	AAAGA	5	147655101	147655105				27	1367	1371
							28	AGGAGAA	7	147655188	147655194				28	1454	1460
							29	AGGAAA	6	147655233	147655238				29	1499	1504
							30	AGAGAA	6	147655242	147655247				30	1508	1513
							31	AAAGA	5	147655254	147655258				31	1520	1524
							32	GAGAAGAG	8	147655267	147655274				32	1533	1540
							33	GAAGGAAA	8	147655318	147655325				33	1584	1591
							34	AGAGAGAAAGG	11	147655343	147655353				34	1609	1619
							35	AAAGGAAAG	9	147655367	147655375				35	1633	1641
							36	AGAAA	5	147655405	147655409				36	1671	1675
							37	AGAAGAGAG	9	147655421	147655429				37	1687	1695
							38	AAAGAAA	7	147655454	147655460				38	1720	1726
							39	GAAGGAGAAA	10	147655511	147655520				39	1777	1786
							40	GAGAGA	6	147655549	147655554				40	1815	1820
							41	AAAGAG	6	147655580	147655585				41	1846	1851
							42	GAAGGAAG	8	147655591	147655598				42	1857	1864
							43	GGAAAGGAA	9	147655609	147655617				43	1875	1883

							44	AAAGA	5	147655656	147655660				44	1922	1926
							45	AGGAAGAAA	9	147655672	147655680				45	1938	1946
							46	AAAGA	5	147655788	147655792				46	2054	2058
							47	AAAGAG	6	147655813	147655818				47	2079	2084
							48	AGGAAA	6	147655845	147655850				48	2111	2116
							49	AGAGA	5	147655865	147655869				49	2131	2135
							50	AAAGGA	6	147655889	147655894				50	2155	2160
							51	GGAGGAAGA	9	147655896	147655904				51	2162	2170
							52	AAAGA	5	147655981	147655985				52	2247	2251
							53	AAAGAGA	7	147655989	147655995				53	2255	2261
							54	AAAGAAGG	8	147655997	147656004				54	2263	2270
							55	AAGAGGAG	8	147656037	147656044				55	2303	2310
							56	AAAGAGA	7	147656118	147656124				56	2384	2390
							57	AGAAAG	6	147656196	147656201				57	2462	2467
							58	AAGGA	5	147656207	147656211				58	2473	2477
							59	AGGAA	5	147656217	147656221				59	2483	2487
							60	AAAGAA	6	147656373	147656378				60	2639	2644
							61	AGAAA	5	147656380	147656384				61	2646	2650
							62	AAGAA	5	147656438	147656442				62	2704	2708
							63	AGAAA	5	147656524	147656528				63	2790	2794
							64	AAAGA	5	147656530	147656534				64	2796	2800
							65	AAAGA	5	147656558	147656562				65	2824	2828
							66	AAGAGAAAG	9	147656636	147656644				66	2902	2910
							67	AGGAAAGA	8	147656646	147656653				67	2912	2919
							68	AAAGAA	6	147656684	147656689				68	2950	2955
							69	AGAAA	5	147656692	147656696				69	2958	2962
							70	AAGAAA	6	147656733	147656738				70	2999	3004
							71	GAAGGAAA	8	147656761	147656768				71	3027	3034
							72	AGAAA	5	147656770	147656774				72	3036	3040
							73	GAAAGGA	7	147656822	147656828				73	3088	3094
							74	AAAGAAA	7	147656863	147656869				74	3129	3135
							75	AAAGAGAGAAGAA	13	147656871	147656883				75	3137	3149
							76	AGAAA	5	147656935	147656939				76	3201	3205
							77	AGAGAA	6	147656954	147656959				77	3220	3225
							78	AGAAA	5	147656989	147656993				78	3255	3259
							79	AGAAGAAA	8	147656998	147657005				79	3264	3271
							80	AGGAAGAAG	9	147657048	147657056				80	3314	3322
							81	AAAGAG	6	147657124	147657129				81	3390	3395
							82	AAGGAGGAA	9	147657173	147657181				82	3439	3447
							83	AGAAA	5	147657217	147657221				83	3483	3487
							84	AGAGA	5	147657284	147657288				84	3550	3554
							85	AAAGAGAA	8	147657299	147657306				85	3565	3572
							86	AAAGA	5	147657488	147657492				86	3754	3758
							87	AGAAA	5	147657523	147657527				87	3789	3793
							88	AGGAGAAGGAAA	12	147657762	147657773				88	4028	4039
							89	AGGAAA	6	147657790	147657795				89	4056	4061
							90	AGAGGAAG	8	147657796	147657803				90	4062	4069
							91	AGAAA	5	147657844	147657848				91	4110	4114
							92	AGAGAG	6	147657967	147657972				92	4233	4238
							93	AAAGAGAA	8	147658009	147658016				93	4275	4282
							94	AGGAA	5	147658027	147658031				94	4293	4297
							95	GAAGGA	6	147658051	147658056				95	4317	4322
							96	AAGGAGAA	8	147658064	147658071				96	4330	4337
							97	AAGGAAA	7	147658088	147658094				97	4354	4360
							98	AAAGAGGA	8	147658097	147658104				98	4363	4370
							99	GGAAGAA	7	147658116	147658122				99	4382	4388
							100	AGGAAGAA	8	147658141	147658148				100	4407	4414
							101	AAAGAG	6	147658275	147658280				101	4541	4546
							102	AAAGAA	6	147658309	147658314				102	4575	4580
							103	AGAGA	5	147658400	147658404				103	4666	4670
							104	AGAAA	5	147658435	147658439				104	4701	4705
							105	GAGAAA	6	147658481	147658486				105	4747	4752
							106	GGAAAGGA	8	147658499	147658506				106	4765	4772
							107	AGAAAG	6	147658553	147658558				107	4819	4824
							108	AAAGA	5	147658613	147658617				108	4879	4883
							109	AGAAGAAA	8	147658651	147658658				109	4917	4924
							110	AAGGA	5	147658694	147658698				110	4960	4964
							111	AAAGA	5	147658733	147658737				111	4999	5003
							112	AAAGAG	6	147658768	147658773				112	5034	5039
							113	AAAGAAA	7	147658787	147658793				113	5053	5059
							114	GGAGAAA	7	147658828	147658834				114	5094	5100
							115	AAGAAA	6	147658904	147658909				115	5170	5175
							116	GAAGGA	6	147658939	147658944				116	5205	5210
							117	AAAGAAGA	8	147658964	147658971				117	5230	5237
							118	GAAGAAA	7	147658996	147659002				118	5262	5268
							119	AGAGAAA	7	147659024	147659030				119	5290	5296
							120	AGGAAA	6	147659095	147659100				120	5361	5366
							121	GGAGAGGA	8	147659112	147659119				121	5378	5385
							122	GGAGAAA	7	147659123	147659129				122	5389	5395
							123	AGGAA	5	147659131	147659135				123	5397	5401
							124	AGAAA	5	147659218	147659222				124	5484	5488
							125	AAAGA	5	147659288	147659292				125	5554	5558
							126	AAAGA	5	147659335	147659339				126	5601	5605
							127	AAGAAA	6	147659381	147659386				127	5647	5652
							128	AAGAA	5	147659499	147659503				128	5765	5769
							129	AGGAAGG	7	147659570	147659576				129	5836	5842

Paired L1PA5 (A)

					r-TC in hg38					r-AG in hg38			r-TC in L1PA5(A)			r-AG in L1PA5(A)	
Subfamily	Chr:Positions	No.	r-TC Sequence	Length	Start Position	End Position	No.	r-AG Sequence	Length	Start Position	End Position	No.	Star Position	End position	No.	Star Position	End position
L1PA5	ChrX:69350758-69356897	1	TTTCT	5	69350836	69350840	1	AGGAA	5	69350783	69350787	1	79	83	1	26	30
		2	CCTTTCCT	8	69350984	69350991	2	AGAAGA	6	69350823	69350828	2	227	234	2	66	71
		3	CTCTTT	6	69351139	69351144	3	AGGAAG	6	69350958	69350963	3	382	387	3	201	206
		4	CTCCTT	6	69351860	69351865	4	AGGAGA	6	69351086	69351091	4	1103	1108	4	329	334
		5	CTCCTT	6	69352405	69352410	5	GAAGAGAG	8	69351385	69351392	5	1648	1653	5	628	635
		6	TCTCTT	6	69352540	69352545	6	AGAGGAAAGA	10	69351550	69351559	6	1783	1788	6	793	802
		7	TTCTT	5	69352593	69352597	7	AGAAGGAAA	9	69351692	69351700	7	1836	1840	7	935	943
		8	TCCTTT	6	69352670	69352675	8	AGAAAGGA	8	69351711	69351718	8	1913	1918	8	954	961
		9	TCTCT	5	69353097	69353101	9	AAAGA	5	69351757	69351761	9	2340	2344	9	1000	1004
		10	TCCTT	5	69353247	69353251	10	AGAAA	5	69351804	69351808	10	2490	2494	10	1047	1051
		11	TTCTTCTC	8	69353441	69353448	11	AAAGAAA	7	69351848	69351854	11	2684	2691	11	1091	1097
		12	TCTCTC	6	69353542	69353547	12	GAGAGAAGAAGG	12	69351911	69351922	12	2785	2790	12	1154	1165
		13	TTCTTT	6	69353681	69353686	13	AAAGGAGGAAG	11	69351950	69351960	13	2924	2929	13	1193	1203
		14	TCTCT	5	69353722	69353726	14	AAAGAAG	7	69351975	69351981	14	2965	2969	14	1218	1224
		15	TCCTTC	6	69353925	69353930	15	AAAGA	5	69351996	69352000	15	3168	3173	15	1239	1243
		16	TTCCTC	6	69354155	69354160	16	AGAGAAG	7	69352033	69352039	16	3398	3403	16	1276	1282
		17	TCTCT	5	69354203	69354207	17	GGAAGAAAGG	10	69352126	69352135	17	3446	3450	17	1369	1378
		18	TTCCTTCT	8	69354332	69354339	18	AAAGA	5	69352148	69352152	18	3575	3582	18	1391	1395
		19	CCTCTCTC	8	69354845	69354852	19	AAGAAGAGAAG	11	69352173	69352183	19	4088	4095	19	1416	1426
		20	TCTCCTT	7	69355012	69355018	20	AGAGAAA	7	69352187	69352193	20	4255	4261	20	1430	1436
		21	TTCTT	5	69355082	69355086	21	AAAGAAA	7	69352203	69352209	21	4325	4329	21	1446	1452
		22	CTTTCTTC	8	69355361	69355368	22	AAGAAA	6	69352224	69352229	22	4604	4611	22	1467	1472
		23	TCTTT	5	69355604	69355608	23	AAAGA	5	69352245	69352249	23	4847	4851	23	1488	1492
		24	CCTTCCTT	8	69355709	69355716	24	AGGAGAA	7	69352332	69352338	24	4952	4959	24	1575	1581
		25	TCCTT	5	69356215	69356219	25	AAGAAA	6	69352377	69352382	25	5458	5462	25	1620	1625
		26	TTCCTC	6	69356331	69356336	26	AGAGAA	6	69352386	69352391	26	5574	5579	26	1629	1634
		27	TCCTTT	6	69356577	69356582	27	AAAGA	5	69352398	69352402	27	5820	5825	27	1641	1645
		28	TCTTTCTT	8	69356611	69356618	28	GAGAAGAG	8	69352411	69352418	28	5854	5861	28	1654	1661
							29	GAAGGAAA	8	69352462	69352469				29	1705	1712
							30	AGAAA	5	69352487	69352491				30	1730	1734
							31	AAAGAGAAG	9	69352511	69352519				31	1754	1762
							32	AGAAA	5	69352549	69352553				32	1792	1796
							33	AAAGAAA	7	69352598	69352604				33	1841	1847
							34	GAAGGAGAAA	10	69352655	69352664				34	1898	1907
							35	GAGAGA	6	69352693	69352698				35	1936	1941
							36	AAAGAG	6	69352724	69352729				36	1967	1972
							37	GAAGGAAG	8	69352735	69352742				37	1978	1985
							38	GGAAAGGAA	9	69352753	69352761				38	1996	2004
							39	AAAGA	5	69352800	69352804				39	2043	2047
							40	AGGAAGAAA	9	69352816	69352824				40	2059	2067
							41	AAAGA	5	69352932	69352936				41	2175	2179
							42	AAAGAG	6	69352957	69352962				42	2200	2205
							43	AGGAAA	6	69352989	69352994				43	2232	2237
							44	AGAGA	5	69353009	69353013				44	2252	2256
							45	AAAGGA	6	69353033	69353038				45	2276	2281
							46	GGAGGAAGA	9	69353040	69353048				46	2283	2291
							47	AAAGA	5	69353125	69353129				47	2368	2372
							48	AAAGAGA	7	69353133	69353139				48	2376	2382
							49	AAAGAAGG	8	69353141	69353148				49	2384	2391
							50	AAGAAGAG	8	69353181	69353188				50	2424	2431
							51	AGAGA	5	69353252	69353256				51	2495	2499
							52	AAAGAGA	7	69353262	69353268				52	2505	2511
							53	AGAAAG	6	69353340	69353345				53	2583	2588
							54	AAGGA	5	69353351	69353355				54	2594	2598
							55	AGGAA	5	69353361	69353365				55	2604	2608
							56	AAGAGAA	7	69353428	69353434				56	2671	2677
							57	AAAGAA	6	69353517	69353522				57	2760	2765
							58	AGAAA	5	69353524	69353528				58	2767	2771
							59	AAGAAA	6	69353582	69353587				59	2825	2830
							60	AGAAA	5	69353668	69353672				60	2911	2915
							61	AAAGA	5	69353674	69353678				61	2917	2921
							62	AAAGA	5	69353702	69353706				62	2945	2949
							63	AAGAGAAAG	9	69353780	69353788				63	3023	3031
							64	AGGAAAGA	8	69353790	69353797				64	3033	3040
							65	GAAAGAA	7	69353827	69353833				65	3070	3076
							66	AGAGAAG	7	69353836	69353842				66	3079	3085
							67	AAGAAA	6	69353877	69353882				67	3120	3125
							68	GAAGGAGA	8	69353905	69353912				68	3148	3155
							69	AGAGA	5	69353914	69353918				69	3157	3161
							70	AAAGA	5	69353965	69353969				70	3208	3212
							71	AAAGAAGAAA	10	69354004	69354013				71	3247	3256
							72	AGAGAGAAGAA	11	69354014	69354024				72	3257	3267
							73	AGAAA	5	69354076	69354080				73	3319	3323
							74	AGAGAA	6	69354095	69354100				74	3338	3343
							75	AGAAA	5	69354130	69354134				75	3373	3377
							76	AGAAGAAA	8	69354139	69354146				76	3382	3389
							77	AGGAAGAAG	9	69354189	69354197				77	3432	3440
							78	AAGGAGGAG	9	69354314	69354322				78	3557	3565
							79	AGAAA	5	69354358	69354362				79	3601	3605
							80	AGAGA	5	69354425	69354429				80	3668	3672
							81	AAAGAGAA	8	69354440	69354447				81	3683	3690
							82	AAAGA	5	69354629	69354633				82	3872	3876
							83	AGGAGAAAGAAA	12	69354903	69354914				83	4146	4157
							84	AGGAAA	6	69354931	69354936				84	4174	4179
							85	AGAGGAAG	8	69354937	69354944				85	4180	4187
							86	AGAAA	5	69354985	69354989				86	4228	4232
							87	AGAGAG	6	69355104	69355109				87	4347	4352
							88	GAAGAGAA	8	69355146	69355153				88	4389	4396
							89	AGGAA	5	69355164	69355168				89	4407	4411
							90	GAAGGA	6	69355188	69355193				90	4431	4436
							91	AAGGAGAA	8	69355201	69355208				91	4444	4451
							92	AAAGAGGA	8	69355234	69355241				92	4477	4484
							93	GGAAGAA	7	69355253	69355259				93	4496	4502
							94	AGGAAGAA	8	69355278	69355285				94	4521	4528

							95	AAAGAG	6	69355412	69355417				95	4655	4660
							96	AAAGAA	6	69355446	69355451				96	4689	4694
							97	AGAGA	5	69355537	69355541				97	4780	4784
							98	AGAAA	5	69355572	69355576				98	4815	4819
							99	AAGAAA	6	69355627	69355632				99	4870	4875
							100	GGAAAGGA	8	69355636	69355643				100	4879	4886
							101	AAAGA	5	69355750	69355754				101	4993	4997
							102	AGAAGAAA	8	69355788	69355795				102	5031	5038
							103	AAGGA	5	69355831	69355835				103	5074	5078
							104	AAAGAG	6	69355905	69355910				104	5148	5153
							105	AAAGAAA	7	69355924	69355930				105	5167	5173
							106	GGAGAAA	7	69355965	69355971				106	5208	5214
							107	AAGAAA	6	69356041	69356046				107	5284	5289
							108	GAAGGA	6	69356077	69356082				108	5320	5325
							109	AAAGAAGA	8	69356097	69356104				109	5340	5347
							110	AGAGAAA	7	69356157	69356163				110	5400	5406
							111	AGGAAA	6	69356228	69356233				111	5471	5476
							112	GGAGAGGA	8	69356245	69356252				112	5488	5495
							113	GGAGAAA	7	69356256	69356262				113	5499	5505
							114	AGGAA	5	69356264	69356268				114	5507	5511
							115	AAGGA	5	69356337	69356341				115	5580	5584
							116	AGAAA	5	69356351	69356355				116	5594	5598
							117	AAAGGA	6	69356396	69356401				117	5639	5644
							118	AAAGA	5	69356421	69356425				118	5664	5668
							119	AAGAAA	6	69356514	69356519				119	5757	5762
							120	AAAGGA	6	69356561	69356566				120	5804	5809
							121	AAGGA	5	69356632	69356636				121	5875	5879
							122	AGGAAGGAGAA	11	69356703	69356713				122	5946	5956
							123	AGGAGA	6	69356767	69356772				123	6010	6015

Paired L1PA5 (B)

					r-TC in hg38					r-AG in hg38			r-TC in L1PA5(B)			r-AG in L1PA5(B)	
Subfamily	Chr:Positions	No.	r-TC Sequence	Length	Start Position	End Position	No.	r-AG Sequence	Length	Start Position	End Position	No.	Star Position	End position	No.	Star Position	End position
L1PA5	ChrX:85583162-85589299	1	TTTCT	5	85583240	85583244	1	AGGAA	5	85583187	85583191	1	79	83	1	26	30
		2	CCTTTCCT	8	85583388	85583395	2	AGAAGA	6	85583227	85583232	2	227	234	2	66	71
		3	TTCTCCT	7	85583803	85583809	3	AGGAGA	6	85583491	85583496	3	642	648	3	330	335
		4	CTTCTCT	7	85584343	85584349	4	AAGGAGG	7	85583682	85583688	4	1182	1188	4	521	527
		5	TCTTT	5	85584446	85584450	5	AGAAA	5	85583748	85583752	5	1285	1289	5	587	591
		6	CTCCTT	6	85584811	85584816	6	GAAGAGAG	8	85583789	85583796	6	1650	1655	6	628	635
		7	TCTCTC	6	85584946	85584951	7	AGAGGAG	7	85583956	85583962	7	1785	1790	7	795	801
		8	TTCTT	5	85584999	85585003	8	AGAAAGAAA	9	85584098	85584106	8	1838	1842	8	937	945
		9	TCTCT	5	85585507	85585511	9	AGAAAGGA	8	85584117	85584124	9	2346	2350	9	956	963
		10	TCCTT	5	85585657	85585661	10	AAAGA	5	85584163	85584167	10	2496	2500	10	1002	1006
		11	TTCTTCTC	8	85585851	85585858	11	AAAGA	5	85584188	85584192	11	2690	2697	11	1027	1031
		12	TCTCTC	6	85585952	85585957	12	AGAAA	5	85584209	85584213	12	2791	2796	12	1048	1052
		13	TTCTTT	6	85586091	85586096	13	AGAGAA	6	85584234	85584239	13	2930	2935	13	1073	1078
		14	TCTCT	5	85586132	85586136	14	AAAGGAA	7	85584253	85584259	14	2971	2975	14	1092	1098
		15	TTCCTC	6	85586552	85586557	15	GAGAGAAGAAGG	12	85584316	85584327	15	3391	3396	15	1155	1166
		16	TCTCT	5	85586600	85586604	16	AAAGGAGGAAA	11	85584355	85584365	16	3439	3443	16	1194	1204
		17	TTCCTTCT	8	85586728	85586735	17	AAAGAAG	7	85584380	85584386	17	3567	3574	17	1219	1225
		18	TCCTTC	6	85587087	85587092	18	AAAGA	5	85584402	85584406	18	3926	3931	18	1241	1245
		19	TCTCCTT	7	85587407	85587413	19	GGAAGAAAGAG	11	85584532	85584542	19	4246	4252	19	1371	1381
		20	TTCCT	5	85587477	85587481	20	GAGAAGAGAAG	11	85584579	85584589	20	4316	4320	20	1418	1428
		21	CTTTCTTC	8	85587760	85587767	21	AGAGGAAA	8	85584593	85584600	21	4599	4606	21	1432	1439
		22	TCTTT	5	85588003	85588007	22	AAGAAA	6	85584630	85584635	22	4842	4846	22	1469	1474
		23	CCTTCTTT	8	85588109	85588116	23	GAAGAGA	7	85584649	85584655	23	4948	4955	23	1488	1494
		24	TTCCTC	6	85588735	85588740	24	AGGAGAA	7	85584738	85584744	24	5574	5579	24	1577	1583
		25	TCCTTT	6	85588981	85588986	25	AGGAAA	6	85584783	85584788	25	5820	5825	25	1622	1627
							26	AGAGAA	6	85584792	85584797				26	1631	1636
							27	GAGAAGAG	8	85584817	85584824				27	1656	1663
							28	GAAGGAAA	8	85584868	85584875				28	1707	1714
							29	AGAGAGAAAGG	11	85584893	85584903				29	1732	1742
							30	AGAAA	5	85584955	85584959				30	1794	1798
							31	AGAAGAGAG	9	85584971	85584979				31	1810	1818
							32	AAAGAAA	7	85585004	85585010				32	1843	1849
							33	AAAGGAGAAA	10	85585061	85585070				33	1900	1909
							34	GAGAGA	6	85585099	85585104				34	1938	1943
							35	GAAGGAAG	8	85585141	85585148				35	1980	1987
							36	GGAAAGGAAA	10	85585159	85585168				36	1998	2007
							37	AGAGA	5	85585206	85585210				37	2045	2049
							38	GAAGAAA	7	85585221	85585227				38	2060	2066
							39	AAAGA	5	85585335	85585339				39	2174	2178
							40	AAAGA	5	85585360	85585364				40	2199	2203
							41	AGGAGA	6	85585392	85585397				41	2231	2236
							42	AGAGA	5	85585412	85585416				42	2251	2255
							43	GGAGGAAGA	9	85585443	85585451				43	2282	2290
							44	AAAGA	5	85585535	85585539				44	2374	2378
							45	AAAGAGA	7	85585543	85585549				45	2382	2388
							46	AAAGAAAG	8	85585551	85585558				46	2390	2397
							47	AAAGGAA	7	85585574	85585580				47	2413	2419
							48	AAGAAGAG	8	85585591	85585598				48	2430	2437
							49	AGAGA	5	85585662	85585666				49	2501	2505
							50	AGAAAGAGG	9	85585670	85585678				50	2509	2517
							51	AGAAAG	6	85585750	85585755				51	2589	2594
							52	AAGAAGGA	8	85585758	85585765				52	2597	2604
							53	AGGAA	5	85585771	85585775				53	2610	2614
							54	AGAAA	5	85585934	85585938				54	2773	2777
							55	AAGAAA	6	85585992	85585997				55	2831	2836
							56	AGAAA	5	85586078	85586082				56	2917	2921
							57	AAAGA	5	85586084	85586088				57	2923	2927
							58	AAGAA	5	85586106	85586110				58	2945	2949
							59	AAAGA	5	85586112	85586116				59	2951	2955
							60	GAAAGAAAG	9	85586190	85586198				60	3029	3037
							61	AGGAAAGA	8	85586200	85586207				61	3039	3046
							62	AAAGAA	6	85586238	85586243				62	3077	3082
							63	AGAGAAG	7	85586246	85586252				63	3085	3091
							64	AAGAAA	6	85586287	85586292				64	3126	3131
							65	AAGGAGA	7	85586316	85586322				65	3155	3161
							66	AGAGA	5	85586324	85586328				66	3163	3167
							67	AAAGA	5	85586366	85586370				67	3205	3209
							68	AAAGAAGAAA	10	85586405	85586414				68	3244	3253
							69	AGAGAGAAGAA	11	85586415	85586425				69	3254	3264
							70	AGAAA	5	85586477	85586481				70	3316	3320
							71	AGAGAA	6	85586492	85586497				71	3331	3336
							72	AGAAA	5	85586527	85586531				72	3366	3370
							73	AGAAGAAA	8	85586536	85586543				73	3375	3382
							74	AGGAAGAAG	9	85586586	85586594				74	3425	3433
							75	AAGAAGGAG	9	85586710	85586718				75	3549	3557
							76	AAAGAAA	7	85586752	85586758				76	3591	3597
							77	AAGAAGGAA	9	85586759	85586767				77	3598	3606
							78	AGAGA	5	85586821	85586825				78	3660	3664
							79	AAAGAAGAGAA	11	85586833	85586843				79	3672	3682
							80	AAGGA	5	85586969	85586973				80	3808	3812
							81	AGAGA	5	85587232	85587236				81	4071	4075
							82	AGGAGAAAGAAA	12	85587298	85587309				82	4137	4148
							83	AGGAAA	6	85587326	85587331				83	4165	4170
							84	AGAGGAAG	8	85587332	85587339				84	4171	4178
							85	AGAGAGA	7	85587503	85587509				85	4342	4348
							86	AAAGAGAA	8	85587545	85587552				86	4384	4391
							87	AGGAA	5	85587563	85587567				87	4402	4406
							88	GAAGGA	6	85587587	85587592				88	4426	4431
							89	AAGGAGAA	8	85587600	85587607				89	4439	4446
							90	AAGGAAA	7	85587624	85587630				90	4463	4469
							91	GAAAGAGGA	9	85587632	85587640				91	4471	4479
							92	GGAAGAA	7	85587652	85587658				92	4491	4497
							93	AGGAAGAA	8	85587677	85587684				93	4516	4523
							94	AAAGAG	6	85587811	85587816				94	4650	4655
							95	AAAGAA	6	85587845	85587850				95	4684	4689
							96	AGAGA	5	85587936	85587940				96	4775	4779
							97	AGAAA	5	85587971	85587975				97	4810	4814
							98	GGAAAGGA	8	85588035	85588042				98	4874	4881
							99	AGAAAG	6	85588090	85588095				99	4929	4934
							100	AAAGA	5	85588150	85588154				100	4989	4993
							101	AGAAGAAA	8	85588188	85588195				101	5027	5034
							102	AAGGA	5	85588231	85588235				102	5070	5074
							103	AAAGAG	6	85588305	85588310				103	5144	5149
							104	AAAGGAA	7	85588324	85588330				104	5163	5169
							105	GGAGAAA	7	85588365	85588371				105	5204	5210
							106	AAGAAGAAA	9	85588441	85588449				106	5280	5288
							107	GAAGGA	6	85588476	85588481				107	5315	5320
							108	AAAGAAGA	8	85588501	85588508				108	5340	5347
							109	AGAGAAA	7	85588561	85588567				109	5400	5406
							110	AGAAAG	6	85588624	85588629				110	5463	5468
							111	AGGAAA	6	85588632	85588637				111	5471	5476
							112	GGAGAGGA	8	85588649	85588656				112	5488	5495
							113	GGAGAAA	7	85588660	85588666				113	5499	5505
							114	AAGAA	5	85588668	85588672				114	5507	5511
							115	AAGGA	5	85588741	85588745				115	5580	5584
							116	AGAAA	5	85588755	85588759				116	5594	5598
							117	AAAGGA	6	85588800	85588805				117	5639	5644
							118	AAAGA	5	85588825	85588829				118	5664	5668
							119	AAAGA	5	85588872	85588876				119	5711	5715
							120	AAGAAA	6	85588918	85588923				120	5757	5762
							121	AAGGA	5	85589036	85589040				121	5875	5879
							122	GGAGGAA	7	85589157	85589163				122	5996	6002
							123	AGGAGA	6	85589171	85589176				123	6010	6015

Paired L1PA8 (A)

					r-TC in hg38					r-AG in hg38			r-TC in L1PA8(A)			r-AG in L1PA8(A)	
Subfamily	Chr:Positions	No.	r-TC Sequence	Length	Start Position	End Position	No.	r-AG Sequence	Length	Start Position	End Position	No.	Star Position	End position	No.	Star Position	End position
L1PA8	Chr12:26310353-26316762	1	TTTCT	5	26310422	26310426	1	AGGAA	5	26310369	26310373	1	70	74	1	17	21
		2	CCTCTCT	7	26311084	26311090	2	GAGGAAG	7	26310595	26310601	2	732	738	2	243	249
		3	TTCCTCCTCTCTT	13	26311094	26311106	3	GGAAAGAGG	9	26310812	26310820	3	742	754	3	460	468
		4	TCTCT	5	26311116	26311120	4	AGGAAG	6	26311025	26311030	4	764	768	4	673	678
		5	TCTTCCT	7	26311169	26311175	5	AAAGAAAGG	9	26311122	26311130	5	817	823	5	770	778
		6	TTCCT	5	26311234	26311238	6	GAAGAGAG	8	26311252	26311259	6	882	886	6	900	907
		7	TCCTT	5	26311458	26311462	7	AAGAGA	6	26311347	26311352	7	1106	1110	7	995	1000
		8	CCTCTTCTCCTTC	13	26311730	26311742	8	AGGAGAG	7	26311386	26311392	8	1378	1390	8	1034	1040
		9	CTCCTT	6	26311755	26311760	9	AGAGGAAGGAA	11	26311437	26311447	9	1403	1408	9	1085	1095
		10	TCTCTCT	7	26312436	26312442	10	AGAAGAGG	8	26311542	26311549	10	2084	2090	10	1190	1197
		11	TCCTTTCC	8	26312566	26312573	11	AGAAGGAAA	9	26311562	26311570	11	2214	2221	11	1210	1218
		12	TCTCT	5	26312988	26312992	12	AGAAAG	6	26311581	26311586	12	2636	2640	12	1229	1234
		13	TTCTT	5	26313138	26313142	13	AAAGGA	6	26311608	26311613	13	2786	2790	13	1256	1261
		14	TTCTTCTC	8	26313332	26313339	14	AAAGA	5	26311652	26311656	14	2980	2987	14	1300	1304
		15	TCTCTC	6	26313429	26313434	15	GAGGAAA	7	26311683	26311689	15	3077	3082	15	1331	1337
		16	TTCTTT	6	26313568	26313573	16	AAAGGAA	7	26311854	26311860	16	3216	3221	16	1502	1508
		17	TCTCT	5	26313609	26313613	17	AAGGAAG	7	26311870	26311876	17	3257	3261	17	1518	1524
		18	TTCCT	5	26314047	26314051	18	AAGAA	5	26311879	26311883	18	3695	3699	18	1527	1531
		19	TTCCTTCT	8	26314226	26314233	19	AGAAAG	6	26311901	26311906	19	3874	3881	19	1549	1554
		20	TTCCTTT	7	26314708	26314714	20	AGAAGAA	7	26311931	26311937	20	4356	4362	20	1579	1585
		21	TCCTCTCTC	9	26314740	26314748	21	GGAAGAAAGGA	11	26312022	26312032	21	4388	4396	21	1670	1680
		22	TTTCT	5	26314777	26314781	22	AGAGA	5	26312037	26312041	22	4425	4429	22	1685	1689
		23	TCTCT	5	26314841	26314845	23	AGAGAAA	7	26312083	26312089	23	4489	4493	23	1731	1737
		24	TTCCT	5	26314955	26314959	24	AAAGAA	6	26312090	26312095	24	4603	4607	24	1738	1743

		25	CTTTCTTC	8	26315213	26315220	25	AAAGGAA	7	26312099	26312105	25	4861	4868	25	1747	1753
		26	TTTCCT	6	26315323	26315328	26	AAGAAA	6	26312120	26312125	26	4971	4976	26	1768	1773
		27	TCTTT	5	26315456	26315460	27	AAAGA	5	26312141	26312145	27	5104	5108	27	1789	1793
		28	TTTCCT	6	26315496	26315501	28	AGGAGA	6	26312228	26312233	28	5144	5149	28	1876	1881
		29	TTCCT	5	26316186	26316190	29	AGGAAA	6	26312273	26312278	29	5834	5838	29	1921	1926
		30	TCTTT	5	26316428	26316432	30	AGAGAA	6	26312282	26312287	30	6076	6080	30	1930	1935
							31	AAGAAGAA	8	26312307	26312314				31	1955	1962
							32	GAAGGAAA	8	26312358	26312365				32	2006	2013
							33	AAAGAGAAAGG	11	26312383	26312393				33	2031	2041
							34	AGAAAGG	7	26312405	26312411				34	2053	2059
							35	AGAAA	5	26312445	26312449				35	2093	2097
							36	AGAAGAGAG	9	26312461	26312469				36	2109	2117
							37	GAAAGAAA	8	26312493	26312500				37	2141	2148
							38	AAAGGAGAAA	10	26312551	26312560				38	2199	2208
							39	AAAGGAAG	8	26312632	26312639				39	2280	2287
							40	AAAGGAAA	8	26312652	26312659				40	2300	2307
							41	GAAGAAA	7	26312714	26312720				41	2362	2368
							42	AAAGAAA	7	26312827	26312833				42	2475	2481
							43	AAAGAG	6	26312846	26312851				43	2494	2499
							44	AGGAGA	6	26312878	26312883				44	2526	2531
							45	AAAGA	5	26312898	26312902				45	2546	2550
							46	AAAGGAA	7	26312921	26312927				46	2569	2575
							47	GGAGGAAGA	9	26312929	26312937				47	2577	2585
							48	AAAGA	5	26313016	26313020				48	2664	2668
							49	AAAGA	5	26313026	26313030				49	2674	2678
							50	AAAGAAGG	8	26313032	26313039				50	2680	2687
							51	AAGAAGAG	8	26313072	26313079				51	2720	2727
							52	AGAGA	5	26313143	26313147				52	2791	2795
							53	AAAGA	5	26313155	26313159				53	2803	2807
							54	AGAAA	5	26313231	26313235				54	2879	2883
							55	AAGGA	5	26313242	26313246				55	2890	2894
							56	AAAGAA	6	26313408	26313413				56	3056	3061
							57	AAGAAA	6	26313469	26313474				57	3117	3122
							58	AGAAA	5	26313555	26313559				58	3203	3207
							59	GAGGAAA	7	26313638	26313644				59	3286	3292
							60	AAAGAAGAAAG	11	26313665	26313675				60	3313	3323
							61	AGGAAAGA	8	26313677	26313684				61	3325	3332
							62	AAAGAA	6	26313715	26313720				62	3363	3368
							63	AGGAAAG	7	26313723	26313729				63	3371	3377
							64	AGAAGA	6	26313757	26313762				64	3405	3410
							65	AAGAAA	6	26313764	26313769				65	3412	3417
							66	GAAGGAGA	8	26313792	26313799				66	3440	3447
							67	AGAGA	5	26313801	26313805				67	3449	3453
							68	AAAGA	5	26313857	26313861				68	3505	3509
							69	AAAGAAGAAA	10	26313896	26313905				69	3544	3553
							70	AGAGAGAA	8	26313906	26313913				70	3554	3561
							71	AGAAA	5	26313968	26313972				71	3616	3620
							72	AGAGAA	6	26313987	26313992				72	3635	3640
							73	AGAAA	5	26314022	26314026				73	3670	3674
							74	AGAAGAAA	8	26314031	26314038				74	3679	3686
							75	AGAAA	5	26314081	26314085				75	3729	3733
							76	AAAGAGGAG	9	26314208	26314216				76	3856	3864
							77	AGAAA	5	26314252	26314256				77	3900	3904
							78	AGAGA	5	26314319	26314323				78	3967	3971
							79	AGAAA	5	26314560	26314564				79	4208	4212
							80	AAGGA	5	26314733	26314737				80	4381	4385
							81	AGGAAG	6	26314827	26314832				81	4475	4480
							82	AGAAA	5	26314873	26314877				82	4521	4525
							83	AGAGAG	6	26314981	26314986				83	4629	4634
							84	AAAGAGAA	8	26315023	26315030				84	4671	4678
							85	AGGAA	5	26315041	26315045				85	4689	4693
							86	GAAGGA	6	26315065	26315070				86	4713	4718
							87	AAGGAGAA	8	26315078	26315085				87	4726	4733
							88	AAGAAA	6	26315102	26315107				88	4750	4755
							89	AAGAGAGGA	9	26315110	26315118				89	4758	4766
							90	AGGAAGAA	8	26315130	26315137				90	4778	4785
							91	AGAAA	5	26315229	26315233				91	4877	4881
							92	AAAGAA	6	26315298	26315303				92	4946	4951
							93	AGAAA	5	26315424	26315428				93	5072	5076
							94	AAGAAA	6	26315479	26315484				94	5127	5132
							95	GGAAAGGA	8	26315488	26315495				95	5136	5143
							96	AGAAA	5	26315542	26315546				96	5190	5194
							97	AAAGA	5	26315602	26315606				97	5250	5254
							98	AGAAGAAA	8	26315640	26315647				98	5288	5295
							99	AAGAAA	6	26315666	26315671				99	5314	5319
							100	GGAAAGA	7	26315681	26315687				100	5329	5335
							101	AAAGAG	6	26315758	26315763				101	5406	5411
							102	GGAGAAA	7	26315818	26315824				102	5466	5472
							103	AAGGAA	6	26315873	26315878				103	5521	5526
							104	AAAGGA	6	26315928	26315933				104	5576	5581
							105	AAAGAGGA	8	26315953	26315960				105	5601	5608
							106	AGAGAAA	7	26316013	26316019				106	5661	5667
							107	AGGAAA	6	26316083	26316088				107	5731	5736
							108	GAAGAAA	7	26316111	26316117				108	5759	5765
							109	GAAGGA	6	26316191	26316196				109	5839	5844
							110	AAAGA	5	26316275	26316279				110	5923	5927
							111	AAAGA	5	26316322	26316326				111	5970	5974
							112	AAAGAAA	7	26316367	26316373				112	6015	6021
							113	AAAGGA	6	26316415	26316420				113	6063	6068
							114	AGGAA	5	26316483	26316487				114	6131	6135
							115	AGAAA	5	26316489	26316493				115	6137	6141
							116	AAGAA	5	26316538	26316542				116	6186	6190
							117	AAAGGAAGG	9	26316598	26316606				117	6246	6254
							118	AGGAGAAA	8	26316616	26316623				118	6264	6271

Paired L1PA8 (B)

					r-TC in hg38					r-AG in hg38			r-TC in L1PA8(B)			r-AG in L1PA8(B)	
Subfamily	Chr:Positions	No.	r-TC Sequence	Length	Start Position	End Position	No.	r-AG Sequence	Length	Start Position	End Position	No.	Star Position	End position	No.	Star Position	End position
L1PA8	Chr20:39969159-39975570	1	TTCTCTC	7	39969264	39969270	1	AAAGAA	6	39969213	39969218	1	106	112	1	55	60
		2	CTCCTTC	7	39969628	39969634	2	AGAGAGGAAAGAAGA	15	39969304	39969318	2	470	476	2	146	160
		3	TTTCT	5	39969715	39969719	3	AAGAGAGG	8	39969382	39969389	3	557	561	3	224	231
		4	TCTTTCCTTCT	11	39969999	39970009	4	AGAAGA	6	39969458	39969463	4	841	851	4	300	305
		5	TTCCT	5	39970177	39970181	5	AGGAA	5	39969889	39969893	5	1019	1023	5	731	735
		6	TCTTT	5	39970242	39970246	6	GAGGAA	6	39970019	39970024	6	1084	1088	6	861	866
		7	CCTTCTCCT	9	39970259	39970267	7	AGAGA	5	39970212	39970216	7	1101	1109	7	1054	1058
		8	CCTCTTT	7	39970525	39970531	8	AGAGGAAGGAG	11	39970221	39970231	8	1367	1373	8	1063	1073
		9	TTCTT	5	39971276	39971280	9	AGGAA	5	39970285	39970289	9	2118	2122	9	1127	1131
		10	TCTTCTCC	8	39971353	39971360	10	AGAAGAGG	8	39970345	39970352	10	2195	2202	10	1187	1194
		11	TCTCT	5	39971784	39971788	11	AGAAAG	6	39970380	39970385	11	2626	2630	11	1222	1227
		12	TTCTT	5	39971933	39971937	12	GAGAAAGAA	9	39970477	39970485	12	2775	2779	12	1319	1327
		13	TTTCT	5	39972087	39972091	13	AAGGA	5	39970562	39970566	13	2929	2933	13	1404	1408
		14	CTTTCTTCTC	10	39972125	39972134	14	AGAAGGAGG	9	39970604	39970612	14	2967	2976	14	1446	1454
		15	CTCCTT	6	39972187	39972192	15	AAAGAAG	7	39970674	39970680	15	3029	3034	15	1516	1522
		16	TCTCTC	6	39972228	39972233	16	AAAGGA	6	39970696	39970701	16	3070	3075	16	1538	1543
		17	TTCTTT	6	39972366	39972371	17	AGAGAGGAA	9	39970733	39970741	17	3208	3213	17	1575	1583
		18	TCTCT	5	39972407	39972411	18	AAGAA	5	39970777	39970781	18	3249	3253	18	1619	1623
		19	TCCTTC	6	39972613	39972618	19	GGAAGAAAGAG	11	39970812	39970822	19	3455	3460	19	1654	1664
		20	TTTCTTCT	8	39973020	39973027	20	AGAGAAA	7	39970873	39970879	20	3862	3869	20	1715	1721
		21	CCTCTCTC	8	39973533	39973540	21	AAAGGAA	7	39970889	39970895	21	4375	4382	21	1731	1737
		22	TCTCT	5	39973633	39973637	22	AAGAAG	6	39970910	39970915	22	4475	4479	22	1752	1757
		23	TTTCT	5	39973654	39973658	23	AAAGA	5	39970931	39970935	23	4496	4500	23	1773	1777
		24	CTCCTT	6	39973693	39973698	24	GAAAGAGA	8	39970963	39970970	24	4535	4540	24	1805	1812
		25	TCTTT	5	39974288	39974292	25	AGAAGAA	7	39971018	39971024	25	5130	5134	25	1860	1866
		26	CCTTCCTC	8	39974382	39974389	26	AGGAAA	6	39971062	39971067	26	5224	5231	26	1904	1909
		27	TTTCTC	6	39974754	39974759	27	AGAGAA	6	39971071	39971076	27	5596	5601	27	1913	1918
		28	TTCCTC	6	39974994	39974999	28	GAGAAGA	7	39971096	39971102	28	5836	5841	28	1938	1944
		29	TCTTTCT	7	39975072	39975078	29	AAGAAA	6	39971111	39971116	29	5914	5920	29	1953	1958
		30	TCTTT	5	39975240	39975244	30	GAAGGAAA	8	39971147	39971154	30	6082	6086	30	1989	1996
		31	TTCTTC	6	39975534	39975539	31	AGAGAGAAAGG	11	39971172	39971182	31	6376	6381	31	2014	2024
							32	AGAAA	5	39971234	39971238				32	2076	2080
							33	AAAGAAA	7	39971281	39971287				33	2123	2129
							34	AAAGGAAA	8	39971338	39971345				34	2180	2187
							35	AAGGAA	6	39971376	39971381				35	2218	2223
							36	GAAGGAAG	8	39971418	39971425				36	2260	2267
							37	GGAAAGGAAA	10	39971436	39971445				37	2278	2287
							38	AAAGA	5	39971483	39971487				38	2325	2329
							39	GAAGAAA	7	39971501	39971507				39	2343	2349
							40	AAAGA	5	39971615	39971619				40	2457	2461
							41	AAAGAG	6	39971640	39971645				41	2482	2487
							42	AGGAGA	6	39971672	39971677				42	2514	2519
							43	AAAGA	5	39971692	39971696				43	2534	2538
							44	AAAGAGA	7	39971715	39971721				44	2557	2563
							45	GAAGGAAA	8	39971723	39971730				45	2565	2572
							46	AAAGA	5	39971811	39971815				46	2653	2657
							47	AAAGA	5	39971821	39971825				47	2663	2667
							48	AAAGAAGAG	9	39971827	39971835				48	2669	2677
							49	AGGAGAA	7	39971907	39971913				49	2749	2755
							50	AGAGA	5	39971938	39971942				50	2780	2784
							51	AAAGAGA	7	39971948	39971954				51	2790	2796
							52	AAGAGAGAAA	10	39972021	39972030				52	2863	2872
							53	AAAGAA	6	39972203	39972208				53	3045	3050
							54	GAGAAA	6	39972267	39972272				54	3109	3114
							55	AAGGAGGAAA	10	39972348	39972357				55	3190	3199
							56	AAGAAG	6	39972360	39972365				56	3202	3207
							57	AAAGAGA	7	39972387	39972393				57	3229	3235
							58	AAGAAAG	7	39972467	39972473				58	3309	3315
							59	AAAGAG	6	39972513	39972518				59	3355	3360
							60	AGAGAAG	7	39972521	39972527				60	3363	3369
							61	AGAAGA	6	39972555	39972560				61	3397	3402
							62	AAGAAA	6	39972562	39972567				62	3404	3409
							63	GAAGGAGA	8	39972590	39972597				63	3432	3439
							64	AGAGA	5	39972599	39972603				64	3441	3445
							65	AAGAAA	6	39972696	39972701				65	3538	3543
							66	AGAGAGAAGAA	11	39972702	39972712				66	3544	3554
							67	AGAAA	5	39972764	39972768				67	3606	3610
							68	AGAGAA	6	39972783	39972788				68	3625	3630
							69	AGAAA	5	39972818	39972822				69	3660	3664
							70	AGAAGAAA	8	39972827	39972834				70	3669	3676
							71	AGGAAGAAG	9	39972876	39972884				71	3718	3726
							72	AAAGAAA	7	39972950	39972956				72	3792	3798
							73	AGAGAGA	7	39972968	39972974				73	3810	3816
							74	AAAGAGAAA	9	39973002	39973010				74	3844	3852
							75	AGAAA	5	39973045	39973049				75	3887	3891
							76	AAGAAGGAA	9	39973050	39973058				76	3892	3900
							77	AGAGA	5	39973112	39973116				77	3954	3958
							78	AGAAA	5	39973353	39973357				78	4195	4199
							79	AAGGA	5	39973525	39973529				79	4367	4371
							80	AAGAGAAA	8	39973591	39973598				80	4433	4440
							81	AGGAAGAGAGGAAG	14	39973611	39973624				81	4453	4466
							82	AGAAA	5	39973665	39973669				82	4507	4511
							83	AAGGA	5	39973757	39973761				83	4599	4603
							84	AGAGAG	6	39973788	39973793				84	4630	4635
							85	AAAGAGAA	8	39973830	39973837				85	4672	4679
							86	AGGAA	5	39973848	39973852				86	4690	4694

							87	AAGAGA	6	39973863	39973868				87	4705	4710
							88	GAAGGA	6	39973872	39973877				88	4714	4719
							89	AAGGAGAG	8	39973885	39973892				89	4727	4734
							90	AAGAAA	6	39973909	39973914				90	4751	4756
							91	AAGAGAGGA	9	39973917	39973925				91	4759	4767
							92	AGGAAGAAG	9	39973962	39973970				92	4804	4812
							93	AAAGAG	6	39974096	39974101				93	4938	4943
							94	AAAGAA	6	39974130	39974135				94	4972	4977
							95	AGAGA	5	39974247	39974251				95	5089	5093
							96	AGAAA	5	39974256	39974260				96	5098	5102
							97	AGAAA	5	39974363	39974367				97	5205	5209
							98	AGAGA	5	39974423	39974427				98	5265	5269
							99	AGAAGAAA	8	39974461	39974468				99	5303	5310
							100	AAAGA	5	39974504	39974508				100	5346	5350
							101	AAAGAG	6	39974578	39974583				101	5420	5425
							102	AAAGAAA	7	39974597	39974603				102	5439	5445
							103	GGAGAAA	7	39974638	39974644				103	5480	5486
							104	GAGAAA	6	39974702	39974707				104	5544	5549
							105	AAAGGA	6	39974736	39974741				105	5578	5583
							106	AAAGAAGA	8	39974761	39974768				106	5603	5610
							107	AGAGAAA	7	39974820	39974826				107	5662	5668
							108	AAGAAA	6	39974891	39974896				108	5733	5738
							109	GGAGAAA	7	39974919	39974925				109	5761	5767
							110	AGGAA	5	39974927	39974931				110	5769	5773
							111	AAGGA	5	39975000	39975004				111	5842	5846
							112	AGAAA	5	39975014	39975018				112	5856	5860
							113	AAAGGAA	7	39975059	39975065				113	5901	5907
							114	AAAGA	5	39975084	39975088				114	5926	5930
							115	AAAGA	5	39975131	39975135				115	5973	5977
							116	AAAGAAA	7	39975176	39975182				116	6018	6024
							117	AAAGAA	6	39975224	39975229				117	6066	6071
							118	AGAAA	5	39975295	39975299				118	6137	6141

Paired L1PA15 (A)

					r-TC in hg38					r-AG in hg38			r-TC in L1PA15(A)			r-AG in L1PA15(A)	
Subfamily	Chr:Positions	No.	r-TC Sequence	Length	Start Position	End Position	No.	r-AG Sequence	Length	Start Position	End Position	No.	Star Position	End position	No.	Star Position	End position
L1PA15	Chr5:12131805-12138332	1	TCTCCT	6	12131831	12131836	1	AGGAAG	6	12131823	12131828	1	27	32	1	19	24
		2	TCTTCT	6	12132420	12132425	2	AAAGAG	6	12131846	12131851	2	616	621	2	42	47
		3	CTTTCT	6	12132698	12132703	3	GAGAAA	6	12131884	12131889	3	894	899	3	80	85
		4	TCCTTCTTCT	10	12132714	12132723	4	AGAAA	5	12131936	12131940	4	910	919	4	132	136
		5	TTCCT	5	12132799	12132803	5	AGGAGAA	7	12131981	12131987	5	995	999	5	177	183
		6	CTTCTT	6	12132933	12132938	6	GAGAAA	6	12132076	12132081	6	1129	1134	6	272	277
		7	CTCTTCT	7	12133264	12133270	7	AGGAAGA	7	12132106	12132112	7	1460	1466	7	302	308
		8	TTTCTT	6	12133336	12133341	8	AGAGAGGA	8	12132263	12132270	8	1532	1537	8	459	466
		9	CTTCCTT	7	12133791	12133797	9	GAGAAA	6	12132426	12132431	9	1987	1993	9	622	627
		10	TTCTT	5	12134046	12134050	10	AAGGAA	6	12132545	12132550	10	2242	2246	10	741	746
		11	TCCTTT	6	12134123	12134128	11	AGGAAG	6	12132621	12132626	11	2319	2324	11	817	822
		12	TCTTT	5	12134292	12134296	12	AAAGGAG	7	12132677	12132683	12	2488	2492	12	873	879
		13	TTCTT	5	12134696	12134700	13	GAGGAA	6	12132737	12132742	13	2892	2896	13	933	938
		14	TTCTTCTC	8	12134887	12134894	14	AAGGA	5	12132939	12132943	14	3083	3090	14	1135	1139
		15	TCTCT	5	12135165	12135169	15	AAAGAAA	7	12133007	12133013	15	3361	3365	15	1203	1209
		16	TTCCT	5	12135589	12135593	16	GGAGAGAA	8	12133023	12133030	16	3785	3789	16	1219	1226
		17	CTTCTCTTC	9	12135800	12135808	17	AGGAGA	6	12133058	12133063	17	3996	4004	17	1254	1259
		18	TTCCT	5	12135998	12136002	18	AAAGAAGAAA	10	12133128	12133137	18	4194	4198	18	1324	1333
		19	TCCTTTC	7	12136128	12136134	19	AAAGAAA	7	12133167	12133173	19	4324	4330	19	1363	1369
		20	CCTCTCTCTCC	11	12136281	12136291	20	AAGAAA	6	12133174	12133179	20	4477	4487	20	1370	1375
		21	TTCCT	5	12136524	12136528	21	AAAGAAA	7	12133180	12133186	21	4720	4724	21	1376	1382
		22	TTTCT	5	12136585	12136589	22	AGAAA	5	12133203	12133207	22	4781	4785	22	1399	1403
		23	TTCCT	5	12136786	12136790	23	AAAGGA	6	12133219	12133224	23	4982	4986	23	1415	1420
		24	TTCTTC	6	12136809	12136814	24	AAGAAAGAA	9	12133243	12133251	24	5005	5010	24	1439	1447
		25	TCTTT	5	12137456	12137460	25	AGAAGAA	7	12133257	12133263	25	5652	5656	25	1453	1459
		26	TTCCTT	6	12137769	12137774	26	AGAAA	5	12133359	12133363	26	5965	5970	26	1555	1559
		27	TCCTCTC	7	12138013	12138019	27	AAGGAAG	7	12133429	12133435	27	6209	6215	27	1625	1631
		28	TCCTT	5	12138252	12138256	28	AAAGA	5	12133489	12133493	28	6448	6452	28	1685	1689
		29	TTCTCTT	7	12138309	12138315	29	AAGAA	5	12133528	12133532	29	6505	6511	29	1724	1728
							30	GAGAAA	6	12133579	12133584				30	1775	1780
							31	AAAGAA	6	12133655	12133660				31	1851	1856
							32	AAAGAGA	7	12133695	12133701				32	1891	1897
							33	GGAGAAAG	8	12133740	12133747				33	1936	1943
							34	AGAAA	5	12134003	12134007				34	2199	2203
							35	AAGAGA	6	12134021	12134026				35	2217	2222
							36	AAAGAAA	7	12134051	12134057				36	2247	2253
							37	GAAGGAGAAA	10	12134107	12134116				37	2303	2312
							38	GAAAGGAG	8	12134187	12134194				38	2383	2390
							39	GGAAAGAAA	9	12134205	12134213				39	2401	2409
							40	AAAGAA	6	12134411	12134416				40	2607	2612
							41	AAAGA	5	12134443	12134447				41	2639	2643
							42	GGAGAAA	7	12134493	12134499				42	2689	2695
							43	GAAAGA	6	12134516	12134521				43	2712	2717
							44	AAAGA	5	12134574	12134578				44	2770	2774
							45	AAAGAGA	7	12134582	12134588				45	2778	2784
							46	AAGGA	5	12134594	12134598				46	2790	2794
							47	AAAGA	5	12134614	12134618				47	2810	2814
							48	AAGAA	5	12134630	12134634				48	2826	2830
							49	AGAGGAG	7	12134668	12134674				49	2864	2870
							50	AAAGAGA	7	12134711	12134717				50	2907	2913
							51	AGAAA	5	12134789	12134793				51	2985	2989
							52	AGAAA	5	12135012	12135016				52	3208	3212
							53	AGAAA	5	12135026	12135030				53	3222	3226
							54	AGAAA	5	12135111	12135115				54	3307	3311
							55	AAGAAA	6	12135118	12135123				55	3314	3319
							56	AAGAGAGAAA	10	12135192	12135201				56	3388	3397
							57	AGAAAGG	7	12135228	12135234				57	3424	3430
							58	AGAAGAA	7	12135264	12135270				58	3460	3466
							59	AGAGAAGGAAG	11	12135273	12135283				59	3469	3479
							60	AGAAA	5	12135285	12135289				60	3481	3485
							61	AGAAGA	6	12135307	12135312				61	3503	3508
							62	GAGAAA	6	12135314	12135319				62	3510	3515
							63	GAAGGAAA	8	12135342	12135349				63	3538	3545
							64	AAGAAA	6	12135357	12135362				64	3553	3558
							65	AAAGA	5	12135372	12135376				65	3568	3572
							66	AAAGAGAA	8	12135443	12135450				66	3639	3646
							67	AGAAA	5	12135505	12135509				67	3701	3705
							68	AAAGAAA	7	12135515	12135521				68	3711	3717
							69	AGAGA	5	12135529	12135533				69	3725	3729
							70	AGAAA	5	12135564	12135568				70	3760	3764
							71	GAAAGAGGAAGAAA	14	12135617	12135630				71	3813	3826
							72	AAAGAAA	7	12135747	12135753				72	3943	3949
							73	AGAGA	5	12135854	12135858				73	4050	4054
							74	AAGAAA	6	12135862	12135867				74	4058	4063
							75	AAAGAAA	7	12135869	12135875				75	4065	4071
							76	AAAGAAA	7	12135922	12135928				76	4118	4124
							77	AGAAA	5	12136101	12136105				77	4297	4301
							78	GAAGGAAA	8	12136165	12136172				78	4361	4368
							79	AGAAA	5	12136242	12136246				79	4438	4442
							80	AAGAGAAAGAGAAAGAA	17	12136339	12136355				80	4535	4551
							81	AGGAAGAG	8	12136373	12136380				81	4569	4576
							82	AGGAAA	6	12136427	12136432				82	4623	4628
							83	AGGAAAG	7	12136479	12136485				83	4675	4681
							84	AGGAA	5	12136563	12136567				84	4759	4763
							85	AGAAA	5	12136610	12136614				85	4806	4810
							86	GAAGGAG	7	12136634	12136640				86	4830	4836
							87	AAGAA	5	12136650	12136654				87	4846	4850
							88	GAAGAAA	7	12136671	12136677				88	4867	4873
							89	AGGAA	5	12136680	12136684				89	4876	4880
							90	AAAGGAA	7	12136761	12136767				90	4957	4963
							91	AGAAA	5	12136823	12136827				91	5019	5023
							92	AAAGAA	6	12136891	12136896				92	5087	5092
							93	AGAGAG	6	12137007	12137012				93	5203	5208
							94	AGAAA	5	12137016	12137020				94	5212	5216
							95	GGAGAAAGGA	10	12137078	12137087				95	5274	5283
							96	AGAAGA	6	12137134	12137139				96	5330	5335
							97	AAAGA	5	12137195	12137199				97	5391	5395
							98	AAAGA	5	12137272	12137276				98	5468	5472
							99	AAAGAG	6	12137346	12137351				99	5542	5547
							100	AAAGAAA	7	12137365	12137371				100	5561	5567
							101	GGAAGAAA	8	12137405	12137412				101	5601	5608
							102	AAGAAA	6	12137461	12137466				102	5657	5662
							103	AGAAAG	6	12137488	12137493				103	5684	5689
							104	AAAGGAAGA	9	12137542	12137550				104	5738	5746
							105	AGAGAAA	7	12137603	12137609				105	5799	5805
							106	GAGAAA	6	12137695	12137700				106	5891	5896
							107	AAGAAA	6	12137701	12137706				107	5897	5902
							108	AAAGGAA	7	12137834	12137840				108	6030	6036
							109	AAAGA	5	12137905	12137909				109	6101	6105
							110	AAAGAAA	7	12137950	12137956				110	6146	6152
							111	AGAAA	5	12138073	12138077				111	6269	6273
							112	AGGAGAGGA	9	12138191	12138199				112	6387	6395
							113	AGAAA	5	12138202	12138206				113	6398	6402

Paired L1PA15 (B)

					r-TC in hg38					r-AG in hg38			r-TC in L1PA15(B)			r-AG in L1PA15(B)	
Subfamily	Chr:Positions	No.	r-TC Sequence	Length	Start Position	End Position	No.	r-AG Sequence	Length	Start Position	End Position	No.	Star Position	End position	No.	Star Position	End position
L1PA15	Chr8:124128949-124135471	1	TTCTTC	6	124129149	124129154	1	AGAGAG	6	124129033	124129038	1	201	206	1	85	90
		2	TTTCCT	6	124129955	124129960	2	AGAGAGGAGG	10	124129040	124129049	2	1007	1012	2	92	101
		3	TCTTCTT	7	124130455	124130461	3	GGAAGAA	7	124129089	124129095	3	1507	1513	3	141	147
		4	TTCTT	5	124130494	124130498	4	AGGAGAA	7	124129181	124129187	4	1546	1550	4	233	239
		5	TCCTT	5	124131336	124131340	5	AGAGAG	6	124129239	124129244	5	2388	2392	5	291	296
		6	TTCTT	5	124131857	124131861	6	AGGAGAAA	8	124129262	124129269	6	2909	2913	6	314	321
		7	CTCTTT	6	124132018	124132023	7	AGAGA	5	124129509	124129513	7	3070	3075	7	561	565
		8	TTCTT	5	124132047	124132051	8	AGGAAG	6	124129576	124129581	8	3099	3103	8	628	633
		9	TTCTTT	6	124132284	124132289	9	GAGAGGAA	8	124129961	124129968	9	3336	3341	9	1013	1020
		10	TCTCT	5	124132325	124132329	10	AGAAA	5	124129986	124129990	10	3377	3381	10	1038	1042
		11	TTTCT	5	124132562	124132566	11	AAAGAAA	7	124130231	124130237	11	3614	3618	11	1283	1289
		12	TTTCT	5	124132928	124132932	12	AAAGAA	6	124130340	124130345	12	3980	3984	12	1392	1397
		13	CTCCTTCCT	9	124132964	124132972	13	AAAGGA	6	124130364	124130369	13	4016	4024	13	1416	1421
		14	TCTCT	5	124133537	124133541	14	AAAGA	5	124130380	124130384	14	4589	4593	14	1432	1436
		15	TTCTT	5	124133559	124133563	15	AAGAAAGAA	9	124130408	124130416	15	4611	4615	15	1460	1468
		16	TTTCT	5	124133582	124133586	16	AAGAA	5	124130424	124130428	16	4634	4638	16	1476	1480
		17	TTCCT	5	124133924	124133928	17	AGAAA	5	124130525	124130529	17	4976	4980	17	1577	1581
		18	TTCTTT	6	124133947	124133952	18	GAAGAAGA	8	124130532	124130539	18	4999	5004	18	1584	1591
		19	TTCTCT	6	124134225	124134230	19	AGGAAGGAAGA	11	124130545	124130555	19	5277	5282	19	1597	1607
		20	CTCCTTCCTT	10	124134288	124134297	20	AGGAGAAAG	9	124130565	124130573	20	5340	5349	20	1617	1625
		21	TTTCT	5	124134476	124134480	21	AAGGAA	6	124130588	124130593	21	5528	5532	21	1640	1645
		22	TTTCCTTT	8	124134490	124134497	22	AAGGAA	6	124130597	124130602	22	5542	5549	22	1649	1654
		23	TTTCTC	6	124134909	124134914	23	AGGAGA	6	124130619	124130624	23	5961	5966	23	1671	1676
							24	AAAGA	5	124130627	124130631				24	1679	1683
							25	AAGAAAGAA	9	124130647	124130655				25	1699	1707
							26	AAGAA	5	124130690	124130694				26	1742	1746
							27	GAAGAAAGAA	10	124130740	124130749				27	1792	1801
							28	AAAGA	5	124130761	124130765				28	1813	1817
							29	AAAGAA	6	124130814	124130819				29	1866	1871
							30	AAAGAGA	7	124130853	124130859				30	1905	1911
							31	AAAGAGG	7	124130890	124130896				31	1942	1948

							32	GAGAAAG	7	124130899	124130905				32	1951	1957
							33	AGAGAGG	7	124130965	124130971				33	2017	2023
							34	AGGAAA	6	124130981	124130986				34	2033	2038
							35	AGAGAA	6	124130990	124130995				35	2042	2047
							36	GAAAGAAA	8	124131066	124131073				36	2118	2125
							37	AAAGGAAG	8	124131082	124131089				37	2134	2141
							38	AGAGAAA	7	124131092	124131098				38	2144	2150
							39	AGAGGAAA	8	124131116	124131123				39	2168	2175
							40	AGAAA	5	124131154	124131158				40	2206	2210
							41	AGAAA	5	124131205	124131209				41	2257	2261
							42	AGAAA	5	124131210	124131214				42	2262	2266
							43	AAGAAA	6	124131256	124131261				43	2308	2313
							44	AGGAGAAA	8	124131262	124131269				44	2314	2321
							45	AAGAGAA	7	124131298	124131304				45	2350	2356
							46	AAAGA	5	124131365	124131369				46	2417	2421
							47	AAGAAA	6	124131385	124131390				47	2437	2442
							48	AAAGAAG	7	124131568	124131574				48	2620	2626
							49	AAGAGA	6	124131600	124131605				49	2652	2657
							50	GGAGAAA	7	124131651	124131657				50	2703	2709
							51	AGAAA	5	124131674	124131678				51	2726	2730
							52	AGGAAA	6	124131681	124131686				52	2733	2738
							53	AAAGA	5	124131749	124131753				53	2801	2805
							54	AAAGA	5	124131755	124131759				54	2807	2811
							55	AGAGA	5	124131862	124131866				55	2914	2918
							56	GAAGAAA	7	124131872	124131878				56	2924	2930
							57	GAGAGA	6	124131903	124131908				57	2955	2960
							58	AAAGA	5	124131957	124131961				58	3009	3013
							59	AGAAA	5	124132111	124132115				59	3163	3167
							60	AGAAA	5	124132172	124132176				60	3224	3228
							61	AAGAAA	6	124132185	124132190				61	3237	3242
							62	AGAAA	5	124132271	124132275				62	3323	3327
							63	AAGAAA	6	124132278	124132283				63	3330	3335
							64	AAAGA	5	124132305	124132309				64	3357	3361
							65	AAGAAGGA	8	124132352	124132359				65	3404	3411
							66	AGAAAGA	7	124132394	124132400				66	3446	3452
							67	AAAGGAA	7	124132429	124132435				67	3481	3487
							68	AGAAA	5	124132438	124132442				68	3490	3494
							69	GAGAAGA	7	124132471	124132477				69	3523	3529
							70	AGAAA	5	124132509	124132513				70	3561	3565
							71	GGAAAGAA	8	124132567	124132574				71	3619	3626
							72	AAAGAAAG	8	124132609	124132616				72	3661	3668
							73	AAAGA	5	124132618	124132622				73	3670	3674
							74	AGAAA	5	124132640	124132644				74	3692	3696
							75	AAAGGAGA	8	124132649	124132656				75	3701	3708
							76	AGAAA	5	124132729	124132733				76	3781	3785
							77	AGAAA	5	124132738	124132742				77	3790	3794
							78	AGAAA	5	124132759	124132763				78	3811	3815
							79	AGGAAGAAA	9	124132788	124132796				79	3840	3848
							80	AGAAA	5	124132859	124132863				80	3911	3915
							81	AGAAGGA	7	124132876	124132882				81	3928	3934
							82	AGAAA	5	124133018	124133022				82	4070	4074
							83	AAAGAAA	7	124133033	124133039				83	4085	4091
							84	AGGAAA	6	124133257	124133262				84	4309	4314
							85	GAAGGAA	7	124133317	124133323				85	4369	4375
							86	AAGAGGAAGAAA	12	124133487	124133498				86	4539	4550
							87	AGGAA	5	124133515	124133519				87	4567	4571
							88	AGAGGAAG	8	124133521	124133528				88	4573	4580
							89	AAGAA	5	124133700	124133704				89	4752	4756
							90	AAAGAA	6	124133731	124133736				90	4783	4788
							91	GAAAGA	6	124133772	124133777				91	4824	4829
							92	AAGAA	5	124133788	124133792				92	4840	4844
							93	AAAGAAA	7	124133809	124133815				93	4861	4867
							94	AGGAAGAAG	9	124133862	124133870				94	4914	4922
							95	AAGAA	5	124133880	124133884				95	4932	4936
							96	AGAAA	5	124133961	124133965				96	5013	5017
							97	AAAGAG	6	124133995	124134000				97	5047	5052
							98	AAAGAA	6	124134027	124134032				98	5079	5084
							99	AGAGA	5	124134146	124134150				99	5198	5202
							100	AGAAA	5	124134153	124134157				100	5205	5209
							101	AAAGA	5	124134220	124134224				101	5272	5276
							102	AAAGA	5	124134316	124134320				102	5368	5372
							103	AAAGA	5	124134397	124134401				103	5449	5453
							104	AAAGA	5	124134411	124134415				104	5463	5467
							105	AAGGAG	6	124134470	124134475				105	5522	5527
							106	GAAAGGAAA	9	124134507	124134515				106	5559	5567
							107	AAAGAGA	7	124134529	124134535				107	5581	5587
							108	AGAGAAA	7	124134550	124134556				108	5602	5608
							109	AAGAGAAA	8	124134622	124134629				109	5674	5681
							110	AGAAA	5	124134662	124134666				110	5714	5718
							111	AAAGAAGA	8	124134674	124134681				111	5726	5733
							112	AGAAA	5	124134738	124134742				112	5790	5794
							113	AAAGAAA	7	124134835	124134841				113	5887	5893
							114	AAAGAA	6	124134915	124134920				114	5967	5972
							115	AAAGGAA	7	124134974	124134980				115	6026	6032
							116	AAAGAAA	7	124135091	124135097				116	6143	6149
							117	AGGAA	5	124135209	124135213				117	6261	6265
							118	AGAAA	5	124135215	124135219				118	6267	6271
							119	AAGAAGG	7	124135281	124135287				119	6333	6339
							120	GGAGAGAA	8	124135326	124135333				120	6378	6385

Paired L1Md_T (A)

					r-TC in mm10					r-AG in mm10			r-TC in L1Md_T(A)			r-AG in L1MD_T(A)	
Subfamily	Chr:Positions	No.	r-TC Sequence	Length	Start Position	End Position	No.	r-AG Sequence	Length	Start Position	End Position	No.	Star Position	End position	No.	Star Position	End position
L1Md_T	ChrX:21527595-21533712	1	CTCTCT	6	21527646	21527651	1	AGAGGA	6	21527640	21527645	1	52	57	1	46	51
		2	CCTTTCT	7	21527745	21527751	2	AGGAA	5	21527725	21527729	2	151	157	2	131	135
		3	TTCCTTT	7	21527767	21527773	3	AAGGA	5	21527731	21527735	3	173	179	3	137	141
		4	TTCCTCC	7	21528805	21528811	4	AGAGAG	6	21527810	21527815	4	1211	1217	4	216	221
		5	TCTTTCC	7	21529087	21529093	5	AAAGGAGA	8	21527829	21527836	5	1493	1499	5	235	242
		6	TCCTTT	6	21529178	21529183	6	AGAGGAAG	8	21527930	21527937	6	1584	1589	6	336	343
		7	CTTTCCTTT	9	21529259	21529267	7	AAAGGA	6	21527996	21528001	7	1665	1673	7	402	407
		8	TCTCT	5	21529272	21529276	8	AGAGAAA	7	21528006	21528012	8	1678	1682	8	412	418
		9	CCTTCTTCTC	10	21529811	21529820	9	AGGAAGGA	8	21528050	21528057	9	2217	2226	9	456	463
		10	TCTTCTCC	8	21529835	21529842	10	AGAAAG	6	21528085	21528090	10	2241	2248	10	491	496
		11	CTCTCCTC	8	21530000	21530007	11	AAGAA	5	21528125	21528129	11	2406	2413	11	531	535
		12	TTCCT	5	21530116	21530120	12	AGAAA	5	21528140	21528144	12	2522	2526	12	546	550
		13	TTCTTT	6	21530312	21530317	13	AAGAGAAA	8	21528211	21528218	13	2718	2723	13	617	624
		14	TCCTTCT	7	21530450	21530456	14	AAGAAGGAAA	10	21528269	21528278	14	2856	2862	14	675	684
		15	TTTCT	5	21530507	21530511	15	AGGAAAG	7	21528282	21528288	15	2913	2917	15	688	694
		16	TTCTTCTC	8	21530685	21530692	16	AAAGAG	6	21528314	21528319	16	3091	3098	16	720	725
		17	TTTCCTT	7	21530814	21530820	17	AAAGA	5	21528321	21528325	17	3220	3226	17	727	731
		18	TTTCTCC	7	21531201	21531207	18	AGAGAG	6	21528338	21528343	18	3607	3613	18	744	749
		19	TTCCT	5	21531233	21531237	19	AGGAAA	6	21528348	21528353	19	3639	3643	19	754	759
		20	CCTTTCTCT	9	21531432	21531440	20	AGAAA	5	21528412	21528416	20	3838	3846	20	818	822
		21	TTTCTCT	7	21531569	21531575	21	AAAGAAA	7	21528421	21528427	21	3975	3981	21	827	833
		22	TTCTTC	6	21531718	21531723	22	GGAGAGAGAAA	11	21528448	21528458	22	4124	4129	22	854	864
		23	CTCTTCTC	8	21531788	21531795	23	AGGAAAGAAA	10	21528463	21528472	23	4194	4201	23	869	878
		24	TCTCCTT	7	21532072	21532078	24	AGGAA	5	21528475	21528479	24	4478	4484	24	881	885
		25	TTCCT	5	21532192	21532196	25	AGGAGA	6	21528510	21528515	25	4598	4602	25	916	921
		26	TTTCT	5	21532266	21532270	26	AGAGAAA	7	21528520	21528526	26	4672	4676	26	926	932
		27	TCTTT	5	21532328	21532332	27	AAAGAAA	7	21528569	21528575	27	4734	4738	27	975	981
		28	TCCTT	5	21532504	21532508	28	AAAGA	5	21528589	21528593	28	4910	4914	28	995	999
		29	TTCCTC	6	21532690	21532695	29	AGGAAA	6	21528612	21528617	29	5096	5101	29	1018	1023
		30	CCTCTCCT	8	21532735	21532742	30	GAGAAGA	7	21528631	21528637	30	5141	5148	30	1037	1043
		31	TTCCT	5	21532936	21532940	31	AGGAAA	6	21528691	21528696	31	5342	5346	31	1097	1102
		32	CCTCCTT	7	21533129	21533135	32	AGAAGAAA	8	21528715	21528722	32	5535	5541	32	1121	1128
		33	TCTCTT	6	21533314	21533319	33	AAAGAAAGAGA	11	21528736	21528746	33	5720	5725	33	1142	1152
							34	AAGAAG	6	21528762	21528767				34	1168	1173
							35	AGAAA	5	21528795	21528799				35	1201	1205
							36	AGAAA	5	21528800	21528804				36	1206	1210
							37	AAAGA	5	21528848	21528852				37	1254	1258
							38	AGAGA	5	21528934	21528938				38	1340	1344
							39	AAGAAAG	7	21528941	21528947				39	1347	1353
							40	AGAAGA	6	21528950	21528955				40	1356	1361
							41	AAGAGAA	7	21528982	21528988				41	1388	1394
							42	GGAGAAA	7	21529043	21529049				42	1449	1455
							43	AAAGGA	6	21529110	21529115				43	1516	1521
							44	AAGAAAG	7	21529168	21529174				44	1574	1580
							45	AAAGAAA	7	21529194	21529200				45	1600	1606
							46	AAGAA	5	21529209	21529213				46	1615	1619
							47	AAAGGAAG	8	21529242	21529249				47	1648	1655
							48	AAAGA	5	21529306	21529310				48	1712	1716
							49	AGAAA	5	21529363	21529367				49	1769	1773
							50	AAAGAGA	7	21529383	21529389				50	1789	1795
							51	AGAAA	5	21529412	21529416				51	1818	1822
							52	GAAGAAA	7	21529440	21529446				52	1846	1852
							53	AAAGA	5	21529507	21529511				53	1913	1917
							54	AAGGAGG	7	21529513	21529519				54	1919	1925
							55	AAAGA	5	21529612	21529616				55	2018	2022
							56	AGAAA	5	21529712	21529716				56	2118	2122
							57	AGAGA	5	21529723	21529727				57	2129	2133
							58	AAAGGA	6	21529801	21529806				58	2207	2212
							59	AAGGAAGA	8	21530023	21530030				59	2429	2436
							60	GAAGAAAGAAA	11	21530034	21530044				60	2440	2450
							61	AAGAGGAAA	9	21530121	21530129				61	2527	2535
							62	AAAGGAAA	8	21530153	21530160				62	2559	2566
							63	AGAGAGAG	8	21530163	21530170				63	2569	2576
							64	AAAGGAAG	8	21530214	21530221				64	2620	2627
							65	AGGAAA	6	21530249	21530254				65	2655	2660
							66	AAAGAA	6	21530286	21530291				66	2692	2697
							67	GAGAAA	6	21530318	21530323				67	2724	2729
							68	AGAAA	5	21530396	21530400				68	2802	2806
							69	AAAGGAAG	8	21530404	21530411				69	2810	2817
							70	GAGAAA	6	21530426	21530431				70	2832	2837
							71	AAAGAG	6	21530617	21530622				71	3023	3028
							72	AGGAA	5	21530626	21530630				72	3032	3036
							73	AAAGAAGA	8	21530666	21530673				73	3072	3079
							74	AGAAA	5	21530710	21530714				74	3116	3120
							75	AAAGAG	6	21530779	21530784				75	3185	3190
							76	AAAGA	5	21530790	21530794				76	3196	3200
							77	AAAGAA	6	21530796	21530801				77	3202	3207
							78	AAGAA	5	21530870	21530874				78	3276	3280
							79	AGAGAAGG	8	21530927	21530934				79	3333	3340
							80	GAGAAAG	7	21531019	21531025				80	3425	3431
							81	GGAAAGA	7	21531065	21531071				81	3471	3477
							82	GAGGAA	6	21531073	21531078				82	3479	3484
							83	GGAAAGAAG	9	21531147	21531155				83	3553	3561
							84	AAGGA	5	21531189	21531193				84	3595	3599
							85	AAGGAGG	7	21531260	21531266				85	3666	3672
							86	GGAAAGGAAGAAA	13	21531284	21531296				86	3690	3702
							87	AGAGAA	6	21531359	21531364				87	3765	3770
							88	AAAGGA	6	21531445	21531450				88	3851	3856
							89	GAGAAAGAAA	10	21531461	21531470				89	3867	3876
							90	AAGGAAA	7	21531473	21531479				90	3879	3885

							91	AAGGAAG	7	21531536	21531542				91	3942	3948
							92	GAAAGA	6	21531544	21531549				92	3950	3955
							93	AAGAA	5	21531560	21531564				93	3966	3970
							94	GAAGAAAGAAA	11	21531576	21531586				94	3982	3992
							95	AAAGAAGA	8	21531589	21531596				95	3995	4002
							96	AGAAGA	6	21531601	21531606				96	4007	4012
							97	GGAAAGA	7	21531608	21531614				97	4014	4020
							98	AGAAAGG	7	21531732	21531738				98	4138	4144
							99	AGGAGAG	7	21531775	21531781				99	4181	4187
							100	AAGGA	5	21531796	21531800				100	4202	4206
							101	AAAGAA	6	21531803	21531808				101	4209	4214
							102	AGAAA	5	21531886	21531890				102	4292	4296
							103	AGAAA	5	21531927	21531931				103	4333	4337
							104	GGAAGAAAGA	10	21531989	21531998				104	4395	4404
							105	AGAAGAA	7	21532045	21532051				105	4451	4457
							106	AAGGAA	6	21532105	21532110				106	4511	4516
							107	AGAGA	5	21532125	21532129				107	4531	4535
							108	AGAGGAGAAAG	11	21532143	21532153				108	4549	4559
							109	AAAGA	5	21532279	21532283				109	4685	4689
							110	AAAGA	5	21532301	21532305				110	4707	4711
							111	GGAAAGGA	8	21532320	21532327				111	4726	4733
							112	AGAGAGG	7	21532347	21532353				112	4753	4759
							113	AAAGAA	6	21532375	21532380				113	4781	4786
							114	AAGAAGG	7	21532384	21532390				114	4790	4796
							115	AGAAA	5	21532399	21532403				115	4805	4809
							116	AAGAA	5	21532445	21532449				116	4851	4855
							117	GAGGAA	6	21532459	21532464				117	4865	4870
							118	AGAGAAG	7	21532476	21532482				118	4882	4888
							119	GGAGAAAGAGGAA	13	21532615	21532627				119	5021	5033
							120	AGAAA	5	21532673	21532677				120	5079	5083
							121	AGAAA	5	21532696	21532700				121	5102	5106
							122	AGAAGA	6	21532755	21532760				122	5161	5166
							123	AAGGA	5	21532777	21532781				123	5183	5187
							124	GGAAAGAA	8	21532832	21532839				124	5238	5245
							125	AGAGGAA	7	21532857	21532863				125	5263	5269
							126	AGAAA	5	21532871	21532875				126	5277	5281
							127	AAAGAA	6	21532917	21532922				127	5323	5328
							128	AAAGGAA	7	21532994	21533000				128	5400	5406
							129	AAGAAGAA	8	21533094	21533101				129	5500	5507
							130	GGAAGGAG	8	21533159	21533166				130	5565	5572
							131	AGAGA	5	21533171	21533175				131	5577	5581
							132	GAAAGGA	7	21533195	21533201				132	5601	5607
							133	AGAGA	5	21533212	21533216				133	5618	5622
							134	AAAGGA	6	21533294	21533299				134	5700	5705
							135	AGAGAAAGAA	10	21533408	21533417				135	5814	5823
							136	AAGGAG	6	21533421	21533426				136	5827	5832
							137	AAAGA	5	21533514	21533518				137	5920	5924
							138	GGAAAGAGAGG	11	21533539	21533549				138	5945	5955

Paired L1Md_T (B)

					r-TC in mm10					r-AG in mm10			r-TC in L1Md_T(B)			r-AG in L1MD_T(B)	
Subfamily	Chr:Positions	No.	r-TC Sequence	Length	Start Position	End Position	No.	r-AG Sequence	Length	Start Position	End Position	No.	Star Position	End position	No.	Star Position	End position
L1Md T	ChrX:29660434-29666551	1	CCTTCTTCTTC	11	29660677	29660687	1	AGAGGA	6	29660490	29660495	1	244	254	1	57	62
		2	TCTCT	5	29660838	29660842	2	AGAAA	5	29660577	29660581	2	405	409	2	144	148
		3	TCCTT	5	29661107	29661111	3	GAGAAAGG	8	29660657	29660664	3	674	678	3	224	231
		4	TCCTT	5	29661149	29661153	4	AGGAGGAGG	9	29660692	29660700	4	716	720	4	259	267
		5	TTCCTCC	7	29661627	29661633	5	AAGAGAG	7	29660734	29660740	5	1194	1200	5	301	307
		6	TCTCTCC	7	29661909	29661915	6	AGAGAGAG	8	29660750	29660757	6	1476	1482	6	317	324
		7	TTCCTC	6	29662000	29662005	7	AGAGGAGAGAA	11	29660776	29660786	7	1567	1572	7	343	353
		8	TTTCCTT	7	29662083	29662089	8	AGAAGAA	7	29660828	29660834	8	1650	1656	8	395	401
		9	TCTCTT	6	29662094	29662099	9	AGAGA	5	29660872	29660876	9	1661	1666	9	439	443
		10	CCTTCTTCTC	10	29662635	29662644	10	AGGAA	5	29660902	29660906	10	2202	2211	10	469	473
		11	CCTCTTCTC	9	29662812	29662820	11	AGGAA	5	29660917	29660921	11	2379	2387	11	484	488
		12	TTTCT	5	29662929	29662933	12	AGAGGA	6	29661036	29661041	12	2496	2500	12	603	608
		13	TTCTTT	6	29663145	29663150	13	AAGAAGG	7	29661046	29661052	13	2712	2717	13	613	619
		14	TCCTTCT	7	29663286	29663292	14	AAAGAAA	7	29661070	29661076	14	2853	2859	14	637	643
		15	TTTCT	5	29663344	29663348	15	AGAAGAA	7	29661080	29661086	15	2911	2915	15	647	653
		16	TTTCTCTT	8	29663647	29663654	16	AAAGAG	6	29661094	29661099	16	3214	3221	16	661	666
		17	CTTTCTCC	8	29664036	29664043	17	AAAGAAA	7	29661112	29661118	17	3603	3610	17	679	685
		18	CCTTTCTCT	9	29664268	29664276	18	AGGAAA	6	29661122	29661127	18	3835	3843	18	689	694
		19	CTTTCTT	7	29664496	29664502	19	AGAGAAA	7	29661154	29661160	19	4063	4069	19	721	727
		20	TTCTTC	6	29664555	29664560	20	AAGAGGAAA	9	29661161	29661169	20	4122	4127	20	728	736
		21	CTCTTCTC	8	29664625	29664632	21	AAAGAAA	7	29661237	29661243	21	4192	4199	21	804	810
		22	TCTCCTT	7	29664909	29664915	22	AGAAA	5	29661270	29661274	22	4476	4482	22	837	841
		23	TTTCT	5	29665103	29665107	23	AGGAAAGAAA	10	29661279	29661288	23	4670	4674	23	846	855
		24	TCTTT	5	29665165	29665169	24	AAGAGA	6	29661326	29661331	24	4732	4736	24	893	898
		25	TCCTT	5	29665341	29665345	25	GGAAGAGAGAA	11	29661333	29661343	25	4908	4912	25	900	910
		26	TTCCTC	6	29665527	29665532	26	AGAAGA	6	29661354	29661359	26	5094	5099	26	921	926
		27	CCTCTCCT	8	29665572	29665579	27	AGAGAA	6	29661366	29661371	27	5139	5146	27	933	938
		28	TTCCT	5	29665773	29665777	28	AAGGAAA	7	29661388	29661394	28	5340	5344	28	955	961
		29	TTTCCT	6	29665911	29665916	29	AAAGA	5	29661408	29661412	29	5478	5483	29	975	979
		30	CTCCTT	6	29665968	29665973	30	AGGAAA	6	29661431	29661436	30	5535	5540	30	998	1003
		31	TCTCTT	6	29666152	29666157	31	AAAGGA	6	29661505	29661510	31	5719	5724	31	1072	1077
							32	GAAGAAA	7	29661538	29661544				32	1105	1111
							33	AAAGAAAG	8	29661558	29661565				33	1125	1132
							34	AAGAAG	6	29661584	29661589				34	1151	1156
							35	AGAAA	5	29661617	29661621				35	1184	1188
							36	AAAGA	5	29661670	29661674				36	1237	1241
							37	AGAGA	5	29661756	29661760				37	1323	1327
							38	AGAAGAG	7	29661772	29661778				38	1339	1345
							39	GGAGAAA	7	29661865	29661871				39	1432	1438
							40	AAAGGA	6	29661932	29661937				40	1499	1504
							41	AGAAA	5	29661945	29661949				41	1512	1516
							42	AAGAA	5	29661961	29661965				42	1528	1532
							43	AGAAA	5	29661981	29661985				43	1548	1552
							44	AAGAAGG	7	29661990	29661996				44	1557	1563
							45	AAAGAAGA	8	29662016	29662023				45	1583	1590
							46	AAGAA	5	29662031	29662035				46	1598	1602
							47	AGGAAG	6	29662066	29662071				47	1633	1638
							48	AAAGA	5	29662128	29662132				48	1695	1699
							49	AAGAA	5	29662161	29662165				49	1728	1732
							50	AGGAGA	6	29662185	29662190				50	1752	1757
							51	AGAGAAA	7	29662199	29662205				51	1766	1772
							52	GAAGAAA	7	29662264	29662270				52	1831	1837
							53	AAAGA	5	29662331	29662335				53	1898	1902
							54	AAGGA	5	29662337	29662341				54	1904	1908
							55	AAGAGGAA	8	29662377	29662384				55	1944	1951
							56	AAAGAAA	7	29662436	29662442				56	2003	2009
							57	AGAAA	5	29662536	29662540				57	2103	2107
							58	AAAGGA	6	29662625	29662630				58	2192	2197
							59	AGAAAG	6	29662779	29662784				59	2346	2351
							60	AAGGAAGGAA	10	29662836	29662845				60	2403	2412
							61	AAAGAAAGAAA	11	29662847	29662857				61	2414	2424
							62	AAAGA	5	29662860	29662864				62	2427	2431
							63	AAGAAGAAA	9	29662965	29662973				63	2532	2540
							64	GGAGAGAG	8	29662976	29662983				64	2543	2550
							65	AGAAA	5	29663021	29663025				65	2588	2592
							66	AAAGGAAA	8	29663027	29663034				66	2594	2601
							67	AAGAGGAG	8	29663046	29663053				67	2613	2620
							68	AGGAAA	6	29663062	29663067				68	2629	2634
							69	AAGAAGAA	8	29663105	29663112				69	2672	2679
							70	AAAGAA	6	29663119	29663124				70	2686	2691
							71	GAGAAA	6	29663151	29663156				71	2718	2723
							72	AGAGA	5	29663202	29663206				72	2769	2773
							73	GAAGAAA	7	29663262	29663268				73	2829	2835
							74	AAAGAAA	7	29663416	29663422				74	2983	2989
							75	AAAGA	5	29663503	29663507				75	3070	3074
							76	AGAAAGA	7	29663611	29663617				76	3178	3184
							77	AAAGA	5	29663624	29663628				77	3191	3195
							78	AGAGAA	6	29663630	29663635				78	3197	3202
							79	AAGAA	5	29663704	29663708				79	3271	3275
							80	AAAGA	5	29663817	29663821				80	3384	3388
							81	AGAAA	5	29663852	29663856				81	3419	3423
							82	AGGAA	5	29663909	29663913				82	3476	3480
							83	GGAGAGAAG	9	29663982	29663990				83	3549	3557
							84	AAAGGAGA	8	29664095	29664102				84	3662	3669
							85	GGAAAGGAGGAAG	13	29664120	29664132				85	3687	3699
							86	AGAGA	5	29664195	29664199				86	3762	3766
							87	AAAGAA	6	29664281	29664286				87	3848	3853
							88	GAGAAAGAAA	10	29664297	29664306				88	3864	3873
							89	AAGGAAG	7	29664372	29664378				89	3939	3945
							90	GAAAGA	6	29664380	29664385				90	3947	3952
							91	AAGAA	5	29664396	29664400				91	3963	3967
							92	GAAGAAAGAAA	11	29664413	29664423				92	3980	3990
							93	AAAGAAA	7	29664426	29664432				93	3993	3999
							94	AGAAGA	6	29664438	29664443				94	4005	4010
							95	GGAAAGA	7	29664445	29664451				95	4012	4018
							96	AGAAGGAG	8	29664569	29664576				96	4136	4143
							97	AAGGA	5	29664633	29664637				97	4200	4204
							98	AAAGAA	6	29664640	29664645				98	4207	4212
							99	AGAAA	5	29664701	29664705				99	4268	4272
							100	AGAGA	5	29664723	29664727				100	4290	4294
							101	AGAAA	5	29664764	29664768				101	4331	4335
							102	GGAAGAAAGA	10	29664826	29664835				102	4393	4402
							103	AGAAGAA	7	29664882	29664888				103	4449	4455
							104	AAAGAA	6	29664942	29664947				104	4509	4514
							105	AGAGA	5	29664962	29664966				105	4529	4533
							106	AGAGGAGAAAG	11	29664980	29664990				106	4547	4557
							107	AAGAA	5	29665067	29665071				107	4634	4638
							108	AAAGAAA	7	29665116	29665122				108	4683	4689
							109	AAAGA	5	29665138	29665142				109	4705	4709
							110	GGAAAGGA	8	29665157	29665164				110	4724	4731
							111	AAAGAA	6	29665212	29665217				111	4779	4784
							112	AAGAAGG	7	29665221	29665227				112	4788	4794
							113	AGAAA	5	29665236	29665240				113	4803	4807
							114	GAGAAA	6	29665296	29665301				114	4863	4868
							115	AGAGAAG	7	29665313	29665319				115	4880	4886
							116	AAGGAAA	7	29665353	29665359				116	4920	4926
							117	GGAGAAAGAGGAA	13	29665452	29665464				117	5019	5031
							118	AGAAA	5	29665533	29665537				118	5100	5104
							119	AGAAGA	6	29665592	29665597				119	5159	5164
							120	AAGAAGGA	8	29665611	29665618				120	5178	5185
							121	GGAAAGAA	8	29665669	29665676				121	5236	5243
							122	AGAAA	5	29665708	29665712				122	5275	5279
							123	AAAGAA	6	29665754	29665759				123	5321	5326
							124	AAAGGAA	7	29665832	29665838				124	5399	5405
							125	AAGAAA	6	29665932	29665937				125	5499	5504
							126	GGAAGGAG	8	29665997	29666004				126	5564	5571
							127	AGAGA	5	29666009	29666013				127	5576	5580
							128	GAAAGGA	7	29666033	29666039				128	5600	5606
							129	AGAGA	5	29666050	29666054				129	5617	5621
							130	AGAGAAAG	8	29666246	29666253				130	5813	5820
							131	AAGGAG	6	29666259	29666264				131	5826	5831
							132	AAAGA	5	29666352	29666356				132	5919	5923

							133	GGAAAGAGAGG	11	29666377	29666387				133	5944	5954

Paired L1_Mdo4 (A)

					r-TC in monDom5					r-AG in monDom5			r-TC in L1_Mdo4(A)			r-AG in L1_Mdo4(A)	
Subfamily	Chr:Positions	No.	r-TC Sequence	Length	Start Position	End Position	No.	r-AG Sequence	Length	Start Position	End Position	No.	Star Position	End position	No.	Star Position	End position
L1_Mdo4	ChrX:28754480-28761226	1	CCCTTCCT	8	28754529	28754536	1	AGAAGGAG	8	28754499	28754506	1	50	57	1	20	27
		2	TCCCCCCTCCC	11	28754707	28754717	2	AGAAGA	6	28754508	28754513	2	228	238	2	29	34
		3	TCTCCTCTT	9	28755120	28755128	3	AGAGGA	6	28754562	28754567	3	641	649	3	83	88
		4	TCTTT	5	28755644	28755648	4	AAGGAA	6	28754600	28754605	4	1165	1169	4	121	126
		5	TTCTT	5	28756190	28756194	5	AGGGGAAGA	9	28754675	28754683	5	1711	1715	5	196	204
		6	TCCCT	5	28756396	28756400	6	AGAAGGAAGG	10	28754685	28754694	6	1917	1921	6	206	215
		7	TTTCT	5	28757428	28757432	7	GAGGAAGGA	9	28754845	28754853	7	2949	2953	7	366	374
		8	CCTTCCTCT	9	28757499	28757507	8	AGAGAGGGAG	10	28754857	28754866	8	3020	3028	8	378	387
		9	TTCTTT	6	28757635	28757640	9	AAGAAG	6	28754926	28754931	9	3156	3161	9	447	452
		10	TCCTTTC	7	28757912	28757918	10	AGGGAAG	7	28754999	28755005	10	3433	3439	10	520	526
		11	TCCTT	5	28757966	28757970	11	AGAGGA	6	28755064	28755069	11	3487	3491	11	585	590
		12	CTCCCT	6	28758455	28758460	12	AGGGAAA	7	28755071	28755077	12	3976	3981	12	592	598
		13	TTCTTT	6	28758584	28758589	13	AGAGA	5	28755102	28755106	13	4105	4110	13	623	627
		14	TCTCCCT	7	28758661	28758667	14	AAGAGA	6	28755114	28755119	14	4182	4188	14	635	640
		15	TTCCT	5	28758897	28758901	15	AAGAGGGGAGA	11	28755137	28755147	15	4418	4422	15	658	668
		16	CCTTTCCT	8	28758936	28758943	16	GAGAGGAG	8	28755178	28755185	16	4457	4464	16	699	706
		17	CTCTTT	6	28759158	28759163	17	AAAGAAGGGG	10	28755212	28755221	17	4679	4684	17	733	742
		18	TTTCT	5	28759284	28759288	18	AGAAA	5	28755240	28755244	18	4805	4809	18	761	765
		19	TTTCT	5	28759566	28759570	19	AAGAAGAAAGAAA	13	28755245	28755257	19	5087	5091	19	766	778
		20	TTTCT	5	28760101	28760105	20	AGGGGGAGAGG	11	28755294	28755304	20	5622	5626	20	815	825
		21	TCTCTT	6	28760338	28760343	21	AGAAA	5	28755322	28755326	21	5859	5864	21	843	847
		22	TCCCT	5	28760902	28760906	22	GGAAGAA	7	28755352	28755358	22	6423	6427	22	873	879
		23	TCTCT	5	28761171	28761175	23	AAGAGAGG	8	28755373	28755380	23	6692	6696	23	894	901
							24	GAGAAA	6	28755453	28755458				24	974	979
							25	AAAGGAGA	8	28755502	28755509				25	1023	1030
							26	GAAAGA	6	28755511	28755516				26	1032	1037
							27	GAAGAAGA	8	28755523	28755530				27	1044	1051
							28	GAAAGA	6	28755532	28755537				28	1053	1058
							29	GAGAAA	6	28755539	28755544				29	1060	1065
							30	AAGGAGA	7	28755545	28755551				30	1066	1072
							31	AAAGA	5	28755554	28755558				31	1075	1079
							32	AAAGAGGA	8	28755565	28755572				32	1086	1093
							33	GAAAGA	6	28755574	28755579				33	1095	1100
							34	AAAGAAA	7	28755588	28755594				34	1109	1115
							35	AGAAGAAGAGGGA	13	28755603	28755615				35	1124	1136
							36	AGGGA	5	28755629	28755633				36	1150	1154
							37	AAGAA	5	28755649	28755653				37	1170	1174
							38	AAAGGA	6	28755718	28755723				38	1239	1244
							39	GAGGAAA	7	28755734	28755740				39	1255	1261
							40	AGAAA	5	28755775	28755779				40	1296	1300
							41	AAGAAGAGA	9	28755787	28755795				41	1308	1316
							42	AGAAGA	6	28755820	28755825				42	1341	1346
							43	AAAGAAA	7	28755834	28755840				43	1355	1361
							44	AGGAAA	6	28755861	28755866				44	1382	1387
							45	AGGAAA	6	28755873	28755878				45	1394	1399
							46	AAGAGGGGAAAG	12	28755900	28755911				46	1421	1432
							47	AGAGA	5	28755913	28755917				47	1434	1438
							48	GAAAGAA	7	28755920	28755926				48	1441	1447
							49	AGGAA	5	28755971	28755975				49	1492	1496
							50	AAAGAAA	7	28756007	28756013				50	1528	1534
							51	AAGAAGAAG	9	28756031	28756039				51	1552	1560
							52	GAAGGA	6	28756099	28756104				52	1620	1625
							53	AAAGA	5	28756108	28756112				53	1629	1633
							54	AAGGGAA	7	28756138	28756144				54	1659	1665
							55	AAGAA	5	28756159	28756163				55	1680	1684
							56	AGGGGAAAG	9	28756197	28756205				56	1718	1726
							57	AAAGA	5	28756251	28756255				57	1772	1776
							58	AGAAA	5	28756268	28756272				58	1789	1793
							59	AAGAGA	6	28756297	28756302				59	1818	1823
							60	AAAGAGGGGGGAAAGAAA	18	28756323	28756340				60	1844	1861
							61	AAGAGA	6	28756369	28756374				61	1890	1895
							62	AAGAAA	6	28756403	28756408				62	1924	1929
							63	AGAAGAA	7	28756457	28756463				63	1978	1984
							64	AGAGGGAA	8	28756472	28756479				64	1993	2000
							65	GAAAGGA	7	28756500	28756506				65	2021	2027
							66	AAGAAG	6	28756600	28756605				66	2121	2126
							67	AAGAAA	6	28756607	28756612				67	2128	2133
							68	AAAGAAA	7	28756629	28756635				68	2150	2156
							69	AAAGAAA	7	28756641	28756647				69	2162	2168
							70	GGGAGGGGGAAGAA	14	28756689	28756702				70	2210	2223
							71	AAAGAGG	7	28756723	28756729				71	2244	2250
							72	AGGAAA	6	28756738	28756743				72	2259	2264
							73	AAAGAGGGAAGAA	13	28756776	28756788				73	2297	2309
							74	AGGGGAG	7	28756830	28756836				74	2351	2357
							75	GGAGGGAAG	9	28756863	28756871				75	2384	2392
							76	AAGAGAGGGAGA	12	28756878	28756889				76	2399	2410
							77	AGGGGAAA	8	28756914	28756921				77	2435	2442
							78	AAGAAGGGAGGGGG	14	28756936	28756949				78	2457	2470
							79	AGAAA	5	28756951	28756955				79	2472	2476
							80	AGGAA	5	28756974	28756978				80	2495	2499
							81	AGGGGGA	7	28756981	28756987				81	2502	2508
							82	AGAAGGAAA	9	28757012	28757020				82	2533	2541
							83	GAAAGAAGAAAGGG	14	28757025	28757038				83	2546	2559
							84	AGAAA	5	28757052	28757056				84	2573	2577
							85	AGGAAA	6	28757183	28757188				85	2704	2709
							86	GAGGAAGG	8	28757196	28757203				86	2717	2724
							87	AAGAGGA	7	28757226	28757232				87	2747	2753
							88	AAAGAAGG	8	28757265	28757272				88	2786	2793
							89	AAAGAGA	7	28757327	28757333				89	2848	2854
							90	AGAGAAGG	8	28757335	28757342				90	2856	2863
							91	GAGGAAA	7	28757377	28757383				91	2898	2904
							92	AAAGGAGAAA	10	28757433	28757442				92	2954	2963
							93	AGAGGAG	7	28757445	28757451				93	2966	2972
							94	AAGGA	5	28757455	28757459				94	2976	2980
							95	AAGAAAGAGG	10	28757542	28757551				95	3063	3072
							96	GAGAGAAG	8	28757553	28757560				96	3074	3081
							97	AGAAA	5	28757574	28757578				97	3095	3099
							98	GGAGAAA	7	28757600	28757606				98	3121	3127
							99	AAGGA	5	28757614	28757618				99	3135	3139
							100	AAAGGAA	7	28757622	28757628				100	3143	3149
							101	AGAAA	5	28757718	28757722				101	3239	3243
							102	GGAGAGA	7	28757783	28757789				102	3304	3310
							103	AGAAA	5	28757858	28757862				103	3379	3383
							104	AGAAA	5	28757876	28757880				104	3397	3401
							105	GAAGAAA	7	28757888	28757894				105	3409	3415
							106	AGGGGGAAA	9	28757951	28757959				106	3472	3480
							107	AAGAAGGG	8	28757992	28757999				107	3513	3520
							108	AGAAAG	6	28758037	28758042				108	3558	3563
							109	AGAGA	5	28758079	28758083				109	3600	3604
							110	AAAGGAGAAG	10	28758095	28758104				110	3616	3625
							111	AAAGAA	6	28758123	28758128				111	3644	3649
							112	AAAGGAAAGAAA	12	28758209	28758220				112	3730	3741
							113	AAAGGGAGA	9	28758250	28758258				113	3771	3779
							114	GAAGAGGAAA	10	28758272	28758281				114	3793	3802
							115	AAAGAGGA	8	28758386	28758393				115	3907	3914
							116	AGAAA	5	28758424	28758428				116	3945	3949
							117	AAAGAA	6	28758449	28758454				117	3970	3975
							118	AAGAAA	6	28758461	28758466				118	3982	3987
							119	AAGAAGG	7	28758565	28758571				119	4086	4092
							120	AAAGAGAGAAAGAAA	15	28758634	28758648				120	4155	4169
							121	AAAGA	5	28758709	28758713				121	4230	4234
							122	AGGAA	5	28758767	28758771				122	4288	4292
							123	AAGGA	5	28758775	28758779				123	4296	4300
							124	AGGAAA	6	28758792	28758797				124	4313	4318
							125	AAGAAA	6	28758866	28758871				125	4387	4392
							126	AGAAAG	6	28758914	28758919				126	4435	4440
							127	AGGAA	5	28758923	28758927				127	4444	4448
							128	AAGGA	5	28759039	28759043				128	4560	4564
							129	AGAAA	5	28759079	28759083				129	4600	4604
							130	AGAGAAGAAA	10	28759101	28759110				130	4622	4631
							131	AAGAAA	6	28759111	28759116				131	4632	4637
							132	GAGGAGA	7	28759140	28759146				132	4661	4667
							133	AAAGAA	6	28759187	28759192				133	4708	4713
							134	AGAGAA	6	28759197	28759202				134	4718	4723
							135	AGGAAAG	7	28759239	28759245				135	4760	4766
							136	AAGAA	5	28759315	28759319				136	4836	4840
							137	AGAAAGAGAAA	11	28759322	28759332				137	4843	4853
							138	AGGAA	5	28759370	28759374				138	4891	4895
							139	AGGAA	5	28759396	28759400				139	4917	4921
							140	AAAGAAA	7	28759579	28759585				140	5100	5106
							141	GGAAGAA	7	28759605	28759611				141	5126	5132
							142	AAAGGA	6	28759613	28759618				142	5134	5139
							143	AAAGA	5	28759621	28759625				143	5142	5146
							144	AGGGAAA	7	28759631	28759637				144	5152	5158
							145	AAAGGAAGG	9	28759656	28759664				145	5177	5185
							146	AAGAGA	6	28759734	28759739				146	5255	5260
							147	AGAAAGGAGGA	11	28759741	28759751				147	5262	5272
							148	AAAGA	5	28759807	28759811				148	5328	5332
							149	GGGAGAGA	8	28759877	28759884				149	5398	5405
							150	AAAGAAGGAAA	11	28759953	28759963				150	5474	5484
							151	GGGAAGGGAAAGA	13	28760012	28760024				151	5533	5545
							152	AAAGAG	6	28760048	28760053				152	5569	5574
							153	AGAAGGGAAG	10	28760135	28760144				153	5656	5665
							154	AAGGGA	6	28760267	28760272				154	5788	5793
							155	AAAGAAA	7	28760295	28760301				155	5816	5822
							156	AGAGA	5	28760352	28760356				156	5873	5877
							157	AAAGGAAAG	9	28760418	28760426				157	5939	5947
							158	GGAGGGGA	8	28760438	28760445				158	5959	5966
							159	AAAGGA	6	28760541	28760546				159	6062	6067
							160	AAAGAGA	7	28760593	28760599				160	6114	6120
							161	AAGGAAA	7	28760604	28760610				161	6125	6131
							162	AAGAA	5	28760622	28760626				162	6143	6147
							163	GAGGAAA	7	28760668	28760674				163	6189	6195
							164	AAAGGAA	7	28760742	28760748				164	6263	6269
							165	GGAGGAA	7	28760758	28760764				165	6279	6285
							166	AGGAAG	6	28760790	28760795				166	6311	6316
							167	GAAAGGAG	8	28760807	28760814				167	6328	6335
							168	AGGAGAG	7	28760822	28760828				168	6343	6349
							169	AGAGA	5	28760839	28760843				169	6360	6364
							170	AGGGA	5	28760918	28760922				170	6439	6443
							171	GAGAAA	6	28760928	28760933				171	6449	6454
							172	AGAGGA	6	28760952	28760957				172	6473	6478
							173	AGAGGAAA	8	28760980	28760987				173	6501	6508
							174	GGAGGGGA	8	28761011	28761018				174	6532	6539
							175	GAGGAGAGA	9	28761026	28761034				175	6547	6555
							176	AAGGAAA	7	28761070	28761076				176	6591	6597
							177	AAGGA	5	28761089	28761093				177	6610	6614
							178	AGGGGAGGGG	10	28761128	28761137				178	6649	6658
							179	GGAGGAGGGGAGGAGGAAA	19	28761143	28761161				179	6664	6682

							180	AGAAA	5	28761162	28761166				180	6683	6687

Paired L1_Mdo4 (B)

					r-TC in monDom5					r-AG in monDom5			r-TC in L1_Mdo4(B)			r-AG in L1_Mdo4(B)	
Subfamily	Chr:Positions	No.	r-TC Sequence	Length	Start Position	End Position	No.	r-AG Sequence	Length	Start Position	End Position	No.	Star Position	End position	No.	Star Position	End position
L1 Mdo4	ChrX:45417090-45423836	1	TTCTT	5	45417137	45417141	1	AGAGA	5	45417163	45417167	1	48	52	1	74	78
		2	CCCTTCCTT	9	45417148	45417156	2	AAGGGGAAGG	10	45417292	45417301	2	59	67	2	203	212
		3	CCCTCCTTCT	10	45417224	45417233	3	AGGAA	5	45417363	45417367	3	135	144	3	274	278
		4	TTCCTCC	7	45417489	45417495	4	GGGGAGGAAG	10	45417453	45417462	4	400	406	4	364	373
		5	TTCCTCCCCT	10	45417755	45417764	5	AGGGAGAAA	9	45417465	45417473	5	666	675	5	376	384
		6	TCTTT	5	45418286	45418290	6	AGGGAGG	7	45417578	45417584	6	1197	1201	6	489	495
		7	TCCTT	5	45418530	45418534	7	AGAGAAG	7	45417609	45417615	7	1441	1445	7	520	526
		8	TTCTT	5	45418826	45418830	8	GGAGAGAAA	9	45417621	45417629	8	1737	1741	8	532	540
		9	TCCCT	5	45419027	45419031	9	GGAGAGAG	8	45417631	45417638	9	1938	1942	9	542	549
		10	TCCTTT	6	45419370	45419375	10	AGAGGA	6	45417735	45417740	10	2281	2286	10	646	651
		11	TTTCT	5	45420045	45420049	11	AGGGAAG	7	45417742	45417748	11	2956	2960	11	653	659
		12	CCTTCCTCT	9	45420116	45420124	12	AGAGA	5	45417765	45417769	12	3027	3035	12	676	680
		13	CCTTCTTT	8	45420252	45420259	13	AAGAGA	6	45417777	45417782	13	3163	3170	13	688	693
		14	CTTTCTC	7	45420354	45420360	14	AAGAGGGGAGA	11	45417797	45417807	14	3265	3271	14	708	718
		15	TCCTTTC	7	45420524	45420530	15	GAGAGGAG	8	45417835	45417842	15	3435	3441	15	746	753
		16	TCCTT	5	45420578	45420582	16	AAAGAAGGGA	10	45417869	45417878	16	3489	3493	16	780	789
		17	CTCCCT	6	45421070	45421075	17	AGAAA	5	45417897	45417901	17	3981	3986	17	808	812
		18	TCTCCCT	7	45421274	45421280	18	AAGAAGAAAGAAA	13	45417902	45417914	18	4185	4191	18	813	825
		19	TTCCT	5	45421510	45421514	19	AAAGAG	6	45417948	45417953	19	4421	4425	19	859	864
		20	CCTTTCCT	8	45421549	45421556	20	AGAGGGGGAGGA	12	45417964	45417975	20	4460	4467	20	875	886
		21	CTCTTT	6	45421770	45421775	21	AAAGGAAA	8	45417982	45417989	21	4681	4686	21	893	900
		22	TCTCT	5	45421906	45421910	22	AGAAA	5	45417994	45417998	22	4817	4821	22	905	909
		23	TCTTT	5	45422181	45422185	23	GGAAGAA	7	45418024	45418030	23	5092	5096	23	935	941
		24	TCTCTT	6	45422957	45422962	24	AAGAGAGG	8	45418057	45418064	24	5868	5873	24	968	975
		25	CTCTTT	6	45423260	45423265	25	AAAGAA	6	45418081	45418086	25	6171	6176	25	992	997
		26	TCCCT	5	45423523	45423527	26	AGAAA	5	45418117	45418121	26	6434	6438	26	1028	1032
		27	TCTTT	5	45423786	45423790	27	AAAGGAGA	8	45418165	45418172	27	6697	6701	27	1076	1083
							28	GAAAGA	6	45418174	45418179				28	1085	1090
							29	GAAAGA	6	45418195	45418200				29	1106	1111
							30	GAAGAAGA	8	45418207	45418214				30	1118	1125
							31	GAAAGA	6	45418216	45418221				31	1127	1132
							32	AAAGAAA	7	45418230	45418236				32	1141	1147
							33	AAAGGAAA	8	45418246	45418253				33	1157	1164
							34	AAGGA	5	45418271	45418275				34	1182	1186
							35	AAGAA	5	45418291	45418295				35	1202	1206
							36	AAAGAA	6	45418357	45418362				36	1268	1273
							37	GAGGAAA	7	45418373	45418379				37	1284	1290
							38	AGAAA	5	45418414	45418418				38	1325	1329
							39	AAGAAGAGA	9	45418426	45418434				39	1337	1345
							40	AAGAA	5	45418441	45418445				40	1352	1356
							41	AGAAGA	6	45418459	45418464				41	1370	1375
							42	AAAGAAA	7	45418473	45418479				42	1384	1390
							43	AGGAAA	6	45418500	45418505				43	1411	1416
							44	AAAGAGAA	8	45418511	45418518				44	1422	1429
							45	AAGAGGGAAA	10	45418539	45418548				45	1450	1459
							46	AGGAA	5	45418610	45418614				46	1521	1525
							47	AAGAA	5	45418631	45418635				47	1542	1546
							48	AAAGAAA	7	45418646	45418652				48	1557	1563
							49	AAGAAGAAG	9	45418667	45418675				49	1578	1586
							50	AAGGAA	6	45418689	45418694				50	1600	1605
							51	AAAGAAAGG	9	45418740	45418748				51	1651	1659
							52	AAGGAAA	7	45418774	45418780				52	1685	1691
							53	AAGAA	5	45418795	45418799				53	1706	1710
							54	AGGGGAAAG	9	45418833	45418841				54	1744	1752
							55	AGAAGAA	7	45418861	45418867				55	1772	1778
							56	AAGAA	5	45418872	45418876				56	1783	1787
							57	AAAGAAA	7	45418882	45418888				57	1793	1799
							58	AGAAA	5	45418904	45418908				58	1815	1819
							59	AAGAGAA	7	45418933	45418939				59	1844	1850
							60	AAAGAGGGGAAA	12	45418959	45418970				60	1870	1881
							61	AAAGAAA	7	45418971	45418977				61	1882	1888
							62	AAGAGA	6	45418997	45419002				62	1908	1913
							63	AAGAAA	6	45419034	45419039				63	1945	1950
							64	AAAGAAG	7	45419089	45419095				64	2000	2006
							65	AGAGGGAA	8	45419103	45419110				65	2014	2021
							66	GAAAGGA	7	45419132	45419138				66	2043	2049
							67	AAAGAAA	7	45419240	45419246				67	2151	2157
							68	AAAGAAA	7	45419252	45419258				68	2163	2169
							69	AAAGAAG	7	45419285	45419291				69	2196	2202
							70	GGGAGGGGGGAGAA	14	45419300	45419313				70	2211	2224
							71	AAAGAGG	7	45419334	45419340				71	2245	2251
							72	AAAGA	5	45419343	45419347				72	2254	2258
							73	AGGAAA	6	45419349	45419354				73	2260	2265
							74	AAAGAGGGAAA	11	45419387	45419397				74	2298	2308
							75	AAAGAA	6	45419420	45419425				75	2331	2336
							76	AGGGGAG	7	45419442	45419448				76	2353	2359
							77	GGGGAGGGAAG	11	45419473	45419483				77	2384	2394
							78	AAGAGAGGGAGAGAG	15	45419490	45419504				78	2401	2415
							79	AGAAAGA	7	45419521	45419527				79	2432	2438
							80	AGGGAAA	7	45419532	45419538				80	2443	2449
							81	AAGGGGGGGAA	11	45419541	45419551				81	2452	2462
							82	AGGGAGGGGG	10	45419557	45419566				82	2468	2477
							83	AGAAAGGGAAG	11	45419568	45419578				83	2479	2489
							84	AAAGGGGA	8	45419597	45419604				84	2508	2515
							85	AGAAGGAAA	9	45419629	45419637				85	2540	2548
							86	GAAAGAAGAAA	11	45419642	45419652				86	2553	2563
							87	AAAGGAG	7	45419661	45419667				87	2572	2578
							88	AGAAA	5	45419669	45419673				88	2580	2584
							89	AAGAAA	6	45419800	45419805				89	2711	2716
							90	GAGAAAGG	8	45419813	45419820				90	2724	2731
							91	AAAGAAGG	8	45419882	45419889				91	2793	2800
							92	AAAGAGA	7	45419944	45419950				92	2855	2861
							93	AGGGAAGG	8	45419952	45419959				93	2863	2870
							94	GAGGAAA	7	45419994	45420000				94	2905	2911
							95	AGAAA	5	45420055	45420059				95	2966	2970
							96	AGAGGAA	7	45420062	45420068				96	2973	2979
							97	AAGGA	5	45420072	45420076				97	2983	2987
							98	AGAAA	5	45420087	45420091				98	2998	3002
							99	AAGAAAGAGG	10	45420161	45420170				99	3072	3081
							100	AAGAGAAG	8	45420172	45420179				100	3083	3090
							101	GGAGAAA	7	45420219	45420225				101	3130	3136
							102	AGGGA	5	45420233	45420237				102	3144	3148
							103	AAAGGAA	7	45420241	45420247				103	3152	3158
							104	AAAGAG	6	45420330	45420335				104	3241	3246
							105	AGAAA	5	45420337	45420341				105	3248	3252
							106	AAGGGAA	7	45420392	45420398				106	3303	3309
							107	AAAGAA	6	45420464	45420469				107	3375	3380
							108	AGAAA	5	45420478	45420482				108	3389	3393
							109	GAAGAAA	7	45420500	45420506				109	3411	3417
							110	AGGGGGAAA	9	45420563	45420571				110	3474	3482
							111	AAGAAGGG	8	45420604	45420611				111	3515	3522
							112	AGAAAG	6	45420649	45420654				112	3560	3565
							113	AGAGA	5	45420691	45420695				113	3602	3606
							114	AAAGGAGAAA	10	45420707	45420716				114	3618	3627
							115	AAAGAA	6	45420735	45420740				115	3646	3651
							116	AAAGGAAAGAAA	12	45420825	45420836				116	3736	3747
							117	AAAGGAGA	8	45420866	45420873				117	3777	3784
							118	GAAGAGGAAA	10	45420887	45420896				118	3798	3807
							119	AAAGAGGAAGAAA	13	45421001	45421013				119	3912	3924
							120	AGAAA	5	45421039	45421043				120	3950	3954
							121	AAGAAA	6	45421044	45421049				121	3955	3960
							122	AAAGAA	6	45421064	45421069				122	3975	3980
							123	AAGAAA	6	45421076	45421081				123	3987	3992
							124	AAAGAA	6	45421130	45421135				124	4041	4046
							125	AAAGGAG	7	45421180	45421186				125	4091	4097
							126	AGAGAAAGAAA	11	45421251	45421261				126	4162	4172
							127	AAAGA	5	45421322	45421326				127	4233	4237
							128	AGGAA	5	45421380	45421384				128	4291	4295
							129	AAGGA	5	45421388	45421392				129	4299	4303
							130	GAGGAAA	7	45421404	45421410				130	4315	4321
							131	AGAAA	5	45421479	45421483				131	4390	4394
							132	AGAAAG	6	45421527	45421532				132	4438	4443
							133	AGGAA	5	45421536	45421540				133	4447	4451
							134	AAGGA	5	45421652	45421656				134	4563	4567
							135	AGAAA	5	45421692	45421696				135	4603	4607
							136	AGAGAAA	7	45421714	45421720				136	4625	4631
							137	AAAGAAA	7	45421722	45421728				137	4633	4639
							138	AAGGAA	6	45421799	45421804				138	4710	4715
							139	AGAGAA	6	45421809	45421814				139	4720	4725
							140	AAGAA	5	45421926	45421930				140	4837	4841
							141	AGAGAGAGAAA	11	45421933	45421943				141	4844	4854
							142	AGGAA	5	45421981	45421985				142	4892	4896
							143	AGGAA	5	45422007	45422011				143	4918	4922
							144	AGAAA	5	45422194	45422198				144	5105	5109
							145	GGAAGAA	7	45422223	45422229				145	5134	5140
							146	AAAGA	5	45422232	45422236				146	5143	5147
							147	AAGGA	5	45422239	45422243				147	5150	5154
							148	AGGGAAA	7	45422249	45422255				148	5160	5166
							149	AAAGGAAGGAGGA	13	45422273	45422285				149	5184	5196
							150	AAGAGA	6	45422351	45422356				150	5262	5267
							151	AGAAAGGAGGA	11	45422358	45422368				151	5269	5279
							152	GGGAGAGA	8	45422496	45422503				152	5407	5414
							153	AAAGAAGGAAA	11	45422572	45422582				153	5483	5493
							154	GGGAAGGGAAAGA	13	45422631	45422643				154	5542	5554
							155	AGAAAGAG	8	45422665	45422672				155	5576	5583
							156	AAAGAG	6	45422828	45422833				156	5739	5744
							157	AAGGGA	6	45422884	45422889				157	5795	5800
							158	AAAGAAA	7	45422912	45422918				158	5823	5829
							159	AGAGAGA	7	45422969	45422975				159	5880	5886
							160	GGAGGGGA	8	45423057	45423064				160	5968	5975
							161	AGGGA	5	45423076	45423080				161	5987	5991
							162	AAAGA	5	45423161	45423165				162	6072	6076
							163	AAAGAGA	7	45423212	45423218				163	6123	6129
							164	AAGGAAA	7	45423223	45423229				164	6134	6140
							165	AAGAA	5	45423241	45423245				165	6152	6156
							166	GAGGGGA	7	45423289	45423295				166	6200	6206
							167	AAAGGAA	7	45423363	45423369				167	6274	6280
							168	GGAGGAA	7	45423379	45423385				168	6290	6296
							169	AGGAAG	6	45423411	45423416				169	6322	6327
							170	GAAAGGAG	8	45423428	45423435				170	6339	6346
							171	AGGAGAA	7	45423443	45423449				171	6354	6360
							172	AGAGA	5	45423460	45423464				172	6371	6375
							173	AGGGA	5	45423539	45423543				173	6450	6454
							174	AGAAGA	6	45423567	45423572				174	6478	6483
							175	GGAGGGGA	8	45423626	45423633				175	6537	6544
							176	GAGGAGAGA	9	45423641	45423649				176	6552	6560
							177	AAGAA	5	45423703	45423707				177	6614	6618
							178	GGGAGGGGGGGAGGGAAGAAA	21	45423756	45423776				178	6667	6687
							179	AGAAA	5	45423777	45423781				179	6688	6692
