# Supplementary material for: Convergence in LINE-1 nucleotide variations can benefit redundantly forming triplexes with lncRNA in mammalian X-chromosome inactivation
Source: Mob DNA. 2019 Jul 30;10:33. doi: 10.1186/s13100-019-0173-4 (PMC6664574; doi:10.1186/s13100-019-0173-4)
Supplement: Supplementary file 9 — Whole alignments of r-AG motif sequences in the paired L1s of the three species. Only the first and last parts of the alignments of human paired L1PA5 and L1PA8 are depicted in Fig. 7c. The paired L1Md_T (mouse) and L1_Mdo4 (mouse) with moderate-to-high sequence identities (91 and 89%, respectively) are also shown. In contrast to colored motifs, non-colored motifs reflect variations in nucleotides. A rectangular line surrounding an entire or part of an alignment indicates an area that was analyzed for sequence identities by BLAST. For example, positions 740–6,118 for (A) and 717–6,117 for (B) of L1Md_T indicate areas analyzed by a BLAST search. (PDF 712 kb) [file 13100_2019_173_MOESM9_ESM.pdf]

Additional file 9: Whole alignments of r-AG motif sequences in the paired L1s of the three species

Only the first and last parts of the alignments of human paired L1PA5 and L1PA8 are depicted in Figure 7c. The paired L1Md_T (mouse) and L1_Mdo4 (opossum) with moderate-to-high sequence identities are also shown. In contrast to colored motifs, non-colored motifs reflect variations in nucleotides. A rectangular line surrounding an entire or part of an alignment indicates an area that was analyzed for sequence identities by BLAST. For example, positions 740–6,118 for (A) and 717–6,117 for (B) of L1Md_T indicate areas analyzed by a BLAST search.

hg38 L1PA5 (A) ChrX 69350758–69356897; (B) ChrX 85583162–85589299

No.	Position	r-AG (A)	r-AG (B)	Position	No.
1	26	AGGAA	AGGAA	26	1
2	66	AGAAGA	AGAAGA	66	2
3	201	AGGAAG	AGGAGA	330	3
4	329	AGGAGA	AAGGAGG	521	4
5	628	GAAGAGAG	AGAAA	587	5
6	793	AGAGGAAAGA	GAAGAGAG	628	6
7	935	AAGAGGAAA	AAGGAG	795	7
8	954	AGAAAGGA	AGAAAGAAA	937	8
9	1000	AAAGA	AGAAAGGA	956	9
10	1047	AGAAA	AAAGA	1002	10
11	1091	AAAGAAA	AAAGA	1027	11
12	1154	GAGAGAAGAAGG	AGAAA	1048	12
13	1193	AAAGGAGGAAG	AGAGAA	1073	13
14	1218	AAAGAAG	AAAGGAA	1092	14
15	1239	AAAGA	GAGAGAAGAAGG	1155	15
16	1276	AGAGAAG	AAAGGAGGAAA	1194	16
17	1369	GGAAGAAAGG	AAAGAAG	1219	17
18	1391	AAAGA	AAAGA	1241	18
19	1416	AAGAAGAAGAAG	GGAAGAAAGG	1371	19
20	1430	AAGAGAAA	GAGAAGAGAAG	1418	20
21	1446	AAAGAAA	AAGGAAA	1432	21
22	1467	AAGAAA	AAGAAA	1469	22
23	1488	AAAGA	GAAGAGA	1488	23
24	1575	AGGAGAA	AGGAGAA	1577	24
25	1620	AAGAAA	AGGAAA	1622	25
26	1629	AGAGA	AGAGA	1631	26
27	1641	AAAGA	GAGAAGAG	1656	27
28	1654	GAGAAGAG	GAAGGAAA	1707	28
29	1705	GAAGAAA	AGAGAGAAAGG	1732	29
30	1730	AGAAA	AGAAA	1794	30
31	1754	AAAGAGAAG	AAAGAGAAG	1810	31
32	1792	AAGAAA	AAAGAAA	1843	32
33	1841	AAAGAAA	AAAGGAGAAA	1900	33
34	1898	GAAGGAGAAA	GAGAGA	1938	34
35	1936	GAGAGA	GAAGGAAG	1980	35
36	1967	AAAGAG	GGAAAGGAAA	1998	36
37	1978	GAAGGAAG	AGAGA	2045	37
38	1996	GGAAAGGAA	GAAGAAA	2060	38
39	2043	AAAGA	AAAGA	2174	39
40	2059	AGGAAGAAA	AAAGA	2199	40
41	2175	AAAGA	AGGAGA	2231	41
42	2200	AAAGAG	AGAGA	2251	42
43	2232	AGGAAG	GGAGGAAGA	2282	43
44	2252	AGAGA	AAAGA	2374	44
45	2276	AAAGAA	AAAGAAGA	2382	45
46	2283	GGAGGAAGA	AAAGAAAG	2390	46
47	2368	AAAGA	AAAGGAA	2413	47
48	2376	AAAGAAGA	AAGAAGAG	2430	48
49	2384	AAAGAAAG	AGAGA	2501	49
50	2424	AAGAAGAG	AGAAAGAGG	2509	50
51	2495	AGAGA	AGAAAG	2589	51
52	2505	AAAGAGA	AAGAGGGA	2597	52
53	2583	AGAAAG	AGGAA	2610	53
54	2594	AAGGA	AGAAA	2773	54
55	2604	AGAAA	AAGAAA	2831	55
56	2671	AAGAGAA	AGAAA	2917	56
57	2760	AAAGAA	AAAGA	2923	57
58	2767	AGAAA	AGAGA	2945	58
59	2825	AAGAAA	AAAGA	2951	59
60	2911	AGAAA	GAAGAAAG	3029	60
61	2917	AAAGA	AGGAAAGA	3039	61
62	2945	AAAGA	AAAGAA	3077	62
63	3023	AAGAGAAAG	AGAGAAG	3085	63
64	3033	AGGAAAGA	AAGAAA	3126	64
65	3070	GAAAGAA	AAAGGAGA	3155	65
66	3079	AGAGAAG	AGAGA	3163	66
67	3120	AAGAAA	AAAGA	3205	67
68	3148	GAAGGAGA	AAAGAAAGAAA	3244	68
69	3157	AGAGA	AAGAGAAGAAGA	3254	69
70	3208	AGAAA	AGAAA	3316	70
71	3247	AAAGAAAGAAA	AGAGAA	3331	71
72	3257	AGAGAAAGAAGA	AGAAA	3366	72
73	3319	AGAAA	AAGAAAA	3375	73
74	3338	AGGAGA	AGGAAGAAG	3425	74
75	3373	AGAAA	AAAGAAGAG	3549	75
76	3382	AGAAGAAA	AAAGAAA	3591	76
77	3432	AGGAAGAAG	AAGAAGGAA	3598	77
78	3557	AAGGAGGAG	AGAGA	3660	78
79	3601	AGAAA	AAAGAAAGAGAA	3672	79
80	3668	AGAGA	AAGGA	3808	80
81	3683	AAAGAGAA	AGAGA	4071	81
82	3872	AAAGA	AGGAGAAAGAAA	4137	82
83	4146	AGGAGAAAGAAA	AGGAAA	4165	83
84	4174	AGGAAA	GGAGGAAG	4171	84
85	4180	AGAGGAAG	AGAGAGA	4342	85
86	4228	AGAA	AAAGAGAAA	4384	86
87	4347	AAGAG	AGGAA	4402	87
88	4389	GAAGAGAA	GAAGGA	4426	88
89	4407	AGGAA	AAGGAGGAA	4439	89
90	4431	GAAGGA	AAGGAAA	4463	90
91	4444	AAGGAGAA	GAAAGAGGA	4471	91
92	4477	AAAGAGAA	GGGAGAA	4491	92
93	4496	GGAAGAA	AGGAAGAA	4516	93
94	4521	AGGAAGAA	AAAGAG	4650	94
95	4655	AAAGAG	AAGAAAA	4684	95
96	4689	AAAGAA	AGAGA	4775	96
97	4780	AGAGA	AAGAAA	4810	97
98	4815	AGAAA	GGAAAGGA	4874	98
99	4870	AAGAAA	AAAGAAG	4929	99
100	4879	GGAAAGGA	AAAGA	4989	100
101	4993	AAAGA	AGAGAAAA	5027	101
102	5031	AGAAGAAA	AAGGA	5070	102
103	5074	AAGGA	AAAGAG	5144	103
104	5148	AAAGAG	AAAGGAA	5163	104
105	5167	AAAGGAA	GGAGGAA	5204	105
106	5208	GGAGGAA	AAGAAGAAA	5229	106
107	5284	AAGAAA	GAAGGA	5315	107
108	5320	GAAGGA	AAAGAAGA	5340	108
109	5340	AAAGAAGA	AGAGAAA	5400	109
110	5400	AGAGAAA	AAGAAG	5463	110
111	5471	AGGAAA	AGGAAA	5471	111
112	5488	GGAGAAGA	GGAGAAGGA	5488	112
113	5499	GGAGGAA	GGAGGAA	5499	113
114	5507	AGGAA	AGGAA	5507	114
115	5580	AAGGA	AAGGA	5580	115
116	5594	AGAAA	AGAAA	5594	116
117	5639	AAAGAA	AAAGGA	5639	117
118	5664	AAAGA	AAAGA	5664	118
119	5757	AGAAAA	AAAGA	5711	119
120	5804	AAAGAA	AAGAAA	5757	120
121	5875	AAGGA	AAGGA	5875	121
122	5946	AGGAAGGAGAA	GGAGGAA	5996	122
123	6010	AGGAGA	AGGAGA	6010	123
	6140 bp			6138 bp	

Boxed areas: (A) 1–6137 [1 – 6,137]; (B) 1–6138 [1 – 6,138]

Sequence identity between (A) and (B): 91% (5,621/6,162)
Completely matched r-AG motifs in (A)(B): 63% (77/123)

hg38 L1PA8 (A) Chr12 26310353–26316762; (B) Chr20 39969159–39975570

No.	Position	r-AG (A)	r-AG (B)	Position	No.
1	17	AGGAA	AAAGAA	55	1
2	243	GAGGAAG	AGAGAGGAAAGAAGA/	146	2
3	460	GGAAAGAGG	AAGAGAGG	224	3
4	673	AGGAAG	AAGAGA	300	4
5	770	AAAGAAGG	AGGAA	731	5
6	900	GAAGAGAG	GAGGAA	861	6
7	995	AAAGAA	AGAGA	1054	7
8	1034	AGGAGAG	AAGAGGAAGGAG	1063	8
9	1085	AGAGGAAGGAA	AGGAA	1127	9
10	1190	AGAGAGG	AGAAGAGG	1187	10
11	1210	AGAGGAAA	AGAAAG	1222	11
12	1229	AGAAAG	GAGAAAGAA	1319	12
13	1256	AAAGGA	AAGGA	1404	13
14	1300	AAAGA	AGAAAGAGG	1446	14
15	1331	GAGGAAA	AAAGAAAG	1516	15
16	1502	AAAGGAA	AAAGGA	1538	16
17	1518	AAAGGAG	AAGAGGAAA	1575	17
18	1527	AAGAA	AAGAA	1619	18
19	1549	AAGAAG	GGAAGAAAGAG	1654	19
20	1579	AAGAAA	AGAGAAA	1715	20
21	1670	GGAAGAAAGGA	AAAGGAA	1731	21
22	1685	AGAGA	AAGAAG	1752	22
23	1731	AGAGAAA	AAAGAA	1773	23
24	1738	AAAGAA	GAAAGAGA	1805	24
25	1747	AAAGGAA	AAGAGAA	1860	25
26	1768	AAGAAA	AGGAAA	1904	26
27	1789	AAAGA	AAGAGAAAA	1913	27
28	1876	AGGAGA	GAGAAGA	1938	28
29	1921	AGGAAA	AAGAAA	1953	29
30	1930	AGAGA	GAAGGAAA	1989	30
31	1955	AAGAGAAA	AGAGAGAAAGG	2014	31
32	2006	GAAGGAAA	AGAAA	2076	32
33	2031	AAAGAGAAAGG	AAGAGAAA	2123	33
34	2053	AAAGAAGG	AAAGGAAA	2180	34
35	2093	AGAAA	AAAGGAAA	2218	35
36	2109	AAGAGAAGG	GGAAAGGAG	2260	36
37	2141	GAAAGAAA	GGAAAGGAAA	2278	37
38	2199	AAAGGAAAGAA	AAAGA	2325	38
39	2280	AAAGGAAG	GAAGAAA	2343	39
40	2300	AAGAGAAA	AAAGA	2457	40
41	2362	GAAGAAA	AAAGAAG	2482	41
42	2475	AAAGAAA	AGGAGA	2514	42
43	2494	AAAGAG	AAAGA	2534	43
44	2526	AGGAGA	AAAGAGA	2557	44
45	2546	AAGAGA	GAAGGAAA	2565	45
46	2569	AAAGGAA	AAAGA	2653	46
47	2577	GGAGGAAA	AAAGA	2663	47
48	2664	AAAGA	AAAGAAGAG	2669	48
49	2674	AAAGAA	AGGAGAA	2749	49
50	2680	AAAGAAGG	AGAGA	2780	50
51	2720	AAGAAGAG	AAAGAGA	2790	51
52	2791	AGAGA	AAGAGAGAAA	2863	52
53	2803	AAGAA	AAGAAA	3045	53
54	2879	AAAGAA	GAGAAA	3109	54
55	2890	AAGGA	AAGGAGGAAA	3190	55
56	3056	AAAGAA	AAGAAG	3202	56
57	3117	AAGAAA	AAGAAA	3229	57
58	3203	AGAAA	AAGGAAA	3309	58
59	3286	GAGGAAA	GAAAGAG	3355	59
60	3313	AAAGAAAGAAG	AGAGAAG	3363	60
61	3325	AGGAAAGA	AGAGAA	3397	61
62	3363	AAAGAA	AAGAAA	3404	62
63	3371	AGGAAAG	GAAGAGAA	3432	63
64	3405	AGAAGA	AGAGA	3441	64
65	3412	AAGAAA	AAAGAA	3538	65
66	3440	GAAGGAGA	AGAGAAAGAGAA	3544	66
67	3449	AGAGA	AGAAA	3606	67
68	3505	AAAGA	AGAAAA	3625	68
69	3544	AAAGAAAGAA	AGAGA	3660	69
70	3554	AGAGAGAA	AGAGAAAA	3669	70
71	3616	AGAAA	AGGAAGAAG	3718	71
72	3635	AGAGAA	AAGAAAA	3792	72
73	3670	AGAAA	AGAGAGA	3810	73
74	3679	AGAGGAAA	AAAGAGAAA	3844	74
75	3729	AGAAA	AGAAA	3887	75
76	3856	AAAGAGGAG	AAAGAGGAA	3892	76
77	3900	AGAAA	AGAGA	3954	77
78	3967	AGAGA	AGAAA	4195	78
79	4208	AGAAA	AAAGA	4367	79
80	4381	AAAGA	AAAGAAAA	4433	80
81	4475	AGGAAG	AGGAGAAGAGGAAG	4453	81
82	4521	AGAAA	AAAGA	4507	82
83	4629	AGAGAG	AAGGA	4599	83
84	4671	AAGAAAA	AGAGAAG	4630	84
85	4689	AGGAA	AAAGAAGAA	4672	85
86	4713	GAAGA	AGGAA	4690	86
87	4726	AAGGAGAA	AAGAGA	4705	87
88	4750	AAGAAA	GAAGA	4714	88
89	4758	AAGAGAAGA	AAGGAGAG	4727	89
90	4778	AGGAGAAA	AGAGAAA	4751	90
91	4877	AGAAA	AAGAAGAGA	4759	91
92	4946	AAAGAA	AAGGAGAAG	4804	92
93	5072	AGAAA	AAAGAG	4938	93
94	5127	AAGAAA	AAAGAA	4972	94
95	5136	GGAAAGGA	AGAGA	5089	95
96	5190	AGAAA	AGAAA	5098	96
97	5250	AGAAA	AGAAA	5205	97
98	5288	AGAAGAAA	AAAGA	5265	98
99	5314	AAGAAA	AGAAGAAA	5303	99
100	5329	GGAAAGA	AAAGA	5346	100
101	5406	AAAGAG	AAAGAG	5420	101
102	5466	GGAAGAA	AGAGAAA	5439	102
103	5521	AAGGAA	GGAAGAA	5480	103
104	5576	AAAGGA	GAGAAA	5544	104
105	5601	AAAGAGGA	AAAGGA	5578	105
106	5661	AGAAGAAA	AAGAGAGA	5603	106
107	5731	AGGAAGAA	AAGAAAA	5662	107
108	5759	GAAGAAA	AGAAAA	5733	108
109	5839	GAAGGA	GGAGAAA	5761	109
110	5923	AAAGA	AGGAA	5769	110
111	5970	AAAGA	AAGGA	5842	111
112	6015	AAAGAAAA	AGAAA	5856	112
113	6063	AAAGGA	AAAGAGGA	5901	113
114	6131	AGGAA	AAAGA	5926	114
115	6137	AGAAA	AAAGA	5973	115
116	6186	AAAGAA	AAGAAAA	6018	116
117	6246	AAAGGAAGG	AAAGAA	6066	117
118	6264	AGGAGAA	AAAGAA	6137	118
	6410 bp			6412 bp	

Boxed areas: (A) 1153–6393 [1,153 – 6,393]; (B) 1150–6402 [1,150 – 6,402]

Sequence identity between (A) and (B): 83% (4,442/5,324)
Completely matched r-AG motifs in (A)(B): 43% (47/109)

mm10 L1Md_T (A) ChrX 21527595–21533712; (B) ChrX 29660434–29666551

No.	Position	r-AG (A)	r-AG (B)	Position	No.
1	46	AGAGGA	AGAGGA	57	1
2	131	AGGAA	AGAAA	144	2
3	137	AAGGA	GAGAAAGG	224	3
4	216	AGAGAG	AGGAGGAGG	259	4
5	235	AAAGGAAGA	AAGAGAG	301	5
6	336	GAGGAAG	AAGAGAG	317	6
7	402	AAAGGA	AGAGGAGAGAA	343	7
8	412	AGAGAAA	AAGAAGA	395	8
9	456	AGGAGGA	AGAGA	439	9
10	491	AGAAG	AGGAA	469	10
11	531	AAGAA	AGGAA	484	11
12	546	AGAAA	AGAGGA	603	12
13	617	AAGAGAAA	AAGAAGG	613	13
14	675	AAGAAGGAAA	AAAGAAA	637	14
15	688	AGGAAAG	AAGAGAA	647	15
16	720	AAAGAG	AAAGAG	661	16
17	727	AAAGAA	AAAGAAA	679	17
			AGGAAA	689	18
18	744	AAGAG	AAGAGAA	721	19
19	754	AAGAAA	AAGAGGAAA	728	20
20	818	AGAAA	AAAGAAA	804	21
21	827	AAAGAAA	AGAAA	837	22
22	854	GGAGAGGAGA	AGGAAGAAA	846	23
23	869	AGGAAGAAA	AAGAGA	893	24
24	881	AGGAA	GGAAGAGAGAA	893	25
25	916	AGGAGA	AAGAGA	921	26
26	926	AAGAAGA	AAGAAGA	933	27
27	975	AAGAAA	AAGAAA	955	28
28	995	AAAGA	AAAGA	975	29
29	1018	AGGAAA	AGGAAA	998	30
30	1037	GAAAGA	AAAGGA	1072	31
31	1097	AGGAAA	GAAGAAA	1105	32
32	1121	AAGAGAAAA	AAAGAAAG	1125	33
33	1142	AAAGAAAGAGA	AAGAAG	1151	34
34	1168	AAGAAG	AGAAA	1184	35
35	1201	AAGAAA	AAAGA	1237	36
36	1206	AGAAA	AGAGA	1323	37
37	1254	AAGAAA	AGAGAAG	1339	38
38	1340	AGAGA	GAGGAAA	1432	39
39	1347	AAGAAAG	AAGAGA	1499	40
40	1356	AAGAGA	AGAAA	1512	41
41	1388	AAGAGAA	AAGAA	1528	42
42	1449	GGAGAAA	AGAAA	1548	43
43	1516	AAAGGA	AAAGAAGG	1557	44
44	1574	AAGAAAG	AAAGAAGAA	1583	45
45	1600	AAGAAAA	AAGAA	1598	46
46	1615	AAGAA	AGGAAG	1633	47
47	1648	AAAGGAAG	AAAGA	1695	48
48	1712	AAAGA	AAGAAA	1728	49
49	1769	AAAGA	AGGAGA	1752	50
50	1789	AAAGAGA	AGAGAAA	1766	51
51	1818	AGAAA	GAAGAAA	1831	52
52	1846	GAAGAAA	AAAGA	1898	53
53	1913	AAAGA	AAGGA	1904	54
54	1919	AAGGAGG	AAGGAGGAA	1944	55
55	2018	AAAGA	AAGAAA	2003	56
56	2118	AGAAA	AGAAA	2103	57
57	2129	AAGAA	AAAGA	2192	58
58	2207	AAAGGA	AAGAAG	2346	59
59	2429	AAGGAGA	AAGGAGGAA	2403	60
60	2440	GAAGAAAGAA	AAAGAAAGAAA	2414	61
61	2527	AAGAAGAA	AAGAAA	2427	62
62	2559	AAGGAAA	AAAGAAGAAA	2532	63
63	2569	AGAGAGAG	GGAGAGAG	2543	64
64	2620	AAAGGAG	AGAAA	2588	65
65	2655	AGGAAA	AGGAAAAA	2594	66
66	2692	AAGAAA	AAAGAGGAG	2613	67
67	2724	GAGAAA	AGGAAA	2629	68
68	2802	AGAAA	AAAGAAGAA	2672	69
69	2810	AAAGGAAG	AAAGAA	2686	70
70	2832	GAAGAAA	AGAGAAA	2718	71
71	3023	AAAGAAG	AGAGA	2769	72
72	3032	AGGAA	GAAGAAA	2829	73
73	3072	AAAGAAGA	AAAGAAA	2983	74
74	3116	AAAGAA	AGAAGA	3070	75
75	3185	AAAGAAG	AAGAAAGA	3178	76
76	3196	AAGAA	AAAGA	3191	77
77	3202	AAGAAA	AGAGAA	3197	78
78	3276	AGAAA	AAGAA	3271	79
79	3333	AAGAAGAA	AAAGA	3384	80
80	3425	GAAGAAG	AGAAA	3419	81
81	3471	GAAGAAA	AGGAA	3476	82
82	3479	GAGGAA	GGAGAGAAG	3549	83
83	3553	GGAGGAAA	AAGAAGAA	3662	84
84	3595	AGGAA	GGAAAGGAGGAAG	3687	85
85	3666	AAGGAGG	AGAGA	3762	86
86	3690	GGAAAGGAAGAA	AAAGAA	3848	87
87	3765	AAGAAGA	AAGGAAGAAA	3864	88
88	3851	AAAGGAA	AAGGAAG	3939	89
89	3867	GAGAAAGAAA	GAAAGA	3947	90
90	3879	AGGAAGAA	AAGAA	3963	91
91	3942	AAGGAAG	AAGAAGAAAA	3980	92
92	3950	GAGAAAGAA	AAGAGAA	3993	93
93	3966	AAGAAA	AAGAGA	4005	94
94	3982	GAAGAAAGAA	GGAAAGA	4012	95
95	3995	AAAGAAGA	AAAGAGGAG	4136	96
96	4007	AGAAAAG	AAGGA	4200	97
97	4014	GGAAAGA	AAAGAA	4207	98
98	4138	AGAAAGG	AGAAA	4268	99
99	4181	AGGAGAG	AGAGA	4290	100
100	4202	AAAGA	AAAGAA	4331	101
101	4209	AAAGAA	GGAAGAAAGA	4393	102
102	4292	AGAAA	AGAGAGAA	4449	103
103	4333	AGAAA	AAAGAAA	4509	104
104	4395	GGAAGAAAGA	AGAGA	4529	105
105	4451	AGAGAAA	AGAGGAAGAAG	4547	106
106	4511	AAGGAAA	AAGAA	4634	107
107	4531	AGAGA	AAAGAAA	4683	108
108	4549	AGAGGAGAAAG	AAAGA	4705	109
109	4685	AAAGA	GGAAAGA	4724	110
110	4707	AAAGAA	AAAGAA	4779	111
111	4726	GGAAAGGA	AAGAAGG	4788	112
112	4753	AGAGAGG	AGAAA	4803	113
113	4781	AAAGAA	AAGAA	4863	114
114	4790	AAGAAGG	AGAGAAG	4880	115
115	4805	AAGAAA	AAAGAAAA	4920	116
116	4851	AAGAA	GGAGAAAGAAGGAA	5019	117
117	4865	AAGGAAA	AAGAA	5100	118
118	4882	AAAGAAAG	AGAGAA	5159	119
119	5021	GGAGAAAGAGGAA	AAAGAAGGA	5178	120
120	5079	AGAAA	GGAAGAAA	5236	121
121	5102	AGAAA	AGAAA	5275	122
122	5161	AGAAGAA	AAAGAAA	5321	123
123	5183	AAGGA	AAAGGAAA	5399	124
124	5238	GGAAAGAA	AAGAAA	5499	125
125	5263	AAAGGAA	GGAAGGAG	5564	126
126	5277	AGAAAGA	AGAGA	5576	127
127	5323	AAAGAA	GAAAGGA	5600	128
128	5400	AAAGAAGA	AGAGA	5617	129
129	5500	AAGAGAAA	GAAGAAAG	5813	130
130	5565	GGAAGAGAG	AAGGAG	5826	131
131	5577	AGAGA	AAAGA	5919	132
132	5601	GAAAGGA	GGAAAGAGAGG	5944	133
133	5618	AGAGA			
134	5700	AAAGGA			
135	5814	AGAGAAAGAA			
136	5827	AAGGAG			
137	5920	AAAGA			
138	5945	GGAAAGAGAGG			
	6118 bp			6118 bp	

Boxed areas: (A) 740–6118 [740 – 6,118]; (B) 717–6117 [717 – 6,117]

Sequence identity between (A) and (B): 91% (4,911/5,417)
Completely matched r-AG motifs in (A): 45% (54/121)
(B): 47% (54/115)

Mdo5 L1_Mdo4 (A) ChrX 28754480–28761226; (B) ChrX 45417090–45423836

No.	Position	r-AG (A)	r-AG (B)	Position	No.
1	20	AGAGGAGG	AGAGA	74	1
2	29	AGAGAA	AAGGGGAAGG	203	2
3	83	AGAGGA	AGGAA	274	3
4	121	AAGGAA	GGGGAGGAAG	364	4
5	196	AGGGGAAGA	AGGGGAGAAA	376	5
6	206	AGAAGGAAGG	AGGGAGG	489	6
7	366	GAGGAGGAA	AGAGAAG	520	7
8	378	AAGAGGGAG	GGAGAGAAA	532	8
9	447	AAGAAG	GGAGAGAG	542	9
10	520	AGGGAAG			
11	585	AGAGGA	AGAGGA	646	10
12	592	AGGGAAA	AGGGAAG	653	11
13	623	AGAGA	AGAGA	676	12
14	635	AAGAGA	AAGAGA	688	13
15	658	AAGAGGGGAGA	AAGAGGGGAGA	708	14
16	699	GAGAGGAG	GAGAGGAG	746	15
17	733	AAAGAAAGGGG	AAAGAAGGGGA	780	16
18	761	AGAAA	AGAAA	808	17
19	766	AAGAAGAAAGAAA	AAGAAGAAAGAAA	813	18
20	815	AGGGGGAGAG	AAAGAG	859	19
21	843	AGAAA	AGAGGGGGAGGA	875	20
22	873	GGAAGAA	AAAGGAAA	893	21
23	894	AAGAGAGG	AAAGAAA	905	22
24	974	GAGAAA	GGAAGAA	935	23
25	1023	AAAGGAGA	AAGAGAGG	968	24
26	1032	GAAAGA	AAAGAA	992	25
27	1044	GAAGAAGAA	AGAAA	1028	26
28	1053	AGAAGAA	AAAGAGGA	1076	27
29	1060	GAGAAA	GAAAGA	1085	28
30	1066	AAGGAGA	GAAAGA	1106	29
31	1075	GAAGA	GAAGAAGA	1118	30
32	1086	AAAGAGGA	GAAAGA	1127	31
33	1095	GAAAGA	AAAGAAA	1141	32
34	1109	AAAGAAA	AAAGGAAA	1157	33
35	1124	AGAGAAGAGGGG	AAGGA	1182	34
36	1150	AAGAGA	AGAAA	1202	35
37	1170	AAGAAA	AAAGAA	1268	36
38	1239	AAAGGA	GAGGAAA	1284	37
39	1255	GAGGAAA	AGAAA	1325	38
40	1296	AGAAA	AAAGAAGAGA	1337	39
41	1308	AAGAAGAGA	AAGAA	1352	40
42	1341	AGAGA	AGAGA	1370	41
43	1355	AAAGAAA	AAAGAAA	1384	42
44	1382	AGGAAA	AGGAAA	1411	43
45	1394	AGGAAA	AAGAGAA	1422	44
46	1421	AAGAGGGGAAAG	AAAGAGGGAAA	1450	45
47	1434	AGAGA	AGGAA	1521	46
48	1441	GAAAGAA	AAGAA	1542	47
49	1492	AGGAA	AAAGAAAA	1557	48
50	1528	AAAGAAA	AAAGAAGAAG	1578	49
51	1552	AAGAAGAAG	AAGGAA	1600	50
52	1620	GAAGGA	AAAGAAAGG	1651	51
53	1629	AAGGAAA	AAGGAAA	1685	52
54	1659	AAGGGAA	AAGAA	1706	53
55	1680	AAGAA	AGGGGAAAG	1744	54
56	1718	AAGGGAAAG	AGAAGAA	1772	55
57	1772	AAAGA	AAGAA	1783	56
58	1789	AAGAAA	AAAGAAAA	1793	57
59	1818	AAGAGA	AAGAAA	1815	58
60	1844	AAAGAGGGGGGAAAGAAA	AAGAGAA	1844	59
61	1890	AAGAGA	AAAGAGGGGAAA	1870	60
62	1924	AAGAAA	AAAGAAAA	1882	61
63	1978	AGAAGAA	AAGAGA	1908	62
64	1993	AGAGGAA	AAGAAA	1945	63
65	2021	GAAAGGA	AAAGAAAG	2000	64
66	2121	AAGAGA	AAGGGGAA	2014	65
67	2128	AAGAAA	GAAAGGA	2043	66
68	2150	AAAGAAA	AAAGAAA	2151	67
69	2162	AAAGAAA	AAAGAAA	2163	68
70	2210	GGGAGGGGAGAAA	AAAGAAAG	2196	69
71	2244	AAAGAGG	AAAGAGGGGGGGAA	2211	70
72	2259	AGGAAA	AAAGAGG	2245	71
73	2297	AAAGAGGGAAGAA	AAAGA	2254	72
74	2351	AGGGGAG	AGGAAA	2260	73
75	2384	GGAGGGAAG	AAAGAGGGGAA	2298	74
76	2399	AAGAGAGGAGGG	AAAGAA	2331	75
77	2435	AGGGGAA	AGGGGAG	2353	76
78	2457	AAGAAGGGAGGGG	GGGGAGGGAAG	2384	77
79	2472	AGAAA	AAGAAGGGAGAGAG	2401	78
80	2495	AGGAA	AAAGAAGA	2432	79
81	2502	AGGGGAA	AGGGAAA	2443	80
82	2533	AAAGGAA	AAAGGGGGAAA	2452	81
83	2546	GAAAGAAGAAAGGG	AGGAGGGGG	2468	82
84	2573	AGAAA	AAAGAGGGAAG	2479	83
85	2704	AGGAAA	AAGGGGA	2508	84
86	2717	GAGGAAGG	AGAGGAAA	2540	85
87	2747	AAAGAGA	GAAAGAAGAAA	2553	86
88	2786	AAAGAAGA	AAAGAGGG	2572	87
89	2848	AAAGAAGA	AGAAA	2580	88
90	2856	AGAGAAGG	AAGAAA	2711	89
91	2898	GAGGAAA	GAGAAAGG	2724	90
92	2954	AAAGGAGAA	AAAAGAAG	2793	91
93	2966	AGGAGAAA	AAGAGGAGA	2855	92
94	2976	AAGGA	AGGGAAGG	2863	93
95	3063	AAAGAAAGAGG	GAGGAAA	2905	94
96	3074	GAGAGAAG	AGAAA	2966	95
97	3083	AAAGAAA	AAAGAGGA	2973	96
98	3121	GGGAGAA	AAAGAA	2983	97
99	3135	AAGGA	AGAAA	2998	98
100	3143	AAAGAAA	AAGAAAGAGG	3072	99
101	3239	AAAGAAA	AAGAGAA	3083	100
102	3304	GGAGAGA	GGAGAAA	3130	101
103	3379	AGAAA	AGGGA	3144	102
104	3397	AGAAA	AAAGGAAA	3152	103
105	3409	GAAGAAA	AAAGAG	3241	104
106	3472	AGGGGAGGA	AAAGAA	3248	105
107	3513	AAGAAGGG	AAGGGAA	3303	106
108	3558	AGAAAG	AAGGAAA	3375	107
109	3600	AGAGA	AGAAA	3389	108
110	3616	AAAGGAAAGAG	GAAGAAAA	3411	109
111	3644	AAAGAAA	AAGGGGAAA	3474	110
112	3730	AAAGAAAGAAA	AAAGAAGGG	3515	111
113	3771	AAAGAAAG	AAAGAAG	3560	112
114	3793	GAAGAGGAAA	AAAGAA	3602	113
115	3907	AAAGGAGGA	AAAGGAGAAA	3618	114
116	3945	AGAAA	AAAGAAA	3646	115
117	3970	AAAGAAA	AAAGAAAGAAA	3736	116
118	3982	AAAGAA	AAAGAAGAA	3777	117
119	4086	AAGAGGAAA	AAGAGGGGAAA	3798	118
120	4155	AAAGAGAGAAAGAAA	AAAGAGGAGGAAA	3912	119
121	4230	AAAGA	AGAAA	3950	120
122	4288	AGAAAGA	AAGAAA	3955	121
123	4296	AAGAAA	AAGAAA	3975	122
124	4313	AGGAAA	AAGAAA	3991	123
125	4387	AAGAAA	AAGAAA	4041	124
126	4435	AAGAAGG	AAAGAAAG	4091	125
127	4466	AAAAGAAA	AAAGAAAAGAAA	4162	126
128	4560	AAGAA	AAAGA	4233	127
129	4600	AGGAA	AGGAA	4291	128
130	4622	AAGAGAAGAA	AAGGA	4299	129
131	4632	AAGGAA	GAGGAAA	4315	130
132	4661	GAGGAA	AGAAA	4390	131
133	4708	AAAGAA	AAAGAAG	4438	132
134	4718	AGAGAA	AGGAA	4447	133
135	4760	AAGGAAG	AAGGA	4563	134
136	4836	AAGAAA	AAGAA	4603	135
137	4843	AGAAAGAAGAAA	AAAGAAA	4625	136
138	4891	AGGAA	AAAGAAAA	4633	137
139	4917	AAGAAA	AAGGAA	4710	138
140	5100	AAAGAAA	AGAGAA	4720	139
141	5126	GGAAGAA	AAGAA	4837	140
142	5134	AAAGGA	AAAGAGAAGAAA	4844	141
143	5142	AAAGA	AGGAA	4892	142
144	5152	AGGGGAA	AGGAA	4918	143
145	5177	AAAGAAGG	AAGAGGAA	5105	144
146	5255	AAGGA	GGAAGAA	5134	145
147	5262	AAGAAGGAGA	AAAGAA	5143	146
148	5328	AAAGA	AAGGA	5150	147
149	5398	GGGAGAAG	AAAGGGGAA	5160	148
150	5474	AAAGAAAGGAAAAA	AAAGAAGGAAGGA	5184	149
151	5533	GGGAAAGGGAAAAA	AAGAGA	5262	150
152	5569	AAAGAG	AGAAAGGGGA	5269	151
153	5656	AGAGAAGGGAG	GGGAAGGA	5407	152
154	5788	AAAGGA	AAAGAAAGGAA	5483	153
155	5816	AAAGAAA	GGGAAGGGAAAGA	5542	154
156	5873	AAGGA	AAAGAAAG	5576	155
157	5939	AAAGGAAAG	AAAGAG	5739	156
158	5959	GGAGGGGA	AAGGAA	5795	157
159	6062	AAAGA	AAAGAAA	5823	158
160	6114	AAAGAAAA	AGAGAGA	5880	159
161	6125	AAGGAAA	GGAGGGGA	5968	160
162	6143	AAGAA	AAGGA	5987	161
163	6189	GAGGAAA	GAAAGA	6072	162
164	6263	AAAGAAA	AAAGAAGA	6123	163
165	6279	AGGAAA	AAGGAAA	6134	164
166	6311	AGGAG	AGGAA	6152	165
167	6328	GAAAGAG	GAGGGGA	6200	166
168	6343	AAGAGAG	AAGAAA	6274	167
169	6360	AGAGA	GGAGGAA	6290	168
170	6439	AGGAA	AGGAAG	6322	169
171	6449	GAGAAA	GAAAGAAG	6339	170
172	6473	AGAGGA	AGGAGAA	6354	171
173	6501	AGGGAAA	AGAGA	6371	172
174	6532	GGAGGGA	AGGGA	6450	173
175	6547	GGAGGAGAA	AGAGAA	6478	174
176	6591	AAGGAA	GGAGGGGA	6537	175
177	6610	AAAGA	GAGGAGAGA	6552	176
178	6649	AGGGGAGGG	AAAGAA	6614	177
179	6664	GGAGGAGGGGAGAGGAAA	GGAGGGGGGGAGGGAGAAA	6667	178
180	6683	AGAAA	AGAAA	6688	179
	6747 bp			6747 bp	

Boxed areas: (A) 566–6734 [566 – 6,734]; (B) 627–6739 [627 – 6,739]

Sequence identity between (A) and B): 89% (5,520/6,230)
Completely matched r-AG motifs in (A)(B): 55% (94/170)
