# Supplementary material for: Convergence in LINE-1 nucleotide variations can benefit redundantly forming triplexes with lncRNA in mammalian X-chromosome inactivation
Source: Mob DNA. 2019 Jul 30;10:33. doi: 10.1186/s13100-019-0173-4 (PMC6664574; doi:10.1186/s13100-019-0173-4)
Supplement: Supplementary file 10 — Detailed data of r-AG motifs in the paired representative L1 subfamilies from the three species. The following table summarizes the data presented in Fig. 8. The order of human subfamilies is based on its phylogenetic tree, while the order of mouse and opossum subfamilies is based on values of L1 sequence identities. r-AG motif identities of the paired L1s with no significant similarity were not determined. NS, no significant sequence similarity; ND, not determined; r-AG MLD, r-AG motif–length distribution. (PDF 323 kb) [file 13100_2019_173_MOESM10_ESM.pdf]

Additional file 10: Detailed data of r-AG motifs in the paired representative L1 subfamilies from the three species

The following table summarizes the data presented in Figure 8. The order of human subfamilies is based on its phylogenetic tree, while the order of mouse and opossum subfamilies is based on values of L1 sequence identities. r-AG motif identities of the paired L1s with no significant similarities were not determined. NS, no significant sequence similarity; ND, not determined; r-AG MLD, r-AG motif–length distribution.

Species	Subfamily	L1 Sequence Identity (%)		r-AG motif Identity (%)		r-AG motif Occupancy (%)	r-AG MLD KS distance	Chr: Positions	L1 length (bp)	No. r-AG	No. r-TC
Human	L1HS (A)	99.1	5,980/6,034	96.9	123/127	14.0	0.009	ChrX: 106469286-106475319	6,034	127	23
	L1HS (B)			96.1	123/128	14.3		ChrX: 147653735-147659767	6,033	129	21
	L1PA2 (A)	97.3	5,863/6,027	91.2	114/125	13.9	0.028	Chr1: 82250045-82256069	6,025	125	21
	L1PA2 (B)			90.5	114/126	13.8		ChrX 88341843-88347867	6,025	126	23
	L1PA3 (A)	95.3	5,863/6,154	81.9	104/127	13.7	0.025	ChrX: 125838861-125845012	6,152	127	26
	L1PA3 (B)			78.8	104/132	14.2		ChrX: 128732738-128738890	6,153	132	23
	L1PA4 (A)	91.3	5,614/6,146	67.2	78/116	12.7	0.026	ChrX: 125838861-125845012	6,133	116	31
	L1PA4 (B)			61.9	78/126	13.7		ChrX: 128732738-128738890	6,133	126	28
	L1PA5 (A)	91.2	5,621/6,162	62.6	77/123	13.5	0.049	ChrX: 69350758-69356897	6,140	123	28
	L1PA5 (B)			62.6	77/123	13.8		ChrX: 85583162-85589299	6,138	123	25
	L1PA6 (A)	89.0	5,465/6,143	57.4	70/122	13.0	0.042	Chr1: 121477125-121483277	6,131	122	27
	L1PA6 (B)			60.3	70/116	12.4		Chr1: 147006521-147012672	6,130	116	27
	L1PA7 (A)	87.0	5,565/6,399	44.4	52/117	12.2	0.055	Chr5: 112538669-112545103	6,131	117	34
	L1PA7 (B)			41.6	52/125	13.2		Chr5: 180889406-180895840	6,130	126	28
	L1PA8 (A)	83.4	4,442/5,324	43.1	47/109	12.3	0.034	Chr9: 29888317-29894745	6,429	119	34
	L1PA8 (B)			43.1	47/109	12.5		Chr9: 115723564-115729988	6,425	126	31
	L1PA8A (A)	83.7	4,532/5,414	34.7	41/118	12.5	0.024	Chr12: 26310353-26316762	6,410	118	30
	L1PA8A (B)			33.1	41/124	13.3		Chr20: 39969159-39975570	6,412	118	31
	L1PA10 (A)	81.2	1,927/2,374	38.1	40/105	13.4	0.033	Chr5: 92829707-92835766	6,060	120	27
	L1PA10 (B)			39.6	40/101	12.7		Chr5: 123983065-123989123	6,059	114	24
	L1PA11 (A)	80.0	4,480/5,599	21.5	23/107	11.6	0.034	Chr5: 114866429-114872869	6,441	113	34
	L1PA11 (B)			19.3	23/119	12.6		Chr8: 79033667-79040102	6,436	122	33
	L1PA12 (A)	77.8	3,102/3,985	25.3	19/75	12.3	0.119	ChrX: 52083347-52089603	6,257	113	36
	L1PA12 (B)			22.4	19/85	12.2		ChrX: 98910031-98916276	6,246	122	33
	L1PA13 (A)	80.3	2,165/2,696	27.5	14/51	10.1	0.091	Chr5: 109289218-109295633	6,416	97	39
	L1PA13 (B)			23.7	14/59	13.8		Chr8: 119462888-119469291	6,404	137	27
	L1PA14 (A)	79.8	1,503/1,884	13.6	11/81	11.9	0.097	Chr3: 167210972-167217890	6,919	126	46
	L1PA14 (B)			12.8	11/86	12.6		Chr4: 58424991-58431952	6,962	130	41
	L1PA15 (A)	NS	NS	ND	ND	11.4	0.065	Chr5: 12131805-12138332	6,528	113	29
	L1PA15 (B)			ND	ND	11.8		Chr8: 124128949-124135471	6,523	120	23
	L1PB1 (A)	81.7	4,700/5,751	35.1	39/111	12.3	0.030	Chr1: 248181162-248197195	6,034	111	29
	L1PB1 (B)			33.1	39/118	12.8		Chr1: 248204247-248210286	6,040	118	25
	L1PB2 (A)	77.9	2,878/3,693	25.0	16/64	11.7	0.042	ChrX: 90842819-90849003	6,185	110	25
	L1PB2 (B)			22.5	16/71	13.4		ChrX:128383405-128389591	6,187	124	26
	L1PB3 (A)	77.6	1,548/1,996	22.6	7/31	14.0	0.035	Chr2: 115978677-115985372	6,696	127	37
	L1PB3 (B)			17.1	7/41	11.8		Chr13: 93236755-93243424	6,670	120	40
	L1PB4 (A)	NS	NS	ND	ND	11.7	0.090	Chr4: 33334416-33341192	6,777	118	30
	L1PB4 (B)			ND	ND	13.3		Chr5: 108277904-108284700	6,797	136	22
	L1MA1 (A)	78.3	734/937	16.7	3/18	14.2	0.047	Chr8: 105815923-105822018	6,096	127	28
	L1MA1 (B)			18.8	3/16	14.9		Chr9: 25946271-25952312	6,042	128	29
	L1MA2 (A)	77.7	4,972/6,403	22.9	30/131	14.4	0.038	Chr3: 104967091-104973370	6,280	131	29
	L1MA2 (B)			22.9	30/131	14.6		Chr5: 119618133-119624408	6,276	133	24
	L1MA3 (A)	NS	NS	ND	ND	14.4	0.074	Chr12: 18958789-18965653	6,865	141	36
	L1MA3 (B)			ND	ND	13.9		Chr13: 28915120-28921960	6,841	143	21
Mouse	L1Md_F2 (A)	99.7	6,123/6,139	98.4	126/128	14.2	0.012	ChrX: 27681403-27687541	6,139	128	34
	L1Md_F2 (B)			99.2	126/127	14.2		ChrX: 28030950-28035913	6,139	127	35
	L1_Mus1 (A)	98.8	6,831/6,916	96.4	135/140	13.5	0.019	ChrX: 26441550-26448462	6,913	140	37
	L1_Mus1 (B)			94.4	135/143	13.8		ChrX: 29583408-29590320	6,913	143	36
	L1Md_A (A)	98.1	6,057/6,173	90.2	120/133	14.5	0.026	ChrX: 33246126-33252301	6,176	133	28
	L1Md_A (B)			89.6	120/134	14.4		ChrX: 60952054-60958229	6,176	134	28
	L1Md_T (A)	90.7	4,911/5,417	44.6	54/121	15.1	0.012	ChrX: 21527595-21533712	6,118	138	33
	L1Md_T (B)			47.0	54/115	14.5		ChrX: 29660434-29666551	6,118	133	31
	L1_Mus2 (A)	86.1	5,211/6,050	38.1	51/134	14.7	0.120	ChrX: 92901986-92908275	6,290	139	31
	L1_Mus2 (B)			38.9	51/131	13.9		ChrX: 133100092-133106382	6,291	135	33
	L1_Mus4 (A)	82.7	2,578/3,116	29.9	29/97	14.1	0.098	Chr1: 23500016-23506071	6,056	132	30
	L1_Mus4 (B)			31.9	29/91	14.5		ChrX: 106536883-106542951	6,069	132	32
	L1_Mus3 (A)	82.0	1,618/1,973	18.4	7/38	16.3	0.045	ChrX: 23912936-23918989	6,054	148	31
	L1_Mus3 (B)			19.4	7/36	15.8		ChrX: 114011695-114017747	6,053	142	29
Opossum	L1-1_MD (A)	97.2	5,158/5,306	85.2	138/162	19.3	0.020	ChrX: 16824893-16831211	6,319	164	23
	L1-1_MD (B)			86.8	138/159	18.9		ChrX: 50400420-50406738	6,319	160	25
	L1_Mdo2 (A)	91.4	2,673/2,924	65.3	62/95	19.8	0.036	ChrX: 15619527-15625648	6,122	161	26
	L1_Mdo2 (B)			60.8	62/102	20.9		ChrX: 24442475-24448597	6,123	175	23
	L1_Mdo4 (A)	88.6	5,520/6,230	55.3	94/170	19.6	0.025	ChrX: 28754480-28761226	6,747	180	23
	L1_Mdo4 (B)			55.3	94/170	19.3		ChrX: 45417090-45423836	6,747	179	27
	L1_Mdo3c (A)	85.5	4,084/4,776	36.4	59/162	19.2	0.051	ChrX: 20866798-20873153	6,356	165	28
	L1_Mdo3c (B)			36.9	59/160	19.3		ChrX: 77077450-77083806	6,357	162	28
	L1_Mdo1 (A)	85.1	3,454/4,061	42.4	42/99	20.0	0.057	ChrX: 59381169-59387638	6,470	177	24
	L1_Mdo1 (B)			44.7	42/94	20.1		ChrX: 76310635-76317104	6,470	169	26
