# Supplementary material for: Convergence in LINE-1 nucleotide variations can benefit redundantly forming triplexes with lncRNA in mammalian X-chromosome inactivation
Source: Mob DNA. 2019 Jul 30;10:33. doi: 10.1186/s13100-019-0173-4 (PMC6664574; doi:10.1186/s13100-019-0173-4)
Supplement: Supplementary file 11 — Cumulative ratios of r-UC (TC)/r-AG motifs including overlaps in the paired L1s and XIST/Xist/Rsx RNAs. The cumulative ratios for 5–7-nucleotide motifs are shown in red. *No. represents motif number including overlaps. These three paired L1s were used in Additional file 9. (PDF 4301 kb) [file 13100_2019_173_MOESM11_ESM.pdf]

Additional file 11: Cumulative ratios of r-UC(TC)/r-AG motifs including overlaps in the paired L1s and XIST/Xist/Rsx RNAs

The cumulative ratios for 5–7-nucleotide motifs are shown in red. *No. represents motif number including overlaps. These three paired L1s were used in Additional file 9.

Human L1PA5 (A) (hg38 assembly)

ChrX:69350758-69356897

nt	r-AG	No.	*No.	Ratio	Cumulative ratio
5	AAAGA	14	41	11.45	11.45
5	AAGGA	4	21	5.87	17.32
5	AGAAA	11	24	6.70	24.02
5	AGAGA	5	24	6.70	30.73
5	AGGAA	4	21	5.87	36.59
6	AAAGAA	2	12	3.35	39.94
6	AAAGAG	4	12	3.35	43.30
6	AAAGGA	3	7	1.96	45.25
6	AAGAAA	7	17	4.75	50.00
6	AGAAAG	1	5	1.40	51.40
6	AGAAGA	1	10	2.79	54.19
6	AGAGAA	2	14	3.91	58.10
6	AGAGAG	1	3	0.84	58.94
6	AGGAAA	3	21	5.87	64.80
6	AGGAAG	1	9	2.51	67.32
6	AGGAGA	2	8	2.23	69.55
6	GAAGGA	2	8	2.23	71.79
6	GAGAGA	1	3	0.84	72.63
7	AAAGAAA	4	5	1.40	74.02
7	AAAGAAG	1	4	1.12	75.14
7	AAAGAGA	2	5	1.40	76.54
7	AAGAGAA	1	6	1.68	78.21
7	AGAGAAA	2	3	0.84	79.05
7	AGAGAAG	2	6	1.68	80.73
7	AGGAGAA	1	5	1.40	82.12
7	GAAAGAA	1	2	0.56	82.68
7	GGAAGAA	1	5	1.40	84.08
7	GGAGAAA	2	4	1.12	**85.20**
8	AAAGAAGA	1	2	0.56	85.75
8	AAAGAAGG	1	1	0.28	86.03
8	AAAGAGAA	1	2	0.56	86.59
8	AAAGAGGA	1	2	0.56	87.15
8	AAGAAGAG	1	2	0.56	87.71
8	AAGGAGAA	1	3	0.84	88.55
8	AGAAAGGA	1	1	0.28	88.83
8	AGAAGAAA	2	3	0.84	89.66
8	AGAGGAAG	1	1	0.28	89.94
8	AGGAAAGA	1	2	0.56	90.50
8	AGGAAGAA	1	3	0.84	91.34
8	GAAGAGAA	1	2	0.56	91.90
8	GAAGAGAG	1	1	0.28	92.18
8	GAAGGAAA	1	2	0.56	92.74
8	GAAGGAAG	1	1	0.28	93.02
8	GAAGGAGA	1	3	0.84	93.85
8	GAGAAGAG	1	1	0.28	94.13
8	GGAAAGGA	1	2	0.56	94.69
8	GGAGAGGA	1	1	0.28	94.97
9	AAAGAGAAG	1	1	0.28	95.25
9	AAGAGAAAG	1	1	0.28	95.53
9	AAGGAGGAG	1	1	0.28	95.81
9	AGAAGGAAA	1	1	0.28	96.09
9	AGGAAGAAA	1	1	0.28	96.37
9	AGGAAGAAG	1	1	0.28	96.65
9	GGAAAGGAA	1	1	0.28	96.93
9	GGAGGAAGA	1	1	0.28	97.21
10	AAAGAAGAAA	1	1	0.28	97.49
10	AGAGGAAAGA	1	1	0.28	97.77
10	GAAGGAGAAA	1	1	0.28	98.04
10	GGAAGAAAGG	1	1	0.28	98.32
11	AAAGGAGGAAG	1	1	0.28	98.60
11	AAGAAGAGAAG	1	1	0.28	98.88
11	AGAGAGAAGAA	1	1	0.28	99.16
11	AGGAAGGAGAA	1	1	0.28	99.44
12	AGGAGAAAGAAA	1	1	0.28	99.72
12	GAGAGAAGAAGG	1	1	0.28	100.00
	Total	123	358	100.00	

Human L1PA5 (B) (hg38 assembly)

ChrX:85583162-85589299

nt	r-AG	No.	*No.	Ratio	Cumulative ratio
5	AAAGA	12	37	9.16	9.16
5	AAGAA	2	33	8.17	17.33
5	AAGGA	4	24	5.94	23.27
5	AGAAA	9	35	8.66	31.93
5	AGAGA	7	24	5.94	37.87
5	AGGAA	3	22	5.45	43.32
6	AAAGAA	2	13	3.22	46.53
6	AAAGAG	2	10	2.48	49.01
6	AAAGGA	1	9	2.23	51.24
6	AAGAAA	4	16	3.96	55.20
6	AGAAAG	3	11	2.72	57.92
6	AGAAGA	1	13	3.22	61.14
6	AGAGAA	3	11	2.72	63.86
6	AGGAAA	3	9	2.23	66.09
6	AGGAGA	3	8	1.98	68.07
6	GAAGGA	2	7	1.73	69.80
6	GAGAGA	1	5	1.24	71.04
7	AAAGAAA	2	6	1.49	72.52
7	AAAGAAG	1	5	1.24	73.76
7	AAAGAGA	1	3	0.74	74.50
7	AAAGGAA	3	4	0.99	75.50
7	AAGGAAA	1	3	0.74	76.24
7	AAGGAGA	1	3	0.74	76.98
7	AAGGAGG	1	2	0.50	77.48
7	AGAGAAA	1	2	0.50	77.97
7	AGAGAAG	1	4	0.99	78.96
7	AGAGAGA	1	3	0.74	79.70
7	AGAGGAG	1	1	0.25	79.95
7	AGGAGAA	1	4	0.99	80.94
7	GAAGAAA	1	6	1.49	82.43
7	GAAGAGA	1	5	1.24	83.66
7	GGAAGAA	1	4	0.99	84.65
7	GGAGAAA	2	4	0.99	85.64
7	GGAGGAA	1	3	0.74	**86.39**
8	AAAGAAAG	1	2	0.50	86.88
8	AAAGAAGA	1	3	0.74	87.62
8	AAAGAGAA	1	1	0.25	87.87
8	AAGAAGAG	1	2	0.50	88.37
8	AAGAAGGA	1	3	0.74	89.11
8	AAGGAGAA	1	2	0.50	89.60
8	AGAAAGGA	1	1	0.25	89.85
8	AGAAGAAA	2	4	0.99	90.84
8	AGAGGAAA	1	1	0.25	91.09
8	AGAGGAAG	1	1	0.25	91.34
8	AGGAAAGA	1	1	0.25	91.58
8	AGGAAGAA	1	2	0.50	92.08
8	GAAGAGAG	1	2	0.50	92.57
8	GAAGGAAA	1	1	0.25	92.82
8	GAAGGAAG	1	1	0.25	93.07
8	GAGAAGAG	1	2	0.50	93.56
8	GGAAAGGA	1	2	0.50	94.06
8	GGAGAGGA	1	1	0.25	94.31
9	AAGAAGAAA	1	2	0.50	94.80
9	AAGAAGGAA	1	1	0.25	95.05
9	AAGAAGGAG	1	1	0.25	95.30
9	AGAAAGAAA	1	2	0.50	95.79
9	AGAAAGAGG	1	1	0.25	96.04
9	AGAAGAGAG	1	1	0.25	96.29
9	AGGAAGAAG	1	1	0.25	96.53
9	GAAAGAAAG	1	1	0.25	96.78
9	GAAAGAGGA	1	1	0.25	97.03
9	GGAGGAAGA	1	1	0.25	97.28
10	AAAGAAGAAA	1	1	0.25	97.52
10	AAAGGAGAAA	1	1	0.25	97.77
10	GGAAAGGAAA	1	1	0.25	98.02
11	AAAGAAGAGAA	1	1	0.25	98.27
11	AAAGGAGGAAA	1	1	0.25	98.51
11	AGAGAGAAAGG	1	1	0.25	98.76
11	AGAGAGAAGAA	1	1	0.25	99.01
11	GAGAAGAGAAG	1	1	0.25	99.26
11	GGAAGAAAGAG	1	1	0.25	99.50
12	AGGAGAAAGAAA	1	1	0.25	99.75
12	GAGAGAAGAAGG	1	1	0.25	100.00
	Total	123	404	100.00	

XIST RNA

NR_001564

nt	r-UC	No.	*No.	Ratio	Cumulative ratio
5	tcctt	3	50	5.15	5.15
5	tctct	5	46	4.74	9.90
5	tcttt	22	75	7.73	17.63
5	ttcct	7	56	5.77	23.40
5	ttctt	9	61	6.29	29.69
5	tttct	20	82	8.45	38.14
6	ctcctt	3	13	1.34	39.48
6	ctctct	2	11	1.13	40.62
6	ctcttt	3	21	2.16	42.78
6	cttcct	4	21	2.16	44.95
6	cttctt	1	11	1.13	46.08
6	ctttct	6	20	2.06	48.14
6	tcctct	2	7	0.72	48.87
6	tccttc	1	7	0.72	49.59
6	tccttt	4	23	2.37	51.96
6	tctctc	2	9	0.93	52.89
6	tctctt	2	20	2.06	54.95
6	tctttc	1	13	1.34	56.29
6	ttcctc	1	9	0.93	57.22
6	ttcctt	4	26	2.68	59.90
6	ttctct	5	21	2.16	62.06
6	ttcttt	7	30	3.09	65.15
6	tttcct	6	19	1.96	67.11
6	tttctc	5	17	1.75	68.87
6	tttctt	9	33	3.40	72.27
7	cctcctt	1	3	0.31	72.58
7	cctcttt	7	8	0.82	73.40
7	ccttctt	1	3	0.31	73.71
7	ctctcct	1	5	0.52	74.23
7	ctctctc	1	3	0.31	74.54
7	ctctctt	1	5	0.52	75.05
7	ctctttc	1	3	0.31	75.36
7	cttcctc	2	4	0.41	75.77
7	cttcctt	1	10	1.03	76.80
7	cttctct	1	6	0.62	77.42
7	ctttcct	1	5	0.52	77.94
7	ctttctt	1	6	0.62	78.56
7	tccttct	1	5	0.52	79.07
7	tctcctt	1	7	0.72	79.79
7	tctctcc	1	3	0.31	80.10
7	tctctct	1	3	0.31	80.41
7	tctcttt	2	9	0.93	81.34
7	tcttctt	1	5	0.52	81.86
7	tctttcc	1	2	0.21	82.06
7	ttcctcc	2	3	0.31	82.37
7	ttcctct	1	1	0.10	82.47
7	ttccttt	1	14	1.44	83.92
7	ttctcct	2	5	0.52	84.43
7	tttcctt	3	11	1.13	85.57
7	tttctcc	1	2	0.21	85.77
7	tttctct	2	9	0.93	86.70
7	tttcttc	1	3	0.31	87.01
7	tttcttt	11	17	1.75	**88.76**
8	cctcctct	1	1	0.10	88.87
8	cctctctt	1	3	0.31	89.18
8	ccttcctc	1	1	0.10	89.28
8	ccttcttt	1	2	0.21	89.48
8	cctttctc	1	3	0.31	89.79
8	ctcctctt	1	1	0.10	89.90
8	ctctcctt	1	3	0.31	90.21
8	ctctcttt	1	3	0.31	90.52
8	ctcttctt	1	3	0.31	90.82
8	cttctcct	1	1	0.10	90.93
8	cttctctc	1	1	0.10	91.03
8	cttcttcc	1	1	0.10	91.13
8	ctttcctt	2	4	0.41	91.55
8	ctttctct	1	3	0.31	91.86
8	tcctcctt	1	1	0.10	91.96
8	tcctctcc	1	1	0.10	92.06
8	tcctcttt	1	1	0.10	92.16
8	tctccttt	1	3	0.31	92.47
8	tctcttct	1	1	0.10	92.58
8	tctctttc	1	2	0.21	92.78
8	tcttcctc	1	1	0.10	92.89
8	tcttctcc	1	1	0.10	92.99
8	tctttctt	1	3	0.31	93.30
8	ttctttct	1	2	0.21	93.51
8	tttccttt	2	5	0.52	94.02
8	tttctctt	1	5	0.52	94.54
8	tttctttc	3	4	0.41	94.95
9	cctctctct	1	1	0.10	95.05
9	cctctcttt	2	2	0.21	95.26
9	cctcttcct	2	2	0.21	95.46
9	cctttcttc	1	1	0.10	95.57
9	cttcctttc	1	7	0.72	96.29
9	ctttccttt	1	2	0.21	96.49
9	ctttctcct	1	1	0.10	96.60
9	tctccttct	1	1	0.10	96.70
9	tctctcctt	1	1	0.10	96.80
9	tctttcttt	1	2	0.21	97.01
9	ttctccttt	1	1	0.10	97.11
9	tttctcttt	2	3	0.31	97.42
9	tttcttcct	1	1	0.10	97.53
10	cctcctttct	1	1	0.10	97.63
10	cctcttcttt	1	1	0.10	97.73
10	ccttcctttc	1	1	0.10	97.84
10	ccttctcttc	1	1	0.10	97.94
10	cctttccttt	1	1	0.10	98.04
10	cctttctctt	1	1	0.10	98.14
10	ctcttctttc	1	1	0.10	98.25
10	tccttccttt	1	1	0.10	98.35
10	tccttctctt	1	2	0.21	98.56
10	tcctttcttt	1	1	0.10	98.66
10	tttcctttct	1	1	0.10	98.76
11	cttccttcttt	1	1	0.10	98.87
11	cttcttctctt	1	1	0.10	98.97
11	tcttcctttcc	5	5	0.52	99.48
11	ttcttctttcc	1	1	0.10	99.59
12	tttccttctctt	1	1	0.10	99.69
14	cctctcctttctct	1	1	0.10	99.79
14	tctctttctttctt	1	1	0.10	99.90
15	ttctctctctcctcc	1	1	0.10	100.00
	Total	255	970	100.00	

Mouse L1Md_T (A) (mm10 assembly)

ChrX:1527595-21533712

nt	r-AG	No.	*No.	Ratio	Cumulative ratio
5	AAAGA	10	39	8.39	8.39
5	AAGAA	5	35	7.53	15.91
5	AAGGA	4	28	6.02	21.94
5	AGAAA	15	42	9.03	30.97
5	AGAGA	5	21	4.52	35.48
5	AGGAA	3	27	5.81	41.29
6	AAAGAA	5	19	4.09	45.38
6	AAAGAG	3	7	1.51	46.88
6	AAAGGA	5	14	3.01	49.89
6	AAGAAG	1	7	1.51	51.40
6	AAGGAA	1	11	2.37	53.76
6	AAGGAG	1	5	1.08	54.84
6	AGAAAG	1	13	2.80	57.63
6	AGAAGA	3	9	1.94	59.57
6	AGAGAA	1	9	1.94	61.51
6	AGAGAG	2	6	1.29	62.80
6	AGAGGA	1	6	1.29	64.09
6	AGGAAA	4	10	2.15	66.24
6	AGGAGA	1	4	0.86	67.10
6	GAAAGA	1	14	3.01	70.11

Mouse L1Md_T (B) (mm10 assembly)

ChrX:29660434-29666551

nt	r-AG	No.	*No.	Ratio	Cumulative ratio
5	AAAGA	10	40	9.48	9.48
5	AAGAA	6	41	9.72	19.19
5	AAGGA	2	19	4.50	23.70
5	AGAAA	13	42	9.95	33.65
5	AGAGA	8	22	5.21	38.86
5	AGGAA	3	18	4.27	43.13
6	AAAGAA	6	20	4.74	47.87
6	AAAGAG	1	4	0.95	48.82
6	AAAGGA	3	9	2.13	50.95
6	AAGAAA	1	18	4.27	55.21
6	AAGAAG	1	8	1.90	57.11
6	AAGAGA	1	4	0.95	58.06
6	AAGGAG	1	5	1.18	59.24
6	AGAAAG	1	11	2.61	61.85
6	AGAAGA	3	10	2.37	64.22
6	AGAGAA	2	9	2.13	66.35
6	AGAGGA	2	8	1.90	68.25
6	AGGAAA	3	8	1.90	70.14
6	AGGAAG	1	4	0.95	71.09
6	AGGAGA	1	4	0.95	72.04

Xist RNA

NR_001463

nt	r-UC	No.	*No.	Ratio	Cumulative ratio
5	TCCTT	6	57	5.68	5.68
5	TCTCT	2	32	3.19	8.87
5	TCTTT	14	60	5.98	14.86
5	TTCCT	2	53	5.28	20.14
5	TTCTT	9	75	7.48	27.62
5	TTTCT	22	90	8.97	36.59
6	CTCTCT	1	6	0.60	37.19
6	CTCTTT	3	13	1.30	38.48
6	CTTCCT	1	32	3.19	41.67
6	CTTTCT	3	22	2.19	43.87
6	TCCTCT	1	4	0.40	44.27
6	TCCTTC	2	8	0.80	45.06
6	TCCTTT	2	15	1.50	46.56
6	TCTCTC	1	5	0.50	47.06
6	TCTCTT	3	18	1.79	48.85
6	TCTTCT	1	5	0.50	49.35
6	TCTTTC	1	16	1.60	50.95
6	TTCCTC	1	8	0.80	51.74
6	TTCCTT	2	36	3.59	55.33
6	TTCTCT	2	16	1.60	56.93

6	GAGAAA	2	12	2.58	72.69
6	GAGGAA	2	6	1.29	73.98
7	AAAGAAA	3	9	1.94	75.91
7	AAAGAGA	1	3	0.65	76.56
7	AAAGGAA	1	6	1.29	77.85
7	AAGAAAG	2	6	1.29	79.14
7	AAGAAGG	1	2	0.43	79.57
7	AAGAGAA	1	2	0.43	80.00
7	AAGGAAA	1	3	0.65	80.65
7	AAGGAAG	1	6	1.29	81.94
7	AAGGAGG	2	2	0.43	82.37
7	AGAAAGG	1	1	0.22	82.58
7	AGAAGAA	1	3	0.65	83.23
7	AGAGAAA	2	5	1.08	84.30
7	AGAGAAG	1	2	0.43	84.73
7	AGAGAGG	1	2	0.43	85.16
7	AGAGGAA	1	4	0.86	86.02
7	AGGAAAG	1	2	0.43	86.45
7	AGGAGAG	1	1	0.22	86.67
7	GAAAGGA	1	3	0.65	87.31
7	GAAGAAA	1	6	1.29	88.60
7	GAGAAAG	1	5	1.08	89.68
7	GAGAAGA	1	1	0.22	89.89
7	GGAAAGA	2	6	1.29	91.18
7	GGAGAAA	1	3	0.65	**91.83**
8	AAAGAAGA	2	2	0.43	92.26
8	AAAGGAAA	1	1	0.22	92.47
8	AAAGGAAG	3	4	0.86	93.33
8	AAAGGAGA	1	1	0.22	93.55
8	AAGAAGAA	1	1	0.22	93.76
8	AAGAGAAA	1	1	0.22	93.98
8	AAGGAAGA	1	2	0.43	94.41
8	AGAAGAAA	1	1	0.22	94.62
8	AGAGAAGG	1	1	0.22	94.84
8	AGAGAGAG	1	1	0.22	95.05
8	AGAGGAAG	1	1	0.22	95.27
8	AGGAAGGA	1	1	0.22	95.48
8	GGAAAGAA	1	3	0.65	96.13
8	GGAAAGGA	1	2	0.43	96.56
8	GGAAGGAG	1	1	0.22	96.77
9	AAGAGGAAA	1	1	0.22	96.99
9	GGAAAGAAG	1	1	0.22	97.20
10	AAGAAGGAAA	1	1	0.22	97.42
10	AGAGAAAGAA	1	1	0.22	97.63
10	AGGAAAGAAA	1	1	0.22	97.85
10	GAGAAAGAAA	1	1	0.22	98.06
10	GGAAGAAAGA	1	1	0.22	98.28
11	AAAGAAAGAGA	1	1	0.22	98.49
11	AGAGGAGAAAG	1	1	0.22	98.71
11	GAAGAAAGAAA	2	2	0.43	99.14
11	GGAAAGAGAGG	1	1	0.22	99.35
11	GGAGAGAGAAA	1	1	0.22	99.57
13	GGAAAGGAAGAAA	1	1	0.22	99.78
13	GGAGAAAGAGGAA	1	1	0.22	100.00
	Total	138	465	100.00	

6	GAAAGA	1	11	2.61	74.64
6	GAGAAA	2	10	2.37	77.01
7	AAAGAAA	7	12	2.84	79.86
7	AAAGGAA	1	2	0.47	80.33
7	AAGAAGG	3	4	0.95	81.28
7	AAGAGAG	1	3	0.71	81.99
7	AAGGAAA	2	3	0.71	82.70
7	AAGGAAG	1	2	0.47	83.18
7	AGAAAGA	1	6	1.42	84.60
7	AGAAGAA	3	5	1.18	85.78
7	AGAAGAG	1	1	0.24	86.02
7	AGAGAAA	2	3	0.71	86.73
7	AGAGAAG	1	2	0.47	87.20
7	GAAAGGA	1	3	0.71	87.91
7	GAAGAAA	3	6	1.42	89.34
7	GGAAAGA	1	4	0.95	90.28
7	GGAGAAA	1	3	0.71	**91.00**
8	AAAGAAAG	1	2	0.47	91.47
8	AAAGAAGA	1	1	0.24	91.71
8	AAAGGAAA	1	1	0.24	91.94
8	AAAGGAGA	1	1	0.24	92.18
8	AAGAAGAA	1	2	0.47	92.65
8	AAGAAGGA	1	1	0.24	92.89
8	AAGAGGAA	1	3	0.71	93.60
8	AAGAGGAG	1	1	0.24	93.84
8	AGAAGGAG	1	1	0.24	94.08
8	AGAGAAAG	1	1	0.24	94.31
8	AGAGAGAG	1	1	0.24	94.55
8	GAGAAAGG	1	1	0.24	94.79
8	GGAAAGAA	1	2	0.47	95.26
8	GGAAAGGA	1	2	0.47	95.73
8	GGAAGGAG	1	1	0.24	95.97
8	GGAGAGAG	1	1	0.24	96.21
9	AAGAAGAAA	1	1	0.24	96.45
9	AAGAGGAAA	1	1	0.24	96.68
9	AGGAGGAGG	1	1	0.24	96.92
9	GGAGAGAAG	1	1	0.24	97.16
10	AAGGAAGGAA	1	1	0.24	97.39
10	AGGAAAGAAA	1	1	0.24	97.63
10	GAGAAAGAAA	1	1	0.24	97.87
10	GGAAGAAAGA	1	1	0.24	98.10
11	AAAGAAAGAAA	1	1	0.24	98.34
11	AGAGGAGAAAG	1	1	0.24	98.58
11	AGAGGAGAGAA	1	1	0.24	98.82
11	GAAGAAAGAAA	1	1	0.24	99.05
11	GGAAAGAGAGG	1	1	0.24	99.29
11	GGAAGAGAGAA	1	1	0.24	99.53
13	GGAAAGGAGGAAG	1	1	0.24	99.76
13	GGAGAAAGAGGAA	1	1	0.24	100.00
	Total	133	422	100.00	

6	TTCTTT	13	30	2.99	59.92
6	TTTCCT	2	12	1.20	61.12
6	TTTCTC	1	12	1.20	62.31
6	TTTCTT	6	48	4.79	67.10
7	CCTCTTT	1	2	0.20	67.30
7	CTCCTTC	1	2	0.20	67.50
7	CTTCCTT	1	24	2.39	69.89
7	CTTCTCT	2	5	0.50	70.39
7	CTTCTTC	1	3	0.30	70.69
7	CTTTCCT	1	6	0.60	71.29
7	CTTTCTT	2	10	1.00	72.28
7	TCCTTCT	1	2	0.20	72.48
7	TCTCCTT	1	6	0.60	73.08
7	TCTCTCC	1	3	0.30	73.38
7	TCTCTTT	4	8	0.80	74.18
7	TCTTCCT	2	27	2.69	76.87
7	TCTTTCT	2	6	0.60	77.47
7	TTCCTTC	1	3	0.30	77.77
7	TTCCTTT	3	10	1.00	78.76
7	TTCTTCC	1	21	2.09	80.86
7	TTTCCTC	1	3	0.30	81.16
7	TTTCCTT	1	6	0.60	81.75
7	TTTCTTC	1	20	1.99	83.75
7	TTTCTTT	4	14	1.40	**85.14**
8	CCTTCCTC	1	2	0.20	85.34
8	CCTTTCTC	2	2	0.20	85.54
8	CCTTTCTT	1	3	0.30	85.84
8	CTCTCTTT	1	2	0.20	86.04
8	CTTCTCTT	1	3	0.30	86.34
8	CTTCTTTC	1	3	0.30	86.64
8	CTTTCCTC	1	2	0.20	86.84
8	CTTTCTCT	3	3	0.30	87.14
8	CTTTCTTT	1	5	0.50	87.64
8	TCCTCTTT	1	1	0.10	87.74
8	TCTCTCTT	1	1	0.10	87.84
8	TCTTCCTT	3	22	2.19	90.03
8	TCTTCTCC	1	3	0.30	90.33
8	TTCTCCTC	1	1	0.10	90.43
8	TTCTCCTT	1	3	0.30	90.73
8	TTCTCTTT	1	2	0.20	90.93
8	TTTCCTTT	1	4	0.40	91.33
8	TTTCTCCT	1	1	0.10	91.43
8	TTTCTCTT	1	2	0.20	91.63
8	TTTCTTCC	1	17	1.69	93.32
8	TTTCTTCT	1	2	0.20	93.52
8	TTTCTTTC	3	8	0.80	94.32
9	CCTCTCTTT	1	1	0.10	94.42
9	CCTCTTCCT	1	2	0.20	94.62
9	CCTTCTCTT	1	1	0.10	94.72
9	CCTTCTTCC	1	1	0.10	94.82
9	CTTCTCTTC	1	1	0.10	94.92
9	CTTTCCTTT	1	3	0.30	95.21
9	CTTTCTTTC	1	3	0.30	95.51
9	TCCTTTCTT	1	1	0.10	95.61
9	TCTTCCTTT	1	1	0.10	95.71
9	TTCTCTTTC	1	1	0.10	95.81
9	TTCTTTCTT	1	3	0.30	96.11
9	TTTCCTTCC	1	1	0.10	96.21
9	TTTCTCTCC	1	1	0.10	96.31
9	TTTCTCTTC	1	1	0.10	96.41
9	TTTCTTTCC	1	1	0.10	96.51
9	TTTCTTTCT	1	2	0.20	96.71
10	CCTCCTTTCC	1	1	0.10	96.81
10	CCTCTTCTCC	1	1	0.10	96.91
10	CCTTCCTTTC	1	1	0.10	97.01
10	CCTTCTCCTT	1	1	0.10	97.11
10	CCTTTCTTTC	1	1	0.10	97.21
10	CTTCTTCCTT	1	1	0.10	97.31
10	TTCTCTCTCC	1	1	0.10	97.41
10	TTCTTCCTCC	1	1	0.10	97.51
10	TTTCTTCCTT	15	16	1.60	99.10
11	CTCTCTTCCTT	1	1	0.10	99.20
12	CCTTCTTTCTTT	1	1	0.10	99.30
12	TCTTTCTTTCTT	1	1	0.10	99.40
12	TTTCTTCTCCTT	1	1	0.10	99.50
13	TCTCCTTTCCTTT	1	1	0.10	99.60
14	CTTTCCTCCTTTCT	1	1	0.10	99.70
15	CCTTCCTCTTCCTCT	1	1	0.10	99.80
15	TTTCTTCCTTCCTTT	1	1	0.10	99.90
16	TCTCCTTCTTTCCTTT	1	1	0.10	100.00
	Total	210	1003	100.00	

Opossum L1_Mdo4 (A)(monDom5 assembly)

ChrX:28754480-28761226

nt	r-AG	No.	*No.	Ratio	Cumulative ratio
5	AAAGA	6	45	7.25	7.25
5	AAGAA	4	47	7.57	14.81
5	AAGGA	5	29	4.67	19.48
5	AGAAA	13	43	6.92	26.41
5	AGAGA	5	24	3.86	30.27
5	AGGAA	6	33	5.31	35.59
5	AGGGA	2	17	2.74	38.33
6	AAAGAA	3	22	3.54	41.87
6	AAAGAG	1	11	1.77	43.64
6	AAAGGA	3	15	2.42	46.05
6	AAGAAA	5	21	3.38	49.44
6	AAGAAG	2	14	2.25	51.69
6	AAGAGA	4	11	1.77	53.46
6	AAGGAA	1	11	1.77	55.23
6	AAGGGA	1	6	0.97	56.20
6	AGAAAG	2	8	1.29	57.49
6	AGAAGA	2	10	1.61	59.10
6	AGAGAA	1	6	0.97	60.06
6	AGAGGA	3	10	1.61	61.67
6	AGGAAA	5	18	2.90	64.57
6	AGGAAG	1	5	0.81	65.38
6	GAAAGA	3	12	1.93	67.31
6	GAAGGA	1	6	0.97	68.28
6	GAGAAA	3	7	1.13	69.40
7	AAAGAAA	7	13	2.09	71.50
7	AAAGAGA	2	4	0.64	72.14
7	AAAGAGG	1	6	0.97	73.11
7	AAAGGAA	2	5	0.81	73.91
7	AAGAAGG	1	6	0.97	74.88
7	AAGAGGA	1	4	0.64	75.52
7	AAGGAAA	2	6	0.97	76.49
7	AAGGAGA	1	4	0.64	77.13
7	AAGGGAA	1	3	0.48	77.62
7	AGAAGAA	1	6	0.97	78.58
7	AGAGGAG	1	2	0.32	78.90
7	AGGAAAG	1	3	0.48	79.39
7	AGGAGAG	1	2	0.32	79.71
7	AGGGAAA	2	3	0.48	80.19
7	AGGGAAG	1	4	0.64	80.84
7	AGGGGAG	1	4	0.64	81.48
7	AGGGGGA	1	4	0.64	82.13
7	GAAAGAA	1	6	0.97	83.09
7	GAAAGGA	1	3	0.48	83.57
7	GAAGAAA	1	4	0.64	84.22
7	GAGGAAA	3	6	0.97	85.19
7	GAGGAGA	1	2	0.32	85.51
7	GGAAGAA	2	4	0.64	86.15
7	GGAGAAA	1	2	0.32	86.47
7	GGAGAGA	1	3	0.48	86.96
7	GGAGGAA	1	2	0.32	**87.28**
8	AAAGAAGG	1	3	0.48	87.76
8	AAAGAGGA	2	2	0.32	88.08
8	AAAGGAGA	1	4	0.64	88.73
8	AAGAAGGG	1	3	0.48	89.21
8	AAGAGAGG	1	2	0.32	89.53

Opossum L1_Mdo4 (B) (monDom5 assembly)

ChrX:45417090-45423836

nt	r-AG	No.	*No.	Ratio	Cumulative ratio
5	AAAGA	4	54	8.96	8.96
5	AAGAA	8	51	8.46	17.41
5	AAGGA	5	27	4.48	21.89
5	AGAAA	15	50	8.29	30.18
5	AGAGA	4	26	4.31	34.49
5	AGGAA	6	29	4.81	39.30
5	AGGGA	3	21	3.48	42.79
6	AAAGAA	7	29	4.81	47.60
6	AAAGAG	3	13	2.16	49.75
6	AAGAAA	4	22	3.65	53.40
6	AAGAGA	3	11	1.82	55.22
6	AAGGAA	2	12	1.99	57.21
6	AAGGGA	1	5	0.83	58.04
6	AGAAAG	2	11	1.82	59.87
6	AGAAGA	2	8	1.33	61.19
6	AGAGAA	1	9	1.49	62.69
6	AGAGGA	1	5	0.83	63.52
6	AGGAAA	2	13	2.16	65.67
6	AGGAAG	1	4	0.66	66.33
6	GAAAGA	3	11	1.82	68.16
7	AAAGAAA	9	15	2.49	70.65
7	AAAGAAG	2	7	1.16	71.81
7	AAAGAGA	2	3	0.50	72.31
7	AAAGAGG	1	6	1.00	73.30
7	AAAGGAA	2	6	1.00	74.30
7	AAAGGAG	2	7	1.16	75.46
7	AAGAGAA	1	3	0.50	75.95
7	AAGGAAA	2	7	1.16	77.11
7	AAGGGAA	1	3	0.50	77.61
7	AGAAAGA	1	5	0.83	78.44
7	AGAAGAA	1	4	0.66	79.10
7	AGAGAAA	1	4	0.66	79.77
7	AGAGAAG	1	2	0.33	80.10
7	AGAGAGA	1	2	0.33	80.43
7	AGAGGAA	1	3	0.50	80.93
7	AGGAGAA	1	2	0.33	81.26
7	AGGGAAA	2	5	0.83	82.09
7	AGGGAAG	1	5	0.83	82.92
7	AGGGAGG	1	2	0.33	83.25
7	AGGGGAG	1	2	0.33	83.58
7	GAAAGGA	1	3	0.50	84.08
7	GAAGAAA	1	5	0.83	84.91
7	GAGGAAA	3	4	0.66	85.57
7	GAGGGGA	1	5	0.83	86.40
7	GGAAGAA	2	4	0.66	87.06
7	GGAGAAA	1	3	0.50	87.56
7	GGAGGAA	1	2	0.33	**87.89**
8	AAAGAAGG	1	3	0.50	88.39
8	AAAGAGAA	1	1	0.17	88.56
8	AAAGGAAA	2	3	0.50	89.05
8	AAAGGAGA	2	3	0.50	89.55
8	AAAGGGGA	1	1	0.17	89.72
8	AAGAAGGG	1	2	0.33	90.05
8	AAGAGAAG	1	1	0.17	90.22
8	AAGAGAGG	1	2	0.33	90.55

Rsx RNA

JQ937287

nt	r-AG	No.	*No.	Ratio	Cumulative ratio
5	aaaga	45	71	2.60	2.60
5	aagaa	4	16	0.59	3.18
5	aagga	16	112	4.10	7.28
5	agaaa	20	76	2.78	10.05
5	agaga	6	39	1.43	11.48
5	aggaa	7	253	9.25	20.73
5	aggga	4	114	4.17	24.90
6	aaagaa	3	16	0.59	25.48
6	aaagga	33	55	2.01	27.50
6	aagaaa	1	31	1.13	28.63
6	aagaag	12	130	4.75	33.38
6	aagaga	6	15	0.55	33.93
6	aaggaa	7	36	1.32	35.25
6	aaggag	5	9	0.33	35.58
6	aaggga	4	101	3.69	39.27
6	agaaag	2	31	1.13	40.40
6	agaaga	13	25	0.91	41.32
6	agagaa	2	14	0.51	41.83
6	agagga	1	15	0.55	42.38
6	aggaaa	23	54	1.97	44.35
6	aggaag	5	169	6.18	50.53
6	aggaga	10	21	0.77	51.30
6	agggaa	1	12	0.44	51.74
6	agggga	1	39	1.43	53.16
6	gaaaga	1	14	0.51	53.67
6	gaagaa	3	2	0.07	53.75
6	gaagga	3	25	0.91	54.66
6	gagaaa	4	23	0.84	55.50
6	gagaga	2	9	0.33	55.83
6	gaggaa	1	31	1.13	56.97
7	aaagaaa	3	9	0.33	57.29
7	aaagaga	2	3	0.11	57.40
7	aaaggaa	1	5	0.18	57.59
7	aaaggga	8	17	0.62	58.21
7	aaggaaa	3	12	0.44	58.65
7	aaggaga	1	2	0.07	58.72
7	aaggagg	1	1	0.04	58.76
7	agaaaga	1	10	0.37	59.12
7	agagaaa	2	6	0.22	59.34
7	agagaag	3	5	0.18	59.52
7	agaggaa	1	10	0.37	59.89
7	aggaaga	1	153	5.59	65.48
7	aggagaa	1	5	0.18	65.67
7	agggaaa	1	6	0.22	65.89
7	aggggaa	1	4	0.15	66.03
7	gaagaaa	1	21	0.77	66.80
7	gaaggga	1	81	2.96	69.76
7	gagagaa	1	3	0.11	69.87
7	gaggaaa	4	14	0.51	70.38
7	gaggaga	3	4	0.15	70.53
7	gaggga	1	36	1.32	71.85
7	ggagaaa	5	13	0.48	72.32
7	ggagaga	2	4	0.15	**72.47**
8	aaaggaaa	1	1	0.04	72.50
8	aaaggaag	1	2	0.07	72.58

8	AGAAGGAG	1	1	0.16	89.69
8	AGAGAAGG	1	1	0.16	89.86
8	AGAGGAAA	1	2	0.32	90.18
8	AGAGGGAA	1	2	0.32	90.50
8	AGGGGAAA	1	3	0.48	90.98
8	GAAAGGAG	1	2	0.32	91.30
8	GAAGAAGA	1	2	0.32	91.63
8	GAGAGAAG	1	1	0.16	91.79
8	GAGAGGAG	1	1	0.16	91.95
8	GAGGAAGG	1	2	0.32	92.27
8	GGAGGGGA	2	3	0.48	92.75
8	GGGAGAGA	1	1	0.16	92.91
9	AAAGGAAAG	1	2	0.32	93.24
9	AAAGGAAGG	1	1	0.16	93.40
9	AAAGGGAGA	1	1	0.16	93.56
9	AAGAAGAAG	1	1	0.16	93.72
9	AAGAAGAGA	1	1	0.16	93.88
9	AGAAGGAAA	1	2	0.32	94.20
9	AGGGGAAAG	1	2	0.32	94.52
9	AGGGGAAGA	1	1	0.16	94.69
9	AGGGGGAAA	1	1	0.16	94.85
9	GAGGAAGGA	1	1	0.16	95.01
9	GAGGAGAGA	1	1	0.16	95.17
9	GGAGGGAAG	1	1	0.16	95.33
10	AAAGAAGGGG	1	1	0.16	95.49
10	AAAGGAGAAA	1	1	0.16	95.65
10	AAAGGAGAAG	1	1	0.16	95.81
10	AAGAAAGAGG	1	1	0.16	95.97
10	AGAAGGAAGG	1	1	0.16	96.14
10	AGAAGGGAAG	1	1	0.16	96.30
10	AGAGAAGAAA	1	1	0.16	96.46
10	AGAGAGGGAG	1	2	0.32	96.78
10	AGGGGAGGGG	1	1	0.16	96.94
10	GAAGAGGAAA	1	1	0.16	97.10
11	AAAGAAGGAAA	1	1	0.16	97.26
11	AAGAGGGGAGA	1	1	0.16	97.42
11	AGAAAGAGAAA	1	1	0.16	97.58
11	AGAAAGGAGGA	1	1	0.16	97.75
11	AGGGGGAGAGG	1	1	0.16	97.91
12	AAAGGAAAGAAA	1	1	0.16	98.07
12	AAGAGAGGGAGA	1	1	0.16	98.23
12	AAGAGGGGAAAG	1	1	0.16	98.39
13	AAAGAGGGAAGAA	1	1	0.16	98.55
13	AAGAAGAAAGAAA	1	1	0.16	98.71
13	AGAAGAAGAGGGA	1	1	0.16	98.87
13	GGGAAGGGAAAGA	1	1	0.16	99.03
14	AAGAAGGGAGGGGG	1	1	0.16	99.19
14	GAAAGAAGAAAGGG	1	1	0.16	99.36
14	GGGAGGGGGAAGAA	1	1	0.16	99.52
15	AAAGAGAGAAAGAAA	1	1	0.16	99.68
18	AAAGAGGGGGGAAAGAAA	1	1	0.16	99.84
19	GGAGGAGGGGAGGAGGAAA	1	1	0.16	100.00
	Total	180	621	100.00	

8	AGAAAGAG	1	2	0.33	90.88
8	AGAGGGAA	1	3	0.50	91.38
8	AGGGAAGG	1	1	0.17	91.54
8	GAAAGGAG	1	2	0.33	91.87
8	GAAGAAGA	1	1	0.17	92.04
8	GAGAAAGG	1	1	0.17	92.21
8	GAGAGGAG	1	1	0.17	92.37
8	GGAGAGAG	1	2	0.33	92.70
8	GGAGGGGA	2	2	0.33	93.03
8	GGGAGAGA	1	2	0.33	93.37
9	AAAGAAAGG	1	1	0.17	93.53
9	AAGAAGAAG	1	1	0.17	93.70
9	AAGAAGAGA	1	1	0.17	93.86
9	AGAAGGAAA	1	2	0.33	94.20
9	AGGGAGAAA	1	1	0.17	94.36
9	AGGGGAAAG	1	1	0.17	94.53
9	AGGGGGAAA	1	1	0.17	94.69
9	GAGGAGAGA	1	1	0.17	94.86
9	GGAGAGAAA	1	1	0.17	95.02
10	AAAGAAGGGA	1	1	0.17	95.19
10	AAAGGAGAAA	1	1	0.17	95.36
10	AAGAAAGAGG	1	1	0.17	95.52
10	AAGAGGGAAA	1	2	0.33	95.85
10	AAGGGGAAGG	1	1	0.17	96.02
10	AGGGAGGGGG	1	1	0.17	96.19
10	GAAGAGGAAA	1	1	0.17	96.35
10	GGGGAGGAAG	1	1	0.17	96.52
11	AAAGAAGGAAA	1	1	0.17	96.68
11	AAAGAGGGAAA	1	1	0.17	96.85
11	AAGAGGGGAGA	1	1	0.17	97.01
11	AAGGGGGGGAA	1	1	0.17	97.18
11	AGAAAGGAGGA	1	1	0.17	97.35
11	AGAAAGGGAAG	1	1	0.17	97.51
11	AGAGAAAGAAA	1	1	0.17	97.68
11	AGAGAGAGAAA	1	1	0.17	97.84
11	GAAAGAAGAAA	1	1	0.17	98.01
11	GGGGAGGGAAG	1	2	0.33	98.34
12	AAAGAGGGGAAA	1	1	0.17	98.51
12	AAAGGAAAGAAA	1	1	0.17	98.67
12	AGAGGGGGAGGA	1	1	0.17	98.84
13	AAAGAGGAAGAAA	1	1	0.17	99.00
13	AAAGGAAGGAGGA	1	1	0.17	99.17
13	AAGAAGAAAGAAA	1	1	0.17	99.34
13	GGGAAGGGAAAGA	1	1	0.17	99.50
14	GGGAGGGGGGAGAA	1	1	0.17	99.67
15	AAGAGAGGGAGAGAG	1	1	0.17	99.83
21	GGGAGGGGGGGAGGGAAGAAA	1	1	0.17	100.00
	Total	179	603	100.00	

8	aaagggaa	2	4	0.15	72.72
8	aagagaaa	1	2	0.07	72.80
8	aagaggaa	1	6	0.22	73.02
8	agaagaag	1	1	0.04	73.05
8	agaaggga	1	72	2.63	75.69
8	agaggaaa	1	5	0.18	75.87
8	aggaaagg	1	5	0.18	76.05
8	aggaagaa	1	115	4.20	80.26
8	gaaaggaa	1	1	0.04	80.29
8	gaagaaga	1	6	0.22	80.51
8	gaaggaga	1	1	0.04	80.55
8	gagggaaa	2	3	0.11	80.66
8	gagggaag	1	2	0.07	80.73
8	ggaagaaa	2	16	0.59	81.32
8	ggaagaag	5	109	3.99	85.30
8	ggaaggaa	4	12	0.44	85.74
8	ggaggaaa	2	5	0.18	85.92
9	aagaggaaa	2	4	0.15	86.07
9	agaaagaag	1	1	0.04	86.11
9	agagagaaa	1	1	0.04	86.14
9	agagaggag	1	2	0.07	86.22
9	agagggaga	1	1	0.04	86.25
9	aggaaagga	1	4	0.15	86.40
9	aggaagaaa	1	12	0.44	86.84
9	aggaagaag	19	102	3.73	90.57
9	aggaagagg	3	35	1.28	91.85
9	aggagaaag	2	4	0.15	91.99
9	aggaggaaa	1	2	0.07	92.07
9	aggaggaag	1	8	0.29	92.36
9	agggggaaa	2	3	0.11	92.47
9	gagagggaa	1	2	0.07	92.54
9	ggaaggaaa	6	8	0.29	92.83
10	aaagaaagga	1	3	0.11	92.94
10	aagagaaggg	1	1	0.04	92.98
10	aaggggaaa	1	1	0.04	93.02
10	agagaagaaa	1	1	0.04	93.05
10	agaggggaaa	1	2	0.07	93.13
10	aggaagaaag	4	10	0.37	93.49
10	aggaaggaaa	1	2	0.07	93.56
10	gaagaaggaa	2	9	0.33	93.89
10	gagagaaaga	1	1	0.04	93.93
10	gagagggaaa	1	1	0.04	93.97
10	gaggaagaag	1	3	0.11	94.08
10	ggaagggaaa	1	1	0.04	94.11
11	aaaggaagaga	1	1	0.04	94.15
11	aagaagagaaa	1	1	0.04	94.19
11	agggggaaga	1	1	0.04	94.22
11	gagaagaaaga	3	3	0.11	94.33
11	ggaaagggaaa	1	1	0.04	94.37
11	ggagagaggag	1	1	0.04	94.41
12	aagaggaaagga	2	2	0.07	94.48
12	aagaggaagaaa	1	1	0.04	94.52
12	agaaagaaagga	2	2	0.07	94.59
12	agaggaagaaga	1	1	0.04	94.63
12	agagggaggaaa	1	1	0.04	94.66
12	aggaagaaagga	5	6	0.22	94.88
12	aggaagaagaga	2	2	0.07	94.95
12	aggaagaaggaa	7	7	0.26	95.21
12	aggaagaaggga	67	67	2.45	97.66
12	aggaagaagggg	4	4	0.15	97.81
12	aggaagaggaga	1	1	0.04	97.84
12	aggaagagggga	31	31	1.13	98.98
12	aggagaaaggga	2	2	0.07	99.05
12	aggaggaaagga	1	1	0.04	99.09
12	aggaggaaggga	7	7	0.26	99.34
12	ggaaagggaaa	1	1	0.04	99.38
12	ggaggaaggaaa	1	1	0.04	99.41
12	gggaagaaagga	2	2	0.07	99.49
12	gggaagaaggga	1	1	0.04	99.52
12	gggagaaaggga	2	2	0.07	99.60
13	aagaggggagaaa	1	1	0.04	99.63
13	aggaaagaaggga	3	3	0.11	99.74
13	aggaagaaaggag	1	1	0.04	99.78
13	ggaaagaaagaaa	1	1	0.04	99.82
13	ggaagaggggaaa	1	1	0.04	99.85
13	gggaggaagaaga	1	1	0.04	99.89
14	ggagagaggaagag	1	1	0.04	99.93
16	agaggagggaagaaga	1	1	0.04	99.96
17	gaggggagaaagagagg	1	1	0.04	100.00
	Total	542	2735	100.00	
